# Calculating Optimal Patient to Nursing Capacity: Comparative Analysis of Traditional and New Methods

**DOI:** 10.2196/59619

**Published:** 2024-11-22

**Authors:** Anna Ware, Terri Blumke, Peter Hoover, David Arreola

**Affiliations:** 1 National Center for Collaborative Healthcare Innovation, Veterans Affairs Palo Alto Health Care System, Palo Alto, CA, United States

**Keywords:** nurse scheduling, nurse, patient ratio, nursing hours per patient day, NHPPD, nursing administration, workload, comparative analysis, nursing, staffing, nurse staffing, registered nurses, nurse assistants, staff allocation, patient capacity

## Abstract

**Background:**

Optimal nurse staffing levels have been shown to impact patients’ prognoses and safety, as well as staff burnout. The predominant method for calculating staffing levels has been patient-to-nurse (P/N) ratios and nursing hours per patient day. However, both methods fall short of addressing the dynamic nature of staffing needs that often fluctuate throughout the day as patients’ clinical status changes and new patients are admitted or discharged from the unit.

**Objective:**

In this evaluation, the Veterans Affairs Palo Alto Health Care System (VAPAHCS) piloted a new dynamic bed count calculation in an effort to target optimal staffing levels every hour to provide greater temporal resolution on nurse staffing levels within the Veterans Health Administration.

**Methods:**

The dynamic bed count uses elements from both the nursing hours per patient day and P/N ratio to calculate current and target staffing levels, every hour, while balancing across nurse types (registered nurses to nurse assistants) to provide improved temporal insight into staff allocation. The dynamic bed count was compared with traditional P/N ratio methods of calculating patient capacity at the VAPAHCS, to assess optimal patient capacity within their acute care ward from January 1, 2023, through May 25, 2023. Descriptive statistics summarized patient capacity variables across the intensive care unit (ICU), medical-surgical ICU, and 3 acute care units. Student *t* tests (2-tailed) were used to analyze differences between patient capacity measures.

**Results:**

Hourly analysis of patient capacity information displayed how the dynamic bed count provided improved temporal resolution on patient capacity. Comparing the dynamic bed count to the P/N ratio, we found the patient capacity, as determined by the P/N ratio, was, on average, higher than that of the dynamic bed count across VAPAHCS acute care units and the medical-surgical ICU (*P*<.001). For example, in acute care unit 3C, the average dynamic bed count was 21.6 (SD 4.2) compared with a P/N ratio of 28.6 (SD 3.2). This suggests that calculating patient capacity using P/N ratios alone could lead to units taking on more patients than what the dynamic bed count suggests the unit can optimally handle.

**Conclusions:**

As a new patient capacity calculation, the dynamic bed count provided additional details and timely information about clinical staffing levels, patient acuity, and patient turnover. Implementing this calculation into the management process has the potential to empower departments to further optimize staffing and patient care.

## Introduction

Nurse staffing levels can impact patients’ prognoses and safety, as well as staff burnout, job satisfaction, workplace injury, and illness [1-4]. A common method for assessing and managing nurse staffing levels is the patient-to-nurse (P/N) ratio, or the total number of patients that are assigned to one nurse during their shift [5-7]. Target ratios are typically based on the type of clinical environment; however, when the unit’s nursing team determines a patient has greater needs, the P/N ratio may be adjusted. The P/N ratio is intended as an efficient tool to assess staffing needs [5], but it primarily focuses on the direct care provided by registered nurses (RNs) and does not inherently account for the full range of nursing and support staff, including licensed practical nurses (LPNs), licensed vocational nurses (LVNs) and nurse assistants (NAs) [8,9].

An alternative to the P/N ratio is the nursing hours per patient day (NHPPD), which account for all nursing types and support staff on the floor, as well as patient complexity, patient turnover, and the presence of higher acuity patients [9-12]. NHPPD provide a comprehensive measure by including the total hours of care provided by each type of nursing staff per patient per day, adjusting for patient acuity, and considering the distribution of nursing care across different shifts and skill levels. Research has shown that using NHPPD significantly decreases mortality and length of stay, and improves patient outcomes [12-14]. While both measures aim to represent the ratio between staffing resources and patient demands, patient needs (and therefore staffing needs) are dynamic throughout the day, and standard intermittent tracking of staffing workloads can lead to inappropriate or incomplete staffing adjustments.

The Veterans Affairs Palo Alto Health Care System (VAPAHCS) is one of the largest medical centers in the Veterans Health Administration (VHA), and operates over 800 patient beds, including 3 acute care units and 6 critical care units [15]. Traditionally, the VAPAHCS used P/N ratios alone to establish patient capacity and staffing levels on the floor in real time for their clinical units. To address the limitations of the P/N ratio and optimize nurse staffing, the VAPAHCS implemented a new staffing solution that integrates the strengths of both the NHPPD and P/N ratio while providing greater temporal resolution, the dynamic bed count. This innovative approach was developed within the Issio Health Care Workforce Optimization Platform [16] through ongoing collaborations with the VAPAHCS charge nurses, nurse managers, and nursing supervisors. The dynamic bed count calculates optimal staffing levels every hour, allowing for more precise and timely adjustments based on real-time patient acuity and turnover.

The need for such a dynamic approach is supported by evidence that traditional staffing metrics often fail to account for the fluctuating nature of patient care demands. For instance, a study found that real-time staffing adjustments based on current patient needs can significantly improve patient outcomes and reduce staff burnout compared with static models [17]. In this pilot assessment, we compare the dynamic bed count to the P/N ratio to describe patient capacity and staffing allocation within the acute ward of the VAPAHCS. The aim of this paper is to evaluate whether the dynamic bed count provides a more accurate and responsive method for determining optimal nurse staffing levels compared with the traditional P/N ratio.

## Methods

### Setting

We analyzed patient capacity data for 3 VAPAHCS acute care units, 1 intensive care unit (ICU), and 1 medical-surgical (Med-Surg) ICU from January 1, 2023, to May 25, 2023. This time frame was selected, as it represents the pilot period for implementing the dynamic bed count solution prior to rolling it out to other VHA hospitals. The selected units were chosen for this pilot due to the fluctuating care requirements for these patients. Being treated for acute conditions (eg, infections, heart conditions, and postoperative care), the care and assistance these patients require vary greatly throughout the day. As such, nursing staff would greatly benefit from a new staffing measurement that accounts for these dynamic and fluctuating patient requirements along with considering various nursing types and specialties. The new measurement was calculated in tandem with legacy methods to ensure that all VA policies and compliance standards were upheld throughout the analysis.

### Patient Capacity Data

Two main data sources were used for this assessment and for the dynamic bed count calculation as follows: (1) the VHA’s national electronic health record database, the Corporate Data Warehouse (CDW [18]) and (2) manually tracked data from nursing staff in real time.

The CDW was queried to provide the dynamic bed count solution information regarding each unit’s patient census. The patient census information is captured through admission and discharge data in the CDW. Specifically, the patient census is a precalculated column in the CDW using admission and discharge information and was collected using SQL queries. The maximum number of beds the unit can support when fully staffed is referred to as the unit’s “authorized capacity” and was also obtained through querying the CDW. The authorized capacity is a relatively stable metric and would only change based on factors that reduce the overall number of physical beds a unit could successfully support such as construction, or long-term staffing constraints. Per the legacy processes, the P/N ratio was established by the units’ nursing supervisors at the beginning of every shift, dictated by California P/N ratio laws [3,19], and was captured by charge nurses entering this information into the Dynamic Bed Count solution. These metrics (patient census, authorized capacity, and P/N ratio) were captured hourly for each unit within the assessment period.

### Dynamic Bed Count

The dynamic bed count calculation within the Issio Health Care Workforce Optimization Platform is designed to represent the number of available staffed beds based on the type of care each nurse is qualified to provide to support their patients. For example, an RN is qualified to perform more tasks when caring for a patient (eg, medication administration, patient triage, and patient education) than an LPN/LVN or NA.

The dynamic bed count uses both target and current staffing levels in its calculation, along with other key staffing variables such as nurse type, shift assignment, patient acuity, and unit regulations. Target staffing levels are the total required minutes of care provided to all patients in the unit within the given hour, considering the average need of a patient as determined by the unit’s NHPPD requirements. NHPPD account for the overall care hours per day that a patient must receive, broken down by shift mix (percentage basis across shifts: night, day, and evening) and skill mix (percentage basis across nursing and support staff skills: RN, LPN/LVN, and NA). Current staffing levels represent the aggregate of the current staff providing direct patient care and their shift assignments. Nursing staff can have their shift assignments (direct or indirect care) dynamically changed throughout the shift, which is entered into the dynamic bed count solution every hour for data accuracy.

The dynamic bed count also considers the average patient’s direct care requirements and allows for granular refinement based on patient acuity adjustments. Patient acuity, as determined by the unit’s charge nurse, uses P/N ratios to adjust care levels based on the required level of care and monitoring. For example, high-acuity patients have a P/N ratio of 1:1, medium-acuity patients have a P/N ratio of 2:1 or 3:1, and low-acuity patients have a P/N ratio of 4:1 or 5:1. Additionally, the calculation includes minutes of care required for admissions and discharges, ensuring that the dynamic bed count reflects the unit’s actual workload every hour. A more detailed description and example of the dynamic bed count calculation is available in Multimedia Appendix 1. The output of the dynamic bed count calculation is displayed on a “Patient Capacity Whiteboard” in Issio’s Command Center or accessible to charge nurses via a web link to inform nursing staff when a unit is under, over, or adequately staffed every hour so they can make the necessary staffing adjustments across adjacent units (Multimedia Appendix 2). By providing a detailed and responsive method for calculating optimal nurse staffing levels, the dynamic bed count enables more accurate and timely adjustments to staff allocation, improving overall patient care and resource management.

### Statistical Analysis

Analyses were performed using Python (version 3.8.5; Python Software Foundation) in a Jupyter Notebook Environment. Descriptive statistics summarized patient capacity variables (patient census, authorized capacity, P/N ratio, and the dynamic bed count). The paired Student *t* test (2-tailed) determined any significant differences between the P/N ratio and dynamic bed count. The unit of analysis for the *t* test was the hourly rates of patient capacity as calculated by both methods. We then plotted the average patient capacity variables for each unit during the assessment period to visually assess differences between the dynamic bed count and the P/N ratio.

To further compare P/N ratios and the dynamic bed count, we analyzed all data points from each unit during the assessment period and calculated the δ between the dynamic bed count and the P/N ratio for each unit. We additionally randomly selected 10 dates and times from the unit with the most variance (ie, SD) in their dynamic bed count calculation hour-by-hour during the assessment period to provide a snapshot of the data points. This was done using Python’s “random” library to generate random numbers corresponding to the indices of dates in our dataset, thereby ensuring unbiased data representation and mitigating any selection bias. This was then plotted to visualize the hourly differences within the selected unit between methods during the assessment period. All graphical representations were accomplished using Python packages such as “matplotlib” for plotting and “pandas” for data manipulation and analysis.

### Ethical Considerations

This quality improvement and assessment project received a Determination of Non-Research from the Stanford Institutional Review Board (IRB; Stanford University, Stanford, California; #73003). The Stanford IRB serves as the affiliated IRB for the VAPAHCS, ensuring ethical oversight and compliance with federal regulations. Informed consent was not required for this project, as it was determined to be a nonresearch quality improvement initiative. All procedures adhered to institutional and federal guidelines to protect participant rights and confidentiality.

## Results

### Patient Capacity Data

**Figure 1.**
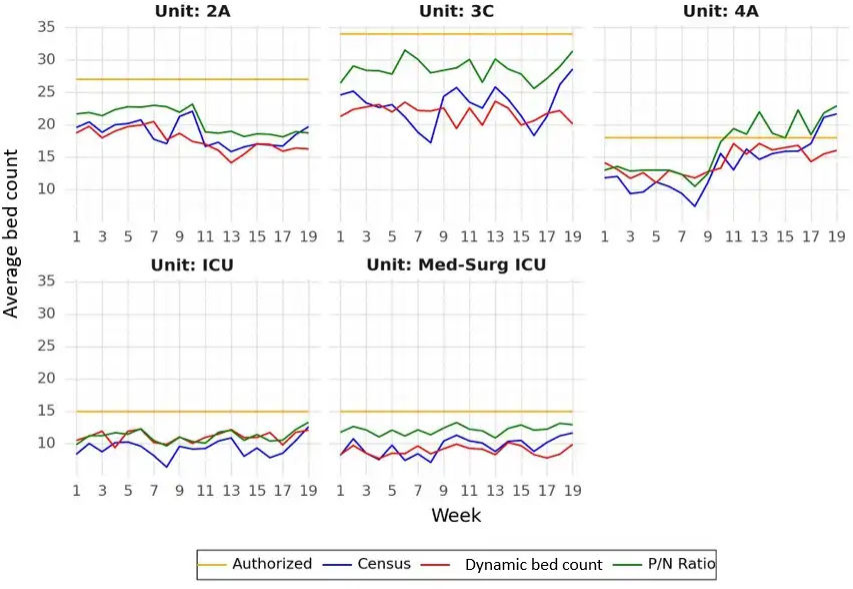
Average patient capacity across Palo Alto Veterans Affairs Health Care System’s acute units, January 1, 2023, through May 25, 2023. In this graph, the yellow lines depict the authorized bed count, which is the maximum number of physical beds a unit could successfully support. The blue lines represent the average patient census for each unit location. The red lines depict the average dynamic bed count calculation for patient capacity while the green lines represent the average patient-to-nurse (P/N) ratio across the assessment period for each acute care unit. Med-Surg ICU: medical-surgical intensive care unit.

Over the assessment period, the number of patients that the different units supported varied (Table 1). For example, the acute care unit 3C had an average of 23.2 (SD 4.5) patients occupying their unit compared with an average of 9.5 (SD 2.0) patients in the ICU. Compared with the P/N ratio, the average dynamic bed count was significantly lower in all acute care unit locations, with the exception of the ICU, (Table 1; *P*<.001). This is further represented in Figure 1, where we can see that the P/N ratio was consistently higher across most units during the assessment period.

**Table 1.** Average patient capacity and occupancy characteristics by Palo Alto Veterans Affairs Health Care System’s acute care units.

Acute care unit locations	Patient census, mean (SD)	Authorized capacity^a^, mean (SD)	Patient capacity calculations		
			Dynamic bed count, mean (SD)	Patient-to-nurse ratio, mean (SD)	*P* value
2A	18.6 (3.8)	27.0 (0.0)	17.5 (2.9)	20.5 (2.1)	<.001
3C	23.2 (4.5)	34.0 (0.0)	21.6 (4.2)	28.6 (3.2)	<.001
4A	14.1 (4.4)	18.0 (0.0)	14.3 (3.5)	16.7 (4.2)	<.001
Intensive care unit	9.5 (2.0)	15.0 (0.0)	11.2 (1.8)	11.2 (1.5)	*.*40
Med-Surg^b^ intensive care unit	9.7 (2.1)	15.0 (0.0)	9.0 (2.1)	12.3 (1.5)	*<.*001
All units	15.0 (6.4)	21.8 (0.0)	14.7 (5.4)	17.8 (6.9)	<.001

a Authorized capacity is the maximum number of beds the unit can support when fully staffed.

b Med-Surg: medical-surgical.

### Dynamic Bed Count

To provide a snapshot of the comparison of P/N ratios and the dynamic bed count, we evaluated the unit with the most variance within the dynamic bed count calculation (displayed in Table 1) across the assessment period. This resulted in a random sample of 10 dates and times within the acute care unit 3C (Table 2). The full dataset is provided in Multimedia Appendix 3. The “Difference” column compares the P/N ratio and the dynamic bed count, where a negative value indicates that the unit should have less patient capacity while a positive value indicates the unit could have taken on additional patients. The amplitude of the value defines how many fewer (negative value) or more (positive value) patients the unit could have handled during any given hour. These data points demonstrate the swing between the quick math “in the moment” patient capacity calculation (P/N ratios) and the standardized, repeatable computation in real time (the dynamic bed count).

**Figure 2.**
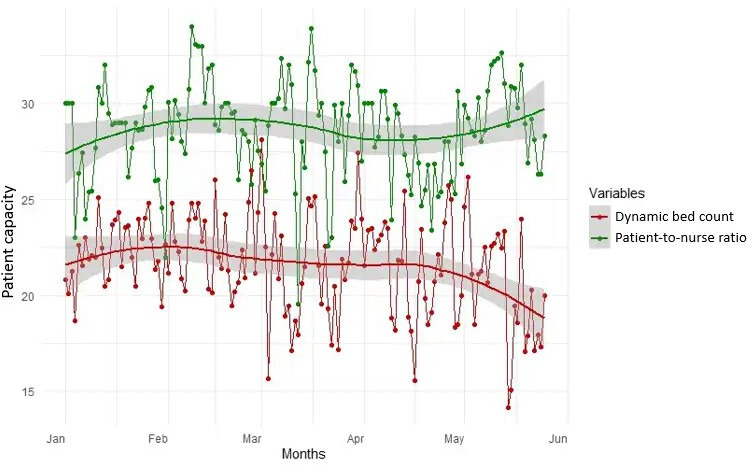
Average patient-to-nurse (P/N) ratio and dynamic bed count calculations across Veterans Affairs Palo Alto Health Care System’s acute unit 3C Location, January 1, 2023, through May 25, 2023. The analysis of patient capacity metrics over the evaluation period revealed an upward trend in the P/N ratio, indicated by the green line, suggesting an increase in the number of patients assigned to each nurse on average. In contrast, the dynamic bed count, shown in red, demonstrates a slight downward trend. Notably, the shaded regions around the trend lines, which represent the SE, suggest greater variability in the dynamic bed count than in the P/N ratio. The divergence in trends between the 2 metrics underscores the complexity of health care resource management and the need for strategies that optimize staffing levels.

Additionally, we graphically represented unit 3C to further display the difference between the P/N ratio and the dynamic bed count calculation throughout the assessment period (Figure 2). In this graph, we can see the difference (δ) between the 2 methods where resource allocation could be improved.

**Table 2.** Random sampling of hours within the acute care unit 3C showing the differences between calculated patient capacity as determined by the patient-to-nurse (P/N) ratio and the dynamic bed count calculation.

Acute care unit location	Date	Time	Patient census, n	Patient capacity calculations		Difference (dynamic bed count vs P/N ratio)
				P/N ratio	Dynamic bed count	
3C	January 1, 2023	7 AM	21	30.0	18.7	−11.3
3C	January 9, 2023	10 PM	29	25.0	20.5	−4.5
3C	February 12, 2023	4 PM	15	30.0	32.2	2.2
3FC	February 19, 2023	6 PM	15	30.0	25.8	−4.2
3C	March 2, 2023	4 PM	29	25.0	25.8	0.8
3C	March 5, 2023	3 PM	22	30.0	26.4	−3.6
3C	April 8, 2023	10 PM	21	25.0	17.6	−7.4
3AC	April 13, 2023	6 AM	20	26.0	29.3	3.3
3C	May 3, 2023	8 AM	26	28.0	17.8	−10.2
3C	May 24, 2023	1 PM	24	29.0	20.3	−8.7

## Discussion

In efforts to improve nurse scheduling and optimize workload across acute care and critical care units of the VAPAHCS, we implemented the dynamic bed count to calculate the optimal workload of each unit every hour. The main findings of this assessment revealed that the dynamic bed count can provide a more accurate and responsive method for determining optimal nurse staffing levels compared with the traditional P/N ratios. Through comparison of the P/N ratio and the dynamic bed count, we found that the P/N ratios implemented by the nursing staff, with the exception of the ICU, resulted in significantly higher calculated patient capacity levels on average than what the unit can adequately accommodate. Inadequate staffing levels could result in an increased risk of burnout and workplace injury among our nursing staff and have been shown to worsen patient outcomes [7,20,21]. Furthermore, when looking at VAPAHCS units hour-by-hour, we found times when the units could support a higher patient capacity than what was calculated by the nursing staff. By accounting for dynamic changes within units with the dynamic bed count, nursing supervisors can allocate staff appropriately across units without necessarily having to hire additional staff.

As seen in previous research, the P/N ratio falls short of fully grasping a true picture of optimal workload. In one study, researchers found that the P/N ratio cannot properly capture admissions and discharges of patients, or where nurses work as a team [22]. For example, the P/N ratio implies that each nurse has responsibility for a set group of patients. However, that is often not the case. A nurse’s patient load changes as patients are admitted and discharged during a shift. For example, this can result in a nurse starting their shift with 5 patients who are eventually replaced by 5 other patients later in the shift as they are admitted/discharged. Additionally, although P/N ratios can be adjusted [17], they inherently only account for RN staffing types and no other support staff on the floor. This can be an important aspect of the team dynamic that is often present in health care units, such as those seen in the VAPAHCS acute care ward.

As an alternative to P/N ratios, the NHPPD have been endorsed by the National Quality Forum to measure appropriate nurse staffing levels [11] and are known to reliably increase the quality of care for patients [10-12]. The NHPPD staffing measure classifies units into 1 of 7 categories as determined by patient complexity, intervention levels, presence of high-dependency beds, and patient turnover [10-12]. It has been implemented in the long-term care clinics within VHA, where researchers found that higher NHPPD levels were inversely associated with falls resulting in major injury [21]. While the NHPPD can be seen as a marked improvement to the P/N ratio, it is important to acknowledge that both methods are based on similar input variables, and both serve to represent the ratio between staffing resources and patient demands. However, the key distinction lies in the level of aggregation and the time frame over which these metrics are applied. P/N ratios provide a snapshot based on the number of patients per nurse, which can be adjusted as needed but typically lacks granularity in real-time adjustments [17]. NHPPD, on the other hand, offer a comprehensive approach by accounting for the total hours of care from all types of nursing staff per patient per day, incorporating patient acuity and turnover [12]. Despite these strengths, NHPPD may fall short of addressing the dynamic nature of staffing needs that can fluctuate throughout the day.

The dynamic bed count addresses these limitations by providing hourly updates on staffing levels and patient acuity, allowing for more precise and timely adjustments. This method separates the assessment of demand (patients, admissions, discharges, and patient acuity) and supply (nurses, skill, and shift assignment), identifying opportunities to move staff or manage patient flow more effectively. Given the capacity use of the units, such as the range of around 19‐28 patients in unit 3C, the potential for optimizing staffing and improving patient outcomes is substantial. By integrating elements from both the P/N ratios and NHPPD, the dynamic bed count offers a balanced and dynamic solution that reflects real-time conditions within the units, thereby enhancing the overall efficiency and effectiveness of nurse staffing.

Tools such as the dynamic bed count solution can be powerful in helping charge nurses better understand the rationale behind the load balancing of staff. By including staffing types and patient acuity, the dynamic bed count can help charge nurses determine the most effective combination of staff to deliver high-quality and cost-effective patient care. This can be especially important in the face of rising demand for health services and shortages of nurses and other health care workers both within the VHA and other US health care sectors [22-24]. Additionally, the diversity of staffing models in our health care system is essential to determine which staff members should be included in the staffing calculations to reflect personnel who deliver direct care relevant to patient outcomes [22].

Implementing the dynamic bed count solution does present certain challenges and limitations. One potential concern is the additional workload on nursing staff to maintain accurate data entry. However, in this pilot implementation, charge nurses only entered data into the dynamic bed count solution at the start of every shift, when their staff was changed from direct to indirect (or vice versa) shift assignments, and when there were changes in patient acuity, which aligns with their existing workflow and did not add extra burden to the nursing staff. The dynamic bed count solution then uses this information to display and communicate optimal patient capacity and suggestions for resource allocation in real time, effectively lessening the burden on charge nurses by providing them with actional insights and reducing the need for manual calculations and adjustments.

Another challenge is the potential cultural adjustment required for staff to embrace new technology and processes. Although all units are part of the same health care system, reallocating personnel from a well-staffed ward to an understaffed ward can be undesirable to the unit losing personnel, and can be a stressful experience for the transferred nurse due to unfamiliarity with the adjacent unit [25]. This challenge can be mitigated through cross-training, or implementing a “buddy system” across units, which in turn can increase job satisfaction [25]. Increasing “float pools” is another strategy to mitigate this issue and has been shown to significantly reduce turnover and overall staffing costs [26,27].

The dynamic bed count’s strength lies in its development, which stemmed from continuous collaborations with VAPAHCS nursing leadership. These collaborations ensured a comprehensive understanding of crucial data points for accurate capacity assessment and validation of the calculation’s precision. Each variable can change at a moment’s notice and can have a major impact on a unit’s capacity. This information, as soon as it is changed, must be presented to “need to know” parties, like nursing supervisors and patient flow coordinators, so quick and accurate decisions can be made about floating staff to areas where they are needed. With the overall complexity differences between P/N ratios and dynamic bed count, it is easy to understand why P/N ratios take place on the floor “in the moment,” but with technology that can account for the additional complexities easily, accurately, and quickly, we can see improved accuracy in the staffing decision-making processes.

This assessment has some limitations. First, these findings are only relevant for the VAPAHCS acute care ward during the assessment period. Our findings could vary for other time frames and in other units. Future assessments will be needed to test the implementation of the dynamic bed count on any improved patient outcomes.

In conclusion, we believe that a new calculation such as the dynamic bed count, as presented here, could be a marked improvement from the P/N ratio for the VAPAHCS acute care ward. Implementing this calculation into a web-based report that supervisors could use to allocate nursing staff could significantly improve the workflow of our health care system.

## Supplementary material

10.2196/59619 Multimedia Appendix 1 Description of the dynamic bed count calculation.

10.2196/59619 Multimedia Appendix 2 Depiction of “Patient Capacity Whiteboard.”

10.2196/59619 Multimedia Appendix 3 Sampling of hours within the acute care unit 3C displaying the differences between calculated patient capacity as determined by the patient-to-nurse ratio and the dynamic bed count calculation.

## Abbreviations

CDW: Corporate Data Warehouse
ICU: intensive care unit
IRB: Institutional Review Board
LPN: licensed practical nurse
LVN: licensed vocational nurse
Med-Surg: medical-surgical
NA: nurse assistant
NHPPD: nursing hours per patient day
P/N: patient-to-nurse
RN: registered nurse
VAPAHCS: Veterans Affairs Palo Alto Health Care System
VHA: Veterans Health Administration
