# Supplementary material for: Calculating Optimal Patient to Nursing Capacity: Comparative Analysis of Traditional and New Methods
Source: JMIR Nurs. 2024 Nov 22;7:e59619. doi: 10.2196/59619 (PMC11612603; doi:10.2196/59619)
Supplement: Multimedia Appendix 1 [file nursing-v7-e59619-s001.docx]

**Appendix 1**: Description of the Dynamic Bed Count Calculation

The Dynamic Bed Count calculation is designed to represent the number of available staffed beds in relation to specific types of nursing qualifications needed to support the patients.

**Target Staffing Levels**

Target staffing levels, also known as the workload of the unit, refer to the total required minutes of care provided to all patients in the unit within a given hour. These levels consider the average need of each patient, as determined by that unit’s calculated Nursing Hours Per Patient Day (NHPPD) requirements. NHPPD requirements are determined daily using staffing policies and guidelines set by VAPAHCS Nursing Leadership, which takes into account historical data, benchmarks, and each unit’s unique business plan, but are not used directly by VAPAHCS nursing staff on the floor. NHPPD accounts for the overall care hours per day that a patient must receive, broken down by shift mix (percentage basis across shifts: night, day, and evening) and skill mix (percentage basis across nursing and support staff skills: RN, LPN/LVN, NA). The NHPPD breakdown determines how many hours of patient care each skill type will need to provide per shift to adequately care for the unit’s patients. Further dividing by the number of hours within each shift gives us the total care requirements in minutes, per hour, per skill.

**Dynamic Bed Count and Patient Acuity**

The Dynamic Bed Count considers the average patient’s direct care requirements and allows granular refinement based on patient acuity adjustments. Patient acuity, determined by the unit’s charge nurse and manually entered into the Dynamic Bed Count solution, uses Patient-to-Nurse (P/N) ratios as the to adjust care levels based on the required level of care and monitoring. For example,

- High acuity: P/N ratio of 1:1
- Medium acuity: P/N ratio of 2:1 or 3:1
- Low acuity: P/N ratio of 4:1 or 5:1

The patient acuity can also be assigned to a particular skill type, increasing the minutes of care provided to that patient by different nursing staff types when necessary. Additionally, if there were any admissions or discharges, the minutes of care required for each admission and discharge would also be added to the total required time of patient care the unit needs to provide.

While the hourly care requirements are initially calculated based on dividing the shift requirement by the hours per shift, there can be significant variation within a shift. Factors such as patient acuity changes, new admissions, discharges, and specific patient needs can all impact the actual care required at different times during a shift. For instance, a high-acuity patient may require more intensive care at certain times, and the arrival of new patients or the discharge of existing ones can also create peaks in care demands. Therefore, the Dynamic Bed Count allows for these adjustments to be made throughout the shift, providing a more accurate and responsive staffing measure.

**Current Staffing Levels**

Current staffing levels, also known as the available output of the unit, are calculated as the aggregate of the current staff providing direct patient care. Nursing staff can have their shift assignment (direct or indirect care) changed dynamically throughout the shift, which was entered every hour to increase accuracy. Subtracting the current staffing levels output from the target staffing levels workload and dividing by the average patient care requirements gives us the additional capacity that the unit can handle, known as the Dynamic Bed Count. The highest tiered skills (RNs) are the limiting capacity within units. If there is no additional capacity for the highest tiered skill within the unit, then the unit is limited to that skill’s patient capacity.

**Example Calculation**

Here is an example of calculating the Dynamic Bed Count for RNs within the day shift of a given unit. First, we would calculate the average direct care (in hours) required from RNs per patient during the day shift in a unit with an NHPPD set at 10, shift mix of 50%-day shift, 30%-evening shift, 20%-night shift, and a skill mix of 75%-RNs and 25%-NAs:

*10 (NHPPD) X 50% (shift mix for day shift) X 75% (skill mix for RNs) = 3.75 (hours of RN care per patient).*

This is then divided by the number of hours per shift (8 hours) to obtain the base number of minutes of care needed per patient per hour:

*3.75 (hours of RN care per patient) / 8 (hours in shift) = 0.47* (*hours of required RN care per patient per hour, or 28.125 minutes).*

If we have a patient with high acuity (i.e., a 1:1 P/N ratio), the level of care for that patient to 60 minutes per hour. If the remaining 9 other patients in this unit have no additional care needs, no extra time is added. Additional time (in minutes) for any admissions and/or discharges within the hour is also included. Therefore, the target staffing levels would be calculated as:

*9 (low acuity patients) X 28.125 (base number of minutes of care needed) + 1 (high acuity patients) X 60 (minutes of care required for high acuity patient) + 0 (number of admissions) X 40 (minutes needed for admission) + 0 (number of discharges ) X 20 (minutes needed for discharges) = 313.125 minutes*.

It is important to note that the number of minutes needed for admissions or discharges can vary depending on the type of unit, the unit’s admission and discharge process, and the level of patient care needed.

For current staffing levels, if we have 7 RNs on the unit providing direct patient care, we multiply the number of RNs by 60 minutes to get the total number of minutes of available care per hour:

*7 (number of RNs) X 60 minutes = 420 total minutes of available care*.

Next, subtracting the target staffing levels from the current staffing levels that we calculated above, and dividing that by the average base number of minutes of care needed per patient per hour gives us the additional capacity the unit can handle, or the Dynamic Bed Count:

*420 (current staffing levels) – 313.125 (target staffing levels) / 28.125 (average base* number of minutes of care needed per patient per hour*) = 3.8 patients that RNs can additionally adequately handle.*

For any support staff on the floor (NAs or LVP/LPVs), the calculation would be adjusted for their skill mix percentage (i.e., 25%) and patient care requirements.
