# Supplementary material for: Calculating Optimal Patient to Nursing Capacity: Comparative Analysis of Traditional and New Methods
Source: JMIR Nurs. 2024 Nov 22;7:e59619. doi: 10.2196/59619 (PMC11612603; doi:10.2196/59619)
Supplement: Multimedia Appendix 2 [file nursing-v7-e59619-s002.docx]

**Supplement 2:** Depiction of ‘Patient Capacity Whiteboard’


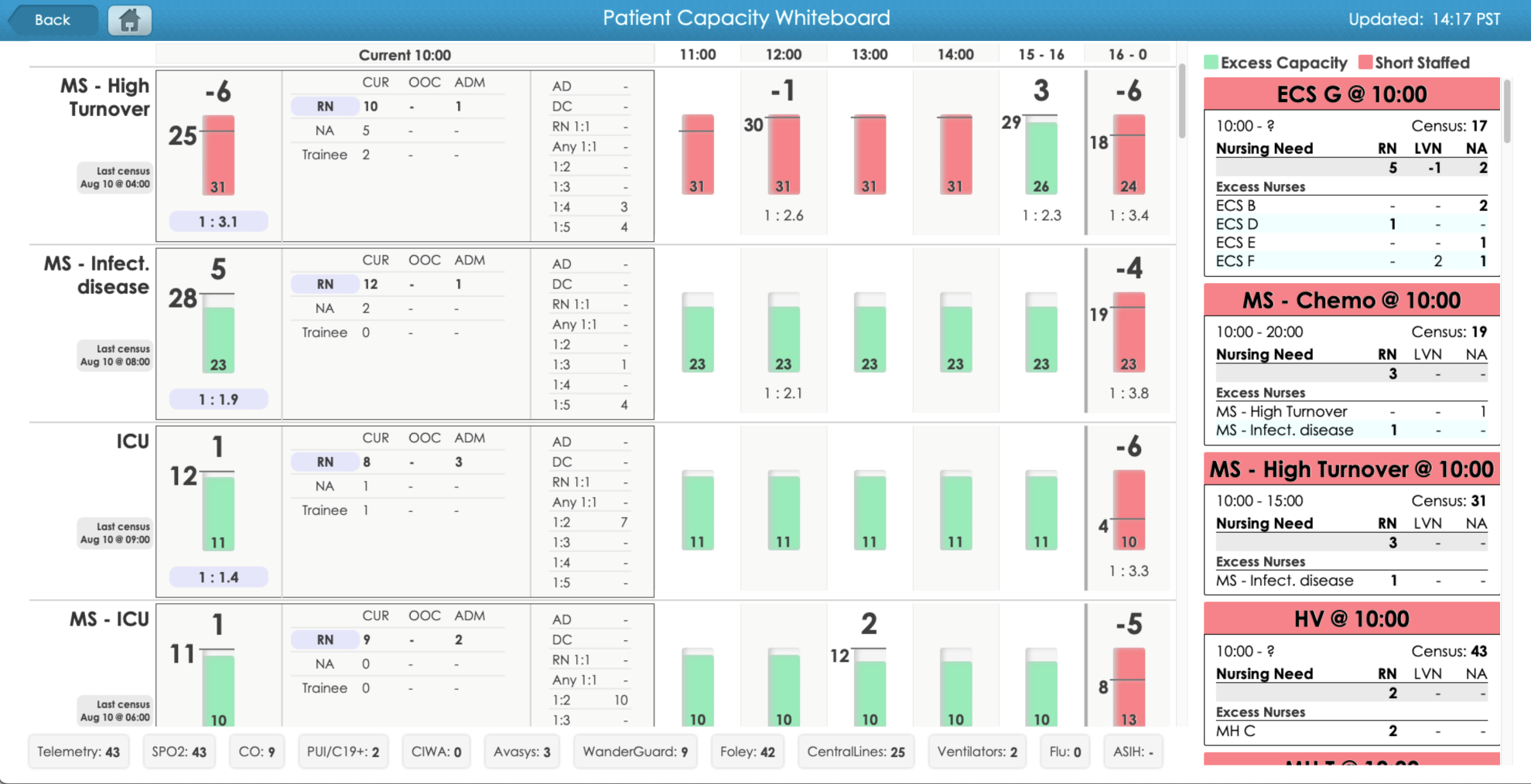


The Patient Capacity Whiteboard is available through Issio’s Command Center^TM^ and is also available to each charge nurse in their respective wards via a weblink to inform nursing staff when a unit was under, over, or adequately staffed per hour so they can make the necessary staffing adjustments across adjacent units.
