# Supplementary material for: Calculating Optimal Patient to Nursing Capacity: Comparative Analysis of Traditional and New Methods
Source: JMIR Nurs. 2024 Nov 22;7:e59619. doi: 10.2196/59619 (PMC11612603; doi:10.2196/59619)
Supplement: Multimedia Appendix 3 [file nursing-v7-e59619-s003.pdf]

Multimedia Appendix 3. Differences between calculated patient capacity as determined by the Patient-to-Nurse ratio and the Dynamic Bed Count calculation.

Acute Care Unit Location	DateTime	Patient Census	P/N Ratio	Dynamic Bed Count	Difference Dynamic Bed Count vs. P/N Ratio
2A	1/1/2023 0:00	18	22.0	11.5	-10.5
2A	1/1/2023 1:00	18	22.0	11.5	-10.5
2A	1/1/2023 2:00	18	22.0	11.5	-10.5
2A	1/1/2023 3:00	18	22.0	11.5	-10.5
2A	1/1/2023 4:00	18	22.0	11.5	-10.5
2A	1/1/2023 5:00	18	22.0	11.5	-10.5
2A	1/1/2023 6:00	18	22.0	11.5	-10.5
2A	1/1/2023 7:00	18	22.0	11.5	-10.5
2A	1/1/2023 8:00	18	20.0	13.1	-6.9
2A	1/1/2023 9:00	18	20.0	13.1	-6.9
2A	1/1/2023 10:00	18	20.0	13.1	-6.9
2A	1/1/2023 11:00	18	20.0	13.1	-6.9
2A	1/1/2023 12:00	18	20.0	13.1	-6.9
2A	1/1/2023 13:00	18	20.0	13.1	-6.9
2A	1/1/2023 14:00	18	20.0	13.1	-6.9
2A	1/1/2023 15:00	18	20.0	13.1	-6.9
2A	1/1/2023 16:00	18	20.0	13.9	-6.1
2A	1/1/2023 17:00	18	20.0	13.9	-6.1
2A	1/1/2023 18:00	18	20.0	13.9	-6.1
2A	1/1/2023 19:00	18	20.0	13.9	-6.1
2A	1/1/2023 20:00	19	22.0	19.5	-2.5
2A	1/1/2023 21:00	19	22.0	19.5	-2.5
2A	1/1/2023 22:00	19	22.0	19.5	-2.5
2A	1/1/2023 23:00	19	22.0	19.5	-2.5
2A	1/2/2023 0:00	19	22.0	20.1	-1.9
2A	1/2/2023 1:00	19	22.0	20.1	-1.9
2A	1/2/2023 2:00	19	22.0	20.1	-1.9
2A	1/2/2023 3:00	19	22.0	20.1	-1.9
2A	1/2/2023 4:00	19	22.0	20.1	-1.9
2A	1/2/2023 5:00	19	22.0	20.1	-1.9
2A	1/2/2023 6:00	19	22.0	20.1	-1.9
2A	1/2/2023 7:00	19	22.0	20.1	-1.9
2A	1/2/2023 8:00	19	22.0	13.1	-8.9
2A	1/2/2023 9:00	19	22.0	13.1	-8.9
2A	1/2/2023 10:00	19	22.0	13.1	-8.9
2A	1/2/2023 11:00	19	22.0	13.1	-8.9
2A	1/2/2023 12:00	19	22.0	13.1	-8.9
2A	1/2/2023 13:00	19	22.0	13.1	-8.9
2A	1/2/2023 14:00	19	22.0	13.1	-8.9
2A	1/2/2023 15:00	19	22.0	13.1	-8.9

2A	1/2/2023 16:00	19	22.0	16.7	-5.3
2A	1/2/2023 17:00	19	22.0	16.7	-5.3
2A	1/2/2023 18:00	19	22.0	16.7	-5.3
2A	1/2/2023 19:00	19	22.0	16.7	-5.3
2A	1/2/2023 20:00	19	22.0	15.6	-6.4
2A	1/2/2023 21:00	15	22.0	17.8	-4.2
2A	1/2/2023 22:00	15	22.0	17.8	-4.2
2A	1/2/2023 23:00	15	22.0	17.8	-4.2
2A	1/3/2023 0:00	15	22.0	17.2	-4.8
2A	1/3/2023 1:00	17	22.0	17.2	-4.8
2A	1/3/2023 2:00	17	22.0	17.2	-4.8
2A	1/3/2023 3:00	17	22.0	17.2	-4.8
2A	1/3/2023 4:00	18	22.0	17.2	-4.8
2A	1/3/2023 5:00	18	22.0	17.2	-4.8
2A	1/3/2023 6:00	18	22.0	17.2	-4.8
2A	1/3/2023 7:00	18	22.0	17.2	-4.8
2A	1/3/2023 8:00	18	22.0	18.4	-3.6
2A	1/3/2023 9:00	18	22.0	18.4	-3.6
2A	1/3/2023 10:00	18	22.0	18.4	-3.6
2A	1/3/2023 11:00	18	22.0	18.4	-3.6
2A	1/3/2023 12:00	18	22.0	21.0	-1.0
2A	1/3/2023 13:00	18	22.0	21.0	-1.0
2A	1/3/2023 14:00	18	22.0	21.0	-1.0
2A	1/3/2023 15:00	18	22.0	21.0	-1.0
2A	1/3/2023 16:00	17	22.0	22.3	0.3
2A	1/3/2023 17:00	17	22.0	22.3	0.3
2A	1/3/2023 18:00	17	22.0	22.3	0.3
2A	1/3/2023 19:00	17	22.0	22.3	0.3
2A	1/3/2023 20:00	20	20.0	16.7	-3.3
2A	1/3/2023 21:00	20	20.0	16.7	-3.3
2A	1/3/2023 22:00	20	20.0	18.1	-1.9
2A	1/3/2023 23:00	20	20.0	19.5	-0.5
2A	1/4/2023 0:00	21	22.0	20.1	-1.9
2A	1/4/2023 1:00	21	22.0	20.1	-1.9
2A	1/4/2023 2:00	21	22.0	20.1	-1.9
2A	1/4/2023 3:00	21	22.0	20.1	-1.9
2A	1/4/2023 4:00	21	22.0	20.1	-1.9
2A	1/4/2023 5:00	21	22.0	20.1	-1.9
2A	1/4/2023 6:00	21	22.0	20.1	-1.9
2A	1/4/2023 7:00	21	22.0	20.1	-1.9
2A	1/4/2023 8:00	21	22.0	18.4	-3.6
2A	1/4/2023 9:00	21	22.0	18.4	-3.6
2A	1/4/2023 10:00	17	20.0	16.8	-3.2
2A	1/4/2023 11:00	17	20.0	18.4	-1.6
2A	1/4/2023 12:00	17	20.0	19.4	-0.6
2A	1/4/2023 13:00	17	20.0	17.8	-2.2
2A	1/4/2023 14:00	17	20.0	21.0	1.0

2A	1/4/2023 15:00	17	20.0	21.0	1.0
2A	1/4/2023 16:00	17	20.0	16.7	-3.3
2A	1/4/2023 17:00	17	20.0	16.7	-3.3
2A	1/4/2023 18:00	17	20.0	16.7	-3.3
2A	1/4/2023 19:00	17	20.0	16.7	-3.3
2A	1/4/2023 20:00	17	20.0	19.5	-0.5
2A	1/4/2023 21:00	17	20.0	19.5	-0.5
2A	1/4/2023 22:00	17	20.0	19.5	-0.5
2A	1/4/2023 23:00	17	20.0	19.5	-0.5
2A	1/5/2023 0:00	17	22.0	17.2	-4.8
2A	1/5/2023 1:00	17	22.0	17.2	-4.8
2A	1/5/2023 2:00	17	22.0	17.2	-4.8
2A	1/5/2023 3:00	17	22.0	17.2	-4.8
2A	1/5/2023 4:00	17	22.0	17.2	-4.8
2A	1/5/2023 5:00	20	22.0	17.2	-4.8
2A	1/5/2023 6:00	20	22.0	17.2	-4.8
2A	1/5/2023 7:00	20	22.0	17.2	-4.8
2A	1/5/2023 8:00	20	21.0	18.4	-2.6
2A	1/5/2023 9:00	20	21.0	18.4	-2.6
2A	1/5/2023 10:00	20	21.0	18.4	-2.6
2A	1/5/2023 11:00	20	21.0	18.4	-2.6
2A	1/5/2023 12:00	18	22.0	18.4	-3.6
2A	1/5/2023 13:00	18	22.0	15.2	-6.8
2A	1/5/2023 14:00	18	22.0	15.2	-6.8
2A	1/5/2023 15:00	18	22.0	18.4	-3.6
2A	1/5/2023 16:00	18	22.0	19.5	-2.5
2A	1/5/2023 17:00	18	22.0	19.5	-2.5
2A	1/5/2023 18:00	18	22.0	19.5	-2.5
2A	1/5/2023 19:00	18	22.0	19.5	-2.5
2A	1/5/2023 20:00	18	22.0	19.5	-2.5
2A	1/5/2023 21:00	19	20.0	19.5	-0.5
2A	1/5/2023 22:00	21	22.0	19.5	-2.5
2A	1/5/2023 23:00	22	22.0	16.7	-5.3
2A	1/6/2023 0:00	22	22.0	14.4	-7.7
2A	1/6/2023 1:00	22	22.0	14.4	-7.7
2A	1/6/2023 2:00	22	22.0	14.4	-7.7
2A	1/6/2023 3:00	22	22.0	14.4	-7.7
2A	1/6/2023 4:00	22	22.0	14.4	-7.7
2A	1/6/2023 5:00	22	22.0	14.4	-7.7
2A	1/6/2023 6:00	22	22.0	14.4	-7.7
2A	1/6/2023 7:00	22	22.0	14.4	-7.7
2A	1/6/2023 8:00	22	22.0	18.4	-3.6
2A	1/6/2023 9:00	22	22.0	18.4	-3.6
2A	1/6/2023 10:00	21	22.0	16.8	-5.2
2A	1/6/2023 11:00	21	22.0	18.4	-3.6
2A	1/6/2023 12:00	21	22.0	18.4	-3.6
2A	1/6/2023 13:00	21	22.0	18.4	-3.6

2A	1/6/2023 14:00	21	22.0	18.4	-3.6
2A	1/6/2023 15:00	21	22.0	18.4	-3.6
2A	1/6/2023 16:00	21	22.0	16.7	-5.3
2A	1/6/2023 17:00	21	22.0	16.7	-5.3
2A	1/6/2023 18:00	21	22.0	16.7	-5.3
2A	1/6/2023 19:00	21	22.0	16.7	-5.3
2A	1/6/2023 20:00	21	22.0	22.3	0.3
2A	1/6/2023 21:00	21	22.0	22.3	0.3
2A	1/6/2023 22:00	21	22.0	22.3	0.3
2A	1/6/2023 23:00	21	22.0	22.3	0.3
2A	1/7/2023 0:00	21	22.0	23.0	1.0
2A	1/7/2023 1:00	21	22.0	23.0	1.0
2A	1/7/2023 2:00	21	22.0	23.0	1.0
2A	1/7/2023 3:00	21	22.0	23.0	1.0
2A	1/7/2023 4:00	21	22.0	23.0	1.0
2A	1/7/2023 5:00	21	22.0	23.0	1.0
2A	1/7/2023 6:00	21	22.0	23.0	1.0
2A	1/7/2023 7:00	21	22.0	23.0	1.0
2A	1/7/2023 8:00	21	22.0	21.0	-1.0
2A	1/7/2023 9:00	21	22.0	21.0	-1.0
2A	1/7/2023 10:00	21	22.0	21.0	-1.0
2A	1/7/2023 11:00	21	22.0	21.0	-1.0
2A	1/7/2023 12:00	21	22.0	21.0	-1.0
2A	1/7/2023 13:00	21	22.0	21.0	-1.0
2A	1/7/2023 14:00	21	22.0	21.0	-1.0
2A	1/7/2023 15:00	21	22.0	21.0	-1.0
2A	1/7/2023 16:00	21	22.0	19.5	-2.5
2A	1/7/2023 17:00	21	22.0	19.5	-2.5
2A	1/7/2023 18:00	21	22.0	19.5	-2.5
2A	1/7/2023 19:00	21	22.0	19.5	-2.5
2A	1/7/2023 20:00	21	22.0	22.3	0.3
2A	1/7/2023 21:00	21	22.0	22.3	0.3
2A	1/7/2023 22:00	21	22.0	19.5	-2.5
2A	1/7/2023 23:00	21	22.0	19.5	-2.5
2A	1/8/2023 0:00	21	22.0	16.1	-5.9
2A	1/8/2023 1:00	21	22.0	16.1	-5.9
2A	1/8/2023 2:00	21	22.0	16.1	-5.9
2A	1/8/2023 3:00	21	22.0	16.1	-5.9
2A	1/8/2023 4:00	21	22.0	16.1	-5.9
2A	1/8/2023 5:00	21	22.0	16.1	-5.9
2A	1/8/2023 6:00	21	22.0	16.1	-5.9
2A	1/8/2023 7:00	21	22.0	16.1	-5.9
2A	1/8/2023 8:00	21	22.0	21.0	-1.0
2A	1/8/2023 9:00	21	22.0	21.0	-1.0
2A	1/8/2023 10:00	21	22.0	21.0	-1.0
2A	1/8/2023 11:00	21	22.0	21.0	-1.0
2A	1/8/2023 12:00	21	22.0	23.6	1.6

2A	1/8/2023 13:00	21	22.0	23.6	1.6
2A	1/8/2023 14:00	21	22.0	23.6	1.6
2A	1/8/2023 15:00	21	22.0	23.6	1.6
2A	1/8/2023 16:00	21	22.0	22.3	0.3
2A	1/8/2023 17:00	21	22.0	22.3	0.3
2A	1/8/2023 18:00	21	22.0	22.3	0.3
2A	1/8/2023 19:00	20	20.0	22.3	2.3
2A	1/8/2023 20:00	20	20.0	20.0	0.0
2A	1/8/2023 21:00	20	20.0	20.0	0.0
2A	1/8/2023 22:00	20	20.0	20.0	0.0
2A	1/8/2023 23:00	20	20.0	20.0	0.0
2A	1/9/2023 0:00	20	20.0	23.0	3.0
2A	1/9/2023 1:00	20	20.0	23.0	3.0
2A	1/9/2023 2:00	20	20.0	23.0	3.0
2A	1/9/2023 3:00	20	20.0	23.0	3.0
2A	1/9/2023 4:00	20	20.0	23.0	3.0
2A	1/9/2023 5:00	20	20.0	23.0	3.0
2A	1/9/2023 6:00	20	20.0	23.0	3.0
2A	1/9/2023 7:00	20	20.0	23.0	3.0
2A	1/9/2023 8:00	20	20.0	23.6	3.6
2A	1/9/2023 9:00	22	22.0	23.6	1.6
2A	1/9/2023 10:00	22	22.0	23.6	1.6
2A	1/9/2023 11:00	22	22.0	23.6	1.6
2A	1/9/2023 12:00	22	22.0	23.6	1.6
2A	1/9/2023 13:00	22	22.0	23.6	1.6
2A	1/9/2023 14:00	22	22.0	23.6	1.6
2A	1/9/2023 15:00	22	22.0	23.6	1.6
2A	1/9/2023 16:00	22	22.0	19.5	-2.5
2A	1/9/2023 17:00	22	22.0	19.5	-2.5
2A	1/9/2023 18:00	22	22.0	19.5	-2.5
2A	1/9/2023 19:00	22	22.0	19.5	-2.5
2A	1/9/2023 20:00	22	22.0	22.3	0.3
2A	1/9/2023 21:00	22	22.0	22.3	0.3
2A	1/9/2023 22:00	22	22.0	22.3	0.3
2A	1/9/2023 23:00	22	22.0	20.9	-1.1
2A	1/10/2023 0:00	22	22.0	20.1	-1.9
2A	1/10/2023 1:00	22	22.0	20.1	-1.9
2A	1/10/2023 2:00	22	22.0	17.2	-4.8
2A	1/10/2023 3:00	22	22.0	17.2	-4.8
2A	1/10/2023 4:00	22	22.0	17.2	-4.8
2A	1/10/2023 5:00	22	22.0	17.2	-4.8
2A	1/10/2023 6:00	22	22.0	17.2	-4.8
2A	1/10/2023 7:00	22	22.0	17.2	-4.8
2A	1/10/2023 8:00	22	22.0	18.4	-3.6
2A	1/10/2023 9:00	22	22.0	18.4	-3.6
2A	1/10/2023 10:00	22	22.0	18.4	-3.6
2A	1/10/2023 11:00	22	22.0	18.4	-3.6

2A	1/10/2023 12:00	22	22.0	18.4	-3.6
2A	1/10/2023 13:00	22	22.0	18.4	-3.6
2A	1/10/2023 14:00	22	22.0	18.4	-3.6
2A	1/10/2023 15:00	22	22.0	18.4	-3.6
2A	1/10/2023 16:00	22	22.0	16.7	-5.3
2A	1/10/2023 17:00	22	22.0	16.7	-5.3
2A	1/10/2023 18:00	22	22.0	16.7	-5.3
2A	1/10/2023 19:00	22	22.0	16.7	-5.3
2A	1/10/2023 20:00	22	22.0	19.5	-2.5
2A	1/10/2023 21:00	22	22.0	19.5	-2.5
2A	1/10/2023 22:00	22	22.0	19.5	-2.5
2A	1/10/2023 23:00	22	22.0	19.5	-2.5
2A	1/11/2023 0:00	22	22.0	17.2	-4.8
2A	1/11/2023 1:00	22	22.0	17.2	-4.8
2A	1/11/2023 2:00	22	22.0	17.2	-4.8
2A	1/11/2023 3:00	22	22.0	17.2	-4.8
2A	1/11/2023 4:00	22	22.0	17.2	-4.8
2A	1/11/2023 5:00	22	22.0	17.2	-4.8
2A	1/11/2023 6:00	22	22.0	17.2	-4.8
2A	1/11/2023 7:00	22	22.0	17.2	-4.8
2A	1/11/2023 8:00	22	22.0	18.4	-3.6
2A	1/11/2023 9:00	22	22.0	18.4	-3.6
2A	1/11/2023 10:00	22	22.0	18.4	-3.6
2A	1/11/2023 11:00	22	22.0	18.4	-3.6
2A	1/11/2023 12:00	22	22.0	23.6	1.6
2A	1/11/2023 13:00	22	22.0	23.6	1.6
2A	1/11/2023 14:00	22	22.0	23.6	1.6
2A	1/11/2023 15:00	22	22.0	23.6	1.6
2A	1/11/2023 16:00	22	22.0	22.3	0.3
2A	1/11/2023 17:00	22	22.0	22.3	0.3
2A	1/11/2023 18:00	22	22.0	22.3	0.3
2A	1/11/2023 19:00	22	22.0	22.3	0.3
2A	1/11/2023 20:00	22	22.0	22.3	0.3
2A	1/11/2023 21:00	22	22.0	22.3	0.3
2A	1/11/2023 22:00	22	22.0	22.3	0.3
2A	1/11/2023 23:00	22	22.0	22.3	0.3
2A	1/12/2023 0:00	22	22.0	20.1	-1.9
2A	1/12/2023 1:00	22	22.0	20.1	-1.9
2A	1/12/2023 2:00	22	22.0	20.1	-1.9
2A	1/12/2023 3:00	22	22.0	20.1	-1.9
2A	1/12/2023 4:00	22	22.0	20.1	-1.9
2A	1/12/2023 5:00	22	22.0	20.1	-1.9
2A	1/12/2023 6:00	22	22.0	20.1	-1.9
2A	1/12/2023 7:00	22	22.0	20.1	-1.9
2A	1/12/2023 8:00	22	22.0	18.4	-3.6
2A	1/12/2023 9:00	22	22.0	18.4	-3.6
2A	1/12/2023 10:00	22	22.0	18.4	-3.6

2A	1/12/2023 11:00	22	22.0	18.4	-3.6
2A	1/12/2023 12:00	22	22.0	21.0	-1.0
2A	1/12/2023 13:00	22	22.0	21.0	-1.0
2A	1/12/2023 14:00	22	22.0	21.0	-1.0
2A	1/12/2023 15:00	22	22.0	21.0	-1.0
2A	1/12/2023 16:00	22	22.0	19.5	-2.5
2A	1/12/2023 17:00	22	22.0	19.5	-2.5
2A	1/12/2023 18:00	22	22.0	19.5	-2.5
2A	1/12/2023 19:00	22	22.0	19.5	-2.5
2A	1/12/2023 20:00	22	22.0	19.5	-2.5
2A	1/12/2023 21:00	22	22.0	19.5	-2.5
2A	1/12/2023 22:00	22	22.0	19.5	-2.5
2A	1/12/2023 23:00	22	22.0	19.5	-2.5
2A	1/13/2023 0:00	22	22.0	17.2	-4.8
2A	1/13/2023 1:00	22	22.0	17.2	-4.8
2A	1/13/2023 2:00	22	22.0	17.2	-4.8
2A	1/13/2023 3:00	22	22.0	17.2	-4.8
2A	1/13/2023 4:00	22	22.0	17.2	-4.8
2A	1/13/2023 5:00	22	22.0	17.2	-4.8
2A	1/13/2023 6:00	22	22.0	17.2	-4.8
2A	1/13/2023 7:00	22	22.0	17.2	-4.8
2A	1/13/2023 8:00	22	22.0	15.7	-6.3
2A	1/13/2023 9:00	22	22.0	15.7	-6.3
2A	1/13/2023 10:00	22	22.0	15.7	-6.3
2A	1/13/2023 11:00	22	22.0	15.7	-6.3
2A	1/13/2023 12:00	22	22.0	18.4	-3.6
2A	1/13/2023 13:00	22	22.0	18.4	-3.6
2A	1/13/2023 14:00	22	22.0	18.4	-3.6
2A	1/13/2023 15:00	22	22.0	18.4	-3.6
2A	1/13/2023 16:00	22	22.0	13.9	-8.1
2A	1/13/2023 17:00	22	22.0	13.9	-8.1
2A	1/13/2023 18:00	22	22.0	13.9	-8.1
2A	1/13/2023 19:00	22	22.0	13.9	-8.1
2A	1/13/2023 20:00	22	22.0	22.3	0.3
2A	1/13/2023 21:00	16	22.0	22.3	0.3
2A	1/13/2023 22:00	16	22.0	22.3	0.3
2A	1/13/2023 23:00	16	22.0	22.3	0.3
2A	1/14/2023 0:00	16	22.0	23.0	1.0
2A	1/14/2023 1:00	16	22.0	23.0	1.0
2A	1/14/2023 2:00	16	22.0	23.0	1.0
2A	1/14/2023 3:00	16	22.0	23.0	1.0
2A	1/14/2023 4:00	16	22.0	23.0	1.0
2A	1/14/2023 5:00	16	22.0	23.0	1.0
2A	1/14/2023 6:00	18	22.0	23.0	1.0
2A	1/14/2023 7:00	18	22.0	23.0	1.0
2A	1/14/2023 8:00	18	22.0	18.4	-3.6
2A	1/14/2023 9:00	18	22.0	18.4	-3.6

2A	1/14/2023 10:00	18	22.0	18.4	-3.6
2A	1/14/2023 11:00	18	22.0	18.4	-3.6
2A	1/14/2023 12:00	18	22.0	18.4	-3.6
2A	1/14/2023 13:00	18	22.0	18.4	-3.6
2A	1/14/2023 14:00	18	22.0	18.4	-3.6
2A	1/14/2023 15:00	18	22.0	18.4	-3.6
2A	1/14/2023 16:00	16	22.0	16.7	-5.3
2A	1/14/2023 17:00	16	22.0	16.7	-5.3
2A	1/14/2023 18:00	16	22.0	16.7	-5.3
2A	1/14/2023 19:00	16	22.0	16.7	-5.3
2A	1/14/2023 20:00	16	22.0	22.3	0.3
2A	1/14/2023 21:00	16	22.0	22.3	0.3
2A	1/14/2023 22:00	17	22.0	22.3	0.3
2A	1/14/2023 23:00	17	22.0	22.3	0.3
2A	1/15/2023 0:00	17	22.0	20.1	-1.9
2A	1/15/2023 1:00	17	22.0	20.1	-1.9
2A	1/15/2023 2:00	17	22.0	20.1	-1.9
2A	1/15/2023 3:00	17	22.0	20.1	-1.9
2A	1/15/2023 4:00	17	22.0	20.1	-1.9
2A	1/15/2023 5:00	17	22.0	20.1	-1.9
2A	1/15/2023 6:00	17	22.0	20.1	-1.9
2A	1/15/2023 7:00	18	22.0	20.1	-1.9
2A	1/15/2023 8:00	18	22.0	21.0	-1.0
2A	1/15/2023 9:00	18	22.0	21.0	-1.0
2A	1/15/2023 10:00	18	22.0	21.0	-1.0
2A	1/15/2023 11:00	18	22.0	21.0	-1.0
2A	1/15/2023 12:00	18	22.0	21.0	-1.0
2A	1/15/2023 13:00	18	22.0	21.0	-1.0
2A	1/15/2023 14:00	18	22.0	21.0	-1.0
2A	1/15/2023 15:00	18	22.0	21.0	-1.0
2A	1/15/2023 16:00	18	22.0	19.5	-2.5
2A	1/15/2023 17:00	18	22.0	19.5	-2.5
2A	1/15/2023 18:00	18	22.0	19.5	-2.5
2A	1/15/2023 19:00	18	22.0	19.5	-2.5
2A	1/15/2023 20:00	18	22.0	19.5	-2.5
2A	1/15/2023 21:00	18	22.0	19.5	-2.5
2A	1/15/2023 22:00	18	22.0	19.5	-2.5
2A	1/15/2023 23:00	18	22.0	19.5	-2.5
2A	1/16/2023 0:00	20	22.0	17.2	-4.8
2A	1/16/2023 1:00	20	22.0	17.2	-4.8
2A	1/16/2023 2:00	20	22.0	17.2	-4.8
2A	1/16/2023 3:00	20	22.0	17.2	-4.8
2A	1/16/2023 4:00	20	22.0	17.2	-4.8
2A	1/16/2023 5:00	20	22.0	17.2	-4.8
2A	1/16/2023 6:00	20	22.0	17.2	-4.8
2A	1/16/2023 7:00	20	22.0	17.2	-4.8
2A	1/16/2023 8:00	20	22.0	21.0	-1.0

2A	1/16/2023 9:00	20	22.0	21.0	-1.0
2A	1/16/2023 10:00	20	22.0	21.0	-1.0
2A	1/16/2023 11:00	20	22.0	21.0	-1.0
2A	1/16/2023 12:00	20	22.0	23.6	1.6
2A	1/16/2023 13:00	18	22.0	23.6	1.6
2A	1/16/2023 14:00	18	22.0	23.6	1.6
2A	1/16/2023 15:00	18	22.0	23.6	1.6
2A	1/16/2023 16:00	18	22.0	22.3	0.3
2A	1/16/2023 17:00	18	22.0	22.3	0.3
2A	1/16/2023 18:00	18	22.0	22.3	0.3
2A	1/16/2023 19:00	18	22.0	22.3	0.3
2A	1/16/2023 20:00	18	22.0	19.5	-2.5
2A	1/16/2023 21:00	18	22.0	19.5	-2.5
2A	1/16/2023 22:00	18	22.0	19.5	-2.5
2A	1/16/2023 23:00	19	22.0	19.5	-2.5
2A	1/17/2023 0:00	20	22.0	17.2	-4.8
2A	1/17/2023 1:00	20	22.0	17.2	-4.8
2A	1/17/2023 2:00	20	22.0	17.2	-4.8
2A	1/17/2023 3:00	20	22.0	17.2	-4.8
2A	1/17/2023 4:00	20	22.0	17.2	-4.8
2A	1/17/2023 5:00	20	22.0	17.2	-4.8
2A	1/17/2023 6:00	20	22.0	17.2	-4.8
2A	1/17/2023 7:00	20	22.0	16.1	-5.9
2A	1/17/2023 8:00	20	22.0	18.9	-3.1
2A	1/17/2023 9:00	20	22.0	18.9	-3.1
2A	1/17/2023 10:00	20	22.0	18.9	-3.1
2A	1/17/2023 11:00	20	22.0	18.9	-3.1
2A	1/17/2023 12:00	20	22.0	23.1	1.1
2A	1/17/2023 13:00	20	22.0	23.1	1.1
2A	1/17/2023 14:00	20	22.0	23.1	1.1
2A	1/17/2023 15:00	19	22.0	24.1	2.1
2A	1/17/2023 16:00	19	22.0	19.5	-2.5
2A	1/17/2023 17:00	19	22.0	19.5	-2.5
2A	1/17/2023 18:00	19	22.0	19.5	-2.5
2A	1/17/2023 19:00	18	22.0	19.5	-2.5
2A	1/17/2023 20:00	20	22.0	17.8	-4.2
2A	1/17/2023 21:00	20	22.0	20.0	-2.0
2A	1/17/2023 22:00	20	22.0	20.0	-2.0
2A	1/17/2023 23:00	20	22.0	20.0	-2.0
2A	1/18/2023 0:00	20	22.0	20.1	-1.9
2A	1/18/2023 1:00	20	22.0	20.1	-1.9
2A	1/18/2023 2:00	20	22.0	20.1	-1.9
2A	1/18/2023 3:00	20	22.0	20.1	-1.9
2A	1/18/2023 4:00	20	22.0	20.1	-1.9
2A	1/18/2023 5:00	20	22.0	20.1	-1.9
2A	1/18/2023 6:00	21	22.0	20.1	-1.9
2A	1/18/2023 7:00	21	22.0	20.1	-1.9

2A	1/18/2023 8:00	21	22.0	18.4	-3.6
2A	1/18/2023 9:00	21	22.0	18.4	-3.6
2A	1/18/2023 10:00	21	22.0	18.4	-3.6
2A	1/18/2023 11:00	21	22.0	18.4	-3.6
2A	1/18/2023 12:00	21	22.0	18.4	-3.6
2A	1/18/2023 13:00	21	22.0	18.4	-3.6
2A	1/18/2023 14:00	21	22.0	18.4	-3.6
2A	1/18/2023 15:00	19	22.0	18.4	-3.6
2A	1/18/2023 16:00	19	22.0	19.5	-2.5
2A	1/18/2023 17:00	19	22.0	19.5	-2.5
2A	1/18/2023 18:00	19	22.0	19.5	-2.5
2A	1/18/2023 19:00	19	22.0	19.5	-2.5
2A	1/18/2023 20:00	19	22.0	16.7	-5.3
2A	1/18/2023 21:00	19	22.0	16.7	-5.3
2A	1/18/2023 22:00	19	22.0	16.7	-5.3
2A	1/18/2023 23:00	19	22.0	16.7	-5.3
2A	1/19/2023 0:00	22	22.0	17.2	-4.8
2A	1/19/2023 1:00	22	22.0	17.2	-4.8
2A	1/19/2023 2:00	22	22.0	17.2	-4.8
2A	1/19/2023 3:00	22	22.0	17.2	-4.8
2A	1/19/2023 4:00	22	22.0	17.2	-4.8
2A	1/19/2023 5:00	22	22.0	17.2	-4.8
2A	1/19/2023 6:00	22	22.0	17.2	-4.8
2A	1/19/2023 7:00	22	22.0	17.2	-4.8
2A	1/19/2023 8:00	22	22.0	18.4	-3.6
2A	1/19/2023 9:00	22	22.0	18.4	-3.6
2A	1/19/2023 10:00	22	22.0	18.4	-3.6
2A	1/19/2023 11:00	19	22.0	18.4	-3.6
2A	1/19/2023 12:00	19	22.0	18.4	-3.6
2A	1/19/2023 13:00	19	22.0	18.4	-3.6
2A	1/19/2023 14:00	19	22.0	18.4	-3.6
2A	1/19/2023 15:00	17	22.0	18.4	-3.6
2A	1/19/2023 16:00	17	22.0	16.7	-5.3
2A	1/19/2023 17:00	17	22.0	16.7	-5.3
2A	1/19/2023 18:00	17	22.0	16.7	-5.3
2A	1/19/2023 19:00	17	22.0	16.7	-5.3
2A	1/19/2023 20:00	19	19.0	16.7	-2.3
2A	1/19/2023 21:00	19	19.0	16.7	-2.3
2A	1/19/2023 22:00	19	19.0	16.7	-2.3
2A	1/19/2023 23:00	19	19.0	16.7	-2.3
2A	1/20/2023 0:00	19	19.0	14.4	-4.7
2A	1/20/2023 1:00	19	19.0	14.4	-4.7
2A	1/20/2023 2:00	19	19.0	14.4	-4.7
2A	1/20/2023 3:00	19	19.0	14.4	-4.7
2A	1/20/2023 4:00	19	19.0	14.4	-4.7
2A	1/20/2023 5:00	19	19.0	14.4	-4.7
2A	1/20/2023 6:00	19	19.0	14.4	-4.7

2A	1/20/2023 7:00	19	19.0	14.4	-4.7
2A	1/20/2023 8:00	22	19.0	13.1	-5.9
2A	1/20/2023 9:00	22	19.0	13.1	-5.9
2A	1/20/2023 10:00	22	19.0	13.1	-5.9
2A	1/20/2023 11:00	22	19.0	13.1	-5.9
2A	1/20/2023 12:00	22	19.0	15.7	-3.3
2A	1/20/2023 13:00	22	19.0	15.7	-3.3
2A	1/20/2023 14:00	22	19.0	15.7	-3.3
2A	1/20/2023 15:00	22	19.0	15.7	-3.3
2A	1/20/2023 16:00	22	19.0	8.4	-10.7
2A	1/20/2023 17:00	22	19.0	8.4	-10.7
2A	1/20/2023 18:00	22	19.0	8.4	-10.7
2A	1/20/2023 19:00	17	22.0	8.4	-13.7
2A	1/20/2023 20:00	22	16.0	16.7	0.7
2A	1/20/2023 21:00	22	16.0	16.7	0.7
2A	1/20/2023 22:00	22	16.0	16.7	0.7
2A	1/20/2023 23:00	22	16.0	16.7	0.7
2A	1/21/2023 0:00	22	16.0	20.1	4.1
2A	1/21/2023 1:00	17	22.0	20.1	-1.9
2A	1/21/2023 2:00	17	22.0	20.1	-1.9
2A	1/21/2023 3:00	17	22.0	20.1	-1.9
2A	1/21/2023 4:00	17	22.0	20.1	-1.9
2A	1/21/2023 5:00	17	22.0	20.1	-1.9
2A	1/21/2023 6:00	17	22.0	20.1	-1.9
2A	1/21/2023 7:00	17	22.0	20.1	-1.9
2A	1/21/2023 8:00	17	22.0	16.8	-5.2
2A	1/21/2023 9:00	17	22.0	16.8	-5.2
2A	1/21/2023 10:00	17	22.0	16.8	-5.2
2A	1/21/2023 11:00	17	22.0	16.8	-5.2
2A	1/21/2023 12:00	17	22.0	18.9	-3.1
2A	1/21/2023 13:00	17	22.0	18.9	-3.1
2A	1/21/2023 14:00	17	22.0	18.9	-3.1
2A	1/21/2023 15:00	17	22.0	18.9	-3.1
2A	1/21/2023 16:00	17	22.0	20.0	-2.0
2A	1/21/2023 17:00	17	22.0	20.0	-2.0
2A	1/21/2023 18:00	17	22.0	20.0	-2.0
2A	1/21/2023 19:00	17	22.0	20.0	-2.0
2A	1/21/2023 20:00	15	22.0	17.8	-4.2
2A	1/21/2023 21:00	15	22.0	17.8	-4.2
2A	1/21/2023 22:00	15	22.0	17.8	-4.2
2A	1/21/2023 23:00	15	22.0	17.8	-4.2
2A	1/22/2023 0:00	15	22.0	17.2	-4.8
2A	1/22/2023 1:00	15	22.0	17.2	-4.8
2A	1/22/2023 2:00	15	22.0	17.2	-4.8
2A	1/22/2023 3:00	15	22.0	17.2	-4.8
2A	1/22/2023 4:00	15	22.0	17.2	-4.8
2A	1/22/2023 5:00	15	22.0	17.2	-4.8

2A	1/22/2023 6:00	15	22.0	17.2	-4.8
2A	1/22/2023 7:00	15	22.0	17.2	-4.8
2A	1/22/2023 8:00	15	22.0	15.7	-6.3
2A	1/22/2023 9:00	15	22.0	15.7	-6.3
2A	1/22/2023 10:00	15	22.0	15.7	-6.3
2A	1/22/2023 11:00	15	22.0	15.7	-6.3
2A	1/22/2023 12:00	15	22.0	18.4	-3.6
2A	1/22/2023 13:00	15	22.0	18.4	-3.6
2A	1/22/2023 14:00	15	22.0	18.4	-3.6
2A	1/22/2023 15:00	15	22.0	18.4	-3.6
2A	1/22/2023 16:00	15	22.0	16.7	-5.3
2A	1/22/2023 17:00	15	22.0	16.7	-5.3
2A	1/22/2023 18:00	15	22.0	16.7	-5.3
2A	1/22/2023 19:00	17	22.0	19.5	-2.5
2A	1/22/2023 20:00	17	22.0	19.5	-2.5
2A	1/22/2023 21:00	17	22.0	19.5	-2.5
2A	1/22/2023 22:00	17	22.0	19.5	-2.5
2A	1/22/2023 23:00	17	22.0	19.5	-2.5
2A	1/23/2023 0:00	17	22.0	20.1	-1.9
2A	1/23/2023 1:00	17	22.0	20.1	-1.9
2A	1/23/2023 2:00	17	22.0	20.1	-1.9
2A	1/23/2023 3:00	18	22.0	20.1	-1.9
2A	1/23/2023 4:00	18	22.0	20.1	-1.9
2A	1/23/2023 5:00	18	22.0	20.1	-1.9
2A	1/23/2023 6:00	19	22.0	20.1	-1.9
2A	1/23/2023 7:00	19	22.0	20.1	-1.9
2A	1/23/2023 8:00	19	22.0	18.4	-3.6
2A	1/23/2023 9:00	19	22.0	18.4	-3.6
2A	1/23/2023 10:00	19	22.0	18.4	-3.6
2A	1/23/2023 11:00	18	22.0	18.4	-3.6
2A	1/23/2023 12:00	19	22.0	15.7	-6.3
2A	1/23/2023 13:00	19	22.0	15.7	-6.3
2A	1/23/2023 14:00	19	22.0	15.7	-6.3
2A	1/23/2023 15:00	19	22.0	15.7	-6.3
2A	1/23/2023 16:00	19	18.0	15.6	-2.4
2A	1/23/2023 17:00	19	18.0	15.6	-2.4
2A	1/23/2023 18:00	19	18.0	15.6	-2.4
2A	1/23/2023 19:00	19	18.0	15.6	-2.4
2A	1/23/2023 20:00	19	18.0	13.9	-4.1
2A	1/23/2023 21:00	19	18.0	13.9	-4.1
2A	1/23/2023 22:00	19	18.0	13.9	-4.1
2A	1/23/2023 23:00	19	21.0	11.1	-9.9
2A	1/24/2023 0:00	19	21.0	14.4	-6.7
2A	1/24/2023 1:00	19	21.0	14.4	-6.7
2A	1/24/2023 2:00	19	21.0	14.4	-6.7
2A	1/24/2023 3:00	19	21.0	14.4	-6.7
2A	1/24/2023 4:00	20	21.0	14.4	-6.7

2A	1/24/2023 5:00	20	21.0	14.4	-6.7
2A	1/24/2023 6:00	20	21.0	14.4	-6.7
2A	1/24/2023 7:00	20	21.0	14.4	-6.7
2A	1/24/2023 8:00	20	21.0	13.1	-7.9
2A	1/24/2023 9:00	20	21.0	13.1	-7.9
2A	1/24/2023 10:00	20	21.0	13.1	-7.9
2A	1/24/2023 11:00	20	21.0	13.1	-7.9
2A	1/24/2023 12:00	20	21.0	13.1	-7.9
2A	1/24/2023 13:00	20	21.0	13.1	-7.9
2A	1/24/2023 14:00	20	21.0	13.1	-7.9
2A	1/24/2023 15:00	20	21.0	13.1	-7.9
2A	1/24/2023 16:00	20	21.0	13.9	-7.1
2A	1/24/2023 17:00	20	21.0	13.9	-7.1
2A	1/24/2023 18:00	20	21.0	13.9	-7.1
2A	1/24/2023 19:00	20	21.0	13.9	-7.1
2A	1/24/2023 20:00	17	21.0	16.7	-4.3
2A	1/24/2023 21:00	17	21.0	16.7	-4.3
2A	1/24/2023 22:00	17	21.0	16.7	-4.3
2A	1/24/2023 23:00	17	21.0	16.7	-4.3
2A	1/25/2023 0:00	17	21.0	17.2	-3.8
2A	1/25/2023 1:00	17	21.0	17.2	-3.8
2A	1/25/2023 2:00	17	21.0	17.2	-3.8
2A	1/25/2023 3:00	17	21.0	17.2	-3.8
2A	1/25/2023 4:00	17	21.0	17.2	-3.8
2A	1/25/2023 5:00	18	23.0	17.2	-5.8
2A	1/25/2023 6:00	18	23.0	17.2	-5.8
2A	1/25/2023 7:00	19	23.0	17.2	-5.8
2A	1/25/2023 8:00	19	23.0	18.4	-4.6
2A	1/25/2023 9:00	19	23.0	18.4	-4.6
2A	1/25/2023 10:00	19	23.0	16.8	-6.2
2A	1/25/2023 11:00	19	23.0	18.4	-4.6
2A	1/25/2023 12:00	19	23.0	21.0	-2.0
2A	1/25/2023 13:00	19	23.0	17.8	-5.2
2A	1/25/2023 14:00	19	23.0	21.0	-2.0
2A	1/25/2023 15:00	19	23.0	21.0	-2.0
2A	1/25/2023 16:00	19	23.0	19.5	-3.5
2A	1/25/2023 17:00	19	23.0	19.5	-3.5
2A	1/25/2023 18:00	19	23.0	19.5	-3.5
2A	1/25/2023 19:00	19	23.0	19.5	-3.5
2A	1/25/2023 20:00	19	23.0	22.3	-0.7
2A	1/25/2023 21:00	22	23.0	22.3	-0.7
2A	1/25/2023 22:00	22	23.0	22.3	-0.7
2A	1/25/2023 23:00	22	23.0	22.3	-0.7
2A	1/26/2023 0:00	22	23.0	23.0	0.0
2A	1/26/2023 1:00	22	23.0	23.0	0.0
2A	1/26/2023 2:00	22	23.0	23.0	0.0
2A	1/26/2023 3:00	22	23.0	23.0	0.0

2A	1/26/2023 4:00	22	23.0	23.0	0.0
2A	1/26/2023 5:00	22	23.0	23.0	0.0
2A	1/26/2023 6:00	22	23.0	23.0	0.0
2A	1/26/2023 7:00	22	23.0	23.0	0.0
2A	1/26/2023 8:00	22	23.0	21.0	-2.0
2A	1/26/2023 9:00	22	23.0	21.0	-2.0
2A	1/26/2023 10:00	22	23.0	21.0	-2.0
2A	1/26/2023 11:00	22	23.0	21.0	-2.0
2A	1/26/2023 12:00	22	23.0	23.6	0.6
2A	1/26/2023 13:00	22	23.0	23.6	0.6
2A	1/26/2023 14:00	22	23.0	23.6	0.6
2A	1/26/2023 15:00	22	23.0	23.6	0.6
2A	1/26/2023 16:00	22	23.0	22.3	-0.7
2A	1/26/2023 17:00	22	23.0	22.3	-0.7
2A	1/26/2023 18:00	22	23.0	22.3	-0.7
2A	1/26/2023 19:00	22	23.0	22.3	-0.7
2A	1/26/2023 20:00	22	23.0	22.3	-0.7
2A	1/26/2023 21:00	22	23.0	19.5	-3.5
2A	1/26/2023 22:00	22	23.0	19.5	-3.5
2A	1/26/2023 23:00	22	23.0	19.5	-3.5
2A	1/27/2023 0:00	22	23.0	17.2	-5.8
2A	1/27/2023 1:00	22	23.0	17.2	-5.8
2A	1/27/2023 2:00	22	23.0	17.2	-5.8
2A	1/27/2023 3:00	22	23.0	17.2	-5.8
2A	1/27/2023 4:00	22	23.0	17.2	-5.8
2A	1/27/2023 5:00	22	23.0	17.2	-5.8
2A	1/27/2023 6:00	22	23.0	17.2	-5.8
2A	1/27/2023 7:00	22	23.0	17.2	-5.8
2A	1/27/2023 8:00	22	23.0	18.4	-4.6
2A	1/27/2023 9:00	22	23.0	18.4	-4.6
2A	1/27/2023 10:00	22	23.0	18.4	-4.6
2A	1/27/2023 11:00	22	23.0	18.4	-4.6
2A	1/27/2023 12:00	22	23.0	23.6	0.6
2A	1/27/2023 13:00	22	23.0	23.6	0.6
2A	1/27/2023 14:00	22	23.0	23.6	0.6
2A	1/27/2023 15:00	17	23.0	22.3	-0.7
2A	1/27/2023 16:00	17	23.0	16.7	-6.3
2A	1/27/2023 17:00	17	23.0	16.7	-6.3
2A	1/27/2023 18:00	17	23.0	16.7	-6.3
2A	1/27/2023 19:00	15	23.0	16.7	-6.3
2A	1/27/2023 20:00	15	23.0	22.3	-0.7
2A	1/27/2023 21:00	15	23.0	22.3	-0.7
2A	1/27/2023 22:00	15	23.0	22.3	-0.7
2A	1/27/2023 23:00	15	23.0	22.3	-0.7
2A	1/28/2023 0:00	15	23.0	20.1	-2.9
2A	1/28/2023 1:00	21	23.0	20.1	-2.9
2A	1/28/2023 2:00	21	23.0	20.1	-2.9

2A	1/28/2023 3:00	21	23.0	20.1	-2.9
2A	1/28/2023 4:00	21	23.0	20.1	-2.9
2A	1/28/2023 5:00	22	23.0	20.1	-2.9
2A	1/28/2023 6:00	22	23.0	20.1	-2.9
2A	1/28/2023 7:00	22	23.0	20.1	-2.9
2A	1/28/2023 8:00	22	23.0	21.0	-2.0
2A	1/28/2023 9:00	21	23.0	21.0	-2.0
2A	1/28/2023 10:00	21	23.0	21.0	-2.0
2A	1/28/2023 11:00	21	23.0	21.0	-2.0
2A	1/28/2023 12:00	21	23.0	23.6	0.6
2A	1/28/2023 13:00	21	23.0	23.6	0.6
2A	1/28/2023 14:00	21	23.0	23.6	0.6
2A	1/28/2023 15:00	20	23.0	23.6	0.6
2A	1/28/2023 16:00	20	23.0	22.3	-0.7
2A	1/28/2023 17:00	20	23.0	22.3	-0.7
2A	1/28/2023 18:00	20	23.0	22.3	-0.7
2A	1/28/2023 19:00	20	23.0	22.3	-0.7
2A	1/28/2023 20:00	20	23.0	22.3	-0.7
2A	1/28/2023 21:00	20	23.0	22.3	-0.7
2A	1/28/2023 22:00	21	23.0	22.3	-0.7
2A	1/28/2023 23:00	21	23.0	22.3	-0.7
2A	1/29/2023 0:00	21	23.0	20.1	-2.9
2A	1/29/2023 1:00	21	23.0	20.1	-2.9
2A	1/29/2023 2:00	21	23.0	20.1	-2.9
2A	1/29/2023 3:00	21	23.0	20.1	-2.9
2A	1/29/2023 4:00	21	23.0	20.1	-2.9
2A	1/29/2023 5:00	21	23.0	20.1	-2.9
2A	1/29/2023 6:00	21	23.0	20.1	-2.9
2A	1/29/2023 7:00	21	23.0	17.2	-5.8
2A	1/29/2023 8:00	21	23.0	18.4	-4.6
2A	1/29/2023 9:00	21	23.0	18.4	-4.6
2A	1/29/2023 10:00	21	23.0	18.4	-4.6
2A	1/29/2023 11:00	21	23.0	18.4	-4.6
2A	1/29/2023 12:00	21	23.0	21.0	-2.0
2A	1/29/2023 13:00	21	23.0	21.0	-2.0
2A	1/29/2023 14:00	21	23.0	21.0	-2.0
2A	1/29/2023 15:00	21	23.0	21.0	-2.0
2A	1/29/2023 16:00	21	23.0	19.5	-3.5
2A	1/29/2023 17:00	21	23.0	19.5	-3.5
2A	1/29/2023 18:00	21	23.0	19.5	-3.5
2A	1/29/2023 19:00	21	23.0	19.5	-3.5
2A	1/29/2023 20:00	21	23.0	19.5	-3.5
2A	1/29/2023 21:00	21	23.0	19.5	-3.5
2A	1/29/2023 22:00	21	23.0	19.5	-3.5
2A	1/29/2023 23:00	19	23.0	21.6	-1.4
2A	1/30/2023 0:00	19	23.0	17.2	-5.8
2A	1/30/2023 1:00	19	23.0	17.2	-5.8

2A	1/30/2023 2:00	19	23.0	17.2	-5.8
2A	1/30/2023 3:00	19	23.0	17.2	-5.8
2A	1/30/2023 4:00	19	23.0	17.2	-5.8
2A	1/30/2023 5:00	19	23.0	17.2	-5.8
2A	1/30/2023 6:00	19	23.0	17.2	-5.8
2A	1/30/2023 7:00	19	23.0	17.2	-5.8
2A	1/30/2023 8:00	21	23.0	18.4	-4.6
2A	1/30/2023 9:00	21	23.0	18.4	-4.6
2A	1/30/2023 10:00	20	23.0	16.8	-6.2
2A	1/30/2023 11:00	20	23.0	18.4	-4.6
2A	1/30/2023 12:00	20	23.0	21.0	-2.0
2A	1/30/2023 13:00	20	23.0	21.0	-2.0
2A	1/30/2023 14:00	20	23.0	17.8	-5.2
2A	1/30/2023 15:00	20	23.0	21.0	-2.0
2A	1/30/2023 16:00	20	23.0	22.3	-0.7
2A	1/30/2023 17:00	20	23.0	22.3	-0.7
2A	1/30/2023 18:00	20	23.0	22.3	-0.7
2A	1/30/2023 19:00	19	23.0	20.6	-2.4
2A	1/30/2023 20:00	19	23.0	16.7	-6.3
2A	1/30/2023 21:00	19	23.0	16.7	-6.3
2A	1/30/2023 22:00	18	23.0	16.7	-6.3
2A	1/30/2023 23:00	18	23.0	16.7	-6.3
2A	1/31/2023 0:00	18	18.0	11.5	-6.5
2A	1/31/2023 1:00	18	18.0	11.5	-6.5
2A	1/31/2023 2:00	18	18.0	11.5	-6.5
2A	1/31/2023 3:00	18	18.0	11.5	-6.5
2A	1/31/2023 4:00	18	18.0	11.5	-6.5
2A	1/31/2023 5:00	18	18.0	11.5	-6.5
2A	1/31/2023 6:00	18	18.0	11.5	-6.5
2A	1/31/2023 7:00	18	23.0	11.5	-11.5
2A	1/31/2023 8:00	18	23.0	14.2	-8.8
2A	1/31/2023 9:00	18	23.0	14.2	-8.8
2A	1/31/2023 10:00	18	23.0	15.7	-7.3
2A	1/31/2023 11:00	18	23.0	15.7	-7.3
2A	1/31/2023 12:00	18	23.0	21.0	-2.0
2A	1/31/2023 13:00	18	23.0	21.0	-2.0
2A	1/31/2023 14:00	18	23.0	21.0	-2.0
2A	1/31/2023 15:00	19	23.0	21.0	-2.0
2A	1/31/2023 16:00	19	23.0	22.3	-0.7
2A	1/31/2023 17:00	19	23.0	22.3	-0.7
2A	1/31/2023 18:00	19	23.0	22.3	-0.7
2A	1/31/2023 19:00	20	23.0	22.3	-0.7
2A	1/31/2023 20:00	20	23.0	22.3	-0.7
2A	1/31/2023 21:00	20	23.0	22.3	-0.7
2A	1/31/2023 22:00	20	23.0	22.3	-0.7
2A	1/31/2023 23:00	20	23.0	22.3	-0.7
2A	2/1/2023 0:00	20	23.0	20.1	-2.9

2A	2/1/2023 1:00	20	23.0	20.1	-2.9
2A	2/1/2023 2:00	22	23.0	20.1	-2.9
2A	2/1/2023 3:00	22	23.0	20.1	-2.9
2A	2/1/2023 4:00	22	23.0	20.1	-2.9
2A	2/1/2023 5:00	22	23.0	20.1	-2.9
2A	2/1/2023 6:00	22	23.0	20.1	-2.9
2A	2/1/2023 7:00	22	23.0	20.1	-2.9
2A	2/1/2023 8:00	22	23.0	21.0	-2.0
2A	2/1/2023 9:00	22	23.0	21.0	-2.0
2A	2/1/2023 10:00	22	23.0	21.0	-2.0
2A	2/1/2023 11:00	22	23.0	21.0	-2.0
2A	2/1/2023 12:00	22	23.0	21.0	-2.0
2A	2/1/2023 13:00	22	23.0	21.0	-2.0
2A	2/1/2023 14:00	22	23.0	21.0	-2.0
2A	2/1/2023 15:00	17	23.0	18.9	-4.1
2A	2/1/2023 16:00	17	23.0	20.0	-3.0
2A	2/1/2023 17:00	17	23.0	20.0	-3.0
2A	2/1/2023 18:00	17	23.0	20.0	-3.0
2A	2/1/2023 19:00	17	23.0	20.0	-3.0
2A	2/1/2023 20:00	20	23.0	19.5	-3.5
2A	2/1/2023 21:00	20	23.0	19.5	-3.5
2A	2/1/2023 22:00	21	23.0	19.5	-3.5
2A	2/1/2023 23:00	21	23.0	19.5	-3.5
2A	2/2/2023 0:00	21	23.0	17.2	-5.8
2A	2/2/2023 1:00	21	23.0	17.2	-5.8
2A	2/2/2023 2:00	21	23.0	17.2	-5.8
2A	2/2/2023 3:00	21	23.0	17.2	-5.8
2A	2/2/2023 4:00	21	23.0	17.2	-5.8
2A	2/2/2023 5:00	21	23.0	17.2	-5.8
2A	2/2/2023 6:00	21	23.0	17.2	-5.8
2A	2/2/2023 7:00	21	23.0	17.2	-5.8
2A	2/2/2023 8:00	22	23.0	21.0	-2.0
2A	2/2/2023 9:00	22	23.0	21.0	-2.0
2A	2/2/2023 10:00	22	23.0	21.0	-2.0
2A	2/2/2023 11:00	22	23.0	21.0	-2.0
2A	2/2/2023 12:00	22	23.0	21.0	-2.0
2A	2/2/2023 13:00	22	23.0	21.0	-2.0
2A	2/2/2023 14:00	22	23.0	21.0	-2.0
2A	2/2/2023 15:00	22	23.0	21.0	-2.0
2A	2/2/2023 16:00	22	23.0	22.3	-0.7
2A	2/2/2023 17:00	22	23.0	22.3	-0.7
2A	2/2/2023 18:00	22	23.0	22.3	-0.7
2A	2/2/2023 19:00	22	23.0	22.3	-0.7
2A	2/2/2023 20:00	22	23.0	22.3	-0.7
2A	2/2/2023 21:00	22	23.0	22.3	-0.7
2A	2/2/2023 22:00	22	23.0	22.3	-0.7
2A	2/2/2023 23:00	22	23.0	22.3	-0.7

2A	2/3/2023 0:00	23	23.0	20.1	-2.9
2A	2/3/2023 1:00	23	23.0	20.1	-2.9
2A	2/3/2023 2:00	23	23.0	20.1	-2.9
2A	2/3/2023 3:00	23	23.0	20.1	-2.9
2A	2/3/2023 4:00	23	23.0	20.1	-2.9
2A	2/3/2023 5:00	23	23.0	20.1	-2.9
2A	2/3/2023 6:00	23	23.0	20.1	-2.9
2A	2/3/2023 7:00	23	23.0	20.1	-2.9
2A	2/3/2023 8:00	23	23.0	18.4	-4.6
2A	2/3/2023 9:00	23	23.0	18.4	-4.6
2A	2/3/2023 10:00	23	23.0	18.4	-4.6
2A	2/3/2023 11:00	23	23.0	18.4	-4.6
2A	2/3/2023 12:00	21	23.0	21.0	-2.0
2A	2/3/2023 13:00	19	23.0	21.0	-2.0
2A	2/3/2023 14:00	19	23.0	21.0	-2.0
2A	2/3/2023 15:00	19	23.0	21.0	-2.0
2A	2/3/2023 16:00	19	23.0	16.7	-6.3
2A	2/3/2023 17:00	19	23.0	16.7	-6.3
2A	2/3/2023 18:00	19	23.0	16.7	-6.3
2A	2/3/2023 19:00	19	23.0	16.7	-6.3
2A	2/3/2023 20:00	21	23.0	22.3	-0.7
2A	2/3/2023 21:00	21	23.0	22.3	-0.7
2A	2/3/2023 22:00	21	23.0	22.3	-0.7
2A	2/3/2023 23:00	21	23.0	22.3	-0.7
2A	2/4/2023 0:00	21	23.0	20.1	-2.9
2A	2/4/2023 1:00	21	23.0	20.1	-2.9
2A	2/4/2023 2:00	21	23.0	20.1	-2.9
2A	2/4/2023 3:00	21	23.0	20.1	-2.9
2A	2/4/2023 4:00	21	23.0	20.1	-2.9
2A	2/4/2023 5:00	21	23.0	20.1	-2.9
2A	2/4/2023 6:00	21	23.0	20.1	-2.9
2A	2/4/2023 7:00	21	23.0	20.1	-2.9
2A	2/4/2023 8:00	21	23.0	21.0	-2.0
2A	2/4/2023 9:00	21	23.0	21.0	-2.0
2A	2/4/2023 10:00	21	23.0	21.0	-2.0
2A	2/4/2023 11:00	21	23.0	21.0	-2.0
2A	2/4/2023 12:00	21	23.0	23.6	0.6
2A	2/4/2023 13:00	21	23.0	23.6	0.6
2A	2/4/2023 14:00	21	23.0	23.6	0.6
2A	2/4/2023 15:00	21	23.0	23.6	0.6
2A	2/4/2023 16:00	21	23.0	22.3	-0.7
2A	2/4/2023 17:00	21	23.0	22.3	-0.7
2A	2/4/2023 18:00	21	23.0	22.3	-0.7
2A	2/4/2023 19:00	21	23.0	22.3	-0.7
2A	2/4/2023 20:00	21	23.0	22.3	-0.7
2A	2/4/2023 21:00	21	23.0	22.3	-0.7
2A	2/4/2023 22:00	19	23.0	22.3	-0.7

2A	2/4/2023 23:00	19	23.0	22.3	-0.7
2A	2/5/2023 0:00	19	23.0	20.1	-2.9
2A	2/5/2023 1:00	19	23.0	20.1	-2.9
2A	2/5/2023 2:00	19	23.0	20.1	-2.9
2A	2/5/2023 3:00	19	23.0	20.1	-2.9
2A	2/5/2023 4:00	19	23.0	20.1	-2.9
2A	2/5/2023 5:00	19	23.0	20.1	-2.9
2A	2/5/2023 6:00	19	23.0	20.1	-2.9
2A	2/5/2023 7:00	19	23.0	20.1	-2.9
2A	2/5/2023 8:00	19	23.0	18.9	-4.1
2A	2/5/2023 9:00	19	23.0	18.9	-4.1
2A	2/5/2023 10:00	19	23.0	18.9	-4.1
2A	2/5/2023 11:00	19	23.0	18.9	-4.1
2A	2/5/2023 12:00	19	23.0	23.1	0.1
2A	2/5/2023 13:00	19	23.0	23.1	0.1
2A	2/5/2023 14:00	19	23.0	23.1	0.1
2A	2/5/2023 15:00	19	23.0	23.1	0.1
2A	2/5/2023 16:00	18	23.0	22.3	-0.7
2A	2/5/2023 17:00	18	23.0	22.3	-0.7
2A	2/5/2023 18:00	18	23.0	22.3	-0.7
2A	2/5/2023 19:00	18	23.0	22.3	-0.7
2A	2/5/2023 20:00	18	23.0	19.5	-3.5
2A	2/5/2023 21:00	18	23.0	19.5	-3.5
2A	2/5/2023 22:00	18	23.0	19.5	-3.5
2A	2/5/2023 23:00	21	23.0	19.5	-3.5
2A	2/6/2023 0:00	21	23.0	20.1	-2.9
2A	2/6/2023 1:00	21	23.0	20.1	-2.9
2A	2/6/2023 2:00	21	23.0	20.1	-2.9
2A	2/6/2023 3:00	21	23.0	20.1	-2.9
2A	2/6/2023 4:00	21	23.0	20.1	-2.9
2A	2/6/2023 5:00	21	23.0	20.1	-2.9
2A	2/6/2023 6:00	21	23.0	20.1	-2.9
2A	2/6/2023 7:00	21	23.0	20.1	-2.9
2A	2/6/2023 8:00	21	23.0	18.4	-4.6
2A	2/6/2023 9:00	19	23.0	16.8	-6.2
2A	2/6/2023 10:00	19	23.0	18.4	-4.6
2A	2/6/2023 11:00	19	23.0	18.4	-4.6
2A	2/6/2023 12:00	19	23.0	19.4	-3.6
2A	2/6/2023 13:00	19	23.0	21.0	-2.0
2A	2/6/2023 14:00	18	23.0	21.0	-2.0
2A	2/6/2023 15:00	18	23.0	21.0	-2.0
2A	2/6/2023 16:00	18	23.0	22.3	-0.7
2A	2/6/2023 17:00	18	23.0	20.6	-2.4
2A	2/6/2023 18:00	18	23.0	22.3	-0.7
2A	2/6/2023 19:00	18	23.0	22.3	-0.7
2A	2/6/2023 20:00	18	23.0	19.5	-3.5
2A	2/6/2023 21:00	19	23.0	19.5	-3.5

2A	2/6/2023 22:00	19	23.0	19.5	-3.5
2A	2/6/2023 23:00	21	22.0	19.5	-2.5
2A	2/7/2023 0:00	21	22.0	17.2	-4.8
2A	2/7/2023 1:00	21	22.0	17.2	-4.8
2A	2/7/2023 2:00	21	22.0	17.2	-4.8
2A	2/7/2023 3:00	21	22.0	17.2	-4.8
2A	2/7/2023 4:00	21	22.0	17.2	-4.8
2A	2/7/2023 5:00	21	22.0	17.2	-4.8
2A	2/7/2023 6:00	21	22.0	17.2	-4.8
2A	2/7/2023 7:00	21	22.0	17.2	-4.8
2A	2/7/2023 8:00	21	22.0	21.0	-1.0
2A	2/7/2023 9:00	21	22.0	21.0	-1.0
2A	2/7/2023 10:00	21	22.0	21.0	-1.0
2A	2/7/2023 11:00	21	22.0	21.0	-1.0
2A	2/7/2023 12:00	21	22.0	21.0	-1.0
2A	2/7/2023 13:00	21	22.0	21.0	-1.0
2A	2/7/2023 14:00	21	22.0	17.8	-4.2
2A	2/7/2023 15:00	21	22.0	21.0	-1.0
2A	2/7/2023 16:00	21	22.0	22.3	0.3
2A	2/7/2023 17:00	21	22.0	22.3	0.3
2A	2/7/2023 18:00	21	22.0	22.3	0.3
2A	2/7/2023 19:00	21	22.0	22.3	0.3
2A	2/7/2023 20:00	19	23.0	22.3	-0.7
2A	2/7/2023 21:00	22	23.0	22.3	-0.7
2A	2/7/2023 22:00	22	23.0	22.3	-0.7
2A	2/7/2023 23:00	22	23.0	22.3	-0.7
2A	2/8/2023 0:00	22	23.0	20.1	-2.9
2A	2/8/2023 1:00	22	23.0	20.1	-2.9
2A	2/8/2023 2:00	22	23.0	20.1	-2.9
2A	2/8/2023 3:00	22	23.0	20.1	-2.9
2A	2/8/2023 4:00	22	23.0	20.1	-2.9
2A	2/8/2023 5:00	22	23.0	20.1	-2.9
2A	2/8/2023 6:00	22	23.0	20.1	-2.9
2A	2/8/2023 7:00	22	23.0	20.1	-2.9
2A	2/8/2023 8:00	22	23.0	15.7	-7.3
2A	2/8/2023 9:00	22	23.0	15.7	-7.3
2A	2/8/2023 10:00	22	23.0	15.7	-7.3
2A	2/8/2023 11:00	22	23.0	15.7	-7.3
2A	2/8/2023 12:00	22	23.0	18.4	-4.6
2A	2/8/2023 13:00	22	23.0	18.4	-4.6
2A	2/8/2023 14:00	22	23.0	18.4	-4.6
2A	2/8/2023 15:00	22	23.0	18.4	-4.6
2A	2/8/2023 16:00	22	23.0	19.5	-3.5
2A	2/8/2023 17:00	22	23.0	19.5	-3.5
2A	2/8/2023 18:00	22	23.0	19.5	-3.5
2A	2/8/2023 19:00	22	23.0	19.5	-3.5
2A	2/8/2023 20:00	22	23.0	25.0	2.0

2A	2/8/2023 21:00	22	23.0	25.0	2.0
2A	2/8/2023 22:00	22	23.0	23.0	0.0
2A	2/8/2023 23:00	22	23.0	22.3	-0.7
2A	2/9/2023 0:00	21	22.0	20.1	-1.9
2A	2/9/2023 1:00	21	22.0	20.1	-1.9
2A	2/9/2023 2:00	21	22.0	20.1	-1.9
2A	2/9/2023 3:00	21	22.0	20.1	-1.9
2A	2/9/2023 4:00	21	22.0	20.1	-1.9
2A	2/9/2023 5:00	21	22.0	20.1	-1.9
2A	2/9/2023 6:00	21	22.0	20.1	-1.9
2A	2/9/2023 7:00	22	22.0	20.1	-1.9
2A	2/9/2023 8:00	22	22.0	18.4	-3.6
2A	2/9/2023 9:00	22	22.0	18.4	-3.6
2A	2/9/2023 10:00	22	22.0	18.4	-3.6
2A	2/9/2023 11:00	22	22.0	18.4	-3.6
2A	2/9/2023 12:00	22	22.0	23.6	1.6
2A	2/9/2023 13:00	22	22.0	23.6	1.6
2A	2/9/2023 14:00	22	22.0	23.6	1.6
2A	2/9/2023 15:00	22	22.0	23.6	1.6
2A	2/9/2023 16:00	22	22.0	22.3	0.3
2A	2/9/2023 17:00	22	22.0	22.3	0.3
2A	2/9/2023 18:00	22	22.0	22.3	0.3
2A	2/9/2023 19:00	22	22.0	22.3	0.3
2A	2/9/2023 20:00	23	22.0	25.0	3.0
2A	2/9/2023 21:00	23	22.0	25.0	3.0
2A	2/9/2023 22:00	23	22.0	25.0	3.0
2A	2/9/2023 23:00	23	22.0	25.0	3.0
2A	2/10/2023 0:00	19	23.0	20.1	-2.9
2A	2/10/2023 1:00	19	23.0	20.1	-2.9
2A	2/10/2023 2:00	19	23.0	20.1	-2.9
2A	2/10/2023 3:00	19	23.0	20.1	-2.9
2A	2/10/2023 4:00	19	23.0	20.1	-2.9
2A	2/10/2023 5:00	23	23.0	20.1	-2.9
2A	2/10/2023 6:00	23	23.0	20.1	-2.9
2A	2/10/2023 7:00	23	23.0	20.1	-2.9
2A	2/10/2023 8:00	23	23.0	21.0	-2.0
2A	2/10/2023 9:00	23	23.0	21.0	-2.0
2A	2/10/2023 10:00	23	23.0	21.0	-2.0
2A	2/10/2023 11:00	23	23.0	20.3	-2.7
2A	2/10/2023 12:00	23	23.0	21.0	-2.0
2A	2/10/2023 13:00	23	23.0	21.0	-2.0
2A	2/10/2023 14:00	23	23.0	21.0	-2.0
2A	2/10/2023 15:00	23	23.0	21.0	-2.0
2A	2/10/2023 16:00	23	23.0	19.5	-3.5
2A	2/10/2023 17:00	23	23.0	19.5	-3.5
2A	2/10/2023 18:00	23	23.0	19.5	-3.5
2A	2/10/2023 19:00	23	23.0	19.5	-3.5

2A	2/10/2023 20:00	23	23.0	22.3	-0.7
2A	2/10/2023 21:00	23	23.0	22.3	-0.7
2A	2/10/2023 22:00	23	23.0	22.3	-0.7
2A	2/10/2023 23:00	23	23.0	22.3	-0.7
2A	2/11/2023 0:00	19	23.0	17.2	-5.8
2A	2/11/2023 1:00	19	23.0	17.2	-5.8
2A	2/11/2023 2:00	19	23.0	17.2	-5.8
2A	2/11/2023 3:00	19	23.0	17.2	-5.8
2A	2/11/2023 4:00	19	23.0	17.2	-5.8
2A	2/11/2023 5:00	19	23.0	17.2	-5.8
2A	2/11/2023 6:00	19	23.0	17.2	-5.8
2A	2/11/2023 7:00	19	23.0	17.2	-5.8
2A	2/11/2023 8:00	20	23.0	18.4	-4.6
2A	2/11/2023 9:00	20	23.0	18.4	-4.6
2A	2/11/2023 10:00	20	23.0	18.4	-4.6
2A	2/11/2023 11:00	19	23.0	18.4	-4.6
2A	2/11/2023 12:00	19	23.0	23.6	0.6
2A	2/11/2023 13:00	19	23.0	23.6	0.6
2A	2/11/2023 14:00	19	23.0	23.6	0.6
2A	2/11/2023 15:00	19	23.0	23.6	0.6
2A	2/11/2023 16:00	19	23.0	19.5	-3.5
2A	2/11/2023 17:00	19	23.0	19.5	-3.5
2A	2/11/2023 18:00	19	23.0	19.5	-3.5
2A	2/11/2023 19:00	19	23.0	19.5	-3.5
2A	2/11/2023 20:00	19	23.0	22.3	-0.7
2A	2/11/2023 21:00	19	23.0	22.3	-0.7
2A	2/11/2023 22:00	19	23.0	22.3	-0.7
2A	2/11/2023 23:00	19	23.0	22.3	-0.7
2A	2/12/2023 0:00	19	23.0	20.1	-2.9
2A	2/12/2023 1:00	19	23.0	20.1	-2.9
2A	2/12/2023 2:00	19	23.0	20.1	-2.9
2A	2/12/2023 3:00	19	23.0	20.1	-2.9
2A	2/12/2023 4:00	19	23.0	20.1	-2.9
2A	2/12/2023 5:00	19	23.0	20.1	-2.9
2A	2/12/2023 6:00	19	23.0	20.1	-2.9
2A	2/12/2023 7:00	19	23.0	20.1	-2.9
2A	2/12/2023 8:00	19	23.0	18.4	-4.6
2A	2/12/2023 9:00	19	23.0	18.4	-4.6
2A	2/12/2023 10:00	19	23.0	16.8	-6.2
2A	2/12/2023 11:00	19	23.0	18.4	-4.6
2A	2/12/2023 12:00	19	23.0	16.8	-6.2
2A	2/12/2023 13:00	19	23.0	18.4	-4.6
2A	2/12/2023 14:00	19	23.0	18.4	-4.6
2A	2/12/2023 15:00	19	23.0	18.4	-4.6
2A	2/12/2023 16:00	19	23.0	13.9	-9.1
2A	2/12/2023 17:00	21	23.0	13.9	-9.1
2A	2/12/2023 18:00	21	23.0	13.9	-9.1

2A	2/12/2023 19:00	21	23.0	13.9	-9.1
2A	2/12/2023 20:00	21	23.0	16.7	-6.3
2A	2/12/2023 21:00	21	23.0	19.5	-3.5
2A	2/12/2023 22:00	21	23.0	19.5	-3.5
2A	2/12/2023 23:00	21	23.0	19.5	-3.5
2A	2/13/2023 0:00	21	23.0	20.1	-2.9
2A	2/13/2023 1:00	21	23.0	20.1	-2.9
2A	2/13/2023 2:00	21	23.0	20.1	-2.9
2A	2/13/2023 3:00	21	23.0	20.1	-2.9
2A	2/13/2023 4:00	21	23.0	20.1	-2.9
2A	2/13/2023 5:00	21	23.0	20.1	-2.9
2A	2/13/2023 6:00	21	23.0	20.1	-2.9
2A	2/13/2023 7:00	21	23.0	20.1	-2.9
2A	2/13/2023 8:00	21	23.0	23.6	0.6
2A	2/13/2023 9:00	21	23.0	23.6	0.6
2A	2/13/2023 10:00	21	23.0	23.6	0.6
2A	2/13/2023 11:00	21	23.0	23.6	0.6
2A	2/13/2023 12:00	21	23.0	23.6	0.6
2A	2/13/2023 13:00	21	23.0	23.6	0.6
2A	2/13/2023 14:00	21	23.0	23.6	0.6
2A	2/13/2023 15:00	21	23.0	23.6	0.6
2A	2/13/2023 16:00	21	23.0	25.0	2.0
2A	2/13/2023 17:00	21	23.0	25.0	2.0
2A	2/13/2023 18:00	21	23.0	25.0	2.0
2A	2/13/2023 19:00	21	23.0	25.0	2.0
2A	2/13/2023 20:00	17	23.0	22.3	-0.7
2A	2/13/2023 21:00	17	23.0	22.3	-0.7
2A	2/13/2023 22:00	17	23.0	22.3	-0.7
2A	2/13/2023 23:00	17	23.0	22.3	-0.7
2A	2/14/2023 0:00	19	23.0	20.1	-2.9
2A	2/14/2023 1:00	19	23.0	20.1	-2.9
2A	2/14/2023 2:00	19	23.0	20.1	-2.9
2A	2/14/2023 3:00	19	23.0	20.1	-2.9
2A	2/14/2023 4:00	19	23.0	20.1	-2.9
2A	2/14/2023 5:00	19	23.0	20.1	-2.9
2A	2/14/2023 6:00	19	23.0	20.1	-2.9
2A	2/14/2023 7:00	19	23.0	20.1	-2.9
2A	2/14/2023 8:00	20	23.0	18.4	-4.6
2A	2/14/2023 9:00	20	23.0	18.4	-4.6
2A	2/14/2023 10:00	20	23.0	18.4	-4.6
2A	2/14/2023 11:00	20	23.0	18.4	-4.6
2A	2/14/2023 12:00	20	23.0	21.0	-2.0
2A	2/14/2023 13:00	20	23.0	21.0	-2.0
2A	2/14/2023 14:00	20	23.0	21.0	-2.0
2A	2/14/2023 15:00	20	23.0	21.0	-2.0
2A	2/14/2023 16:00	20	23.0	22.3	-0.7
2A	2/14/2023 17:00	20	23.0	22.3	-0.7

2A	2/14/2023 18:00	20	23.0	22.3	-0.7
2A	2/14/2023 19:00	20	23.0	22.3	-0.7
2A	2/14/2023 20:00	18	23.0	22.3	-0.7
2A	2/14/2023 21:00	18	23.0	22.3	-0.7
2A	2/14/2023 22:00	18	23.0	22.3	-0.7
2A	2/14/2023 23:00	18	23.0	22.3	-0.7
2A	2/15/2023 0:00	20	23.0	20.1	-2.9
2A	2/15/2023 1:00	20	23.0	20.1	-2.9
2A	2/15/2023 2:00	20	23.0	20.1	-2.9
2A	2/15/2023 3:00	20	23.0	20.1	-2.9
2A	2/15/2023 4:00	20	23.0	20.1	-2.9
2A	2/15/2023 5:00	20	23.0	20.1	-2.9
2A	2/15/2023 6:00	20	23.0	20.1	-2.9
2A	2/15/2023 7:00	20	23.0	23.0	0.0
2A	2/15/2023 8:00	20	23.0	21.0	-2.0
2A	2/15/2023 9:00	20	23.0	21.0	-2.0
2A	2/15/2023 10:00	20	23.0	21.0	-2.0
2A	2/15/2023 11:00	20	23.0	21.0	-2.0
2A	2/15/2023 12:00	20	23.0	23.6	0.6
2A	2/15/2023 13:00	20	23.0	23.6	0.6
2A	2/15/2023 14:00	20	23.0	23.6	0.6
2A	2/15/2023 15:00	20	23.0	23.6	0.6
2A	2/15/2023 16:00	20	23.0	22.3	-0.7
2A	2/15/2023 17:00	20	23.0	22.3	-0.7
2A	2/15/2023 18:00	20	23.0	22.3	-0.7
2A	2/15/2023 19:00	20	23.0	22.3	-0.7
2A	2/15/2023 20:00	20	23.0	19.5	-3.5
2A	2/15/2023 21:00	21	23.0	19.5	-3.5
2A	2/15/2023 22:00	21	23.0	19.5	-3.5
2A	2/15/2023 23:00	21	23.0	19.5	-3.5
2A	2/16/2023 0:00	21	23.0	20.1	-2.9
2A	2/16/2023 1:00	21	23.0	20.1	-2.9
2A	2/16/2023 2:00	21	23.0	20.1	-2.9
2A	2/16/2023 3:00	21	23.0	20.1	-2.9
2A	2/16/2023 4:00	21	23.0	20.1	-2.9
2A	2/16/2023 5:00	21	23.0	20.1	-2.9
2A	2/16/2023 6:00	21	23.0	20.1	-2.9
2A	2/16/2023 7:00	21	23.0	20.1	-2.9
2A	2/16/2023 8:00	21	23.0	21.0	-2.0
2A	2/16/2023 9:00	21	23.0	21.0	-2.0
2A	2/16/2023 10:00	19	23.0	17.8	-5.2
2A	2/16/2023 11:00	19	23.0	21.0	-2.0
2A	2/16/2023 12:00	19	23.0	21.0	-2.0
2A	2/16/2023 13:00	19	23.0	16.3	-6.7
2A	2/16/2023 14:00	19	23.0	19.4	-3.6
2A	2/16/2023 15:00	19	23.0	21.0	-2.0
2A	2/16/2023 16:00	19	23.0	22.3	-0.7

2A	2/16/2023 17:00	19	23.0	20.6	-2.4
2A	2/16/2023 18:00	19	23.0	20.6	-2.4
2A	2/16/2023 19:00	19	23.0	22.3	-0.7
2A	2/16/2023 20:00	19	23.0	19.5	-3.5
2A	2/16/2023 21:00	19	23.0	19.5	-3.5
2A	2/16/2023 22:00	19	23.0	19.5	-3.5
2A	2/16/2023 23:00	18	23.0	19.5	-3.5
2A	2/17/2023 0:00	18	23.0	17.2	-5.8
2A	2/17/2023 1:00	18	23.0	17.2	-5.8
2A	2/17/2023 2:00	17	23.0	17.2	-5.8
2A	2/17/2023 3:00	17	23.0	17.2	-5.8
2A	2/17/2023 4:00	17	23.0	17.2	-5.8
2A	2/17/2023 5:00	17	23.0	17.2	-5.8
2A	2/17/2023 6:00	17	23.0	17.2	-5.8
2A	2/17/2023 7:00	17	23.0	17.2	-5.8
2A	2/17/2023 8:00	17	23.0	18.4	-4.6
2A	2/17/2023 9:00	17	23.0	18.4	-4.6
2A	2/17/2023 10:00	17	23.0	18.4	-4.6
2A	2/17/2023 11:00	17	23.0	18.4	-4.6
2A	2/17/2023 12:00	15	23.0	18.4	-4.6
2A	2/17/2023 13:00	15	23.0	18.4	-4.6
2A	2/17/2023 14:00	15	23.0	18.4	-4.6
2A	2/17/2023 15:00	15	23.0	18.4	-4.6
2A	2/17/2023 16:00	13	23.0	19.5	-3.5
2A	2/17/2023 17:00	13	23.0	19.5	-3.5
2A	2/17/2023 18:00	13	23.0	19.5	-3.5
2A	2/17/2023 19:00	13	23.0	19.5	-3.5
2A	2/17/2023 20:00	14	23.0	16.7	-6.3
2A	2/17/2023 21:00	14	23.0	16.7	-6.3
2A	2/17/2023 22:00	14	23.0	16.7	-6.3
2A	2/17/2023 23:00	14	23.0	16.7	-6.3
2A	2/18/2023 0:00	14	23.0	14.4	-8.7
2A	2/18/2023 1:00	14	23.0	14.4	-8.7
2A	2/18/2023 2:00	14	23.0	14.4	-8.7
2A	2/18/2023 3:00	14	23.0	14.4	-8.7
2A	2/18/2023 4:00	14	23.0	14.4	-8.7
2A	2/18/2023 5:00	14	23.0	14.4	-8.7
2A	2/18/2023 6:00	14	23.0	14.4	-8.7
2A	2/18/2023 7:00	14	23.0	14.4	-8.7
2A	2/18/2023 8:00	15	23.0	21.0	-2.0
2A	2/18/2023 9:00	15	23.0	21.0	-2.0
2A	2/18/2023 10:00	15	23.0	21.0	-2.0
2A	2/18/2023 11:00	15	23.0	22.0	-1.0
2A	2/18/2023 12:00	15	23.0	23.1	0.1
2A	2/18/2023 13:00	15	23.0	23.1	0.1
2A	2/18/2023 14:00	15	23.0	23.1	0.1
2A	2/18/2023 15:00	15	23.0	23.1	0.1

2A	2/18/2023 16:00	15	23.0	22.3	-0.7
2A	2/18/2023 17:00	15	23.0	22.3	-0.7
2A	2/18/2023 18:00	15	23.0	22.3	-0.7
2A	2/18/2023 19:00	15	23.0	22.3	-0.7
2A	2/18/2023 20:00	15	23.0	22.3	-0.7
2A	2/18/2023 21:00	15	23.0	22.3	-0.7
2A	2/18/2023 22:00	15	23.0	22.3	-0.7
2A	2/18/2023 23:00	15	23.0	22.3	-0.7
2A	2/19/2023 0:00	15	23.0	23.0	0.0
2A	2/19/2023 1:00	15	23.0	23.0	0.0
2A	2/19/2023 2:00	15	23.0	23.0	0.0
2A	2/19/2023 3:00	15	23.0	23.0	0.0
2A	2/19/2023 4:00	15	23.0	23.0	0.0
2A	2/19/2023 5:00	15	23.0	23.0	0.0
2A	2/19/2023 6:00	15	23.0	23.0	0.0
2A	2/19/2023 7:00	15	23.0	23.0	0.0
2A	2/19/2023 8:00	15	23.0	21.0	-2.0
2A	2/19/2023 9:00	15	23.0	21.0	-2.0
2A	2/19/2023 10:00	15	23.0	21.0	-2.0
2A	2/19/2023 11:00	15	23.0	21.0	-2.0
2A	2/19/2023 12:00	15	23.0	23.1	0.1
2A	2/19/2023 13:00	15	23.0	23.1	0.1
2A	2/19/2023 14:00	15	23.0	23.1	0.1
2A	2/19/2023 15:00	14	23.0	23.1	0.1
2A	2/19/2023 16:00	14	23.0	22.3	-0.7
2A	2/19/2023 17:00	14	23.0	22.3	-0.7
2A	2/19/2023 18:00	14	23.0	22.3	-0.7
2A	2/19/2023 19:00	14	23.0	22.3	-0.7
2A	2/19/2023 20:00	14	23.0	16.7	-6.3
2A	2/19/2023 21:00	14	23.0	16.7	-6.3
2A	2/19/2023 22:00	14	23.0	16.7	-6.3
2A	2/19/2023 23:00	14	23.0	16.7	-6.3
2A	2/20/2023 0:00	14	23.0	17.2	-5.8
2A	2/20/2023 1:00	14	23.0	17.2	-5.8
2A	2/20/2023 2:00	14	23.0	17.2	-5.8
2A	2/20/2023 3:00	14	23.0	17.2	-5.8
2A	2/20/2023 4:00	14	23.0	17.2	-5.8
2A	2/20/2023 5:00	14	23.0	17.2	-5.8
2A	2/20/2023 6:00	14	23.0	17.2	-5.8
2A	2/20/2023 7:00	14	23.0	17.2	-5.8
2A	2/20/2023 8:00	14	23.0	18.4	-4.6
2A	2/20/2023 9:00	14	23.0	18.4	-4.6
2A	2/20/2023 10:00	14	23.0	18.4	-4.6
2A	2/20/2023 11:00	14	23.0	18.4	-4.6
2A	2/20/2023 12:00	14	23.0	18.4	-4.6
2A	2/20/2023 13:00	14	23.0	18.4	-4.6
2A	2/20/2023 14:00	14	23.0	18.4	-4.6

2A	2/20/2023 15:00	14	23.0	18.4	-4.6
2A	2/20/2023 16:00	14	23.0	19.5	-3.5
2A	2/20/2023 17:00	14	23.0	19.5	-3.5
2A	2/20/2023 18:00	14	23.0	19.5	-3.5
2A	2/20/2023 19:00	14	23.0	19.5	-3.5
2A	2/20/2023 20:00	12	23.0	19.5	-3.5
2A	2/20/2023 21:00	12	23.0	19.5	-3.5
2A	2/20/2023 22:00	12	23.0	19.5	-3.5
2A	2/20/2023 23:00	12	23.0	19.5	-3.5
2A	2/21/2023 0:00	12	23.0	20.1	-2.9
2A	2/21/2023 1:00	12	23.0	20.1	-2.9
2A	2/21/2023 2:00	12	23.0	20.1	-2.9
2A	2/21/2023 3:00	12	23.0	20.1	-2.9
2A	2/21/2023 4:00	13	23.0	20.1	-2.9
2A	2/21/2023 5:00	13	23.0	20.1	-2.9
2A	2/21/2023 6:00	13	23.0	20.1	-2.9
2A	2/21/2023 7:00	13	23.0	20.1	-2.9
2A	2/21/2023 8:00	13	23.0	18.4	-4.6
2A	2/21/2023 9:00	13	23.0	18.4	-4.6
2A	2/21/2023 10:00	13	23.0	18.4	-4.6
2A	2/21/2023 11:00	13	23.0	18.4	-4.6
2A	2/21/2023 12:00	13	23.0	18.4	-4.6
2A	2/21/2023 13:00	13	23.0	18.4	-4.6
2A	2/21/2023 14:00	13	23.0	18.4	-4.6
2A	2/21/2023 15:00	13	23.0	18.4	-4.6
2A	2/21/2023 16:00	13	23.0	16.7	-6.3
2A	2/21/2023 17:00	11	23.0	16.7	-6.3
2A	2/21/2023 18:00	11	23.0	16.7	-6.3
2A	2/21/2023 19:00	11	23.0	16.7	-6.3
2A	2/21/2023 20:00	12	23.0	16.7	-6.3
2A	2/21/2023 21:00	12	23.0	16.7	-6.3
2A	2/21/2023 22:00	13	23.0	16.7	-6.3
2A	2/21/2023 23:00	13	23.0	16.7	-6.3
2A	2/22/2023 0:00	13	23.0	14.4	-8.7
2A	2/22/2023 1:00	13	23.0	14.4	-8.7
2A	2/22/2023 2:00	13	23.0	14.4	-8.7
2A	2/22/2023 3:00	13	23.0	14.4	-8.7
2A	2/22/2023 4:00	14	23.0	14.4	-8.7
2A	2/22/2023 5:00	14	23.0	14.4	-8.7
2A	2/22/2023 6:00	14	23.0	14.4	-8.7
2A	2/22/2023 7:00	14	23.0	14.4	-8.7
2A	2/22/2023 8:00	14	23.0	18.4	-4.6
2A	2/22/2023 9:00	14	23.0	18.4	-4.6
2A	2/22/2023 10:00	14	23.0	18.4	-4.6
2A	2/22/2023 11:00	14	23.0	18.4	-4.6
2A	2/22/2023 12:00	14	23.0	21.0	-2.0
2A	2/22/2023 13:00	14	23.0	21.0	-2.0

2A	2/22/2023 14:00	14	23.0	21.0	-2.0
2A	2/22/2023 15:00	14	23.0	21.0	-2.0
2A	2/22/2023 16:00	14	23.0	19.5	-3.5
2A	2/22/2023 17:00	14	23.0	19.5	-3.5
2A	2/22/2023 18:00	14	23.0	19.5	-3.5
2A	2/22/2023 19:00	14	23.0	19.5	-3.5
2A	2/22/2023 20:00	20	23.0	16.7	-6.3
2A	2/22/2023 21:00	21	23.0	16.7	-6.3
2A	2/22/2023 22:00	21	23.0	16.7	-6.3
2A	2/22/2023 23:00	21	23.0	16.7	-6.3
2A	2/23/2023 0:00	21	23.0	14.4	-8.7
2A	2/23/2023 1:00	21	23.0	14.4	-8.7
2A	2/23/2023 2:00	21	23.0	14.4	-8.7
2A	2/23/2023 3:00	21	23.0	14.4	-8.7
2A	2/23/2023 4:00	21	23.0	14.4	-8.7
2A	2/23/2023 5:00	21	23.0	14.4	-8.7
2A	2/23/2023 6:00	21	23.0	14.4	-8.7
2A	2/23/2023 7:00	21	22.0	14.4	-7.7
2A	2/23/2023 8:00	23	22.0	15.7	-6.3
2A	2/23/2023 9:00	23	22.0	15.7	-6.3
2A	2/23/2023 10:00	23	22.0	15.7	-6.3
2A	2/23/2023 11:00	23	22.0	15.7	-6.3
2A	2/23/2023 12:00	23	22.0	17.1	-5.0
2A	2/23/2023 13:00	23	22.0	18.4	-3.6
2A	2/23/2023 14:00	23	22.0	18.4	-3.6
2A	2/23/2023 15:00	23	22.0	18.4	-3.6
2A	2/23/2023 16:00	23	22.0	16.7	-5.3
2A	2/23/2023 17:00	23	22.0	16.7	-5.3
2A	2/23/2023 18:00	23	22.0	16.7	-5.3
2A	2/23/2023 19:00	23	22.0	16.7	-5.3
2A	2/23/2023 20:00	17	23.0	13.9	-9.1
2A	2/23/2023 21:00	17	23.0	13.9	-9.1
2A	2/23/2023 22:00	17	23.0	13.9	-9.1
2A	2/23/2023 23:00	17	23.0	13.9	-9.1
2A	2/24/2023 0:00	17	23.0	14.4	-8.7
2A	2/24/2023 1:00	17	23.0	14.4	-8.7
2A	2/24/2023 2:00	20	23.0	14.4	-8.7
2A	2/24/2023 3:00	20	23.0	14.4	-8.7
2A	2/24/2023 4:00	20	23.0	14.4	-8.7
2A	2/24/2023 5:00	20	23.0	14.4	-8.7
2A	2/24/2023 6:00	21	23.0	14.4	-8.7
2A	2/24/2023 7:00	21	23.0	14.4	-8.7
2A	2/24/2023 8:00	21	23.0	14.7	-8.3
2A	2/24/2023 9:00	21	23.0	14.7	-8.3
2A	2/24/2023 10:00	21	23.0	14.7	-8.3
2A	2/24/2023 11:00	21	23.0	14.7	-8.3
2A	2/24/2023 12:00	21	23.0	16.8	-6.2

2A	2/24/2023 13:00	21	23.0	16.8	-6.2
2A	2/24/2023 14:00	21	23.0	16.8	-6.2
2A	2/24/2023 15:00	21	23.0	16.8	-6.2
2A	2/24/2023 16:00	21	23.0	16.7	-6.3
2A	2/24/2023 17:00	21	23.0	16.7	-6.3
2A	2/24/2023 18:00	21	23.0	16.7	-6.3
2A	2/24/2023 19:00	21	23.0	16.7	-6.3
2A	2/24/2023 20:00	17	23.0	22.3	-0.7
2A	2/24/2023 21:00	17	23.0	22.3	-0.7
2A	2/24/2023 22:00	17	23.0	22.3	-0.7
2A	2/24/2023 23:00	17	23.0	22.3	-0.7
2A	2/25/2023 0:00	17	23.0	20.1	-2.9
2A	2/25/2023 1:00	19	23.0	20.1	-2.9
2A	2/25/2023 2:00	19	23.0	20.1	-2.9
2A	2/25/2023 3:00	19	23.0	20.1	-2.9
2A	2/25/2023 4:00	19	23.0	20.1	-2.9
2A	2/25/2023 5:00	19	23.0	20.1	-2.9
2A	2/25/2023 6:00	19	23.0	20.1	-2.9
2A	2/25/2023 7:00	19	23.0	20.1	-2.9
2A	2/25/2023 8:00	19	23.0	15.7	-7.3
2A	2/25/2023 9:00	19	23.0	15.7	-7.3
2A	2/25/2023 10:00	19	23.0	15.7	-7.3
2A	2/25/2023 11:00	19	23.0	15.7	-7.3
2A	2/25/2023 12:00	19	23.0	18.4	-4.6
2A	2/25/2023 13:00	19	23.0	18.4	-4.6
2A	2/25/2023 14:00	19	23.0	18.4	-4.6
2A	2/25/2023 15:00	19	23.0	18.4	-4.6
2A	2/25/2023 16:00	17	23.0	16.7	-6.3
2A	2/25/2023 17:00	17	23.0	16.7	-6.3
2A	2/25/2023 18:00	18	23.0	16.7	-6.3
2A	2/25/2023 19:00	18	23.0	16.7	-6.3
2A	2/25/2023 20:00	18	23.0	19.5	-3.5
2A	2/25/2023 21:00	18	23.0	19.5	-3.5
2A	2/25/2023 22:00	18	23.0	19.5	-3.5
2A	2/25/2023 23:00	18	23.0	19.5	-3.5
2A	2/26/2023 0:00	18	23.0	20.1	-2.9
2A	2/26/2023 1:00	18	23.0	20.1	-2.9
2A	2/26/2023 2:00	18	23.0	20.1	-2.9
2A	2/26/2023 3:00	18	23.0	20.1	-2.9
2A	2/26/2023 4:00	18	23.0	20.1	-2.9
2A	2/26/2023 5:00	18	23.0	20.1	-2.9
2A	2/26/2023 6:00	18	23.0	20.1	-2.9
2A	2/26/2023 7:00	18	23.0	20.1	-2.9
2A	2/26/2023 8:00	19	23.0	18.4	-4.6
2A	2/26/2023 9:00	19	23.0	18.4	-4.6
2A	2/26/2023 10:00	19	23.0	18.4	-4.6
2A	2/26/2023 11:00	19	23.0	17.8	-5.2

2A	2/26/2023 12:00	19	23.0	21.0	-2.0
2A	2/26/2023 13:00	19	23.0	21.0	-2.0
2A	2/26/2023 14:00	19	23.0	21.0	-2.0
2A	2/26/2023 15:00	19	23.0	21.0	-2.0
2A	2/26/2023 16:00	19	23.0	19.5	-3.5
2A	2/26/2023 17:00	20	20.0	19.5	-0.5
2A	2/26/2023 18:00	20	20.0	19.5	-0.5
2A	2/26/2023 19:00	20	20.0	19.5	-0.5
2A	2/26/2023 20:00	19	20.0	16.7	-3.3
2A	2/26/2023 21:00	19	20.0	16.7	-3.3
2A	2/26/2023 22:00	19	20.0	16.7	-3.3
2A	2/26/2023 23:00	19	20.0	16.7	-3.3
2A	2/27/2023 0:00	19	20.0	15.8	-4.2
2A	2/27/2023 1:00	19	20.0	17.2	-2.8
2A	2/27/2023 2:00	19	20.0	17.2	-2.8
2A	2/27/2023 3:00	20	20.0	17.2	-2.8
2A	2/27/2023 4:00	20	20.0	17.2	-2.8
2A	2/27/2023 5:00	20	20.0	17.2	-2.8
2A	2/27/2023 6:00	19	20.0	17.2	-2.8
2A	2/27/2023 7:00	19	20.0	17.2	-2.8
2A	2/27/2023 8:00	19	20.0	15.7	-4.3
2A	2/27/2023 9:00	19	20.0	15.7	-4.3
2A	2/27/2023 10:00	19	20.0	15.7	-4.3
2A	2/27/2023 11:00	19	20.0	15.7	-4.3
2A	2/27/2023 12:00	19	20.0	21.0	1.0
2A	2/27/2023 13:00	19	20.0	21.0	1.0
2A	2/27/2023 14:00	19	20.0	21.0	1.0
2A	2/27/2023 15:00	19	20.0	21.0	1.0
2A	2/27/2023 16:00	19	20.0	19.5	-0.5
2A	2/27/2023 17:00	19	20.0	19.5	-0.5
2A	2/27/2023 18:00	19	20.0	19.5	-0.5
2A	2/27/2023 19:00	19	20.0	19.5	-0.5
2A	2/27/2023 20:00	19	20.0	24.5	4.5
2A	2/27/2023 21:00	19	20.0	24.5	4.5
2A	2/27/2023 22:00	19	20.0	24.5	4.5
2A	2/27/2023 23:00	19	20.0	24.5	4.5
2A	2/28/2023 0:00	22	23.0	20.1	-2.9
2A	2/28/2023 1:00	22	23.0	20.1	-2.9
2A	2/28/2023 2:00	22	23.0	20.1	-2.9
2A	2/28/2023 3:00	22	23.0	20.1	-2.9
2A	2/28/2023 4:00	22	23.0	20.1	-2.9
2A	2/28/2023 5:00	22	23.0	20.1	-2.9
2A	2/28/2023 6:00	22	23.0	20.1	-2.9
2A	2/28/2023 7:00	22	23.0	20.1	-2.9
2A	2/28/2023 8:00	22	23.0	18.4	-4.6
2A	2/28/2023 9:00	22	23.0	18.4	-4.6
2A	2/28/2023 10:00	22	23.0	18.4	-4.6

2A	2/28/2023 11:00	22	23.0	18.4	-4.6
2A	2/28/2023 12:00	22	23.0	21.0	-2.0
2A	2/28/2023 13:00	22	23.0	21.0	-2.0
2A	2/28/2023 14:00	22	23.0	21.0	-2.0
2A	2/28/2023 15:00	22	23.0	21.0	-2.0
2A	2/28/2023 16:00	22	23.0	19.5	-3.5
2A	2/28/2023 17:00	22	23.0	19.5	-3.5
2A	2/28/2023 18:00	22	23.0	19.5	-3.5
2A	2/28/2023 19:00	22	23.0	19.5	-3.5
2A	2/28/2023 20:00	22	23.0	22.3	-0.7
2A	2/28/2023 21:00	18	20.0	16.7	-3.3
2A	2/28/2023 22:00	18	20.0	16.7	-3.3
2A	2/28/2023 23:00	18	20.0	16.7	-3.3
2A	3/1/2023 0:00	18	20.0	14.4	-5.7
2A	3/1/2023 1:00	18	20.0	14.4	-5.7
2A	3/1/2023 2:00	18	20.0	14.4	-5.7
2A	3/1/2023 3:00	18	20.0	14.4	-5.7
2A	3/1/2023 4:00	18	20.0	14.4	-5.7
2A	3/1/2023 5:00	18	20.0	14.4	-5.7
2A	3/1/2023 6:00	18	20.0	14.4	-5.7
2A	3/1/2023 7:00	18	20.0	14.4	-5.7
2A	3/1/2023 8:00	18	20.0	18.4	-1.6
2A	3/1/2023 9:00	18	20.0	18.4	-1.6
2A	3/1/2023 10:00	18	20.0	18.4	-1.6
2A	3/1/2023 11:00	18	20.0	18.4	-1.6
2A	3/1/2023 12:00	18	20.0	21.0	1.0
2A	3/1/2023 13:00	18	20.0	21.0	1.0
2A	3/1/2023 14:00	18	20.0	21.0	1.0
2A	3/1/2023 15:00	18	20.0	21.0	1.0
2A	3/1/2023 16:00	18	20.0	19.5	-0.5
2A	3/1/2023 17:00	18	20.0	19.5	-0.5
2A	3/1/2023 18:00	18	20.0	19.5	-0.5
2A	3/1/2023 19:00	18	20.0	19.5	-0.5
2A	3/1/2023 20:00	18	20.0	19.5	-0.5
2A	3/1/2023 21:00	18	20.0	19.5	-0.5
2A	3/1/2023 22:00	18	20.0	19.5	-0.5
2A	3/1/2023 23:00	18	20.0	19.5	-0.5
2A	3/2/2023 0:00	18	20.0	20.1	0.1
2A	3/2/2023 1:00	21	22.0	20.1	-1.9
2A	3/2/2023 2:00	21	22.0	20.1	-1.9
2A	3/2/2023 3:00	21	22.0	20.1	-1.9
2A	3/2/2023 4:00	21	22.0	20.1	-1.9
2A	3/2/2023 5:00	21	22.0	20.1	-1.9
2A	3/2/2023 6:00	21	22.0	20.1	-1.9
2A	3/2/2023 7:00	21	22.0	20.1	-1.9
2A	3/2/2023 8:00	20	23.0	15.7	-7.3
2A	3/2/2023 9:00	23	23.0	15.7	-7.3

2A	3/2/2023 10:00	23	23.0	16.8	-6.2
2A	3/2/2023 11:00	23	23.0	18.4	-4.6
2A	3/2/2023 12:00	23	23.0	18.4	-4.6
2A	3/2/2023 13:00	23	23.0	18.4	-4.6
2A	3/2/2023 14:00	23	23.0	15.2	-7.8
2A	3/2/2023 15:00	23	23.0	18.4	-4.6
2A	3/2/2023 16:00	23	23.0	19.5	-3.5
2A	3/2/2023 17:00	23	23.0	19.5	-3.5
2A	3/2/2023 18:00	23	23.0	19.5	-3.5
2A	3/2/2023 19:00	23	23.0	19.5	-3.5
2A	3/2/2023 20:00	23	23.0	22.3	-0.7
2A	3/2/2023 21:00	23	23.0	22.3	-0.7
2A	3/2/2023 22:00	23	23.0	22.3	-0.7
2A	3/2/2023 23:00	23	23.0	22.3	-0.7
2A	3/3/2023 0:00	23	21.0	20.1	-0.9
2A	3/3/2023 1:00	23	21.0	20.1	-0.9
2A	3/3/2023 2:00	23	21.0	20.1	-0.9
2A	3/3/2023 3:00	23	21.0	20.1	-0.9
2A	3/3/2023 4:00	23	21.0	20.1	-0.9
2A	3/3/2023 5:00	23	21.0	20.1	-0.9
2A	3/3/2023 6:00	23	21.0	20.1	-0.9
2A	3/3/2023 7:00	23	21.0	20.1	-0.9
2A	3/3/2023 8:00	22	23.0	18.4	-4.6
2A	3/3/2023 9:00	22	23.0	16.8	-6.2
2A	3/3/2023 10:00	22	23.0	18.4	-4.6
2A	3/3/2023 11:00	22	23.0	18.4	-4.6
2A	3/3/2023 12:00	22	23.0	18.4	-4.6
2A	3/3/2023 13:00	22	23.0	15.2	-7.8
2A	3/3/2023 14:00	22	23.0	16.8	-6.2
2A	3/3/2023 15:00	22	23.0	18.4	-4.6
2A	3/3/2023 16:00	22	23.0	19.5	-3.5
2A	3/3/2023 17:00	22	23.0	19.5	-3.5
2A	3/3/2023 18:00	22	23.0	19.5	-3.5
2A	3/3/2023 19:00	22	23.0	19.5	-3.5
2A	3/3/2023 20:00	22	23.0	19.5	-3.5
2A	3/3/2023 21:00	22	23.0	19.5	-3.5
2A	3/3/2023 22:00	22	23.0	19.5	-3.5
2A	3/3/2023 23:00	22	23.0	19.5	-3.5
2A	3/4/2023 0:00	23	23.0	14.4	-8.7
2A	3/4/2023 1:00	23	23.0	14.4	-8.7
2A	3/4/2023 2:00	23	23.0	14.4	-8.7
2A	3/4/2023 3:00	23	23.0	14.4	-8.7
2A	3/4/2023 4:00	23	23.0	14.4	-8.7
2A	3/4/2023 5:00	23	23.0	14.4	-8.7
2A	3/4/2023 6:00	23	23.0	14.4	-8.7
2A	3/4/2023 7:00	23	23.0	14.4	-8.7
2A	3/4/2023 8:00	23	23.0	15.7	-7.3

2A	3/4/2023 9:00	23	23.0	15.7	-7.3
2A	3/4/2023 10:00	23	23.0	13.1	-9.9
2A	3/4/2023 11:00	23	23.0	14.7	-8.3
2A	3/4/2023 12:00	23	23.0	16.8	-6.2
2A	3/4/2023 13:00	23	23.0	16.8	-6.2
2A	3/4/2023 14:00	23	23.0	16.8	-6.2
2A	3/4/2023 15:00	23	23.0	16.8	-6.2
2A	3/4/2023 16:00	23	23.0	16.7	-6.3
2A	3/4/2023 17:00	23	23.0	16.7	-6.3
2A	3/4/2023 18:00	23	23.0	16.7	-6.3
2A	3/4/2023 19:00	23	23.0	16.7	-6.3
2A	3/4/2023 20:00	23	23.0	22.3	-0.7
2A	3/4/2023 21:00	23	23.0	22.3	-0.7
2A	3/4/2023 22:00	23	23.0	22.3	-0.7
2A	3/4/2023 23:00	23	23.0	22.3	-0.7
2A	3/5/2023 0:00	23	23.0	20.1	-2.9
2A	3/5/2023 1:00	23	23.0	20.1	-2.9
2A	3/5/2023 2:00	23	23.0	20.1	-2.9
2A	3/5/2023 3:00	23	23.0	20.1	-2.9
2A	3/5/2023 4:00	23	23.0	20.1	-2.9
2A	3/5/2023 5:00	23	23.0	20.1	-2.9
2A	3/5/2023 6:00	23	23.0	20.1	-2.9
2A	3/5/2023 7:00	23	23.0	20.1	-2.9
2A	3/5/2023 8:00	23	23.0	18.9	-4.1
2A	3/5/2023 9:00	23	23.0	18.9	-4.1
2A	3/5/2023 10:00	23	23.0	18.9	-4.1
2A	3/5/2023 11:00	23	23.0	18.9	-4.1
2A	3/5/2023 12:00	23	23.0	21.0	-2.0
2A	3/5/2023 13:00	23	23.0	21.0	-2.0
2A	3/5/2023 14:00	23	23.0	21.0	-2.0
2A	3/5/2023 15:00	23	23.0	21.0	-2.0
2A	3/5/2023 16:00	23	23.0	16.7	-6.3
2A	3/5/2023 17:00	23	23.0	16.7	-6.3
2A	3/5/2023 18:00	23	23.0	16.7	-6.3
2A	3/5/2023 19:00	23	23.0	16.7	-6.3
2A	3/5/2023 20:00	23	23.0	19.5	-3.5
2A	3/5/2023 21:00	23	23.0	19.5	-3.5
2A	3/5/2023 22:00	23	23.0	19.5	-3.5
2A	3/5/2023 23:00	23	23.0	19.5	-3.5
2A	3/6/2023 0:00	23	23.0	20.1	-2.9
2A	3/6/2023 1:00	23	23.0	20.1	-2.9
2A	3/6/2023 2:00	23	23.0	20.1	-2.9
2A	3/6/2023 3:00	23	23.0	20.1	-2.9
2A	3/6/2023 4:00	23	23.0	20.1	-2.9
2A	3/6/2023 5:00	23	23.0	20.1	-2.9
2A	3/6/2023 6:00	23	23.0	20.1	-2.9
2A	3/6/2023 7:00	23	23.0	20.1	-2.9

2A	3/6/2023 8:00	23	22.0	18.4	-3.6
2A	3/6/2023 9:00	23	23.0	18.4	-4.6
2A	3/6/2023 10:00	23	23.0	18.4	-4.6
2A	3/6/2023 11:00	23	23.0	18.4	-4.6
2A	3/6/2023 12:00	23	23.0	21.0	-2.0
2A	3/6/2023 13:00	23	23.0	21.0	-2.0
2A	3/6/2023 14:00	23	23.0	21.0	-2.0
2A	3/6/2023 15:00	23	23.0	21.0	-2.0
2A	3/6/2023 16:00	23	23.0	22.3	-0.7
2A	3/6/2023 17:00	23	23.0	22.3	-0.7
2A	3/6/2023 18:00	23	23.0	22.3	-0.7
2A	3/6/2023 19:00	23	23.0	22.3	-0.7
2A	3/6/2023 20:00	23	23.0	16.7	-6.3
2A	3/6/2023 21:00	23	23.0	16.7	-6.3
2A	3/6/2023 22:00	23	23.0	16.7	-6.3
2A	3/6/2023 23:00	23	23.0	16.7	-6.3
2A	3/7/2023 0:00	23	23.0	14.4	-8.7
2A	3/7/2023 1:00	23	23.0	14.4	-8.7
2A	3/7/2023 2:00	23	23.0	14.4	-8.7
2A	3/7/2023 3:00	23	23.0	14.4	-8.7
2A	3/7/2023 4:00	23	23.0	14.4	-8.7
2A	3/7/2023 5:00	23	23.0	14.4	-8.7
2A	3/7/2023 6:00	23	23.0	14.4	-8.7
2A	3/7/2023 7:00	23	23.0	14.4	-8.7
2A	3/7/2023 8:00	23	23.0	15.7	-7.3
2A	3/7/2023 9:00	23	23.0	15.7	-7.3
2A	3/7/2023 10:00	22	23.0	15.7	-7.3
2A	3/7/2023 11:00	22	23.0	15.7	-7.3
2A	3/7/2023 12:00	22	23.0	18.4	-4.6
2A	3/7/2023 13:00	22	23.0	18.4	-4.6
2A	3/7/2023 14:00	22	23.0	18.4	-4.6
2A	3/7/2023 15:00	22	23.0	18.4	-4.6
2A	3/7/2023 16:00	22	23.0	19.5	-3.5
2A	3/7/2023 17:00	22	23.0	18.1	-4.9
2A	3/7/2023 18:00	22	23.0	16.7	-6.3
2A	3/7/2023 19:00	22	23.0	16.7	-6.3
2A	3/7/2023 20:00	22	23.0	19.5	-3.5
2A	3/7/2023 21:00	22	23.0	19.5	-3.5
2A	3/7/2023 22:00	22	23.0	19.5	-3.5
2A	3/7/2023 23:00	22	23.0	19.5	-3.5
2A	3/8/2023 0:00	23	23.0	17.2	-5.8
2A	3/8/2023 1:00	23	23.0	17.2	-5.8
2A	3/8/2023 2:00	23	23.0	17.2	-5.8
2A	3/8/2023 3:00	23	23.0	17.2	-5.8
2A	3/8/2023 4:00	23	23.0	17.2	-5.8
2A	3/8/2023 5:00	22	23.0	17.2	-5.8
2A	3/8/2023 6:00	22	23.0	17.2	-5.8

2A	3/8/2023 7:00	22	23.0	17.2	-5.8
2A	3/8/2023 8:00	22	23.0	13.1	-9.9
2A	3/8/2023 9:00	22	23.0	13.1	-9.9
2A	3/8/2023 10:00	22	23.0	13.1	-9.9
2A	3/8/2023 11:00	22	23.0	13.1	-9.9
2A	3/8/2023 12:00	22	23.0	17.1	-6.0
2A	3/8/2023 13:00	22	23.0	18.4	-4.6
2A	3/8/2023 14:00	22	23.0	18.4	-4.6
2A	3/8/2023 15:00	22	23.0	18.4	-4.6
2A	3/8/2023 16:00	22	23.0	19.5	-3.5
2A	3/8/2023 17:00	22	23.0	19.5	-3.5
2A	3/8/2023 18:00	22	23.0	19.5	-3.5
2A	3/8/2023 19:00	22	23.0	19.5	-3.5
2A	3/8/2023 20:00	22	23.0	16.7	-6.3
2A	3/8/2023 21:00	22	23.0	16.7	-6.3
2A	3/8/2023 22:00	22	23.0	16.7	-6.3
2A	3/8/2023 23:00	22	23.0	15.0	-8.0
2A	3/9/2023 0:00	22	23.0	17.2	-5.8
2A	3/9/2023 1:00	22	23.0	17.2	-5.8
2A	3/9/2023 2:00	22	23.0	17.2	-5.8
2A	3/9/2023 3:00	22	23.0	17.2	-5.8
2A	3/9/2023 4:00	22	23.0	17.2	-5.8
2A	3/9/2023 5:00	22	23.0	17.2	-5.8
2A	3/9/2023 6:00	22	23.0	17.2	-5.8
2A	3/9/2023 7:00	23	25.0	17.2	-7.8
2A	3/9/2023 8:00	23	25.0	15.7	-9.3
2A	3/9/2023 9:00	23	25.0	15.7	-9.3
2A	3/9/2023 10:00	23	25.0	15.7	-9.3
2A	3/9/2023 11:00	23	25.0	15.7	-9.3
2A	3/9/2023 12:00	23	25.0	15.7	-9.3
2A	3/9/2023 13:00	23	25.0	15.7	-9.3
2A	3/9/2023 14:00	23	25.0	15.7	-9.3
2A	3/9/2023 15:00	23	25.0	15.7	-9.3
2A	3/9/2023 16:00	23	25.0	16.7	-8.3
2A	3/9/2023 17:00	23	25.0	16.7	-8.3
2A	3/9/2023 18:00	23	25.0	16.7	-8.3
2A	3/9/2023 19:00	23	25.0	18.1	-6.9
2A	3/9/2023 20:00	23	25.0	19.5	-5.5
2A	3/9/2023 21:00	23	25.0	19.5	-5.5
2A	3/9/2023 22:00	23	25.0	19.5	-5.5
2A	3/9/2023 23:00	23	25.0	19.5	-5.5
2A	3/10/2023 0:00	24	25.0	17.2	-7.8
2A	3/10/2023 1:00	25	25.0	17.9	-7.1
2A	3/10/2023 2:00	25	25.0	20.1	-4.9
2A	3/10/2023 3:00	25	25.0	20.1	-4.9
2A	3/10/2023 4:00	25	25.0	20.1	-4.9
2A	3/10/2023 5:00	25	25.0	20.1	-4.9

2A	3/10/2023 6:00	25	25.0	20.1	-4.9
2A	3/10/2023 7:00	25	25.0	20.1	-4.9
2A	3/10/2023 8:00	25	25.0	15.7	-9.3
2A	3/10/2023 9:00	25	25.0	15.7	-9.3
2A	3/10/2023 10:00	25	25.0	15.7	-9.3
2A	3/10/2023 11:00	25	25.0	15.7	-9.3
2A	3/10/2023 12:00	25	25.0	18.4	-6.6
2A	3/10/2023 13:00	25	25.0	18.4	-6.6
2A	3/10/2023 14:00	25	25.0	18.4	-6.6
2A	3/10/2023 15:00	25	25.0	18.4	-6.6
2A	3/10/2023 16:00	25	25.0	16.7	-8.3
2A	3/10/2023 17:00	25	25.0	16.7	-8.3
2A	3/10/2023 18:00	25	25.0	16.7	-8.3
2A	3/10/2023 19:00	25	25.0	16.7	-8.3
2A	3/10/2023 20:00	25	25.0	16.7	-8.3
2A	3/10/2023 21:00	25	25.0	16.7	-8.3
2A	3/10/2023 22:00	25	25.0	16.7	-8.3
2A	3/10/2023 23:00	25	25.0	16.7	-8.3
2A	3/11/2023 0:00	25	25.0	17.2	-7.8
2A	3/11/2023 1:00	25	25.0	17.2	-7.8
2A	3/11/2023 2:00	25	25.0	17.2	-7.8
2A	3/11/2023 3:00	25	25.0	17.2	-7.8
2A	3/11/2023 4:00	25	25.0	17.2	-7.8
2A	3/11/2023 5:00	25	25.0	17.2	-7.8
2A	3/11/2023 6:00	23	25.0	17.2	-7.8
2A	3/11/2023 7:00	23	25.0	19.4	-5.6
2A	3/11/2023 8:00	23	25.0	15.7	-9.3
2A	3/11/2023 9:00	23	25.0	15.7	-9.3
2A	3/11/2023 10:00	23	25.0	15.7	-9.3
2A	3/11/2023 11:00	23	25.0	15.7	-9.3
2A	3/11/2023 12:00	23	25.0	15.7	-9.3
2A	3/11/2023 13:00	23	25.0	15.7	-9.3
2A	3/11/2023 14:00	23	25.0	15.7	-9.3
2A	3/11/2023 15:00	23	25.0	15.7	-9.3
2A	3/11/2023 16:00	19	23.0	13.9	-9.1
2A	3/11/2023 17:00	19	23.0	13.9	-9.1
2A	3/11/2023 18:00	19	23.0	13.9	-9.1
2A	3/11/2023 19:00	19	23.0	13.9	-9.1
2A	3/11/2023 20:00	19	23.0	19.5	-3.5
2A	3/11/2023 21:00	19	23.0	19.5	-3.5
2A	3/11/2023 22:00	16	20.0	19.5	-0.5
2A	3/11/2023 23:00	16	20.0	19.5	-0.5
2A	3/12/2023 0:00	16	20.0	18.4	-1.6
2A	3/12/2023 1:00	16	20.0	18.4	-1.6
2A	3/12/2023 2:00	16	20.0	18.4	-1.6
2A	3/12/2023 3:00	16	20.0	18.4	-1.6
2A	3/12/2023 4:00	16	20.0	18.4	-1.6

2A	3/12/2023 5:00	16	20.0	18.4	-1.6
2A	3/12/2023 6:00	16	20.0	18.4	-1.6
2A	3/12/2023 7:00	18	20.0	16.1	-3.9
2A	3/12/2023 8:00	18	20.0	18.4	-1.6
2A	3/12/2023 9:00	18	20.0	18.4	-1.6
2A	3/12/2023 10:00	18	20.0	18.4	-1.6
2A	3/12/2023 11:00	18	20.0	18.4	-1.6
2A	3/12/2023 12:00	18	20.0	18.4	-1.6
2A	3/12/2023 13:00	18	20.0	18.4	-1.6
2A	3/12/2023 14:00	18	20.0	18.4	-1.6
2A	3/12/2023 15:00	18	20.0	18.4	-1.6
2A	3/12/2023 16:00	18	20.0	13.9	-6.1
2A	3/12/2023 17:00	18	20.0	13.9	-6.1
2A	3/12/2023 18:00	18	20.0	13.9	-6.1
2A	3/12/2023 19:00	18	20.0	13.9	-6.1
2A	3/12/2023 20:00	18	19.0	16.7	-2.3
2A	3/12/2023 21:00	18	19.0	16.7	-2.3
2A	3/12/2023 22:00	18	19.0	16.7	-2.3
2A	3/12/2023 23:00	18	19.0	16.7	-2.3
2A	3/13/2023 0:00	18	19.0	14.4	-4.7
2A	3/13/2023 1:00	18	19.0	14.4	-4.7
2A	3/13/2023 2:00	18	19.0	14.4	-4.7
2A	3/13/2023 3:00	18	19.0	14.4	-4.7
2A	3/13/2023 4:00	18	19.0	14.4	-4.7
2A	3/13/2023 5:00	18	19.0	14.4	-4.7
2A	3/13/2023 6:00	18	19.0	14.4	-4.7
2A	3/13/2023 7:00	18	19.0	14.4	-4.7
2A	3/13/2023 8:00	18	19.0	15.7	-3.3
2A	3/13/2023 9:00	18	19.0	15.7	-3.3
2A	3/13/2023 10:00	18	19.0	15.7	-3.3
2A	3/13/2023 11:00	18	19.0	15.7	-3.3
2A	3/13/2023 12:00	18	19.0	18.4	-0.6
2A	3/13/2023 13:00	18	19.0	18.4	-0.6
2A	3/13/2023 14:00	18	19.0	18.4	-0.6
2A	3/13/2023 15:00	18	19.0	18.4	-0.6
2A	3/13/2023 16:00	18	19.0	16.7	-2.3
2A	3/13/2023 17:00	18	19.0	16.7	-2.3
2A	3/13/2023 18:00	18	19.0	16.7	-2.3
2A	3/13/2023 19:00	18	19.0	16.7	-2.3
2A	3/13/2023 20:00	19	19.0	19.5	0.5
2A	3/13/2023 21:00	19	19.0	19.5	0.5
2A	3/13/2023 22:00	19	19.0	19.5	0.5
2A	3/13/2023 23:00	19	19.0	19.5	0.5
2A	3/14/2023 0:00	19	19.0	17.2	-1.8
2A	3/14/2023 1:00	19	19.0	17.2	-1.8
2A	3/14/2023 2:00	19	19.0	17.2	-1.8
2A	3/14/2023 3:00	19	19.0	17.2	-1.8

2A	3/14/2023 4:00	19	19.0	17.2	-1.8
2A	3/14/2023 5:00	19	19.0	17.2	-1.8
2A	3/14/2023 6:00	19	19.0	17.2	-1.8
2A	3/14/2023 7:00	19	19.0	17.2	-1.8
2A	3/14/2023 8:00	19	19.0	18.4	-0.6
2A	3/14/2023 9:00	19	19.0	18.4	-0.6
2A	3/14/2023 10:00	19	19.0	18.4	-0.6
2A	3/14/2023 11:00	19	19.0	18.4	-0.6
2A	3/14/2023 12:00	19	19.0	21.0	2.0
2A	3/14/2023 13:00	19	19.0	21.0	2.0
2A	3/14/2023 14:00	19	19.0	21.0	2.0
2A	3/14/2023 15:00	19	19.0	21.0	2.0
2A	3/14/2023 16:00	19	19.0	19.5	0.5
2A	3/14/2023 17:00	19	19.0	19.5	0.5
2A	3/14/2023 18:00	19	19.0	19.5	0.5
2A	3/14/2023 19:00	19	19.0	19.5	0.5
2A	3/14/2023 20:00	13	19.0	17.8	-1.2
2A	3/14/2023 21:00	14	19.0	17.8	-1.2
2A	3/14/2023 22:00	14	19.0	17.8	-1.2
2A	3/14/2023 23:00	14	19.0	17.8	-1.2
2A	3/15/2023 0:00	15	19.0	17.2	-1.8
2A	3/15/2023 1:00	15	19.0	17.2	-1.8
2A	3/15/2023 2:00	15	19.0	17.2	-1.8
2A	3/15/2023 3:00	15	19.0	17.2	-1.8
2A	3/15/2023 4:00	15	18.0	17.2	-0.8
2A	3/15/2023 5:00	15	18.0	17.2	-0.8
2A	3/15/2023 6:00	15	18.0	17.2	-0.8
2A	3/15/2023 7:00	15	18.0	17.2	-0.8
2A	3/15/2023 8:00	15	18.0	10.5	-7.5
2A	3/15/2023 9:00	15	18.0	10.5	-7.5
2A	3/15/2023 10:00	15	18.0	10.5	-7.5
2A	3/15/2023 11:00	15	18.0	10.5	-7.5
2A	3/15/2023 12:00	15	18.0	13.1	-4.9
2A	3/15/2023 13:00	15	18.0	13.1	-4.9
2A	3/15/2023 14:00	15	18.0	13.1	-4.9
2A	3/15/2023 15:00	15	18.0	13.1	-4.9
2A	3/15/2023 16:00	15	18.0	13.9	-4.1
2A	3/15/2023 17:00	15	18.0	13.9	-4.1
2A	3/15/2023 18:00	15	18.0	13.9	-4.1
2A	3/15/2023 19:00	15	18.0	13.9	-4.1
2A	3/15/2023 20:00	16	19.0	16.7	-2.3
2A	3/15/2023 21:00	16	19.0	16.7	-2.3
2A	3/15/2023 22:00	16	19.0	16.7	-2.3
2A	3/15/2023 23:00	16	19.0	16.7	-2.3
2A	3/16/2023 0:00	16	19.0	17.2	-1.8
2A	3/16/2023 1:00	16	19.0	17.2	-1.8
2A	3/16/2023 2:00	16	19.0	17.2	-1.8

2A	3/16/2023 3:00	16	19.0	17.2	-1.8
2A	3/16/2023 4:00	16	19.0	17.2	-1.8
2A	3/16/2023 5:00	16	19.0	17.2	-1.8
2A	3/16/2023 6:00	16	19.0	17.2	-1.8
2A	3/16/2023 7:00	16	19.0	17.2	-1.8
2A	3/16/2023 8:00	17	19.0	15.7	-3.3
2A	3/16/2023 9:00	15	19.0	15.7	-3.3
2A	3/16/2023 10:00	15	19.0	15.7	-3.3
2A	3/16/2023 11:00	15	19.0	15.7	-3.3
2A	3/16/2023 12:00	15	19.0	14.2	-4.8
2A	3/16/2023 13:00	15	19.0	15.7	-3.3
2A	3/16/2023 14:00	15	19.0	15.7	-3.3
2A	3/16/2023 15:00	15	19.0	15.7	-3.3
2A	3/16/2023 16:00	15	19.0	16.7	-2.3
2A	3/16/2023 17:00	15	19.0	16.7	-2.3
2A	3/16/2023 18:00	15	19.0	16.7	-2.3
2A	3/16/2023 19:00	15	19.0	16.7	-2.3
2A	3/16/2023 20:00	15	19.0	16.7	-2.3
2A	3/16/2023 21:00	16	19.0	16.7	-2.3
2A	3/16/2023 22:00	16	19.0	16.7	-2.3
2A	3/16/2023 23:00	16	19.0	16.7	-2.3
2A	3/17/2023 0:00	16	19.0	14.4	-4.7
2A	3/17/2023 1:00	16	19.0	14.4	-4.7
2A	3/17/2023 2:00	16	19.0	14.4	-4.7
2A	3/17/2023 3:00	16	19.0	14.4	-4.7
2A	3/17/2023 4:00	16	19.0	14.4	-4.7
2A	3/17/2023 5:00	16	19.0	14.4	-4.7
2A	3/17/2023 6:00	16	19.0	14.4	-4.7
2A	3/17/2023 7:00	16	19.0	14.4	-4.7
2A	3/17/2023 8:00	16	19.0	13.1	-5.9
2A	3/17/2023 9:00	16	19.0	13.1	-5.9
2A	3/17/2023 10:00	16	19.0	13.1	-5.9
2A	3/17/2023 11:00	16	19.0	13.1	-5.9
2A	3/17/2023 12:00	16	19.0	15.7	-3.3
2A	3/17/2023 13:00	16	19.0	15.7	-3.3
2A	3/17/2023 14:00	16	19.0	15.7	-3.3
2A	3/17/2023 15:00	16	19.0	15.7	-3.3
2A	3/17/2023 16:00	17	19.0	13.9	-5.1
2A	3/17/2023 17:00	17	19.0	13.9	-5.1
2A	3/17/2023 18:00	17	19.0	13.9	-5.1
2A	3/17/2023 19:00	17	19.0	13.9	-5.1
2A	3/17/2023 20:00	17	19.0	19.5	0.5
2A	3/17/2023 21:00	17	19.0	19.5	0.5
2A	3/17/2023 22:00	17	19.0	19.5	0.5
2A	3/17/2023 23:00	17	19.0	19.5	0.5
2A	3/18/2023 0:00	17	19.0	17.2	-1.8
2A	3/18/2023 1:00	17	19.0	17.2	-1.8

2A	3/18/2023 2:00	18	19.0	17.2	-1.8
2A	3/18/2023 3:00	18	19.0	17.2	-1.8
2A	3/18/2023 4:00	18	19.0	17.2	-1.8
2A	3/18/2023 5:00	18	19.0	17.2	-1.8
2A	3/18/2023 6:00	18	19.0	17.2	-1.8
2A	3/18/2023 7:00	18	19.0	17.2	-1.8
2A	3/18/2023 8:00	18	19.0	18.4	-0.6
2A	3/18/2023 9:00	18	19.0	18.4	-0.6
2A	3/18/2023 10:00	18	19.0	18.4	-0.6
2A	3/18/2023 11:00	18	19.0	18.4	-0.6
2A	3/18/2023 12:00	18	19.0	21.0	2.0
2A	3/18/2023 13:00	18	19.0	21.0	2.0
2A	3/18/2023 14:00	18	19.0	21.0	2.0
2A	3/18/2023 15:00	18	19.0	21.0	2.0
2A	3/18/2023 16:00	18	19.0	16.7	-2.3
2A	3/18/2023 17:00	18	19.0	16.7	-2.3
2A	3/18/2023 18:00	18	19.0	16.7	-2.3
2A	3/18/2023 19:00	18	19.0	16.7	-2.3
2A	3/18/2023 20:00	15	19.0	22.3	3.3
2A	3/18/2023 21:00	15	19.0	22.3	3.3
2A	3/18/2023 22:00	15	19.0	22.3	3.3
2A	3/18/2023 23:00	15	19.0	22.3	3.3
2A	3/19/2023 0:00	15	19.0	20.1	1.1
2A	3/19/2023 1:00	15	19.0	20.1	1.1
2A	3/19/2023 2:00	15	19.0	20.1	1.1
2A	3/19/2023 3:00	15	19.0	20.1	1.1
2A	3/19/2023 4:00	15	19.0	20.1	1.1
2A	3/19/2023 5:00	15	19.0	20.1	1.1
2A	3/19/2023 6:00	15	19.0	20.1	1.1
2A	3/19/2023 7:00	15	19.0	20.1	1.1
2A	3/19/2023 8:00	15	19.0	18.4	-0.6
2A	3/19/2023 9:00	15	19.0	18.4	-0.6
2A	3/19/2023 10:00	15	19.0	18.4	-0.6
2A	3/19/2023 11:00	15	19.0	18.4	-0.6
2A	3/19/2023 12:00	16	19.0	21.0	2.0
2A	3/19/2023 13:00	16	19.0	21.0	2.0
2A	3/19/2023 14:00	16	19.0	21.0	2.0
2A	3/19/2023 15:00	16	19.0	21.0	2.0
2A	3/19/2023 16:00	17	19.0	16.7	-2.3
2A	3/19/2023 17:00	17	19.0	16.7	-2.3
2A	3/19/2023 18:00	17	19.0	16.7	-2.3
2A	3/19/2023 19:00	17	19.0	16.7	-2.3
2A	3/19/2023 20:00	17	19.0	16.7	-2.3
2A	3/19/2023 21:00	17	19.0	16.7	-2.3
2A	3/19/2023 22:00	17	19.0	16.7	-2.3
2A	3/19/2023 23:00	17	19.0	16.7	-2.3
2A	3/20/2023 0:00	17	19.0	17.2	-1.8

2A	3/20/2023 1:00	17	19.0	17.2	-1.8
2A	3/20/2023 2:00	17	19.0	17.2	-1.8
2A	3/20/2023 3:00	17	19.0	17.2	-1.8
2A	3/20/2023 4:00	17	19.0	17.2	-1.8
2A	3/20/2023 5:00	17	19.0	17.2	-1.8
2A	3/20/2023 6:00	17	19.0	17.2	-1.8
2A	3/20/2023 7:00	17	19.0	17.2	-1.8
2A	3/20/2023 8:00	17	19.0	18.4	-0.6
2A	3/20/2023 9:00	17	19.0	18.4	-0.6
2A	3/20/2023 10:00	18	19.0	18.4	-0.6
2A	3/20/2023 11:00	19	19.0	18.4	-0.6
2A	3/20/2023 12:00	19	19.0	18.4	-0.6
2A	3/20/2023 13:00	19	19.0	18.4	-0.6
2A	3/20/2023 14:00	19	19.0	18.4	-0.6
2A	3/20/2023 15:00	19	19.0	18.4	-0.6
2A	3/20/2023 16:00	15	19.0	16.7	-2.3
2A	3/20/2023 17:00	16	19.0	16.7	-2.3
2A	3/20/2023 18:00	16	19.0	16.7	-2.3
2A	3/20/2023 19:00	17	19.0	16.7	-2.3
2A	3/20/2023 20:00	17	19.0	19.5	0.5
2A	3/20/2023 21:00	18	19.0	19.5	0.5
2A	3/20/2023 22:00	18	19.0	19.5	0.5
2A	3/20/2023 23:00	18	19.0	18.8	-0.2
2A	3/21/2023 0:00	18	19.0	13.8	-5.2
2A	3/21/2023 1:00	19	19.0	13.8	-5.2
2A	3/21/2023 2:00	19	19.0	13.8	-5.2
2A	3/21/2023 3:00	19	19.0	13.8	-5.2
2A	3/21/2023 4:00	19	19.0	13.8	-5.2
2A	3/21/2023 5:00	19	19.0	13.8	-5.2
2A	3/21/2023 6:00	19	19.0	13.8	-5.2
2A	3/21/2023 7:00	19	19.0	13.8	-5.2
2A	3/21/2023 8:00	19	19.0	13.1	-5.9
2A	3/21/2023 9:00	19	19.0	13.1	-5.9
2A	3/21/2023 10:00	19	19.0	13.1	-5.9
2A	3/21/2023 11:00	19	19.0	13.1	-5.9
2A	3/21/2023 12:00	19	19.0	13.1	-5.9
2A	3/21/2023 13:00	19	19.0	13.1	-5.9
2A	3/21/2023 14:00	19	19.0	13.1	-5.9
2A	3/21/2023 15:00	19	19.0	13.1	-5.9
2A	3/21/2023 16:00	19	19.0	13.9	-5.1
2A	3/21/2023 17:00	19	19.0	13.9	-5.1
2A	3/21/2023 18:00	19	19.0	13.9	-5.1
2A	3/21/2023 19:00	19	19.0	13.9	-5.1
2A	3/21/2023 20:00	18	19.0	11.1	-7.9
2A	3/21/2023 21:00	18	19.0	13.9	-5.1
2A	3/21/2023 22:00	18	19.0	13.9	-5.1
2A	3/21/2023 23:00	18	19.0	13.9	-5.1

2A	3/22/2023 0:00	18	19.0	12.9	-6.1
2A	3/22/2023 1:00	18	19.0	14.4	-4.7
2A	3/22/2023 2:00	18	19.0	14.4	-4.7
2A	3/22/2023 3:00	18	19.0	14.4	-4.7
2A	3/22/2023 4:00	18	19.0	14.4	-4.7
2A	3/22/2023 5:00	18	19.0	14.4	-4.7
2A	3/22/2023 6:00	18	19.0	14.4	-4.7
2A	3/22/2023 7:00	18	19.0	14.4	-4.7
2A	3/22/2023 8:00	18	19.0	18.4	-0.6
2A	3/22/2023 9:00	18	19.0	18.4	-0.6
2A	3/22/2023 10:00	18	19.0	18.4	-0.6
2A	3/22/2023 11:00	18	19.0	18.4	-0.6
2A	3/22/2023 12:00	18	19.0	18.4	-0.6
2A	3/22/2023 13:00	18	19.0	18.4	-0.6
2A	3/22/2023 14:00	18	19.0	18.4	-0.6
2A	3/22/2023 15:00	18	19.0	18.4	-0.6
2A	3/22/2023 16:00	18	19.0	16.7	-2.3
2A	3/22/2023 17:00	18	19.0	16.7	-2.3
2A	3/22/2023 18:00	18	19.0	16.7	-2.3
2A	3/22/2023 19:00	18	19.0	18.1	-0.9
2A	3/22/2023 20:00	18	19.0	16.7	-2.3
2A	3/22/2023 21:00	18	19.0	16.7	-2.3
2A	3/22/2023 22:00	18	19.0	16.7	-2.3
2A	3/22/2023 23:00	18	19.0	16.7	-2.3
2A	3/23/2023 0:00	18	19.0	17.2	-1.8
2A	3/23/2023 1:00	18	19.0	17.2	-1.8
2A	3/23/2023 2:00	18	19.0	17.2	-1.8
2A	3/23/2023 3:00	18	19.0	17.2	-1.8
2A	3/23/2023 4:00	18	19.0	17.2	-1.8
2A	3/23/2023 5:00	18	19.0	17.2	-1.8
2A	3/23/2023 6:00	18	19.0	17.2	-1.8
2A	3/23/2023 7:00	18	18.0	17.2	-0.8
2A	3/23/2023 8:00	18	18.0	13.1	-4.9
2A	3/23/2023 9:00	18	18.0	13.1	-4.9
2A	3/23/2023 10:00	18	18.0	13.1	-4.9
2A	3/23/2023 11:00	18	18.0	13.1	-4.9
2A	3/23/2023 12:00	18	18.0	13.1	-4.9
2A	3/23/2023 13:00	18	18.0	13.1	-4.9
2A	3/23/2023 14:00	18	18.0	13.1	-4.9
2A	3/23/2023 15:00	18	18.0	13.1	-4.9
2A	3/23/2023 16:00	18	18.0	13.9	-4.1
2A	3/23/2023 17:00	18	18.0	13.9	-4.1
2A	3/23/2023 18:00	18	18.0	13.9	-4.1
2A	3/23/2023 19:00	18	18.0	13.9	-4.1
2A	3/23/2023 20:00	18	18.0	11.1	-6.9
2A	3/23/2023 21:00	18	18.0	11.1	-6.9
2A	3/23/2023 22:00	17	18.0	11.1	-6.9

2A	3/23/2023 23:00	17	18.0	11.1	-6.9
2A	3/24/2023 0:00	17	18.0	11.5	-6.5
2A	3/24/2023 1:00	17	18.0	11.5	-6.5
2A	3/24/2023 2:00	17	18.0	11.5	-6.5
2A	3/24/2023 3:00	17	18.0	11.5	-6.5
2A	3/24/2023 4:00	17	18.0	11.5	-6.5
2A	3/24/2023 5:00	17	18.0	11.5	-6.5
2A	3/24/2023 6:00	17	18.0	11.5	-6.5
2A	3/24/2023 7:00	17	18.0	11.5	-6.5
2A	3/24/2023 8:00	17	18.0	13.1	-4.9
2A	3/24/2023 9:00	17	18.0	13.1	-4.9
2A	3/24/2023 10:00	17	18.0	13.1	-4.9
2A	3/24/2023 11:00	17	18.0	13.1	-4.9
2A	3/24/2023 12:00	17	18.0	15.7	-2.3
2A	3/24/2023 13:00	17	18.0	15.7	-2.3
2A	3/24/2023 14:00	17	18.0	15.7	-2.3
2A	3/24/2023 15:00	17	18.0	15.7	-2.3
2A	3/24/2023 16:00	17	18.0	13.9	-4.1
2A	3/24/2023 17:00	17	18.0	13.9	-4.1
2A	3/24/2023 18:00	17	18.0	13.9	-4.1
2A	3/24/2023 19:00	17	18.0	13.9	-4.1
2A	3/24/2023 20:00	16	18.0	16.7	-1.3
2A	3/24/2023 21:00	16	18.0	16.7	-1.3
2A	3/24/2023 22:00	16	18.0	16.7	-1.3
2A	3/24/2023 23:00	15	18.0	16.7	-1.3
2A	3/25/2023 0:00	15	18.0	17.2	-0.8
2A	3/25/2023 1:00	16	18.0	17.2	-0.8
2A	3/25/2023 2:00	16	18.0	17.2	-0.8
2A	3/25/2023 3:00	16	18.0	17.2	-0.8
2A	3/25/2023 4:00	16	18.0	17.2	-0.8
2A	3/25/2023 5:00	16	18.0	17.2	-0.8
2A	3/25/2023 6:00	16	18.0	17.2	-0.8
2A	3/25/2023 7:00	16	18.0	17.2	-0.8
2A	3/25/2023 8:00	16	19.0	18.4	-0.6
2A	3/25/2023 9:00	16	19.0	18.4	-0.6
2A	3/25/2023 10:00	16	19.0	18.4	-0.6
2A	3/25/2023 11:00	16	19.0	16.8	-2.2
2A	3/25/2023 12:00	16	19.0	15.2	-3.8
2A	3/25/2023 13:00	16	19.0	16.8	-2.2
2A	3/25/2023 14:00	16	19.0	18.4	-0.6
2A	3/25/2023 15:00	16	19.0	18.4	-0.6
2A	3/25/2023 16:00	16	19.0	15.0	-4.0
2A	3/25/2023 17:00	16	19.0	16.7	-2.3
2A	3/25/2023 18:00	16	19.0	16.7	-2.3
2A	3/25/2023 19:00	16	19.0	16.7	-2.3
2A	3/25/2023 20:00	16	19.0	19.5	0.5
2A	3/25/2023 21:00	16	19.0	19.5	0.5

2A	3/25/2023 22:00	16	19.0	19.5	0.5
2A	3/25/2023 23:00	16	19.0	19.5	0.5
2A	3/26/2023 0:00	16	19.0	18.4	-0.6
2A	3/26/2023 1:00	17	19.0	18.4	-0.6
2A	3/26/2023 2:00	17	19.0	18.4	-0.6
2A	3/26/2023 3:00	17	19.0	18.4	-0.6
2A	3/26/2023 4:00	17	19.0	18.4	-0.6
2A	3/26/2023 5:00	17	19.0	18.4	-0.6
2A	3/26/2023 6:00	17	19.0	18.4	-0.6
2A	3/26/2023 7:00	17	19.0	18.4	-0.6
2A	3/26/2023 8:00	17	19.0	21.0	2.0
2A	3/26/2023 9:00	17	19.0	21.0	2.0
2A	3/26/2023 10:00	17	19.0	21.0	2.0
2A	3/26/2023 11:00	17	19.0	21.0	2.0
2A	3/26/2023 12:00	17	19.0	21.0	2.0
2A	3/26/2023 13:00	17	19.0	21.0	2.0
2A	3/26/2023 14:00	15	19.0	21.0	2.0
2A	3/26/2023 15:00	15	19.0	19.4	0.4
2A	3/26/2023 16:00	15	19.0	19.5	0.5
2A	3/26/2023 17:00	15	19.0	17.8	-1.2
2A	3/26/2023 18:00	15	19.0	19.5	0.5
2A	3/26/2023 19:00	15	19.0	19.5	0.5
2A	3/26/2023 20:00	15	19.0	16.7	-2.3
2A	3/26/2023 21:00	15	19.0	16.7	-2.3
2A	3/26/2023 22:00	16	19.0	16.7	-2.3
2A	3/26/2023 23:00	16	19.0	16.7	-2.3
2A	3/27/2023 0:00	16	19.0	15.8	-3.2
2A	3/27/2023 1:00	16	19.0	17.2	-1.8
2A	3/27/2023 2:00	16	19.0	17.2	-1.8
2A	3/27/2023 3:00	16	19.0	17.2	-1.8
2A	3/27/2023 4:00	16	19.0	17.2	-1.8
2A	3/27/2023 5:00	16	19.0	17.2	-1.8
2A	3/27/2023 6:00	16	19.0	17.2	-1.8
2A	3/27/2023 7:00	16	19.0	17.2	-1.8
2A	3/27/2023 8:00	16	19.0	13.1	-5.9
2A	3/27/2023 9:00	16	19.0	13.1	-5.9
2A	3/27/2023 10:00	16	19.0	13.1	-5.9
2A	3/27/2023 11:00	16	19.0	13.1	-5.9
2A	3/27/2023 12:00	16	19.0	15.7	-3.3
2A	3/27/2023 13:00	16	19.0	15.7	-3.3
2A	3/27/2023 14:00	16	19.0	15.7	-3.3
2A	3/27/2023 15:00	16	19.0	15.7	-3.3
2A	3/27/2023 16:00	16	19.0	16.7	-2.3
2A	3/27/2023 17:00	16	19.0	16.7	-2.3
2A	3/27/2023 18:00	16	19.0	16.7	-2.3
2A	3/27/2023 19:00	16	19.0	16.7	-2.3
2A	3/27/2023 20:00	15	19.0	13.9	-5.1

2A	3/27/2023 21:00	15	19.0	13.9	-5.1
2A	3/27/2023 22:00	15	19.0	13.9	-5.1
2A	3/27/2023 23:00	15	19.0	13.9	-5.1
2A	3/28/2023 0:00	15	19.0	11.5	-7.5
2A	3/28/2023 1:00	15	19.0	11.5	-7.5
2A	3/28/2023 2:00	15	19.0	11.5	-7.5
2A	3/28/2023 3:00	15	19.0	11.5	-7.5
2A	3/28/2023 4:00	15	19.0	11.5	-7.5
2A	3/28/2023 5:00	15	19.0	11.5	-7.5
2A	3/28/2023 6:00	15	19.0	11.5	-7.5
2A	3/28/2023 7:00	15	19.0	11.5	-7.5
2A	3/28/2023 8:00	15	19.0	10.5	-8.5
2A	3/28/2023 9:00	15	19.0	10.5	-8.5
2A	3/28/2023 10:00	15	19.0	10.5	-8.5
2A	3/28/2023 11:00	15	19.0	10.5	-8.5
2A	3/28/2023 12:00	15	19.0	13.1	-5.9
2A	3/28/2023 13:00	15	19.0	13.1	-5.9
2A	3/28/2023 14:00	15	19.0	13.1	-5.9
2A	3/28/2023 15:00	15	19.0	13.1	-5.9
2A	3/28/2023 16:00	15	19.0	13.9	-5.1
2A	3/28/2023 17:00	15	19.0	13.9	-5.1
2A	3/28/2023 18:00	15	19.0	13.9	-5.1
2A	3/28/2023 19:00	18	19.0	13.9	-5.1
2A	3/28/2023 20:00	18	19.0	16.7	-2.3
2A	3/28/2023 21:00	18	19.0	16.7	-2.3
2A	3/28/2023 22:00	18	19.0	16.7	-2.3
2A	3/28/2023 23:00	18	19.0	16.7	-2.3
2A	3/29/2023 0:00	18	19.0	11.5	-7.5
2A	3/29/2023 1:00	18	19.0	11.5	-7.5
2A	3/29/2023 2:00	19	19.0	11.5	-7.5
2A	3/29/2023 3:00	19	19.0	11.5	-7.5
2A	3/29/2023 4:00	19	19.0	11.5	-7.5
2A	3/29/2023 5:00	19	19.0	11.5	-7.5
2A	3/29/2023 6:00	19	19.0	11.5	-7.5
2A	3/29/2023 7:00	19	19.0	11.5	-7.5
2A	3/29/2023 8:00	19	19.0	15.7	-3.3
2A	3/29/2023 9:00	19	19.0	15.7	-3.3
2A	3/29/2023 10:00	19	19.0	15.7	-3.3
2A	3/29/2023 11:00	19	19.0	15.7	-3.3
2A	3/29/2023 12:00	19	19.0	18.4	-0.6
2A	3/29/2023 13:00	19	19.0	18.4	-0.6
2A	3/29/2023 14:00	19	19.0	18.4	-0.6
2A	3/29/2023 15:00	15	19.0	18.4	-0.6
2A	3/29/2023 16:00	15	19.0	16.7	-2.3
2A	3/29/2023 17:00	15	19.0	16.7	-2.3
2A	3/29/2023 18:00	15	19.0	16.7	-2.3
2A	3/29/2023 19:00	15	19.0	16.7	-2.3

2A	3/29/2023 20:00	15	19.0	6.7	-12.3
2A	3/29/2023 21:00	15	19.0	6.7	-12.3
2A	3/29/2023 22:00	15	19.0	6.7	-12.3
2A	3/29/2023 23:00	15	19.0	6.7	-12.3
2A	3/30/2023 0:00	15	19.0	4.6	-14.4
2A	3/30/2023 1:00	15	19.0	4.6	-14.4
2A	3/30/2023 2:00	15	19.0	4.6	-14.4
2A	3/30/2023 3:00	15	19.0	4.6	-14.4
2A	3/30/2023 4:00	15	19.0	4.6	-14.4
2A	3/30/2023 5:00	15	19.0	4.6	-14.4
2A	3/30/2023 6:00	15	19.0	4.6	-14.4
2A	3/30/2023 7:00	16	19.0	17.2	-1.8
2A	3/30/2023 8:00	16	19.0	15.7	-3.3
2A	3/30/2023 9:00	16	19.0	15.7	-3.3
2A	3/30/2023 10:00	16	19.0	15.7	-3.3
2A	3/30/2023 11:00	16	19.0	11.0	-8.0
2A	3/30/2023 12:00	16	19.0	15.7	-3.3
2A	3/30/2023 13:00	16	19.0	14.2	-4.8
2A	3/30/2023 14:00	16	19.0	12.6	-6.4
2A	3/30/2023 15:00	16	19.0	15.7	-3.3
2A	3/30/2023 16:00	16	19.0	11.7	-7.3
2A	3/30/2023 17:00	16	19.0	16.7	-2.3
2A	3/30/2023 18:00	16	19.0	16.7	-2.3
2A	3/30/2023 19:00	16	19.0	16.7	-2.3
2A	3/30/2023 20:00	16	19.0	16.7	-2.3
2A	3/30/2023 21:00	16	19.0	16.7	-2.3
2A	3/30/2023 22:00	16	19.0	16.7	-2.3
2A	3/30/2023 23:00	16	19.0	16.7	-2.3
2A	3/31/2023 0:00	16	19.0	17.2	-1.8
2A	3/31/2023 1:00	16	19.0	17.2	-1.8
2A	3/31/2023 2:00	17	19.0	17.2	-1.8
2A	3/31/2023 3:00	17	19.0	17.2	-1.8
2A	3/31/2023 4:00	17	19.0	17.2	-1.8
2A	3/31/2023 5:00	17	19.0	17.2	-1.8
2A	3/31/2023 6:00	17	19.0	17.2	-1.8
2A	3/31/2023 7:00	17	19.0	17.2	-1.8
2A	3/31/2023 8:00	15	19.0	13.1	-5.9
2A	3/31/2023 9:00	15	19.0	13.1	-5.9
2A	3/31/2023 10:00	15	19.0	10.0	-9.0
2A	3/31/2023 11:00	15	19.0	13.1	-5.9
2A	3/31/2023 12:00	15	19.0	13.1	-5.9
2A	3/31/2023 13:00	15	19.0	10.0	-9.0
2A	3/31/2023 14:00	15	19.0	11.5	-7.5
2A	3/31/2023 15:00	15	19.0	13.1	-5.9
2A	3/31/2023 16:00	15	19.0	13.9	-5.1
2A	3/31/2023 17:00	15	19.0	13.9	-5.1
2A	3/31/2023 18:00	15	19.0	13.9	-5.1

2A	3/31/2023 19:00	15	19.0	13.9	-5.1
2A	3/31/2023 20:00	14	19.0	14.6	-4.4
2A	3/31/2023 21:00	14	19.0	13.9	-5.1
2A	3/31/2023 22:00	14	19.0	13.9	-5.1
2A	3/31/2023 23:00	14	19.0	15.3	-3.7
2A	4/1/2023 0:00	15	19.0	17.2	-1.8
2A	4/1/2023 1:00	15	19.0	17.2	-1.8
2A	4/1/2023 2:00	15	19.0	17.2	-1.8
2A	4/1/2023 3:00	15	19.0	17.2	-1.8
2A	4/1/2023 4:00	15	19.0	17.2	-1.8
2A	4/1/2023 5:00	15	19.0	17.2	-1.8
2A	4/1/2023 6:00	15	19.0	17.2	-1.8
2A	4/1/2023 7:00	15	19.0	17.2	-1.8
2A	4/1/2023 8:00	16	19.0	14.7	-4.3
2A	4/1/2023 9:00	16	19.0	14.7	-4.3
2A	4/1/2023 10:00	16	19.0	14.7	-4.3
2A	4/1/2023 11:00	16	19.0	14.7	-4.3
2A	4/1/2023 12:00	16	19.0	14.7	-4.3
2A	4/1/2023 13:00	16	19.0	14.7	-4.3
2A	4/1/2023 14:00	16	19.0	14.7	-4.3
2A	4/1/2023 15:00	16	19.0	14.7	-4.3
2A	4/1/2023 16:00	16	19.0	13.9	-5.1
2A	4/1/2023 17:00	16	19.0	13.9	-5.1
2A	4/1/2023 18:00	16	19.0	13.9	-5.1
2A	4/1/2023 19:00	16	19.0	13.9	-5.1
2A	4/1/2023 20:00	15	19.0	13.9	-5.1
2A	4/1/2023 21:00	15	19.0	13.9	-5.1
2A	4/1/2023 22:00	15	19.0	13.9	-5.1
2A	4/1/2023 23:00	15	19.0	13.9	-5.1
2A	4/2/2023 0:00	15	19.0	14.4	-4.7
2A	4/2/2023 1:00	15	19.0	14.4	-4.7
2A	4/2/2023 2:00	15	19.0	14.4	-4.7
2A	4/2/2023 3:00	15	19.0	14.4	-4.7
2A	4/2/2023 4:00	15	19.0	14.4	-4.7
2A	4/2/2023 5:00	15	19.0	14.4	-4.7
2A	4/2/2023 6:00	16	19.0	14.4	-4.7
2A	4/2/2023 7:00	16	19.0	14.4	-4.7
2A	4/2/2023 8:00	16	19.0	16.8	-2.2
2A	4/2/2023 9:00	16	19.0	16.8	-2.2
2A	4/2/2023 10:00	16	19.0	16.8	-2.2
2A	4/2/2023 11:00	16	19.0	16.8	-2.2
2A	4/2/2023 12:00	16	19.0	16.8	-2.2
2A	4/2/2023 13:00	16	19.0	16.8	-2.2
2A	4/2/2023 14:00	16	19.0	16.8	-2.2
2A	4/2/2023 15:00	16	19.0	16.8	-2.2
2A	4/2/2023 16:00	16	19.0	13.9	-5.1
2A	4/2/2023 17:00	16	19.0	13.9	-5.1

2A	4/2/2023 18:00	16	19.0	13.9	-5.1
2A	4/2/2023 19:00	14	19.0	13.9	-5.1
2A	4/2/2023 20:00	14	19.0	13.9	-5.1
2A	4/2/2023 21:00	15	19.0	13.9	-5.1
2A	4/2/2023 22:00	15	19.0	13.9	-5.1
2A	4/2/2023 23:00	15	19.0	13.9	-5.1
2A	4/3/2023 0:00	15	19.0	17.2	-1.8
2A	4/3/2023 1:00	15	19.0	17.2	-1.8
2A	4/3/2023 2:00	15	19.0	17.2	-1.8
2A	4/3/2023 3:00	15	19.0	17.2	-1.8
2A	4/3/2023 4:00	16	19.0	17.2	-1.8
2A	4/3/2023 5:00	16	19.0	17.2	-1.8
2A	4/3/2023 6:00	16	19.0	17.2	-1.8
2A	4/3/2023 7:00	16	19.0	17.2	-1.8
2A	4/3/2023 8:00	16	19.0	13.1	-5.9
2A	4/3/2023 9:00	16	19.0	13.1	-5.9
2A	4/3/2023 10:00	16	19.0	13.1	-5.9
2A	4/3/2023 11:00	16	19.0	13.1	-5.9
2A	4/3/2023 12:00	15	18.0	13.1	-4.9
2A	4/3/2023 13:00	15	18.0	13.1	-4.9
2A	4/3/2023 14:00	15	18.0	13.1	-4.9
2A	4/3/2023 15:00	15	18.0	13.1	-4.9
2A	4/3/2023 16:00	15	18.0	13.9	-4.1
2A	4/3/2023 17:00	15	18.0	13.9	-4.1
2A	4/3/2023 18:00	15	18.0	13.9	-4.1
2A	4/3/2023 19:00	15	18.0	13.9	-4.1
2A	4/3/2023 20:00	16	19.0	13.9	-5.1
2A	4/3/2023 21:00	16	19.0	13.9	-5.1
2A	4/3/2023 22:00	16	19.0	13.9	-5.1
2A	4/3/2023 23:00	16	19.0	13.9	-5.1
2A	4/4/2023 0:00	16	19.0	14.4	-4.7
2A	4/4/2023 1:00	16	19.0	14.4	-4.7
2A	4/4/2023 2:00	16	19.0	14.4	-4.7
2A	4/4/2023 3:00	16	19.0	14.4	-4.7
2A	4/4/2023 4:00	17	19.0	14.4	-4.7
2A	4/4/2023 5:00	17	19.0	14.4	-4.7
2A	4/4/2023 6:00	17	19.0	14.4	-4.7
2A	4/4/2023 7:00	17	19.0	14.4	-4.7
2A	4/4/2023 8:00	17	19.0	13.1	-5.9
2A	4/4/2023 9:00	17	19.0	15.7	-3.3
2A	4/4/2023 10:00	18	19.0	15.7	-3.3
2A	4/4/2023 11:00	18	19.0	15.7	-3.3
2A	4/4/2023 12:00	18	19.0	18.4	-0.6
2A	4/4/2023 13:00	18	19.0	18.4	-0.6
2A	4/4/2023 14:00	17	19.0	18.4	-0.6
2A	4/4/2023 15:00	17	19.0	18.4	-0.6
2A	4/4/2023 16:00	17	19.0	19.5	0.5

2A	4/4/2023 17:00	17	19.0	19.5	0.5
2A	4/4/2023 18:00	17	19.0	19.5	0.5
2A	4/4/2023 19:00	15	17.0	19.5	2.5
2A	4/4/2023 20:00	15	17.0	16.7	-0.3
2A	4/4/2023 21:00	15	17.0	16.7	-0.3
2A	4/4/2023 22:00	15	17.0	16.7	-0.3
2A	4/4/2023 23:00	15	17.0	16.7	-0.3
2A	4/5/2023 0:00	16	17.0	11.5	-5.5
2A	4/5/2023 1:00	16	17.0	11.5	-5.5
2A	4/5/2023 2:00	16	17.0	11.5	-5.5
2A	4/5/2023 3:00	16	17.0	11.5	-5.5
2A	4/5/2023 4:00	16	17.0	11.5	-5.5
2A	4/5/2023 5:00	16	17.0	11.5	-5.5
2A	4/5/2023 6:00	16	17.0	11.5	-5.5
2A	4/5/2023 7:00	16	17.0	11.5	-5.5
2A	4/5/2023 8:00	16	19.0	15.7	-3.3
2A	4/5/2023 9:00	16	19.0	15.7	-3.3
2A	4/5/2023 10:00	16	19.0	15.7	-3.3
2A	4/5/2023 11:00	16	19.0	15.7	-3.3
2A	4/5/2023 12:00	16	19.0	18.4	-0.6
2A	4/5/2023 13:00	16	19.0	18.4	-0.6
2A	4/5/2023 14:00	16	19.0	18.4	-0.6
2A	4/5/2023 15:00	16	19.0	18.4	-0.6
2A	4/5/2023 16:00	16	19.0	16.7	-2.3
2A	4/5/2023 17:00	16	19.0	16.7	-2.3
2A	4/5/2023 18:00	16	19.0	16.7	-2.3
2A	4/5/2023 19:00	15	18.0	16.7	-1.3
2A	4/5/2023 20:00	16	18.0	15.6	-2.4
2A	4/5/2023 21:00	16	18.0	15.6	-2.4
2A	4/5/2023 22:00	16	18.0	15.6	-2.4
2A	4/5/2023 23:00	16	18.0	15.6	-2.4
2A	4/6/2023 0:00	16	18.0	14.4	-3.7
2A	4/6/2023 1:00	16	18.0	14.4	-3.7
2A	4/6/2023 2:00	16	18.0	14.4	-3.7
2A	4/6/2023 3:00	16	18.0	14.4	-3.7
2A	4/6/2023 4:00	16	18.0	14.4	-3.7
2A	4/6/2023 5:00	16	17.0	14.4	-2.7
2A	4/6/2023 6:00	16	17.0	14.4	-2.7
2A	4/6/2023 7:00	16	17.0	14.4	-2.7
2A	4/6/2023 8:00	16	17.0	12.6	-4.4
2A	4/6/2023 9:00	16	17.0	10.5	-6.5
2A	4/6/2023 10:00	16	17.0	10.5	-6.5
2A	4/6/2023 11:00	16	17.0	10.5	-6.5
2A	4/6/2023 12:00	16	17.0	10.5	-6.5
2A	4/6/2023 13:00	16	17.0	10.5	-6.5
2A	4/6/2023 14:00	16	17.0	10.5	-6.5
2A	4/6/2023 15:00	16	17.0	10.5	-6.5

2A	4/6/2023 16:00	16	17.0	13.4	-3.6
2A	4/6/2023 17:00	16	17.0	13.4	-3.6
2A	4/6/2023 18:00	16	17.0	13.4	-3.6
2A	4/6/2023 19:00	16	17.0	13.4	-3.6
2A	4/6/2023 20:00	17	17.0	15.6	-1.4
2A	4/6/2023 21:00	17	17.0	15.6	-1.4
2A	4/6/2023 22:00	17	17.0	15.6	-1.4
2A	4/6/2023 23:00	17	17.0	15.6	-1.4
2A	4/7/2023 0:00	19	17.0	14.4	-2.7
2A	4/7/2023 1:00	19	17.0	14.4	-2.7
2A	4/7/2023 2:00	19	17.0	14.4	-2.7
2A	4/7/2023 3:00	19	17.0	14.4	-2.7
2A	4/7/2023 4:00	19	17.0	14.4	-2.7
2A	4/7/2023 5:00	19	17.0	14.4	-2.7
2A	4/7/2023 6:00	19	17.0	14.4	-2.7
2A	4/7/2023 7:00	19	17.0	14.4	-2.7
2A	4/7/2023 8:00	17	19.0	15.7	-3.3
2A	4/7/2023 9:00	17	19.0	15.7	-3.3
2A	4/7/2023 10:00	17	19.0	15.7	-3.3
2A	4/7/2023 11:00	17	19.0	15.7	-3.3
2A	4/7/2023 12:00	17	19.0	17.1	-2.0
2A	4/7/2023 13:00	17	19.0	18.4	-0.6
2A	4/7/2023 14:00	17	19.0	18.4	-0.6
2A	4/7/2023 15:00	16	19.0	18.4	-0.6
2A	4/7/2023 16:00	16	19.0	15.6	-3.4
2A	4/7/2023 17:00	16	19.0	15.6	-3.4
2A	4/7/2023 18:00	16	19.0	15.6	-3.4
2A	4/7/2023 19:00	18	19.0	15.6	-3.4
2A	4/7/2023 20:00	18	19.0	16.7	-2.3
2A	4/7/2023 21:00	18	19.0	16.7	-2.3
2A	4/7/2023 22:00	18	19.0	16.7	-2.3
2A	4/7/2023 23:00	18	19.0	16.7	-2.3
2A	4/8/2023 0:00	19	19.0	20.1	1.1
2A	4/8/2023 1:00	19	19.0	20.1	1.1
2A	4/8/2023 2:00	19	19.0	20.1	1.1
2A	4/8/2023 3:00	19	19.0	20.1	1.1
2A	4/8/2023 4:00	19	19.0	20.1	1.1
2A	4/8/2023 5:00	19	19.0	20.1	1.1
2A	4/8/2023 6:00	19	19.0	20.1	1.1
2A	4/8/2023 7:00	19	19.0	20.1	1.1
2A	4/8/2023 8:00	16	18.0	21.0	3.0
2A	4/8/2023 9:00	16	18.0	21.0	3.0
2A	4/8/2023 10:00	16	18.0	21.0	3.0
2A	4/8/2023 11:00	16	18.0	21.0	3.0
2A	4/8/2023 12:00	16	18.0	18.4	0.4
2A	4/8/2023 13:00	16	18.0	16.8	-1.2
2A	4/8/2023 14:00	16	18.0	18.4	0.4

2A	4/8/2023 15:00	16	18.0	16.8	-1.2
2A	4/8/2023 16:00	16	18.0	15.0	-3.0
2A	4/8/2023 17:00	16	18.0	16.7	-1.3
2A	4/8/2023 18:00	16	18.0	16.7	-1.3
2A	4/8/2023 19:00	16	18.0	16.7	-1.3
2A	4/8/2023 20:00	17	18.0	13.9	-4.1
2A	4/8/2023 21:00	17	18.0	13.9	-4.1
2A	4/8/2023 22:00	17	18.0	13.9	-4.1
2A	4/8/2023 23:00	17	18.0	13.9	-4.1
2A	4/9/2023 0:00	17	18.0	16.1	-1.9
2A	4/9/2023 1:00	17	18.0	16.1	-1.9
2A	4/9/2023 2:00	17	18.0	16.1	-1.9
2A	4/9/2023 3:00	17	18.0	16.1	-1.9
2A	4/9/2023 4:00	17	18.0	16.1	-1.9
2A	4/9/2023 5:00	17	18.0	16.1	-1.9
2A	4/9/2023 6:00	17	18.0	16.1	-1.9
2A	4/9/2023 7:00	17	18.0	16.1	-1.9
2A	4/9/2023 8:00	17	18.0	14.7	-3.3
2A	4/9/2023 9:00	17	18.0	14.7	-3.3
2A	4/9/2023 10:00	17	18.0	14.7	-3.3
2A	4/9/2023 11:00	17	18.0	14.7	-3.3
2A	4/9/2023 12:00	17	18.0	13.1	-4.9
2A	4/9/2023 13:00	17	18.0	11.5	-6.5
2A	4/9/2023 14:00	17	18.0	11.5	-6.5
2A	4/9/2023 15:00	17	18.0	13.1	-4.9
2A	4/9/2023 16:00	17	18.0	13.9	-4.1
2A	4/9/2023 17:00	17	18.0	13.9	-4.1
2A	4/9/2023 18:00	17	18.0	13.9	-4.1
2A	4/9/2023 19:00	17	18.0	13.9	-4.1
2A	4/9/2023 20:00	17	18.0	16.7	-1.3
2A	4/9/2023 21:00	17	18.0	16.7	-1.3
2A	4/9/2023 22:00	17	18.0	16.7	-1.3
2A	4/9/2023 23:00	17	18.0	19.5	1.5
2A	4/10/2023 0:00	17	18.0	20.1	2.1
2A	4/10/2023 1:00	17	18.0	20.1	2.1
2A	4/10/2023 2:00	17	18.0	20.1	2.1
2A	4/10/2023 3:00	17	18.0	20.1	2.1
2A	4/10/2023 4:00	17	18.0	20.1	2.1
2A	4/10/2023 5:00	17	18.0	20.1	2.1
2A	4/10/2023 6:00	17	18.0	20.1	2.1
2A	4/10/2023 7:00	17	18.0	20.1	2.1
2A	4/10/2023 8:00	17	18.0	16.8	-1.2
2A	4/10/2023 9:00	17	18.0	16.8	-1.2
2A	4/10/2023 10:00	17	18.0	16.8	-1.2
2A	4/10/2023 11:00	17	18.0	16.8	-1.2
2A	4/10/2023 12:00	17	18.0	18.9	0.9
2A	4/10/2023 13:00	17	18.0	18.9	0.9

2A	4/10/2023 14:00	17	18.0	18.9	0.9
2A	4/10/2023 15:00	17	18.0	18.9	0.9
2A	4/10/2023 16:00	17	18.0	17.8	-0.2
2A	4/10/2023 17:00	17	18.0	17.8	-0.2
2A	4/10/2023 18:00	17	18.0	17.8	-0.2
2A	4/10/2023 19:00	17	18.0	17.8	-0.2
2A	4/10/2023 20:00	17	18.0	13.9	-4.1
2A	4/10/2023 21:00	17	19.0	13.9	-5.1
2A	4/10/2023 22:00	17	19.0	13.9	-5.1
2A	4/10/2023 23:00	17	19.0	13.9	-5.1
2A	4/11/2023 0:00	17	19.0	14.4	-4.7
2A	4/11/2023 1:00	17	19.0	17.2	-1.8
2A	4/11/2023 2:00	18	19.0	17.2	-1.8
2A	4/11/2023 3:00	18	19.0	17.2	-1.8
2A	4/11/2023 4:00	18	19.0	17.2	-1.8
2A	4/11/2023 5:00	18	19.0	17.2	-1.8
2A	4/11/2023 6:00	18	19.0	17.2	-1.8
2A	4/11/2023 7:00	18	19.0	17.2	-1.8
2A	4/11/2023 8:00	18	19.0	14.7	-4.3
2A	4/11/2023 9:00	18	19.0	14.7	-4.3
2A	4/11/2023 10:00	18	19.0	14.7	-4.3
2A	4/11/2023 11:00	18	19.0	13.1	-5.9
2A	4/11/2023 12:00	18	19.0	21.0	2.0
2A	4/11/2023 13:00	18	19.0	21.0	2.0
2A	4/11/2023 14:00	18	19.0	21.0	2.0
2A	4/11/2023 15:00	18	19.0	21.0	2.0
2A	4/11/2023 16:00	18	19.0	19.5	0.5
2A	4/11/2023 17:00	18	19.0	19.5	0.5
2A	4/11/2023 18:00	18	19.0	19.5	0.5
2A	4/11/2023 19:00	18	19.0	19.5	0.5
2A	4/11/2023 20:00	18	19.0	16.7	-2.3
2A	4/11/2023 21:00	17	19.0	16.7	-2.3
2A	4/11/2023 22:00	17	19.0	16.7	-2.3
2A	4/11/2023 23:00	17	19.0	16.7	-2.3
2A	4/12/2023 0:00	17	19.0	17.2	-1.8
2A	4/12/2023 1:00	17	19.0	17.2	-1.8
2A	4/12/2023 2:00	17	19.0	17.2	-1.8
2A	4/12/2023 3:00	17	19.0	17.2	-1.8
2A	4/12/2023 4:00	17	19.0	17.2	-1.8
2A	4/12/2023 5:00	17	19.0	17.2	-1.8
2A	4/12/2023 6:00	17	19.0	17.2	-1.8
2A	4/12/2023 7:00	17	19.0	17.2	-1.8
2A	4/12/2023 8:00	17	19.0	18.4	-0.6
2A	4/12/2023 9:00	17	19.0	18.4	-0.6
2A	4/12/2023 10:00	17	19.0	18.4	-0.6
2A	4/12/2023 11:00	17	19.0	18.4	-0.6
2A	4/12/2023 12:00	17	19.0	15.7	-3.3

2A	4/12/2023 13:00	17	19.0	15.7	-3.3
2A	4/12/2023 14:00	17	19.0	15.7	-3.3
2A	4/12/2023 15:00	17	19.0	15.7	-3.3
2A	4/12/2023 16:00	17	19.0	16.7	-2.3
2A	4/12/2023 17:00	17	19.0	16.7	-2.3
2A	4/12/2023 18:00	17	19.0	16.7	-2.3
2A	4/12/2023 19:00	17	19.0	16.7	-2.3
2A	4/12/2023 20:00	17	19.0	19.5	0.5
2A	4/12/2023 21:00	17	19.0	19.5	0.5
2A	4/12/2023 22:00	17	19.0	19.5	0.5
2A	4/12/2023 23:00	17	19.0	19.5	0.5
2A	4/13/2023 0:00	17	19.0	20.1	1.1
2A	4/13/2023 1:00	17	19.0	20.1	1.1
2A	4/13/2023 2:00	18	19.0	20.1	1.1
2A	4/13/2023 3:00	18	19.0	20.1	1.1
2A	4/13/2023 4:00	18	19.0	20.1	1.1
2A	4/13/2023 5:00	18	19.0	20.1	1.1
2A	4/13/2023 6:00	18	19.0	20.1	1.1
2A	4/13/2023 7:00	18	19.0	20.1	1.1
2A	4/13/2023 8:00	18	19.0	16.8	-2.2
2A	4/13/2023 9:00	18	19.0	16.8	-2.2
2A	4/13/2023 10:00	18	19.0	16.8	-2.2
2A	4/13/2023 11:00	15	18.0	16.8	-1.2
2A	4/13/2023 12:00	15	18.0	18.4	0.4
2A	4/13/2023 13:00	15	18.0	18.4	0.4
2A	4/13/2023 14:00	15	18.0	18.4	0.4
2A	4/13/2023 15:00	15	18.0	18.4	0.4
2A	4/13/2023 16:00	15	18.0	16.7	-1.3
2A	4/13/2023 17:00	15	18.0	16.7	-1.3
2A	4/13/2023 18:00	15	18.0	16.7	-1.3
2A	4/13/2023 19:00	15	18.0	16.7	-1.3
2A	4/13/2023 20:00	15	18.0	19.5	1.5
2A	4/13/2023 21:00	16	18.0	19.5	1.5
2A	4/13/2023 22:00	16	18.0	19.5	1.5
2A	4/13/2023 23:00	16	18.0	19.5	1.5
2A	4/14/2023 0:00	16	18.0	20.1	2.1
2A	4/14/2023 1:00	17	18.0	17.2	-0.8
2A	4/14/2023 2:00	17	18.0	17.2	-0.8
2A	4/14/2023 3:00	17	18.0	17.2	-0.8
2A	4/14/2023 4:00	17	18.0	17.2	-0.8
2A	4/14/2023 5:00	17	18.0	17.2	-0.8
2A	4/14/2023 6:00	17	18.0	17.2	-0.8
2A	4/14/2023 7:00	17	18.0	17.2	-0.8
2A	4/14/2023 8:00	17	18.0	12.6	-5.4
2A	4/14/2023 9:00	17	18.0	12.6	-5.4
2A	4/14/2023 10:00	17	18.0	12.6	-5.4
2A	4/14/2023 11:00	17	18.0	12.6	-5.4

2A	4/14/2023 12:00	17	18.0	14.7	-3.3
2A	4/14/2023 13:00	17	18.0	14.7	-3.3
2A	4/14/2023 14:00	16	18.0	14.7	-3.3
2A	4/14/2023 15:00	16	18.0	14.7	-3.3
2A	4/14/2023 16:00	16	18.0	13.9	-4.1
2A	4/14/2023 17:00	16	18.0	13.9	-4.1
2A	4/14/2023 18:00	16	18.0	13.9	-4.1
2A	4/14/2023 19:00	16	18.0	13.9	-4.1
2A	4/14/2023 20:00	18	19.0	16.7	-2.3
2A	4/14/2023 21:00	18	19.0	16.7	-2.3
2A	4/14/2023 22:00	18	19.0	16.7	-2.3
2A	4/14/2023 23:00	18	19.0	16.7	-2.3
2A	4/15/2023 0:00	18	19.0	17.2	-1.8
2A	4/15/2023 1:00	18	19.0	17.2	-1.8
2A	4/15/2023 2:00	18	19.0	17.2	-1.8
2A	4/15/2023 3:00	19	19.0	17.2	-1.8
2A	4/15/2023 4:00	19	19.0	17.2	-1.8
2A	4/15/2023 5:00	19	19.0	17.2	-1.8
2A	4/15/2023 6:00	19	19.0	17.2	-1.8
2A	4/15/2023 7:00	19	19.0	17.2	-1.8
2A	4/15/2023 8:00	19	19.0	14.7	-4.3
2A	4/15/2023 9:00	19	19.0	14.7	-4.3
2A	4/15/2023 10:00	19	19.0	14.7	-4.3
2A	4/15/2023 11:00	19	19.0	14.7	-4.3
2A	4/15/2023 12:00	19	19.0	16.8	-2.2
2A	4/15/2023 13:00	19	19.0	16.8	-2.2
2A	4/15/2023 14:00	19	19.0	16.8	-2.2
2A	4/15/2023 15:00	17	19.0	16.8	-2.2
2A	4/15/2023 16:00	17	19.0	16.7	-2.3
2A	4/15/2023 17:00	17	19.0	16.7	-2.3
2A	4/15/2023 18:00	17	19.0	16.7	-2.3
2A	4/15/2023 19:00	16	18.0	16.7	-1.3
2A	4/15/2023 20:00	16	18.0	16.7	-1.3
2A	4/15/2023 21:00	16	18.0	16.7	-1.3
2A	4/15/2023 22:00	16	18.0	16.7	-1.3
2A	4/15/2023 23:00	16	18.0	16.7	-1.3
2A	4/16/2023 0:00	16	18.0	13.8	-4.2
2A	4/16/2023 1:00	16	18.0	13.8	-4.2
2A	4/16/2023 2:00	16	18.0	13.8	-4.2
2A	4/16/2023 3:00	16	18.0	13.8	-4.2
2A	4/16/2023 4:00	16	18.0	13.8	-4.2
2A	4/16/2023 5:00	16	18.0	13.8	-4.2
2A	4/16/2023 6:00	16	18.0	13.8	-4.2
2A	4/16/2023 7:00	16	19.0	13.8	-5.2
2A	4/16/2023 8:00	16	19.0	14.7	-4.3
2A	4/16/2023 9:00	16	19.0	14.7	-4.3
2A	4/16/2023 10:00	16	19.0	14.7	-4.3

2A	4/16/2023 11:00	16	19.0	14.7	-4.3
2A	4/16/2023 12:00	16	19.0	18.9	-0.1
2A	4/16/2023 13:00	16	19.0	18.9	-0.1
2A	4/16/2023 14:00	16	19.0	18.9	-0.1
2A	4/16/2023 15:00	16	19.0	18.9	-0.1
2A	4/16/2023 16:00	16	19.0	16.7	-2.3
2A	4/16/2023 17:00	16	19.0	16.7	-2.3
2A	4/16/2023 18:00	16	19.0	16.7	-2.3
2A	4/16/2023 19:00	17	19.0	16.7	-2.3
2A	4/16/2023 20:00	17	19.0	17.8	-1.2
2A	4/16/2023 21:00	17	19.0	17.8	-1.2
2A	4/16/2023 22:00	17	19.0	17.8	-1.2
2A	4/16/2023 23:00	17	19.0	17.8	-1.2
2A	4/17/2023 0:00	17	19.0	16.1	-2.9
2A	4/17/2023 1:00	17	19.0	16.1	-2.9
2A	4/17/2023 2:00	18	19.0	16.1	-2.9
2A	4/17/2023 3:00	18	19.0	16.1	-2.9
2A	4/17/2023 4:00	18	19.0	16.1	-2.9
2A	4/17/2023 5:00	18	19.0	16.1	-2.9
2A	4/17/2023 6:00	18	19.0	16.1	-2.9
2A	4/17/2023 7:00	18	19.0	16.1	-2.9
2A	4/17/2023 8:00	18	19.0	16.8	-2.2
2A	4/17/2023 9:00	17	19.0	16.8	-2.2
2A	4/17/2023 10:00	17	19.0	16.8	-2.2
2A	4/17/2023 11:00	17	19.0	16.8	-2.2
2A	4/17/2023 12:00	17	19.0	21.0	2.0
2A	4/17/2023 13:00	17	19.0	21.0	2.0
2A	4/17/2023 14:00	17	19.0	21.0	2.0
2A	4/17/2023 15:00	17	19.0	21.0	2.0
2A	4/17/2023 16:00	17	19.0	16.7	-2.3
2A	4/17/2023 17:00	17	19.0	16.7	-2.3
2A	4/17/2023 18:00	15	19.0	16.7	-2.3
2A	4/17/2023 19:00	15	19.0	16.7	-2.3
2A	4/17/2023 20:00	15	19.0	13.9	-5.1
2A	4/17/2023 21:00	15	19.0	13.9	-5.1
2A	4/17/2023 22:00	15	19.0	13.9	-5.1
2A	4/17/2023 23:00	15	19.0	13.9	-5.1
2A	4/18/2023 0:00	17	18.0	11.5	-6.5
2A	4/18/2023 1:00	17	18.0	11.5	-6.5
2A	4/18/2023 2:00	17	18.0	11.5	-6.5
2A	4/18/2023 3:00	17	18.0	11.5	-6.5
2A	4/18/2023 4:00	17	18.0	11.5	-6.5
2A	4/18/2023 5:00	17	18.0	11.5	-6.5
2A	4/18/2023 6:00	17	18.0	11.5	-6.5
2A	4/18/2023 7:00	17	18.0	11.5	-6.5
2A	4/18/2023 8:00	17	18.0	18.4	0.4
2A	4/18/2023 9:00	17	18.0	18.4	0.4

2A	4/18/2023 10:00	17	18.0	18.4	0.4
2A	4/18/2023 11:00	17	18.0	18.4	0.4
2A	4/18/2023 12:00	17	18.0	21.0	3.0
2A	4/18/2023 13:00	17	18.0	21.0	3.0
2A	4/18/2023 14:00	17	18.0	21.0	3.0
2A	4/18/2023 15:00	17	18.0	21.0	3.0
2A	4/18/2023 16:00	17	18.0	16.7	-1.3
2A	4/18/2023 17:00	17	18.0	18.8	0.8
2A	4/18/2023 18:00	17	18.0	19.5	1.5
2A	4/18/2023 19:00	17	18.0	19.5	1.5
2A	4/18/2023 20:00	17	19.0	19.5	0.5
2A	4/18/2023 21:00	18	19.0	17.8	-1.2
2A	4/18/2023 22:00	18	19.0	19.5	0.5
2A	4/18/2023 23:00	18	19.0	19.5	0.5
2A	4/19/2023 0:00	18	19.0	17.2	-1.8
2A	4/19/2023 1:00	18	19.0	17.2	-1.8
2A	4/19/2023 2:00	18	19.0	17.2	-1.8
2A	4/19/2023 3:00	18	19.0	17.2	-1.8
2A	4/19/2023 4:00	18	19.0	17.2	-1.8
2A	4/19/2023 5:00	18	19.0	17.2	-1.8
2A	4/19/2023 6:00	18	19.0	17.2	-1.8
2A	4/19/2023 7:00	18	19.0	17.2	-1.8
2A	4/19/2023 8:00	19	19.0	12.6	-6.4
2A	4/19/2023 9:00	19	19.0	12.6	-6.4
2A	4/19/2023 10:00	19	19.0	12.6	-6.4
2A	4/19/2023 11:00	19	19.0	12.6	-6.4
2A	4/19/2023 12:00	19	19.0	15.7	-3.3
2A	4/19/2023 13:00	19	19.0	15.7	-3.3
2A	4/19/2023 14:00	19	19.0	15.7	-3.3
2A	4/19/2023 15:00	19	19.0	15.7	-3.3
2A	4/19/2023 16:00	13	19.0	16.7	-2.3
2A	4/19/2023 17:00	13	19.0	16.7	-2.3
2A	4/19/2023 18:00	13	19.0	16.7	-2.3
2A	4/19/2023 19:00	16	19.0	16.7	-2.3
2A	4/19/2023 20:00	16	19.0	19.5	0.5
2A	4/19/2023 21:00	16	19.0	19.5	0.5
2A	4/19/2023 22:00	16	19.0	19.5	0.5
2A	4/19/2023 23:00	16	19.0	19.5	0.5
2A	4/20/2023 0:00	18	19.0	17.2	-1.8
2A	4/20/2023 1:00	18	19.0	17.2	-1.8
2A	4/20/2023 2:00	18	19.0	17.2	-1.8
2A	4/20/2023 3:00	18	19.0	17.2	-1.8
2A	4/20/2023 4:00	18	19.0	17.2	-1.8
2A	4/20/2023 5:00	18	19.0	17.2	-1.8
2A	4/20/2023 6:00	18	19.0	17.2	-1.8
2A	4/20/2023 7:00	18	19.0	17.2	-1.8
2A	4/20/2023 8:00	18	19.0	16.8	-2.2

2A	4/20/2023 9:00	18	19.0	16.8	-2.2
2A	4/20/2023 10:00	18	19.0	16.8	-2.2
2A	4/20/2023 11:00	18	19.0	16.8	-2.2
2A	4/20/2023 12:00	18	19.0	16.8	-2.2
2A	4/20/2023 13:00	18	19.0	16.8	-2.2
2A	4/20/2023 14:00	18	19.0	16.8	-2.2
2A	4/20/2023 15:00	15	19.0	15.2	-3.8
2A	4/20/2023 16:00	15	19.0	13.4	-5.6
2A	4/20/2023 17:00	15	19.0	13.4	-5.6
2A	4/20/2023 18:00	15	19.0	13.4	-5.6
2A	4/20/2023 19:00	15	19.0	13.9	-5.1
2A	4/20/2023 20:00	15	19.0	16.7	-2.3
2A	4/20/2023 21:00	16	19.0	16.7	-2.3
2A	4/20/2023 22:00	16	19.0	16.7	-2.3
2A	4/20/2023 23:00	16	19.0	16.7	-2.3
2A	4/21/2023 0:00	16	19.0	16.1	-2.9
2A	4/21/2023 1:00	16	19.0	16.1	-2.9
2A	4/21/2023 2:00	16	19.0	16.1	-2.9
2A	4/21/2023 3:00	16	19.0	16.1	-2.9
2A	4/21/2023 4:00	16	19.0	16.1	-2.9
2A	4/21/2023 5:00	16	19.0	16.1	-2.9
2A	4/21/2023 6:00	16	19.0	16.1	-2.9
2A	4/21/2023 7:00	16	19.0	16.1	-2.9
2A	4/21/2023 8:00	16	19.0	15.7	-3.3
2A	4/21/2023 9:00	16	19.0	15.7	-3.3
2A	4/21/2023 10:00	16	19.0	15.7	-3.3
2A	4/21/2023 11:00	16	19.0	15.7	-3.3
2A	4/21/2023 12:00	16	19.0	18.4	-0.6
2A	4/21/2023 13:00	16	19.0	18.4	-0.6
2A	4/21/2023 14:00	16	19.0	18.4	-0.6
2A	4/21/2023 15:00	16	19.0	16.4	-2.6
2A	4/21/2023 16:00	16	19.0	16.7	-2.3
2A	4/21/2023 17:00	16	19.0	16.7	-2.3
2A	4/21/2023 18:00	16	19.0	16.7	-2.3
2A	4/21/2023 19:00	16	19.0	16.7	-2.3
2A	4/21/2023 20:00	16	19.0	15.6	-3.4
2A	4/21/2023 21:00	18	19.0	16.7	-2.3
2A	4/21/2023 22:00	18	19.0	16.7	-2.3
2A	4/21/2023 23:00	18	19.0	16.7	-2.3
2A	4/22/2023 0:00	18	19.0	17.2	-1.8
2A	4/22/2023 1:00	18	19.0	17.2	-1.8
2A	4/22/2023 2:00	18	19.0	17.2	-1.8
2A	4/22/2023 3:00	18	19.0	17.2	-1.8
2A	4/22/2023 4:00	18	19.0	17.2	-1.8
2A	4/22/2023 5:00	18	19.0	17.2	-1.8
2A	4/22/2023 6:00	18	19.0	17.2	-1.8
2A	4/22/2023 7:00	18	19.0	17.2	-1.8

2A	4/22/2023 8:00	18	19.0	18.4	-0.6
2A	4/22/2023 9:00	18	19.0	18.4	-0.6
2A	4/22/2023 10:00	18	19.0	18.4	-0.6
2A	4/22/2023 11:00	18	19.0	18.4	-0.6
2A	4/22/2023 12:00	18	19.0	21.0	2.0
2A	4/22/2023 13:00	18	19.0	21.0	2.0
2A	4/22/2023 14:00	18	19.0	21.0	2.0
2A	4/22/2023 15:00	17	17.0	21.0	4.0
2A	4/22/2023 16:00	18	19.0	19.5	0.5
2A	4/22/2023 17:00	18	19.0	19.5	0.5
2A	4/22/2023 18:00	18	19.0	19.5	0.5
2A	4/22/2023 19:00	17	17.0	19.5	2.5
2A	4/22/2023 20:00	16	17.0	17.8	0.8
2A	4/22/2023 21:00	16	17.0	17.8	0.8
2A	4/22/2023 22:00	16	17.0	17.8	0.8
2A	4/22/2023 23:00	16	17.0	17.8	0.8
2A	4/23/2023 0:00	16	17.0	16.1	-0.9
2A	4/23/2023 1:00	16	17.0	16.1	-0.9
2A	4/23/2023 2:00	16	17.0	16.1	-0.9
2A	4/23/2023 3:00	16	17.0	16.1	-0.9
2A	4/23/2023 4:00	16	17.0	16.1	-0.9
2A	4/23/2023 5:00	16	17.0	16.1	-0.9
2A	4/23/2023 6:00	17	17.0	16.1	-0.9
2A	4/23/2023 7:00	17	17.0	16.1	-0.9
2A	4/23/2023 8:00	17	17.0	18.4	1.4
2A	4/23/2023 9:00	17	17.0	18.4	1.4
2A	4/23/2023 10:00	17	17.0	18.4	1.4
2A	4/23/2023 11:00	17	17.0	18.4	1.4
2A	4/23/2023 12:00	17	17.0	21.0	4.0
2A	4/23/2023 13:00	17	17.0	21.0	4.0
2A	4/23/2023 14:00	17	17.0	21.0	4.0
2A	4/23/2023 15:00	17	17.0	21.0	4.0
2A	4/23/2023 16:00	15	17.0	16.7	-0.3
2A	4/23/2023 17:00	15	17.0	16.7	-0.3
2A	4/23/2023 18:00	15	17.0	16.7	-0.3
2A	4/23/2023 19:00	15	17.0	16.7	-0.3
2A	4/23/2023 20:00	15	17.0	17.8	0.8
2A	4/23/2023 21:00	15	17.0	17.8	0.8
2A	4/23/2023 22:00	15	17.0	17.8	0.8
2A	4/23/2023 23:00	15	17.0	17.8	0.8
2A	4/24/2023 0:00	15	17.0	17.2	0.2
2A	4/24/2023 1:00	15	17.0	17.2	0.2
2A	4/24/2023 2:00	15	17.0	17.2	0.2
2A	4/24/2023 3:00	15	17.0	17.2	0.2
2A	4/24/2023 4:00	15	17.0	17.2	0.2
2A	4/24/2023 5:00	15	17.0	17.2	0.2
2A	4/24/2023 6:00	15	17.0	17.2	0.2

2A	4/24/2023 7:00	15	17.0	17.2	0.2
2A	4/24/2023 8:00	15	17.0	18.4	1.4
2A	4/24/2023 9:00	15	17.0	18.4	1.4
2A	4/24/2023 10:00	15	17.0	18.4	1.4
2A	4/24/2023 11:00	15	17.0	18.4	1.4
2A	4/24/2023 12:00	15	17.0	21.0	4.0
2A	4/24/2023 13:00	15	17.0	21.0	4.0
2A	4/24/2023 14:00	15	17.0	21.0	4.0
2A	4/24/2023 15:00	17	17.0	18.9	1.9
2A	4/24/2023 16:00	16	15.0	15.6	0.6
2A	4/24/2023 17:00	16	15.0	15.6	0.6
2A	4/24/2023 18:00	16	15.0	15.6	0.6
2A	4/24/2023 19:00	15	16.0	15.6	-0.4
2A	4/24/2023 20:00	15	16.0	13.4	-2.6
2A	4/24/2023 21:00	15	16.0	13.4	-2.6
2A	4/24/2023 22:00	15	16.0	13.4	-2.6
2A	4/24/2023 23:00	15	16.0	13.4	-2.6
2A	4/25/2023 0:00	15	17.0	9.2	-7.8
2A	4/25/2023 1:00	15	17.0	9.2	-7.8
2A	4/25/2023 2:00	16	16.0	7.5	-8.5
2A	4/25/2023 3:00	16	16.0	9.2	-6.8
2A	4/25/2023 4:00	16	16.0	9.2	-6.8
2A	4/25/2023 5:00	16	16.0	9.2	-6.8
2A	4/25/2023 6:00	16	16.0	9.2	-6.8
2A	4/25/2023 7:00	16	16.0	9.2	-6.8
2A	4/25/2023 8:00	16	16.0	14.7	-1.3
2A	4/25/2023 9:00	16	16.0	14.7	-1.3
2A	4/25/2023 10:00	16	16.0	15.7	-0.3
2A	4/25/2023 11:00	16	16.0	15.7	-0.3
2A	4/25/2023 12:00	16	16.0	18.4	2.4
2A	4/25/2023 13:00	16	16.0	18.4	2.4
2A	4/25/2023 14:00	16	16.0	18.4	2.4
2A	4/25/2023 15:00	13	19.0	18.4	-0.6
2A	4/25/2023 16:00	13	19.0	13.9	-5.1
2A	4/25/2023 17:00	13	19.0	13.9	-5.1
2A	4/25/2023 18:00	13	19.0	13.9	-5.1
2A	4/25/2023 19:00	13	19.0	13.9	-5.1
2A	4/25/2023 20:00	15	19.0	16.7	-2.3
2A	4/25/2023 21:00	15	19.0	16.7	-2.3
2A	4/25/2023 22:00	15	19.0	16.7	-2.3
2A	4/25/2023 23:00	15	19.0	16.7	-2.3
2A	4/26/2023 0:00	15	19.0	17.2	-1.8
2A	4/26/2023 1:00	15	19.0	17.2	-1.8
2A	4/26/2023 2:00	15	19.0	17.2	-1.8
2A	4/26/2023 3:00	15	19.0	17.2	-1.8
2A	4/26/2023 4:00	15	19.0	17.2	-1.8
2A	4/26/2023 5:00	15	19.0	17.2	-1.8

2A	4/26/2023 6:00	17	19.0	17.2	-1.8
2A	4/26/2023 7:00	17	19.0	17.2	-1.8
2A	4/26/2023 8:00	17	19.0	13.1	-5.9
2A	4/26/2023 9:00	17	19.0	13.1	-5.9
2A	4/26/2023 10:00	17	19.0	13.1	-5.9
2A	4/26/2023 11:00	17	19.0	13.1	-5.9
2A	4/26/2023 12:00	17	19.0	13.1	-5.9
2A	4/26/2023 13:00	17	19.0	13.1	-5.9
2A	4/26/2023 14:00	17	19.0	13.1	-5.9
2A	4/26/2023 15:00	17	19.0	13.1	-5.9
2A	4/26/2023 16:00	17	19.0	13.9	-5.1
2A	4/26/2023 17:00	17	19.0	13.9	-5.1
2A	4/26/2023 18:00	17	19.0	13.9	-5.1
2A	4/26/2023 19:00	17	19.0	13.9	-5.1
2A	4/26/2023 20:00	19	15.0	13.9	-1.1
2A	4/26/2023 21:00	19	15.0	13.9	-1.1
2A	4/26/2023 22:00	19	15.0	13.9	-1.1
2A	4/26/2023 23:00	19	15.0	13.9	-1.1
2A	4/27/2023 0:00	18	17.0	13.8	-3.2
2A	4/27/2023 1:00	18	17.0	13.8	-3.2
2A	4/27/2023 2:00	18	17.0	13.8	-3.2
2A	4/27/2023 3:00	18	17.0	13.8	-3.2
2A	4/27/2023 4:00	18	17.0	13.8	-3.2
2A	4/27/2023 5:00	18	17.0	13.8	-3.2
2A	4/27/2023 6:00	18	17.0	13.8	-3.2
2A	4/27/2023 7:00	18	17.0	13.8	-3.2
2A	4/27/2023 8:00	17	18.0	16.8	-1.2
2A	4/27/2023 9:00	17	18.0	16.8	-1.2
2A	4/27/2023 10:00	17	18.0	16.8	-1.2
2A	4/27/2023 11:00	17	18.0	16.8	-1.2
2A	4/27/2023 12:00	17	18.0	18.4	0.4
2A	4/27/2023 13:00	17	18.0	18.4	0.4
2A	4/27/2023 14:00	17	18.0	18.4	0.4
2A	4/27/2023 15:00	17	18.0	18.4	0.4
2A	4/27/2023 16:00	17	18.0	19.5	1.5
2A	4/27/2023 17:00	17	18.0	19.5	1.5
2A	4/27/2023 18:00	17	18.0	19.5	1.5
2A	4/27/2023 19:00	16	19.0	19.5	0.5
2A	4/27/2023 20:00	16	19.0	16.7	-2.3
2A	4/27/2023 21:00	16	19.0	16.7	-2.3
2A	4/27/2023 22:00	16	19.0	16.7	-2.3
2A	4/27/2023 23:00	16	19.0	16.7	-2.3
2A	4/28/2023 0:00	16	19.0	14.4	-4.7
2A	4/28/2023 1:00	16	19.0	14.4	-4.7
2A	4/28/2023 2:00	16	19.0	14.4	-4.7
2A	4/28/2023 3:00	16	19.0	14.4	-4.7
2A	4/28/2023 4:00	16	19.0	14.4	-4.7

2A	4/28/2023 5:00	16	19.0	14.4	-4.7
2A	4/28/2023 6:00	16	19.0	14.4	-4.7
2A	4/28/2023 7:00	16	19.0	14.4	-4.7
2A	4/28/2023 8:00	16	19.0	13.1	-5.9
2A	4/28/2023 9:00	16	19.0	13.1	-5.9
2A	4/28/2023 10:00	16	19.0	13.1	-5.9
2A	4/28/2023 11:00	16	19.0	13.1	-5.9
2A	4/28/2023 12:00	16	19.0	13.1	-5.9
2A	4/28/2023 13:00	16	19.0	13.1	-5.9
2A	4/28/2023 14:00	16	19.0	13.1	-5.9
2A	4/28/2023 15:00	16	19.0	13.1	-5.9
2A	4/28/2023 16:00	16	19.0	11.1	-7.9
2A	4/28/2023 17:00	16	19.0	11.1	-7.9
2A	4/28/2023 18:00	16	19.0	11.1	-7.9
2A	4/28/2023 19:00	17	19.0	11.1	-7.9
2A	4/28/2023 20:00	17	19.0	15.6	-3.4
2A	4/28/2023 21:00	17	19.0	15.6	-3.4
2A	4/28/2023 22:00	17	19.0	15.6	-3.4
2A	4/28/2023 23:00	17	19.0	15.6	-3.4
2A	4/29/2023 0:00	17	19.0	16.1	-2.9
2A	4/29/2023 1:00	17	19.0	16.1	-2.9
2A	4/29/2023 2:00	18	19.0	16.1	-2.9
2A	4/29/2023 3:00	18	19.0	16.1	-2.9
2A	4/29/2023 4:00	18	19.0	16.1	-2.9
2A	4/29/2023 5:00	18	19.0	16.1	-2.9
2A	4/29/2023 6:00	18	19.0	16.1	-2.9
2A	4/29/2023 7:00	18	19.0	16.1	-2.9
2A	4/29/2023 8:00	18	19.0	18.4	-0.6
2A	4/29/2023 9:00	18	19.0	18.4	-0.6
2A	4/29/2023 10:00	18	19.0	18.4	-0.6
2A	4/29/2023 11:00	18	19.0	18.4	-0.6
2A	4/29/2023 12:00	18	19.0	19.7	0.7
2A	4/29/2023 13:00	18	19.0	21.0	2.0
2A	4/29/2023 14:00	18	19.0	21.0	2.0
2A	4/29/2023 15:00	18	19.0	21.0	2.0
2A	4/29/2023 16:00	18	19.0	16.7	-2.3
2A	4/29/2023 17:00	18	19.0	16.7	-2.3
2A	4/29/2023 18:00	18	19.0	16.7	-2.3
2A	4/29/2023 19:00	18	19.0	16.7	-2.3
2A	4/29/2023 20:00	18	19.0	19.5	0.5
2A	4/29/2023 21:00	18	19.0	19.5	0.5
2A	4/29/2023 22:00	18	19.0	19.5	0.5
2A	4/29/2023 23:00	18	19.0	19.5	0.5
2A	4/30/2023 0:00	18	19.0	18.4	-0.6
2A	4/30/2023 1:00	19	19.0	18.4	-0.6
2A	4/30/2023 2:00	19	19.0	18.4	-0.6
2A	4/30/2023 3:00	19	19.0	18.4	-0.6

2A	4/30/2023 4:00	19	19.0	18.4	-0.6
2A	4/30/2023 5:00	19	19.0	18.4	-0.6
2A	4/30/2023 6:00	19	19.0	18.4	-0.6
2A	4/30/2023 7:00	19	19.0	18.4	-0.6
2A	4/30/2023 8:00	19	19.0	16.8	-2.2
2A	4/30/2023 9:00	19	19.0	16.8	-2.2
2A	4/30/2023 10:00	19	19.0	16.8	-2.2
2A	4/30/2023 11:00	19	19.0	16.8	-2.2
2A	4/30/2023 12:00	19	19.0	18.9	-0.1
2A	4/30/2023 13:00	19	19.0	18.9	-0.1
2A	4/30/2023 14:00	19	19.0	18.9	-0.1
2A	4/30/2023 15:00	19	19.0	18.9	-0.1
2A	4/30/2023 16:00	19	19.0	16.7	-2.3
2A	4/30/2023 17:00	19	19.0	16.7	-2.3
2A	4/30/2023 18:00	19	19.0	16.7	-2.3
2A	4/30/2023 19:00	19	19.0	16.7	-2.3
2A	4/30/2023 20:00	17	19.0	19.5	0.5
2A	4/30/2023 21:00	17	19.0	19.5	0.5
2A	4/30/2023 22:00	17	19.0	19.5	0.5
2A	4/30/2023 23:00	17	19.0	18.1	-0.9
2A	5/1/2023 0:00	17	19.0	17.2	-1.8
2A	5/1/2023 1:00	17	19.0	17.2	-1.8
2A	5/1/2023 2:00	17	19.0	17.2	-1.8
2A	5/1/2023 3:00	17	19.0	17.2	-1.8
2A	5/1/2023 4:00	17	19.0	17.2	-1.8
2A	5/1/2023 5:00	17	19.0	17.2	-1.8
2A	5/1/2023 6:00	17	19.0	17.2	-1.8
2A	5/1/2023 7:00	17	19.0	17.2	-1.8
2A	5/1/2023 8:00	17	19.0	13.1	-5.9
2A	5/1/2023 9:00	17	19.0	13.1	-5.9
2A	5/1/2023 10:00	17	19.0	13.1	-5.9
2A	5/1/2023 11:00	17	19.0	13.1	-5.9
2A	5/1/2023 12:00	17	19.0	15.7	-3.3
2A	5/1/2023 13:00	17	19.0	15.7	-3.3
2A	5/1/2023 14:00	17	19.0	15.7	-3.3
2A	5/1/2023 15:00	17	19.0	15.7	-3.3
2A	5/1/2023 16:00	17	19.0	16.7	-2.3
2A	5/1/2023 17:00	18	19.0	16.7	-2.3
2A	5/1/2023 18:00	18	19.0	16.7	-2.3
2A	5/1/2023 19:00	18	19.0	16.7	-2.3
2A	5/1/2023 20:00	18	19.0	19.5	0.5
2A	5/1/2023 21:00	18	19.0	19.5	0.5
2A	5/1/2023 22:00	19	19.0	19.5	0.5
2A	5/1/2023 23:00	19	19.0	19.5	0.5
2A	5/2/2023 0:00	19	19.0	17.2	-1.8
2A	5/2/2023 1:00	19	19.0	17.2	-1.8
2A	5/2/2023 2:00	19	19.0	17.2	-1.8

2A	5/2/2023 3:00	19	19.0	17.2	-1.8
2A	5/2/2023 4:00	19	19.0	17.2	-1.8
2A	5/2/2023 5:00	19	19.0	17.2	-1.8
2A	5/2/2023 6:00	19	19.0	17.2	-1.8
2A	5/2/2023 7:00	19	19.0	17.2	-1.8
2A	5/2/2023 8:00	19	19.0	15.7	-3.3
2A	5/2/2023 9:00	19	19.0	15.7	-3.3
2A	5/2/2023 10:00	19	19.0	15.7	-3.3
2A	5/2/2023 11:00	19	19.0	15.7	-3.3
2A	5/2/2023 12:00	19	19.0	15.7	-3.3
2A	5/2/2023 13:00	19	19.0	15.7	-3.3
2A	5/2/2023 14:00	19	19.0	15.7	-3.3
2A	5/2/2023 15:00	19	19.0	15.7	-3.3
2A	5/2/2023 16:00	19	19.0	16.7	-2.3
2A	5/2/2023 17:00	19	19.0	16.7	-2.3
2A	5/2/2023 18:00	19	19.0	16.7	-2.3
2A	5/2/2023 19:00	19	19.0	16.7	-2.3
2A	5/2/2023 20:00	19	19.0	16.7	-2.3
2A	5/2/2023 21:00	19	19.0	16.7	-2.3
2A	5/2/2023 22:00	19	19.0	16.7	-2.3
2A	5/2/2023 23:00	19	19.0	16.7	-2.3
2A	5/3/2023 0:00	19	19.0	17.2	-1.8
2A	5/3/2023 1:00	19	19.0	17.2	-1.8
2A	5/3/2023 2:00	19	19.0	17.2	-1.8
2A	5/3/2023 3:00	19	19.0	17.2	-1.8
2A	5/3/2023 4:00	19	19.0	17.2	-1.8
2A	5/3/2023 5:00	19	19.0	17.2	-1.8
2A	5/3/2023 6:00	19	19.0	17.2	-1.8
2A	5/3/2023 7:00	19	19.0	17.2	-1.8
2A	5/3/2023 8:00	19	19.0	13.1	-5.9
2A	5/3/2023 9:00	19	19.0	13.1	-5.9
2A	5/3/2023 10:00	19	19.0	13.1	-5.9
2A	5/3/2023 11:00	19	19.0	13.1	-5.9
2A	5/3/2023 12:00	19	19.0	11.5	-7.5
2A	5/3/2023 13:00	19	19.0	13.1	-5.9
2A	5/3/2023 14:00	19	19.0	13.1	-5.9
2A	5/3/2023 15:00	19	19.0	13.1	-5.9
2A	5/3/2023 16:00	19	19.0	13.9	-5.1
2A	5/3/2023 17:00	19	19.0	13.9	-5.1
2A	5/3/2023 18:00	19	19.0	13.9	-5.1
2A	5/3/2023 19:00	18	19.0	13.9	-5.1
2A	5/3/2023 20:00	18	19.0	19.5	0.5
2A	5/3/2023 21:00	18	19.0	19.5	0.5
2A	5/3/2023 22:00	18	19.0	19.5	0.5
2A	5/3/2023 23:00	18	19.0	19.5	0.5
2A	5/4/2023 0:00	18	19.0	17.2	-1.8
2A	5/4/2023 1:00	18	19.0	17.2	-1.8

2A	5/4/2023 2:00	18	19.0	17.2	-1.8
2A	5/4/2023 3:00	18	19.0	17.2	-1.8
2A	5/4/2023 4:00	19	19.0	17.2	-1.8
2A	5/4/2023 5:00	19	19.0	20.1	1.1
2A	5/4/2023 6:00	19	19.0	20.1	1.1
2A	5/4/2023 7:00	19	19.0	20.1	1.1
2A	5/4/2023 8:00	19	19.0	15.7	-3.3
2A	5/4/2023 9:00	19	19.0	15.7	-3.3
2A	5/4/2023 10:00	19	19.0	15.7	-3.3
2A	5/4/2023 11:00	19	19.0	15.7	-3.3
2A	5/4/2023 12:00	19	19.0	15.7	-3.3
2A	5/4/2023 13:00	19	19.0	15.7	-3.3
2A	5/4/2023 14:00	19	19.0	15.7	-3.3
2A	5/4/2023 15:00	19	19.0	15.7	-3.3
2A	5/4/2023 16:00	19	19.0	17.8	-1.2
2A	5/4/2023 17:00	19	19.0	17.8	-1.2
2A	5/4/2023 18:00	19	19.0	17.8	-1.2
2A	5/4/2023 19:00	18	19.0	15.6	-3.4
2A	5/4/2023 20:00	18	18.0	16.7	-1.3
2A	5/4/2023 21:00	18	18.0	16.7	-1.3
2A	5/4/2023 22:00	18	18.0	16.7	-1.3
2A	5/4/2023 23:00	18	18.0	16.7	-1.3
2A	5/5/2023 0:00	18	18.0	14.4	-3.7
2A	5/5/2023 1:00	18	18.0	14.4	-3.7
2A	5/5/2023 2:00	18	18.0	14.4	-3.7
2A	5/5/2023 3:00	18	18.0	14.4	-3.7
2A	5/5/2023 4:00	18	18.0	14.4	-3.7
2A	5/5/2023 5:00	18	18.0	14.4	-3.7
2A	5/5/2023 6:00	18	19.0	14.4	-4.7
2A	5/5/2023 7:00	18	19.0	14.4	-4.7
2A	5/5/2023 8:00	18	19.0	16.8	-2.2
2A	5/5/2023 9:00	18	19.0	16.8	-2.2
2A	5/5/2023 10:00	18	19.0	16.8	-2.2
2A	5/5/2023 11:00	18	19.0	16.8	-2.2
2A	5/5/2023 12:00	18	19.0	18.9	-0.1
2A	5/5/2023 13:00	18	19.0	18.9	-0.1
2A	5/5/2023 14:00	18	19.0	18.9	-0.1
2A	5/5/2023 15:00	18	19.0	18.9	-0.1
2A	5/5/2023 16:00	18	19.0	17.8	-1.2
2A	5/5/2023 17:00	18	19.0	17.8	-1.2
2A	5/5/2023 18:00	18	19.0	17.8	-1.2
2A	5/5/2023 19:00	18	19.0	17.8	-1.2
2A	5/5/2023 20:00	19	19.0	16.7	-2.3
2A	5/5/2023 21:00	19	19.0	16.7	-2.3
2A	5/5/2023 22:00	19	19.0	16.7	-2.3
2A	5/5/2023 23:00	19	19.0	16.7	-2.3
2A	5/6/2023 0:00	19	19.0	17.2	-1.8

2A	5/6/2023 1:00	19	19.0	17.2	-1.8
2A	5/6/2023 2:00	19	19.0	17.2	-1.8
2A	5/6/2023 3:00	19	19.0	17.2	-1.8
2A	5/6/2023 4:00	19	19.0	17.2	-1.8
2A	5/6/2023 5:00	19	19.0	17.2	-1.8
2A	5/6/2023 6:00	19	19.0	17.2	-1.8
2A	5/6/2023 7:00	19	19.0	17.2	-1.8
2A	5/6/2023 8:00	19	19.0	15.7	-3.3
2A	5/6/2023 9:00	19	19.0	15.7	-3.3
2A	5/6/2023 10:00	19	19.0	15.7	-3.3
2A	5/6/2023 11:00	19	19.0	15.7	-3.3
2A	5/6/2023 12:00	19	19.0	15.7	-3.3
2A	5/6/2023 13:00	19	19.0	15.7	-3.3
2A	5/6/2023 14:00	19	19.0	15.7	-3.3
2A	5/6/2023 15:00	17	19.0	18.4	-0.6
2A	5/6/2023 16:00	17	19.0	16.7	-2.3
2A	5/6/2023 17:00	17	19.0	16.7	-2.3
2A	5/6/2023 18:00	17	19.0	16.7	-2.3
2A	5/6/2023 19:00	17	19.0	13.9	-5.1
2A	5/6/2023 20:00	19	19.0	16.7	-2.3
2A	5/6/2023 21:00	19	19.0	16.7	-2.3
2A	5/6/2023 22:00	19	19.0	16.7	-2.3
2A	5/6/2023 23:00	19	19.0	16.7	-2.3
2A	5/7/2023 0:00	19	19.0	17.2	-1.8
2A	5/7/2023 1:00	19	19.0	17.2	-1.8
2A	5/7/2023 2:00	19	19.0	17.2	-1.8
2A	5/7/2023 3:00	19	19.0	17.2	-1.8
2A	5/7/2023 4:00	19	19.0	17.2	-1.8
2A	5/7/2023 5:00	19	19.0	17.2	-1.8
2A	5/7/2023 6:00	19	19.0	17.2	-1.8
2A	5/7/2023 7:00	19	19.0	17.2	-1.8
2A	5/7/2023 8:00	19	19.0	15.7	-3.3
2A	5/7/2023 9:00	19	19.0	15.7	-3.3
2A	5/7/2023 10:00	19	19.0	15.7	-3.3
2A	5/7/2023 11:00	19	19.0	15.7	-3.3
2A	5/7/2023 12:00	19	19.0	15.7	-3.3
2A	5/7/2023 13:00	19	19.0	15.7	-3.3
2A	5/7/2023 14:00	19	19.0	15.7	-3.3
2A	5/7/2023 15:00	19	19.0	15.7	-3.3
2A	5/7/2023 16:00	19	19.0	13.9	-5.1
2A	5/7/2023 17:00	19	19.0	13.9	-5.1
2A	5/7/2023 18:00	19	19.0	13.9	-5.1
2A	5/7/2023 19:00	19	19.0	13.9	-5.1
2A	5/7/2023 20:00	19	19.0	16.7	-2.3
2A	5/7/2023 21:00	18	19.0	16.7	-2.3
2A	5/7/2023 22:00	18	19.0	16.7	-2.3
2A	5/7/2023 23:00	18	19.0	16.7	-2.3

2A	5/8/2023 0:00	18	19.0	17.2	-1.8
2A	5/8/2023 1:00	18	19.0	17.2	-1.8
2A	5/8/2023 2:00	18	19.0	17.2	-1.8
2A	5/8/2023 3:00	18	19.0	17.2	-1.8
2A	5/8/2023 4:00	18	19.0	17.2	-1.8
2A	5/8/2023 5:00	18	19.0	17.2	-1.8
2A	5/8/2023 6:00	18	19.0	17.2	-1.8
2A	5/8/2023 7:00	18	19.0	17.2	-1.8
2A	5/8/2023 8:00	18	19.0	15.7	-3.3
2A	5/8/2023 9:00	18	19.0	15.7	-3.3
2A	5/8/2023 10:00	18	19.0	15.7	-3.3
2A	5/8/2023 11:00	18	19.0	15.7	-3.3
2A	5/8/2023 12:00	18	19.0	15.7	-3.3
2A	5/8/2023 13:00	18	19.0	15.7	-3.3
2A	5/8/2023 14:00	18	19.0	15.7	-3.3
2A	5/8/2023 15:00	18	19.0	15.7	-3.3
2A	5/8/2023 16:00	18	19.0	16.7	-2.3
2A	5/8/2023 17:00	18	19.0	16.7	-2.3
2A	5/8/2023 18:00	18	19.0	16.7	-2.3
2A	5/8/2023 19:00	18	19.0	16.7	-2.3
2A	5/8/2023 20:00	18	19.0	13.9	-5.1
2A	5/8/2023 21:00	18	19.0	13.9	-5.1
2A	5/8/2023 22:00	18	19.0	13.9	-5.1
2A	5/8/2023 23:00	18	19.0	13.9	-5.1
2A	5/9/2023 0:00	19	19.0	11.5	-7.5
2A	5/9/2023 1:00	19	19.0	11.5	-7.5
2A	5/9/2023 2:00	19	19.0	11.5	-7.5
2A	5/9/2023 3:00	19	19.0	11.5	-7.5
2A	5/9/2023 4:00	19	19.0	11.5	-7.5
2A	5/9/2023 5:00	19	19.0	11.5	-7.5
2A	5/9/2023 6:00	19	19.0	11.5	-7.5
2A	5/9/2023 7:00	19	19.0	11.5	-7.5
2A	5/9/2023 8:00	19	19.0	15.7	-3.3
2A	5/9/2023 9:00	19	19.0	15.7	-3.3
2A	5/9/2023 10:00	19	19.0	15.7	-3.3
2A	5/9/2023 11:00	19	19.0	15.7	-3.3
2A	5/9/2023 12:00	19	19.0	15.7	-3.3
2A	5/9/2023 13:00	19	19.0	15.7	-3.3
2A	5/9/2023 14:00	19	19.0	15.7	-3.3
2A	5/9/2023 15:00	16	19.0	15.7	-3.3
2A	5/9/2023 16:00	16	19.0	13.4	-5.6
2A	5/9/2023 17:00	16	19.0	13.4	-5.6
2A	5/9/2023 18:00	16	19.0	13.4	-5.6
2A	5/9/2023 19:00	16	19.0	13.4	-5.6
2A	5/9/2023 20:00	16	19.0	13.9	-5.1
2A	5/9/2023 21:00	16	19.0	13.9	-5.1
2A	5/9/2023 22:00	16	19.0	13.9	-5.1

2A	5/9/2023 23:00	19	19.0	13.9	-5.1
2A	5/10/2023 0:00	19	19.0	14.4	-4.7
2A	5/10/2023 1:00	19	19.0	14.4	-4.7
2A	5/10/2023 2:00	19	19.0	14.4	-4.7
2A	5/10/2023 3:00	19	19.0	14.4	-4.7
2A	5/10/2023 4:00	19	19.0	14.4	-4.7
2A	5/10/2023 5:00	19	19.0	14.4	-4.7
2A	5/10/2023 6:00	19	19.0	14.4	-4.7
2A	5/10/2023 7:00	19	19.0	14.4	-4.7
2A	5/10/2023 8:00	19	19.0	17.1	-2.0
2A	5/10/2023 9:00	19	19.0	18.4	-0.6
2A	5/10/2023 10:00	19	19.0	18.4	-0.6
2A	5/10/2023 11:00	19	19.0	17.8	-1.2
2A	5/10/2023 12:00	19	19.0	21.0	2.0
2A	5/10/2023 13:00	19	19.0	21.0	2.0
2A	5/10/2023 14:00	19	19.0	21.0	2.0
2A	5/10/2023 15:00	19	19.0	21.0	2.0
2A	5/10/2023 16:00	19	19.0	15.6	-3.4
2A	5/10/2023 17:00	19	19.0	15.6	-3.4
2A	5/10/2023 18:00	19	19.0	15.6	-3.4
2A	5/10/2023 19:00	19	19.0	15.6	-3.4
2A	5/10/2023 20:00	19	19.0	16.7	-2.3
2A	5/10/2023 21:00	19	19.0	16.7	-2.3
2A	5/10/2023 22:00	19	19.0	16.7	-2.3
2A	5/10/2023 23:00	19	19.0	18.1	-0.9
2A	5/11/2023 0:00	19	19.0	17.2	-1.8
2A	5/11/2023 1:00	19	19.0	17.2	-1.8
2A	5/11/2023 2:00	19	19.0	17.2	-1.8
2A	5/11/2023 3:00	19	19.0	17.2	-1.8
2A	5/11/2023 4:00	19	19.0	17.2	-1.8
2A	5/11/2023 5:00	19	19.0	17.2	-1.8
2A	5/11/2023 6:00	19	19.0	17.2	-1.8
2A	5/11/2023 7:00	19	19.0	17.2	-1.8
2A	5/11/2023 8:00	19	19.0	18.4	-0.6
2A	5/11/2023 9:00	19	19.0	18.4	-0.6
2A	5/11/2023 10:00	19	19.0	18.4	-0.6
2A	5/11/2023 11:00	19	19.0	18.4	-0.6
2A	5/11/2023 12:00	19	19.0	18.4	-0.6
2A	5/11/2023 13:00	19	19.0	18.4	-0.6
2A	5/11/2023 14:00	18	19.0	18.4	-0.6
2A	5/11/2023 15:00	18	19.0	18.4	-0.6
2A	5/11/2023 16:00	18	19.0	19.5	0.5
2A	5/11/2023 17:00	18	19.0	19.5	0.5
2A	5/11/2023 18:00	18	19.0	19.5	0.5
2A	5/11/2023 19:00	18	19.0	19.5	0.5
2A	5/11/2023 20:00	18	19.0	19.5	0.5
2A	5/11/2023 21:00	18	19.0	19.5	0.5

2A	5/11/2023 22:00	18	19.0	19.5	0.5
2A	5/11/2023 23:00	17	17.0	19.5	2.5
2A	5/12/2023 0:00	17	17.0	20.1	3.1
2A	5/12/2023 1:00	17	17.0	20.1	3.1
2A	5/12/2023 2:00	17	17.0	20.1	3.1
2A	5/12/2023 3:00	17	17.0	20.1	3.1
2A	5/12/2023 4:00	17	17.0	20.1	3.1
2A	5/12/2023 5:00	17	17.0	20.1	3.1
2A	5/12/2023 6:00	17	17.0	20.1	3.1
2A	5/12/2023 7:00	17	17.0	20.1	3.1
2A	5/12/2023 8:00	17	17.0	13.1	-3.9
2A	5/12/2023 9:00	17	17.0	13.1	-3.9
2A	5/12/2023 10:00	17	17.0	13.1	-3.9
2A	5/12/2023 11:00	17	17.0	13.1	-3.9
2A	5/12/2023 12:00	17	17.0	13.1	-3.9
2A	5/12/2023 13:00	17	17.0	13.1	-3.9
2A	5/12/2023 14:00	17	17.0	13.1	-3.9
2A	5/12/2023 15:00	17	17.0	13.1	-3.9
2A	5/12/2023 16:00	17	17.0	13.9	-3.1
2A	5/12/2023 17:00	17	19.0	13.9	-5.1
2A	5/12/2023 18:00	17	19.0	13.9	-5.1
2A	5/12/2023 19:00	17	19.0	13.9	-5.1
2A	5/12/2023 20:00	17	19.0	16.7	-2.3
2A	5/12/2023 21:00	17	19.0	16.7	-2.3
2A	5/12/2023 22:00	17	19.0	16.7	-2.3
2A	5/12/2023 23:00	17	19.0	16.7	-2.3
2A	5/13/2023 0:00	17	19.0	17.2	-1.8
2A	5/13/2023 1:00	17	19.0	17.2	-1.8
2A	5/13/2023 2:00	17	19.0	17.2	-1.8
2A	5/13/2023 3:00	17	19.0	17.2	-1.8
2A	5/13/2023 4:00	17	19.0	17.2	-1.8
2A	5/13/2023 5:00	17	19.0	17.2	-1.8
2A	5/13/2023 6:00	17	19.0	17.2	-1.8
2A	5/13/2023 7:00	17	19.0	17.2	-1.8
2A	5/13/2023 8:00	17	19.0	15.7	-3.3
2A	5/13/2023 9:00	17	19.0	15.7	-3.3
2A	5/13/2023 10:00	17	19.0	15.7	-3.3
2A	5/13/2023 11:00	17	19.0	15.7	-3.3
2A	5/13/2023 12:00	17	19.0	18.4	-0.6
2A	5/13/2023 13:00	17	19.0	18.4	-0.6
2A	5/13/2023 14:00	17	19.0	18.4	-0.6
2A	5/13/2023 15:00	118	19.0	14.7	-4.3
2A	5/13/2023 16:00	118	19.0	13.4	-5.6
2A	5/13/2023 17:00	118	19.0	13.4	-5.6
2A	5/13/2023 18:00	17	19.0	13.4	-5.6
2A	5/13/2023 19:00	18	19.0	16.7	-2.3
2A	5/13/2023 20:00	18	19.0	19.5	0.5

2A	5/13/2023 21:00	18	19.0	19.5	0.5
2A	5/13/2023 22:00	18	19.0	19.5	0.5
2A	5/13/2023 23:00	18	19.0	19.5	0.5
2A	5/14/2023 0:00	18	19.0	17.2	-1.8
2A	5/14/2023 1:00	18	19.0	17.2	-1.8
2A	5/14/2023 2:00	18	19.0	17.2	-1.8
2A	5/14/2023 3:00	18	19.0	17.2	-1.8
2A	5/14/2023 4:00	18	19.0	17.2	-1.8
2A	5/14/2023 5:00	18	19.0	17.2	-1.8
2A	5/14/2023 6:00	18	19.0	17.2	-1.8
2A	5/14/2023 7:00	18	19.0	17.2	-1.8
2A	5/14/2023 8:00	18	19.0	12.6	-6.4
2A	5/14/2023 9:00	18	19.0	13.1	-5.9
2A	5/14/2023 10:00	18	19.0	13.1	-5.9
2A	5/14/2023 11:00	18	19.0	13.1	-5.9
2A	5/14/2023 12:00	18	19.0	15.7	-3.3
2A	5/14/2023 13:00	18	19.0	15.7	-3.3
2A	5/14/2023 14:00	18	19.0	15.7	-3.3
2A	5/14/2023 15:00	16	19.0	15.2	-3.8
2A	5/14/2023 16:00	16	19.0	16.7	-2.3
2A	5/14/2023 17:00	16	19.0	16.7	-2.3
2A	5/14/2023 18:00	16	19.0	16.7	-2.3
2A	5/14/2023 19:00	18	18.0	16.7	-1.3
2A	5/14/2023 20:00	18	18.0	16.7	-1.3
2A	5/14/2023 21:00	18	18.0	16.7	-1.3
2A	5/14/2023 22:00	18	18.0	16.7	-1.3
2A	5/14/2023 23:00	18	18.0	16.7	-1.3
2A	5/15/2023 0:00	18	18.0	14.4	-3.7
2A	5/15/2023 1:00	18	18.0	14.4	-3.7
2A	5/15/2023 2:00	18	18.0	14.4	-3.7
2A	5/15/2023 3:00	18	18.0	14.4	-3.7
2A	5/15/2023 4:00	18	18.0	14.4	-3.7
2A	5/15/2023 5:00	18	18.0	14.4	-3.7
2A	5/15/2023 6:00	18	18.0	14.4	-3.7
2A	5/15/2023 7:00	18	18.0	14.4	-3.7
2A	5/15/2023 8:00	19	18.0	15.7	-2.3
2A	5/15/2023 9:00	19	18.0	15.7	-2.3
2A	5/15/2023 10:00	19	18.0	15.7	-2.3
2A	5/15/2023 11:00	19	18.0	15.7	-2.3
2A	5/15/2023 12:00	19	18.0	18.4	0.4
2A	5/15/2023 13:00	19	18.0	18.4	0.4
2A	5/15/2023 14:00	19	18.0	18.4	0.4
2A	5/15/2023 15:00	19	15.0	18.4	3.4
2A	5/15/2023 16:00	19	15.0	16.7	1.7
2A	5/15/2023 17:00	19	15.0	16.7	1.7
2A	5/15/2023 18:00	19	15.0	16.7	1.7
2A	5/15/2023 19:00	19	15.0	16.7	1.7

2A	5/15/2023 20:00	16	19.0	19.5	0.5
2A	5/15/2023 21:00	16	19.0	19.5	0.5
2A	5/15/2023 22:00	16	19.0	19.5	0.5
2A	5/15/2023 23:00	16	19.0	19.5	0.5
2A	5/16/2023 0:00	16	19.0	20.1	1.1
2A	5/16/2023 1:00	16	19.0	20.1	1.1
2A	5/16/2023 2:00	18	19.0	20.1	1.1
2A	5/16/2023 3:00	18	19.0	20.1	1.1
2A	5/16/2023 4:00	18	19.0	20.1	1.1
2A	5/16/2023 5:00	18	19.0	20.1	1.1
2A	5/16/2023 6:00	18	19.0	20.1	1.1
2A	5/16/2023 7:00	18	19.0	20.1	1.1
2A	5/16/2023 8:00	18	19.0	18.4	-0.6
2A	5/16/2023 9:00	18	19.0	18.4	-0.6
2A	5/16/2023 10:00	18	19.0	18.4	-0.6
2A	5/16/2023 11:00	18	19.0	18.4	-0.6
2A	5/16/2023 12:00	18	19.0	18.4	-0.6
2A	5/16/2023 13:00	18	19.0	18.4	-0.6
2A	5/16/2023 14:00	18	19.0	18.4	-0.6
2A	5/16/2023 15:00	18	19.0	18.4	-0.6
2A	5/16/2023 16:00	18	19.0	19.5	0.5
2A	5/16/2023 17:00	18	19.0	19.5	0.5
2A	5/16/2023 18:00	18	19.0	19.5	0.5
2A	5/16/2023 19:00	18	19.0	19.5	0.5
2A	5/16/2023 20:00	18	19.0	16.7	-2.3
2A	5/16/2023 21:00	18	19.0	16.7	-2.3
2A	5/16/2023 22:00	18	19.0	16.7	-2.3
2A	5/16/2023 23:00	18	19.0	16.7	-2.3
2A	5/17/2023 0:00	19	19.0	14.4	-4.7
2A	5/17/2023 1:00	19	19.0	14.4	-4.7
2A	5/17/2023 2:00	19	19.0	14.4	-4.7
2A	5/17/2023 3:00	19	19.0	14.4	-4.7
2A	5/17/2023 4:00	19	19.0	14.4	-4.7
2A	5/17/2023 5:00	19	19.0	14.4	-4.7
2A	5/17/2023 6:00	19	19.0	14.4	-4.7
2A	5/17/2023 7:00	19	19.0	14.4	-4.7
2A	5/17/2023 8:00	19	19.0	18.4	-0.6
2A	5/17/2023 9:00	19	19.0	18.4	-0.6
2A	5/17/2023 10:00	19	19.0	18.4	-0.6
2A	5/17/2023 11:00	19	19.0	18.4	-0.6
2A	5/17/2023 12:00	19	19.0	21.0	2.0
2A	5/17/2023 13:00	19	19.0	21.0	2.0
2A	5/17/2023 14:00	19	19.0	21.0	2.0
2A	5/17/2023 15:00	19	19.0	21.0	2.0
2A	5/17/2023 16:00	19	19.0	19.5	0.5
2A	5/17/2023 17:00	19	19.0	19.5	0.5
2A	5/17/2023 18:00	19	19.0	19.5	0.5

2A	5/17/2023 19:00	19	19.0	19.5	0.5
2A	5/17/2023 20:00	19	19.0	19.5	0.5
2A	5/17/2023 21:00	19	19.0	19.5	0.5
2A	5/17/2023 22:00	19	19.0	19.5	0.5
2A	5/17/2023 23:00	19	19.0	19.5	0.5
2A	5/18/2023 0:00	19	19.0	18.7	-0.4
2A	5/18/2023 1:00	19	19.0	20.1	1.1
2A	5/18/2023 2:00	19	19.0	20.1	1.1
2A	5/18/2023 3:00	19	19.0	20.1	1.1
2A	5/18/2023 4:00	19	19.0	20.1	1.1
2A	5/18/2023 5:00	19	19.0	20.1	1.1
2A	5/18/2023 6:00	19	19.0	20.1	1.1
2A	5/18/2023 7:00	19	19.0	20.1	1.1
2A	5/18/2023 8:00	19	19.0	15.7	-3.3
2A	5/18/2023 9:00	19	19.0	14.7	-4.3
2A	5/18/2023 10:00	19	19.0	14.7	-4.3
2A	5/18/2023 11:00	19	19.0	14.7	-4.3
2A	5/18/2023 12:00	19	19.0	14.7	-4.3
2A	5/18/2023 13:00	19	19.0	14.7	-4.3
2A	5/18/2023 14:00	19	19.0	14.7	-4.3
2A	5/18/2023 15:00	17	19.0	14.7	-4.3
2A	5/18/2023 16:00	17	19.0	16.7	-2.3
2A	5/18/2023 17:00	17	19.0	16.7	-2.3
2A	5/18/2023 18:00	17	19.0	16.7	-2.3
2A	5/18/2023 19:00	17	19.0	16.7	-2.3
2A	5/18/2023 20:00	17	19.0	13.9	-5.1
2A	5/18/2023 21:00	17	19.0	13.9	-5.1
2A	5/18/2023 22:00	19	19.0	13.9	-5.1
2A	5/18/2023 23:00	19	19.0	13.9	-5.1
2A	5/19/2023 0:00	19	19.0	14.4	-4.7
2A	5/19/2023 1:00	19	19.0	14.4	-4.7
2A	5/19/2023 2:00	19	19.0	14.4	-4.7
2A	5/19/2023 3:00	19	19.0	14.4	-4.7
2A	5/19/2023 4:00	19	19.0	14.4	-4.7
2A	5/19/2023 5:00	19	19.0	14.4	-4.7
2A	5/19/2023 6:00	19	19.0	14.4	-4.7
2A	5/19/2023 7:00	19	19.0	14.4	-4.7
2A	5/19/2023 8:00	19	19.0	14.7	-4.3
2A	5/19/2023 9:00	19	19.0	14.7	-4.3
2A	5/19/2023 10:00	19	19.0	14.7	-4.3
2A	5/19/2023 11:00	19	19.0	14.7	-4.3
2A	5/19/2023 12:00	19	19.0	16.8	-2.2
2A	5/19/2023 13:00	19	19.0	16.8	-2.2
2A	5/19/2023 14:00	19	19.0	16.8	-2.2
2A	5/19/2023 15:00	17	19.0	16.8	-2.2
2A	5/19/2023 16:00	17	19.0	16.7	-2.3
2A	5/19/2023 17:00	17	19.0	16.7	-2.3

2A	5/19/2023 18:00	17	19.0	16.7	-2.3
2A	5/19/2023 19:00	17	19.0	16.7	-2.3
2A	5/19/2023 20:00	19	19.0	16.7	-2.3
2A	5/19/2023 21:00	19	19.0	16.7	-2.3
2A	5/19/2023 22:00	19	19.0	16.7	-2.3
2A	5/19/2023 23:00	19	19.0	16.7	-2.3
2A	5/20/2023 0:00	19	19.0	17.2	-1.8
2A	5/20/2023 1:00	19	19.0	17.2	-1.8
2A	5/20/2023 2:00	19	19.0	17.2	-1.8
2A	5/20/2023 3:00	19	19.0	17.2	-1.8
2A	5/20/2023 4:00	19	19.0	17.2	-1.8
2A	5/20/2023 5:00	19	19.0	17.2	-1.8
2A	5/20/2023 6:00	19	19.0	17.2	-1.8
2A	5/20/2023 7:00	19	19.0	17.2	-1.8
2A	5/20/2023 8:00	19	19.0	18.4	-0.6
2A	5/20/2023 9:00	19	19.0	18.4	-0.6
2A	5/20/2023 10:00	19	19.0	18.4	-0.6
2A	5/20/2023 11:00	19	19.0	18.4	-0.6
2A	5/20/2023 12:00	19	19.0	15.7	-3.3
2A	5/20/2023 13:00	19	19.0	15.7	-3.3
2A	5/20/2023 14:00	18	16.0	9.5	-6.6
2A	5/20/2023 15:00	18	16.0	15.7	-0.3
2A	5/20/2023 16:00	18	16.0	16.7	0.7
2A	5/20/2023 17:00	18	16.0	16.7	0.7
2A	5/20/2023 18:00	18	16.0	16.7	0.7
2A	5/20/2023 19:00	16	18.0	16.7	-1.3
2A	5/20/2023 20:00	16	18.0	16.7	-1.3
2A	5/20/2023 21:00	16	18.0	16.7	-1.3
2A	5/20/2023 22:00	16	18.0	16.7	-1.3
2A	5/20/2023 23:00	16	18.0	16.7	-1.3
2A	5/21/2023 0:00	16	18.0	20.1	2.1
2A	5/21/2023 1:00	16	19.0	20.1	1.1
2A	5/21/2023 2:00	16	19.0	20.1	1.1
2A	5/21/2023 3:00	16	19.0	20.1	1.1
2A	5/21/2023 4:00	16	19.0	20.1	1.1
2A	5/21/2023 5:00	18	19.0	20.1	1.1
2A	5/21/2023 6:00	18	19.0	20.1	1.1
2A	5/21/2023 7:00	18	19.0	20.1	1.1
2A	5/21/2023 8:00	18	19.0	18.4	-0.6
2A	5/21/2023 9:00	18	19.0	18.4	-0.6
2A	5/21/2023 10:00	18	19.0	18.4	-0.6
2A	5/21/2023 11:00	17	19.0	18.4	-0.6
2A	5/21/2023 12:00	17	19.0	15.7	-3.3
2A	5/21/2023 13:00	17	19.0	15.7	-3.3
2A	5/21/2023 14:00	17	19.0	15.7	-3.3
2A	5/21/2023 15:00	17	19.0	15.7	-3.3
2A	5/21/2023 16:00	17	19.0	16.7	-2.3

2A	5/21/2023 17:00	17	19.0	16.7	-2.3
2A	5/21/2023 18:00	17	19.0	16.7	-2.3
2A	5/21/2023 19:00	17	19.0	16.7	-2.3
2A	5/21/2023 20:00	17	19.0	16.7	-2.3
2A	5/21/2023 21:00	17	19.0	16.7	-2.3
2A	5/21/2023 22:00	17	19.0	16.7	-2.3
2A	5/21/2023 23:00	17	19.0	16.7	-2.3
2A	5/22/2023 0:00	17	19.0	17.2	-1.8
2A	5/22/2023 1:00	17	19.0	17.2	-1.8
2A	5/22/2023 2:00	17	19.0	17.2	-1.8
2A	5/22/2023 3:00	17	19.0	17.2	-1.8
2A	5/22/2023 4:00	17	19.0	17.2	-1.8
2A	5/22/2023 5:00	17	19.0	17.2	-1.8
2A	5/22/2023 6:00	17	19.0	17.2	-1.8
2A	5/22/2023 7:00	17	19.0	17.2	-1.8
2A	5/22/2023 8:00	17	19.0	15.7	-3.3
2A	5/22/2023 9:00	17	19.0	15.7	-3.3
2A	5/22/2023 10:00	17	19.0	15.7	-3.3
2A	5/22/2023 11:00	17	19.0	15.7	-3.3
2A	5/22/2023 12:00	17	19.0	18.4	-0.6
2A	5/22/2023 13:00	17	19.0	18.4	-0.6
2A	5/22/2023 14:00	17	19.0	18.4	-0.6
2A	5/22/2023 15:00	17	19.0	18.4	-0.6
2A	5/22/2023 16:00	17	19.0	16.7	-2.3
2A	5/22/2023 17:00	16	19.0	16.7	-2.3
2A	5/22/2023 18:00	16	19.0	16.7	-2.3
2A	5/22/2023 19:00	19	19.0	16.7	-2.3
2A	5/22/2023 20:00	19	19.0	17.8	-1.2
2A	5/22/2023 21:00	19	19.0	17.8	-1.2
2A	5/22/2023 22:00	19	19.0	17.8	-1.2
2A	5/22/2023 23:00	19	19.0	17.8	-1.2
2A	5/23/2023 0:00	19	19.0	16.1	-2.9
2A	5/23/2023 1:00	19	19.0	16.1	-2.9
2A	5/23/2023 2:00	19	19.0	16.1	-2.9
2A	5/23/2023 3:00	19	19.0	16.1	-2.9
2A	5/23/2023 4:00	19	19.0	16.1	-2.9
2A	5/23/2023 5:00	19	19.0	16.1	-2.9
2A	5/23/2023 6:00	19	19.0	16.1	-2.9
2A	5/23/2023 7:00	19	19.0	16.1	-2.9
2A	5/23/2023 8:00	19	19.0	14.7	-4.3
2A	5/23/2023 9:00	19	19.0	14.7	-4.3
2A	5/23/2023 10:00	19	19.0	14.7	-4.3
2A	5/23/2023 11:00	19	19.0	14.7	-4.3
2A	5/23/2023 12:00	19	19.0	16.8	-2.2
2A	5/23/2023 13:00	19	19.0	16.8	-2.2
2A	5/23/2023 14:00	19	19.0	16.8	-2.2
2A	5/23/2023 15:00	18	19.0	14.7	-4.3

2A	5/23/2023 16:00	18	19.0	13.9	-5.1
2A	5/23/2023 17:00	18	19.0	13.9	-5.1
2A	5/23/2023 18:00	18	19.0	13.9	-5.1
2A	5/23/2023 19:00	19	19.0	16.7	-2.3
2A	5/23/2023 20:00	19	19.0	16.7	-2.3
2A	5/23/2023 21:00	19	19.0	16.7	-2.3
2A	5/23/2023 22:00	19	19.0	16.7	-2.3
2A	5/23/2023 23:00	19	19.0	16.7	-2.3
2A	5/24/2023 0:00	19	19.0	17.2	-1.8
2A	5/24/2023 1:00	19	19.0	17.2	-1.8
2A	5/24/2023 2:00	19	19.0	17.2	-1.8
2A	5/24/2023 3:00	19	19.0	17.2	-1.8
2A	5/24/2023 4:00	19	19.0	17.2	-1.8
2A	5/24/2023 5:00	19	19.0	17.2	-1.8
2A	5/24/2023 6:00	19	19.0	17.2	-1.8
2A	5/24/2023 7:00	19	19.0	17.2	-1.8
2A	5/24/2023 8:00	19	19.0	13.1	-5.9
2A	5/24/2023 9:00	19	19.0	13.1	-5.9
2A	5/24/2023 10:00	19	19.0	13.1	-5.9
2A	5/24/2023 11:00	19	19.0	13.1	-5.9
2A	5/24/2023 12:00	19	19.0	13.1	-5.9
2A	5/24/2023 13:00	19	19.0	13.1	-5.9
2A	5/24/2023 14:00	19	19.0	13.1	-5.9
2A	5/24/2023 15:00	19	19.0	13.1	-5.9
2A	5/24/2023 16:00	19	19.0	13.9	-5.1
2A	5/24/2023 17:00	19	19.0	13.9	-5.1
2A	5/24/2023 18:00	19	19.0	13.9	-5.1
2A	5/24/2023 19:00	19	19.0	13.9	-5.1
2A	5/24/2023 20:00	19	19.0	16.7	-2.3
2A	5/24/2023 21:00	19	19.0	16.7	-2.3
2A	5/24/2023 22:00	19	19.0	16.7	-2.3
2A	5/24/2023 23:00	19	19.0	16.7	-2.3
2A	5/25/2023 0:00	19	19.0	17.2	-1.8
2A	5/25/2023 1:00	19	19.0	17.2	-1.8
2A	5/25/2023 2:00	19	19.0	17.2	-1.8
2A	5/25/2023 3:00	19	19.0	17.2	-1.8
2A	5/25/2023 4:00	19	19.0	17.2	-1.8
2A	5/25/2023 5:00	19	19.0	17.2	-1.8
2A	5/25/2023 6:00	19	19.0	17.2	-1.8
2A	5/25/2023 7:00	19	19.0	17.2	-1.8
2A	5/25/2023 8:00	19	19.0	15.7	-3.3
2A	5/25/2023 9:00	19	19.0	15.7	-3.3
2A	5/25/2023 10:00	19	19.0	15.7	-3.3
2A	5/25/2023 11:00	19	19.0	15.7	-3.3
2A	5/25/2023 12:00	19	19.0	15.7	-3.3
2A	5/25/2023 13:00	19	19.0	15.7	-3.3
2A	5/25/2023 14:00	19	19.0	15.7	-3.3

2A	5/25/2023 15:00	19	19.0	15.7	-3.3
2A	5/25/2023 16:00	19	19.0	16.7	-2.3
2A	5/25/2023 17:00	19	19.0	16.7	-2.3
2A	5/25/2023 18:00	19	19.0	16.7	-2.3
2A	5/25/2023 19:00	19	19.0	16.7	-2.3
2A	5/25/2023 20:00	19	19.0	19.5	0.5
2A	5/25/2023 21:00	19	19.0	19.5	0.5
2A	5/25/2023 22:00	19	19.0	19.5	0.5
2A	5/25/2023 23:00	19	19.0	19.5	0.5
3C	1/1/2023 0:00	22	30.0	20.0	-10.0
3C	1/1/2023 1:00	22	30.0	20.0	-10.0
3C	1/1/2023 2:00	22	30.0	20.0	-10.0
3C	1/1/2023 3:00	22	30.0	20.0	-10.0
3C	1/1/2023 4:00	22	30.0	20.0	-10.0
3C	1/1/2023 5:00	22	30.0	20.0	-10.0
3C	1/1/2023 6:00	22	30.0	20.0	-10.0
3C	1/1/2023 7:00	21	30.0	18.7	-11.3
3C	1/1/2023 8:00	21	30.0	16.3	-13.7
3C	1/1/2023 9:00	21	30.0	16.3	-13.7
3C	1/1/2023 10:00	21	30.0	16.3	-13.7
3C	1/1/2023 11:00	21	30.0	16.3	-13.7
3C	1/1/2023 12:00	21	30.0	20.3	-9.7
3C	1/1/2023 13:00	21	30.0	20.3	-9.7
3C	1/1/2023 14:00	21	30.0	20.3	-9.7
3C	1/1/2023 15:00	21	30.0	20.3	-9.7
3C	1/1/2023 16:00	21	30.0	25.8	-4.2
3C	1/1/2023 17:00	21	30.0	25.8	-4.2
3C	1/1/2023 18:00	21	30.0	25.8	-4.2
3C	1/1/2023 19:00	21	30.0	23.4	-6.6
3C	1/1/2023 20:00	21	30.0	21.1	-8.9
3C	1/1/2023 21:00	22	30.0	23.4	-6.6
3C	1/1/2023 22:00	22	30.0	23.4	-6.6
3C	1/1/2023 23:00	22	30.0	25.8	-4.2
3C	1/2/2023 0:00	22	30.0	23.3	-6.7
3C	1/2/2023 1:00	22	30.0	23.3	-6.7
3C	1/2/2023 2:00	22	30.0	23.3	-6.7
3C	1/2/2023 3:00	22	30.0	23.3	-6.7
3C	1/2/2023 4:00	22	30.0	23.3	-6.7
3C	1/2/2023 5:00	22	30.0	23.3	-6.7
3C	1/2/2023 6:00	23	30.0	23.3	-6.7
3C	1/2/2023 7:00	23	30.0	23.3	-6.7
3C	1/2/2023 8:00	23	30.0	15.3	-14.7
3C	1/2/2023 9:00	23	30.0	15.3	-14.7
3C	1/2/2023 10:00	23	30.0	15.3	-14.7
3C	1/2/2023 11:00	20	30.0	15.3	-14.7
3C	1/2/2023 12:00	20	30.0	20.3	-9.7
3C	1/2/2023 13:00	20	30.0	18.3	-11.7

3C	1/2/2023 14:00	20	30.0	18.3	-11.7
3C	1/2/2023 15:00	20	30.0	18.3	-11.7
3C	1/2/2023 16:00	20	30.0	23.4	-6.6
3C	1/2/2023 17:00	20	30.0	23.4	-6.6
3C	1/2/2023 18:00	20	30.0	23.4	-6.6
3C	1/2/2023 19:00	20	30.0	23.4	-6.6
3C	1/2/2023 20:00	20	30.0	16.4	-13.6
3C	1/2/2023 21:00	20	30.0	16.4	-13.6
3C	1/2/2023 22:00	20	30.0	16.4	-13.6
3C	1/2/2023 23:00	20	30.0	16.4	-13.6
3C	1/3/2023 0:00	20	30.0	23.3	-6.7
3C	1/3/2023 1:00	20	30.0	23.3	-6.7
3C	1/3/2023 2:00	20	30.0	23.3	-6.7
3C	1/3/2023 3:00	20	30.0	23.3	-6.7
3C	1/3/2023 4:00	20	30.0	23.3	-6.7
3C	1/3/2023 5:00	20	30.0	23.3	-6.7
3C	1/3/2023 6:00	20	30.0	23.3	-6.7
3C	1/3/2023 7:00	20	30.0	23.3	-6.7
3C	1/3/2023 8:00	21	30.0	22.4	-7.6
3C	1/3/2023 9:00	21	30.0	22.4	-7.6
3C	1/3/2023 10:00	21	30.0	22.4	-7.6
3C	1/3/2023 11:00	21	30.0	22.4	-7.6
3C	1/3/2023 12:00	21	30.0	20.3	-9.7
3C	1/3/2023 13:00	21	30.0	20.3	-9.7
3C	1/3/2023 14:00	21	30.0	20.3	-9.7
3C	1/3/2023 15:00	21	30.0	20.3	-9.7
3C	1/3/2023 16:00	21	30.0	23.4	-6.6
3C	1/3/2023 17:00	21	30.0	23.4	-6.6
3C	1/3/2023 18:00	21	30.0	23.4	-6.6
3C	1/3/2023 19:00	21	30.0	23.4	-6.6
3C	1/3/2023 20:00	22	30.0	14.6	-15.4
3C	1/3/2023 21:00	22	30.0	14.6	-15.4
3C	1/3/2023 22:00	22	30.0	14.6	-15.4
3C	1/3/2023 23:00	22	30.0	14.6	-15.4
3C	1/4/2023 0:00	22	23.0	16.7	-6.3
3C	1/4/2023 1:00	22	23.0	16.7	-6.3
3C	1/4/2023 2:00	22	23.0	16.7	-6.3
3C	1/4/2023 3:00	22	23.0	16.7	-6.3
3C	1/4/2023 4:00	22	23.0	16.7	-6.3
3C	1/4/2023 5:00	22	23.0	16.7	-6.3
3C	1/4/2023 6:00	22	23.0	16.7	-6.3
3C	1/4/2023 7:00	22	23.0	16.7	-6.3
3C	1/4/2023 8:00	22	23.0	20.3	-2.7
3C	1/4/2023 9:00	22	23.0	20.3	-2.7
3C	1/4/2023 10:00	22	23.0	20.3	-2.7
3C	1/4/2023 11:00	22	23.0	20.3	-2.7
3C	1/4/2023 12:00	22	23.0	20.3	-2.7

3C	1/4/2023 13:00	22	23.0	20.3	-2.7
3C	1/4/2023 14:00	25	23.0	20.3	-2.7
3C	1/4/2023 15:00	25	23.0	20.3	-2.7
3C	1/4/2023 16:00	25	23.0	20.5	-2.5
3C	1/4/2023 17:00	25	23.0	20.5	-2.5
3C	1/4/2023 18:00	25	23.0	20.5	-2.5
3C	1/4/2023 19:00	26	23.0	20.5	-2.5
3C	1/4/2023 20:00	26	23.0	17.6	-5.4
3C	1/4/2023 21:00	26	23.0	17.6	-5.4
3C	1/4/2023 22:00	26	23.0	17.6	-5.4
3C	1/4/2023 23:00	26	23.0	17.6	-5.4
3C	1/5/2023 0:00	26	27.0	16.7	-10.3
3C	1/5/2023 1:00	26	27.0	16.7	-10.3
3C	1/5/2023 2:00	26	27.0	16.7	-10.3
3C	1/5/2023 3:00	26	27.0	16.7	-10.3
3C	1/5/2023 4:00	26	27.0	20.0	-7.0
3C	1/5/2023 5:00	26	27.0	20.0	-7.0
3C	1/5/2023 6:00	26	27.0	20.0	-7.0
3C	1/5/2023 7:00	31	27.0	20.0	-7.0
3C	1/5/2023 8:00	31	27.0	20.3	-6.7
3C	1/5/2023 9:00	31	27.0	20.3	-6.7
3C	1/5/2023 10:00	31	27.0	20.3	-6.7
3C	1/5/2023 11:00	31	27.0	20.3	-6.7
3C	1/5/2023 12:00	31	27.0	28.0	1.0
3C	1/5/2023 13:00	31	27.0	28.0	1.0
3C	1/5/2023 14:00	31	27.0	28.0	1.0
3C	1/5/2023 15:00	28	25.0	28.0	3.0
3C	1/5/2023 16:00	28	25.0	28.1	3.1
3C	1/5/2023 17:00	28	25.0	28.1	3.1
3C	1/5/2023 18:00	28	25.0	28.1	3.1
3C	1/5/2023 19:00	28	25.0	28.1	3.1
3C	1/5/2023 20:00	28	25.0	23.4	-1.6
3C	1/5/2023 21:00	28	25.0	23.4	-1.6
3C	1/5/2023 22:00	28	25.0	23.4	-1.6
3C	1/5/2023 23:00	25	28.0	19.9	-8.1
3C	1/6/2023 0:00	25	28.0	18.7	-9.3
3C	1/6/2023 1:00	27	28.0	18.7	-9.3
3C	1/6/2023 2:00	27	28.0	18.7	-9.3
3C	1/6/2023 3:00	27	28.0	18.7	-9.3
3C	1/6/2023 4:00	27	28.0	18.7	-9.3
3C	1/6/2023 5:00	27	28.0	18.7	-9.3
3C	1/6/2023 6:00	27	28.0	18.7	-9.3
3C	1/6/2023 7:00	27	28.0	18.7	-9.3
3C	1/6/2023 8:00	31	28.0	20.3	-7.7
3C	1/6/2023 9:00	31	28.0	20.3	-7.7
3C	1/6/2023 10:00	31	28.0	20.3	-7.7
3C	1/6/2023 11:00	31	27.0	20.3	-6.7

3C	1/6/2023 12:00	31	27.0	22.9	-4.1
3C	1/6/2023 13:00	31	27.0	22.9	-4.1
3C	1/6/2023 14:00	31	27.0	22.9	-4.1
3C	1/6/2023 15:00	31	27.0	22.9	-4.1
3C	1/6/2023 16:00	31	27.0	20.5	-6.5
3C	1/6/2023 17:00	31	27.0	20.5	-6.5
3C	1/6/2023 18:00	31	27.0	20.5	-6.5
3C	1/6/2023 19:00	31	27.0	20.5	-6.5
3C	1/6/2023 20:00	31	27.0	28.1	1.1
3C	1/6/2023 21:00	31	27.0	28.1	1.1
3C	1/6/2023 22:00	31	27.0	28.1	1.1
3C	1/6/2023 23:00	31	27.0	28.1	1.1
3C	1/7/2023 0:00	31	27.0	26.7	-0.4
3C	1/7/2023 1:00	31	27.0	26.7	-0.4
3C	1/7/2023 2:00	31	19.0	26.7	7.7
3C	1/7/2023 3:00	31	27.0	26.7	-0.4
3C	1/7/2023 4:00	31	27.0	26.7	-0.4
3C	1/7/2023 5:00	31	27.0	26.7	-0.4
3C	1/7/2023 6:00	31	27.0	26.7	-0.4
3C	1/7/2023 7:00	28	28.0	26.7	-1.4
3C	1/7/2023 8:00	28	28.0	20.3	-7.7
3C	1/7/2023 9:00	28	28.0	20.3	-7.7
3C	1/7/2023 10:00	28	28.0	20.3	-7.7
3C	1/7/2023 11:00	28	28.0	20.3	-7.7
3C	1/7/2023 12:00	28	28.0	20.3	-7.7
3C	1/7/2023 13:00	28	28.0	20.3	-7.7
3C	1/7/2023 14:00	28	28.0	20.3	-7.7
3C	1/7/2023 15:00	30	19.0	20.3	1.3
3C	1/7/2023 16:00	30	19.0	23.4	4.4
3C	1/7/2023 17:00	30	19.0	23.4	4.4
3C	1/7/2023 18:00	30	19.0	23.4	4.4
3C	1/7/2023 19:00	22	19.0	23.4	4.4
3C	1/7/2023 20:00	22	19.0	20.5	1.5
3C	1/7/2023 21:00	22	19.0	20.5	1.5
3C	1/7/2023 22:00	22	19.0	20.5	1.5
3C	1/7/2023 23:00	22	19.0	20.5	1.5
3C	1/8/2023 0:00	22	19.0	23.3	4.3
3C	1/8/2023 1:00	22	19.0	23.3	4.3
3C	1/8/2023 2:00	22	23.0	23.3	0.3
3C	1/8/2023 3:00	22	23.0	23.3	0.3
3C	1/8/2023 4:00	22	23.0	23.3	0.3
3C	1/8/2023 5:00	22	23.0	23.3	0.3
3C	1/8/2023 6:00	22	23.0	23.3	0.3
3C	1/8/2023 7:00	22	28.0	23.3	-4.7
3C	1/8/2023 8:00	22	28.0	22.4	-5.6
3C	1/8/2023 9:00	22	28.0	22.4	-5.6
3C	1/8/2023 10:00	22	28.0	22.4	-5.6

3C	1/8/2023 11:00	22	28.0	22.4	-5.6
3C	1/8/2023 12:00	22	28.0	22.4	-5.6
3C	1/8/2023 13:00	22	28.0	22.4	-5.6
3C	1/8/2023 14:00	22	28.0	22.4	-5.6
3C	1/8/2023 15:00	20	28.0	22.4	-5.6
3C	1/8/2023 16:00	20	28.0	23.4	-4.6
3C	1/8/2023 17:00	20	28.0	23.4	-4.6
3C	1/8/2023 18:00	20	28.0	23.4	-4.6
3C	1/8/2023 19:00	19	28.0	23.4	-4.6
3C	1/8/2023 20:00	19	23.0	16.4	-6.6
3C	1/8/2023 21:00	19	23.0	16.4	-6.6
3C	1/8/2023 22:00	19	23.0	16.4	-6.6
3C	1/8/2023 23:00	19	23.0	16.4	-6.6
3C	1/9/2023 0:00	19	23.0	23.3	0.3
3C	1/9/2023 1:00	19	23.0	23.3	0.3
3C	1/9/2023 2:00	19	23.0	23.3	0.3
3C	1/9/2023 3:00	19	23.0	23.3	0.3
3C	1/9/2023 4:00	19	23.0	23.3	0.3
3C	1/9/2023 5:00	19	23.0	23.3	0.3
3C	1/9/2023 6:00	19	23.0	23.3	0.3
3C	1/9/2023 7:00	21	30.0	23.3	-6.7
3C	1/9/2023 8:00	21	30.0	22.9	-7.1
3C	1/9/2023 9:00	21	30.0	22.9	-7.1
3C	1/9/2023 10:00	21	30.0	22.9	-7.1
3C	1/9/2023 11:00	21	30.0	22.4	-7.6
3C	1/9/2023 12:00	21	30.0	25.4	-4.6
3C	1/9/2023 13:00	21	30.0	25.4	-4.6
3C	1/9/2023 14:00	21	30.0	25.4	-4.6
3C	1/9/2023 15:00	21	21.0	25.4	4.4
3C	1/9/2023 16:00	21	21.0	17.6	-3.4
3C	1/9/2023 17:00	21	21.0	17.6	-3.4
3C	1/9/2023 18:00	21	21.0	17.6	-3.4
3C	1/9/2023 19:00	29	25.0	19.0	-6.0
3C	1/9/2023 20:00	29	25.0	20.5	-4.5
3C	1/9/2023 21:00	29	25.0	20.5	-4.5
3C	1/9/2023 22:00	29	25.0	20.5	-4.5
3C	1/9/2023 23:00	28	25.0	17.6	-7.4
3C	1/10/2023 0:00	28	25.0	18.7	-6.3
3C	1/10/2023 1:00	28	25.0	18.7	-6.3
3C	1/10/2023 2:00	28	25.0	18.7	-6.3
3C	1/10/2023 3:00	28	25.0	18.7	-6.3
3C	1/10/2023 4:00	28	25.0	18.7	-6.3
3C	1/10/2023 5:00	28	25.0	18.7	-6.3
3C	1/10/2023 6:00	28	25.0	18.7	-6.3
3C	1/10/2023 7:00	28	25.0	18.7	-6.3
3C	1/10/2023 8:00	28	29.0	17.8	-11.2
3C	1/10/2023 9:00	28	29.0	17.8	-11.2

3C	1/10/2023 10:00	28	29.0	17.8	-11.2
3C	1/10/2023 11:00	28	29.0	17.8	-11.2
3C	1/10/2023 12:00	28	29.0	22.9	-6.1
3C	1/10/2023 13:00	28	29.0	22.9	-6.1
3C	1/10/2023 14:00	28	29.0	22.9	-6.1
3C	1/10/2023 15:00	28	29.0	22.9	-6.1
3C	1/10/2023 16:00	28	29.0	23.4	-5.6
3C	1/10/2023 17:00	28	29.0	23.4	-5.6
3C	1/10/2023 18:00	28	29.0	23.4	-5.6
3C	1/10/2023 19:00	28	29.0	23.4	-5.6
3C	1/10/2023 20:00	28	29.0	30.5	1.5
3C	1/10/2023 21:00	28	29.0	30.5	1.5
3C	1/10/2023 22:00	28	29.0	30.5	1.5
3C	1/10/2023 23:00	28	29.0	30.5	1.5
3C	1/11/2023 0:00	28	29.0	26.7	-2.4
3C	1/11/2023 1:00	28	29.0	26.7	-2.4
3C	1/11/2023 2:00	28	29.0	26.7	-2.4
3C	1/11/2023 3:00	28	29.0	26.7	-2.4
3C	1/11/2023 4:00	28	29.0	26.7	-2.4
3C	1/11/2023 5:00	28	29.0	26.7	-2.4
3C	1/11/2023 6:00	28	29.0	26.7	-2.4
3C	1/11/2023 7:00	28	33.0	26.7	-6.4
3C	1/11/2023 8:00	29	33.0	20.3	-12.7
3C	1/11/2023 9:00	29	33.0	20.3	-12.7
3C	1/11/2023 10:00	29	33.0	20.3	-12.7
3C	1/11/2023 11:00	29	33.0	20.3	-12.7
3C	1/11/2023 12:00	29	33.0	25.4	-7.6
3C	1/11/2023 13:00	29	33.0	25.4	-7.6
3C	1/11/2023 14:00	29	33.0	25.4	-7.6
3C	1/11/2023 15:00	29	33.0	25.4	-7.6
3C	1/11/2023 16:00	26	30.0	25.8	-4.2
3C	1/11/2023 17:00	26	30.0	25.8	-4.2
3C	1/11/2023 18:00	26	30.0	25.8	-4.2
3C	1/11/2023 19:00	26	30.0	25.8	-4.2
3C	1/11/2023 20:00	26	30.0	25.8	-4.2
3C	1/11/2023 21:00	26	30.0	25.8	-4.2
3C	1/11/2023 22:00	26	30.0	25.8	-4.2
3C	1/11/2023 23:00	26	30.0	25.8	-4.2
3C	1/12/2023 0:00	26	30.0	29.3	-0.7
3C	1/12/2023 1:00	26	30.0	29.3	-0.7
3C	1/12/2023 2:00	26	30.0	29.3	-0.7
3C	1/12/2023 3:00	26	30.0	29.3	-0.7
3C	1/12/2023 4:00	26	30.0	29.3	-0.7
3C	1/12/2023 5:00	26	30.0	29.3	-0.7
3C	1/12/2023 6:00	26	30.0	29.3	-0.7
3C	1/12/2023 7:00	26	30.0	30.0	0.0
3C	1/12/2023 8:00	26	30.0	17.8	-12.2

3C	1/12/2023 9:00	26	30.0	17.8	-12.2
3C	1/12/2023 10:00	26	30.0	17.8	-12.2
3C	1/12/2023 11:00	26	30.0	17.8	-12.2
3C	1/12/2023 12:00	26	30.0	17.8	-12.2
3C	1/12/2023 13:00	26	30.0	17.8	-12.2
3C	1/12/2023 14:00	26	30.0	17.8	-12.2
3C	1/12/2023 15:00	26	30.0	17.8	-12.2
3C	1/12/2023 16:00	26	30.0	14.6	-15.4
3C	1/12/2023 17:00	26	30.0	14.6	-15.4
3C	1/12/2023 18:00	26	30.0	14.6	-15.4
3C	1/12/2023 19:00	29	30.0	14.6	-15.4
3C	1/12/2023 20:00	29	30.0	25.8	-4.2
3C	1/12/2023 21:00	29	30.0	25.8	-4.2
3C	1/12/2023 22:00	29	30.0	25.8	-4.2
3C	1/12/2023 23:00	29	30.0	25.8	-4.2
3C	1/13/2023 0:00	29	30.0	23.3	-6.7
3C	1/13/2023 1:00	29	30.0	23.3	-6.7
3C	1/13/2023 2:00	29	30.0	23.3	-6.7
3C	1/13/2023 3:00	29	30.0	23.3	-6.7
3C	1/13/2023 4:00	29	30.0	23.3	-6.7
3C	1/13/2023 5:00	29	30.0	23.3	-6.7
3C	1/13/2023 6:00	29	30.0	23.3	-6.7
3C	1/13/2023 7:00	29	30.0	23.3	-6.7
3C	1/13/2023 8:00	29	33.0	17.8	-15.2
3C	1/13/2023 9:00	29	33.0	17.8	-15.2
3C	1/13/2023 10:00	29	33.0	17.8	-15.2
3C	1/13/2023 11:00	29	33.0	17.8	-15.2
3C	1/13/2023 12:00	29	33.0	20.3	-12.7
3C	1/13/2023 13:00	29	33.0	20.3	-12.7
3C	1/13/2023 14:00	29	33.0	20.3	-12.7
3C	1/13/2023 15:00	29	33.0	20.3	-12.7
3C	1/13/2023 16:00	29	33.0	17.6	-15.4
3C	1/13/2023 17:00	29	33.0	17.6	-15.4
3C	1/13/2023 18:00	29	33.0	17.6	-15.4
3C	1/13/2023 19:00	29	33.0	17.6	-15.4
3C	1/13/2023 20:00	29	33.0	20.5	-12.5
3C	1/13/2023 21:00	29	33.0	20.5	-12.5
3C	1/13/2023 22:00	29	33.0	20.5	-12.5
3C	1/13/2023 23:00	26	33.0	20.5	-12.5
3C	1/14/2023 0:00	26	33.0	20.0	-13.0
3C	1/14/2023 1:00	26	33.0	20.0	-13.0
3C	1/14/2023 2:00	26	33.0	20.0	-13.0
3C	1/14/2023 3:00	26	33.0	20.0	-13.0
3C	1/14/2023 4:00	26	33.0	20.0	-13.0
3C	1/14/2023 5:00	26	33.0	20.0	-13.0
3C	1/14/2023 6:00	26	33.0	20.0	-13.0
3C	1/14/2023 7:00	26	29.0	20.0	-9.0

3C	1/14/2023 8:00	26	29.0	17.8	-11.2
3C	1/14/2023 9:00	26	29.0	17.8	-11.2
3C	1/14/2023 10:00	26	29.0	17.8	-11.2
3C	1/14/2023 11:00	26	29.0	17.8	-11.2
3C	1/14/2023 12:00	22	28.0	20.3	-7.7
3C	1/14/2023 13:00	22	28.0	20.3	-7.7
3C	1/14/2023 14:00	22	28.0	20.3	-7.7
3C	1/14/2023 15:00	22	28.0	20.3	-7.7
3C	1/14/2023 16:00	21	28.0	23.4	-4.6
3C	1/14/2023 17:00	21	28.0	23.4	-4.6
3C	1/14/2023 18:00	21	28.0	23.4	-4.6
3C	1/14/2023 19:00	21	28.0	23.4	-4.6
3C	1/14/2023 20:00	21	27.0	23.4	-3.6
3C	1/14/2023 21:00	21	27.0	23.4	-3.6
3C	1/14/2023 22:00	21	27.0	23.4	-3.6
3C	1/14/2023 23:00	21	27.0	23.4	-3.6
3C	1/15/2023 0:00	21	27.0	21.3	-5.7
3C	1/15/2023 1:00	21	27.0	21.3	-5.7
3C	1/15/2023 2:00	21	30.0	21.3	-8.7
3C	1/15/2023 3:00	21	29.0	21.3	-7.7
3C	1/15/2023 4:00	21	29.0	21.3	-7.7
3C	1/15/2023 5:00	22	29.0	21.3	-7.7
3C	1/15/2023 6:00	22	29.0	21.3	-7.7
3C	1/15/2023 7:00	20	28.0	21.3	-6.7
3C	1/15/2023 8:00	20	28.0	22.4	-5.6
3C	1/15/2023 9:00	20	28.0	22.4	-5.6
3C	1/15/2023 10:00	20	28.0	22.4	-5.6
3C	1/15/2023 11:00	20	28.0	22.4	-5.6
3C	1/15/2023 12:00	20	28.0	24.4	-3.6
3C	1/15/2023 13:00	20	28.0	24.4	-3.6
3C	1/15/2023 14:00	20	28.0	24.4	-3.6
3C	1/15/2023 15:00	17	30.0	24.4	-5.6
3C	1/15/2023 16:00	17	30.0	29.3	-0.7
3C	1/15/2023 17:00	17	30.0	29.3	-0.7
3C	1/15/2023 18:00	17	30.0	29.3	-0.7
3C	1/15/2023 19:00	17	30.0	29.3	-0.7
3C	1/15/2023 20:00	17	30.0	23.4	-6.6
3C	1/15/2023 21:00	17	30.0	23.4	-6.6
3C	1/15/2023 22:00	17	30.0	23.4	-6.6
3C	1/15/2023 23:00	22	29.0	23.4	-5.6
3C	1/16/2023 0:00	22	29.0	24.0	-5.0
3C	1/16/2023 1:00	22	29.0	21.3	-7.7
3C	1/16/2023 2:00	22	29.0	21.3	-7.7
3C	1/16/2023 3:00	22	29.0	21.3	-7.7
3C	1/16/2023 4:00	23	29.0	24.0	-5.0
3C	1/16/2023 5:00	23	29.0	24.0	-5.0
3C	1/16/2023 6:00	23	29.0	24.0	-5.0

3C	1/16/2023 7:00	23	29.0	24.0	-5.0
3C	1/16/2023 8:00	23	29.0	25.4	-3.6
3C	1/16/2023 9:00	23	29.0	25.4	-3.6
3C	1/16/2023 10:00	23	29.0	25.4	-3.6
3C	1/16/2023 11:00	23	29.0	25.4	-3.6
3C	1/16/2023 12:00	23	29.0	25.4	-3.6
3C	1/16/2023 13:00	23	29.0	25.4	-3.6
3C	1/16/2023 14:00	23	29.0	25.4	-3.6
3C	1/16/2023 15:00	24	29.0	25.4	-3.6
3C	1/16/2023 16:00	24	29.0	25.8	-3.2
3C	1/16/2023 17:00	24	29.0	25.8	-3.2
3C	1/16/2023 18:00	24	29.0	25.8	-3.2
3C	1/16/2023 19:00	25	29.0	25.8	-3.2
3C	1/16/2023 20:00	25	29.0	21.1	-7.9
3C	1/16/2023 21:00	25	29.0	21.1	-7.9
3C	1/16/2023 22:00	25	29.0	21.1	-7.9
3C	1/16/2023 23:00	25	29.0	21.1	-7.9
3C	1/17/2023 0:00	25	29.0	26.7	-2.4
3C	1/17/2023 1:00	26	29.0	26.7	-2.4
3C	1/17/2023 2:00	26	29.0	26.7	-2.4
3C	1/17/2023 3:00	26	29.0	26.7	-2.4
3C	1/17/2023 4:00	26	29.0	26.7	-2.4
3C	1/17/2023 5:00	26	29.0	26.7	-2.4
3C	1/17/2023 6:00	26	29.0	26.7	-2.4
3C	1/17/2023 7:00	27	29.0	26.7	-2.4
3C	1/17/2023 8:00	27	29.0	20.3	-8.7
3C	1/17/2023 9:00	27	29.0	20.3	-8.7
3C	1/17/2023 10:00	27	29.0	20.3	-8.7
3C	1/17/2023 11:00	27	29.0	20.3	-8.7
3C	1/17/2023 12:00	27	29.0	25.4	-3.6
3C	1/17/2023 13:00	27	29.0	25.4	-3.6
3C	1/17/2023 14:00	27	29.0	25.4	-3.6
3C	1/17/2023 15:00	27	29.0	25.4	-3.6
3C	1/17/2023 16:00	27	29.0	23.4	-5.6
3C	1/17/2023 17:00	27	29.0	23.4	-5.6
3C	1/17/2023 18:00	27	29.0	23.4	-5.6
3C	1/17/2023 19:00	27	29.0	23.4	-5.6
3C	1/17/2023 20:00	27	29.0	23.4	-5.6
3C	1/17/2023 21:00	27	29.0	23.4	-5.6
3C	1/17/2023 22:00	27	29.0	23.4	-5.6
3C	1/17/2023 23:00	27	29.0	23.4	-5.6
3C	1/18/2023 0:00	27	29.0	16.7	-12.3
3C	1/18/2023 1:00	27	29.0	16.7	-12.3
3C	1/18/2023 2:00	27	29.0	16.7	-12.3
3C	1/18/2023 3:00	27	29.0	16.7	-12.3
3C	1/18/2023 4:00	27	29.0	16.7	-12.3
3C	1/18/2023 5:00	27	29.0	16.7	-12.3

3C	1/18/2023 6:00	27	29.0	16.7	-12.3
3C	1/18/2023 7:00	27	29.0	16.7	-12.3
3C	1/18/2023 8:00	27	29.0	20.3	-8.7
3C	1/18/2023 9:00	27	29.0	20.3	-8.7
3C	1/18/2023 10:00	27	29.0	20.3	-8.7
3C	1/18/2023 11:00	27	29.0	20.3	-8.7
3C	1/18/2023 12:00	27	29.0	25.4	-3.6
3C	1/18/2023 13:00	27	29.0	25.4	-3.6
3C	1/18/2023 14:00	27	29.0	25.4	-3.6
3C	1/18/2023 15:00	23	29.0	25.4	-3.6
3C	1/18/2023 16:00	23	29.0	23.4	-5.6
3C	1/18/2023 17:00	23	29.0	23.4	-5.6
3C	1/18/2023 18:00	23	29.0	23.4	-5.6
3C	1/18/2023 19:00	23	29.0	23.4	-5.6
3C	1/18/2023 20:00	23	29.0	26.4	-2.7
3C	1/18/2023 21:00	23	29.0	26.4	-2.7
3C	1/18/2023 22:00	23	29.0	26.4	-2.7
3C	1/18/2023 23:00	23	29.0	26.4	-2.7
3C	1/19/2023 0:00	23	29.0	26.7	-2.4
3C	1/19/2023 1:00	23	29.0	26.7	-2.4
3C	1/19/2023 2:00	23	29.0	26.7	-2.4
3C	1/19/2023 3:00	23	29.0	26.7	-2.4
3C	1/19/2023 4:00	23	29.0	26.7	-2.4
3C	1/19/2023 5:00	23	29.0	26.7	-2.4
3C	1/19/2023 6:00	23	29.0	26.7	-2.4
3C	1/19/2023 7:00	26	29.0	26.7	-2.4
3C	1/19/2023 8:00	26	29.0	20.3	-8.7
3C	1/19/2023 9:00	26	29.0	20.3	-8.7
3C	1/19/2023 10:00	26	29.0	20.3	-8.7
3C	1/19/2023 11:00	26	29.0	20.3	-8.7
3C	1/19/2023 12:00	26	29.0	25.4	-3.6
3C	1/19/2023 13:00	26	29.0	25.4	-3.6
3C	1/19/2023 14:00	26	29.0	25.4	-3.6
3C	1/19/2023 15:00	26	29.0	25.4	-3.6
3C	1/19/2023 16:00	26	29.0	23.4	-5.6
3C	1/19/2023 17:00	26	29.0	23.4	-5.6
3C	1/19/2023 18:00	26	29.0	23.4	-5.6
3C	1/19/2023 19:00	26	29.0	23.4	-5.6
3C	1/19/2023 20:00	26	29.0	18.7	-10.3
3C	1/19/2023 21:00	26	29.0	18.7	-10.3
3C	1/19/2023 22:00	26	29.0	18.7	-10.3
3C	1/19/2023 23:00	26	29.0	18.7	-10.3
3C	1/20/2023 0:00	26	29.0	23.3	-5.7
3C	1/20/2023 1:00	26	29.0	23.3	-5.7
3C	1/20/2023 2:00	26	29.0	23.3	-5.7
3C	1/20/2023 3:00	26	29.0	23.3	-5.7
3C	1/20/2023 4:00	26	29.0	23.3	-5.7

3C	1/20/2023 5:00	26	29.0	23.3	-5.7
3C	1/20/2023 6:00	26	29.0	23.3	-5.7
3C	1/20/2023 7:00	23	25.0	23.3	-1.7
3C	1/20/2023 8:00	23	25.0	17.8	-7.2
3C	1/20/2023 9:00	23	25.0	17.8	-7.2
3C	1/20/2023 10:00	23	25.0	17.8	-7.2
3C	1/20/2023 11:00	23	25.0	17.8	-7.2
3C	1/20/2023 12:00	23	25.0	22.9	-2.1
3C	1/20/2023 13:00	23	25.0	22.9	-2.1
3C	1/20/2023 14:00	23	25.0	22.9	-2.1
3C	1/20/2023 15:00	23	25.0	22.9	-2.1
3C	1/20/2023 16:00	23	25.0	26.4	1.4
3C	1/20/2023 17:00	23	25.0	26.4	1.4
3C	1/20/2023 18:00	23	25.0	26.4	1.4
3C	1/20/2023 19:00	23	25.0	26.4	1.4
3C	1/20/2023 20:00	23	25.0	28.1	3.1
3C	1/20/2023 21:00	23	25.0	28.1	3.1
3C	1/20/2023 22:00	23	25.0	28.1	3.1
3C	1/20/2023 23:00	23	25.0	28.1	3.1
3C	1/21/2023 0:00	23	25.0	24.0	-1.0
3C	1/21/2023 1:00	23	25.0	24.0	-1.0
3C	1/21/2023 2:00	23	25.0	24.0	-1.0
3C	1/21/2023 3:00	23	25.0	24.0	-1.0
3C	1/21/2023 4:00	23	25.0	24.0	-1.0
3C	1/21/2023 5:00	23	25.0	24.0	-1.0
3C	1/21/2023 6:00	23	25.0	24.0	-1.0
3C	1/21/2023 7:00	23	25.0	16.7	-8.3
3C	1/21/2023 8:00	21	29.0	18.3	-10.7
3C	1/21/2023 9:00	21	29.0	18.3	-10.7
3C	1/21/2023 10:00	21	29.0	18.3	-10.7
3C	1/21/2023 11:00	21	29.0	18.3	-10.7
3C	1/21/2023 12:00	21	29.0	22.4	-6.6
3C	1/21/2023 13:00	21	29.0	22.4	-6.6
3C	1/21/2023 14:00	21	29.0	22.4	-6.6
3C	1/21/2023 15:00	17	29.0	24.4	-4.6
3C	1/21/2023 16:00	17	29.0	25.8	-3.2
3C	1/21/2023 17:00	17	29.0	25.8	-3.2
3C	1/21/2023 18:00	17	29.0	25.8	-3.2
3C	1/21/2023 19:00	18	29.0	25.8	-3.2
3C	1/21/2023 20:00	18	29.0	18.7	-10.3
3C	1/21/2023 21:00	18	29.0	18.7	-10.3
3C	1/21/2023 22:00	18	29.0	18.7	-10.3
3C	1/21/2023 23:00	18	29.0	18.7	-10.3
3C	1/22/2023 0:00	18	29.0	21.3	-7.7
3C	1/22/2023 1:00	18	29.0	21.3	-7.7
3C	1/22/2023 2:00	18	29.0	21.3	-7.7
3C	1/22/2023 3:00	18	29.0	21.3	-7.7

3C	1/22/2023 4:00	18	29.0	21.3	-7.7
3C	1/22/2023 5:00	18	29.0	21.3	-7.7
3C	1/22/2023 6:00	18	29.0	21.3	-7.7
3C	1/22/2023 7:00	18	29.0	20.0	-9.0
3C	1/22/2023 8:00	19	29.0	18.3	-10.7
3C	1/22/2023 9:00	19	29.0	18.3	-10.7
3C	1/22/2023 10:00	19	29.0	18.3	-10.7
3C	1/22/2023 11:00	19	29.0	18.3	-10.7
3C	1/22/2023 12:00	19	29.0	22.4	-6.6
3C	1/22/2023 13:00	19	29.0	22.4	-6.6
3C	1/22/2023 14:00	19	29.0	22.4	-6.6
3C	1/22/2023 15:00	19	29.0	22.4	-6.6
3C	1/22/2023 16:00	19	29.0	21.1	-7.9
3C	1/22/2023 17:00	19	29.0	21.1	-7.9
3C	1/22/2023 18:00	19	29.0	21.1	-7.9
3C	1/22/2023 19:00	19	29.0	21.1	-7.9
3C	1/22/2023 20:00	19	29.0	18.7	-10.3
3C	1/22/2023 21:00	19	29.0	18.7	-10.3
3C	1/22/2023 22:00	19	29.0	18.7	-10.3
3C	1/22/2023 23:00	19	29.0	18.7	-10.3
3C	1/23/2023 0:00	19	29.0	23.3	-5.7
3C	1/23/2023 1:00	19	29.0	23.3	-5.7
3C	1/23/2023 2:00	19	29.0	23.3	-5.7
3C	1/23/2023 3:00	19	29.0	23.3	-5.7
3C	1/23/2023 4:00	19	29.0	23.3	-5.7
3C	1/23/2023 5:00	21	29.0	23.3	-5.7
3C	1/23/2023 6:00	21	29.0	23.3	-5.7
3C	1/23/2023 7:00	21	29.0	23.3	-5.7
3C	1/23/2023 8:00	21	29.0	17.8	-11.2
3C	1/23/2023 9:00	21	29.0	17.8	-11.2
3C	1/23/2023 10:00	21	29.0	17.8	-11.2
3C	1/23/2023 11:00	21	29.0	20.3	-8.7
3C	1/23/2023 12:00	21	29.0	25.4	-3.6
3C	1/23/2023 13:00	21	29.0	25.4	-3.6
3C	1/23/2023 14:00	21	29.0	25.4	-3.6
3C	1/23/2023 15:00	21	28.0	25.4	-2.6
3C	1/23/2023 16:00	21	28.0	29.3	1.3
3C	1/23/2023 17:00	21	28.0	29.3	1.3
3C	1/23/2023 18:00	21	28.0	29.3	1.3
3C	1/23/2023 19:00	28	28.0	29.3	1.3
3C	1/23/2023 20:00	28	28.0	25.8	-2.2
3C	1/23/2023 21:00	27	28.0	23.4	-4.6
3C	1/23/2023 22:00	27	28.0	23.4	-4.6
3C	1/23/2023 23:00	27	27.0	23.4	-3.6
3C	1/24/2023 0:00	27	27.0	18.7	-8.3
3C	1/24/2023 1:00	27	27.0	18.7	-8.3
3C	1/24/2023 2:00	27	31.0	18.7	-12.3

3C	1/24/2023 3:00	27	27.0	18.7	-8.3
3C	1/24/2023 4:00	27	27.0	18.7	-8.3
3C	1/24/2023 5:00	27	27.0	18.7	-8.3
3C	1/24/2023 6:00	26	27.0	18.7	-8.3
3C	1/24/2023 7:00	26	27.0	20.0	-7.0
3C	1/24/2023 8:00	26	27.0	20.3	-6.7
3C	1/24/2023 9:00	26	27.0	20.3	-6.7
3C	1/24/2023 10:00	26	27.0	20.3	-6.7
3C	1/24/2023 11:00	26	27.0	20.3	-6.7
3C	1/24/2023 12:00	26	27.0	22.9	-4.1
3C	1/24/2023 13:00	26	27.0	22.9	-4.1
3C	1/24/2023 14:00	26	27.0	22.9	-4.1
3C	1/24/2023 15:00	22	31.0	22.9	-8.1
3C	1/24/2023 16:00	22	31.0	27.8	-3.2
3C	1/24/2023 17:00	22	31.0	29.3	-1.7
3C	1/24/2023 18:00	22	31.0	26.4	-4.7
3C	1/24/2023 19:00	23	31.0	26.4	-4.7
3C	1/24/2023 20:00	23	31.0	29.3	-1.7
3C	1/24/2023 21:00	23	31.0	29.3	-1.7
3C	1/24/2023 22:00	23	31.0	29.3	-1.7
3C	1/24/2023 23:00	23	31.0	29.3	-1.7
3C	1/25/2023 0:00	23	27.0	26.7	-0.4
3C	1/25/2023 1:00	23	27.0	26.7	-0.4
3C	1/25/2023 2:00	23	31.0	26.7	-4.4
3C	1/25/2023 3:00	23	31.0	26.7	-4.4
3C	1/25/2023 4:00	23	31.0	26.7	-4.4
3C	1/25/2023 5:00	23	31.0	26.7	-4.4
3C	1/25/2023 6:00	23	31.0	26.7	-4.4
3C	1/25/2023 7:00	23	31.0	26.7	-4.4
3C	1/25/2023 8:00	23	31.0	20.3	-10.7
3C	1/25/2023 9:00	23	31.0	20.3	-10.7
3C	1/25/2023 10:00	23	27.0	20.3	-6.7
3C	1/25/2023 11:00	23	27.0	20.3	-6.7
3C	1/25/2023 12:00	23	27.0	22.9	-4.1
3C	1/25/2023 13:00	23	27.0	22.9	-4.1
3C	1/25/2023 14:00	23	27.0	22.9	-4.1
3C	1/25/2023 15:00	19	31.0	22.9	-8.1
3C	1/25/2023 16:00	19	31.0	23.4	-7.6
3C	1/25/2023 17:00	19	31.0	23.4	-7.6
3C	1/25/2023 18:00	19	31.0	21.1	-9.9
3C	1/25/2023 19:00	19	31.0	21.1	-9.9
3C	1/25/2023 20:00	19	31.0	24.6	-6.4
3C	1/25/2023 21:00	19	31.0	25.8	-5.2
3C	1/25/2023 22:00	19	31.0	25.8	-5.2
3C	1/25/2023 23:00	19	31.0	25.2	-5.8
3C	1/26/2023 0:00	19	31.0	24.0	-7.0
3C	1/26/2023 1:00	19	31.0	24.0	-7.0

3C	1/26/2023 2:00	19	32.0	24.0	-8.0
3C	1/26/2023 3:00	19	32.0	24.0	-8.0
3C	1/26/2023 4:00	19	32.0	24.0	-8.0
3C	1/26/2023 5:00	19	32.0	24.0	-8.0
3C	1/26/2023 6:00	19	26.0	26.7	0.7
3C	1/26/2023 7:00	19	26.0	26.7	0.7
3C	1/26/2023 8:00	30	26.0	21.6	-4.4
3C	1/26/2023 9:00	30	26.0	22.9	-3.1
3C	1/26/2023 10:00	30	31.0	22.9	-8.1
3C	1/26/2023 11:00	30	31.0	22.9	-8.1
3C	1/26/2023 12:00	30	31.0	25.4	-5.6
3C	1/26/2023 13:00	30	31.0	25.4	-5.6
3C	1/26/2023 14:00	30	31.0	25.4	-5.6
3C	1/26/2023 15:00	25	32.0	25.4	-6.6
3C	1/26/2023 16:00	25	32.0	23.4	-8.6
3C	1/26/2023 17:00	25	32.0	23.4	-8.6
3C	1/26/2023 18:00	25	32.0	23.4	-8.6
3C	1/26/2023 19:00	25	32.0	23.4	-8.6
3C	1/26/2023 20:00	25	32.0	28.1	-3.9
3C	1/26/2023 21:00	25	32.0	28.1	-3.9
3C	1/26/2023 22:00	25	32.0	28.1	-3.9
3C	1/26/2023 23:00	25	32.0	28.1	-3.9
3C	1/27/2023 0:00	25	32.0	26.7	-5.4
3C	1/27/2023 1:00	25	32.0	26.7	-5.4
3C	1/27/2023 2:00	25	32.0	26.7	-5.4
3C	1/27/2023 3:00	25	32.0	26.7	-5.4
3C	1/27/2023 4:00	25	32.0	26.7	-5.4
3C	1/27/2023 5:00	25	32.0	26.7	-5.4
3C	1/27/2023 6:00	25	32.0	26.7	-5.4
3C	1/27/2023 7:00	25	32.0	26.7	-5.4
3C	1/27/2023 8:00	25	32.0	17.8	-14.2
3C	1/27/2023 9:00	25	32.0	17.8	-14.2
3C	1/27/2023 10:00	25	32.0	17.8	-14.2
3C	1/27/2023 11:00	25	32.0	17.8	-14.2
3C	1/27/2023 12:00	25	32.0	20.3	-11.7
3C	1/27/2023 13:00	25	32.0	20.3	-11.7
3C	1/27/2023 14:00	25	32.0	20.3	-11.7
3C	1/27/2023 15:00	25	32.0	20.3	-11.7
3C	1/27/2023 16:00	25	32.0	20.5	-11.5
3C	1/27/2023 17:00	25	32.0	20.5	-11.5
3C	1/27/2023 18:00	25	32.0	20.5	-11.5
3C	1/27/2023 19:00	21	32.0	20.5	-11.5
3C	1/27/2023 20:00	21	25.0	25.8	0.8
3C	1/27/2023 21:00	21	25.0	25.8	0.8
3C	1/27/2023 22:00	23	25.0	25.8	0.8
3C	1/27/2023 23:00	23	25.0	25.8	0.8
3C	1/28/2023 0:00	23	26.0	23.3	-2.7

3C	1/28/2023 1:00	23	26.0	23.3	-2.7
3C	1/28/2023 2:00	23	25.0	23.3	-1.7
3C	1/28/2023 3:00	23	26.0	23.3	-2.7
3C	1/28/2023 4:00	23	26.0	23.3	-2.7
3C	1/28/2023 5:00	23	26.0	23.3	-2.7
3C	1/28/2023 6:00	23	26.0	23.3	-2.7
3C	1/28/2023 7:00	24	26.0	23.3	-2.7
3C	1/28/2023 8:00	21	26.0	17.8	-8.2
3C	1/28/2023 9:00	21	26.0	17.8	-8.2
3C	1/28/2023 10:00	21	26.0	17.8	-8.2
3C	1/28/2023 11:00	21	26.0	17.8	-8.2
3C	1/28/2023 12:00	21	26.0	20.3	-5.7
3C	1/28/2023 13:00	21	26.0	20.3	-5.7
3C	1/28/2023 14:00	21	26.0	20.3	-5.7
3C	1/28/2023 15:00	20	26.0	20.3	-5.7
3C	1/28/2023 16:00	20	26.0	20.5	-5.5
3C	1/28/2023 17:00	20	26.0	20.5	-5.5
3C	1/28/2023 18:00	19	26.0	20.5	-5.5
3C	1/28/2023 19:00	19	26.0	20.5	-5.5
3C	1/28/2023 20:00	19	26.0	23.4	-2.6
3C	1/28/2023 21:00	19	26.0	23.4	-2.6
3C	1/28/2023 22:00	21	26.0	23.4	-2.6
3C	1/28/2023 23:00	21	26.0	20.5	-5.5
3C	1/29/2023 0:00	21	26.0	18.7	-7.3
3C	1/29/2023 1:00	21	26.0	18.7	-7.3
3C	1/29/2023 2:00	21	26.0	18.7	-7.3
3C	1/29/2023 3:00	21	26.0	18.7	-7.3
3C	1/29/2023 4:00	21	26.0	18.7	-7.3
3C	1/29/2023 5:00	21	26.0	18.7	-7.3
3C	1/29/2023 6:00	21	26.0	18.7	-7.3
3C	1/29/2023 7:00	21	26.0	18.7	-7.3
3C	1/29/2023 8:00	20	26.0	17.8	-8.2
3C	1/29/2023 9:00	20	26.0	17.8	-8.2
3C	1/29/2023 10:00	20	26.0	17.8	-8.2
3C	1/29/2023 11:00	20	26.0	17.8	-8.2
3C	1/29/2023 12:00	20	26.0	22.4	-3.6
3C	1/29/2023 13:00	20	26.0	22.4	-3.6
3C	1/29/2023 14:00	20	26.0	22.4	-3.6
3C	1/29/2023 15:00	20	26.0	22.4	-3.6
3C	1/29/2023 16:00	20	26.0	26.4	0.4
3C	1/29/2023 17:00	20	26.0	26.4	0.4
3C	1/29/2023 18:00	20	26.0	26.4	0.4
3C	1/29/2023 19:00	20	26.0	29.3	3.3
3C	1/29/2023 20:00	20	26.0	26.4	0.4
3C	1/29/2023 21:00	20	26.0	26.4	0.4
3C	1/29/2023 22:00	20	26.0	26.4	0.4
3C	1/29/2023 23:00	21	26.0	25.8	-0.2

3C	1/30/2023 0:00	21	24.0	21.3	-2.7
3C	1/30/2023 1:00	21	24.0	21.3	-2.7
3C	1/30/2023 2:00	21	22.0	21.3	-0.7
3C	1/30/2023 3:00	21	22.0	21.3	-0.7
3C	1/30/2023 4:00	21	22.0	21.3	-0.7
3C	1/30/2023 5:00	21	22.0	21.3	-0.7
3C	1/30/2023 6:00	21	22.0	21.3	-0.7
3C	1/30/2023 7:00	21	22.0	21.3	-0.7
3C	1/30/2023 8:00	21	22.0	17.8	-4.2
3C	1/30/2023 9:00	21	22.0	17.8	-4.2
3C	1/30/2023 10:00	21	24.0	17.8	-6.2
3C	1/30/2023 11:00	21	24.0	17.8	-6.2
3C	1/30/2023 12:00	21	24.0	20.3	-3.7
3C	1/30/2023 13:00	21	24.0	20.3	-3.7
3C	1/30/2023 14:00	21	24.0	20.3	-3.7
3C	1/30/2023 15:00	20	34.0	20.3	-13.7
3C	1/30/2023 16:00	20	34.0	18.7	-15.3
3C	1/30/2023 17:00	20	34.0	18.7	-15.3
3C	1/30/2023 18:00	20	34.0	18.7	-15.3
3C	1/30/2023 19:00	22	22.0	20.5	-1.5
3C	1/30/2023 20:00	22	22.0	16.4	-5.6
3C	1/30/2023 21:00	22	22.0	16.4	-5.6
3C	1/30/2023 22:00	22	22.0	16.4	-5.6
3C	1/30/2023 23:00	22	22.0	16.4	-5.6
3C	1/31/2023 0:00	22	22.0	20.0	-2.0
3C	1/31/2023 1:00	22	22.0	20.0	-2.0
3C	1/31/2023 2:00	22	22.0	20.0	-2.0
3C	1/31/2023 3:00	22	22.0	20.0	-2.0
3C	1/31/2023 4:00	22	22.0	20.0	-2.0
3C	1/31/2023 5:00	22	22.0	20.0	-2.0
3C	1/31/2023 6:00	22	22.0	20.0	-2.0
3C	1/31/2023 7:00	22	22.0	20.0	-2.0
3C	1/31/2023 8:00	22	22.0	17.8	-4.2
3C	1/31/2023 9:00	22	22.0	17.8	-4.2
3C	1/31/2023 10:00	22	22.0	17.8	-4.2
3C	1/31/2023 11:00	22	22.0	17.8	-4.2
3C	1/31/2023 12:00	22	22.0	25.4	3.4
3C	1/31/2023 13:00	22	22.0	25.4	3.4
3C	1/31/2023 14:00	22	22.0	25.4	3.4
3C	1/31/2023 15:00	22	22.0	25.4	3.4
3C	1/31/2023 16:00	22	22.0	29.3	7.3
3C	1/31/2023 17:00	22	22.0	29.3	7.3
3C	1/31/2023 18:00	22	22.0	29.3	7.3
3C	1/31/2023 19:00	22	22.0	29.3	7.3
3C	1/31/2023 20:00	22	22.0	23.4	1.4
3C	1/31/2023 21:00	22	22.0	23.4	1.4
3C	1/31/2023 22:00	22	22.0	23.4	1.4

3C	1/31/2023 23:00	22	22.0	23.4	1.4
3C	2/1/2023 0:00	22	28.0	20.0	-8.0
3C	2/1/2023 1:00	22	28.0	20.0	-8.0
3C	2/1/2023 2:00	22	28.0	20.0	-8.0
3C	2/1/2023 3:00	22	28.0	20.0	-8.0
3C	2/1/2023 4:00	22	28.0	20.0	-8.0
3C	2/1/2023 5:00	22	28.0	20.0	-8.0
3C	2/1/2023 6:00	22	28.0	20.0	-8.0
3C	2/1/2023 7:00	25	28.0	18.7	-9.3
3C	2/1/2023 8:00	25	30.0	20.3	-9.7
3C	2/1/2023 9:00	25	30.0	20.3	-9.7
3C	2/1/2023 10:00	25	30.0	20.3	-9.7
3C	2/1/2023 11:00	25	30.0	20.3	-9.7
3C	2/1/2023 12:00	25	30.0	22.9	-7.1
3C	2/1/2023 13:00	25	30.0	22.9	-7.1
3C	2/1/2023 14:00	25	30.0	22.9	-7.1
3C	2/1/2023 15:00	25	32.0	22.9	-9.1
3C	2/1/2023 16:00	25	32.0	20.5	-11.5
3C	2/1/2023 17:00	25	32.0	20.5	-11.5
3C	2/1/2023 18:00	25	32.0	20.5	-11.5
3C	2/1/2023 19:00	25	32.0	20.5	-11.5
3C	2/1/2023 20:00	25	32.0	23.4	-8.6
3C	2/1/2023 21:00	25	32.0	23.4	-8.6
3C	2/1/2023 22:00	25	32.0	23.4	-8.6
3C	2/1/2023 23:00	25	32.0	23.4	-8.6
3C	2/2/2023 0:00	25	30.0	26.7	-3.4
3C	2/2/2023 1:00	25	30.0	26.7	-3.4
3C	2/2/2023 2:00	25	28.0	26.7	-1.4
3C	2/2/2023 3:00	25	28.0	26.7	-1.4
3C	2/2/2023 4:00	25	28.0	26.7	-1.4
3C	2/2/2023 5:00	25	28.0	26.7	-1.4
3C	2/2/2023 6:00	25	28.0	26.7	-1.4
3C	2/2/2023 7:00	29	28.0	26.7	-1.4
3C	2/2/2023 8:00	29	28.0	22.9	-5.1
3C	2/2/2023 9:00	29	28.0	22.9	-5.1
3C	2/2/2023 10:00	29	28.0	22.9	-5.1
3C	2/2/2023 11:00	29	28.0	22.9	-5.1
3C	2/2/2023 12:00	29	28.0	22.9	-5.1
3C	2/2/2023 13:00	29	28.0	22.9	-5.1
3C	2/2/2023 14:00	29	28.0	22.9	-5.1
3C	2/2/2023 15:00	29	28.0	22.9	-5.1
3C	2/2/2023 16:00	29	28.0	26.4	-1.7
3C	2/2/2023 17:00	29	28.0	26.4	-1.7
3C	2/2/2023 18:00	29	28.0	26.4	-1.7
3C	2/2/2023 19:00	29	28.0	26.4	-1.7
3C	2/2/2023 20:00	29	28.0	23.4	-4.6
3C	2/2/2023 21:00	29	28.0	23.4	-4.6

3C	2/2/2023 22:00	29	28.0	23.4	-4.6
3C	2/2/2023 23:00	29	28.0	23.4	-4.6
3C	2/3/2023 0:00	29	29.0	23.3	-5.7
3C	2/3/2023 1:00	29	29.0	23.3	-5.7
3C	2/3/2023 2:00	29	29.0	23.3	-5.7
3C	2/3/2023 3:00	29	28.0	23.3	-4.7
3C	2/3/2023 4:00	29	28.0	23.3	-4.7
3C	2/3/2023 5:00	29	28.0	23.3	-4.7
3C	2/3/2023 6:00	29	28.0	23.3	-4.7
3C	2/3/2023 7:00	29	33.0	23.3	-9.7
3C	2/3/2023 8:00	29	33.0	20.3	-12.7
3C	2/3/2023 9:00	29	33.0	20.3	-12.7
3C	2/3/2023 10:00	29	33.0	20.3	-12.7
3C	2/3/2023 11:00	29	33.0	20.3	-12.7
3C	2/3/2023 12:00	29	33.0	22.9	-10.1
3C	2/3/2023 13:00	29	33.0	22.9	-10.1
3C	2/3/2023 14:00	29	33.0	22.9	-10.1
3C	2/3/2023 15:00	23	29.0	22.9	-6.1
3C	2/3/2023 16:00	23	29.0	23.4	-5.6
3C	2/3/2023 17:00	23	29.0	23.4	-5.6
3C	2/3/2023 18:00	23	29.0	23.4	-5.6
3C	2/3/2023 19:00	21	29.0	23.4	-5.6
3C	2/3/2023 20:00	21	29.0	23.4	-5.6
3C	2/3/2023 21:00	21	29.0	23.4	-5.6
3C	2/3/2023 22:00	21	29.0	23.4	-5.6
3C	2/3/2023 23:00	21	29.0	23.4	-5.6
3C	2/4/2023 0:00	21	29.0	23.3	-5.7
3C	2/4/2023 1:00	21	29.0	23.3	-5.7
3C	2/4/2023 2:00	21	30.0	23.3	-6.7
3C	2/4/2023 3:00	21	29.0	23.3	-5.7
3C	2/4/2023 4:00	21	29.0	23.3	-5.7
3C	2/4/2023 5:00	21	29.0	23.3	-5.7
3C	2/4/2023 6:00	21	29.0	23.3	-5.7
3C	2/4/2023 7:00	21	29.0	23.3	-5.7
3C	2/4/2023 8:00	23	29.0	16.3	-12.7
3C	2/4/2023 9:00	23	29.0	16.3	-12.7
3C	2/4/2023 10:00	23	29.0	16.3	-12.7
3C	2/4/2023 11:00	23	29.0	16.3	-12.7
3C	2/4/2023 12:00	23	29.0	21.4	-7.6
3C	2/4/2023 13:00	23	29.0	22.4	-6.6
3C	2/4/2023 14:00	23	29.0	22.4	-6.6
3C	2/4/2023 15:00	18	30.0	22.4	-7.6
3C	2/4/2023 16:00	18	30.0	25.8	-4.2
3C	2/4/2023 17:00	18	30.0	25.8	-4.2
3C	2/4/2023 18:00	18	30.0	25.8	-4.2
3C	2/4/2023 19:00	20	30.0	23.4	-6.6
3C	2/4/2023 20:00	20	30.0	23.4	-6.6

3C	2/4/2023 21:00	20	30.0	23.4	-6.6
3C	2/4/2023 22:00	20	30.0	23.4	-6.6
3C	2/4/2023 23:00	20	30.0	23.4	-6.6
3C	2/5/2023 0:00	20	29.0	24.0	-5.0
3C	2/5/2023 1:00	20	29.0	24.0	-5.0
3C	2/5/2023 2:00	20	30.0	24.0	-6.0
3C	2/5/2023 3:00	20	30.0	24.0	-6.0
3C	2/5/2023 4:00	20	30.0	24.0	-6.0
3C	2/5/2023 5:00	20	30.0	24.0	-6.0
3C	2/5/2023 6:00	20	30.0	24.0	-6.0
3C	2/5/2023 7:00	20	30.0	24.0	-6.0
3C	2/5/2023 8:00	20	30.0	14.2	-15.8
3C	2/5/2023 9:00	20	30.0	14.2	-15.8
3C	2/5/2023 10:00	20	24.0	14.2	-9.8
3C	2/5/2023 11:00	20	24.0	14.2	-9.8
3C	2/5/2023 12:00	20	24.0	18.3	-5.7
3C	2/5/2023 13:00	20	24.0	18.3	-5.7
3C	2/5/2023 14:00	20	24.0	18.3	-5.7
3C	2/5/2023 15:00	18	26.0	18.3	-7.7
3C	2/5/2023 16:00	18	26.0	23.4	-2.6
3C	2/5/2023 17:00	18	26.0	23.4	-2.6
3C	2/5/2023 18:00	18	26.0	23.4	-2.6
3C	2/5/2023 19:00	19	30.0	23.4	-6.6
3C	2/5/2023 20:00	19	30.0	21.1	-8.9
3C	2/5/2023 21:00	20	30.0	21.1	-8.9
3C	2/5/2023 22:00	20	30.0	21.1	-8.9
3C	2/5/2023 23:00	20	30.0	21.1	-8.9
3C	2/6/2023 0:00	20	30.0	26.7	-3.4
3C	2/6/2023 1:00	21	30.0	26.7	-3.4
3C	2/6/2023 2:00	21	27.0	26.7	-0.4
3C	2/6/2023 3:00	21	30.0	26.7	-3.4
3C	2/6/2023 4:00	21	27.0	26.7	-0.4
3C	2/6/2023 5:00	21	27.0	26.7	-0.4
3C	2/6/2023 6:00	21	27.0	26.7	-0.4
3C	2/6/2023 7:00	21	27.0	26.7	-0.4
3C	2/6/2023 8:00	21	27.0	15.3	-11.7
3C	2/6/2023 9:00	21	27.0	15.3	-11.7
3C	2/6/2023 10:00	21	27.0	15.3	-11.7
3C	2/6/2023 11:00	21	27.0	15.3	-11.7
3C	2/6/2023 12:00	23	27.0	17.8	-9.2
3C	2/6/2023 13:00	23	27.0	17.8	-9.2
3C	2/6/2023 14:00	23	27.0	17.8	-9.2
3C	2/6/2023 15:00	23	27.0	17.8	-9.2
3C	2/6/2023 16:00	23	27.0	16.4	-10.6
3C	2/6/2023 17:00	23	27.0	16.4	-10.6
3C	2/6/2023 18:00	23	27.0	16.4	-10.6
3C	2/6/2023 19:00	23	27.0	16.4	-10.6

3C	2/6/2023 20:00	24	27.0	18.7	-8.3
3C	2/6/2023 21:00	24	27.0	18.7	-8.3
3C	2/6/2023 22:00	24	27.0	18.7	-8.3
3C	2/6/2023 23:00	24	27.0	18.7	-8.3
3C	2/7/2023 0:00	24	27.0	22.7	-4.4
3C	2/7/2023 1:00	24	27.0	24.0	-3.0
3C	2/7/2023 2:00	24	27.0	24.0	-3.0
3C	2/7/2023 3:00	24	27.0	24.0	-3.0
3C	2/7/2023 4:00	24	27.0	24.0	-3.0
3C	2/7/2023 5:00	24	27.0	24.0	-3.0
3C	2/7/2023 6:00	24	30.0	24.0	-6.0
3C	2/7/2023 7:00	24	30.0	24.0	-6.0
3C	2/7/2023 8:00	24	30.0	20.3	-9.7
3C	2/7/2023 9:00	24	30.0	20.3	-9.7
3C	2/7/2023 10:00	24	30.0	20.3	-9.7
3C	2/7/2023 11:00	24	30.0	20.3	-9.7
3C	2/7/2023 12:00	24	30.0	25.4	-4.6
3C	2/7/2023 13:00	24	30.0	25.4	-4.6
3C	2/7/2023 14:00	24	30.0	22.9	-7.1
3C	2/7/2023 15:00	18	34.0	22.9	-11.1
3C	2/7/2023 16:00	16	34.0	23.4	-10.6
3C	2/7/2023 17:00	16	34.0	23.4	-10.6
3C	2/7/2023 18:00	16	34.0	23.4	-10.6
3C	2/7/2023 19:00	16	34.0	23.4	-10.6
3C	2/7/2023 20:00	16	34.0	28.1	-5.9
3C	2/7/2023 21:00	16	34.0	28.1	-5.9
3C	2/7/2023 22:00	16	34.0	28.1	-5.9
3C	2/7/2023 23:00	16	34.0	28.1	-5.9
3C	2/8/2023 0:00	16	34.0	26.7	-7.4
3C	2/8/2023 1:00	16	34.0	26.7	-7.4
3C	2/8/2023 2:00	16	34.0	26.7	-7.4
3C	2/8/2023 3:00	23	34.0	26.7	-7.4
3C	2/8/2023 4:00	23	34.0	26.7	-7.4
3C	2/8/2023 5:00	23	34.0	26.7	-7.4
3C	2/8/2023 6:00	23	34.0	26.7	-7.4
3C	2/8/2023 7:00	23	34.0	29.3	-4.7
3C	2/8/2023 8:00	23	34.0	17.8	-16.2
3C	2/8/2023 9:00	23	34.0	17.8	-16.2
3C	2/8/2023 10:00	23	34.0	17.8	-16.2
3C	2/8/2023 11:00	21	34.0	17.8	-16.2
3C	2/8/2023 12:00	21	34.0	25.4	-8.6
3C	2/8/2023 13:00	21	34.0	25.4	-8.6
3C	2/8/2023 14:00	21	34.0	25.4	-8.6
3C	2/8/2023 15:00	21	34.0	25.4	-8.6
3C	2/8/2023 16:00	21	34.0	25.8	-8.2
3C	2/8/2023 17:00	22	34.0	25.8	-8.2
3C	2/8/2023 18:00	22	34.0	25.8	-8.2

3C	2/8/2023 19:00	22	34.0	25.8	-8.2
3C	2/8/2023 20:00	22	34.0	25.8	-8.2
3C	2/8/2023 21:00	22	34.0	25.8	-8.2
3C	2/8/2023 22:00	22	34.0	25.8	-8.2
3C	2/8/2023 23:00	22	34.0	25.8	-8.2
3C	2/9/2023 0:00	22	34.0	29.3	-4.7
3C	2/9/2023 1:00	22	34.0	29.3	-4.7
3C	2/9/2023 2:00	22	33.0	29.3	-3.7
3C	2/9/2023 3:00	22	33.0	29.3	-3.7
3C	2/9/2023 4:00	22	33.0	29.3	-3.7
3C	2/9/2023 5:00	25	33.0	26.7	-6.4
3C	2/9/2023 6:00	25	33.0	26.7	-6.4
3C	2/9/2023 7:00	25	33.0	26.7	-6.4
3C	2/9/2023 8:00	25	33.0	20.3	-12.7
3C	2/9/2023 9:00	25	33.0	20.3	-12.7
3C	2/9/2023 10:00	25	33.0	20.3	-12.7
3C	2/9/2023 11:00	25	33.0	22.9	-10.1
3C	2/9/2023 12:00	25	33.0	20.3	-12.7
3C	2/9/2023 13:00	25	33.0	20.3	-12.7
3C	2/9/2023 14:00	25	33.0	20.3	-12.7
3C	2/9/2023 15:00	25	33.0	20.3	-12.7
3C	2/9/2023 16:00	25	33.0	20.5	-12.5
3C	2/9/2023 17:00	25	33.0	20.5	-12.5
3C	2/9/2023 18:00	25	33.0	20.5	-12.5
3C	2/9/2023 19:00	25	33.0	20.5	-12.5
3C	2/9/2023 20:00	25	33.0	25.8	-7.2
3C	2/9/2023 21:00	25	33.0	25.8	-7.2
3C	2/9/2023 22:00	25	33.0	25.8	-7.2
3C	2/9/2023 23:00	25	33.0	25.8	-7.2
3C	2/10/2023 0:00	25	33.0	29.3	-3.7
3C	2/10/2023 1:00	25	33.0	29.3	-3.7
3C	2/10/2023 2:00	25	33.0	29.3	-3.7
3C	2/10/2023 3:00	25	33.0	29.3	-3.7
3C	2/10/2023 4:00	25	33.0	29.3	-3.7
3C	2/10/2023 5:00	25	33.0	29.3	-3.7
3C	2/10/2023 6:00	25	33.0	29.3	-3.7
3C	2/10/2023 7:00	25	33.0	29.3	-3.7
3C	2/10/2023 8:00	25	33.0	20.3	-12.7
3C	2/10/2023 9:00	25	33.0	20.3	-12.7
3C	2/10/2023 10:00	25	33.0	20.3	-12.7
3C	2/10/2023 11:00	25	33.0	20.3	-12.7
3C	2/10/2023 12:00	25	33.0	22.9	-10.1
3C	2/10/2023 13:00	25	33.0	22.9	-10.1
3C	2/10/2023 14:00	25	33.0	22.9	-10.1
3C	2/10/2023 15:00	19	33.0	22.9	-10.1
3C	2/10/2023 16:00	19	33.0	23.4	-9.6
3C	2/10/2023 17:00	19	33.0	23.4	-9.6

3C	2/10/2023 18:00	19	33.0	23.4	-9.6
3C	2/10/2023 19:00	19	33.0	23.4	-9.6
3C	2/10/2023 20:00	19	33.0	23.4	-9.6
3C	2/10/2023 21:00	19	33.0	23.4	-9.6
3C	2/10/2023 22:00	19	33.0	23.4	-9.6
3C	2/10/2023 23:00	19	33.0	23.4	-9.6
3C	2/11/2023 0:00	19	33.0	23.3	-9.7
3C	2/11/2023 1:00	19	33.0	23.3	-9.7
3C	2/11/2023 2:00	19	33.0	23.3	-9.7
3C	2/11/2023 3:00	19	33.0	23.3	-9.7
3C	2/11/2023 4:00	19	33.0	23.3	-9.7
3C	2/11/2023 5:00	19	33.0	23.3	-9.7
3C	2/11/2023 6:00	19	33.0	23.3	-9.7
3C	2/11/2023 7:00	19	33.0	23.3	-9.7
3C	2/11/2023 8:00	19	33.0	17.8	-15.2
3C	2/11/2023 9:00	19	33.0	17.8	-15.2
3C	2/11/2023 10:00	19	33.0	17.8	-15.2
3C	2/11/2023 11:00	19	33.0	17.8	-15.2
3C	2/11/2023 12:00	19	33.0	22.9	-10.1
3C	2/11/2023 13:00	19	33.0	22.9	-10.1
3C	2/11/2023 14:00	19	33.0	22.9	-10.1
3C	2/11/2023 15:00	30	33.0	22.9	-10.1
3C	2/11/2023 16:00	30	33.0	26.4	-6.7
3C	2/11/2023 17:00	30	33.0	26.4	-6.7
3C	2/11/2023 18:00	30	33.0	26.4	-6.7
3C	2/11/2023 19:00	30	33.0	24.9	-8.1
3C	2/11/2023 20:00	30	33.0	23.4	-9.6
3C	2/11/2023 21:00	30	33.0	23.4	-9.6
3C	2/11/2023 22:00	15	33.0	23.4	-9.6
3C	2/11/2023 23:00	15	33.0	23.4	-9.6
3C	2/12/2023 0:00	15	30.0	23.3	-6.7
3C	2/12/2023 1:00	15	30.0	23.3	-6.7
3C	2/12/2023 2:00	15	30.0	23.3	-6.7
3C	2/12/2023 3:00	15	30.0	23.3	-6.7
3C	2/12/2023 4:00	15	30.0	23.3	-6.7
3C	2/12/2023 5:00	15	30.0	23.3	-6.7
3C	2/12/2023 6:00	15	30.0	23.3	-6.7
3C	2/12/2023 7:00	15	30.0	23.3	-6.7
3C	2/12/2023 8:00	15	30.0	18.3	-11.7
3C	2/12/2023 9:00	15	30.0	18.3	-11.7
3C	2/12/2023 10:00	15	30.0	19.3	-10.7
3C	2/12/2023 11:00	15	30.0	20.3	-9.7
3C	2/12/2023 12:00	15	30.0	24.4	-5.6
3C	2/12/2023 13:00	15	30.0	24.4	-5.6
3C	2/12/2023 14:00	15	30.0	24.4	-5.6
3C	2/12/2023 15:00	15	30.0	24.4	-5.6
3C	2/12/2023 16:00	15	30.0	32.2	2.2

3C	2/12/2023 17:00	15	30.0	32.2	2.2
3C	2/12/2023 18:00	15	30.0	32.2	2.2
3C	2/12/2023 19:00	15	30.0	32.2	2.2
3C	2/12/2023 20:00	15	30.0	21.1	-8.9
3C	2/12/2023 21:00	15	30.0	21.1	-8.9
3C	2/12/2023 22:00	15	30.0	21.1	-8.9
3C	2/12/2023 23:00	15	30.0	21.1	-8.9
3C	2/13/2023 0:00	15	30.0	21.3	-8.7
3C	2/13/2023 1:00	15	30.0	21.3	-8.7
3C	2/13/2023 2:00	15	32.0	21.3	-10.7
3C	2/13/2023 3:00	15	32.0	21.3	-10.7
3C	2/13/2023 4:00	15	32.0	20.0	-12.0
3C	2/13/2023 5:00	15	32.0	18.7	-13.3
3C	2/13/2023 6:00	15	32.0	18.7	-13.3
3C	2/13/2023 7:00	19	32.0	21.3	-10.7
3C	2/13/2023 8:00	19	32.0	17.8	-14.2
3C	2/13/2023 9:00	19	32.0	17.8	-14.2
3C	2/13/2023 10:00	19	32.0	17.8	-14.2
3C	2/13/2023 11:00	19	32.0	17.8	-14.2
3C	2/13/2023 12:00	19	32.0	20.3	-11.7
3C	2/13/2023 13:00	19	32.0	20.3	-11.7
3C	2/13/2023 14:00	19	32.0	20.3	-11.7
3C	2/13/2023 15:00	18	32.0	15.3	-16.7
3C	2/13/2023 16:00	18	32.0	17.6	-14.4
3C	2/13/2023 17:00	18	32.0	17.6	-14.4
3C	2/13/2023 18:00	18	32.0	17.6	-14.4
3C	2/13/2023 19:00	20	32.0	21.1	-10.9
3C	2/13/2023 20:00	20	32.0	25.8	-6.2
3C	2/13/2023 21:00	20	32.0	25.8	-6.2
3C	2/13/2023 22:00	20	32.0	25.8	-6.2
3C	2/13/2023 23:00	20	32.0	25.8	-6.2
3C	2/14/2023 0:00	24	32.0	20.0	-12.0
3C	2/14/2023 1:00	24	32.0	20.0	-12.0
3C	2/14/2023 2:00	24	32.0	20.0	-12.0
3C	2/14/2023 3:00	24	32.0	20.0	-12.0
3C	2/14/2023 4:00	24	32.0	20.0	-12.0
3C	2/14/2023 5:00	24	32.0	20.0	-12.0
3C	2/14/2023 6:00	24	32.0	20.0	-12.0
3C	2/14/2023 7:00	24	32.0	20.0	-12.0
3C	2/14/2023 8:00	24	32.0	12.7	-19.3
3C	2/14/2023 9:00	24	32.0	12.7	-19.3
3C	2/14/2023 10:00	24	32.0	12.7	-19.3
3C	2/14/2023 11:00	24	32.0	12.7	-19.3
3C	2/14/2023 12:00	24	32.0	17.8	-14.2
3C	2/14/2023 13:00	24	32.0	17.8	-14.2
3C	2/14/2023 14:00	24	32.0	17.8	-14.2
3C	2/14/2023 15:00	21	32.0	20.3	-11.7

3C	2/14/2023 16:00	21	32.0	23.4	-8.6
3C	2/14/2023 17:00	21	32.0	23.4	-8.6
3C	2/14/2023 18:00	21	32.0	23.4	-8.6
3C	2/14/2023 19:00	23	32.0	23.4	-8.6
3C	2/14/2023 20:00	23	32.0	26.4	-5.7
3C	2/14/2023 21:00	23	32.0	26.4	-5.7
3C	2/14/2023 22:00	23	32.0	26.4	-5.7
3C	2/14/2023 23:00	23	32.0	26.4	-5.7
3C	2/15/2023 0:00	23	30.0	26.7	-3.4
3C	2/15/2023 1:00	23	30.0	29.3	-0.7
3C	2/15/2023 2:00	23	28.0	29.3	1.3
3C	2/15/2023 3:00	23	28.0	29.3	1.3
3C	2/15/2023 4:00	23	28.0	26.7	-1.4
3C	2/15/2023 5:00	23	28.0	26.7	-1.4
3C	2/15/2023 6:00	23	28.0	26.7	-1.4
3C	2/15/2023 7:00	23	28.0	26.7	-1.4
3C	2/15/2023 8:00	23	28.0	25.4	-2.6
3C	2/15/2023 9:00	23	28.0	25.4	-2.6
3C	2/15/2023 10:00	23	30.0	25.4	-4.6
3C	2/15/2023 11:00	23	30.0	25.4	-4.6
3C	2/15/2023 12:00	23	30.0	28.0	-2.0
3C	2/15/2023 13:00	23	30.0	28.0	-2.0
3C	2/15/2023 14:00	23	30.0	28.0	-2.0
3C	2/15/2023 15:00	23	30.0	28.0	-2.0
3C	2/15/2023 16:00	23	30.0	23.4	-6.6
3C	2/15/2023 17:00	23	30.0	23.4	-6.6
3C	2/15/2023 18:00	23	30.0	23.4	-6.6
3C	2/15/2023 19:00	21	28.0	26.4	-1.7
3C	2/15/2023 20:00	21	28.0	23.4	-4.6
3C	2/15/2023 21:00	21	28.0	23.4	-4.6
3C	2/15/2023 22:00	21	28.0	23.4	-4.6
3C	2/15/2023 23:00	21	28.0	23.4	-4.6
3C	2/16/2023 0:00	21	28.0	26.7	-1.4
3C	2/16/2023 1:00	21	28.0	26.7	-1.4
3C	2/16/2023 2:00	21	28.0	26.7	-1.4
3C	2/16/2023 3:00	21	28.0	26.7	-1.4
3C	2/16/2023 4:00	21	28.0	26.7	-1.4
3C	2/16/2023 5:00	21	28.0	26.7	-1.4
3C	2/16/2023 6:00	21	28.0	26.7	-1.4
3C	2/16/2023 7:00	21	28.0	21.3	-6.7
3C	2/16/2023 8:00	21	28.0	15.3	-12.7
3C	2/16/2023 9:00	21	28.0	15.3	-12.7
3C	2/16/2023 10:00	21	29.0	15.3	-13.7
3C	2/16/2023 11:00	21	29.0	15.3	-13.7
3C	2/16/2023 12:00	21	29.0	22.4	-6.6
3C	2/16/2023 13:00	21	29.0	22.4	-6.6
3C	2/16/2023 14:00	21	29.0	22.4	-6.6

3C	2/16/2023 15:00	21	29.0	22.4	-6.6
3C	2/16/2023 16:00	21	29.0	18.7	-10.3
3C	2/16/2023 17:00	21	29.0	18.7	-10.3
3C	2/16/2023 18:00	21	29.0	18.7	-10.3
3C	2/16/2023 19:00	21	29.0	18.7	-10.3
3C	2/16/2023 20:00	21	29.0	23.4	-5.6
3C	2/16/2023 21:00	21	29.0	23.4	-5.6
3C	2/16/2023 22:00	21	29.0	23.4	-5.6
3C	2/16/2023 23:00	21	29.0	23.4	-5.6
3C	2/17/2023 0:00	21	28.0	21.3	-6.7
3C	2/17/2023 1:00	21	28.0	21.3	-6.7
3C	2/17/2023 2:00	21	30.0	21.3	-8.7
3C	2/17/2023 3:00	21	30.0	21.3	-8.7
3C	2/17/2023 4:00	21	30.0	21.3	-8.7
3C	2/17/2023 5:00	18	30.0	21.3	-8.7
3C	2/17/2023 6:00	18	30.0	21.3	-8.7
3C	2/17/2023 7:00	18	30.0	21.3	-8.7
3C	2/17/2023 8:00	18	30.0	18.3	-11.7
3C	2/17/2023 9:00	18	30.0	18.3	-11.7
3C	2/17/2023 10:00	18	30.0	18.3	-11.7
3C	2/17/2023 11:00	18	30.0	18.3	-11.7
3C	2/17/2023 12:00	18	30.0	18.3	-11.7
3C	2/17/2023 13:00	18	30.0	18.3	-11.7
3C	2/17/2023 14:00	18	30.0	18.3	-11.7
3C	2/17/2023 15:00	14	30.0	20.3	-9.7
3C	2/17/2023 16:00	14	30.0	20.5	-9.5
3C	2/17/2023 17:00	14	30.0	20.5	-9.5
3C	2/17/2023 18:00	14	30.0	20.5	-9.5
3C	2/17/2023 19:00	14	30.0	20.5	-9.5
3C	2/17/2023 20:00	14	30.0	28.1	-1.9
3C	2/17/2023 21:00	14	30.0	28.1	-1.9
3C	2/17/2023 22:00	14	30.0	28.1	-1.9
3C	2/17/2023 23:00	14	30.0	28.1	-1.9
3C	2/18/2023 0:00	14	30.0	26.7	-3.4
3C	2/18/2023 1:00	14	30.0	26.7	-3.4
3C	2/18/2023 2:00	14	30.0	26.7	-3.4
3C	2/18/2023 3:00	14	30.0	26.7	-3.4
3C	2/18/2023 4:00	14	30.0	26.7	-3.4
3C	2/18/2023 5:00	14	30.0	26.7	-3.4
3C	2/18/2023 6:00	14	30.0	26.7	-3.4
3C	2/18/2023 7:00	16	30.0	26.7	-3.4
3C	2/18/2023 8:00	16	30.0	24.4	-5.6
3C	2/18/2023 9:00	16	30.0	24.4	-5.6
3C	2/18/2023 10:00	16	30.0	24.4	-5.6
3C	2/18/2023 11:00	16	30.0	25.4	-4.6
3C	2/18/2023 12:00	16	30.0	25.4	-4.6
3C	2/18/2023 13:00	16	30.0	25.4	-4.6

3C	2/18/2023 14:00	16	30.0	25.4	-4.6
3C	2/18/2023 15:00	15	30.0	24.4	-5.6
3C	2/18/2023 16:00	15	30.0	23.4	-6.6
3C	2/18/2023 17:00	15	30.0	23.4	-6.6
3C	2/18/2023 18:00	15	30.0	23.4	-6.6
3C	2/18/2023 19:00	15	30.0	23.4	-6.6
3C	2/18/2023 20:00	15	30.0	18.7	-11.3
3C	2/18/2023 21:00	15	30.0	18.7	-11.3
3C	2/18/2023 22:00	15	30.0	18.7	-11.3
3C	2/18/2023 23:00	15	30.0	18.7	-11.3
3C	2/19/2023 0:00	15	30.0	24.0	-6.0
3C	2/19/2023 1:00	15	30.0	24.0	-6.0
3C	2/19/2023 2:00	15	30.0	24.0	-6.0
3C	2/19/2023 3:00	15	30.0	24.0	-6.0
3C	2/19/2023 4:00	15	30.0	24.0	-6.0
3C	2/19/2023 5:00	15	30.0	24.0	-6.0
3C	2/19/2023 6:00	15	30.0	24.0	-6.0
3C	2/19/2023 7:00	15	30.0	24.0	-6.0
3C	2/19/2023 8:00	15	30.0	18.3	-11.7
3C	2/19/2023 9:00	15	30.0	18.3	-11.7
3C	2/19/2023 10:00	15	30.0	18.3	-11.7
3C	2/19/2023 11:00	15	30.0	18.3	-11.7
3C	2/19/2023 12:00	15	30.0	18.3	-11.7
3C	2/19/2023 13:00	15	30.0	18.3	-11.7
3C	2/19/2023 14:00	15	30.0	18.3	-11.7
3C	2/19/2023 15:00	16	30.0	18.3	-11.7
3C	2/19/2023 16:00	16	30.0	23.4	-6.6
3C	2/19/2023 17:00	16	30.0	23.4	-6.6
3C	2/19/2023 18:00	15	30.0	25.8	-4.2
3C	2/19/2023 19:00	15	30.0	25.8	-4.2
3C	2/19/2023 20:00	15	30.0	18.7	-11.3
3C	2/19/2023 21:00	15	30.0	18.7	-11.3
3C	2/19/2023 22:00	15	30.0	18.7	-11.3
3C	2/19/2023 23:00	15	30.0	18.7	-11.3
3C	2/20/2023 0:00	15	30.0	18.7	-11.3
3C	2/20/2023 1:00	15	30.0	18.7	-11.3
3C	2/20/2023 2:00	15	30.0	18.7	-11.3
3C	2/20/2023 3:00	15	30.0	18.7	-11.3
3C	2/20/2023 4:00	15	30.0	18.7	-11.3
3C	2/20/2023 5:00	15	30.0	18.7	-11.3
3C	2/20/2023 6:00	15	30.0	18.7	-11.3
3C	2/20/2023 7:00	15	30.0	18.7	-11.3
3C	2/20/2023 8:00	15	30.0	18.3	-11.7
3C	2/20/2023 9:00	15	30.0	18.3	-11.7
3C	2/20/2023 10:00	15	30.0	18.3	-11.7
3C	2/20/2023 11:00	15	30.0	18.3	-11.7
3C	2/20/2023 12:00	15	30.0	20.3	-9.7

3C	2/20/2023 13:00	15	30.0	20.3	-9.7
3C	2/20/2023 14:00	15	30.0	20.3	-9.7
3C	2/20/2023 15:00	15	30.0	20.3	-9.7
3C	2/20/2023 16:00	15	30.0	23.4	-6.6
3C	2/20/2023 17:00	15	30.0	23.4	-6.6
3C	2/20/2023 18:00	15	30.0	20.5	-9.5
3C	2/20/2023 19:00	15	30.0	20.5	-9.5
3C	2/20/2023 20:00	15	23.0	18.7	-4.3
3C	2/20/2023 21:00	15	28.0	18.7	-9.3
3C	2/20/2023 22:00	15	28.0	18.7	-9.3
3C	2/20/2023 23:00	15	28.0	18.7	-9.3
3C	2/21/2023 0:00	15	28.0	21.3	-6.7
3C	2/21/2023 1:00	15	28.0	21.3	-6.7
3C	2/21/2023 2:00	15	28.0	21.3	-6.7
3C	2/21/2023 3:00	15	28.0	21.3	-6.7
3C	2/21/2023 4:00	15	28.0	21.3	-6.7
3C	2/21/2023 5:00	15	30.0	21.3	-8.7
3C	2/21/2023 6:00	15	30.0	21.3	-8.7
3C	2/21/2023 7:00	15	30.0	21.3	-8.7
3C	2/21/2023 8:00	15	30.0	17.8	-12.2
3C	2/21/2023 9:00	15	30.0	17.8	-12.2
3C	2/21/2023 10:00	15	30.0	17.8	-12.2
3C	2/21/2023 11:00	15	30.0	17.8	-12.2
3C	2/21/2023 12:00	15	30.0	22.9	-7.1
3C	2/21/2023 13:00	12	30.0	22.9	-7.1
3C	2/21/2023 14:00	12	30.0	22.9	-7.1
3C	2/21/2023 15:00	12	30.0	21.6	-8.4
3C	2/21/2023 16:00	11	30.0	20.5	-9.5
3C	2/21/2023 17:00	11	30.0	20.5	-9.5
3C	2/21/2023 18:00	11	30.0	20.5	-9.5
3C	2/21/2023 19:00	12	30.0	20.5	-9.5
3C	2/21/2023 20:00	12	30.0	17.6	-12.4
3C	2/21/2023 21:00	12	30.0	17.6	-12.4
3C	2/21/2023 22:00	12	30.0	17.6	-12.4
3C	2/21/2023 23:00	12	30.0	17.6	-12.4
3C	2/22/2023 0:00	12	30.0	15.0	-15.0
3C	2/22/2023 1:00	12	30.0	16.7	-13.3
3C	2/22/2023 2:00	12	24.0	16.7	-7.3
3C	2/22/2023 3:00	12	24.0	16.7	-7.3
3C	2/22/2023 4:00	12	24.0	16.7	-7.3
3C	2/22/2023 5:00	13	24.0	16.7	-7.3
3C	2/22/2023 6:00	13	24.0	16.7	-7.3
3C	2/22/2023 7:00	13	24.0	16.7	-7.3
3C	2/22/2023 8:00	13	24.0	22.4	-1.6
3C	2/22/2023 9:00	13	24.0	22.4	-1.6
3C	2/22/2023 10:00	13	30.0	22.4	-7.6
3C	2/22/2023 11:00	13	30.0	22.4	-7.6

3C	2/22/2023 12:00	13	30.0	26.7	-3.3
3C	2/22/2023 13:00	13	30.0	28.0	-2.0
3C	2/22/2023 14:00	13	30.0	28.0	-2.0
3C	2/22/2023 15:00	13	30.0	28.0	-2.0
3C	2/22/2023 16:00	15	24.0	23.4	-0.6
3C	2/22/2023 17:00	15	24.0	23.4	-0.6
3C	2/22/2023 18:00	15	24.0	23.4	-0.6
3C	2/22/2023 19:00	18	24.0	23.4	-0.6
3C	2/22/2023 20:00	18	24.0	17.6	-6.4
3C	2/22/2023 21:00	18	24.0	17.6	-6.4
3C	2/22/2023 22:00	18	24.0	17.6	-6.4
3C	2/22/2023 23:00	18	24.0	17.6	-6.4
3C	2/23/2023 0:00	18	24.0	23.3	-0.7
3C	2/23/2023 1:00	18	24.0	23.3	-0.7
3C	2/23/2023 2:00	18	29.0	23.3	-5.7
3C	2/23/2023 3:00	18	29.0	23.3	-5.7
3C	2/23/2023 4:00	18	29.0	23.3	-5.7
3C	2/23/2023 5:00	21	29.0	23.3	-5.7
3C	2/23/2023 6:00	21	29.0	23.3	-5.7
3C	2/23/2023 7:00	21	29.0	23.3	-5.7
3C	2/23/2023 8:00	21	29.0	20.3	-8.7
3C	2/23/2023 9:00	21	29.0	20.3	-8.7
3C	2/23/2023 10:00	21	29.0	20.3	-8.7
3C	2/23/2023 11:00	21	29.0	20.3	-8.7
3C	2/23/2023 12:00	21	29.0	20.3	-8.7
3C	2/23/2023 13:00	21	29.0	20.3	-8.7
3C	2/23/2023 14:00	21	29.0	20.3	-8.7
3C	2/23/2023 15:00	21	29.0	20.3	-8.7
3C	2/23/2023 16:00	21	29.0	23.4	-5.6
3C	2/23/2023 17:00	21	29.0	23.4	-5.6
3C	2/23/2023 18:00	21	29.0	23.4	-5.6
3C	2/23/2023 19:00	23	29.0	23.4	-5.6
3C	2/23/2023 20:00	23	29.0	23.4	-5.6
3C	2/23/2023 21:00	23	29.0	23.4	-5.6
3C	2/23/2023 22:00	23	29.0	23.4	-5.6
3C	2/23/2023 23:00	23	29.0	23.4	-5.6
3C	2/24/2023 0:00	23	29.0	23.3	-5.7
3C	2/24/2023 1:00	24	29.0	23.3	-5.7
3C	2/24/2023 2:00	24	29.0	23.3	-5.7
3C	2/24/2023 3:00	24	29.0	23.3	-5.7
3C	2/24/2023 4:00	24	29.0	23.3	-5.7
3C	2/24/2023 5:00	24	29.0	23.3	-5.7
3C	2/24/2023 6:00	24	29.0	23.3	-5.7
3C	2/24/2023 7:00	24	29.0	23.3	-5.7
3C	2/24/2023 8:00	24	29.0	20.3	-8.7
3C	2/24/2023 9:00	24	29.0	20.3	-8.7
3C	2/24/2023 10:00	21	28.0	20.3	-7.7

3C	2/24/2023 11:00	21	28.0	20.3	-7.7
3C	2/24/2023 12:00	21	28.0	20.3	-7.7
3C	2/24/2023 13:00	21	28.0	20.3	-7.7
3C	2/24/2023 14:00	21	28.0	20.3	-7.7
3C	2/24/2023 15:00	21	28.0	20.3	-7.7
3C	2/24/2023 16:00	21	28.0	17.6	-10.4
3C	2/24/2023 17:00	21	28.0	17.6	-10.4
3C	2/24/2023 18:00	21	28.0	17.6	-10.4
3C	2/24/2023 19:00	18	28.0	17.6	-10.4
3C	2/24/2023 20:00	18	28.0	20.5	-7.5
3C	2/24/2023 21:00	18	28.0	20.5	-7.5
3C	2/24/2023 22:00	18	28.0	20.5	-7.5
3C	2/24/2023 23:00	18	28.0	20.5	-7.5
3C	2/25/2023 0:00	17	28.0	23.3	-4.7
3C	2/25/2023 1:00	17	28.0	23.3	-4.7
3C	2/25/2023 2:00	17	28.0	23.3	-4.7
3C	2/25/2023 3:00	17	28.0	23.3	-4.7
3C	2/25/2023 4:00	17	28.0	23.3	-4.7
3C	2/25/2023 5:00	17	28.0	23.3	-4.7
3C	2/25/2023 6:00	17	28.0	23.3	-4.7
3C	2/25/2023 7:00	17	28.0	23.3	-4.7
3C	2/25/2023 8:00	17	28.0	22.4	-5.6
3C	2/25/2023 9:00	17	28.0	22.4	-5.6
3C	2/25/2023 10:00	17	28.0	22.4	-5.6
3C	2/25/2023 11:00	17	28.0	22.4	-5.6
3C	2/25/2023 12:00	17	28.0	26.4	-1.6
3C	2/25/2023 13:00	18	28.0	24.4	-3.6
3C	2/25/2023 14:00	18	28.0	24.4	-3.6
3C	2/25/2023 15:00	18	28.0	24.4	-3.6
3C	2/25/2023 16:00	18	28.0	26.4	-1.7
3C	2/25/2023 17:00	18	28.0	26.4	-1.7
3C	2/25/2023 18:00	18	28.0	26.4	-1.7
3C	2/25/2023 19:00	18	28.0	26.4	-1.7
3C	2/25/2023 20:00	18	28.0	28.1	0.1
3C	2/25/2023 21:00	18	28.0	28.1	0.1
3C	2/25/2023 22:00	18	28.0	28.1	0.1
3C	2/25/2023 23:00	18	28.0	30.5	2.5
3C	2/26/2023 0:00	18	28.0	29.3	1.3
3C	2/26/2023 1:00	18	28.0	29.3	1.3
3C	2/26/2023 2:00	18	27.0	29.3	2.3
3C	2/26/2023 3:00	18	28.0	29.3	1.3
3C	2/26/2023 4:00	18	28.0	29.3	1.3
3C	2/26/2023 5:00	18	28.0	29.3	1.3
3C	2/26/2023 6:00	18	28.0	29.3	1.3
3C	2/26/2023 7:00	18	28.0	29.3	1.3
3C	2/26/2023 8:00	18	24.0	18.3	-5.7
3C	2/26/2023 9:00	18	24.0	18.3	-5.7

3C	2/26/2023 10:00	18	24.0	18.3	-5.7
3C	2/26/2023 11:00	18	24.0	19.3	-4.7
3C	2/26/2023 12:00	18	24.0	26.4	2.4
3C	2/26/2023 13:00	18	24.0	26.4	2.4
3C	2/26/2023 14:00	18	24.0	26.4	2.4
3C	2/26/2023 15:00	18	24.0	24.4	0.4
3C	2/26/2023 16:00	18	24.0	30.5	6.5
3C	2/26/2023 17:00	18	24.0	30.5	6.5
3C	2/26/2023 18:00	18	24.0	30.5	6.5
3C	2/26/2023 19:00	18	24.0	30.5	6.5
3C	2/26/2023 20:00	19	27.0	25.8	-1.2
3C	2/26/2023 21:00	19	27.0	25.8	-1.2
3C	2/26/2023 22:00	19	27.0	25.8	-1.2
3C	2/26/2023 23:00	19	27.0	24.6	-2.4
3C	2/27/2023 0:00	19	30.0	18.7	-11.3
3C	2/27/2023 1:00	19	30.0	18.7	-11.3
3C	2/27/2023 2:00	19	30.0	18.7	-11.3
3C	2/27/2023 3:00	19	30.0	18.7	-11.3
3C	2/27/2023 4:00	19	23.0	18.7	-4.3
3C	2/27/2023 5:00	19	23.0	18.7	-4.3
3C	2/27/2023 6:00	19	23.0	18.7	-4.3
3C	2/27/2023 7:00	19	30.0	18.7	-11.3
3C	2/27/2023 8:00	19	30.0	20.3	-9.7
3C	2/27/2023 9:00	19	30.0	20.3	-9.7
3C	2/27/2023 10:00	19	30.0	20.3	-9.7
3C	2/27/2023 11:00	19	30.0	20.3	-9.7
3C	2/27/2023 12:00	19	30.0	22.4	-7.6
3C	2/27/2023 13:00	19	30.0	22.4	-7.6
3C	2/27/2023 14:00	19	30.0	22.4	-7.6
3C	2/27/2023 15:00	21	30.0	22.4	-7.6
3C	2/27/2023 16:00	21	30.0	28.1	-1.9
3C	2/27/2023 17:00	21	30.0	28.1	-1.9
3C	2/27/2023 18:00	21	30.0	28.1	-1.9
3C	2/27/2023 19:00	22	30.0	28.1	-1.9
3C	2/27/2023 20:00	22	30.0	18.7	-11.3
3C	2/27/2023 21:00	22	30.0	18.7	-11.3
3C	2/27/2023 22:00	22	30.0	18.7	-11.3
3C	2/27/2023 23:00	22	30.0	18.7	-11.3
3C	2/28/2023 0:00	22	30.0	29.3	-0.7
3C	2/28/2023 1:00	22	30.0	29.3	-0.7
3C	2/28/2023 2:00	22	25.0	29.3	4.3
3C	2/28/2023 3:00	22	30.0	29.3	-0.7
3C	2/28/2023 4:00	22	30.0	29.3	-0.7
3C	2/28/2023 5:00	22	30.0	29.3	-0.7
3C	2/28/2023 6:00	22	29.0	29.3	0.3
3C	2/28/2023 7:00	22	29.0	30.0	1.0
3C	2/28/2023 8:00	22	29.0	22.9	-6.1

3C	2/28/2023 9:00	22	29.0	22.9	-6.1
3C	2/28/2023 10:00	22	29.0	22.9	-6.1
3C	2/28/2023 11:00	22	29.0	22.9	-6.1
3C	2/28/2023 12:00	22	29.0	20.3	-8.7
3C	2/28/2023 13:00	22	29.0	20.3	-8.7
3C	2/28/2023 14:00	22	29.0	20.3	-8.7
3C	2/28/2023 15:00	21	25.0	20.3	-4.7
3C	2/28/2023 16:00	21	25.0	23.4	-1.6
3C	2/28/2023 17:00	21	25.0	23.4	-1.6
3C	2/28/2023 18:00	21	25.0	23.4	-1.6
3C	2/28/2023 19:00	21	25.0	23.4	-1.6
3C	2/28/2023 20:00	21	25.0	20.5	-4.5
3C	2/28/2023 21:00	21	25.0	20.5	-4.5
3C	2/28/2023 22:00	21	25.0	20.5	-4.5
3C	2/28/2023 23:00	21	25.0	20.5	-4.5
3C	3/1/2023 0:00	21	25.0	23.3	-1.7
3C	3/1/2023 1:00	21	25.0	23.3	-1.7
3C	3/1/2023 2:00	21	25.0	23.3	-1.7
3C	3/1/2023 3:00	21	25.0	23.3	-1.7
3C	3/1/2023 4:00	21	25.0	23.3	-1.7
3C	3/1/2023 5:00	21	25.0	23.3	-1.7
3C	3/1/2023 6:00	21	25.0	23.3	-1.7
3C	3/1/2023 7:00	21	25.0	23.3	-1.7
3C	3/1/2023 8:00	21	25.0	30.5	5.5
3C	3/1/2023 9:00	21	25.0	30.5	5.5
3C	3/1/2023 10:00	21	25.0	30.5	5.5
3C	3/1/2023 11:00	21	25.0	30.5	5.5
3C	3/1/2023 12:00	21	25.0	34.0	9.0
3C	3/1/2023 13:00	24	29.0	34.0	5.0
3C	3/1/2023 14:00	24	29.0	34.0	5.0
3C	3/1/2023 15:00	24	29.0	34.0	5.0
3C	3/1/2023 16:00	24	29.0	34.0	5.0
3C	3/1/2023 17:00	24	29.0	34.0	5.0
3C	3/1/2023 18:00	24	29.0	34.0	5.0
3C	3/1/2023 19:00	24	29.0	34.0	5.0
3C	3/1/2023 20:00	24	29.0	23.4	-5.6
3C	3/1/2023 21:00	24	29.0	23.4	-5.6
3C	3/1/2023 22:00	24	29.0	23.4	-5.6
3C	3/1/2023 23:00	24	29.0	23.4	-5.6
3C	3/2/2023 0:00	24	30.0	21.7	-8.3
3C	3/2/2023 1:00	24	30.0	23.3	-6.7
3C	3/2/2023 2:00	24	25.0	23.3	-1.7
3C	3/2/2023 3:00	26	25.0	23.3	-1.7
3C	3/2/2023 4:00	26	25.0	23.3	-1.7
3C	3/2/2023 5:00	26	25.0	23.3	-1.7
3C	3/2/2023 6:00	26	25.0	23.3	-1.7
3C	3/2/2023 7:00	26	25.0	23.3	-1.7

3C	3/2/2023 8:00	29	25.0	20.3	-4.7
3C	3/2/2023 9:00	29	25.0	20.3	-4.7
3C	3/2/2023 10:00	29	25.0	20.3	-4.7
3C	3/2/2023 11:00	29	25.0	20.3	-4.7
3C	3/2/2023 12:00	29	25.0	22.4	-2.6
3C	3/2/2023 13:00	29	25.0	22.4	-2.6
3C	3/2/2023 14:00	29	25.0	22.4	-2.6
3C	3/2/2023 15:00	29	25.0	22.4	-2.6
3C	3/2/2023 16:00	29	25.0	25.8	0.8
3C	3/2/2023 17:00	29	25.0	25.8	0.8
3C	3/2/2023 18:00	29	25.0	25.8	0.8
3C	3/2/2023 19:00	29	25.0	25.8	0.8
3C	3/2/2023 20:00	29	25.0	25.8	0.8
3C	3/2/2023 21:00	27	25.0	18.7	-6.3
3C	3/2/2023 22:00	27	25.0	18.7	-6.3
3C	3/2/2023 23:00	27	25.0	18.7	-6.3
3C	3/3/2023 0:00	27	28.0	10.7	-17.3
3C	3/3/2023 1:00	27	28.0	13.3	-14.7
3C	3/3/2023 2:00	27	30.0	13.3	-16.7
3C	3/3/2023 3:00	27	28.0	13.3	-14.7
3C	3/3/2023 4:00	27	28.0	10.7	-17.3
3C	3/3/2023 5:00	27	28.0	10.7	-17.3
3C	3/3/2023 6:00	27	28.0	10.7	-17.3
3C	3/3/2023 7:00	27	28.0	8.0	-20.0
3C	3/3/2023 8:00	27	28.0	14.2	-13.8
3C	3/3/2023 9:00	27	28.0	14.2	-13.8
3C	3/3/2023 10:00	27	28.0	14.2	-13.8
3C	3/3/2023 11:00	27	28.0	14.2	-13.8
3C	3/3/2023 12:00	27	28.0	16.3	-11.7
3C	3/3/2023 13:00	27	28.0	16.3	-11.7
3C	3/3/2023 14:00	27	28.0	16.3	-11.7
3C	3/3/2023 15:00	25	30.0	20.3	-9.7
3C	3/3/2023 16:00	25	30.0	18.7	-11.3
3C	3/3/2023 17:00	25	30.0	18.7	-11.3
3C	3/3/2023 18:00	25	30.0	18.7	-11.3
3C	3/3/2023 19:00	25	30.0	18.7	-11.3
3C	3/3/2023 20:00	25	30.0	21.1	-8.9
3C	3/3/2023 21:00	25	30.0	21.1	-8.9
3C	3/3/2023 22:00	25	30.0	21.1	-8.9
3C	3/3/2023 23:00	25	30.0	21.1	-8.9
3C	3/4/2023 0:00	25	30.0	18.7	-11.3
3C	3/4/2023 1:00	25	30.0	18.7	-11.3
3C	3/4/2023 2:00	25	30.0	18.7	-11.3
3C	3/4/2023 3:00	25	30.0	18.7	-11.3
3C	3/4/2023 4:00	25	30.0	18.7	-11.3
3C	3/4/2023 5:00	25	30.0	18.7	-11.3
3C	3/4/2023 6:00	25	30.0	18.7	-11.3

3C	3/4/2023 7:00	25	30.0	18.7	-11.3
3C	3/4/2023 8:00	29	30.0	22.4	-7.6
3C	3/4/2023 9:00	29	30.0	22.4	-7.6
3C	3/4/2023 10:00	29	30.0	22.4	-7.6
3C	3/4/2023 11:00	29	30.0	22.4	-7.6
3C	3/4/2023 12:00	29	30.0	24.4	-5.6
3C	3/4/2023 13:00	29	30.0	24.4	-5.6
3C	3/4/2023 14:00	29	30.0	24.4	-5.6
3C	3/4/2023 15:00	29	30.0	24.4	-5.6
3C	3/4/2023 16:00	29	30.0	28.1	-1.9
3C	3/4/2023 17:00	29	30.0	28.1	-1.9
3C	3/4/2023 18:00	29	30.0	28.1	-1.9
3C	3/4/2023 19:00	29	30.0	28.1	-1.9
3C	3/4/2023 20:00	29	30.0	20.5	-9.5
3C	3/4/2023 21:00	29	30.0	20.5	-9.5
3C	3/4/2023 22:00	29	30.0	20.5	-9.5
3C	3/4/2023 23:00	29	30.0	20.5	-9.5
3C	3/5/2023 0:00	29	30.0	23.3	-6.7
3C	3/5/2023 1:00	29	30.0	23.3	-6.7
3C	3/5/2023 2:00	29	30.0	23.3	-6.7
3C	3/5/2023 3:00	29	30.0	23.3	-6.7
3C	3/5/2023 4:00	29	30.0	23.3	-6.7
3C	3/5/2023 5:00	29	30.0	23.3	-6.7
3C	3/5/2023 6:00	29	30.0	23.3	-6.7
3C	3/5/2023 7:00	25	30.0	26.7	-3.4
3C	3/5/2023 8:00	25	30.0	22.4	-7.6
3C	3/5/2023 9:00	25	30.0	22.4	-7.6
3C	3/5/2023 10:00	25	30.0	22.4	-7.6
3C	3/5/2023 11:00	25	30.0	22.4	-7.6
3C	3/5/2023 12:00	25	30.0	26.4	-3.6
3C	3/5/2023 13:00	25	30.0	26.4	-3.6
3C	3/5/2023 14:00	25	30.0	26.4	-3.6
3C	3/5/2023 15:00	22	30.0	26.4	-3.6
3C	3/5/2023 16:00	22	30.0	30.5	0.5
3C	3/5/2023 17:00	22	30.0	30.5	0.5
3C	3/5/2023 18:00	22	30.0	30.5	0.5
3C	3/5/2023 19:00	23	30.0	30.5	0.5
3C	3/5/2023 20:00	23	30.0	18.7	-11.3
3C	3/5/2023 21:00	23	30.0	18.7	-11.3
3C	3/5/2023 22:00	23	30.0	18.7	-11.3
3C	3/5/2023 23:00	23	30.0	18.7	-11.3
3C	3/6/2023 0:00	23	30.0	20.0	-10.0
3C	3/6/2023 1:00	23	30.0	20.0	-10.0
3C	3/6/2023 2:00	23	30.0	20.0	-10.0
3C	3/6/2023 3:00	23	30.0	20.0	-10.0
3C	3/6/2023 4:00	23	30.0	20.0	-10.0
3C	3/6/2023 5:00	23	30.0	20.0	-10.0

3C	3/6/2023 6:00	23	30.0	20.0	-10.0
3C	3/6/2023 7:00	25	30.0	20.0	-10.0
3C	3/6/2023 8:00	25	30.0	20.3	-9.7
3C	3/6/2023 9:00	25	30.0	20.3	-9.7
3C	3/6/2023 10:00	25	30.0	20.3	-9.7
3C	3/6/2023 11:00	25	30.0	20.3	-9.7
3C	3/6/2023 12:00	28	30.0	20.3	-9.7
3C	3/6/2023 13:00	28	30.0	20.3	-9.7
3C	3/6/2023 14:00	28	30.0	20.3	-9.7
3C	3/6/2023 15:00	28	30.0	20.3	-9.7
3C	3/6/2023 16:00	28	30.0	23.4	-6.6
3C	3/6/2023 17:00	28	30.0	23.4	-6.6
3C	3/6/2023 18:00	29	31.0	23.4	-7.6
3C	3/6/2023 19:00	29	31.0	23.4	-7.6
3C	3/6/2023 20:00	29	31.0	21.1	-9.9
3C	3/6/2023 21:00	29	31.0	21.1	-9.9
3C	3/6/2023 22:00	29	31.0	21.1	-9.9
3C	3/6/2023 23:00	29	31.0	21.1	-9.9
3C	3/7/2023 0:00	30	30.0	24.0	-6.0
3C	3/7/2023 1:00	30	30.0	24.0	-6.0
3C	3/7/2023 2:00	30	30.0	24.0	-6.0
3C	3/7/2023 3:00	30	30.0	24.0	-6.0
3C	3/7/2023 4:00	30	30.0	24.0	-6.0
3C	3/7/2023 5:00	30	30.0	24.0	-6.0
3C	3/7/2023 6:00	30	30.0	24.0	-6.0
3C	3/7/2023 7:00	30	34.0	24.0	-10.0
3C	3/7/2023 8:00	30	34.0	20.3	-13.7
3C	3/7/2023 9:00	30	34.0	20.3	-13.7
3C	3/7/2023 10:00	30	34.0	20.3	-13.7
3C	3/7/2023 11:00	30	34.0	20.3	-13.7
3C	3/7/2023 12:00	30	34.0	22.4	-11.6
3C	3/7/2023 13:00	30	34.0	22.4	-11.6
3C	3/7/2023 14:00	30	34.0	22.4	-11.6
3C	3/7/2023 15:00	30	34.0	22.4	-11.6
3C	3/7/2023 16:00	30	34.0	25.8	-8.2
3C	3/7/2023 17:00	30	34.0	25.8	-8.2
3C	3/7/2023 18:00	30	34.0	25.8	-8.2
3C	3/7/2023 19:00	30	34.0	25.8	-8.2
3C	3/7/2023 20:00	30	34.0	23.4	-10.6
3C	3/7/2023 21:00	30	34.0	23.4	-10.6
3C	3/7/2023 22:00	27	28.0	21.1	-6.9
3C	3/7/2023 23:00	27	28.0	21.1	-6.9
3C	3/8/2023 0:00	27	28.0	18.7	-9.3
3C	3/8/2023 1:00	29	30.0	18.7	-11.3
3C	3/8/2023 2:00	29	30.0	18.7	-11.3
3C	3/8/2023 3:00	30	30.0	18.7	-11.3
3C	3/8/2023 4:00	30	30.0	18.7	-11.3

3C	3/8/2023 5:00	30	30.0	18.7	-11.3
3C	3/8/2023 6:00	30	30.0	18.7	-11.3
3C	3/8/2023 7:00	30	30.0	18.7	-11.3
3C	3/8/2023 8:00	30	30.0	18.3	-11.7
3C	3/8/2023 9:00	30	30.0	18.3	-11.7
3C	3/8/2023 10:00	30	30.0	18.3	-11.7
3C	3/8/2023 11:00	30	30.0	18.3	-11.7
3C	3/8/2023 12:00	30	30.0	20.3	-9.7
3C	3/8/2023 13:00	30	30.0	20.3	-9.7
3C	3/8/2023 14:00	30	30.0	20.3	-9.7
3C	3/8/2023 15:00	30	30.0	20.3	-9.7
3C	3/8/2023 16:00	30	30.0	18.7	-11.3
3C	3/8/2023 17:00	30	30.0	18.7	-11.3
3C	3/8/2023 18:00	30	30.0	18.7	-11.3
3C	3/8/2023 19:00	29	29.0	18.7	-10.3
3C	3/8/2023 20:00	29	29.0	18.7	-10.3
3C	3/8/2023 21:00	29	29.0	18.7	-10.3
3C	3/8/2023 22:00	29	29.0	18.7	-10.3
3C	3/8/2023 23:00	29	29.0	18.7	-10.3
3C	3/9/2023 0:00	29	29.0	18.7	-10.3
3C	3/9/2023 1:00	29	29.0	18.7	-10.3
3C	3/9/2023 2:00	29	29.0	18.7	-10.3
3C	3/9/2023 3:00	29	29.0	18.7	-10.3
3C	3/9/2023 4:00	29	29.0	18.7	-10.3
3C	3/9/2023 5:00	29	29.0	18.7	-10.3
3C	3/9/2023 6:00	29	29.0	18.7	-10.3
3C	3/9/2023 7:00	28	34.0	18.7	-15.3
3C	3/9/2023 8:00	28	34.0	17.3	-16.7
3C	3/9/2023 9:00	28	34.0	18.3	-15.7
3C	3/9/2023 10:00	28	34.0	18.3	-15.7
3C	3/9/2023 11:00	28	34.0	18.3	-15.7
3C	3/9/2023 12:00	28	34.0	20.3	-13.7
3C	3/9/2023 13:00	28	34.0	20.3	-13.7
3C	3/9/2023 14:00	28	34.0	20.3	-13.7
3C	3/9/2023 15:00	24	33.0	20.3	-12.7
3C	3/9/2023 16:00	24	33.0	20.5	-12.5
3C	3/9/2023 17:00	24	33.0	20.5	-12.5
3C	3/9/2023 18:00	24	33.0	20.5	-12.5
3C	3/9/2023 19:00	28	33.0	20.5	-12.5
3C	3/9/2023 20:00	28	33.0	23.4	-9.6
3C	3/9/2023 21:00	29	33.0	18.7	-14.3
3C	3/9/2023 22:00	31	31.0	18.7	-12.3
3C	3/9/2023 23:00	31	31.0	21.1	-9.9
3C	3/10/2023 0:00	31	31.0	18.7	-12.3
3C	3/10/2023 1:00	31	31.0	18.7	-12.3
3C	3/10/2023 2:00	31	31.0	18.7	-12.3
3C	3/10/2023 3:00	31	31.0	18.7	-12.3

3C	3/10/2023 4:00	31	31.0	18.7	-12.3
3C	3/10/2023 5:00	31	31.0	18.7	-12.3
3C	3/10/2023 6:00	31	31.0	18.7	-12.3
3C	3/10/2023 7:00	31	31.0	16.0	-15.0
3C	3/10/2023 8:00	31	31.0	17.8	-13.2
3C	3/10/2023 9:00	31	31.0	17.8	-13.2
3C	3/10/2023 10:00	31	31.0	17.8	-13.2
3C	3/10/2023 11:00	31	31.0	17.8	-13.2
3C	3/10/2023 12:00	31	31.0	17.8	-13.2
3C	3/10/2023 13:00	31	31.0	17.8	-13.2
3C	3/10/2023 14:00	31	31.0	17.8	-13.2
3C	3/10/2023 15:00	25	31.0	17.8	-13.2
3C	3/10/2023 16:00	25	31.0	14.1	-17.0
3C	3/10/2023 17:00	25	31.0	14.1	-17.0
3C	3/10/2023 18:00	25	31.0	14.1	-17.0
3C	3/10/2023 19:00	25	31.0	14.1	-17.0
3C	3/10/2023 20:00	25	31.0	16.4	-14.6
3C	3/10/2023 21:00	25	31.0	16.4	-14.6
3C	3/10/2023 22:00	25	31.0	16.4	-14.6
3C	3/10/2023 23:00	25	31.0	16.4	-14.6
3C	3/11/2023 0:00	24	27.0	16.0	-11.0
3C	3/11/2023 1:00	24	27.0	16.0	-11.0
3C	3/11/2023 2:00	24	27.0	16.0	-11.0
3C	3/11/2023 3:00	24	27.0	16.0	-11.0
3C	3/11/2023 4:00	24	27.0	16.0	-11.0
3C	3/11/2023 5:00	24	27.0	16.0	-11.0
3C	3/11/2023 6:00	24	26.0	16.0	-10.0
3C	3/11/2023 7:00	24	26.0	16.0	-10.0
3C	3/11/2023 8:00	24	26.0	16.3	-9.7
3C	3/11/2023 9:00	24	26.0	16.3	-9.7
3C	3/11/2023 10:00	24	26.0	16.3	-9.7
3C	3/11/2023 11:00	24	26.0	16.3	-9.7
3C	3/11/2023 12:00	24	26.0	18.3	-7.7
3C	3/11/2023 13:00	24	26.0	18.3	-7.7
3C	3/11/2023 14:00	16	24.0	20.3	-3.7
3C	3/11/2023 15:00	16	24.0	20.3	-3.7
3C	3/11/2023 16:00	16	24.0	25.8	1.8
3C	3/11/2023 17:00	16	24.0	25.8	1.8
3C	3/11/2023 18:00	16	24.0	25.8	1.8
3C	3/11/2023 19:00	16	24.0	25.8	1.8
3C	3/11/2023 20:00	16	24.0	18.7	-5.3
3C	3/11/2023 21:00	17	23.0	18.7	-4.3
3C	3/11/2023 22:00	17	23.0	18.7	-4.3
3C	3/11/2023 23:00	17	23.0	18.7	-4.3
3C	3/12/2023 0:00	17	23.0	16.0	-7.0
3C	3/12/2023 1:00	17	18.0	16.0	-2.0
3C	3/12/2023 2:00	17	18.0	16.0	-2.0

3C	3/12/2023 3:00	17	18.0	16.0	-2.0
3C	3/12/2023 4:00	17	18.0	16.0	-2.0
3C	3/12/2023 5:00	17	18.0	16.0	-2.0
3C	3/12/2023 6:00	17	18.0	16.0	-2.0
3C	3/12/2023 7:00	17	18.0	16.0	-2.0
3C	3/12/2023 8:00	17	18.0	16.3	-1.7
3C	3/12/2023 9:00	17	18.0	16.3	-1.7
3C	3/12/2023 10:00	17	18.0	16.3	-1.7
3C	3/12/2023 11:00	17	18.0	16.3	-1.7
3C	3/12/2023 12:00	17	18.0	18.3	0.3
3C	3/12/2023 13:00	17	18.0	18.3	0.3
3C	3/12/2023 14:00	17	18.0	18.3	0.3
3C	3/12/2023 15:00	17	18.0	18.3	0.3
3C	3/12/2023 16:00	17	18.0	23.4	5.4
3C	3/12/2023 17:00	17	18.0	23.4	5.4
3C	3/12/2023 18:00	17	18.0	23.4	5.4
3C	3/12/2023 19:00	17	18.0	23.4	5.4
3C	3/12/2023 20:00	19	26.0	17.6	-8.4
3C	3/12/2023 21:00	19	26.0	17.6	-8.4
3C	3/12/2023 22:00	19	26.0	17.6	-8.4
3C	3/12/2023 23:00	19	26.0	17.6	-8.4
3C	3/13/2023 0:00	19	26.0	20.0	-6.0
3C	3/13/2023 1:00	19	26.0	20.0	-6.0
3C	3/13/2023 2:00	19	26.0	20.0	-6.0
3C	3/13/2023 3:00	19	26.0	20.0	-6.0
3C	3/13/2023 4:00	19	26.0	20.0	-6.0
3C	3/13/2023 5:00	19	26.0	20.0	-6.0
3C	3/13/2023 6:00	20	34.0	21.3	-12.7
3C	3/13/2023 7:00	20	34.0	21.3	-12.7
3C	3/13/2023 8:00	20	30.0	20.3	-9.7
3C	3/13/2023 9:00	20	30.0	20.3	-9.7
3C	3/13/2023 10:00	20	30.0	20.3	-9.7
3C	3/13/2023 11:00	20	30.0	20.3	-9.7
3C	3/13/2023 12:00	20	30.0	20.3	-9.7
3C	3/13/2023 13:00	20	30.0	20.3	-9.7
3C	3/13/2023 14:00	20	30.0	22.9	-7.1
3C	3/13/2023 15:00	20	30.0	22.9	-7.1
3C	3/13/2023 16:00	20	30.0	26.4	-3.7
3C	3/13/2023 17:00	20	30.0	26.4	-3.7
3C	3/13/2023 18:00	20	30.0	26.4	-3.7
3C	3/13/2023 19:00	20	30.0	26.4	-3.7
3C	3/13/2023 20:00	19	22.0	14.6	-7.4
3C	3/13/2023 21:00	19	22.0	14.6	-7.4
3C	3/13/2023 22:00	19	22.0	14.6	-7.4
3C	3/13/2023 23:00	19	22.0	14.6	-7.4
3C	3/14/2023 0:00	19	22.0	23.3	1.3
3C	3/14/2023 1:00	19	22.0	23.3	1.3

3C	3/14/2023 2:00	19	30.0	23.3	-6.7
3C	3/14/2023 3:00	19	22.0	23.3	1.3
3C	3/14/2023 4:00	19	22.0	23.3	1.3
3C	3/14/2023 5:00	19	22.0	23.3	1.3
3C	3/14/2023 6:00	19	22.0	23.3	1.3
3C	3/14/2023 7:00	19	22.0	23.3	1.3
3C	3/14/2023 8:00	19	22.0	17.8	-4.2
3C	3/14/2023 9:00	19	22.0	17.8	-4.2
3C	3/14/2023 10:00	19	22.0	17.8	-4.2
3C	3/14/2023 11:00	20	30.0	17.8	-12.2
3C	3/14/2023 12:00	20	30.0	17.8	-12.2
3C	3/14/2023 13:00	20	30.0	17.8	-12.2
3C	3/14/2023 14:00	20	30.0	17.8	-12.2
3C	3/14/2023 15:00	20	30.0	17.8	-12.2
3C	3/14/2023 16:00	20	30.0	20.5	-9.5
3C	3/14/2023 17:00	20	30.0	20.5	-9.5
3C	3/14/2023 18:00	20	30.0	20.5	-9.5
3C	3/14/2023 19:00	20	30.0	20.5	-9.5
3C	3/14/2023 20:00	20	30.0	26.4	-3.7
3C	3/14/2023 21:00	20	30.0	26.4	-3.7
3C	3/14/2023 22:00	20	30.0	26.4	-3.7
3C	3/14/2023 23:00	20	30.0	26.4	-3.7
3C	3/15/2023 0:00	20	33.0	26.7	-6.4
3C	3/15/2023 1:00	20	33.0	26.7	-6.4
3C	3/15/2023 2:00	20	31.0	26.7	-4.4
3C	3/15/2023 3:00	20	30.0	26.7	-3.4
3C	3/15/2023 4:00	20	30.0	26.7	-3.4
3C	3/15/2023 5:00	20	30.0	26.7	-3.4
3C	3/15/2023 6:00	20	30.0	26.7	-3.4
3C	3/15/2023 7:00	24	33.0	26.7	-6.4
3C	3/15/2023 8:00	24	33.0	20.3	-12.7
3C	3/15/2023 9:00	24	33.0	20.3	-12.7
3C	3/15/2023 10:00	24	33.0	20.3	-12.7
3C	3/15/2023 11:00	24	33.0	20.3	-12.7
3C	3/15/2023 12:00	24	33.0	25.4	-7.6
3C	3/15/2023 13:00	24	33.0	25.4	-7.6
3C	3/15/2023 14:00	24	33.0	25.4	-7.6
3C	3/15/2023 15:00	24	34.0	24.4	-9.6
3C	3/15/2023 16:00	24	34.0	23.4	-10.6
3C	3/15/2023 17:00	24	34.0	23.4	-10.6
3C	3/15/2023 18:00	24	34.0	23.4	-10.6
3C	3/15/2023 19:00	24	31.0	23.4	-7.6
3C	3/15/2023 20:00	24	31.0	28.1	-2.9
3C	3/15/2023 21:00	24	31.0	28.1	-2.9
3C	3/15/2023 22:00	24	31.0	28.1	-2.9
3C	3/15/2023 23:00	24	31.0	28.1	-2.9
3C	3/16/2023 0:00	24	33.0	32.0	-1.0

3C	3/16/2023 1:00	24	33.0	32.0	-1.0
3C	3/16/2023 2:00	24	34.0	32.0	-2.0
3C	3/16/2023 3:00	24	34.0	32.0	-2.0
3C	3/16/2023 4:00	24	34.0	32.0	-2.0
3C	3/16/2023 5:00	24	34.0	32.0	-2.0
3C	3/16/2023 6:00	24	34.0	32.0	-2.0
3C	3/16/2023 7:00	27	34.0	24.0	-10.0
3C	3/16/2023 8:00	27	34.0	18.3	-15.7
3C	3/16/2023 9:00	27	34.0	18.3	-15.7
3C	3/16/2023 10:00	27	34.0	18.3	-15.7
3C	3/16/2023 11:00	27	34.0	18.3	-15.7
3C	3/16/2023 12:00	27	34.0	20.3	-13.7
3C	3/16/2023 13:00	27	34.0	20.3	-13.7
3C	3/16/2023 14:00	27	34.0	20.3	-13.7
3C	3/16/2023 15:00	28	34.0	22.9	-11.1
3C	3/16/2023 16:00	28	34.0	23.4	-10.6
3C	3/16/2023 17:00	28	34.0	23.4	-10.6
3C	3/16/2023 18:00	28	34.0	23.4	-10.6
3C	3/16/2023 19:00	32	34.0	23.4	-10.6
3C	3/16/2023 20:00	32	34.0	23.4	-10.6
3C	3/16/2023 21:00	32	34.0	23.4	-10.6
3C	3/16/2023 22:00	32	34.0	23.4	-10.6
3C	3/16/2023 23:00	32	34.0	23.4	-10.6
3C	3/17/2023 0:00	32	34.0	25.3	-8.7
3C	3/17/2023 1:00	32	34.0	26.7	-7.4
3C	3/17/2023 2:00	32	29.0	26.7	-2.4
3C	3/17/2023 3:00	32	34.0	26.7	-7.4
3C	3/17/2023 4:00	32	34.0	26.7	-7.4
3C	3/17/2023 5:00	32	34.0	26.7	-7.4
3C	3/17/2023 6:00	32	34.0	26.7	-7.4
3C	3/17/2023 7:00	32	34.0	26.7	-7.4
3C	3/17/2023 8:00	32	34.0	20.3	-13.7
3C	3/17/2023 9:00	31	34.0	20.3	-13.7
3C	3/17/2023 10:00	31	34.0	20.3	-13.7
3C	3/17/2023 11:00	31	34.0	20.3	-13.7
3C	3/17/2023 12:00	31	34.0	25.4	-8.6
3C	3/17/2023 13:00	31	34.0	25.4	-8.6
3C	3/17/2023 14:00	26	29.0	25.4	-3.6
3C	3/17/2023 15:00	26	29.0	25.4	-3.6
3C	3/17/2023 16:00	26	29.0	25.8	-3.2
3C	3/17/2023 17:00	26	29.0	25.8	-3.2
3C	3/17/2023 18:00	26	29.0	25.8	-3.2
3C	3/17/2023 19:00	26	29.0	25.8	-3.2
3C	3/17/2023 20:00	26	29.0	26.4	-2.7
3C	3/17/2023 21:00	26	29.0	26.4	-2.7
3C	3/17/2023 22:00	26	29.0	26.4	-2.7
3C	3/17/2023 23:00	26	29.0	26.4	-2.7

3C	3/18/2023 0:00	26	29.0	23.3	-5.7
3C	3/18/2023 1:00	26	29.0	23.3	-5.7
3C	3/18/2023 2:00	26	29.0	23.3	-5.7
3C	3/18/2023 3:00	26	29.0	23.3	-5.7
3C	3/18/2023 4:00	26	29.0	23.3	-5.7
3C	3/18/2023 5:00	26	29.0	23.3	-5.7
3C	3/18/2023 6:00	26	29.0	23.3	-5.7
3C	3/18/2023 7:00	26	29.0	23.3	-5.7
3C	3/18/2023 8:00	26	29.0	20.3	-8.7
3C	3/18/2023 9:00	26	29.0	20.3	-8.7
3C	3/18/2023 10:00	26	29.0	20.3	-8.7
3C	3/18/2023 11:00	26	29.0	20.3	-8.7
3C	3/18/2023 12:00	26	29.0	22.4	-6.6
3C	3/18/2023 13:00	26	29.0	22.4	-6.6
3C	3/18/2023 14:00	26	29.0	22.4	-6.6
3C	3/18/2023 15:00	22	30.0	22.4	-7.6
3C	3/18/2023 16:00	22	30.0	23.4	-6.6
3C	3/18/2023 17:00	22	30.0	23.4	-6.6
3C	3/18/2023 18:00	22	30.0	23.4	-6.6
3C	3/18/2023 19:00	22	30.0	23.4	-6.6
3C	3/18/2023 20:00	22	30.0	16.4	-13.6
3C	3/18/2023 21:00	22	30.0	16.4	-13.6
3C	3/18/2023 22:00	22	30.0	16.4	-13.6
3C	3/18/2023 23:00	22	30.0	16.4	-13.6
3C	3/19/2023 0:00	22	30.0	23.3	-6.7
3C	3/19/2023 1:00	22	30.0	23.3	-6.7
3C	3/19/2023 2:00	22	30.0	23.3	-6.7
3C	3/19/2023 3:00	22	30.0	23.3	-6.7
3C	3/19/2023 4:00	22	30.0	23.3	-6.7
3C	3/19/2023 5:00	22	30.0	23.3	-6.7
3C	3/19/2023 6:00	22	30.0	23.3	-6.7
3C	3/19/2023 7:00	22	30.0	23.3	-6.7
3C	3/19/2023 8:00	21	30.0	15.3	-14.7
3C	3/19/2023 9:00	21	30.0	15.3	-14.7
3C	3/19/2023 10:00	21	30.0	15.3	-14.7
3C	3/19/2023 11:00	21	30.0	15.3	-14.7
3C	3/19/2023 12:00	21	30.0	20.3	-9.7
3C	3/19/2023 13:00	21	30.0	20.3	-9.7
3C	3/19/2023 14:00	21	30.0	20.3	-9.7
3C	3/19/2023 15:00	21	30.0	20.3	-9.7
3C	3/19/2023 16:00	21	30.0	21.1	-8.9
3C	3/19/2023 17:00	21	30.0	21.1	-8.9
3C	3/19/2023 18:00	21	30.0	21.1	-8.9
3C	3/19/2023 19:00	21	30.0	21.1	-8.9
3C	3/19/2023 20:00	21	30.0	14.1	-16.0
3C	3/19/2023 21:00	21	30.0	14.1	-16.0
3C	3/19/2023 22:00	21	30.0	14.1	-16.0

3C	3/19/2023 23:00	21	30.0	14.1	-16.0
3C	3/20/2023 0:00	21	30.0	18.3	-11.7
3C	3/20/2023 1:00	21	30.0	20.0	-10.0
3C	3/20/2023 2:00	21	30.0	20.0	-10.0
3C	3/20/2023 3:00	21	30.0	20.0	-10.0
3C	3/20/2023 4:00	21	30.0	20.0	-10.0
3C	3/20/2023 5:00	21	30.0	20.0	-10.0
3C	3/20/2023 6:00	21	30.0	20.0	-10.0
3C	3/20/2023 7:00	21	30.0	20.0	-10.0
3C	3/20/2023 8:00	21	30.0	20.3	-9.7
3C	3/20/2023 9:00	21	30.0	20.3	-9.7
3C	3/20/2023 10:00	21	30.0	20.3	-9.7
3C	3/20/2023 11:00	21	30.0	20.3	-9.7
3C	3/20/2023 12:00	21	30.0	25.4	-4.6
3C	3/20/2023 13:00	21	30.0	25.4	-4.6
3C	3/20/2023 14:00	21	30.0	25.4	-4.6
3C	3/20/2023 15:00	21	30.0	25.4	-4.6
3C	3/20/2023 16:00	21	30.0	29.3	-0.7
3C	3/20/2023 17:00	21	30.0	29.3	-0.7
3C	3/20/2023 18:00	21	30.0	29.3	-0.7
3C	3/20/2023 19:00	16	18.0	30.7	12.7
3C	3/20/2023 20:00	16	18.0	20.5	2.5
3C	3/20/2023 21:00	16	18.0	20.5	2.5
3C	3/20/2023 22:00	16	18.0	20.5	2.5
3C	3/20/2023 23:00	16	18.0	20.5	2.5
3C	3/21/2023 0:00	16	18.0	23.3	5.3
3C	3/21/2023 1:00	16	18.0	23.3	5.3
3C	3/21/2023 2:00	16	23.0	23.3	0.3
3C	3/21/2023 3:00	16	23.0	23.3	0.3
3C	3/21/2023 4:00	16	23.0	23.3	0.3
3C	3/21/2023 5:00	16	18.0	23.3	5.3
3C	3/21/2023 6:00	16	18.0	23.3	5.3
3C	3/21/2023 7:00	22	26.0	23.3	-2.7
3C	3/21/2023 8:00	22	26.0	22.9	-3.1
3C	3/21/2023 9:00	22	26.0	22.9	-3.1
3C	3/21/2023 10:00	22	23.0	22.9	-0.1
3C	3/21/2023 11:00	22	23.0	22.9	-0.1
3C	3/21/2023 12:00	22	23.0	20.3	-2.7
3C	3/21/2023 13:00	22	23.0	20.3	-2.7
3C	3/21/2023 14:00	22	23.0	20.3	-2.7
3C	3/21/2023 15:00	21	23.0	19.1	-3.9
3C	3/21/2023 16:00	21	23.0	11.7	-11.3
3C	3/21/2023 17:00	21	23.0	11.7	-11.3
3C	3/21/2023 18:00	21	23.0	11.7	-11.3
3C	3/21/2023 19:00	23	23.0	11.7	-11.3
3C	3/21/2023 20:00	23	23.0	14.6	-8.4
3C	3/21/2023 21:00	23	23.0	14.6	-8.4

3C	3/21/2023 22:00	23	23.0	14.6	-8.4
3C	3/21/2023 23:00	23	23.0	14.6	-8.4
3C	3/22/2023 0:00	23	23.0	16.7	-6.3
3C	3/22/2023 1:00	23	23.0	16.7	-6.3
3C	3/22/2023 2:00	23	23.0	16.7	-6.3
3C	3/22/2023 3:00	23	23.0	16.7	-6.3
3C	3/22/2023 4:00	23	23.0	16.7	-6.3
3C	3/22/2023 5:00	23	23.0	16.7	-6.3
3C	3/22/2023 6:00	23	23.0	16.7	-6.3
3C	3/22/2023 7:00	23	23.0	16.7	-6.3
3C	3/22/2023 8:00	23	23.0	15.3	-7.7
3C	3/22/2023 9:00	23	23.0	15.3	-7.7
3C	3/22/2023 10:00	23	23.0	15.3	-7.7
3C	3/22/2023 11:00	23	23.0	15.3	-7.7
3C	3/22/2023 12:00	23	23.0	17.8	-5.2
3C	3/22/2023 13:00	23	23.0	17.8	-5.2
3C	3/22/2023 14:00	23	23.0	17.8	-5.2
3C	3/22/2023 15:00	23	23.0	17.8	-5.2
3C	3/22/2023 16:00	23	23.0	20.5	-2.5
3C	3/22/2023 17:00	23	23.0	20.5	-2.5
3C	3/22/2023 18:00	23	23.0	20.5	-2.5
3C	3/22/2023 19:00	23	23.0	20.5	-2.5
3C	3/22/2023 20:00	23	23.0	17.6	-5.4
3C	3/22/2023 21:00	23	23.0	17.6	-5.4
3C	3/22/2023 22:00	23	23.0	17.6	-5.4
3C	3/22/2023 23:00	23	23.0	17.6	-5.4
3C	3/23/2023 0:00	23	23.0	23.3	0.3
3C	3/23/2023 1:00	23	23.0	23.3	0.3
3C	3/23/2023 2:00	23	32.0	23.3	-8.7
3C	3/23/2023 3:00	23	23.0	23.3	0.3
3C	3/23/2023 4:00	23	23.0	23.3	0.3
3C	3/23/2023 5:00	29	33.0	23.3	-9.7
3C	3/23/2023 6:00	29	33.0	23.3	-9.7
3C	3/23/2023 7:00	29	32.0	23.3	-8.7
3C	3/23/2023 8:00	29	32.0	20.3	-11.7
3C	3/23/2023 9:00	29	32.0	20.3	-11.7
3C	3/23/2023 10:00	29	32.0	20.3	-11.7
3C	3/23/2023 11:00	29	32.0	20.3	-11.7
3C	3/23/2023 12:00	29	32.0	17.8	-14.2
3C	3/23/2023 13:00	29	32.0	17.8	-14.2
3C	3/23/2023 14:00	29	32.0	17.8	-14.2
3C	3/23/2023 15:00	29	32.0	17.8	-14.2
3C	3/23/2023 16:00	29	32.0	20.5	-11.5
3C	3/23/2023 17:00	29	32.0	20.5	-11.5
3C	3/23/2023 18:00	29	32.0	20.5	-11.5
3C	3/23/2023 19:00	29	32.0	20.5	-11.5
3C	3/23/2023 20:00	28	28.0	17.6	-10.4

3C	3/23/2023 21:00	28	28.0	17.6	-10.4
3C	3/23/2023 22:00	28	28.0	17.6	-10.4
3C	3/23/2023 23:00	28	28.0	17.6	-10.4
3C	3/24/2023 0:00	28	28.0	20.0	-8.0
3C	3/24/2023 1:00	28	28.0	20.0	-8.0
3C	3/24/2023 2:00	28	28.0	20.0	-8.0
3C	3/24/2023 3:00	28	28.0	20.0	-8.0
3C	3/24/2023 4:00	28	28.0	20.0	-8.0
3C	3/24/2023 5:00	28	28.0	20.0	-8.0
3C	3/24/2023 6:00	28	28.0	20.0	-8.0
3C	3/24/2023 7:00	28	28.0	20.0	-8.0
3C	3/24/2023 8:00	28	28.0	12.7	-15.3
3C	3/24/2023 9:00	28	28.0	12.7	-15.3
3C	3/24/2023 10:00	28	28.0	12.7	-15.3
3C	3/24/2023 11:00	28	28.0	12.7	-15.3
3C	3/24/2023 12:00	28	28.0	15.3	-12.7
3C	3/24/2023 13:00	28	28.0	15.3	-12.7
3C	3/24/2023 14:00	28	28.0	15.3	-12.7
3C	3/24/2023 15:00	28	28.0	15.3	-12.7
3C	3/24/2023 16:00	28	28.0	11.7	-16.3
3C	3/24/2023 17:00	28	28.0	11.7	-16.3
3C	3/24/2023 18:00	28	28.0	11.7	-16.3
3C	3/24/2023 19:00	28	28.0	11.7	-16.3
3C	3/24/2023 20:00	28	28.0	23.4	-4.6
3C	3/24/2023 21:00	28	28.0	23.4	-4.6
3C	3/24/2023 22:00	28	28.0	23.4	-4.6
3C	3/24/2023 23:00	28	28.0	23.4	-4.6
3C	3/25/2023 0:00	21	30.0	23.3	-6.7
3C	3/25/2023 1:00	21	30.0	23.3	-6.7
3C	3/25/2023 2:00	21	30.0	23.3	-6.7
3C	3/25/2023 3:00	21	30.0	23.3	-6.7
3C	3/25/2023 4:00	21	30.0	23.3	-6.7
3C	3/25/2023 5:00	21	30.0	23.3	-6.7
3C	3/25/2023 6:00	21	30.0	23.3	-6.7
3C	3/25/2023 7:00	21	30.0	23.3	-6.7
3C	3/25/2023 8:00	21	30.0	20.3	-9.7
3C	3/25/2023 9:00	21	30.0	20.3	-9.7
3C	3/25/2023 10:00	21	30.0	20.3	-9.7
3C	3/25/2023 11:00	21	30.0	20.3	-9.7
3C	3/25/2023 12:00	21	30.0	20.3	-9.7
3C	3/25/2023 13:00	21	30.0	20.3	-9.7
3C	3/25/2023 14:00	21	30.0	20.3	-9.7
3C	3/25/2023 15:00	18	30.0	20.3	-9.7
3C	3/25/2023 16:00	18	30.0	23.4	-6.6
3C	3/25/2023 17:00	18	30.0	23.4	-6.6
3C	3/25/2023 18:00	18	30.0	23.4	-6.6
3C	3/25/2023 19:00	18	30.0	23.4	-6.6

3C	3/25/2023 20:00	18	30.0	20.5	-9.5
3C	3/25/2023 21:00	18	30.0	20.5	-9.5
3C	3/25/2023 22:00	18	30.0	20.5	-9.5
3C	3/25/2023 23:00	18	30.0	20.5	-9.5
3C	3/26/2023 0:00	20	28.0	23.3	-4.7
3C	3/26/2023 1:00	20	28.0	23.3	-4.7
3C	3/26/2023 2:00	20	28.0	23.3	-4.7
3C	3/26/2023 3:00	20	28.0	23.3	-4.7
3C	3/26/2023 4:00	20	28.0	23.3	-4.7
3C	3/26/2023 5:00	20	28.0	23.3	-4.7
3C	3/26/2023 6:00	20	28.0	23.3	-4.7
3C	3/26/2023 7:00	20	28.0	23.3	-4.7
3C	3/26/2023 8:00	20	28.0	18.3	-9.7
3C	3/26/2023 9:00	20	28.0	18.3	-9.7
3C	3/26/2023 10:00	20	28.0	18.3	-9.7
3C	3/26/2023 11:00	20	28.0	18.3	-9.7
3C	3/26/2023 12:00	20	28.0	18.3	-9.7
3C	3/26/2023 13:00	20	28.0	18.3	-9.7
3C	3/26/2023 14:00	18	23.0	18.3	-4.7
3C	3/26/2023 15:00	18	23.0	18.3	-4.7
3C	3/26/2023 16:00	18	23.0	23.4	0.4
3C	3/26/2023 17:00	18	23.0	23.4	0.4
3C	3/26/2023 18:00	18	23.0	23.4	0.4
3C	3/26/2023 19:00	19	23.0	23.4	0.4
3C	3/26/2023 20:00	19	23.0	17.6	-5.4
3C	3/26/2023 21:00	19	23.0	17.6	-5.4
3C	3/26/2023 22:00	19	23.0	17.6	-5.4
3C	3/26/2023 23:00	19	23.0	20.5	-2.5
3C	3/27/2023 0:00	19	23.0	20.0	-3.0
3C	3/27/2023 1:00	19	23.0	18.7	-4.3
3C	3/27/2023 2:00	19	23.0	18.7	-4.3
3C	3/27/2023 3:00	19	23.0	18.7	-4.3
3C	3/27/2023 4:00	19	23.0	18.7	-4.3
3C	3/27/2023 5:00	19	23.0	18.7	-4.3
3C	3/27/2023 6:00	19	23.0	18.7	-4.3
3C	3/27/2023 7:00	20	32.0	18.7	-13.3
3C	3/27/2023 8:00	20	32.0	20.3	-11.7
3C	3/27/2023 9:00	20	32.0	20.3	-11.7
3C	3/27/2023 10:00	20	32.0	20.3	-11.7
3C	3/27/2023 11:00	26	32.0	20.3	-11.7
3C	3/27/2023 12:00	22	32.0	25.4	-6.6
3C	3/27/2023 13:00	22	32.0	25.4	-6.6
3C	3/27/2023 14:00	22	32.0	25.4	-6.6
3C	3/27/2023 15:00	26	32.0	25.4	-6.6
3C	3/27/2023 16:00	26	32.0	25.8	-6.2
3C	3/27/2023 17:00	26	32.0	25.8	-6.2
3C	3/27/2023 18:00	26	32.0	25.8	-6.2

3C	3/27/2023 19:00	29	32.0	25.8	-6.2
3C	3/27/2023 20:00	29	32.0	21.1	-10.9
3C	3/27/2023 21:00	29	32.0	21.1	-10.9
3C	3/27/2023 22:00	29	32.0	21.1	-10.9
3C	3/27/2023 23:00	29	32.0	21.1	-10.9
3C	3/28/2023 0:00	29	32.0	26.7	-5.4
3C	3/28/2023 1:00	29	32.0	26.7	-5.4
3C	3/28/2023 2:00	29	32.0	26.7	-5.4
3C	3/28/2023 3:00	29	32.0	26.7	-5.4
3C	3/28/2023 4:00	29	32.0	26.7	-5.4
3C	3/28/2023 5:00	29	32.0	26.7	-5.4
3C	3/28/2023 6:00	29	32.0	26.7	-5.4
3C	3/28/2023 7:00	31	32.0	26.7	-5.4
3C	3/28/2023 8:00	31	32.0	20.3	-11.7
3C	3/28/2023 9:00	31	32.0	20.3	-11.7
3C	3/28/2023 10:00	31	32.0	20.3	-11.7
3C	3/28/2023 11:00	31	32.0	20.3	-11.7
3C	3/28/2023 12:00	31	32.0	22.9	-9.1
3C	3/28/2023 13:00	31	32.0	22.9	-9.1
3C	3/28/2023 14:00	31	32.0	22.9	-9.1
3C	3/28/2023 15:00	31	32.0	22.9	-9.1
3C	3/28/2023 16:00	31	32.0	26.4	-5.7
3C	3/28/2023 17:00	31	32.0	26.4	-5.7
3C	3/28/2023 18:00	31	32.0	26.4	-5.7
3C	3/28/2023 19:00	31	32.0	26.4	-5.7
3C	3/28/2023 20:00	31	32.0	20.5	-11.5
3C	3/28/2023 21:00	31	32.0	20.5	-11.5
3C	3/28/2023 22:00	31	32.0	20.5	-11.5
3C	3/28/2023 23:00	31	32.0	20.5	-11.5
3C	3/29/2023 0:00	31	32.0	23.3	-8.7
3C	3/29/2023 1:00	31	32.0	23.3	-8.7
3C	3/29/2023 2:00	31	32.0	23.3	-8.7
3C	3/29/2023 3:00	31	32.0	23.3	-8.7
3C	3/29/2023 4:00	31	32.0	23.3	-8.7
3C	3/29/2023 5:00	31	32.0	23.3	-8.7
3C	3/29/2023 6:00	31	32.0	23.3	-8.7
3C	3/29/2023 7:00	31	32.0	23.3	-8.7
3C	3/29/2023 8:00	31	32.0	22.4	-9.6
3C	3/29/2023 9:00	31	32.0	22.4	-9.6
3C	3/29/2023 10:00	31	32.0	22.4	-9.6
3C	3/29/2023 11:00	29	32.0	22.4	-9.6
3C	3/29/2023 12:00	29	32.0	22.4	-9.6
3C	3/29/2023 13:00	29	32.0	22.4	-9.6
3C	3/29/2023 14:00	29	32.0	22.4	-9.6
3C	3/29/2023 15:00	29	32.0	21.4	-10.6
3C	3/29/2023 16:00	25	31.0	17.6	-13.4
3C	3/29/2023 17:00	25	31.0	17.6	-13.4

3C	3/29/2023 18:00	25	31.0	17.6	-13.4
3C	3/29/2023 19:00	25	31.0	17.6	-13.4
3C	3/29/2023 20:00	25	31.0	32.2	1.2
3C	3/29/2023 21:00	25	31.0	32.2	1.2
3C	3/29/2023 22:00	25	31.0	32.2	1.2
3C	3/29/2023 23:00	25	31.0	32.2	1.2
3C	3/30/2023 0:00	25	31.0	32.0	1.0
3C	3/30/2023 1:00	25	31.0	32.0	1.0
3C	3/30/2023 2:00	25	31.0	32.0	1.0
3C	3/30/2023 3:00	25	31.0	32.0	1.0
3C	3/30/2023 4:00	25	31.0	32.0	1.0
3C	3/30/2023 5:00	25	31.0	32.0	1.0
3C	3/30/2023 6:00	25	31.0	32.0	1.0
3C	3/30/2023 7:00	29	34.0	32.0	-2.0
3C	3/30/2023 8:00	29	34.0	25.4	-8.6
3C	3/30/2023 9:00	29	34.0	25.4	-8.6
3C	3/30/2023 10:00	29	34.0	25.4	-8.6
3C	3/30/2023 11:00	29	34.0	25.4	-8.6
3C	3/30/2023 12:00	29	34.0	25.4	-8.6
3C	3/30/2023 13:00	29	34.0	25.4	-8.6
3C	3/30/2023 14:00	29	34.0	25.4	-8.6
3C	3/30/2023 15:00	29	34.0	25.4	-8.6
3C	3/30/2023 16:00	23	28.0	26.4	-1.7
3C	3/30/2023 17:00	23	28.0	26.4	-1.7
3C	3/30/2023 18:00	23	28.0	26.4	-1.7
3C	3/30/2023 19:00	23	28.0	26.4	-1.7
3C	3/30/2023 20:00	27	27.0	23.4	-3.6
3C	3/30/2023 21:00	27	27.0	23.4	-3.6
3C	3/30/2023 22:00	27	27.0	23.4	-3.6
3C	3/30/2023 23:00	27	27.0	23.4	-3.6
3C	3/31/2023 0:00	27	27.0	23.3	-3.7
3C	3/31/2023 1:00	27	27.0	23.3	-3.7
3C	3/31/2023 2:00	27	27.0	23.3	-3.7
3C	3/31/2023 3:00	27	27.0	23.3	-3.7
3C	3/31/2023 4:00	27	27.0	23.3	-3.7
3C	3/31/2023 5:00	27	27.0	23.3	-3.7
3C	3/31/2023 6:00	27	27.0	23.3	-3.7
3C	3/31/2023 7:00	27	27.0	23.3	-3.7
3C	3/31/2023 8:00	27	27.0	22.9	-4.1
3C	3/31/2023 9:00	27	27.0	22.9	-4.1
3C	3/31/2023 10:00	27	27.0	22.9	-4.1
3C	3/31/2023 11:00	27	27.0	22.9	-4.1
3C	3/31/2023 12:00	27	27.0	25.4	-1.6
3C	3/31/2023 13:00	27	27.0	25.4	-1.6
3C	3/31/2023 14:00	27	27.0	25.4	-1.6
3C	3/31/2023 15:00	27	27.0	25.4	-1.6
3C	3/31/2023 16:00	27	27.0	23.4	-3.6

3C	3/31/2023 17:00	27	27.0	23.4	-3.6
3C	3/31/2023 18:00	27	27.0	23.4	-3.6
3C	3/31/2023 19:00	27	27.0	23.4	-3.6
3C	3/31/2023 20:00	27	27.0	25.8	-1.2
3C	3/31/2023 21:00	27	27.0	25.8	-1.2
3C	3/31/2023 22:00	27	27.0	25.8	-1.2
3C	3/31/2023 23:00	27	27.0	24.6	-2.4
3C	4/1/2023 0:00	23	30.0	22.7	-7.4
3C	4/1/2023 1:00	23	30.0	23.3	-6.7
3C	4/1/2023 2:00	23	30.0	23.3	-6.7
3C	4/1/2023 3:00	23	30.0	23.3	-6.7
3C	4/1/2023 4:00	23	30.0	23.3	-6.7
3C	4/1/2023 5:00	23	30.0	23.3	-6.7
3C	4/1/2023 6:00	23	30.0	23.3	-6.7
3C	4/1/2023 7:00	24	30.0	22.7	-7.4
3C	4/1/2023 8:00	24	30.0	20.3	-9.7
3C	4/1/2023 9:00	24	30.0	20.3	-9.7
3C	4/1/2023 10:00	24	30.0	20.3	-9.7
3C	4/1/2023 11:00	24	30.0	20.3	-9.7
3C	4/1/2023 12:00	24	30.0	20.3	-9.7
3C	4/1/2023 13:00	24	30.0	20.3	-9.7
3C	4/1/2023 14:00	24	30.0	20.3	-9.7
3C	4/1/2023 15:00	22	30.0	20.3	-9.7
3C	4/1/2023 16:00	22	30.0	23.4	-6.6
3C	4/1/2023 17:00	22	30.0	23.4	-6.6
3C	4/1/2023 18:00	22	30.0	23.4	-6.6
3C	4/1/2023 19:00	22	30.0	23.4	-6.6
3C	4/1/2023 20:00	22	30.0	18.7	-11.3
3C	4/1/2023 21:00	22	30.0	18.7	-11.3
3C	4/1/2023 22:00	23	30.0	18.7	-11.3
3C	4/1/2023 23:00	23	30.0	18.7	-11.3
3C	4/2/2023 0:00	23	30.0	26.7	-3.4
3C	4/2/2023 1:00	23	30.0	26.7	-3.4
3C	4/2/2023 2:00	23	30.0	26.7	-3.4
3C	4/2/2023 3:00	23	30.0	26.7	-3.4
3C	4/2/2023 4:00	23	30.0	26.7	-3.4
3C	4/2/2023 5:00	23	30.0	26.7	-3.4
3C	4/2/2023 6:00	23	30.0	26.7	-3.4
3C	4/2/2023 7:00	23	30.0	26.7	-3.4
3C	4/2/2023 8:00	23	30.0	17.8	-12.2
3C	4/2/2023 9:00	23	30.0	17.8	-12.2
3C	4/2/2023 10:00	23	30.0	17.8	-12.2
3C	4/2/2023 11:00	23	30.0	17.8	-12.2
3C	4/2/2023 12:00	23	30.0	22.4	-7.6
3C	4/2/2023 13:00	23	30.0	22.4	-7.6
3C	4/2/2023 14:00	23	30.0	22.4	-7.6
3C	4/2/2023 15:00	21	30.0	22.4	-7.6

3C	4/2/2023 16:00	21	30.0	23.4	-6.6
3C	4/2/2023 17:00	21	30.0	23.4	-6.6
3C	4/2/2023 18:00	21	30.0	23.4	-6.6
3C	4/2/2023 19:00	21	30.0	23.4	-6.6
3C	4/2/2023 20:00	21	30.0	23.4	-6.6
3C	4/2/2023 21:00	21	30.0	23.4	-6.6
3C	4/2/2023 22:00	21	30.0	23.4	-6.6
3C	4/2/2023 23:00	21	30.0	23.4	-6.6
3C	4/3/2023 0:00	21	30.0	26.7	-3.4
3C	4/3/2023 1:00	21	30.0	26.7	-3.4
3C	4/3/2023 2:00	21	30.0	26.7	-3.4
3C	4/3/2023 3:00	21	30.0	26.7	-3.4
3C	4/3/2023 4:00	22	30.0	26.7	-3.4
3C	4/3/2023 5:00	22	30.0	26.7	-3.4
3C	4/3/2023 6:00	22	30.0	26.7	-3.4
3C	4/3/2023 7:00	22	30.0	26.7	-3.4
3C	4/3/2023 8:00	22	30.0	22.4	-7.6
3C	4/3/2023 9:00	22	30.0	22.4	-7.6
3C	4/3/2023 10:00	22	30.0	22.4	-7.6
3C	4/3/2023 11:00	23	30.0	22.4	-7.6
3C	4/3/2023 12:00	23	30.0	24.4	-5.6
3C	4/3/2023 13:00	23	30.0	24.4	-5.6
3C	4/3/2023 14:00	23	30.0	24.4	-5.6
3C	4/3/2023 15:00	27	30.0	24.4	-5.6
3C	4/3/2023 16:00	27	30.0	23.4	-6.6
3C	4/3/2023 17:00	27	30.0	23.4	-6.6
3C	4/3/2023 18:00	27	30.0	23.4	-6.6
3C	4/3/2023 19:00	27	30.0	23.4	-6.6
3C	4/3/2023 20:00	27	30.0	17.6	-12.4
3C	4/3/2023 21:00	27	30.0	17.6	-12.4
3C	4/3/2023 22:00	27	30.0	17.6	-12.4
3C	4/3/2023 23:00	27	30.0	17.6	-12.4
3C	4/4/2023 0:00	27	30.0	26.7	-3.4
3C	4/4/2023 1:00	27	30.0	26.7	-3.4
3C	4/4/2023 2:00	27	30.0	26.7	-3.4
3C	4/4/2023 3:00	27	30.0	26.7	-3.4
3C	4/4/2023 4:00	29	33.0	26.7	-6.4
3C	4/4/2023 5:00	29	33.0	26.7	-6.4
3C	4/4/2023 6:00	29	33.0	26.7	-6.4
3C	4/4/2023 7:00	29	33.0	26.7	-6.4
3C	4/4/2023 8:00	29	33.0	25.4	-7.6
3C	4/4/2023 9:00	29	33.0	25.4	-7.6
3C	4/4/2023 10:00	29	33.0	25.4	-7.6
3C	4/4/2023 11:00	29	33.0	25.4	-7.6
3C	4/4/2023 12:00	29	33.0	20.3	-12.7
3C	4/4/2023 13:00	23	23.0	20.3	-2.7
3C	4/4/2023 14:00	23	23.0	20.3	-2.7

3C	4/4/2023 15:00	23	23.0	20.3	-2.7
3C	4/4/2023 16:00	23	23.0	23.4	0.4
3C	4/4/2023 17:00	23	23.0	23.4	0.4
3C	4/4/2023 18:00	23	23.0	23.4	0.4
3C	4/4/2023 19:00	23	22.0	23.4	1.4
3C	4/4/2023 20:00	23	22.0	11.7	-10.3
3C	4/4/2023 21:00	23	22.0	11.7	-10.3
3C	4/4/2023 22:00	23	22.0	11.7	-10.3
3C	4/4/2023 23:00	23	22.0	11.7	-10.3
3C	4/5/2023 0:00	23	22.0	16.7	-5.3
3C	4/5/2023 1:00	23	22.0	16.7	-5.3
3C	4/5/2023 2:00	23	22.0	16.7	-5.3
3C	4/5/2023 3:00	23	22.0	16.7	-5.3
3C	4/5/2023 4:00	23	22.0	16.7	-5.3
3C	4/5/2023 5:00	23	22.0	16.7	-5.3
3C	4/5/2023 6:00	23	22.0	16.7	-5.3
3C	4/5/2023 7:00	22	34.0	16.7	-17.3
3C	4/5/2023 8:00	22	34.0	28.0	-6.0
3C	4/5/2023 9:00	22	34.0	28.0	-6.0
3C	4/5/2023 10:00	22	34.0	28.0	-6.0
3C	4/5/2023 11:00	22	34.0	28.0	-6.0
3C	4/5/2023 12:00	22	34.0	28.0	-6.0
3C	4/5/2023 13:00	22	34.0	26.1	-7.9
3C	4/5/2023 14:00	22	34.0	25.4	-8.6
3C	4/5/2023 15:00	20	28.0	25.4	-2.6
3C	4/5/2023 16:00	20	28.0	29.3	1.3
3C	4/5/2023 17:00	20	28.0	29.3	1.3
3C	4/5/2023 18:00	20	28.0	29.3	1.3
3C	4/5/2023 19:00	20	28.0	29.3	1.3
3C	4/5/2023 20:00	20	28.0	20.5	-7.5
3C	4/5/2023 21:00	20	28.0	20.5	-7.5
3C	4/5/2023 22:00	20	28.0	20.5	-7.5
3C	4/5/2023 23:00	20	28.0	20.5	-7.5
3C	4/6/2023 0:00	20	28.0	23.3	-4.7
3C	4/6/2023 1:00	20	28.0	23.3	-4.7
3C	4/6/2023 2:00	20	28.0	23.3	-4.7
3C	4/6/2023 3:00	20	28.0	23.3	-4.7
3C	4/6/2023 4:00	20	28.0	23.3	-4.7
3C	4/6/2023 5:00	24	28.0	23.3	-4.7
3C	4/6/2023 6:00	24	28.0	23.3	-4.7
3C	4/6/2023 7:00	24	32.0	23.3	-8.7
3C	4/6/2023 8:00	24	32.0	20.3	-11.7
3C	4/6/2023 9:00	24	32.0	20.3	-11.7
3C	4/6/2023 10:00	24	32.0	20.3	-11.7
3C	4/6/2023 11:00	24	32.0	20.3	-11.7
3C	4/6/2023 12:00	24	32.0	22.9	-9.1
3C	4/6/2023 13:00	24	32.0	22.9	-9.1

3C	4/6/2023 14:00	24	32.0	22.9	-9.1
3C	4/6/2023 15:00	26	32.0	22.9	-9.1
3C	4/6/2023 16:00	26	32.0	23.4	-8.6
3C	4/6/2023 17:00	26	32.0	23.4	-8.6
3C	4/6/2023 18:00	26	32.0	23.4	-8.6
3C	4/6/2023 19:00	29	31.0	23.4	-7.6
3C	4/6/2023 20:00	29	31.0	24.6	-6.4
3C	4/6/2023 21:00	29	31.0	25.8	-5.2
3C	4/6/2023 22:00	29	31.0	25.8	-5.2
3C	4/6/2023 23:00	29	31.0	25.8	-5.2
3C	4/7/2023 0:00	29	31.0	24.0	-7.0
3C	4/7/2023 1:00	31	31.0	24.0	-7.0
3C	4/7/2023 2:00	31	31.0	24.0	-7.0
3C	4/7/2023 3:00	31	31.0	24.0	-7.0
3C	4/7/2023 4:00	31	31.0	24.0	-7.0
3C	4/7/2023 5:00	31	31.0	24.0	-7.0
3C	4/7/2023 6:00	31	31.0	24.0	-7.0
3C	4/7/2023 7:00	31	31.0	24.0	-7.0
3C	4/7/2023 8:00	31	31.0	22.9	-8.1
3C	4/7/2023 9:00	31	31.0	22.9	-8.1
3C	4/7/2023 10:00	31	31.0	22.9	-8.1
3C	4/7/2023 11:00	31	31.0	22.9	-8.1
3C	4/7/2023 12:00	31	31.0	22.9	-8.1
3C	4/7/2023 13:00	31	31.0	22.9	-8.1
3C	4/7/2023 14:00	31	31.0	22.9	-8.1
3C	4/7/2023 15:00	21	30.0	22.9	-7.1
3C	4/7/2023 16:00	21	30.0	23.4	-6.6
3C	4/7/2023 17:00	21	30.0	23.4	-6.6
3C	4/7/2023 18:00	21	30.0	23.4	-6.6
3C	4/7/2023 19:00	25	30.0	23.4	-6.6
3C	4/7/2023 20:00	25	30.0	25.8	-4.2
3C	4/7/2023 21:00	25	30.0	25.8	-4.2
3C	4/7/2023 22:00	25	30.0	25.8	-4.2
3C	4/7/2023 23:00	25	30.0	25.8	-4.2
3C	4/8/2023 0:00	25	30.0	26.7	-3.4
3C	4/8/2023 1:00	25	30.0	26.7	-3.4
3C	4/8/2023 2:00	25	30.0	26.7	-3.4
3C	4/8/2023 3:00	25	30.0	26.7	-3.4
3C	4/8/2023 4:00	25	30.0	26.7	-3.4
3C	4/8/2023 5:00	25	30.0	26.7	-3.4
3C	4/8/2023 6:00	25	30.0	26.7	-3.4
3C	4/8/2023 7:00	25	30.0	26.7	-3.4
3C	4/8/2023 8:00	25	30.0	22.4	-7.6
3C	4/8/2023 9:00	25	30.0	22.4	-7.6
3C	4/8/2023 10:00	25	30.0	22.4	-7.6
3C	4/8/2023 11:00	25	30.0	22.4	-7.6
3C	4/8/2023 12:00	25	30.0	22.4	-7.6

3C	4/8/2023 13:00	25	30.0	22.4	-7.6
3C	4/8/2023 14:00	25	30.0	22.4	-7.6
3C	4/8/2023 15:00	21	30.0	20.3	-9.7
3C	4/8/2023 16:00	21	30.0	25.8	-4.2
3C	4/8/2023 17:00	21	30.0	25.8	-4.2
3C	4/8/2023 18:00	21	30.0	25.8	-4.2
3C	4/8/2023 19:00	21	30.0	26.4	-3.7
3C	4/8/2023 20:00	21	25.0	17.6	-7.4
3C	4/8/2023 21:00	21	25.0	17.6	-7.4
3C	4/8/2023 22:00	21	25.0	17.6	-7.4
3C	4/8/2023 23:00	21	25.0	17.6	-7.4
3C	4/9/2023 0:00	21	25.0	20.0	-5.0
3C	4/9/2023 1:00	21	25.0	21.3	-3.7
3C	4/9/2023 2:00	21	25.0	21.3	-3.7
3C	4/9/2023 3:00	21	25.0	21.3	-3.7
3C	4/9/2023 4:00	21	25.0	21.3	-3.7
3C	4/9/2023 5:00	21	24.0	20.0	-4.0
3C	4/9/2023 6:00	21	24.0	20.0	-4.0
3C	4/9/2023 7:00	21	24.0	20.0	-4.0
3C	4/9/2023 8:00	21	24.0	16.3	-7.7
3C	4/9/2023 9:00	21	24.0	16.3	-7.7
3C	4/9/2023 10:00	21	24.0	16.3	-7.7
3C	4/9/2023 11:00	21	24.0	16.3	-7.7
3C	4/9/2023 12:00	21	24.0	16.3	-7.7
3C	4/9/2023 13:00	21	24.0	16.3	-7.7
3C	4/9/2023 14:00	19	23.0	18.3	-4.7
3C	4/9/2023 15:00	19	23.0	18.3	-4.7
3C	4/9/2023 16:00	19	23.0	20.5	-2.5
3C	4/9/2023 17:00	19	23.0	20.5	-2.5
3C	4/9/2023 18:00	19	23.0	20.5	-2.5
3C	4/9/2023 19:00	19	23.0	22.0	-1.0
3C	4/9/2023 20:00	19	23.0	17.6	-5.4
3C	4/9/2023 21:00	19	23.0	17.6	-5.4
3C	4/9/2023 22:00	19	23.0	17.6	-5.4
3C	4/9/2023 23:00	20	26.0	16.4	-9.6
3C	4/10/2023 0:00	20	26.0	21.3	-4.7
3C	4/10/2023 1:00	20	26.0	21.3	-4.7
3C	4/10/2023 2:00	20	26.0	21.3	-4.7
3C	4/10/2023 3:00	21	26.0	21.3	-4.7
3C	4/10/2023 4:00	21	26.0	21.3	-4.7
3C	4/10/2023 5:00	21	26.0	21.3	-4.7
3C	4/10/2023 6:00	21	26.0	21.3	-4.7
3C	4/10/2023 7:00	21	26.0	21.3	-4.7
3C	4/10/2023 8:00	21	33.0	18.3	-14.7
3C	4/10/2023 9:00	21	33.0	18.3	-14.7
3C	4/10/2023 10:00	21	33.0	18.3	-14.7
3C	4/10/2023 11:00	21	33.0	18.3	-14.7

3C	4/10/2023 12:00	22	34.0	18.3	-15.7
3C	4/10/2023 13:00	22	34.0	18.3	-15.7
3C	4/10/2023 14:00	22	34.0	18.3	-15.7
3C	4/10/2023 15:00	21	34.0	18.3	-15.7
3C	4/10/2023 16:00	21	34.0	14.1	-20.0
3C	4/10/2023 17:00	21	34.0	14.1	-20.0
3C	4/10/2023 18:00	21	34.0	14.1	-20.0
3C	4/10/2023 19:00	25	28.0	11.7	-16.3
3C	4/10/2023 20:00	25	28.0	16.4	-11.6
3C	4/10/2023 21:00	25	28.0	16.4	-11.6
3C	4/10/2023 22:00	25	28.0	16.4	-11.6
3C	4/10/2023 23:00	25	28.0	16.4	-11.6
3C	4/11/2023 0:00	25	28.0	24.0	-4.0
3C	4/11/2023 1:00	25	28.0	24.0	-4.0
3C	4/11/2023 2:00	25	28.0	24.0	-4.0
3C	4/11/2023 3:00	25	28.0	24.0	-4.0
3C	4/11/2023 4:00	25	28.0	24.0	-4.0
3C	4/11/2023 5:00	25	28.0	24.0	-4.0
3C	4/11/2023 6:00	25	30.0	26.7	-3.4
3C	4/11/2023 7:00	25	30.0	26.7	-3.4
3C	4/11/2023 8:00	25	30.0	20.3	-9.7
3C	4/11/2023 9:00	25	30.0	20.3	-9.7
3C	4/11/2023 10:00	25	30.0	20.3	-9.7
3C	4/11/2023 11:00	25	30.0	19.1	-10.9
3C	4/11/2023 12:00	25	30.0	17.8	-12.2
3C	4/11/2023 13:00	25	30.0	17.8	-12.2
3C	4/11/2023 14:00	25	30.0	17.8	-12.2
3C	4/11/2023 15:00	25	30.0	17.8	-12.2
3C	4/11/2023 16:00	25	30.0	20.5	-9.5
3C	4/11/2023 17:00	25	30.0	20.5	-9.5
3C	4/11/2023 18:00	25	30.0	20.5	-9.5
3C	4/11/2023 19:00	25	30.0	20.5	-9.5
3C	4/11/2023 20:00	25	30.0	23.4	-6.6
3C	4/11/2023 21:00	25	30.0	23.4	-6.6
3C	4/11/2023 22:00	25	30.0	23.4	-6.6
3C	4/11/2023 23:00	25	30.0	23.4	-6.6
3C	4/12/2023 0:00	25	30.0	26.7	-3.4
3C	4/12/2023 1:00	25	30.0	26.7	-3.4
3C	4/12/2023 2:00	25	30.0	26.7	-3.4
3C	4/12/2023 3:00	25	30.0	26.7	-3.4
3C	4/12/2023 4:00	25	30.0	26.7	-3.4
3C	4/12/2023 5:00	25	30.0	26.7	-3.4
3C	4/12/2023 6:00	25	30.0	23.3	-6.7
3C	4/12/2023 7:00	25	30.0	23.3	-6.7
3C	4/12/2023 8:00	25	30.0	20.3	-9.7
3C	4/12/2023 9:00	25	30.0	20.3	-9.7
3C	4/12/2023 10:00	25	30.0	20.3	-9.7

3C	4/12/2023 11:00	25	30.0	20.3	-9.7
3C	4/12/2023 12:00	25	30.0	17.8	-12.2
3C	4/12/2023 13:00	25	30.0	17.8	-12.2
3C	4/12/2023 14:00	20	26.0	17.8	-8.2
3C	4/12/2023 15:00	20	26.0	17.8	-8.2
3C	4/12/2023 16:00	20	26.0	20.5	-5.5
3C	4/12/2023 17:00	20	26.0	20.5	-5.5
3C	4/12/2023 18:00	20	26.0	20.5	-5.5
3C	4/12/2023 19:00	20	26.0	20.5	-5.5
3C	4/12/2023 20:00	20	26.0	20.5	-5.5
3C	4/12/2023 21:00	20	26.0	20.5	-5.5
3C	4/12/2023 22:00	20	26.0	20.5	-5.5
3C	4/12/2023 23:00	20	26.0	20.5	-5.5
3C	4/13/2023 0:00	20	26.0	29.3	3.3
3C	4/13/2023 1:00	20	26.0	29.3	3.3
3C	4/13/2023 2:00	20	26.0	29.3	3.3
3C	4/13/2023 3:00	20	26.0	29.3	3.3
3C	4/13/2023 4:00	20	26.0	29.3	3.3
3C	4/13/2023 5:00	20	26.0	29.3	3.3
3C	4/13/2023 6:00	20	26.0	29.3	3.3
3C	4/13/2023 7:00	23	30.0	29.3	-0.7
3C	4/13/2023 8:00	23	30.0	24.4	-5.6
3C	4/13/2023 9:00	23	30.0	24.4	-5.6
3C	4/13/2023 10:00	23	30.0	24.4	-5.6
3C	4/13/2023 11:00	23	30.0	24.4	-5.6
3C	4/13/2023 12:00	23	30.0	22.4	-7.6
3C	4/13/2023 13:00	23	30.0	22.4	-7.6
3C	4/13/2023 14:00	23	26.0	22.9	-3.1
3C	4/13/2023 15:00	23	26.0	22.9	-3.1
3C	4/13/2023 16:00	23	26.0	26.4	0.4
3C	4/13/2023 17:00	23	26.0	26.4	0.4
3C	4/13/2023 18:00	23	26.0	26.4	0.4
3C	4/13/2023 19:00	23	26.0	26.4	0.4
3C	4/13/2023 20:00	23	26.0	20.5	-5.5
3C	4/13/2023 21:00	23	26.0	20.5	-5.5
3C	4/13/2023 22:00	22	28.0	20.5	-7.5
3C	4/13/2023 23:00	23	28.0	20.5	-7.5
3C	4/14/2023 0:00	23	28.0	20.0	-8.0
3C	4/14/2023 1:00	23	28.0	20.0	-8.0
3C	4/14/2023 2:00	23	28.0	20.0	-8.0
3C	4/14/2023 3:00	23	28.0	18.7	-9.3
3C	4/14/2023 4:00	23	28.0	18.7	-9.3
3C	4/14/2023 5:00	23	28.0	18.7	-9.3
3C	4/14/2023 6:00	23	28.0	18.7	-9.3
3C	4/14/2023 7:00	23	27.0	18.7	-8.3
3C	4/14/2023 8:00	23	27.0	17.8	-9.2
3C	4/14/2023 9:00	21	27.0	17.8	-9.2

3C	4/14/2023 10:00	21	27.0	17.8	-9.2
3C	4/14/2023 11:00	21	27.0	17.8	-9.2
3C	4/14/2023 12:00	21	27.0	17.8	-9.2
3C	4/14/2023 13:00	21	27.0	17.8	-9.2
3C	4/14/2023 14:00	21	27.0	17.8	-9.2
3C	4/14/2023 15:00	21	27.0	17.8	-9.2
3C	4/14/2023 16:00	17	27.0	17.6	-9.4
3C	4/14/2023 17:00	17	22.0	16.8	-5.2
3C	4/14/2023 18:00	17	22.0	14.6	-7.4
3C	4/14/2023 19:00	19	28.0	14.6	-13.4
3C	4/14/2023 20:00	19	28.0	23.4	-4.6
3C	4/14/2023 21:00	20	20.0	23.4	3.4
3C	4/14/2023 22:00	20	20.0	23.4	3.4
3C	4/14/2023 23:00	20	24.0	23.4	-0.6
3C	4/15/2023 0:00	20	24.0	21.3	-2.7
3C	4/15/2023 1:00	20	24.0	21.3	-2.7
3C	4/15/2023 2:00	20	24.0	21.3	-2.7
3C	4/15/2023 3:00	20	24.0	21.3	-2.7
3C	4/15/2023 4:00	20	24.0	21.3	-2.7
3C	4/15/2023 5:00	20	24.0	21.3	-2.7
3C	4/15/2023 6:00	20	24.0	21.3	-2.7
3C	4/15/2023 7:00	20	24.0	21.3	-2.7
3C	4/15/2023 8:00	20	24.0	16.3	-7.7
3C	4/15/2023 9:00	20	24.0	16.3	-7.7
3C	4/15/2023 10:00	20	24.0	16.3	-7.7
3C	4/15/2023 11:00	20	24.0	16.3	-7.7
3C	4/15/2023 12:00	20	24.0	20.3	-3.7
3C	4/15/2023 13:00	20	24.0	20.3	-3.7
3C	4/15/2023 14:00	16	30.0	20.3	-9.7
3C	4/15/2023 15:00	16	30.0	20.3	-9.7
3C	4/15/2023 16:00	16	30.0	21.1	-8.9
3C	4/15/2023 17:00	16	30.0	21.1	-8.9
3C	4/15/2023 18:00	16	30.0	21.1	-8.9
3C	4/15/2023 19:00	18	24.0	17.6	-6.4
3C	4/15/2023 20:00	18	24.0	9.4	-14.6
3C	4/15/2023 21:00	18	24.0	9.4	-14.6
3C	4/15/2023 22:00	18	24.0	9.4	-14.6
3C	4/15/2023 23:00	18	24.0	9.4	-14.6
3C	4/16/2023 0:00	18	24.0	16.0	-8.0
3C	4/16/2023 1:00	18	24.0	16.0	-8.0
3C	4/16/2023 2:00	18	24.0	16.0	-8.0
3C	4/16/2023 3:00	18	24.0	16.0	-8.0
3C	4/16/2023 4:00	18	24.0	16.0	-8.0
3C	4/16/2023 5:00	18	24.0	16.0	-8.0
3C	4/16/2023 6:00	18	24.0	16.0	-8.0
3C	4/16/2023 7:00	18	30.0	16.0	-14.0
3C	4/16/2023 8:00	18	30.0	14.2	-15.8

3C	4/16/2023 9:00	18	30.0	14.2	-15.8
3C	4/16/2023 10:00	18	30.0	14.2	-15.8
3C	4/16/2023 11:00	18	30.0	15.3	-14.7
3C	4/16/2023 12:00	18	30.0	16.3	-13.7
3C	4/16/2023 13:00	18	30.0	16.3	-13.7
3C	4/16/2023 14:00	18	30.0	16.3	-13.7
3C	4/16/2023 15:00	18	30.0	16.3	-13.7
3C	4/16/2023 16:00	18	30.0	14.1	-16.0
3C	4/16/2023 17:00	18	30.0	14.1	-16.0
3C	4/16/2023 18:00	18	30.0	14.1	-16.0
3C	4/16/2023 19:00	19	30.0	14.1	-16.0
3C	4/16/2023 20:00	19	30.0	16.4	-13.6
3C	4/16/2023 21:00	19	30.0	16.4	-13.6
3C	4/16/2023 22:00	19	30.0	16.4	-13.6
3C	4/16/2023 23:00	19	30.0	16.4	-13.6
3C	4/17/2023 0:00	19	30.0	21.3	-8.7
3C	4/17/2023 1:00	19	30.0	21.3	-8.7
3C	4/17/2023 2:00	19	30.0	21.3	-8.7
3C	4/17/2023 3:00	19	30.0	21.3	-8.7
3C	4/17/2023 4:00	19	30.0	21.3	-8.7
3C	4/17/2023 5:00	19	30.0	21.3	-8.7
3C	4/17/2023 6:00	19	28.0	21.3	-6.7
3C	4/17/2023 7:00	19	28.0	21.3	-6.7
3C	4/17/2023 8:00	19	28.0	20.3	-7.7
3C	4/17/2023 9:00	19	30.0	20.3	-9.7
3C	4/17/2023 10:00	19	30.0	20.3	-9.7
3C	4/17/2023 11:00	19	30.0	20.3	-9.7
3C	4/17/2023 12:00	19	28.0	24.4	-3.6
3C	4/17/2023 13:00	18	28.0	24.4	-3.6
3C	4/17/2023 14:00	18	28.0	24.4	-3.6
3C	4/17/2023 15:00	18	23.0	22.4	-0.6
3C	4/17/2023 16:00	18	23.0	23.4	0.4
3C	4/17/2023 17:00	18	23.0	23.4	0.4
3C	4/17/2023 18:00	18	23.0	23.4	0.4
3C	4/17/2023 19:00	18	23.0	23.4	0.4
3C	4/17/2023 20:00	18	23.0	14.1	-9.0
3C	4/17/2023 21:00	18	23.0	14.1	-9.0
3C	4/17/2023 22:00	18	23.0	14.1	-9.0
3C	4/17/2023 23:00	18	23.0	14.1	-9.0
3C	4/18/2023 0:00	18	23.0	18.7	-4.3
3C	4/18/2023 1:00	18	23.0	18.7	-4.3
3C	4/18/2023 2:00	18	23.0	18.7	-4.3
3C	4/18/2023 3:00	18	23.0	18.7	-4.3
3C	4/18/2023 4:00	18	23.0	18.7	-4.3
3C	4/18/2023 5:00	18	23.0	18.7	-4.3
3C	4/18/2023 6:00	20	23.0	20.0	-3.0
3C	4/18/2023 7:00	20	23.0	20.0	-3.0

3C	4/18/2023 8:00	20	28.0	25.4	-2.6
3C	4/18/2023 9:00	20	28.0	25.4	-2.6
3C	4/18/2023 10:00	20	28.0	25.4	-2.6
3C	4/18/2023 11:00	18	28.0	25.4	-2.6
3C	4/18/2023 12:00	18	28.0	28.0	0.0
3C	4/18/2023 13:00	18	28.0	28.0	0.0
3C	4/18/2023 14:00	20	28.0	28.0	0.0
3C	4/18/2023 15:00	20	28.0	28.0	0.0
3C	4/18/2023 16:00	20	23.0	31.0	8.0
3C	4/18/2023 17:00	20	23.0	28.1	5.1
3C	4/18/2023 18:00	20	23.0	28.1	5.1
3C	4/18/2023 19:00	21	23.0	28.1	5.1
3C	4/18/2023 20:00	21	23.0	20.5	-2.5
3C	4/18/2023 21:00	21	23.0	20.5	-2.5
3C	4/18/2023 22:00	21	23.0	20.5	-2.5
3C	4/18/2023 23:00	21	23.0	20.5	-2.5
3C	4/19/2023 0:00	21	23.0	16.7	-6.3
3C	4/19/2023 1:00	21	23.0	16.7	-6.3
3C	4/19/2023 2:00	21	23.0	16.7	-6.3
3C	4/19/2023 3:00	21	23.0	16.7	-6.3
3C	4/19/2023 4:00	21	23.0	16.7	-6.3
3C	4/19/2023 5:00	21	23.0	16.7	-6.3
3C	4/19/2023 6:00	21	23.0	16.7	-6.3
3C	4/19/2023 7:00	21	23.0	16.7	-6.3
3C	4/19/2023 8:00	21	23.0	20.3	-2.7
3C	4/19/2023 9:00	21	23.0	20.3	-2.7
3C	4/19/2023 10:00	21	23.0	20.3	-2.7
3C	4/19/2023 11:00	21	23.0	19.3	-3.7
3C	4/19/2023 12:00	20	28.0	22.4	-5.6
3C	4/19/2023 13:00	20	28.0	22.4	-5.6
3C	4/19/2023 14:00	20	28.0	22.4	-5.6
3C	4/19/2023 15:00	20	28.0	22.4	-5.6
3C	4/19/2023 16:00	20	28.0	25.8	-2.2
3C	4/19/2023 17:00	20	28.0	25.8	-2.2
3C	4/19/2023 18:00	20	28.0	25.8	-2.2
3C	4/19/2023 19:00	20	28.0	25.8	-2.2
3C	4/19/2023 20:00	20	28.0	17.6	-10.4
3C	4/19/2023 21:00	20	28.0	17.6	-10.4
3C	4/19/2023 22:00	20	28.0	17.6	-10.4
3C	4/19/2023 23:00	20	28.0	17.6	-10.4
3C	4/20/2023 0:00	20	28.0	20.0	-8.0
3C	4/20/2023 1:00	20	28.0	20.0	-8.0
3C	4/20/2023 2:00	20	28.0	20.0	-8.0
3C	4/20/2023 3:00	20	28.0	20.0	-8.0
3C	4/20/2023 4:00	20	28.0	20.0	-8.0
3C	4/20/2023 5:00	20	28.0	20.0	-8.0
3C	4/20/2023 6:00	20	28.0	20.0	-8.0

3C	4/20/2023 7:00	20	28.0	20.0	-8.0
3C	4/20/2023 8:00	20	28.0	20.3	-7.7
3C	4/20/2023 9:00	20	28.0	20.3	-7.7
3C	4/20/2023 10:00	20	28.0	20.3	-7.7
3C	4/20/2023 11:00	20	28.0	20.3	-7.7
3C	4/20/2023 12:00	20	28.0	15.3	-12.7
3C	4/20/2023 13:00	20	28.0	15.3	-12.7
3C	4/20/2023 14:00	20	28.0	15.3	-12.7
3C	4/20/2023 15:00	17	27.0	15.9	-11.1
3C	4/20/2023 16:00	17	27.0	17.6	-9.4
3C	4/20/2023 17:00	17	27.0	17.6	-9.4
3C	4/20/2023 18:00	17	27.0	17.6	-9.4
3C	4/20/2023 19:00	17	23.0	17.6	-5.4
3C	4/20/2023 20:00	18	23.0	17.6	-5.4
3C	4/20/2023 21:00	18	23.0	17.6	-5.4
3C	4/20/2023 22:00	18	23.0	17.6	-5.4
3C	4/20/2023 23:00	18	23.0	17.6	-5.4
3C	4/21/2023 0:00	18	23.0	18.7	-4.3
3C	4/21/2023 1:00	18	23.0	18.7	-4.3
3C	4/21/2023 2:00	18	23.0	18.7	-4.3
3C	4/21/2023 3:00	18	23.0	18.7	-4.3
3C	4/21/2023 4:00	18	23.0	18.7	-4.3
3C	4/21/2023 5:00	18	23.0	18.7	-4.3
3C	4/21/2023 6:00	18	23.0	18.7	-4.3
3C	4/21/2023 7:00	18	23.0	18.7	-4.3
3C	4/21/2023 8:00	18	23.0	20.3	-2.7
3C	4/21/2023 9:00	18	23.0	20.3	-2.7
3C	4/21/2023 10:00	18	23.0	20.3	-2.7
3C	4/21/2023 11:00	18	23.0	20.3	-2.7
3C	4/21/2023 12:00	18	23.0	20.3	-2.7
3C	4/21/2023 13:00	18	23.0	20.3	-2.7
3C	4/21/2023 14:00	18	23.0	20.3	-2.7
3C	4/21/2023 15:00	18	23.0	20.3	-2.7
3C	4/21/2023 16:00	18	23.0	17.6	-5.4
3C	4/21/2023 17:00	18	23.0	17.6	-5.4
3C	4/21/2023 18:00	18	23.0	17.6	-5.4
3C	4/21/2023 19:00	15	23.0	17.6	-5.4
3C	4/21/2023 20:00	15	23.0	20.5	-2.5
3C	4/21/2023 21:00	15	23.0	20.5	-2.5
3C	4/21/2023 22:00	15	28.0	17.6	-10.4
3C	4/21/2023 23:00	15	28.0	17.6	-10.4
3C	4/22/2023 0:00	15	28.0	20.0	-8.0
3C	4/22/2023 1:00	15	28.0	20.0	-8.0
3C	4/22/2023 2:00	15	28.0	20.0	-8.0
3C	4/22/2023 3:00	15	28.0	20.0	-8.0
3C	4/22/2023 4:00	15	28.0	20.0	-8.0
3C	4/22/2023 5:00	15	28.0	20.0	-8.0

3C	4/22/2023 6:00	15	28.0	20.0	-8.0
3C	4/22/2023 7:00	15	28.0	20.0	-8.0
3C	4/22/2023 8:00	16	28.0	20.3	-7.7
3C	4/22/2023 9:00	16	28.0	20.3	-7.7
3C	4/22/2023 10:00	16	28.0	20.3	-7.7
3C	4/22/2023 11:00	16	28.0	20.3	-7.7
3C	4/22/2023 12:00	16	28.0	22.9	-5.1
3C	4/22/2023 13:00	16	28.0	22.9	-5.1
3C	4/22/2023 14:00	16	28.0	22.9	-5.1
3C	4/22/2023 15:00	16	28.0	22.9	-5.1
3C	4/22/2023 16:00	16	28.0	26.4	-1.7
3C	4/22/2023 17:00	16	28.0	26.4	-1.7
3C	4/22/2023 18:00	16	28.0	26.4	-1.7
3C	4/22/2023 19:00	16	28.0	26.4	-1.7
3C	4/22/2023 20:00	17	21.0	14.6	-6.4
3C	4/22/2023 21:00	17	21.0	14.6	-6.4
3C	4/22/2023 22:00	17	21.0	14.6	-6.4
3C	4/22/2023 23:00	17	21.0	14.6	-6.4
3C	4/23/2023 0:00	17	21.0	16.7	-4.3
3C	4/23/2023 1:00	17	21.0	16.7	-4.3
3C	4/23/2023 2:00	17	21.0	16.7	-4.3
3C	4/23/2023 3:00	17	21.0	16.7	-4.3
3C	4/23/2023 4:00	17	21.0	16.7	-4.3
3C	4/23/2023 5:00	17	21.0	16.7	-4.3
3C	4/23/2023 6:00	17	21.0	16.7	-4.3
3C	4/23/2023 7:00	17	21.0	16.7	-4.3
3C	4/23/2023 8:00	17	30.0	22.4	-7.6
3C	4/23/2023 9:00	17	30.0	22.4	-7.6
3C	4/23/2023 10:00	17	30.0	22.4	-7.6
3C	4/23/2023 11:00	17	30.0	22.4	-7.6
3C	4/23/2023 12:00	17	30.0	24.4	-5.6
3C	4/23/2023 13:00	17	30.0	24.4	-5.6
3C	4/23/2023 14:00	17	30.0	24.4	-5.6
3C	4/23/2023 15:00	18	30.0	24.4	-5.6
3C	4/23/2023 16:00	18	30.0	32.2	2.2
3C	4/23/2023 17:00	18	30.0	32.2	2.2
3C	4/23/2023 18:00	18	30.0	32.2	2.2
3C	4/23/2023 19:00	19	21.0	32.2	11.2
3C	4/23/2023 20:00	19	21.0	20.5	-0.5
3C	4/23/2023 21:00	19	21.0	20.5	-0.5
3C	4/23/2023 22:00	19	21.0	20.5	-0.5
3C	4/23/2023 23:00	19	21.0	20.5	-0.5
3C	4/24/2023 0:00	19	21.0	21.3	0.3
3C	4/24/2023 1:00	19	21.0	21.3	0.3
3C	4/24/2023 2:00	19	21.0	21.3	0.3
3C	4/24/2023 3:00	19	21.0	21.3	0.3
3C	4/24/2023 4:00	19	21.0	21.3	0.3

3C	4/24/2023 5:00	19	26.0	21.3	-4.7
3C	4/24/2023 6:00	19	26.0	21.3	-4.7
3C	4/24/2023 7:00	19	26.0	21.3	-4.7
3C	4/24/2023 8:00	19	26.0	20.3	-5.7
3C	4/24/2023 9:00	19	26.0	20.3	-5.7
3C	4/24/2023 10:00	19	26.0	20.3	-5.7
3C	4/24/2023 11:00	19	26.0	20.3	-5.7
3C	4/24/2023 12:00	19	26.0	22.9	-3.1
3C	4/24/2023 13:00	19	26.0	22.9	-3.1
3C	4/24/2023 14:00	19	26.0	22.9	-3.1
3C	4/24/2023 15:00	14	26.0	22.9	-3.1
3C	4/24/2023 16:00	14	26.0	21.1	-4.9
3C	4/24/2023 17:00	14	26.0	21.1	-4.9
3C	4/24/2023 18:00	14	26.0	21.1	-4.9
3C	4/24/2023 19:00	19	28.0	23.4	-4.6
3C	4/24/2023 20:00	19	28.0	18.7	-9.3
3C	4/24/2023 21:00	19	28.0	18.7	-9.3
3C	4/24/2023 22:00	19	28.0	18.7	-9.3
3C	4/24/2023 23:00	19	28.0	18.7	-9.3
3C	4/25/2023 0:00	17	28.0	23.3	-4.7
3C	4/25/2023 1:00	17	28.0	23.3	-4.7
3C	4/25/2023 2:00	17	28.0	23.3	-4.7
3C	4/25/2023 3:00	17	28.0	23.3	-4.7
3C	4/25/2023 4:00	17	28.0	23.3	-4.7
3C	4/25/2023 5:00	17	28.0	23.3	-4.7
3C	4/25/2023 6:00	17	28.0	23.3	-4.7
3C	4/25/2023 7:00	17	28.0	23.3	-4.7
3C	4/25/2023 8:00	17	28.0	17.8	-10.2
3C	4/25/2023 9:00	17	28.0	17.8	-10.2
3C	4/25/2023 10:00	17	28.0	17.8	-10.2
3C	4/25/2023 11:00	17	28.0	17.8	-10.2
3C	4/25/2023 12:00	17	28.0	22.9	-5.1
3C	4/25/2023 13:00	17	28.0	22.9	-5.1
3C	4/25/2023 14:00	17	28.0	22.9	-5.1
3C	4/25/2023 15:00	17	28.0	22.9	-5.1
3C	4/25/2023 16:00	17	28.0	26.4	-1.7
3C	4/25/2023 17:00	17	28.0	26.4	-1.7
3C	4/25/2023 18:00	17	28.0	26.4	-1.7
3C	4/25/2023 19:00	23	28.0	25.8	-2.2
3C	4/25/2023 20:00	23	28.0	29.3	1.3
3C	4/25/2023 21:00	23	28.0	29.3	1.3
3C	4/25/2023 22:00	23	28.0	29.3	1.3
3C	4/25/2023 23:00	23	28.0	29.3	1.3
3C	4/26/2023 0:00	23	28.0	26.7	-1.4
3C	4/26/2023 1:00	23	28.0	26.7	-1.4
3C	4/26/2023 2:00	23	28.0	26.7	-1.4
3C	4/26/2023 3:00	23	28.0	26.7	-1.4

3C	4/26/2023 4:00	23	28.0	26.7	-1.4
3C	4/26/2023 5:00	23	28.0	26.7	-1.4
3C	4/26/2023 6:00	23	28.0	26.7	-1.4
3C	4/26/2023 7:00	23	28.0	26.7	-1.4
3C	4/26/2023 8:00	23	28.0	22.9	-5.1
3C	4/26/2023 9:00	23	28.0	22.9	-5.1
3C	4/26/2023 10:00	23	28.0	22.9	-5.1
3C	4/26/2023 11:00	23	28.0	22.9	-5.1
3C	4/26/2023 12:00	23	28.0	25.4	-2.6
3C	4/26/2023 13:00	23	28.0	25.4	-2.6
3C	4/26/2023 14:00	23	28.0	25.4	-2.6
3C	4/26/2023 15:00	23	28.0	25.4	-2.6
3C	4/26/2023 16:00	23	28.0	23.4	-4.6
3C	4/26/2023 17:00	23	28.0	23.4	-4.6
3C	4/26/2023 18:00	23	28.0	23.4	-4.6
3C	4/26/2023 19:00	23	28.0	23.4	-4.6
3C	4/26/2023 20:00	23	28.0	29.3	1.3
3C	4/26/2023 21:00	23	28.0	29.3	1.3
3C	4/26/2023 22:00	23	28.0	29.3	1.3
3C	4/26/2023 23:00	23	28.0	29.3	1.3
3C	4/27/2023 0:00	23	28.0	34.0	6.0
3C	4/27/2023 1:00	26	28.0	32.0	4.0
3C	4/27/2023 2:00	26	28.0	32.0	4.0
3C	4/27/2023 3:00	26	28.0	32.0	4.0
3C	4/27/2023 4:00	26	28.0	32.0	4.0
3C	4/27/2023 5:00	26	28.0	32.0	4.0
3C	4/27/2023 6:00	26	28.0	32.0	4.0
3C	4/27/2023 7:00	26	28.0	32.0	4.0
3C	4/27/2023 8:00	26	28.0	22.9	-5.1
3C	4/27/2023 9:00	26	28.0	22.9	-5.1
3C	4/27/2023 10:00	26	28.0	22.9	-5.1
3C	4/27/2023 11:00	26	28.0	22.9	-5.1
3C	4/27/2023 12:00	26	28.0	20.3	-7.7
3C	4/27/2023 13:00	26	28.0	20.3	-7.7
3C	4/27/2023 14:00	26	28.0	20.3	-7.7
3C	4/27/2023 15:00	21	22.0	20.3	-1.7
3C	4/27/2023 16:00	21	22.0	23.4	1.4
3C	4/27/2023 17:00	21	22.0	23.4	1.4
3C	4/27/2023 18:00	21	22.0	23.4	1.4
3C	4/27/2023 19:00	21	22.0	23.4	1.4
3C	4/27/2023 20:00	22	23.0	18.7	-4.3
3C	4/27/2023 21:00	22	23.0	18.7	-4.3
3C	4/27/2023 22:00	22	23.0	18.7	-4.3
3C	4/27/2023 23:00	22	23.0	18.7	-4.3
3C	4/28/2023 0:00	22	23.0	18.7	-4.3
3C	4/28/2023 1:00	22	23.0	18.7	-4.3
3C	4/28/2023 2:00	22	23.0	18.7	-4.3

3C	4/28/2023 3:00	22	23.0	18.7	-4.3
3C	4/28/2023 4:00	22	23.0	18.7	-4.3
3C	4/28/2023 5:00	22	23.0	18.7	-4.3
3C	4/28/2023 6:00	22	23.0	18.7	-4.3
3C	4/28/2023 7:00	22	23.0	20.0	-3.0
3C	4/28/2023 8:00	22	23.0	15.3	-7.7
3C	4/28/2023 9:00	22	23.0	15.3	-7.7
3C	4/28/2023 10:00	22	23.0	15.3	-7.7
3C	4/28/2023 11:00	22	23.0	15.3	-7.7
3C	4/28/2023 12:00	22	23.0	17.8	-5.2
3C	4/28/2023 13:00	22	23.0	17.8	-5.2
3C	4/28/2023 14:00	22	23.0	17.8	-5.2
3C	4/28/2023 15:00	22	23.0	17.8	-5.2
3C	4/28/2023 16:00	22	23.0	18.7	-4.3
3C	4/28/2023 17:00	22	23.0	18.7	-4.3
3C	4/28/2023 18:00	22	23.0	18.7	-4.3
3C	4/28/2023 19:00	20	34.0	16.4	-17.6
3C	4/28/2023 20:00	20	34.0	21.1	-12.9
3C	4/28/2023 21:00	20	34.0	21.1	-12.9
3C	4/28/2023 22:00	20	34.0	21.1	-12.9
3C	4/28/2023 23:00	20	34.0	21.1	-12.9
3C	4/29/2023 0:00	20	34.0	21.3	-12.7
3C	4/29/2023 1:00	20	34.0	21.3	-12.7
3C	4/29/2023 2:00	20	34.0	21.3	-12.7
3C	4/29/2023 3:00	20	34.0	21.3	-12.7
3C	4/29/2023 4:00	20	34.0	21.3	-12.7
3C	4/29/2023 5:00	20	34.0	21.3	-12.7
3C	4/29/2023 6:00	20	34.0	21.3	-12.7
3C	4/29/2023 7:00	20	34.0	24.0	-10.0
3C	4/29/2023 8:00	20	34.0	16.3	-17.7
3C	4/29/2023 9:00	20	34.0	16.3	-17.7
3C	4/29/2023 10:00	20	34.0	16.3	-17.7
3C	4/29/2023 11:00	20	34.0	16.3	-17.7
3C	4/29/2023 12:00	21	31.0	16.3	-14.7
3C	4/29/2023 13:00	21	31.0	16.3	-14.7
3C	4/29/2023 14:00	21	31.0	16.3	-14.7
3C	4/29/2023 15:00	19	26.0	15.3	-10.7
3C	4/29/2023 16:00	19	26.0	16.4	-9.6
3C	4/29/2023 17:00	19	26.0	16.4	-9.6
3C	4/29/2023 18:00	19	26.0	16.4	-9.6
3C	4/29/2023 19:00	21	26.0	16.4	-9.6
3C	4/29/2023 20:00	21	26.0	18.7	-7.3
3C	4/29/2023 21:00	21	26.0	18.7	-7.3
3C	4/29/2023 22:00	21	26.0	18.7	-7.3
3C	4/29/2023 23:00	21	26.0	18.7	-7.3
3C	4/30/2023 0:00	21	26.0	21.3	-4.7
3C	4/30/2023 1:00	21	26.0	21.3	-4.7

3C	4/30/2023 2:00	21	26.0	21.3	-4.7
3C	4/30/2023 3:00	21	26.0	21.3	-4.7
3C	4/30/2023 4:00	21	26.0	21.3	-4.7
3C	4/30/2023 5:00	25	26.0	23.3	-2.7
3C	4/30/2023 6:00	25	26.0	23.3	-2.7
3C	4/30/2023 7:00	25	28.0	21.3	-6.7
3C	4/30/2023 8:00	25	28.0	18.3	-9.7
3C	4/30/2023 9:00	25	28.0	18.3	-9.7
3C	4/30/2023 10:00	25	28.0	18.3	-9.7
3C	4/30/2023 11:00	25	28.0	18.3	-9.7
3C	4/30/2023 12:00	25	28.0	20.3	-7.7
3C	4/30/2023 13:00	25	28.0	20.3	-7.7
3C	4/30/2023 14:00	25	28.0	20.3	-7.7
3C	4/30/2023 15:00	23	27.0	20.3	-6.7
3C	4/30/2023 16:00	23	27.0	18.7	-8.3
3C	4/30/2023 17:00	23	27.0	18.7	-8.3
3C	4/30/2023 18:00	23	27.0	18.7	-8.3
3C	4/30/2023 19:00	24	26.0	16.4	-9.6
3C	4/30/2023 20:00	24	26.0	18.7	-7.3
3C	4/30/2023 21:00	24	26.0	18.7	-7.3
3C	4/30/2023 22:00	24	26.0	18.7	-7.3
3C	4/30/2023 23:00	24	26.0	21.1	-4.9
3C	5/1/2023 0:00	24	26.0	22.7	-3.4
3C	5/1/2023 1:00	24	26.0	24.0	-2.0
3C	5/1/2023 2:00	24	26.0	24.0	-2.0
3C	5/1/2023 3:00	24	26.0	24.0	-2.0
3C	5/1/2023 4:00	24	26.0	24.0	-2.0
3C	5/1/2023 5:00	24	26.0	26.7	0.7
3C	5/1/2023 6:00	24	26.0	26.7	0.7
3C	5/1/2023 7:00	23	30.0	24.0	-6.0
3C	5/1/2023 8:00	24	32.0	22.4	-9.6
3C	5/1/2023 9:00	24	32.0	22.4	-9.6
3C	5/1/2023 10:00	24	32.0	22.4	-9.6
3C	5/1/2023 11:00	24	32.0	22.4	-9.6
3C	5/1/2023 12:00	24	32.0	30.5	-1.5
3C	5/1/2023 13:00	24	32.0	30.5	-1.5
3C	5/1/2023 14:00	24	32.0	29.2	-2.8
3C	5/1/2023 15:00	23	33.0	28.0	-5.0
3C	5/1/2023 16:00	23	33.0	23.4	-9.6
3C	5/1/2023 17:00	23	33.0	23.4	-9.6
3C	5/1/2023 18:00	23	33.0	23.4	-9.6
3C	5/1/2023 19:00	27	30.0	23.4	-6.6
3C	5/1/2023 20:00	27	30.0	23.4	-6.6
3C	5/1/2023 21:00	27	30.0	23.4	-6.6
3C	5/1/2023 22:00	27	30.0	23.4	-6.6
3C	5/1/2023 23:00	27	30.0	23.4	-6.6
3C	5/2/2023 0:00	27	30.0	29.3	-0.7

3C	5/2/2023 1:00	27	30.0	29.3	-0.7
3C	5/2/2023 2:00	27	30.0	29.3	-0.7
3C	5/2/2023 3:00	27	30.0	29.3	-0.7
3C	5/2/2023 4:00	27	30.0	29.3	-0.7
3C	5/2/2023 5:00	27	30.0	29.3	-0.7
3C	5/2/2023 6:00	27	30.0	30.0	0.0
3C	5/2/2023 7:00	27	30.0	30.0	0.0
3C	5/2/2023 8:00	27	30.0	17.8	-12.2
3C	5/2/2023 9:00	27	30.0	17.8	-12.2
3C	5/2/2023 10:00	27	30.0	17.8	-12.2
3C	5/2/2023 11:00	27	30.0	17.8	-12.2
3C	5/2/2023 12:00	29	30.0	25.4	-4.6
3C	5/2/2023 13:00	29	30.0	25.4	-4.6
3C	5/2/2023 14:00	29	30.0	25.4	-4.6
3C	5/2/2023 15:00	27	28.0	25.4	-2.6
3C	5/2/2023 16:00	27	28.0	29.3	1.3
3C	5/2/2023 17:00	27	28.0	29.3	1.3
3C	5/2/2023 18:00	27	28.0	29.3	1.3
3C	5/2/2023 19:00	26	28.0	26.4	-1.7
3C	5/2/2023 20:00	26	28.0	26.4	-1.7
3C	5/2/2023 21:00	26	28.0	26.4	-1.7
3C	5/2/2023 22:00	26	28.0	26.4	-1.7
3C	5/2/2023 23:00	26	28.0	26.4	-1.7
3C	5/3/2023 0:00	26	28.0	23.3	-4.7
3C	5/3/2023 1:00	26	28.0	23.3	-4.7
3C	5/3/2023 2:00	26	28.0	23.3	-4.7
3C	5/3/2023 3:00	26	28.0	23.3	-4.7
3C	5/3/2023 4:00	26	28.0	23.3	-4.7
3C	5/3/2023 5:00	26	28.0	23.3	-4.7
3C	5/3/2023 6:00	26	28.0	23.3	-4.7
3C	5/3/2023 7:00	26	28.0	23.3	-4.7
3C	5/3/2023 8:00	26	28.0	17.8	-10.2
3C	5/3/2023 9:00	26	28.0	17.8	-10.2
3C	5/3/2023 10:00	26	28.0	17.8	-10.2
3C	5/3/2023 11:00	26	29.0	17.8	-11.2
3C	5/3/2023 12:00	26	29.0	21.4	-7.6
3C	5/3/2023 13:00	26	29.0	20.3	-8.7
3C	5/3/2023 14:00	26	29.0	20.3	-8.7
3C	5/3/2023 15:00	26	29.0	20.3	-8.7
3C	5/3/2023 16:00	26	29.0	21.1	-7.9
3C	5/3/2023 17:00	26	29.0	21.1	-7.9
3C	5/3/2023 18:00	26	29.0	21.1	-7.9
3C	5/3/2023 19:00	26	29.0	21.1	-7.9
3C	5/3/2023 20:00	26	29.0	20.5	-8.5
3C	5/3/2023 21:00	26	29.0	20.5	-8.5
3C	5/3/2023 22:00	26	29.0	20.5	-8.5
3C	5/3/2023 23:00	26	29.0	20.5	-8.5

3C	5/4/2023 0:00	26	29.0	18.7	-10.3
3C	5/4/2023 1:00	26	29.0	18.7	-10.3
3C	5/4/2023 2:00	26	29.0	18.7	-10.3
3C	5/4/2023 3:00	26	29.0	18.7	-10.3
3C	5/4/2023 4:00	26	29.0	18.7	-10.3
3C	5/4/2023 5:00	26	29.0	18.7	-10.3
3C	5/4/2023 6:00	26	29.0	18.7	-10.3
3C	5/4/2023 7:00	26	29.0	18.7	-10.3
3C	5/4/2023 8:00	28	28.0	15.3	-12.7
3C	5/4/2023 9:00	28	28.0	15.3	-12.7
3C	5/4/2023 10:00	28	28.0	15.3	-12.7
3C	5/4/2023 11:00	28	28.0	15.3	-12.7
3C	5/4/2023 12:00	27	28.0	20.3	-7.7
3C	5/4/2023 13:00	27	28.0	20.3	-7.7
3C	5/4/2023 14:00	27	28.0	20.3	-7.7
3C	5/4/2023 15:00	27	28.0	20.3	-7.7
3C	5/4/2023 16:00	27	28.0	20.5	-7.5
3C	5/4/2023 17:00	27	28.0	20.5	-7.5
3C	5/4/2023 18:00	27	28.0	20.5	-7.5
3C	5/4/2023 19:00	27	28.0	20.5	-7.5
3C	5/4/2023 20:00	27	28.0	17.6	-10.4
3C	5/4/2023 21:00	27	28.0	17.6	-10.4
3C	5/4/2023 22:00	27	28.0	17.6	-10.4
3C	5/4/2023 23:00	27	28.0	17.6	-10.4
3C	5/5/2023 0:00	27	28.0	16.7	-11.3
3C	5/5/2023 1:00	27	28.0	16.7	-11.3
3C	5/5/2023 2:00	27	28.0	16.7	-11.3
3C	5/5/2023 3:00	27	28.0	16.7	-11.3
3C	5/5/2023 4:00	27	28.0	16.7	-11.3
3C	5/5/2023 5:00	27	28.0	16.7	-11.3
3C	5/5/2023 6:00	27	28.0	16.7	-11.3
3C	5/5/2023 7:00	28	33.0	20.0	-13.0
3C	5/5/2023 8:00	28	33.0	24.2	-8.9
3C	5/5/2023 9:00	28	33.0	22.9	-10.1
3C	5/5/2023 10:00	28	33.0	22.9	-10.1
3C	5/5/2023 11:00	28	33.0	22.9	-10.1
3C	5/5/2023 12:00	28	33.0	25.4	-7.6
3C	5/5/2023 13:00	28	33.0	25.4	-7.6
3C	5/5/2023 14:00	28	33.0	25.4	-7.6
3C	5/5/2023 15:00	28	33.0	25.4	-7.6
3C	5/5/2023 16:00	27	33.0	23.4	-9.6
3C	5/5/2023 17:00	27	33.0	23.4	-9.6
3C	5/5/2023 18:00	27	28.0	23.4	-4.6
3C	5/5/2023 19:00	27	28.0	23.4	-4.6
3C	5/5/2023 20:00	27	28.0	20.5	-7.5
3C	5/5/2023 21:00	27	28.0	20.5	-7.5
3C	5/5/2023 22:00	27	28.0	20.5	-7.5

3C	5/5/2023 23:00	27	28.0	20.5	-7.5
3C	5/6/2023 0:00	27	28.0	16.7	-11.3
3C	5/6/2023 1:00	27	28.0	16.7	-11.3
3C	5/6/2023 2:00	27	28.0	16.7	-11.3
3C	5/6/2023 3:00	27	28.0	16.7	-11.3
3C	5/6/2023 4:00	27	28.0	16.7	-11.3
3C	5/6/2023 5:00	27	28.0	16.7	-11.3
3C	5/6/2023 6:00	27	28.0	16.7	-11.3
3C	5/6/2023 7:00	27	28.0	20.0	-8.0
3C	5/6/2023 8:00	27	28.0	22.9	-5.1
3C	5/6/2023 9:00	27	28.0	22.9	-5.1
3C	5/6/2023 10:00	27	28.0	22.4	-5.6
3C	5/6/2023 11:00	27	28.0	22.4	-5.6
3C	5/6/2023 12:00	27	28.0	24.4	-3.6
3C	5/6/2023 13:00	27	28.0	24.4	-3.6
3C	5/6/2023 14:00	27	28.0	24.4	-3.6
3C	5/6/2023 15:00	24	28.0	22.4	-5.6
3C	5/6/2023 16:00	24	28.0	24.6	-3.4
3C	5/6/2023 17:00	23	28.0	23.4	-4.6
3C	5/6/2023 18:00	24	28.0	23.4	-4.6
3C	5/6/2023 19:00	24	28.0	23.4	-4.6
3C	5/6/2023 20:00	24	28.0	22.3	-5.8
3C	5/6/2023 21:00	24	28.0	23.4	-4.6
3C	5/6/2023 22:00	24	28.0	23.4	-4.6
3C	5/6/2023 23:00	24	28.0	23.4	-4.6
3C	5/7/2023 0:00	24	28.0	23.3	-4.7
3C	5/7/2023 1:00	24	28.0	23.3	-4.7
3C	5/7/2023 2:00	24	28.0	23.3	-4.7
3C	5/7/2023 3:00	24	28.0	23.3	-4.7
3C	5/7/2023 4:00	24	28.0	23.3	-4.7
3C	5/7/2023 5:00	24	28.0	23.3	-4.7
3C	5/7/2023 6:00	24	28.0	23.3	-4.7
3C	5/7/2023 7:00	24	28.0	26.7	-1.4
3C	5/7/2023 8:00	28	30.0	18.3	-11.7
3C	5/7/2023 9:00	28	30.0	18.3	-11.7
3C	5/7/2023 10:00	28	30.0	18.3	-11.7
3C	5/7/2023 11:00	28	30.0	18.3	-11.7
3C	5/7/2023 12:00	28	30.0	22.4	-7.6
3C	5/7/2023 13:00	28	30.0	22.4	-7.6
3C	5/7/2023 14:00	27	30.0	22.4	-7.6
3C	5/7/2023 15:00	26	28.0	22.4	-5.6
3C	5/7/2023 16:00	26	28.0	23.4	-4.6
3C	5/7/2023 17:00	26	28.0	23.4	-4.6
3C	5/7/2023 18:00	26	28.0	23.4	-4.6
3C	5/7/2023 19:00	26	28.0	23.4	-4.6
3C	5/7/2023 20:00	26	28.0	23.4	-4.6
3C	5/7/2023 21:00	26	28.0	23.4	-4.6

3C	5/7/2023 22:00	26	28.0	23.4	-4.6
3C	5/7/2023 23:00	26	28.0	23.4	-4.6
3C	5/8/2023 0:00	26	28.0	22.7	-5.4
3C	5/8/2023 1:00	26	28.0	24.0	-4.0
3C	5/8/2023 2:00	26	28.0	24.0	-4.0
3C	5/8/2023 3:00	26	28.0	24.0	-4.0
3C	5/8/2023 4:00	26	28.0	24.0	-4.0
3C	5/8/2023 5:00	26	28.0	24.0	-4.0
3C	5/8/2023 6:00	26	28.0	24.0	-4.0
3C	5/8/2023 7:00	27	28.0	26.7	-1.4
3C	5/8/2023 8:00	27	32.0	20.3	-11.7
3C	5/8/2023 9:00	27	32.0	20.3	-11.7
3C	5/8/2023 10:00	27	32.0	20.3	-11.7
3C	5/8/2023 11:00	27	32.0	20.3	-11.7
3C	5/8/2023 12:00	27	32.0	20.3	-11.7
3C	5/8/2023 13:00	27	32.0	20.3	-11.7
3C	5/8/2023 14:00	27	32.0	20.3	-11.7
3C	5/8/2023 15:00	27	32.0	20.3	-11.7
3C	5/8/2023 16:00	27	32.0	17.6	-14.4
3C	5/8/2023 17:00	27	32.0	17.6	-14.4
3C	5/8/2023 18:00	27	32.0	17.6	-14.4
3C	5/8/2023 19:00	27	32.0	17.6	-14.4
3C	5/8/2023 20:00	27	32.0	17.6	-14.4
3C	5/8/2023 21:00	27	32.0	17.6	-14.4
3C	5/8/2023 22:00	27	32.0	17.6	-14.4
3C	5/8/2023 23:00	27	32.0	17.6	-14.4
3C	5/9/2023 0:00	27	32.0	23.3	-8.7
3C	5/9/2023 1:00	27	32.0	23.3	-8.7
3C	5/9/2023 2:00	27	32.0	23.3	-8.7
3C	5/9/2023 3:00	27	32.0	23.3	-8.7
3C	5/9/2023 4:00	27	32.0	23.3	-8.7
3C	5/9/2023 5:00	31	32.0	23.3	-8.7
3C	5/9/2023 6:00	31	32.0	23.3	-8.7
3C	5/9/2023 7:00	31	32.0	23.3	-8.7
3C	5/9/2023 8:00	31	32.0	22.4	-9.6
3C	5/9/2023 9:00	31	32.0	22.4	-9.6
3C	5/9/2023 10:00	31	32.0	22.4	-9.6
3C	5/9/2023 11:00	31	32.0	22.4	-9.6
3C	5/9/2023 12:00	31	32.0	22.4	-9.6
3C	5/9/2023 13:00	31	32.0	22.4	-9.6
3C	5/9/2023 14:00	31	32.0	22.4	-9.6
3C	5/9/2023 15:00	31	32.0	22.4	-9.6
3C	5/9/2023 16:00	31	32.0	23.4	-8.6
3C	5/9/2023 17:00	31	32.0	23.4	-8.6
3C	5/9/2023 18:00	31	32.0	23.4	-8.6
3C	5/9/2023 19:00	31	32.0	23.4	-8.6
3C	5/9/2023 20:00	31	32.0	20.5	-11.5

3C	5/9/2023 21:00	31	32.0	20.5	-11.5
3C	5/9/2023 22:00	31	32.0	20.5	-11.5
3C	5/9/2023 23:00	31	32.0	20.5	-11.5
3C	5/10/2023 0:00	31	32.0	20.0	-12.0
3C	5/10/2023 1:00	31	32.0	20.0	-12.0
3C	5/10/2023 2:00	31	32.0	20.0	-12.0
3C	5/10/2023 3:00	31	32.0	20.0	-12.0
3C	5/10/2023 4:00	31	32.0	20.0	-12.0
3C	5/10/2023 5:00	31	32.0	20.0	-12.0
3C	5/10/2023 6:00	31	32.0	20.0	-12.0
3C	5/10/2023 7:00	31	32.0	20.0	-12.0
3C	5/10/2023 8:00	31	32.0	20.3	-11.7
3C	5/10/2023 9:00	31	32.0	20.3	-11.7
3C	5/10/2023 10:00	31	32.0	20.3	-11.7
3C	5/10/2023 11:00	31	32.0	20.3	-11.7
3C	5/10/2023 12:00	31	32.0	25.4	-6.6
3C	5/10/2023 13:00	30	33.0	25.4	-7.6
3C	5/10/2023 14:00	30	33.0	25.4	-7.6
3C	5/10/2023 15:00	30	33.0	25.4	-7.6
3C	5/10/2023 16:00	30	33.0	23.4	-9.6
3C	5/10/2023 17:00	30	33.0	23.4	-9.6
3C	5/10/2023 18:00	29	33.0	26.4	-6.7
3C	5/10/2023 19:00	29	33.0	26.4	-6.7
3C	5/10/2023 20:00	29	33.0	25.8	-7.2
3C	5/10/2023 21:00	31	31.0	25.8	-5.2
3C	5/10/2023 22:00	31	31.0	25.8	-5.2
3C	5/10/2023 23:00	31	31.0	25.8	-5.2
3C	5/11/2023 0:00	31	31.0	24.0	-7.0
3C	5/11/2023 1:00	31	31.0	24.0	-7.0
3C	5/11/2023 2:00	31	31.0	24.0	-7.0
3C	5/11/2023 3:00	31	31.0	24.0	-7.0
3C	5/11/2023 4:00	31	31.0	24.0	-7.0
3C	5/11/2023 5:00	31	31.0	24.0	-7.0
3C	5/11/2023 6:00	31	31.0	25.3	-5.7
3C	5/11/2023 7:00	31	31.0	26.7	-4.4
3C	5/11/2023 8:00	31	33.0	22.9	-10.1
3C	5/11/2023 9:00	31	33.0	22.9	-10.1
3C	5/11/2023 10:00	31	33.0	22.9	-10.1
3C	5/11/2023 11:00	31	33.0	22.9	-10.1
3C	5/11/2023 12:00	31	33.0	22.9	-10.1
3C	5/11/2023 13:00	29	33.0	22.9	-10.1
3C	5/11/2023 14:00	29	33.0	22.9	-10.1
3C	5/11/2023 15:00	26	33.0	22.9	-10.1
3C	5/11/2023 16:00	26	33.0	24.6	-8.4
3C	5/11/2023 17:00	27	33.0	23.4	-9.6
3C	5/11/2023 18:00	27	33.0	23.4	-9.6
3C	5/11/2023 19:00	28	33.0	23.4	-9.6

3C	5/11/2023 20:00	28	33.0	19.9	-13.1
3C	5/11/2023 21:00	31	33.0	21.1	-11.9
3C	5/11/2023 22:00	31	33.0	21.1	-11.9
3C	5/11/2023 23:00	31	33.0	21.1	-11.9
3C	5/12/2023 0:00	33	33.0	20.0	-13.0
3C	5/12/2023 1:00	33	33.0	20.0	-13.0
3C	5/12/2023 2:00	33	33.0	20.0	-13.0
3C	5/12/2023 3:00	33	33.0	20.0	-13.0
3C	5/12/2023 4:00	33	33.0	20.0	-13.0
3C	5/12/2023 5:00	33	33.0	20.0	-13.0
3C	5/12/2023 6:00	33	33.0	20.0	-13.0
3C	5/12/2023 7:00	33	33.0	20.0	-13.0
3C	5/12/2023 8:00	33	33.0	20.3	-12.7
3C	5/12/2023 9:00	33	33.0	20.3	-12.7
3C	5/12/2023 10:00	33	33.0	20.3	-12.7
3C	5/12/2023 11:00	33	33.0	20.3	-12.7
3C	5/12/2023 12:00	33	33.0	22.9	-10.1
3C	5/12/2023 13:00	33	33.0	22.9	-10.1
3C	5/12/2023 14:00	33	33.0	22.9	-10.1
3C	5/12/2023 15:00	26	32.0	22.9	-9.1
3C	5/12/2023 16:00	26	32.0	23.4	-8.6
3C	5/12/2023 17:00	26	32.0	23.4	-8.6
3C	5/12/2023 18:00	26	32.0	23.4	-8.6
3C	5/12/2023 19:00	26	32.0	23.4	-8.6
3C	5/12/2023 20:00	26	32.0	28.1	-3.9
3C	5/12/2023 21:00	26	32.0	28.1	-3.9
3C	5/12/2023 22:00	26	32.0	28.1	-3.9
3C	5/12/2023 23:00	26	32.0	28.1	-3.9
3C	5/13/2023 0:00	29	32.0	26.7	-5.4
3C	5/13/2023 1:00	30	32.0	26.7	-5.4
3C	5/13/2023 2:00	30	32.0	26.7	-5.4
3C	5/13/2023 3:00	30	32.0	26.7	-5.4
3C	5/13/2023 4:00	30	32.0	26.7	-5.4
3C	5/13/2023 5:00	30	32.0	26.7	-5.4
3C	5/13/2023 6:00	30	32.0	26.7	-5.4
3C	5/13/2023 7:00	30	32.0	26.7	-5.4
3C	5/13/2023 8:00	30	32.0	20.3	-11.7
3C	5/13/2023 9:00	30	32.0	20.3	-11.7
3C	5/13/2023 10:00	30	32.0	20.3	-11.7
3C	5/13/2023 11:00	30	32.0	20.3	-11.7
3C	5/13/2023 12:00	30	32.0	22.4	-9.6
3C	5/13/2023 13:00	30	32.0	22.4	-9.6
3C	5/13/2023 14:00	30	32.0	22.4	-9.6
3C	5/13/2023 15:00	23	30.0	22.4	-7.6
3C	5/13/2023 16:00	23	30.0	25.8	-4.2
3C	5/13/2023 17:00	23	30.0	25.8	-4.2
3C	5/13/2023 18:00	23	30.0	25.8	-4.2

3C	5/13/2023 19:00	24	29.0	23.4	-5.6
3C	5/13/2023 20:00	24	29.0	18.7	-10.3
3C	5/13/2023 21:00	25	29.0	18.7	-10.3
3C	5/13/2023 22:00	25	29.0	18.7	-10.3
3C	5/13/2023 23:00	25	29.0	18.7	-10.3
3C	5/14/2023 0:00	25	29.0	16.0	-13.0
3C	5/14/2023 1:00	25	29.0	16.0	-13.0
3C	5/14/2023 2:00	25	29.0	16.0	-13.0
3C	5/14/2023 3:00	25	29.0	16.0	-13.0
3C	5/14/2023 4:00	25	29.0	16.0	-13.0
3C	5/14/2023 5:00	25	29.0	16.0	-13.0
3C	5/14/2023 6:00	25	29.0	18.7	-10.3
3C	5/14/2023 7:00	24	28.0	18.7	-9.3
3C	5/14/2023 8:00	24	28.0	18.7	-9.3
3C	5/14/2023 9:00	24	28.0	18.7	-9.3
3C	5/14/2023 10:00	24	28.0	16.0	-12.0
3C	5/14/2023 11:00	24	28.0	16.0	-12.0
3C	5/14/2023 12:00	24	28.0	16.0	-12.0
3C	5/14/2023 13:00	24	28.0	16.0	-12.0
3C	5/14/2023 14:00	24	28.0	16.0	-12.0
3C	5/14/2023 15:00	25	30.0	16.0	-14.0
3C	5/14/2023 16:00	25	30.0	16.0	-14.0
3C	5/14/2023 17:00	25	30.0	16.0	-14.0
3C	5/14/2023 18:00	25	30.0	16.0	-14.0
3C	5/14/2023 19:00	26	29.0	16.0	-13.0
3C	5/14/2023 20:00	26	29.0	2.3	-26.7
3C	5/14/2023 21:00	26	29.0	2.3	-26.7
3C	5/14/2023 22:00	26	29.0	2.3	-26.7
3C	5/14/2023 23:00	28	29.0	2.3	-26.7
3C	5/15/2023 0:00	28	29.0	5.3	-23.7
3C	5/15/2023 1:00	28	29.0	5.3	-23.7
3C	5/15/2023 2:00	28	29.0	5.3	-23.7
3C	5/15/2023 3:00	28	29.0	5.3	-23.7
3C	5/15/2023 4:00	28	29.0	5.3	-23.7
3C	5/15/2023 5:00	28	29.0	5.3	-23.7
3C	5/15/2023 6:00	28	29.0	5.3	-23.7
3C	5/15/2023 7:00	28	29.0	5.3	-23.7
3C	5/15/2023 8:00	28	29.0	20.3	-8.7
3C	5/15/2023 9:00	28	29.0	20.3	-8.7
3C	5/15/2023 10:00	28	29.0	20.3	-8.7
3C	5/15/2023 11:00	28	29.0	20.3	-8.7
3C	5/15/2023 12:00	28	29.0	22.4	-6.6
3C	5/15/2023 13:00	28	29.0	22.4	-6.6
3C	5/15/2023 14:00	39	33.0	22.4	-10.6
3C	5/15/2023 15:00	39	33.0	22.4	-10.6
3C	5/15/2023 16:00	39	33.0	20.5	-12.5
3C	5/15/2023 17:00	39	33.0	20.5	-12.5

3C	5/15/2023 18:00	39	33.0	20.5	-12.5
3C	5/15/2023 19:00	31	34.0	20.5	-13.5
3C	5/15/2023 20:00	31	34.0	16.4	-17.6
3C	5/15/2023 21:00	31	34.0	16.4	-17.6
3C	5/15/2023 22:00	31	34.0	16.4	-17.6
3C	5/15/2023 23:00	31	34.0	16.4	-17.6
3C	5/16/2023 0:00	31	34.0	13.3	-20.7
3C	5/16/2023 1:00	31	34.0	13.3	-20.7
3C	5/16/2023 2:00	31	34.0	13.3	-20.7
3C	5/16/2023 3:00	31	34.0	13.3	-20.7
3C	5/16/2023 4:00	31	34.0	13.3	-20.7
3C	5/16/2023 5:00	31	34.0	13.3	-20.7
3C	5/16/2023 6:00	30	30.0	13.3	-16.7
3C	5/16/2023 7:00	30	30.0	13.3	-16.7
3C	5/16/2023 8:00	30	30.0	20.3	-9.7
3C	5/16/2023 9:00	30	30.0	20.3	-9.7
3C	5/16/2023 10:00	30	30.0	20.3	-9.7
3C	5/16/2023 11:00	30	30.0	20.3	-9.7
3C	5/16/2023 12:00	30	30.0	22.9	-7.1
3C	5/16/2023 13:00	30	30.0	22.9	-7.1
3C	5/16/2023 14:00	30	30.0	22.9	-7.1
3C	5/16/2023 15:00	30	30.0	22.9	-7.1
3C	5/16/2023 16:00	30	30.0	26.4	-3.7
3C	5/16/2023 17:00	30	30.0	26.4	-3.7
3C	5/16/2023 18:00	30	30.0	26.4	-3.7
3C	5/16/2023 19:00	30	29.0	26.4	-2.7
3C	5/16/2023 20:00	30	29.0	20.5	-8.5
3C	5/16/2023 21:00	30	29.0	20.5	-8.5
3C	5/16/2023 22:00	30	29.0	20.5	-8.5
3C	5/16/2023 23:00	30	29.0	20.5	-8.5
3C	5/17/2023 0:00	30	29.0	20.0	-9.0
3C	5/17/2023 1:00	30	29.0	20.0	-9.0
3C	5/17/2023 2:00	30	29.0	20.0	-9.0
3C	5/17/2023 3:00	30	29.0	20.0	-9.0
3C	5/17/2023 4:00	30	29.0	20.0	-9.0
3C	5/17/2023 5:00	30	30.0	20.0	-10.0
3C	5/17/2023 6:00	30	30.0	20.0	-10.0
3C	5/17/2023 7:00	30	30.0	20.0	-10.0
3C	5/17/2023 8:00	30	30.0	12.7	-17.3
3C	5/17/2023 9:00	30	30.0	12.7	-17.3
3C	5/17/2023 10:00	30	30.0	12.7	-17.3
3C	5/17/2023 11:00	30	30.0	12.7	-17.3
3C	5/17/2023 12:00	30	30.0	17.8	-12.2
3C	5/17/2023 13:00	30	30.0	17.8	-12.2
3C	5/17/2023 14:00	30	30.0	17.8	-12.2
3C	5/17/2023 15:00	30	30.0	17.8	-12.2
3C	5/17/2023 16:00	30	30.0	14.6	-15.4

3C	5/17/2023 17:00	30	30.0	14.6	-15.4
3C	5/17/2023 18:00	30	30.0	14.6	-15.4
3C	5/17/2023 19:00	30	30.0	14.6	-15.4
3C	5/17/2023 20:00	30	30.0	26.4	-3.7
3C	5/17/2023 21:00	30	30.0	26.4	-3.7
3C	5/17/2023 22:00	30	30.0	26.4	-3.7
3C	5/17/2023 23:00	30	30.0	26.4	-3.7
3C	5/18/2023 0:00	30	30.0	26.7	-3.4
3C	5/18/2023 1:00	30	30.0	26.7	-3.4
3C	5/18/2023 2:00	30	30.0	26.7	-3.4
3C	5/18/2023 3:00	30	30.0	26.7	-3.4
3C	5/18/2023 4:00	30	30.0	26.7	-3.4
3C	5/18/2023 5:00	30	30.0	26.7	-3.4
3C	5/18/2023 6:00	30	30.0	26.7	-3.4
3C	5/18/2023 7:00	34	34.0	26.7	-7.4
3C	5/18/2023 8:00	34	34.0	22.9	-11.1
3C	5/18/2023 9:00	34	34.0	22.9	-11.1
3C	5/18/2023 10:00	34	34.0	22.9	-11.1
3C	5/18/2023 11:00	34	34.0	22.9	-11.1
3C	5/18/2023 12:00	34	34.0	28.0	-6.0
3C	5/18/2023 13:00	34	34.0	28.0	-6.0
3C	5/18/2023 14:00	34	34.0	28.0	-6.0
3C	5/18/2023 15:00	34	34.0	28.0	-6.0
3C	5/18/2023 16:00	34	34.0	21.1	-12.9
3C	5/18/2023 17:00	34	34.0	21.1	-12.9
3C	5/18/2023 18:00	34	34.0	21.1	-12.9
3C	5/18/2023 19:00	27	30.0	21.1	-8.9
3C	5/18/2023 20:00	27	30.0	18.7	-11.3
3C	5/18/2023 21:00	27	30.0	18.7	-11.3
3C	5/18/2023 22:00	27	30.0	18.7	-11.3
3C	5/18/2023 23:00	27	30.0	18.7	-11.3
3C	5/19/2023 0:00	27	30.0	13.3	-16.7
3C	5/19/2023 1:00	27	30.0	13.3	-16.7
3C	5/19/2023 2:00	27	30.0	13.3	-16.7
3C	5/19/2023 3:00	27	30.0	13.3	-16.7
3C	5/19/2023 4:00	27	30.0	13.3	-16.7
3C	5/19/2023 5:00	27	30.0	13.3	-16.7
3C	5/19/2023 6:00	34	30.0	13.3	-16.7
3C	5/19/2023 7:00	34	30.0	13.3	-16.7
3C	5/19/2023 8:00	34	30.0	20.3	-9.7
3C	5/19/2023 9:00	34	30.0	20.3	-9.7
3C	5/19/2023 10:00	34	30.0	20.3	-9.7
3C	5/19/2023 11:00	27	30.0	20.3	-9.7
3C	5/19/2023 12:00	27	30.0	20.3	-9.7
3C	5/19/2023 13:00	27	30.0	20.3	-9.7
3C	5/19/2023 14:00	27	30.0	20.3	-9.7
3C	5/19/2023 15:00	27	30.0	20.3	-9.7

3C	5/19/2023 16:00	27	30.0	23.4	-6.6
3C	5/19/2023 17:00	27	30.0	23.4	-6.6
3C	5/19/2023 18:00	27	30.0	23.4	-6.6
3C	5/19/2023 19:00	25	25.0	23.4	-1.6
3C	5/19/2023 20:00	25	25.0	11.7	-13.3
3C	5/19/2023 21:00	25	25.0	11.7	-13.3
3C	5/19/2023 22:00	25	25.0	11.7	-13.3
3C	5/19/2023 23:00	25	25.0	11.7	-13.3
3C	5/20/2023 0:00	25	25.0	13.3	-11.7
3C	5/20/2023 1:00	25	25.0	13.3	-11.7
3C	5/20/2023 2:00	25	25.0	13.3	-11.7
3C	5/20/2023 3:00	25	25.0	13.3	-11.7
3C	5/20/2023 4:00	25	25.0	13.3	-11.7
3C	5/20/2023 5:00	25	25.0	13.3	-11.7
3C	5/20/2023 6:00	25	25.0	13.3	-11.7
3C	5/20/2023 7:00	25	25.0	13.3	-11.7
3C	5/20/2023 8:00	25	25.0	18.3	-6.7
3C	5/20/2023 9:00	25	25.0	18.3	-6.7
3C	5/20/2023 10:00	25	25.0	18.3	-6.7
3C	5/20/2023 11:00	25	25.0	18.3	-6.7
3C	5/20/2023 12:00	25	25.0	20.3	-4.7
3C	5/20/2023 13:00	25	25.0	20.3	-4.7
3C	5/20/2023 14:00	25	25.0	20.3	-4.7
3C	5/20/2023 15:00	22	30.0	20.3	-9.7
3C	5/20/2023 16:00	22	30.0	23.4	-6.6
3C	5/20/2023 17:00	22	30.0	23.4	-6.6
3C	5/20/2023 18:00	22	30.0	23.4	-6.6
3C	5/20/2023 19:00	24	30.0	23.4	-6.6
3C	5/20/2023 20:00	24	30.0	18.7	-11.3
3C	5/20/2023 21:00	24	30.0	18.7	-11.3
3C	5/20/2023 22:00	24	30.0	18.7	-11.3
3C	5/20/2023 23:00	24	30.0	18.7	-11.3
3C	5/21/2023 0:00	24	30.0	20.0	-10.0
3C	5/21/2023 1:00	24	30.0	20.0	-10.0
3C	5/21/2023 2:00	24	30.0	20.0	-10.0
3C	5/21/2023 3:00	24	30.0	20.0	-10.0
3C	5/21/2023 4:00	24	30.0	20.0	-10.0
3C	5/21/2023 5:00	24	30.0	20.0	-10.0
3C	5/21/2023 6:00	24	30.0	20.0	-10.0
3C	5/21/2023 7:00	29	26.0	20.0	-6.0
3C	5/21/2023 8:00	29	26.0	18.3	-7.7
3C	5/21/2023 9:00	29	26.0	18.3	-7.7
3C	5/21/2023 10:00	29	26.0	18.3	-7.7
3C	5/21/2023 11:00	29	26.0	18.3	-7.7
3C	5/21/2023 12:00	23	30.0	18.3	-11.7
3C	5/21/2023 13:00	23	30.0	18.3	-11.7
3C	5/21/2023 14:00	23	30.0	18.3	-11.7

3C	5/21/2023 15:00	23	30.0	20.3	-9.7
3C	5/21/2023 16:00	23	30.0	23.4	-6.6
3C	5/21/2023 17:00	23	30.0	23.4	-6.6
3C	5/21/2023 18:00	23	30.0	23.4	-6.6
3C	5/21/2023 19:00	23	30.0	23.4	-6.6
3C	5/21/2023 20:00	23	30.0	21.1	-8.9
3C	5/21/2023 21:00	23	30.0	21.1	-8.9
3C	5/21/2023 22:00	23	30.0	21.1	-8.9
3C	5/21/2023 23:00	23	30.0	21.1	-8.9
3C	5/22/2023 0:00	23	30.0	21.3	-8.7
3C	5/22/2023 1:00	23	30.0	21.3	-8.7
3C	5/22/2023 2:00	23	30.0	21.3	-8.7
3C	5/22/2023 3:00	23	30.0	21.3	-8.7
3C	5/22/2023 4:00	23	30.0	21.3	-8.7
3C	5/22/2023 5:00	23	30.0	21.3	-8.7
3C	5/22/2023 6:00	23	30.0	21.3	-8.7
3C	5/22/2023 7:00	23	30.0	21.3	-8.7
3C	5/22/2023 8:00	23	30.0	14.2	-15.8
3C	5/22/2023 9:00	23	30.0	14.2	-15.8
3C	5/22/2023 10:00	23	30.0	14.2	-15.8
3C	5/22/2023 11:00	23	30.0	14.2	-15.8
3C	5/22/2023 12:00	23	30.0	12.7	-17.3
3C	5/22/2023 13:00	23	30.0	12.7	-17.3
3C	5/22/2023 14:00	21	29.0	15.3	-13.7
3C	5/22/2023 15:00	21	29.0	15.3	-13.7
3C	5/22/2023 16:00	21	29.0	17.6	-11.4
3C	5/22/2023 17:00	21	29.0	17.6	-11.4
3C	5/22/2023 18:00	21	29.0	17.6	-11.4
3C	5/22/2023 19:00	22	22.0	17.6	-4.4
3C	5/22/2023 20:00	22	22.0	14.6	-7.4
3C	5/22/2023 21:00	22	22.0	14.6	-7.4
3C	5/22/2023 22:00	22	22.0	14.1	-8.0
3C	5/22/2023 23:00	22	22.0	14.1	-8.0
3C	5/23/2023 0:00	22	22.0	16.0	-6.0
3C	5/23/2023 1:00	22	22.0	16.0	-6.0
3C	5/23/2023 2:00	22	22.0	16.0	-6.0
3C	5/23/2023 3:00	22	22.0	16.0	-6.0
3C	5/23/2023 4:00	22	22.0	16.0	-6.0
3C	5/23/2023 5:00	22	22.0	16.0	-6.0
3C	5/23/2023 6:00	22	22.0	16.0	-6.0
3C	5/23/2023 7:00	22	22.0	16.0	-6.0
3C	5/23/2023 8:00	22	22.0	16.3	-5.7
3C	5/23/2023 9:00	22	22.0	16.3	-5.7
3C	5/23/2023 10:00	22	22.0	16.3	-5.7
3C	5/23/2023 11:00	21	30.0	20.3	-9.7
3C	5/23/2023 12:00	21	30.0	20.3	-9.7
3C	5/23/2023 13:00	21	30.0	20.3	-9.7

3C	5/23/2023 14:00	21	30.0	20.3	-9.7
3C	5/23/2023 15:00	21	30.0	20.3	-9.7
3C	5/23/2023 16:00	21	30.0	20.5	-9.5
3C	5/23/2023 17:00	21	30.0	20.5	-9.5
3C	5/23/2023 18:00	21	30.0	20.5	-9.5
3C	5/23/2023 19:00	21	30.0	20.5	-9.5
3C	5/23/2023 20:00	21	30.0	17.6	-12.4
3C	5/23/2023 21:00	21	30.0	17.6	-12.4
3C	5/23/2023 22:00	21	30.0	17.6	-12.4
3C	5/23/2023 23:00	21	30.0	17.6	-12.4
3C	5/24/2023 0:00	24	25.0	16.7	-8.3
3C	5/24/2023 1:00	24	25.0	16.7	-8.3
3C	5/24/2023 2:00	24	25.0	16.7	-8.3
3C	5/24/2023 3:00	24	25.0	16.7	-8.3
3C	5/24/2023 4:00	24	25.0	16.7	-8.3
3C	5/24/2023 5:00	24	25.0	16.7	-8.3
3C	5/24/2023 6:00	24	25.0	16.7	-8.3
3C	5/24/2023 7:00	24	29.0	16.0	-13.0
3C	5/24/2023 8:00	24	29.0	16.3	-12.7
3C	5/24/2023 9:00	24	29.0	16.3	-12.7
3C	5/24/2023 10:00	24	29.0	16.3	-12.7
3C	5/24/2023 11:00	24	29.0	16.3	-12.7
3C	5/24/2023 12:00	24	29.0	20.3	-8.7
3C	5/24/2023 13:00	24	29.0	20.3	-8.7
3C	5/24/2023 14:00	24	29.0	20.3	-8.7
3C	5/24/2023 15:00	20	25.0	22.4	-2.6
3C	5/24/2023 16:00	20	25.0	17.6	-7.4
3C	5/24/2023 17:00	20	25.0	17.6	-7.4
3C	5/24/2023 18:00	20	25.0	17.6	-7.4
3C	5/24/2023 19:00	20	25.0	16.1	-8.9
3C	5/24/2023 20:00	20	25.0	16.4	-8.6
3C	5/24/2023 21:00	20	25.0	16.4	-8.6
3C	5/24/2023 22:00	20	25.0	16.4	-8.6
3C	5/24/2023 23:00	20	25.0	16.4	-8.6
3C	5/25/2023 0:00	20	25.0	16.0	-9.0
3C	5/25/2023 1:00	20	25.0	16.0	-9.0
3C	5/25/2023 2:00	20	25.0	16.0	-9.0
3C	5/25/2023 3:00	20	25.0	16.0	-9.0
3C	5/25/2023 4:00	20	25.0	16.0	-9.0
3C	5/25/2023 5:00	20	25.0	16.0	-9.0
3C	5/25/2023 6:00	20	25.0	16.0	-9.0
3C	5/25/2023 7:00	20	25.0	16.0	-9.0
3C	5/25/2023 8:00	21	30.0	17.8	-12.2
3C	5/25/2023 9:00	21	30.0	17.8	-12.2
3C	5/25/2023 10:00	21	30.0	17.8	-12.2
3C	5/25/2023 11:00	21	30.0	17.8	-12.2
3C	5/25/2023 12:00	21	30.0	22.9	-7.1

3C	5/25/2023 13:00	21	30.0	22.9	-7.1
3C	5/25/2023 14:00	21	30.0	22.9	-7.1
3C	5/25/2023 15:00	21	30.0	22.9	-7.1
3C	5/25/2023 16:00	21	30.0	26.4	-3.7
3C	5/25/2023 17:00	21	30.0	26.4	-3.7
3C	5/25/2023 18:00	21	30.0	25.8	-4.2
3C	5/25/2023 19:00	21	30.0	25.8	-4.2
3C	5/25/2023 20:00	21	30.0	21.1	-8.9
3C	5/25/2023 21:00	21	30.0	21.1	-8.9
3C	5/25/2023 22:00	21	30.0	21.1	-8.9
3C	5/25/2023 23:00	21	30.0	21.1	-8.9
4A	1/1/2023 0:00	12	11.0	13.8	2.8
4A	1/1/2023 1:00	12	11.0	13.8	2.8
4A	1/1/2023 2:00	12	11.0	13.8	2.8
4A	1/1/2023 3:00	12	11.0	13.8	2.8
4A	1/1/2023 4:00	12	11.0	13.8	2.8
4A	1/1/2023 5:00	12	11.0	13.8	2.8
4A	1/1/2023 6:00	12	11.0	13.8	2.8
4A	1/1/2023 7:00	12	11.0	13.8	2.8
4A	1/1/2023 8:00	12	11.0	15.5	4.5
4A	1/1/2023 9:00	12	11.0	15.5	4.5
4A	1/1/2023 10:00	12	11.0	15.5	4.5
4A	1/1/2023 11:00	12	11.0	15.5	4.5
4A	1/1/2023 12:00	12	11.0	18.0	7.0
4A	1/1/2023 13:00	12	11.0	18.0	7.0
4A	1/1/2023 14:00	12	11.0	18.0	7.0
4A	1/1/2023 15:00	12	11.0	15.5	4.5
4A	1/1/2023 16:00	12	11.0	17.2	6.2
4A	1/1/2023 17:00	11	13.0	17.2	4.2
4A	1/1/2023 18:00	11	13.0	17.2	4.2
4A	1/1/2023 19:00	11	13.0	17.2	4.2
4A	1/1/2023 20:00	11	13.0	16.6	3.6
4A	1/1/2023 21:00	11	13.0	16.6	3.6
4A	1/1/2023 22:00	11	13.0	16.6	3.6
4A	1/1/2023 23:00	11	13.0	16.6	3.6
4A	1/2/2023 0:00	11	13.0	13.8	0.8
4A	1/2/2023 1:00	11	13.0	13.8	0.8
4A	1/2/2023 2:00	11	13.0	13.8	0.8
4A	1/2/2023 3:00	11	13.0	13.8	0.8
4A	1/2/2023 4:00	11	13.0	13.8	0.8
4A	1/2/2023 5:00	11	13.0	13.8	0.8
4A	1/2/2023 6:00	11	13.0	13.8	0.8
4A	1/2/2023 7:00	11	13.0	15.2	2.2
4A	1/2/2023 8:00	11	13.0	15.5	2.5
4A	1/2/2023 9:00	11	13.0	15.5	2.5
4A	1/2/2023 10:00	11	13.0	15.5	2.5
4A	1/2/2023 11:00	11	13.0	15.5	2.5

4A	1/2/2023 12:00	11	13.0	12.9	-0.1
4A	1/2/2023 13:00	11	13.0	12.9	-0.1
4A	1/2/2023 14:00	11	13.0	12.9	-0.1
4A	1/2/2023 15:00	11	13.0	12.9	-0.1
4A	1/2/2023 16:00	11	13.0	16.6	3.6
4A	1/2/2023 17:00	11	13.0	16.6	3.6
4A	1/2/2023 18:00	11	13.0	16.6	3.6
4A	1/2/2023 19:00	8	13.0	16.6	3.6
4A	1/2/2023 20:00	10	13.0	16.6	3.6
4A	1/2/2023 21:00	10	13.0	16.6	3.6
4A	1/2/2023 22:00	10	13.0	16.6	3.6
4A	1/2/2023 23:00	10	13.0	16.6	3.6
4A	1/3/2023 0:00	10	13.0	18.0	5.0
4A	1/3/2023 1:00	10	13.0	18.0	5.0
4A	1/3/2023 2:00	10	13.0	18.0	5.0
4A	1/3/2023 3:00	10	13.0	18.0	5.0
4A	1/3/2023 4:00	11	13.0	18.0	5.0
4A	1/3/2023 5:00	11	13.0	18.0	5.0
4A	1/3/2023 6:00	11	13.0	18.0	5.0
4A	1/3/2023 7:00	11	13.0	18.0	5.0
4A	1/3/2023 8:00	11	13.0	12.9	-0.1
4A	1/3/2023 9:00	11	13.0	12.9	-0.1
4A	1/3/2023 10:00	11	13.0	12.9	-0.1
4A	1/3/2023 11:00	11	13.0	12.9	-0.1
4A	1/3/2023 12:00	11	13.0	10.4	-2.7
4A	1/3/2023 13:00	11	13.0	10.4	-2.7
4A	1/3/2023 14:00	11	13.0	10.4	-2.7
4A	1/3/2023 15:00	11	13.0	10.4	-2.7
4A	1/3/2023 16:00	11	13.0	13.8	0.8
4A	1/3/2023 17:00	11	13.0	13.8	0.8
4A	1/3/2023 18:00	11	13.0	13.8	0.8
4A	1/3/2023 19:00	11	13.0	13.8	0.8
4A	1/3/2023 20:00	11	13.0	17.2	4.2
4A	1/3/2023 21:00	11	13.0	17.2	4.2
4A	1/3/2023 22:00	11	13.0	17.2	4.2
4A	1/3/2023 23:00	11	13.0	17.2	4.2
4A	1/4/2023 0:00	11	13.0	18.0	5.0
4A	1/4/2023 1:00	11	13.0	18.0	5.0
4A	1/4/2023 2:00	11	13.0	18.0	5.0
4A	1/4/2023 3:00	11	13.0	18.0	5.0
4A	1/4/2023 4:00	11	13.0	18.0	5.0
4A	1/4/2023 5:00	11	13.0	18.0	5.0
4A	1/4/2023 6:00	11	13.0	18.0	5.0
4A	1/4/2023 7:00	11	13.0	18.0	5.0
4A	1/4/2023 8:00	11	13.0	18.0	5.0
4A	1/4/2023 9:00	11	13.0	18.0	5.0
4A	1/4/2023 10:00	11	13.0	18.0	5.0

4A	1/4/2023 11:00	11	13.0	18.0	5.0
4A	1/4/2023 12:00	11	13.0	15.5	2.5
4A	1/4/2023 13:00	11	13.0	15.5	2.5
4A	1/4/2023 14:00	11	13.0	15.5	2.5
4A	1/4/2023 15:00	11	13.0	15.5	2.5
4A	1/4/2023 16:00	11	13.0	17.2	4.2
4A	1/4/2023 17:00	11	13.0	17.2	4.2
4A	1/4/2023 18:00	11	13.0	17.2	4.2
4A	1/4/2023 19:00	11	13.0	17.2	4.2
4A	1/4/2023 20:00	11	13.0	6.9	-6.1
4A	1/4/2023 21:00	11	13.0	6.9	-6.1
4A	1/4/2023 22:00	11	13.0	6.9	-6.1
4A	1/4/2023 23:00	11	13.0	6.9	-6.1
4A	1/5/2023 0:00	11	13.0	6.9	-6.1
4A	1/5/2023 1:00	11	13.0	6.9	-6.1
4A	1/5/2023 2:00	11	13.0	6.9	-6.1
4A	1/5/2023 3:00	11	13.0	6.9	-6.1
4A	1/5/2023 4:00	11	13.0	6.9	-6.1
4A	1/5/2023 5:00	11	13.0	6.9	-6.1
4A	1/5/2023 6:00	11	13.0	6.9	-6.1
4A	1/5/2023 7:00	11	13.0	6.9	-6.1
4A	1/5/2023 8:00	11	13.0	15.5	2.5
4A	1/5/2023 9:00	11	13.0	15.5	2.5
4A	1/5/2023 10:00	11	13.0	15.5	2.5
4A	1/5/2023 11:00	11	13.0	15.5	2.5
4A	1/5/2023 12:00	11	13.0	15.5	2.5
4A	1/5/2023 13:00	11	13.0	15.5	2.5
4A	1/5/2023 14:00	11	13.0	15.5	2.5
4A	1/5/2023 15:00	11	13.0	15.5	2.5
4A	1/5/2023 16:00	11	13.0	18.0	5.0
4A	1/5/2023 17:00	11	13.0	18.0	5.0
4A	1/5/2023 18:00	11	13.0	18.0	5.0
4A	1/5/2023 19:00	11	13.0	18.0	5.0
4A	1/5/2023 20:00	11	13.0	8.6	-4.4
4A	1/5/2023 21:00	11	13.0	10.4	-2.7
4A	1/5/2023 22:00	11	13.0	10.4	-2.7
4A	1/5/2023 23:00	11	13.0	10.4	-2.7
4A	1/6/2023 0:00	11	13.0	10.4	-2.7
4A	1/6/2023 1:00	11	13.0	10.4	-2.7
4A	1/6/2023 2:00	11	13.0	10.4	-2.7
4A	1/6/2023 3:00	11	13.0	10.4	-2.7
4A	1/6/2023 4:00	11	13.0	10.4	-2.7
4A	1/6/2023 5:00	11	13.0	10.4	-2.7
4A	1/6/2023 6:00	11	13.0	10.4	-2.7
4A	1/6/2023 7:00	11	13.0	10.4	-2.7
4A	1/6/2023 8:00	13	13.0	8.3	-4.7
4A	1/6/2023 9:00	13	13.0	8.3	-4.7

4A	1/6/2023 10:00	13	13.0	8.3	-4.7
4A	1/6/2023 11:00	13	13.0	8.3	-4.7
4A	1/6/2023 12:00	13	13.0	11.4	-1.6
4A	1/6/2023 13:00	13	13.0	12.4	-0.6
4A	1/6/2023 14:00	13	13.0	12.4	-0.6
4A	1/6/2023 15:00	13	13.0	12.4	-0.6
4A	1/6/2023 16:00	13	13.0	16.6	3.6
4A	1/6/2023 17:00	13	13.0	16.6	3.6
4A	1/6/2023 18:00	13	13.0	16.6	3.6
4A	1/6/2023 19:00	13	13.0	16.6	3.6
4A	1/6/2023 20:00	13	13.0	13.8	0.8
4A	1/6/2023 21:00	13	13.0	13.8	0.8
4A	1/6/2023 22:00	13	13.0	13.8	0.8
4A	1/6/2023 23:00	13	13.0	13.8	0.8
4A	1/7/2023 0:00	13	13.0	10.4	-2.7
4A	1/7/2023 1:00	13	13.0	10.4	-2.7
4A	1/7/2023 2:00	13	13.0	10.4	-2.7
4A	1/7/2023 3:00	13	13.0	10.4	-2.7
4A	1/7/2023 4:00	13	13.0	10.4	-2.7
4A	1/7/2023 5:00	13	13.0	10.4	-2.7
4A	1/7/2023 6:00	13	13.0	10.4	-2.7
4A	1/7/2023 7:00	13	13.0	10.4	-2.7
4A	1/7/2023 8:00	13	13.0	14.5	1.5
4A	1/7/2023 9:00	13	13.0	14.5	1.5
4A	1/7/2023 10:00	13	13.0	14.5	1.5
4A	1/7/2023 11:00	13	13.0	14.5	1.5
4A	1/7/2023 12:00	13	13.0	15.5	2.5
4A	1/7/2023 13:00	13	13.0	15.5	2.5
4A	1/7/2023 14:00	13	13.0	15.5	2.5
4A	1/7/2023 15:00	13	13.0	15.5	2.5
4A	1/7/2023 16:00	13	13.0	18.0	5.0
4A	1/7/2023 17:00	13	13.0	18.0	5.0
4A	1/7/2023 18:00	13	13.0	18.0	5.0
4A	1/7/2023 19:00	13	13.0	18.0	5.0
4A	1/7/2023 20:00	12	13.0	11.0	-2.0
4A	1/7/2023 21:00	12	13.0	11.0	-2.0
4A	1/7/2023 22:00	12	13.0	11.0	-2.0
4A	1/7/2023 23:00	12	13.0	11.0	-2.0
4A	1/8/2023 0:00	14	13.0	13.8	0.8
4A	1/8/2023 1:00	14	13.0	13.8	0.8
4A	1/8/2023 2:00	14	13.0	13.8	0.8
4A	1/8/2023 3:00	14	13.0	13.8	0.8
4A	1/8/2023 4:00	14	13.0	13.8	0.8
4A	1/8/2023 5:00	14	13.0	13.8	0.8
4A	1/8/2023 6:00	14	13.0	13.8	0.8
4A	1/8/2023 7:00	14	13.0	13.8	0.8
4A	1/8/2023 8:00	14	13.0	18.0	5.0

4A	1/8/2023 9:00	14	13.0	18.0	5.0
4A	1/8/2023 10:00	14	13.0	18.0	5.0
4A	1/8/2023 11:00	14	13.0	18.0	5.0
4A	1/8/2023 12:00	14	13.0	15.5	2.5
4A	1/8/2023 13:00	14	13.0	15.5	2.5
4A	1/8/2023 14:00	14	13.0	15.5	2.5
4A	1/8/2023 15:00	14	13.0	15.5	2.5
4A	1/8/2023 16:00	14	13.0	18.0	5.0
4A	1/8/2023 17:00	14	13.0	18.0	5.0
4A	1/8/2023 18:00	14	13.0	18.0	5.0
4A	1/8/2023 19:00	14	13.0	18.0	5.0
4A	1/8/2023 20:00	14	13.0	18.0	5.0
4A	1/8/2023 21:00	14	13.0	18.0	5.0
4A	1/8/2023 22:00	14	13.0	18.0	5.0
4A	1/8/2023 23:00	14	13.0	18.0	5.0
4A	1/9/2023 0:00	14	13.0	18.0	5.0
4A	1/9/2023 1:00	14	13.0	18.0	5.0
4A	1/9/2023 2:00	14	13.0	18.0	5.0
4A	1/9/2023 3:00	14	13.0	18.0	5.0
4A	1/9/2023 4:00	14	13.0	18.0	5.0
4A	1/9/2023 5:00	14	13.0	18.0	5.0
4A	1/9/2023 6:00	14	13.0	18.0	5.0
4A	1/9/2023 7:00	14	13.0	18.0	5.0
4A	1/9/2023 8:00	14	16.0	18.0	2.0
4A	1/9/2023 9:00	14	16.0	18.0	2.0
4A	1/9/2023 10:00	14	16.0	18.0	2.0
4A	1/9/2023 11:00	14	16.0	18.0	2.0
4A	1/9/2023 12:00	14	16.0	18.0	2.0
4A	1/9/2023 13:00	14	16.0	18.0	2.0
4A	1/9/2023 14:00	14	16.0	18.0	2.0
4A	1/9/2023 15:00	14	16.0	18.0	2.0
4A	1/9/2023 16:00	14	16.0	17.2	1.2
4A	1/9/2023 17:00	14	16.0	17.2	1.2
4A	1/9/2023 18:00	14	16.0	17.2	1.2
4A	1/9/2023 19:00	14	16.0	17.2	1.2
4A	1/9/2023 20:00	14	16.0	17.2	1.2
4A	1/9/2023 21:00	14	16.0	17.2	1.2
4A	1/9/2023 22:00	14	16.0	17.2	1.2
4A	1/9/2023 23:00	14	16.0	17.2	1.2
4A	1/10/2023 0:00	14	16.0	16.6	0.6
4A	1/10/2023 1:00	14	16.0	16.6	0.6
4A	1/10/2023 2:00	14	16.0	16.6	0.6
4A	1/10/2023 3:00	14	16.0	16.6	0.6
4A	1/10/2023 4:00	14	16.0	16.6	0.6
4A	1/10/2023 5:00	14	16.0	16.6	0.6
4A	1/10/2023 6:00	14	16.0	16.6	0.6
4A	1/10/2023 7:00	14	16.0	17.2	1.2

4A	1/10/2023 8:00	16	16.0	10.4	-5.7
4A	1/10/2023 9:00	16	16.0	10.4	-5.7
4A	1/10/2023 10:00	16	16.0	10.4	-5.7
4A	1/10/2023 11:00	16	16.0	10.4	-5.7
4A	1/10/2023 12:00	16	16.0	12.4	-3.6
4A	1/10/2023 13:00	16	16.0	12.4	-3.6
4A	1/10/2023 14:00	16	16.0	12.4	-3.6
4A	1/10/2023 15:00	16	16.0	11.4	-4.6
4A	1/10/2023 16:00	16	16.0	13.8	-2.2
4A	1/10/2023 17:00	16	16.0	13.8	-2.2
4A	1/10/2023 18:00	16	16.0	13.8	-2.2
4A	1/10/2023 19:00	16	16.0	13.8	-2.2
4A	1/10/2023 20:00	16	16.0	13.8	-2.2
4A	1/10/2023 21:00	16	16.0	13.8	-2.2
4A	1/10/2023 22:00	11	11.0	13.8	2.8
4A	1/10/2023 23:00	11	11.0	13.8	2.8
4A	1/11/2023 0:00	11	11.0	13.8	2.8
4A	1/11/2023 1:00	11	11.0	13.8	2.8
4A	1/11/2023 2:00	11	11.0	13.8	2.8
4A	1/11/2023 3:00	11	11.0	13.8	2.8
4A	1/11/2023 4:00	11	11.0	13.8	2.8
4A	1/11/2023 5:00	11	11.0	13.8	2.8
4A	1/11/2023 6:00	11	11.0	13.8	2.8
4A	1/11/2023 7:00	11	11.0	13.8	2.8
4A	1/11/2023 8:00	11	13.0	12.4	-0.6
4A	1/11/2023 9:00	11	13.0	12.4	-0.6
4A	1/11/2023 10:00	11	13.0	12.4	-0.6
4A	1/11/2023 11:00	11	13.0	12.4	-0.6
4A	1/11/2023 12:00	11	13.0	12.4	-0.6
4A	1/11/2023 13:00	11	13.0	12.4	-0.6
4A	1/11/2023 14:00	11	13.0	12.4	-0.6
4A	1/11/2023 15:00	11	13.0	12.4	-0.6
4A	1/11/2023 16:00	11	13.0	16.6	3.6
4A	1/11/2023 17:00	11	13.0	16.6	3.6
4A	1/11/2023 18:00	11	13.0	16.6	3.6
4A	1/11/2023 19:00	11	13.0	16.6	3.6
4A	1/11/2023 20:00	11	13.0	11.7	-1.3
4A	1/11/2023 21:00	11	13.0	13.8	0.8
4A	1/11/2023 22:00	11	13.0	13.8	0.8
4A	1/11/2023 23:00	11	13.0	13.8	0.8
4A	1/12/2023 0:00	11	13.0	13.8	0.8
4A	1/12/2023 1:00	13	13.0	13.8	0.8
4A	1/12/2023 2:00	13	13.0	13.8	0.8
4A	1/12/2023 3:00	13	13.0	13.8	0.8
4A	1/12/2023 4:00	13	13.0	13.8	0.8
4A	1/12/2023 5:00	13	13.0	13.8	0.8
4A	1/12/2023 6:00	13	13.0	13.8	0.8

4A	1/12/2023 7:00	13	13.0	13.8	0.8
4A	1/12/2023 8:00	13	13.0	7.8	-5.2
4A	1/12/2023 9:00	13	13.0	7.8	-5.2
4A	1/12/2023 10:00	13	13.0	7.8	-5.2
4A	1/12/2023 11:00	13	13.0	7.8	-5.2
4A	1/12/2023 12:00	13	13.0	7.8	-5.2
4A	1/12/2023 13:00	13	13.0	7.8	-5.2
4A	1/12/2023 14:00	13	13.0	7.8	-5.2
4A	1/12/2023 15:00	13	13.0	7.8	-5.2
4A	1/12/2023 16:00	13	13.0	10.4	-2.7
4A	1/12/2023 17:00	13	13.0	10.4	-2.7
4A	1/12/2023 18:00	13	13.0	10.4	-2.7
4A	1/12/2023 19:00	13	13.0	10.4	-2.7
4A	1/12/2023 20:00	13	13.0	13.8	0.8
4A	1/12/2023 21:00	13	13.0	13.8	0.8
4A	1/12/2023 22:00	13	13.0	13.8	0.8
4A	1/12/2023 23:00	13	13.0	13.8	0.8
4A	1/13/2023 0:00	12	13.0	13.8	0.8
4A	1/13/2023 1:00	12	13.0	13.8	0.8
4A	1/13/2023 2:00	12	13.0	13.8	0.8
4A	1/13/2023 3:00	12	13.0	13.8	0.8
4A	1/13/2023 4:00	12	13.0	13.8	0.8
4A	1/13/2023 5:00	12	13.0	13.8	0.8
4A	1/13/2023 6:00	12	13.0	13.8	0.8
4A	1/13/2023 7:00	12	13.0	13.8	0.8
4A	1/13/2023 8:00	12	13.0	7.8	-5.2
4A	1/13/2023 9:00	12	13.0	7.8	-5.2
4A	1/13/2023 10:00	12	13.0	7.8	-5.2
4A	1/13/2023 11:00	12	13.0	7.8	-5.2
4A	1/13/2023 12:00	12	13.0	12.9	-0.1
4A	1/13/2023 13:00	12	13.0	12.9	-0.1
4A	1/13/2023 14:00	12	13.0	12.9	-0.1
4A	1/13/2023 15:00	12	13.0	12.9	-0.1
4A	1/13/2023 16:00	12	13.0	11.0	-2.0
4A	1/13/2023 17:00	12	13.0	11.0	-2.0
4A	1/13/2023 18:00	12	13.0	11.0	-2.0
4A	1/13/2023 19:00	12	13.0	11.0	-2.0
4A	1/13/2023 20:00	11	13.0	16.6	3.6
4A	1/13/2023 21:00	11	13.0	14.5	1.5
4A	1/13/2023 22:00	11	13.0	13.8	0.8
4A	1/13/2023 23:00	11	13.0	13.8	0.8
4A	1/14/2023 0:00	11	13.0	13.8	0.8
4A	1/14/2023 1:00	11	13.0	13.8	0.8
4A	1/14/2023 2:00	11	13.0	13.8	0.8
4A	1/14/2023 3:00	11	13.0	13.8	0.8
4A	1/14/2023 4:00	11	13.0	13.8	0.8
4A	1/14/2023 5:00	11	13.0	13.8	0.8

4A	1/14/2023 6:00	11	13.0	13.8	0.8
4A	1/14/2023 7:00	11	13.0	13.8	0.8
4A	1/14/2023 8:00	11	13.0	6.2	-6.8
4A	1/14/2023 9:00	11	13.0	6.2	-6.8
4A	1/14/2023 10:00	11	13.0	6.2	-6.8
4A	1/14/2023 11:00	11	13.0	6.2	-6.8
4A	1/14/2023 12:00	11	13.0	8.3	-4.7
4A	1/14/2023 13:00	11	13.0	8.3	-4.7
4A	1/14/2023 14:00	11	13.0	8.3	-4.7
4A	1/14/2023 15:00	11	13.0	8.3	-4.7
4A	1/14/2023 16:00	11	13.0	13.8	0.8
4A	1/14/2023 17:00	11	13.0	13.8	0.8
4A	1/14/2023 18:00	11	13.0	13.8	0.8
4A	1/14/2023 19:00	11	13.0	13.8	0.8
4A	1/14/2023 20:00	9	13.0	13.8	0.8
4A	1/14/2023 21:00	9	13.0	13.8	0.8
4A	1/14/2023 22:00	9	13.0	13.8	0.8
4A	1/14/2023 23:00	9	13.0	13.8	0.8
4A	1/15/2023 0:00	9	13.0	8.3	-4.7
4A	1/15/2023 1:00	9	13.0	8.3	-4.7
4A	1/15/2023 2:00	9	13.0	8.3	-4.7
4A	1/15/2023 3:00	9	13.0	8.3	-4.7
4A	1/15/2023 4:00	9	13.0	8.3	-4.7
4A	1/15/2023 5:00	9	13.0	8.3	-4.7
4A	1/15/2023 6:00	9	13.0	8.3	-4.7
4A	1/15/2023 7:00	9	13.0	8.3	-4.7
4A	1/15/2023 8:00	9	13.0	10.4	-2.7
4A	1/15/2023 9:00	9	13.0	10.4	-2.7
4A	1/15/2023 10:00	9	13.0	10.4	-2.7
4A	1/15/2023 11:00	9	13.0	10.4	-2.7
4A	1/15/2023 12:00	9	13.0	10.4	-2.7
4A	1/15/2023 13:00	9	13.0	10.4	-2.7
4A	1/15/2023 14:00	9	13.0	10.4	-2.7
4A	1/15/2023 15:00	9	13.0	10.4	-2.7
4A	1/15/2023 16:00	9	13.0	16.6	3.6
4A	1/15/2023 17:00	9	13.0	16.6	3.6
4A	1/15/2023 18:00	9	13.0	16.6	3.6
4A	1/15/2023 19:00	9	13.0	16.6	3.6
4A	1/15/2023 20:00	9	13.0	13.8	0.8
4A	1/15/2023 21:00	8	13.0	13.8	0.8
4A	1/15/2023 22:00	8	13.0	13.8	0.8
4A	1/15/2023 23:00	8	13.0	13.8	0.8
4A	1/16/2023 0:00	8	13.0	11.0	-2.0
4A	1/16/2023 1:00	8	13.0	11.0	-2.0
4A	1/16/2023 2:00	8	13.0	11.0	-2.0
4A	1/16/2023 3:00	8	13.0	11.0	-2.0
4A	1/16/2023 4:00	8	13.0	11.0	-2.0

4A	1/16/2023 5:00	8	13.0	11.0	-2.0
4A	1/16/2023 6:00	8	13.0	11.0	-2.0
4A	1/16/2023 7:00	8	13.0	11.0	-2.0
4A	1/16/2023 8:00	8	13.0	10.4	-2.7
4A	1/16/2023 9:00	8	13.0	10.4	-2.7
4A	1/16/2023 10:00	8	13.0	10.4	-2.7
4A	1/16/2023 11:00	8	13.0	10.4	-2.7
4A	1/16/2023 12:00	8	13.0	12.4	-0.6
4A	1/16/2023 13:00	8	13.0	12.4	-0.6
4A	1/16/2023 14:00	8	13.0	12.4	-0.6
4A	1/16/2023 15:00	8	13.0	12.4	-0.6
4A	1/16/2023 16:00	8	13.0	9.7	-3.3
4A	1/16/2023 17:00	8	13.0	13.8	0.8
4A	1/16/2023 18:00	8	13.0	13.8	0.8
4A	1/16/2023 19:00	8	13.0	13.8	0.8
4A	1/16/2023 20:00	8	13.0	11.0	-2.0
4A	1/16/2023 21:00	8	13.0	11.0	-2.0
4A	1/16/2023 22:00	8	13.0	11.0	-2.0
4A	1/16/2023 23:00	8	13.0	11.0	-2.0
4A	1/17/2023 0:00	8	13.0	13.8	0.8
4A	1/17/2023 1:00	8	13.0	13.8	0.8
4A	1/17/2023 2:00	8	13.0	13.8	0.8
4A	1/17/2023 3:00	8	13.0	13.8	0.8
4A	1/17/2023 4:00	8	13.0	13.8	0.8
4A	1/17/2023 5:00	8	13.0	13.8	0.8
4A	1/17/2023 6:00	8	13.0	13.8	0.8
4A	1/17/2023 7:00	8	13.0	13.8	0.8
4A	1/17/2023 8:00	8	13.0	10.4	-2.7
4A	1/17/2023 9:00	8	13.0	10.4	-2.7
4A	1/17/2023 10:00	8	13.0	10.4	-2.7
4A	1/17/2023 11:00	8	13.0	10.4	-2.7
4A	1/17/2023 12:00	8	13.0	7.8	-5.2
4A	1/17/2023 13:00	9	13.0	2.6	-10.4
4A	1/17/2023 14:00	9	13.0	7.8	-5.2
4A	1/17/2023 15:00	9	13.0	7.8	-5.2
4A	1/17/2023 16:00	9	13.0	4.1	-8.9
4A	1/17/2023 17:00	9	13.0	6.2	-6.8
4A	1/17/2023 18:00	9	13.0	8.3	-4.7
4A	1/17/2023 19:00	9	13.0	8.3	-4.7
4A	1/17/2023 20:00	8	13.0	9.7	-3.3
4A	1/17/2023 21:00	8	13.0	10.4	-2.7
4A	1/17/2023 22:00	8	13.0	10.4	-2.7
4A	1/17/2023 23:00	8	13.0	10.4	-2.7
4A	1/18/2023 0:00	8	13.0	10.4	-2.7
4A	1/18/2023 1:00	8	13.0	10.4	-2.7
4A	1/18/2023 2:00	8	13.0	10.4	-2.7
4A	1/18/2023 3:00	8	13.0	10.4	-2.7

4A	1/18/2023 4:00	8	13.0	10.4	-2.7
4A	1/18/2023 5:00	8	13.0	10.4	-2.7
4A	1/18/2023 6:00	8	13.0	10.4	-2.7
4A	1/18/2023 7:00	8	13.0	10.4	-2.7
4A	1/18/2023 8:00	8	13.0	10.4	-2.7
4A	1/18/2023 9:00	8	13.0	10.4	-2.7
4A	1/18/2023 10:00	8	13.0	10.4	-2.7
4A	1/18/2023 11:00	8	13.0	10.4	-2.7
4A	1/18/2023 12:00	8	13.0	10.4	-2.7
4A	1/18/2023 13:00	8	13.0	10.4	-2.7
4A	1/18/2023 14:00	8	13.0	10.4	-2.7
4A	1/18/2023 15:00	8	13.0	10.4	-2.7
4A	1/18/2023 16:00	8	13.0	13.8	0.8
4A	1/18/2023 17:00	8	13.0	13.8	0.8
4A	1/18/2023 18:00	8	13.0	13.8	0.8
4A	1/18/2023 19:00	8	13.0	13.8	0.8
4A	1/18/2023 20:00	8	13.0	6.9	-6.1
4A	1/18/2023 21:00	8	10.0	10.4	0.4
4A	1/18/2023 22:00	8	10.0	10.4	0.4
4A	1/18/2023 23:00	8	10.0	10.4	0.4
4A	1/19/2023 0:00	9	10.0	13.8	3.8
4A	1/19/2023 1:00	9	10.0	13.8	3.8
4A	1/19/2023 2:00	9	10.0	13.8	3.8
4A	1/19/2023 3:00	9	10.0	13.8	3.8
4A	1/19/2023 4:00	9	10.0	13.8	3.8
4A	1/19/2023 5:00	9	10.0	13.8	3.8
4A	1/19/2023 6:00	9	10.0	13.8	3.8
4A	1/19/2023 7:00	9	10.0	13.8	3.8
4A	1/19/2023 8:00	9	13.0	10.4	-2.7
4A	1/19/2023 9:00	9	13.0	10.4	-2.7
4A	1/19/2023 10:00	9	13.0	9.3	-3.7
4A	1/19/2023 11:00	9	13.0	8.3	-4.7
4A	1/19/2023 12:00	9	13.0	9.3	-3.7
4A	1/19/2023 13:00	9	13.0	10.4	-2.7
4A	1/19/2023 14:00	9	13.0	10.4	-2.7
4A	1/19/2023 15:00	9	13.0	10.4	-2.7
4A	1/19/2023 16:00	9	13.0	13.8	0.8
4A	1/19/2023 17:00	9	13.0	13.8	0.8
4A	1/19/2023 18:00	9	13.0	13.8	0.8
4A	1/19/2023 19:00	9	13.0	13.8	0.8
4A	1/19/2023 20:00	9	13.0	13.8	0.8
4A	1/19/2023 21:00	9	13.0	13.8	0.8
4A	1/19/2023 22:00	9	13.0	13.8	0.8
4A	1/19/2023 23:00	9	13.0	13.8	0.8
4A	1/20/2023 0:00	11	14.0	13.8	-0.2
4A	1/20/2023 1:00	11	14.0	13.8	-0.2
4A	1/20/2023 2:00	11	14.0	13.8	-0.2

4A	1/20/2023 3:00	11	14.0	13.8	-0.2
4A	1/20/2023 4:00	11	14.0	13.8	-0.2
4A	1/20/2023 5:00	11	14.0	13.8	-0.2
4A	1/20/2023 6:00	11	14.0	13.8	-0.2
4A	1/20/2023 7:00	11	14.0	13.8	-0.2
4A	1/20/2023 8:00	11	13.0	10.4	-2.7
4A	1/20/2023 9:00	11	13.0	10.4	-2.7
4A	1/20/2023 10:00	11	13.0	10.4	-2.7
4A	1/20/2023 11:00	11	13.0	10.4	-2.7
4A	1/20/2023 12:00	11	13.0	10.4	-2.7
4A	1/20/2023 13:00	11	13.0	10.4	-2.7
4A	1/20/2023 14:00	11	13.0	10.4	-2.7
4A	1/20/2023 15:00	11	13.0	8.3	-4.7
4A	1/20/2023 16:00	11	13.0	11.0	-2.0
4A	1/20/2023 17:00	11	13.0	11.0	-2.0
4A	1/20/2023 18:00	11	13.0	11.0	-2.0
4A	1/20/2023 19:00	11	13.0	11.0	-2.0
4A	1/20/2023 20:00	11	13.0	13.8	0.8
4A	1/20/2023 21:00	11	13.0	13.8	0.8
4A	1/20/2023 22:00	11	13.0	13.8	0.8
4A	1/20/2023 23:00	11	13.0	13.8	0.8
4A	1/21/2023 0:00	11	13.0	11.0	-2.0
4A	1/21/2023 1:00	11	13.0	11.0	-2.0
4A	1/21/2023 2:00	11	13.0	11.0	-2.0
4A	1/21/2023 3:00	11	13.0	11.0	-2.0
4A	1/21/2023 4:00	11	13.0	11.0	-2.0
4A	1/21/2023 5:00	11	13.0	11.0	-2.0
4A	1/21/2023 6:00	11	13.0	11.0	-2.0
4A	1/21/2023 7:00	11	13.0	12.4	-0.6
4A	1/21/2023 8:00	11	13.0	10.4	-2.7
4A	1/21/2023 9:00	11	13.0	10.4	-2.7
4A	1/21/2023 10:00	11	13.0	10.4	-2.7
4A	1/21/2023 11:00	11	13.0	10.4	-2.7
4A	1/21/2023 12:00	11	13.0	12.9	-0.1
4A	1/21/2023 13:00	11	13.0	12.9	-0.1
4A	1/21/2023 14:00	11	13.0	12.9	-0.1
4A	1/21/2023 15:00	11	13.0	12.9	-0.1
4A	1/21/2023 16:00	11	13.0	16.6	3.6
4A	1/21/2023 17:00	11	13.0	16.6	3.6
4A	1/21/2023 18:00	11	13.0	16.6	3.6
4A	1/21/2023 19:00	11	13.0	16.6	3.6
4A	1/21/2023 20:00	11	13.0	18.0	5.0
4A	1/21/2023 21:00	11	13.0	18.0	5.0
4A	1/21/2023 22:00	11	13.0	18.0	5.0
4A	1/21/2023 23:00	11	13.0	18.0	5.0
4A	1/22/2023 0:00	11	13.0	13.8	0.8
4A	1/22/2023 1:00	11	13.0	13.8	0.8

4A	1/22/2023 2:00	11	13.0	13.8	0.8
4A	1/22/2023 3:00	11	13.0	13.8	0.8
4A	1/22/2023 4:00	11	13.0	13.8	0.8
4A	1/22/2023 5:00	11	13.0	13.8	0.8
4A	1/22/2023 6:00	11	13.0	13.8	0.8
4A	1/22/2023 7:00	11	13.0	15.2	2.2
4A	1/22/2023 8:00	11	13.0	10.4	-2.7
4A	1/22/2023 9:00	11	13.0	10.4	-2.7
4A	1/22/2023 10:00	11	13.0	10.4	-2.7
4A	1/22/2023 11:00	11	13.0	10.4	-2.7
4A	1/22/2023 12:00	11	13.0	10.4	-2.7
4A	1/22/2023 13:00	11	13.0	10.4	-2.7
4A	1/22/2023 14:00	11	13.0	10.4	-2.7
4A	1/22/2023 15:00	11	13.0	10.4	-2.7
4A	1/22/2023 16:00	11	13.0	11.0	-2.0
4A	1/22/2023 17:00	11	13.0	11.0	-2.0
4A	1/22/2023 18:00	11	13.0	11.0	-2.0
4A	1/22/2023 19:00	11	13.0	11.0	-2.0
4A	1/22/2023 20:00	7	13.0	11.0	-2.0
4A	1/22/2023 21:00	7	13.0	11.0	-2.0
4A	1/22/2023 22:00	7	13.0	11.0	-2.0
4A	1/22/2023 23:00	7	13.0	11.0	-2.0
4A	1/23/2023 0:00	8	13.0	13.8	0.8
4A	1/23/2023 1:00	8	13.0	13.8	0.8
4A	1/23/2023 2:00	8	13.0	13.8	0.8
4A	1/23/2023 3:00	8	13.0	13.8	0.8
4A	1/23/2023 4:00	8	13.0	13.8	0.8
4A	1/23/2023 5:00	8	13.0	13.8	0.8
4A	1/23/2023 6:00	8	13.0	13.8	0.8
4A	1/23/2023 7:00	8	13.0	13.8	0.8
4A	1/23/2023 8:00	8	13.0	10.4	-2.7
4A	1/23/2023 9:00	8	13.0	10.4	-2.7
4A	1/23/2023 10:00	8	13.0	10.4	-2.7
4A	1/23/2023 11:00	8	13.0	10.4	-2.7
4A	1/23/2023 12:00	8	13.0	12.4	-0.6
4A	1/23/2023 13:00	8	13.0	12.4	-0.6
4A	1/23/2023 14:00	8	13.0	12.4	-0.6
4A	1/23/2023 15:00	8	13.0	12.4	-0.6
4A	1/23/2023 16:00	8	13.0	13.8	0.8
4A	1/23/2023 17:00	8	13.0	13.8	0.8
4A	1/23/2023 18:00	8	13.0	13.8	0.8
4A	1/23/2023 19:00	8	13.0	13.8	0.8
4A	1/23/2023 20:00	8	13.0	13.8	0.8
4A	1/23/2023 21:00	8	13.0	13.8	0.8
4A	1/23/2023 22:00	8	13.0	13.8	0.8
4A	1/23/2023 23:00	8	13.0	13.8	0.8
4A	1/24/2023 0:00	8	13.0	16.6	3.6

4A	1/24/2023 1:00	8	13.0	16.6	3.6
4A	1/24/2023 2:00	11	13.0	16.6	3.6
4A	1/24/2023 3:00	11	13.0	16.6	3.6
4A	1/24/2023 4:00	11	13.0	16.6	3.6
4A	1/24/2023 5:00	11	13.0	16.6	3.6
4A	1/24/2023 6:00	11	13.0	16.6	3.6
4A	1/24/2023 7:00	11	13.0	16.6	3.6
4A	1/24/2023 8:00	11	13.0	12.4	-0.6
4A	1/24/2023 9:00	11	13.0	12.4	-0.6
4A	1/24/2023 10:00	11	13.0	12.4	-0.6
4A	1/24/2023 11:00	11	13.0	12.4	-0.6
4A	1/24/2023 12:00	11	13.0	12.4	-0.6
4A	1/24/2023 13:00	11	13.0	12.4	-0.6
4A	1/24/2023 14:00	11	13.0	12.4	-0.6
4A	1/24/2023 15:00	11	13.0	12.4	-0.6
4A	1/24/2023 16:00	11	13.0	13.8	0.8
4A	1/24/2023 17:00	11	13.0	13.8	0.8
4A	1/24/2023 18:00	11	13.0	13.8	0.8
4A	1/24/2023 19:00	11	13.0	13.8	0.8
4A	1/24/2023 20:00	11	13.0	13.8	0.8
4A	1/24/2023 21:00	11	13.0	10.4	-2.7
4A	1/24/2023 22:00	11	13.0	10.4	-2.7
4A	1/24/2023 23:00	11	13.0	10.4	-2.7
4A	1/25/2023 0:00	11	13.0	10.4	-2.7
4A	1/25/2023 1:00	11	13.0	10.4	-2.7
4A	1/25/2023 2:00	11	13.0	10.4	-2.7
4A	1/25/2023 3:00	11	13.0	10.4	-2.7
4A	1/25/2023 4:00	11	13.0	10.4	-2.7
4A	1/25/2023 5:00	11	13.0	10.4	-2.7
4A	1/25/2023 6:00	11	13.0	10.4	-2.7
4A	1/25/2023 7:00	11	13.0	10.4	-2.7
4A	1/25/2023 8:00	11	13.0	12.9	-0.1
4A	1/25/2023 9:00	11	13.0	12.9	-0.1
4A	1/25/2023 10:00	11	13.0	12.9	-0.1
4A	1/25/2023 11:00	11	13.0	12.9	-0.1
4A	1/25/2023 12:00	11	13.0	12.9	-0.1
4A	1/25/2023 13:00	11	13.0	12.9	-0.1
4A	1/25/2023 14:00	11	13.0	12.9	-0.1
4A	1/25/2023 15:00	11	13.0	12.9	-0.1
4A	1/25/2023 16:00	7	13.0	16.6	3.6
4A	1/25/2023 17:00	7	13.0	16.6	3.6
4A	1/25/2023 18:00	7	13.0	16.6	3.6
4A	1/25/2023 19:00	7	13.0	16.6	3.6
4A	1/25/2023 20:00	8	13.0	13.8	0.8
4A	1/25/2023 21:00	8	13.0	13.8	0.8
4A	1/25/2023 22:00	8	13.0	13.8	0.8
4A	1/25/2023 23:00	8	13.0	13.8	0.8

4A	1/26/2023 0:00	8	13.0	17.2	4.2
4A	1/26/2023 1:00	8	13.0	17.2	4.2
4A	1/26/2023 2:00	8	13.0	17.2	4.2
4A	1/26/2023 3:00	8	13.0	17.2	4.2
4A	1/26/2023 4:00	8	13.0	17.2	4.2
4A	1/26/2023 5:00	8	13.0	17.2	4.2
4A	1/26/2023 6:00	8	13.0	17.2	4.2
4A	1/26/2023 7:00	8	13.0	17.2	4.2
4A	1/26/2023 8:00	8	13.0	10.4	-2.7
4A	1/26/2023 9:00	8	13.0	10.4	-2.7
4A	1/26/2023 10:00	8	13.0	10.4	-2.7
4A	1/26/2023 11:00	8	13.0	10.4	-2.7
4A	1/26/2023 12:00	8	13.0	7.8	-5.2
4A	1/26/2023 13:00	8	13.0	7.8	-5.2
4A	1/26/2023 14:00	8	13.0	7.8	-5.2
4A	1/26/2023 15:00	8	13.0	7.8	-5.2
4A	1/26/2023 16:00	8	13.0	10.4	-2.7
4A	1/26/2023 17:00	8	13.0	10.4	-2.7
4A	1/26/2023 18:00	8	13.0	10.4	-2.7
4A	1/26/2023 19:00	8	13.0	10.4	-2.7
4A	1/26/2023 20:00	8	13.0	10.4	-2.7
4A	1/26/2023 21:00	8	13.0	10.4	-2.7
4A	1/26/2023 22:00	8	13.0	10.4	-2.7
4A	1/26/2023 23:00	8	13.0	10.4	-2.7
4A	1/27/2023 0:00	8	13.0	10.4	-2.7
4A	1/27/2023 1:00	8	13.0	10.4	-2.7
4A	1/27/2023 2:00	11	13.0	10.4	-2.7
4A	1/27/2023 3:00	11	13.0	10.4	-2.7
4A	1/27/2023 4:00	11	13.0	10.4	-2.7
4A	1/27/2023 5:00	11	13.0	10.4	-2.7
4A	1/27/2023 6:00	11	13.0	10.4	-2.7
4A	1/27/2023 7:00	11	13.0	10.4	-2.7
4A	1/27/2023 8:00	11	13.0	10.4	-2.7
4A	1/27/2023 9:00	11	13.0	10.4	-2.7
4A	1/27/2023 10:00	11	13.0	10.4	-2.7
4A	1/27/2023 11:00	11	13.0	10.4	-2.7
4A	1/27/2023 12:00	11	13.0	10.4	-2.7
4A	1/27/2023 13:00	11	13.0	10.4	-2.7
4A	1/27/2023 14:00	11	13.0	10.4	-2.7
4A	1/27/2023 15:00	11	13.0	10.4	-2.7
4A	1/27/2023 16:00	11	13.0	10.4	-2.7
4A	1/27/2023 17:00	11	13.0	10.4	-2.7
4A	1/27/2023 18:00	11	13.0	10.4	-2.7
4A	1/27/2023 19:00	11	13.0	10.4	-2.7
4A	1/27/2023 20:00	11	13.0	13.8	0.8
4A	1/27/2023 21:00	11	13.0	13.8	0.8
4A	1/27/2023 22:00	11	13.0	13.8	0.8

4A	1/27/2023 23:00	11	13.0	13.8	0.8
4A	1/28/2023 0:00	11	13.0	11.0	-2.0
4A	1/28/2023 1:00	11	13.0	11.0	-2.0
4A	1/28/2023 2:00	11	13.0	11.0	-2.0
4A	1/28/2023 3:00	11	13.0	11.0	-2.0
4A	1/28/2023 4:00	11	13.0	11.0	-2.0
4A	1/28/2023 5:00	11	13.0	11.0	-2.0
4A	1/28/2023 6:00	11	13.0	11.0	-2.0
4A	1/28/2023 7:00	11	13.0	11.0	-2.0
4A	1/28/2023 8:00	10	13.0	10.4	-2.7
4A	1/28/2023 9:00	10	13.0	10.4	-2.7
4A	1/28/2023 10:00	10	13.0	10.4	-2.7
4A	1/28/2023 11:00	10	13.0	10.4	-2.7
4A	1/28/2023 12:00	10	13.0	12.9	-0.1
4A	1/28/2023 13:00	10	13.0	12.9	-0.1
4A	1/28/2023 14:00	10	13.0	12.9	-0.1
4A	1/28/2023 15:00	10	13.0	12.9	-0.1
4A	1/28/2023 16:00	10	13.0	17.2	4.2
4A	1/28/2023 17:00	10	13.0	17.2	4.2
4A	1/28/2023 18:00	10	13.0	17.2	4.2
4A	1/28/2023 19:00	10	13.0	17.2	4.2
4A	1/28/2023 20:00	10	13.0	17.2	4.2
4A	1/28/2023 21:00	10	13.0	17.2	4.2
4A	1/28/2023 22:00	10	13.0	17.2	4.2
4A	1/28/2023 23:00	10	13.0	17.2	4.2
4A	1/29/2023 0:00	10	13.0	13.8	0.8
4A	1/29/2023 1:00	10	13.0	13.8	0.8
4A	1/29/2023 2:00	10	13.0	13.8	0.8
4A	1/29/2023 3:00	10	13.0	13.8	0.8
4A	1/29/2023 4:00	10	13.0	13.8	0.8
4A	1/29/2023 5:00	10	13.0	13.8	0.8
4A	1/29/2023 6:00	10	13.0	13.8	0.8
4A	1/29/2023 7:00	10	13.0	13.8	0.8
4A	1/29/2023 8:00	10	13.0	8.3	-4.7
4A	1/29/2023 9:00	10	13.0	8.3	-4.7
4A	1/29/2023 10:00	10	13.0	8.3	-4.7
4A	1/29/2023 11:00	10	13.0	8.3	-4.7
4A	1/29/2023 12:00	10	13.0	10.4	-2.7
4A	1/29/2023 13:00	10	13.0	10.4	-2.7
4A	1/29/2023 14:00	10	13.0	10.4	-2.7
4A	1/29/2023 15:00	10	13.0	10.4	-2.7
4A	1/29/2023 16:00	9	13.0	13.8	0.8
4A	1/29/2023 17:00	9	13.0	13.8	0.8
4A	1/29/2023 18:00	9	13.0	13.8	0.8
4A	1/29/2023 19:00	9	13.0	13.8	0.8
4A	1/29/2023 20:00	9	13.0	13.8	0.8
4A	1/29/2023 21:00	9	13.0	13.8	0.8

4A	1/29/2023 22:00	9	13.0	13.1	0.1
4A	1/29/2023 23:00	9	13.0	11.0	-2.0
4A	1/30/2023 0:00	9	13.0	5.5	-7.5
4A	1/30/2023 1:00	9	13.0	5.5	-7.5
4A	1/30/2023 2:00	9	13.0	5.5	-7.5
4A	1/30/2023 3:00	9	13.0	5.5	-7.5
4A	1/30/2023 4:00	9	13.0	5.5	-7.5
4A	1/30/2023 5:00	9	13.0	5.5	-7.5
4A	1/30/2023 6:00	9	13.0	5.5	-7.5
4A	1/30/2023 7:00	9	13.0	5.5	-7.5
4A	1/30/2023 8:00	9	13.0	8.3	-4.7
4A	1/30/2023 9:00	9	13.0	8.3	-4.7
4A	1/30/2023 10:00	9	13.0	8.3	-4.7
4A	1/30/2023 11:00	9	13.0	8.3	-4.7
4A	1/30/2023 12:00	9	13.0	9.3	-3.7
4A	1/30/2023 13:00	9	13.0	10.4	-2.7
4A	1/30/2023 14:00	9	13.0	10.4	-2.7
4A	1/30/2023 15:00	9	13.0	10.4	-2.7
4A	1/30/2023 16:00	9	13.0	13.8	0.8
4A	1/30/2023 17:00	9	13.0	13.8	0.8
4A	1/30/2023 18:00	9	13.0	13.8	0.8
4A	1/30/2023 19:00	9	13.0	13.8	0.8
4A	1/30/2023 20:00	9	13.0	11.0	-2.0
4A	1/30/2023 21:00	12	13.0	13.8	0.8
4A	1/30/2023 22:00	12	13.0	13.8	0.8
4A	1/30/2023 23:00	12	13.0	12.1	-0.9
4A	1/31/2023 0:00	12	13.0	11.0	-2.0
4A	1/31/2023 1:00	12	13.0	11.0	-2.0
4A	1/31/2023 2:00	12	13.0	11.0	-2.0
4A	1/31/2023 3:00	12	13.0	11.0	-2.0
4A	1/31/2023 4:00	12	13.0	11.0	-2.0
4A	1/31/2023 5:00	12	13.0	11.0	-2.0
4A	1/31/2023 6:00	12	13.0	11.0	-2.0
4A	1/31/2023 7:00	12	13.0	12.4	-0.6
4A	1/31/2023 8:00	12	13.0	15.5	2.5
4A	1/31/2023 9:00	12	13.0	15.5	2.5
4A	1/31/2023 10:00	12	13.0	15.5	2.5
4A	1/31/2023 11:00	12	13.0	15.5	2.5
4A	1/31/2023 12:00	12	13.0	12.9	-0.1
4A	1/31/2023 13:00	12	13.0	12.9	-0.1
4A	1/31/2023 14:00	12	13.0	12.9	-0.1
4A	1/31/2023 15:00	12	13.0	12.9	-0.1
4A	1/31/2023 16:00	12	13.0	17.2	4.2
4A	1/31/2023 17:00	12	13.0	17.2	4.2
4A	1/31/2023 18:00	12	13.0	17.2	4.2
4A	1/31/2023 19:00	12	13.0	17.2	4.2
4A	1/31/2023 20:00	12	13.0	13.8	0.8

4A	1/31/2023 21:00	12	13.0	13.8	0.8
4A	1/31/2023 22:00	12	13.0	13.8	0.8
4A	1/31/2023 23:00	12	13.0	13.8	0.8
4A	2/1/2023 0:00	11	13.0	10.4	-2.7
4A	2/1/2023 1:00	11	13.0	10.4	-2.7
4A	2/1/2023 2:00	11	13.0	10.4	-2.7
4A	2/1/2023 3:00	11	13.0	10.4	-2.7
4A	2/1/2023 4:00	11	13.0	10.4	-2.7
4A	2/1/2023 5:00	11	13.0	10.4	-2.7
4A	2/1/2023 6:00	12	13.0	10.4	-2.7
4A	2/1/2023 7:00	12	13.0	10.4	-2.7
4A	2/1/2023 8:00	12	13.0	10.4	-2.7
4A	2/1/2023 9:00	12	13.0	10.4	-2.7
4A	2/1/2023 10:00	12	13.0	10.4	-2.7
4A	2/1/2023 11:00	12	13.0	10.4	-2.7
4A	2/1/2023 12:00	12	13.0	10.4	-2.7
4A	2/1/2023 13:00	12	13.0	10.4	-2.7
4A	2/1/2023 14:00	12	13.0	10.4	-2.7
4A	2/1/2023 15:00	12	13.0	10.4	-2.7
4A	2/1/2023 16:00	12	13.0	13.8	0.8
4A	2/1/2023 17:00	12	13.0	13.8	0.8
4A	2/1/2023 18:00	12	13.0	13.8	0.8
4A	2/1/2023 19:00	12	13.0	13.8	0.8
4A	2/1/2023 20:00	10	13.0	10.4	-2.7
4A	2/1/2023 21:00	10	13.0	10.4	-2.7
4A	2/1/2023 22:00	10	13.0	10.4	-2.7
4A	2/1/2023 23:00	11	13.0	10.4	-2.7
4A	2/2/2023 0:00	11	13.0	10.4	-2.7
4A	2/2/2023 1:00	11	13.0	10.4	-2.7
4A	2/2/2023 2:00	11	13.0	10.4	-2.7
4A	2/2/2023 3:00	11	13.0	10.4	-2.7
4A	2/2/2023 4:00	11	13.0	10.4	-2.7
4A	2/2/2023 5:00	11	13.0	10.4	-2.7
4A	2/2/2023 6:00	11	13.0	10.4	-2.7
4A	2/2/2023 7:00	11	13.0	10.4	-2.7
4A	2/2/2023 8:00	11	13.0	10.4	-2.7
4A	2/2/2023 9:00	11	13.0	10.4	-2.7
4A	2/2/2023 10:00	11	13.0	10.4	-2.7
4A	2/2/2023 11:00	11	13.0	10.4	-2.7
4A	2/2/2023 12:00	11	13.0	12.9	-0.1
4A	2/2/2023 13:00	11	13.0	12.9	-0.1
4A	2/2/2023 14:00	11	13.0	12.9	-0.1
4A	2/2/2023 15:00	11	13.0	12.9	-0.1
4A	2/2/2023 16:00	11	13.0	16.6	3.6
4A	2/2/2023 17:00	12	13.0	16.6	3.6
4A	2/2/2023 18:00	12	13.0	16.6	3.6
4A	2/2/2023 19:00	12	13.0	16.6	3.6

4A	2/2/2023 20:00	12	13.0	16.6	3.6
4A	2/2/2023 21:00	12	13.0	16.6	3.6
4A	2/2/2023 22:00	12	13.0	16.6	3.6
4A	2/2/2023 23:00	12	13.0	16.6	3.6
4A	2/3/2023 0:00	12	13.0	13.8	0.8
4A	2/3/2023 1:00	12	13.0	13.8	0.8
4A	2/3/2023 2:00	12	13.0	13.8	0.8
4A	2/3/2023 3:00	12	13.0	13.8	0.8
4A	2/3/2023 4:00	12	13.0	13.8	0.8
4A	2/3/2023 5:00	12	13.0	13.8	0.8
4A	2/3/2023 6:00	12	13.0	13.8	0.8
4A	2/3/2023 7:00	12	13.0	13.8	0.8
4A	2/3/2023 8:00	12	13.0	7.8	-5.2
4A	2/3/2023 9:00	12	13.0	7.8	-5.2
4A	2/3/2023 10:00	12	13.0	7.8	-5.2
4A	2/3/2023 11:00	12	13.0	7.8	-5.2
4A	2/3/2023 12:00	12	13.0	7.8	-5.2
4A	2/3/2023 13:00	12	13.0	7.8	-5.2
4A	2/3/2023 14:00	12	13.0	7.8	-5.2
4A	2/3/2023 15:00	12	13.0	7.2	-5.8
4A	2/3/2023 16:00	12	13.0	13.8	0.8
4A	2/3/2023 17:00	12	13.0	13.8	0.8
4A	2/3/2023 18:00	12	13.0	13.8	0.8
4A	2/3/2023 19:00	12	13.0	12.1	-0.9
4A	2/3/2023 20:00	12	13.0	10.4	-2.7
4A	2/3/2023 21:00	12	13.0	10.4	-2.7
4A	2/3/2023 22:00	12	13.0	10.4	-2.7
4A	2/3/2023 23:00	12	13.0	10.4	-2.7
4A	2/4/2023 0:00	11	13.0	2.6	-10.4
4A	2/4/2023 1:00	11	13.0	2.6	-10.4
4A	2/4/2023 2:00	11	13.0	2.6	-10.4
4A	2/4/2023 3:00	11	13.0	2.6	-10.4
4A	2/4/2023 4:00	11	13.0	2.6	-10.4
4A	2/4/2023 5:00	11	13.0	2.6	-10.4
4A	2/4/2023 6:00	11	13.0	2.6	-10.4
4A	2/4/2023 7:00	11	13.0	2.6	-10.4
4A	2/4/2023 8:00	11	13.0	12.4	-0.6
4A	2/4/2023 9:00	11	13.0	12.4	-0.6
4A	2/4/2023 10:00	11	13.0	12.4	-0.6
4A	2/4/2023 11:00	11	13.0	12.4	-0.6
4A	2/4/2023 12:00	11	13.0	10.4	-2.7
4A	2/4/2023 13:00	11	13.0	10.4	-2.7
4A	2/4/2023 14:00	11	13.0	10.4	-2.7
4A	2/4/2023 15:00	11	13.0	10.4	-2.7
4A	2/4/2023 16:00	11	13.0	8.3	-4.7
4A	2/4/2023 17:00	11	13.0	8.3	-4.7
4A	2/4/2023 18:00	11	13.0	8.3	-4.7

4A	2/4/2023 19:00	11	13.0	8.3	-4.7
4A	2/4/2023 20:00	11	13.0	13.8	0.8
4A	2/4/2023 21:00	11	13.0	13.8	0.8
4A	2/4/2023 22:00	11	13.0	13.8	0.8
4A	2/4/2023 23:00	11	13.0	13.8	0.8
4A	2/5/2023 0:00	11	13.0	11.0	-2.0
4A	2/5/2023 1:00	11	13.0	11.0	-2.0
4A	2/5/2023 2:00	11	13.0	11.0	-2.0
4A	2/5/2023 3:00	11	13.0	11.0	-2.0
4A	2/5/2023 4:00	11	13.0	11.0	-2.0
4A	2/5/2023 5:00	11	13.0	11.0	-2.0
4A	2/5/2023 6:00	11	13.0	11.0	-2.0
4A	2/5/2023 7:00	11	13.0	11.0	-2.0
4A	2/5/2023 8:00	11	13.0	12.4	-0.6
4A	2/5/2023 9:00	11	13.0	12.9	-0.1
4A	2/5/2023 10:00	11	13.0	12.9	-0.1
4A	2/5/2023 11:00	11	13.0	12.9	-0.1
4A	2/5/2023 12:00	11	13.0	12.9	-0.1
4A	2/5/2023 13:00	11	13.0	12.9	-0.1
4A	2/5/2023 14:00	11	13.0	12.9	-0.1
4A	2/5/2023 15:00	11	13.0	12.9	-0.1
4A	2/5/2023 16:00	11	13.0	8.3	-4.7
4A	2/5/2023 17:00	11	13.0	8.3	-4.7
4A	2/5/2023 18:00	11	13.0	8.3	-4.7
4A	2/5/2023 19:00	11	13.0	8.3	-4.7
4A	2/5/2023 20:00	11	13.0	16.6	3.6
4A	2/5/2023 21:00	11	13.0	16.6	3.6
4A	2/5/2023 22:00	11	13.0	16.6	3.6
4A	2/5/2023 23:00	11	13.0	16.6	3.6
4A	2/6/2023 0:00	11	13.0	13.8	0.8
4A	2/6/2023 1:00	11	13.0	13.8	0.8
4A	2/6/2023 2:00	11	13.0	13.8	0.8
4A	2/6/2023 3:00	11	13.0	13.8	0.8
4A	2/6/2023 4:00	11	13.0	13.8	0.8
4A	2/6/2023 5:00	11	13.0	13.8	0.8
4A	2/6/2023 6:00	11	13.0	13.8	0.8
4A	2/6/2023 7:00	11	13.0	13.8	0.8
4A	2/6/2023 8:00	11	13.0	12.9	-0.1
4A	2/6/2023 9:00	11	13.0	12.9	-0.1
4A	2/6/2023 10:00	11	13.0	12.9	-0.1
4A	2/6/2023 11:00	11	13.0	12.9	-0.1
4A	2/6/2023 12:00	11	13.0	12.9	-0.1
4A	2/6/2023 13:00	11	13.0	12.9	-0.1
4A	2/6/2023 14:00	11	13.0	12.9	-0.1
4A	2/6/2023 15:00	11	13.0	12.9	-0.1
4A	2/6/2023 16:00	11	13.0	13.8	0.8
4A	2/6/2023 17:00	11	13.0	13.8	0.8

4A	2/6/2023 18:00	11	13.0	13.8	0.8
4A	2/6/2023 19:00	11	13.0	13.8	0.8
4A	2/6/2023 20:00	11	13.0	13.8	0.8
4A	2/6/2023 21:00	11	13.0	13.8	0.8
4A	2/6/2023 22:00	11	13.0	13.8	0.8
4A	2/6/2023 23:00	11	13.0	13.8	0.8
4A	2/7/2023 0:00	11	13.0	13.8	0.8
4A	2/7/2023 1:00	11	13.0	13.8	0.8
4A	2/7/2023 2:00	11	13.0	13.8	0.8
4A	2/7/2023 3:00	11	13.0	13.8	0.8
4A	2/7/2023 4:00	11	13.0	13.8	0.8
4A	2/7/2023 5:00	11	13.0	13.8	0.8
4A	2/7/2023 6:00	11	13.0	13.8	0.8
4A	2/7/2023 7:00	11	13.0	13.8	0.8
4A	2/7/2023 8:00	11	13.0	10.4	-2.7
4A	2/7/2023 9:00	11	13.0	10.4	-2.7
4A	2/7/2023 10:00	11	13.0	10.4	-2.7
4A	2/7/2023 11:00	11	13.0	10.4	-2.7
4A	2/7/2023 12:00	11	13.0	12.9	-0.1
4A	2/7/2023 13:00	11	13.0	12.9	-0.1
4A	2/7/2023 14:00	11	13.0	12.9	-0.1
4A	2/7/2023 15:00	11	13.0	12.9	-0.1
4A	2/7/2023 16:00	11	13.0	13.8	0.8
4A	2/7/2023 17:00	11	13.0	13.8	0.8
4A	2/7/2023 18:00	11	13.0	13.8	0.8
4A	2/7/2023 19:00	11	13.0	13.8	0.8
4A	2/7/2023 20:00	11	13.0	13.8	0.8
4A	2/7/2023 21:00	12	13.0	13.8	0.8
4A	2/7/2023 22:00	12	13.0	13.8	0.8
4A	2/7/2023 23:00	12	13.0	13.8	0.8
4A	2/8/2023 0:00	12	13.0	13.8	0.8
4A	2/8/2023 1:00	12	13.0	13.8	0.8
4A	2/8/2023 2:00	12	13.0	13.8	0.8
4A	2/8/2023 3:00	12	13.0	13.8	0.8
4A	2/8/2023 4:00	12	13.0	13.8	0.8
4A	2/8/2023 5:00	12	13.0	13.8	0.8
4A	2/8/2023 6:00	12	13.0	13.8	0.8
4A	2/8/2023 7:00	12	13.0	13.8	0.8
4A	2/8/2023 8:00	12	13.0	10.4	-2.7
4A	2/8/2023 9:00	12	13.0	10.4	-2.7
4A	2/8/2023 10:00	12	13.0	10.4	-2.7
4A	2/8/2023 11:00	12	13.0	10.4	-2.7
4A	2/8/2023 12:00	12	13.0	10.4	-2.7
4A	2/8/2023 13:00	12	13.0	10.4	-2.7
4A	2/8/2023 14:00	12	13.0	10.4	-2.7
4A	2/8/2023 15:00	12	13.0	10.4	-2.7
4A	2/8/2023 16:00	12	13.0	13.8	0.8

4A	2/8/2023 17:00	12	13.0	13.8	0.8
4A	2/8/2023 18:00	12	13.0	13.8	0.8
4A	2/8/2023 19:00	12	13.0	13.8	0.8
4A	2/8/2023 20:00	10	13.0	13.8	0.8
4A	2/8/2023 21:00	10	13.0	13.8	0.8
4A	2/8/2023 22:00	10	13.0	13.8	0.8
4A	2/8/2023 23:00	10	13.0	13.8	0.8
4A	2/9/2023 0:00	10	13.0	17.2	4.2
4A	2/9/2023 1:00	10	13.0	17.2	4.2
4A	2/9/2023 2:00	10	13.0	17.2	4.2
4A	2/9/2023 3:00	10	13.0	17.2	4.2
4A	2/9/2023 4:00	10	13.0	17.2	4.2
4A	2/9/2023 5:00	10	13.0	17.2	4.2
4A	2/9/2023 6:00	10	13.0	17.2	4.2
4A	2/9/2023 7:00	10	13.0	17.2	4.2
4A	2/9/2023 8:00	10	13.0	10.4	-2.7
4A	2/9/2023 9:00	10	13.0	10.4	-2.7
4A	2/9/2023 10:00	10	13.0	10.4	-2.7
4A	2/9/2023 11:00	10	13.0	10.4	-2.7
4A	2/9/2023 12:00	10	13.0	7.8	-5.2
4A	2/9/2023 13:00	10	13.0	7.8	-5.2
4A	2/9/2023 14:00	10	13.0	7.8	-5.2
4A	2/9/2023 15:00	10	13.0	7.8	-5.2
4A	2/9/2023 16:00	10	13.0	10.4	-2.7
4A	2/9/2023 17:00	10	13.0	10.4	-2.7
4A	2/9/2023 18:00	10	13.0	10.4	-2.7
4A	2/9/2023 19:00	10	13.0	10.4	-2.7
4A	2/9/2023 20:00	10	13.0	10.4	-2.7
4A	2/9/2023 21:00	10	13.0	10.4	-2.7
4A	2/9/2023 22:00	10	13.0	10.4	-2.7
4A	2/9/2023 23:00	10	13.0	10.4	-2.7
4A	2/10/2023 0:00	9	13.0	16.6	3.6
4A	2/10/2023 1:00	9	13.0	16.6	3.6
4A	2/10/2023 2:00	9	13.0	16.6	3.6
4A	2/10/2023 3:00	9	13.0	16.6	3.6
4A	2/10/2023 4:00	9	13.0	16.6	3.6
4A	2/10/2023 5:00	9	13.0	16.6	3.6
4A	2/10/2023 6:00	11	13.0	16.6	3.6
4A	2/10/2023 7:00	11	13.0	17.2	4.2
4A	2/10/2023 8:00	11	13.0	10.4	-2.7
4A	2/10/2023 9:00	11	13.0	10.4	-2.7
4A	2/10/2023 10:00	11	13.0	10.4	-2.7
4A	2/10/2023 11:00	11	13.0	10.4	-2.7
4A	2/10/2023 12:00	11	13.0	10.4	-2.7
4A	2/10/2023 13:00	11	13.0	10.4	-2.7
4A	2/10/2023 14:00	11	13.0	10.4	-2.7
4A	2/10/2023 15:00	11	13.0	10.4	-2.7

4A	2/10/2023 16:00	11	13.0	10.4	-2.7
4A	2/10/2023 17:00	11	13.0	10.4	-2.7
4A	2/10/2023 18:00	11	13.0	10.4	-2.7
4A	2/10/2023 19:00	11	13.0	10.4	-2.7
4A	2/10/2023 20:00	7	13.0	17.2	4.2
4A	2/10/2023 21:00	7	13.0	17.2	4.2
4A	2/10/2023 22:00	7	13.0	17.2	4.2
4A	2/10/2023 23:00	9	13.0	17.2	4.2
4A	2/11/2023 0:00	9	13.0	13.8	0.8
4A	2/11/2023 1:00	9	13.0	13.8	0.8
4A	2/11/2023 2:00	9	13.0	13.8	0.8
4A	2/11/2023 3:00	9	13.0	13.8	0.8
4A	2/11/2023 4:00	9	13.0	13.8	0.8
4A	2/11/2023 5:00	9	13.0	13.8	0.8
4A	2/11/2023 6:00	9	13.0	13.8	0.8
4A	2/11/2023 7:00	9	13.0	13.8	0.8
4A	2/11/2023 8:00	9	13.0	7.8	-5.2
4A	2/11/2023 9:00	9	13.0	7.8	-5.2
4A	2/11/2023 10:00	9	13.0	7.8	-5.2
4A	2/11/2023 11:00	9	13.0	7.8	-5.2
4A	2/11/2023 12:00	9	13.0	10.4	-2.7
4A	2/11/2023 13:00	9	13.0	10.4	-2.7
4A	2/11/2023 14:00	9	13.0	10.4	-2.7
4A	2/11/2023 15:00	9	13.0	10.4	-2.7
4A	2/11/2023 16:00	9	13.0	13.8	0.8
4A	2/11/2023 17:00	9	13.0	13.8	0.8
4A	2/11/2023 18:00	9	13.0	13.8	0.8
4A	2/11/2023 19:00	10	13.0	13.8	0.8
4A	2/11/2023 20:00	10	13.0	17.2	4.2
4A	2/11/2023 21:00	10	13.0	17.2	4.2
4A	2/11/2023 22:00	10	13.0	17.2	4.2
4A	2/11/2023 23:00	10	13.0	17.2	4.2
4A	2/12/2023 0:00	10	13.0	13.8	0.8
4A	2/12/2023 1:00	10	13.0	13.8	0.8
4A	2/12/2023 2:00	10	13.0	13.8	0.8
4A	2/12/2023 3:00	10	13.0	13.8	0.8
4A	2/12/2023 4:00	10	13.0	13.8	0.8
4A	2/12/2023 5:00	10	13.0	13.8	0.8
4A	2/12/2023 6:00	11	13.0	13.8	0.8
4A	2/12/2023 7:00	11	13.0	13.8	0.8
4A	2/12/2023 8:00	11	13.0	7.8	-5.2
4A	2/12/2023 9:00	11	13.0	7.8	-5.2
4A	2/12/2023 10:00	11	13.0	7.8	-5.2
4A	2/12/2023 11:00	11	13.0	7.8	-5.2
4A	2/12/2023 12:00	11	13.0	10.4	-2.7
4A	2/12/2023 13:00	11	13.0	10.4	-2.7
4A	2/12/2023 14:00	11	13.0	10.4	-2.7

4A	2/12/2023 15:00	11	13.0	10.4	-2.7
4A	2/12/2023 16:00	10	13.0	13.8	0.8
4A	2/12/2023 17:00	10	13.0	13.8	0.8
4A	2/12/2023 18:00	10	13.0	13.8	0.8
4A	2/12/2023 19:00	10	13.0	13.8	0.8
4A	2/12/2023 20:00	10	13.0	17.2	4.2
4A	2/12/2023 21:00	10	13.0	17.2	4.2
4A	2/12/2023 22:00	10	13.0	17.2	4.2
4A	2/12/2023 23:00	10	13.0	17.2	4.2
4A	2/13/2023 0:00	10	13.0	13.8	0.8
4A	2/13/2023 1:00	10	13.0	13.8	0.8
4A	2/13/2023 2:00	10	13.0	13.8	0.8
4A	2/13/2023 3:00	10	13.0	13.8	0.8
4A	2/13/2023 4:00	10	13.0	13.8	0.8
4A	2/13/2023 5:00	10	13.0	13.8	0.8
4A	2/13/2023 6:00	10	13.0	13.8	0.8
4A	2/13/2023 7:00	10	13.0	13.8	0.8
4A	2/13/2023 8:00	10	13.0	7.8	-5.2
4A	2/13/2023 9:00	10	13.0	7.8	-5.2
4A	2/13/2023 10:00	10	13.0	7.8	-5.2
4A	2/13/2023 11:00	10	13.0	7.8	-5.2
4A	2/13/2023 12:00	10	13.0	7.8	-5.2
4A	2/13/2023 13:00	10	13.0	7.8	-5.2
4A	2/13/2023 14:00	10	13.0	7.8	-5.2
4A	2/13/2023 15:00	10	13.0	7.8	-5.2
4A	2/13/2023 16:00	10	13.0	10.4	-2.7
4A	2/13/2023 17:00	10	13.0	10.4	-2.7
4A	2/13/2023 18:00	10	13.0	10.4	-2.7
4A	2/13/2023 19:00	10	13.0	10.4	-2.7
4A	2/13/2023 20:00	10	13.0	13.8	0.8
4A	2/13/2023 21:00	10	13.0	13.8	0.8
4A	2/13/2023 22:00	11	13.0	13.8	0.8
4A	2/13/2023 23:00	11	13.0	13.8	0.8
4A	2/14/2023 0:00	11	13.0	13.8	0.8
4A	2/14/2023 1:00	11	13.0	13.8	0.8
4A	2/14/2023 2:00	11	13.0	13.8	0.8
4A	2/14/2023 3:00	11	13.0	13.8	0.8
4A	2/14/2023 4:00	11	13.0	13.8	0.8
4A	2/14/2023 5:00	11	13.0	13.8	0.8
4A	2/14/2023 6:00	11	13.0	13.8	0.8
4A	2/14/2023 7:00	11	13.0	13.8	0.8
4A	2/14/2023 8:00	11	13.0	6.2	-6.8
4A	2/14/2023 9:00	11	13.0	6.2	-6.8
4A	2/14/2023 10:00	11	13.0	6.2	-6.8
4A	2/14/2023 11:00	11	13.0	6.2	-6.8
4A	2/14/2023 12:00	11	13.0	8.3	-4.7
4A	2/14/2023 13:00	11	13.0	8.3	-4.7

4A	2/14/2023 14:00	11	13.0	8.3	-4.7
4A	2/14/2023 15:00	11	13.0	8.3	-4.7
4A	2/14/2023 16:00	11	13.0	13.8	0.8
4A	2/14/2023 17:00	11	13.0	13.8	0.8
4A	2/14/2023 18:00	11	13.0	13.8	0.8
4A	2/14/2023 19:00	11	13.0	13.8	0.8
4A	2/14/2023 20:00	11	13.0	17.2	4.2
4A	2/14/2023 21:00	11	13.0	17.2	4.2
4A	2/14/2023 22:00	11	13.0	17.2	4.2
4A	2/14/2023 23:00	11	13.0	17.2	4.2
4A	2/15/2023 0:00	11	13.0	16.6	3.6
4A	2/15/2023 1:00	11	13.0	16.6	3.6
4A	2/15/2023 2:00	12	13.0	16.6	3.6
4A	2/15/2023 3:00	12	13.0	16.6	3.6
4A	2/15/2023 4:00	12	13.0	16.6	3.6
4A	2/15/2023 5:00	12	13.0	16.6	3.6
4A	2/15/2023 6:00	12	13.0	16.6	3.6
4A	2/15/2023 7:00	12	13.0	17.2	4.2
4A	2/15/2023 8:00	12	13.0	12.9	-0.1
4A	2/15/2023 9:00	12	13.0	12.9	-0.1
4A	2/15/2023 10:00	12	13.0	12.9	-0.1
4A	2/15/2023 11:00	12	13.0	12.9	-0.1
4A	2/15/2023 12:00	12	13.0	15.5	2.5
4A	2/15/2023 13:00	12	13.0	18.0	5.0
4A	2/15/2023 14:00	12	13.0	18.0	5.0
4A	2/15/2023 15:00	12	13.0	18.0	5.0
4A	2/15/2023 16:00	12	13.0	18.0	5.0
4A	2/15/2023 17:00	12	13.0	18.0	5.0
4A	2/15/2023 18:00	12	13.0	18.0	5.0
4A	2/15/2023 19:00	12	13.0	18.0	5.0
4A	2/15/2023 20:00	12	13.0	18.0	5.0
4A	2/15/2023 21:00	12	13.0	18.0	5.0
4A	2/15/2023 22:00	12	13.0	18.0	5.0
4A	2/15/2023 23:00	12	13.0	18.0	5.0
4A	2/16/2023 0:00	12	13.0	16.6	3.6
4A	2/16/2023 1:00	12	13.0	16.6	3.6
4A	2/16/2023 2:00	10	13.0	16.6	3.6
4A	2/16/2023 3:00	10	13.0	16.6	3.6
4A	2/16/2023 4:00	10	13.0	16.6	3.6
4A	2/16/2023 5:00	10	13.0	16.6	3.6
4A	2/16/2023 6:00	10	13.0	16.6	3.6
4A	2/16/2023 7:00	10	13.0	16.6	3.6
4A	2/16/2023 8:00	10	13.0	10.4	-2.7
4A	2/16/2023 9:00	10	13.0	10.4	-2.7
4A	2/16/2023 10:00	10	13.0	10.4	-2.7
4A	2/16/2023 11:00	10	13.0	10.4	-2.7
4A	2/16/2023 12:00	10	13.0	7.8	-5.2

4A	2/16/2023 13:00	10	13.0	7.8	-5.2
4A	2/16/2023 14:00	10	13.0	7.8	-5.2
4A	2/16/2023 15:00	10	13.0	7.8	-5.2
4A	2/16/2023 16:00	6	13.0	10.4	-2.7
4A	2/16/2023 17:00	6	13.0	10.4	-2.7
4A	2/16/2023 18:00	6	13.0	10.4	-2.7
4A	2/16/2023 19:00	6	13.0	10.4	-2.7
4A	2/16/2023 20:00	7	13.0	14.7	1.7
4A	2/16/2023 21:00	7	13.0	13.8	0.8
4A	2/16/2023 22:00	7	13.0	13.8	0.8
4A	2/16/2023 23:00	7	13.0	13.8	0.8
4A	2/17/2023 0:00	8	13.0	13.8	0.8
4A	2/17/2023 1:00	8	13.0	13.8	0.8
4A	2/17/2023 2:00	8	13.0	13.8	0.8
4A	2/17/2023 3:00	8	13.0	13.8	0.8
4A	2/17/2023 4:00	8	13.0	13.8	0.8
4A	2/17/2023 5:00	8	13.0	13.8	0.8
4A	2/17/2023 6:00	8	13.0	13.8	0.8
4A	2/17/2023 7:00	8	13.0	13.8	0.8
4A	2/17/2023 8:00	8	13.0	2.6	-10.4
4A	2/17/2023 9:00	8	13.0	2.6	-10.4
4A	2/17/2023 10:00	8	13.0	2.6	-10.4
4A	2/17/2023 11:00	8	13.0	2.6	-10.4
4A	2/17/2023 12:00	8	13.0	5.2	-7.8
4A	2/17/2023 13:00	8	13.0	5.2	-7.8
4A	2/17/2023 14:00	8	13.0	5.2	-7.8
4A	2/17/2023 15:00	8	13.0	5.2	-7.8
4A	2/17/2023 16:00	8	11.0	6.9	-4.1
4A	2/17/2023 17:00	8	11.0	6.9	-4.1
4A	2/17/2023 18:00	8	11.0	6.9	-4.1
4A	2/17/2023 19:00	8	11.0	6.9	-4.1
4A	2/17/2023 20:00	8	11.0	16.4	5.4
4A	2/17/2023 21:00	8	11.0	13.8	2.8
4A	2/17/2023 22:00	8	11.0	13.8	2.8
4A	2/17/2023 23:00	8	11.0	13.8	2.8
4A	2/18/2023 0:00	8	11.0	13.8	2.8
4A	2/18/2023 1:00	8	11.0	13.8	2.8
4A	2/18/2023 2:00	8	11.0	13.8	2.8
4A	2/18/2023 3:00	8	11.0	13.8	2.8
4A	2/18/2023 4:00	8	11.0	13.8	2.8
4A	2/18/2023 5:00	8	11.0	13.8	2.8
4A	2/18/2023 6:00	8	11.0	13.8	2.8
4A	2/18/2023 7:00	8	11.0	13.8	2.8
4A	2/18/2023 8:00	8	11.0	10.4	-0.7
4A	2/18/2023 9:00	8	11.0	10.4	-0.7
4A	2/18/2023 10:00	8	11.0	10.4	-0.7
4A	2/18/2023 11:00	8	11.0	10.4	-0.7

4A	2/18/2023 12:00	8	11.0	15.5	4.5
4A	2/18/2023 13:00	8	11.0	15.5	4.5
4A	2/18/2023 14:00	8	11.0	15.5	4.5
4A	2/18/2023 15:00	8	11.0	15.5	4.5
4A	2/18/2023 16:00	8	11.0	18.0	7.0
4A	2/18/2023 17:00	8	11.0	18.0	7.0
4A	2/18/2023 18:00	8	11.0	18.0	7.0
4A	2/18/2023 19:00	8	11.0	18.0	7.0
4A	2/18/2023 20:00	8	11.0	13.8	2.8
4A	2/18/2023 21:00	8	11.0	13.8	2.8
4A	2/18/2023 22:00	8	11.0	13.8	2.8
4A	2/18/2023 23:00	8	11.0	13.8	2.8
4A	2/19/2023 0:00	8	11.0	6.9	-4.1
4A	2/19/2023 1:00	8	11.0	6.9	-4.1
4A	2/19/2023 2:00	8	11.0	6.9	-4.1
4A	2/19/2023 3:00	8	11.0	6.9	-4.1
4A	2/19/2023 4:00	8	11.0	6.9	-4.1
4A	2/19/2023 5:00	8	11.0	6.9	-4.1
4A	2/19/2023 6:00	8	11.0	6.9	-4.1
4A	2/19/2023 7:00	8	11.0	6.9	-4.1
4A	2/19/2023 8:00	8	11.0	7.8	-3.2
4A	2/19/2023 9:00	8	11.0	7.8	-3.2
4A	2/19/2023 10:00	8	11.0	7.8	-3.2
4A	2/19/2023 11:00	8	11.0	7.8	-3.2
4A	2/19/2023 12:00	8	11.0	10.4	-0.7
4A	2/19/2023 13:00	8	11.0	10.4	-0.7
4A	2/19/2023 14:00	8	11.0	10.4	-0.7
4A	2/19/2023 15:00	8	11.0	10.4	-0.7
4A	2/19/2023 16:00	8	11.0	13.8	2.8
4A	2/19/2023 17:00	8	11.0	13.8	2.8
4A	2/19/2023 18:00	8	11.0	13.8	2.8
4A	2/19/2023 19:00	8	11.0	13.8	2.8
4A	2/19/2023 20:00	6	11.0	13.8	2.8
4A	2/19/2023 21:00	6	11.0	13.8	2.8
4A	2/19/2023 22:00	6	11.0	13.8	2.8
4A	2/19/2023 23:00	6	11.0	13.8	2.8
4A	2/20/2023 0:00	6	11.0	11.0	0.0
4A	2/20/2023 1:00	6	11.0	11.0	0.0
4A	2/20/2023 2:00	6	11.0	11.0	0.0
4A	2/20/2023 3:00	7	11.0	13.8	2.8
4A	2/20/2023 4:00	7	11.0	13.8	2.8
4A	2/20/2023 5:00	7	11.0	13.8	2.8
4A	2/20/2023 6:00	7	11.0	13.8	2.8
4A	2/20/2023 7:00	7	11.0	13.8	2.8
4A	2/20/2023 8:00	7	11.0	12.4	1.4
4A	2/20/2023 9:00	7	11.0	12.4	1.4
4A	2/20/2023 10:00	7	11.0	12.4	1.4

4A	2/20/2023 11:00	7	11.0	12.4	1.4
4A	2/20/2023 12:00	7	11.0	12.4	1.4
4A	2/20/2023 13:00	7	11.0	12.4	1.4
4A	2/20/2023 14:00	7	11.0	12.4	1.4
4A	2/20/2023 15:00	7	11.0	12.4	1.4
4A	2/20/2023 16:00	7	11.0	13.8	2.8
4A	2/20/2023 17:00	7	11.0	13.8	2.8
4A	2/20/2023 18:00	7	11.0	15.5	4.5
4A	2/20/2023 19:00	7	11.0	17.2	6.2
4A	2/20/2023 20:00	9	11.0	17.2	6.2
4A	2/20/2023 21:00	9	11.0	17.2	6.2
4A	2/20/2023 22:00	9	11.0	17.2	6.2
4A	2/20/2023 23:00	9	11.0	17.2	6.2
4A	2/21/2023 0:00	9	11.0	16.6	5.6
4A	2/21/2023 1:00	9	11.0	16.6	5.6
4A	2/21/2023 2:00	9	11.0	16.6	5.6
4A	2/21/2023 3:00	9	11.0	16.6	5.6
4A	2/21/2023 4:00	9	11.0	16.6	5.6
4A	2/21/2023 5:00	9	11.0	16.6	5.6
4A	2/21/2023 6:00	9	11.0	16.6	5.6
4A	2/21/2023 7:00	9	11.0	16.6	5.6
4A	2/21/2023 8:00	9	16.0	12.4	-3.6
4A	2/21/2023 9:00	9	16.0	12.4	-3.6
4A	2/21/2023 10:00	9	16.0	12.4	-3.6
4A	2/21/2023 11:00	9	16.0	12.4	-3.6
4A	2/21/2023 12:00	9	16.0	12.4	-3.6
4A	2/21/2023 13:00	9	16.0	12.4	-3.6
4A	2/21/2023 14:00	9	16.0	12.4	-3.6
4A	2/21/2023 15:00	9	16.0	11.4	-4.6
4A	2/21/2023 16:00	8	11.0	10.4	-0.7
4A	2/21/2023 17:00	8	11.0	10.4	-0.7
4A	2/21/2023 18:00	8	11.0	10.4	-0.7
4A	2/21/2023 19:00	8	11.0	10.4	-0.7
4A	2/21/2023 20:00	8	11.0	17.2	6.2
4A	2/21/2023 21:00	8	10.0	17.2	7.2
4A	2/21/2023 22:00	8	10.0	17.2	7.2
4A	2/21/2023 23:00	8	10.0	17.2	7.2
4A	2/22/2023 0:00	8	10.0	13.8	3.8
4A	2/22/2023 1:00	8	10.0	13.8	3.8
4A	2/22/2023 2:00	8	10.0	13.8	3.8
4A	2/22/2023 3:00	8	10.0	13.8	3.8
4A	2/22/2023 4:00	8	10.0	13.8	3.8
4A	2/22/2023 5:00	8	10.0	13.8	3.8
4A	2/22/2023 6:00	8	10.0	13.8	3.8
4A	2/22/2023 7:00	8	10.0	13.8	3.8
4A	2/22/2023 8:00	8	10.0	10.4	0.4
4A	2/22/2023 9:00	8	10.0	10.4	0.4

4A	2/22/2023 10:00	8	10.0	10.4	0.4
4A	2/22/2023 11:00	8	10.0	10.4	0.4
4A	2/22/2023 12:00	8	10.0	12.9	2.9
4A	2/22/2023 13:00	8	10.0	12.9	2.9
4A	2/22/2023 14:00	8	10.0	12.9	2.9
4A	2/22/2023 15:00	8	10.0	12.9	2.9
4A	2/22/2023 16:00	8	10.0	13.8	3.8
4A	2/22/2023 17:00	8	10.0	13.8	3.8
4A	2/22/2023 18:00	8	10.0	13.8	3.8
4A	2/22/2023 19:00	6	8.0	13.8	5.8
4A	2/22/2023 20:00	6	8.0	10.4	2.4
4A	2/22/2023 21:00	6	8.0	10.4	2.4
4A	2/22/2023 22:00	6	8.0	10.4	2.4
4A	2/22/2023 23:00	7	8.0	10.4	2.4
4A	2/23/2023 0:00	7	8.0	6.9	-1.1
4A	2/23/2023 1:00	7	8.0	6.9	-1.1
4A	2/23/2023 2:00	7	8.0	6.9	-1.1
4A	2/23/2023 3:00	7	8.0	6.9	-1.1
4A	2/23/2023 4:00	7	8.0	6.9	-1.1
4A	2/23/2023 5:00	7	8.0	6.9	-1.1
4A	2/23/2023 6:00	7	8.0	6.9	-1.1
4A	2/23/2023 7:00	7	8.0	6.9	-1.1
4A	2/23/2023 8:00	7	8.0	7.8	-0.2
4A	2/23/2023 9:00	7	8.0	7.8	-0.2
4A	2/23/2023 10:00	7	8.0	7.8	-0.2
4A	2/23/2023 11:00	7	8.0	7.8	-0.2
4A	2/23/2023 12:00	7	8.0	7.8	-0.2
4A	2/23/2023 13:00	7	8.0	7.8	-0.2
4A	2/23/2023 14:00	7	8.0	7.8	-0.2
4A	2/23/2023 15:00	7	8.0	7.8	-0.2
4A	2/23/2023 16:00	7	8.0	10.4	2.4
4A	2/23/2023 17:00	7	8.0	10.4	2.4
4A	2/23/2023 18:00	7	8.0	10.4	2.4
4A	2/23/2023 19:00	7	8.0	10.4	2.4
4A	2/23/2023 20:00	7	8.0	17.2	9.2
4A	2/23/2023 21:00	7	8.0	17.2	9.2
4A	2/23/2023 22:00	7	8.0	17.2	9.2
4A	2/23/2023 23:00	7	8.0	17.2	9.2
4A	2/24/2023 0:00	7	8.0	17.2	9.2
4A	2/24/2023 1:00	7	8.0	17.2	9.2
4A	2/24/2023 2:00	7	8.0	17.2	9.2
4A	2/24/2023 3:00	7	8.0	17.2	9.2
4A	2/24/2023 4:00	7	8.0	17.2	9.2
4A	2/24/2023 5:00	7	8.0	17.2	9.2
4A	2/24/2023 6:00	7	8.0	17.2	9.2
4A	2/24/2023 7:00	7	8.0	17.2	9.2
4A	2/24/2023 8:00	7	8.0	7.8	-0.2

4A	2/24/2023 9:00	7	8.0	7.8	-0.2
4A	2/24/2023 10:00	7	8.0	7.8	-0.2
4A	2/24/2023 11:00	7	8.0	7.8	-0.2
4A	2/24/2023 12:00	7	8.0	7.8	-0.2
4A	2/24/2023 13:00	7	8.0	7.8	-0.2
4A	2/24/2023 14:00	7	8.0	7.8	-0.2
4A	2/24/2023 15:00	7	8.0	7.8	-0.2
4A	2/24/2023 16:00	7	8.0	10.4	2.4
4A	2/24/2023 17:00	7	8.0	10.4	2.4
4A	2/24/2023 18:00	7	8.0	10.4	2.4
4A	2/24/2023 19:00	7	8.0	10.4	2.4
4A	2/24/2023 20:00	7	8.0	10.4	2.4
4A	2/24/2023 21:00	7	8.0	10.4	2.4
4A	2/24/2023 22:00	7	8.0	10.4	2.4
4A	2/24/2023 23:00	7	8.0	10.4	2.4
4A	2/25/2023 0:00	7	8.0	13.8	5.8
4A	2/25/2023 1:00	7	8.0	13.8	5.8
4A	2/25/2023 2:00	7	8.0	13.8	5.8
4A	2/25/2023 3:00	7	8.0	13.8	5.8
4A	2/25/2023 4:00	7	8.0	13.8	5.8
4A	2/25/2023 5:00	5	8.0	10.4	2.4
4A	2/25/2023 6:00	5	8.0	10.4	2.4
4A	2/25/2023 7:00	5	8.0	10.4	2.4
4A	2/25/2023 8:00	5	13.0	5.2	-7.8
4A	2/25/2023 9:00	5	13.0	4.1	-8.9
4A	2/25/2023 10:00	5	13.0	4.1	-8.9
4A	2/25/2023 11:00	5	13.0	4.1	-8.9
4A	2/25/2023 12:00	5	13.0	5.2	-7.8
4A	2/25/2023 13:00	5	13.0	5.2	-7.8
4A	2/25/2023 14:00	5	13.0	5.2	-7.8
4A	2/25/2023 15:00	5	13.0	5.2	-7.8
4A	2/25/2023 16:00	5	13.0	6.9	-6.1
4A	2/25/2023 17:00	5	13.0	6.9	-6.1
4A	2/25/2023 18:00	5	13.0	6.9	-6.1
4A	2/25/2023 19:00	5	13.0	6.9	-6.1
4A	2/25/2023 20:00	5	13.0	10.4	-2.7
4A	2/25/2023 21:00	5	13.0	10.4	-2.7
4A	2/25/2023 22:00	8	13.0	10.4	-2.7
4A	2/25/2023 23:00	8	13.0	10.4	-2.7
4A	2/26/2023 0:00	8	13.0	8.3	-4.7
4A	2/26/2023 1:00	8	13.0	8.3	-4.7
4A	2/26/2023 2:00	8	13.0	8.3	-4.7
4A	2/26/2023 3:00	8	13.0	8.3	-4.7
4A	2/26/2023 4:00	8	13.0	8.3	-4.7
4A	2/26/2023 5:00	8	13.0	8.3	-4.7
4A	2/26/2023 6:00	8	13.0	8.3	-4.7
4A	2/26/2023 7:00	8	13.0	8.3	-4.7

4A	2/26/2023 8:00	10	13.0	12.4	-0.6
4A	2/26/2023 9:00	10	13.0	12.4	-0.6
4A	2/26/2023 10:00	10	13.0	12.4	-0.6
4A	2/26/2023 11:00	10	13.0	12.4	-0.6
4A	2/26/2023 12:00	10	13.0	12.4	-0.6
4A	2/26/2023 13:00	10	13.0	12.4	-0.6
4A	2/26/2023 14:00	10	13.0	12.4	-0.6
4A	2/26/2023 15:00	10	13.0	12.4	-0.6
4A	2/26/2023 16:00	8	13.0	18.0	5.0
4A	2/26/2023 17:00	8	13.0	18.0	5.0
4A	2/26/2023 18:00	8	13.0	18.0	5.0
4A	2/26/2023 19:00	8	13.0	18.0	5.0
4A	2/26/2023 20:00	8	13.0	13.8	0.8
4A	2/26/2023 21:00	8	13.0	13.8	0.8
4A	2/26/2023 22:00	8	13.0	13.8	0.8
4A	2/26/2023 23:00	8	13.0	13.8	0.8
4A	2/27/2023 0:00	8	13.0	8.3	-4.7
4A	2/27/2023 1:00	8	13.0	8.3	-4.7
4A	2/27/2023 2:00	8	13.0	8.3	-4.7
4A	2/27/2023 3:00	8	13.0	8.3	-4.7
4A	2/27/2023 4:00	8	13.0	8.3	-4.7
4A	2/27/2023 5:00	8	13.0	8.3	-4.7
4A	2/27/2023 6:00	8	13.0	8.3	-4.7
4A	2/27/2023 7:00	8	13.0	9.7	-3.3
4A	2/27/2023 8:00	8	13.0	10.4	-2.7
4A	2/27/2023 9:00	8	13.0	10.4	-2.7
4A	2/27/2023 10:00	8	13.0	10.4	-2.7
4A	2/27/2023 11:00	8	13.0	10.4	-2.7
4A	2/27/2023 12:00	8	13.0	10.4	-2.7
4A	2/27/2023 13:00	8	13.0	10.4	-2.7
4A	2/27/2023 14:00	8	13.0	10.4	-2.7
4A	2/27/2023 15:00	8	13.0	10.4	-2.7
4A	2/27/2023 16:00	8	13.0	16.6	3.6
4A	2/27/2023 17:00	8	13.0	16.6	3.6
4A	2/27/2023 18:00	8	13.0	16.6	3.6
4A	2/27/2023 19:00	8	13.0	16.6	3.6
4A	2/27/2023 20:00	8	13.0	13.8	0.8
4A	2/27/2023 21:00	8	13.0	13.8	0.8
4A	2/27/2023 22:00	8	13.0	13.8	0.8
4A	2/27/2023 23:00	12	13.0	13.8	0.8
4A	2/28/2023 0:00	12	13.0	11.0	-2.0
4A	2/28/2023 1:00	12	13.0	11.0	-2.0
4A	2/28/2023 2:00	12	13.0	11.0	-2.0
4A	2/28/2023 3:00	12	13.0	11.0	-2.0
4A	2/28/2023 4:00	12	13.0	11.0	-2.0
4A	2/28/2023 5:00	12	13.0	11.0	-2.0
4A	2/28/2023 6:00	12	13.0	11.0	-2.0

4A	2/28/2023 7:00	12	13.0	12.4	-0.6
4A	2/28/2023 8:00	12	13.0	10.4	-2.7
4A	2/28/2023 9:00	12	13.0	10.4	-2.7
4A	2/28/2023 10:00	12	13.0	10.4	-2.7
4A	2/28/2023 11:00	12	13.0	10.4	-2.7
4A	2/28/2023 12:00	12	13.0	12.4	-0.6
4A	2/28/2023 13:00	12	13.0	12.4	-0.6
4A	2/28/2023 14:00	12	13.0	12.4	-0.6
4A	2/28/2023 15:00	12	13.0	11.4	-1.6
4A	2/28/2023 16:00	12	13.0	16.6	3.6
4A	2/28/2023 17:00	12	13.0	16.6	3.6
4A	2/28/2023 18:00	12	13.0	16.6	3.6
4A	2/28/2023 19:00	12	13.0	16.6	3.6
4A	2/28/2023 20:00	12	13.0	10.4	-2.7
4A	2/28/2023 21:00	12	13.0	10.4	-2.7
4A	2/28/2023 22:00	12	13.0	10.4	-2.7
4A	2/28/2023 23:00	12	13.0	10.4	-2.7
4A	3/1/2023 0:00	12	13.0	13.8	0.8
4A	3/1/2023 1:00	12	13.0	13.8	0.8
4A	3/1/2023 2:00	12	13.0	13.8	0.8
4A	3/1/2023 3:00	12	13.0	13.8	0.8
4A	3/1/2023 4:00	12	13.0	13.8	0.8
4A	3/1/2023 5:00	12	13.0	13.8	0.8
4A	3/1/2023 6:00	12	13.0	13.8	0.8
4A	3/1/2023 7:00	12	13.0	13.8	0.8
4A	3/1/2023 8:00	12	13.0	18.0	5.0
4A	3/1/2023 9:00	12	13.0	18.0	5.0
4A	3/1/2023 10:00	12	13.0	18.0	5.0
4A	3/1/2023 11:00	12	13.0	18.0	5.0
4A	3/1/2023 12:00	12	13.0	18.0	5.0
4A	3/1/2023 13:00	12	13.0	18.0	5.0
4A	3/1/2023 14:00	12	13.0	18.0	5.0
4A	3/1/2023 15:00	12	13.0	18.0	5.0
4A	3/1/2023 16:00	12	13.0	18.0	5.0
4A	3/1/2023 17:00	12	13.0	18.0	5.0
4A	3/1/2023 18:00	12	13.0	18.0	5.0
4A	3/1/2023 19:00	12	13.0	18.0	5.0
4A	3/1/2023 20:00	12	13.0	10.4	-2.7
4A	3/1/2023 21:00	12	13.0	10.4	-2.7
4A	3/1/2023 22:00	12	13.0	10.4	-2.7
4A	3/1/2023 23:00	12	13.0	10.4	-2.7
4A	3/2/2023 0:00	12	13.0	8.3	-4.7
4A	3/2/2023 1:00	9	13.0	8.3	-4.7
4A	3/2/2023 2:00	9	13.0	8.3	-4.7
4A	3/2/2023 3:00	9	13.0	8.3	-4.7
4A	3/2/2023 4:00	9	13.0	8.3	-4.7
4A	3/2/2023 5:00	9	13.0	8.3	-4.7

4A	3/2/2023 6:00	9	13.0	8.3	-4.7
4A	3/2/2023 7:00	9	13.0	8.3	-4.7
4A	3/2/2023 8:00	10	11.0	10.4	-0.7
4A	3/2/2023 9:00	10	11.0	10.4	-0.7
4A	3/2/2023 10:00	10	11.0	10.4	-0.7
4A	3/2/2023 11:00	10	11.0	10.4	-0.7
4A	3/2/2023 12:00	10	11.0	8.3	-2.7
4A	3/2/2023 13:00	10	11.0	8.3	-2.7
4A	3/2/2023 14:00	10	11.0	8.3	-2.7
4A	3/2/2023 15:00	10	11.0	8.3	-2.7
4A	3/2/2023 16:00	10	11.0	13.8	2.8
4A	3/2/2023 17:00	10	11.0	13.8	2.8
4A	3/2/2023 18:00	10	11.0	13.8	2.8
4A	3/2/2023 19:00	10	11.0	13.8	2.8
4A	3/2/2023 20:00	10	11.0	17.2	6.2
4A	3/2/2023 21:00	10	11.0	17.2	6.2
4A	3/2/2023 22:00	10	11.0	17.2	6.2
4A	3/2/2023 23:00	10	11.0	17.2	6.2
4A	3/3/2023 0:00	10	11.0	16.6	5.6
4A	3/3/2023 1:00	10	11.0	16.6	5.6
4A	3/3/2023 2:00	10	11.0	16.6	5.6
4A	3/3/2023 3:00	10	11.0	16.6	5.6
4A	3/3/2023 4:00	10	11.0	16.6	5.6
4A	3/3/2023 5:00	10	11.0	16.6	5.6
4A	3/3/2023 6:00	10	11.0	16.6	5.6
4A	3/3/2023 7:00	10	11.0	17.2	6.2
4A	3/3/2023 8:00	10	11.0	14.5	3.5
4A	3/3/2023 9:00	10	11.0	14.5	3.5
4A	3/3/2023 10:00	10	11.0	12.4	1.4
4A	3/3/2023 11:00	10	11.0	12.4	1.4
4A	3/3/2023 12:00	10	11.0	14.5	3.5
4A	3/3/2023 13:00	10	11.0	14.5	3.5
4A	3/3/2023 14:00	10	11.0	14.5	3.5
4A	3/3/2023 15:00	10	11.0	13.5	2.5
4A	3/3/2023 16:00	10	11.0	10.4	-0.7
4A	3/3/2023 17:00	10	11.0	10.4	-0.7
4A	3/3/2023 18:00	10	11.0	10.4	-0.7
4A	3/3/2023 19:00	10	11.0	10.4	-0.7
4A	3/3/2023 20:00	13	11.0	13.8	2.8
4A	3/3/2023 21:00	13	11.0	13.8	2.8
4A	3/3/2023 22:00	13	11.0	13.8	2.8
4A	3/3/2023 23:00	13	11.0	13.8	2.8
4A	3/4/2023 0:00	13	11.0	11.0	0.0
4A	3/4/2023 1:00	13	11.0	11.0	0.0
4A	3/4/2023 2:00	13	11.0	11.0	0.0
4A	3/4/2023 3:00	13	11.0	11.0	0.0
4A	3/4/2023 4:00	13	11.0	11.0	0.0

4A	3/4/2023 5:00	13	11.0	11.0	0.0
4A	3/4/2023 6:00	13	11.0	11.0	0.0
4A	3/4/2023 7:00	13	11.0	12.4	1.4
4A	3/4/2023 8:00	13	13.0	14.5	1.5
4A	3/4/2023 9:00	13	13.0	14.5	1.5
4A	3/4/2023 10:00	13	13.0	14.5	1.5
4A	3/4/2023 11:00	13	13.0	14.5	1.5
4A	3/4/2023 12:00	13	13.0	16.6	3.6
4A	3/4/2023 13:00	13	13.0	16.6	3.6
4A	3/4/2023 14:00	13	13.0	16.6	3.6
4A	3/4/2023 15:00	13	13.0	15.5	2.5
4A	3/4/2023 16:00	13	13.0	18.0	5.0
4A	3/4/2023 17:00	13	13.0	18.0	5.0
4A	3/4/2023 18:00	13	13.0	18.0	5.0
4A	3/4/2023 19:00	13	13.0	18.0	5.0
4A	3/4/2023 20:00	12	13.0	8.3	-4.7
4A	3/4/2023 21:00	12	13.0	8.3	-4.7
4A	3/4/2023 22:00	12	13.0	8.3	-4.7
4A	3/4/2023 23:00	12	13.0	8.3	-4.7
4A	3/5/2023 0:00	12	13.0	5.5	-7.5
4A	3/5/2023 1:00	12	13.0	5.5	-7.5
4A	3/5/2023 2:00	12	13.0	5.5	-7.5
4A	3/5/2023 3:00	12	13.0	5.5	-7.5
4A	3/5/2023 4:00	12	13.0	5.5	-7.5
4A	3/5/2023 5:00	12	13.0	5.5	-7.5
4A	3/5/2023 6:00	12	13.0	5.5	-7.5
4A	3/5/2023 7:00	12	13.0	6.9	-6.1
4A	3/5/2023 8:00	12	13.0	12.4	-0.6
4A	3/5/2023 9:00	12	13.0	12.4	-0.6
4A	3/5/2023 10:00	12	13.0	12.4	-0.6
4A	3/5/2023 11:00	12	13.0	12.4	-0.6
4A	3/5/2023 12:00	12	13.0	14.5	1.5
4A	3/5/2023 13:00	12	13.0	14.5	1.5
4A	3/5/2023 14:00	12	13.0	14.5	1.5
4A	3/5/2023 15:00	12	13.0	13.5	0.5
4A	3/5/2023 16:00	12	13.0	18.0	5.0
4A	3/5/2023 17:00	12	13.0	18.0	5.0
4A	3/5/2023 18:00	12	13.0	18.0	5.0
4A	3/5/2023 19:00	12	13.0	18.0	5.0
4A	3/5/2023 20:00	12	13.0	16.6	3.6
4A	3/5/2023 21:00	12	13.0	16.6	3.6
4A	3/5/2023 22:00	12	13.0	16.6	3.6
4A	3/5/2023 23:00	12	13.0	16.6	3.6
4A	3/6/2023 0:00	13	13.0	13.8	0.8
4A	3/6/2023 1:00	13	13.0	13.8	0.8
4A	3/6/2023 2:00	13	13.0	13.8	0.8
4A	3/6/2023 3:00	13	13.0	13.8	0.8

4A	3/6/2023 4:00	13	13.0	13.8	0.8
4A	3/6/2023 5:00	13	13.0	13.8	0.8
4A	3/6/2023 6:00	13	13.0	13.8	0.8
4A	3/6/2023 7:00	13	13.0	13.8	0.8
4A	3/6/2023 8:00	13	13.0	8.3	-4.7
4A	3/6/2023 9:00	13	13.0	8.3	-4.7
4A	3/6/2023 10:00	13	13.0	8.3	-4.7
4A	3/6/2023 11:00	13	13.0	8.3	-4.7
4A	3/6/2023 12:00	13	13.0	10.4	-2.7
4A	3/6/2023 13:00	13	13.0	10.4	-2.7
4A	3/6/2023 14:00	13	13.0	10.4	-2.7
4A	3/6/2023 15:00	13	13.0	10.4	-2.7
4A	3/6/2023 16:00	13	13.0	13.8	0.8
4A	3/6/2023 17:00	13	13.0	13.8	0.8
4A	3/6/2023 18:00	13	13.0	13.8	0.8
4A	3/6/2023 19:00	13	13.0	13.8	0.8
4A	3/6/2023 20:00	13	13.0	6.9	-6.1
4A	3/6/2023 21:00	13	13.0	6.9	-6.1
4A	3/6/2023 22:00	13	13.0	6.9	-6.1
4A	3/6/2023 23:00	13	13.0	6.9	-6.1
4A	3/7/2023 0:00	13	13.0	6.9	-6.1
4A	3/7/2023 1:00	13	13.0	6.9	-6.1
4A	3/7/2023 2:00	13	13.0	6.9	-6.1
4A	3/7/2023 3:00	13	13.0	6.9	-6.1
4A	3/7/2023 4:00	13	13.0	6.9	-6.1
4A	3/7/2023 5:00	13	13.0	6.9	-6.1
4A	3/7/2023 6:00	13	13.0	6.9	-6.1
4A	3/7/2023 7:00	13	13.0	6.9	-6.1
4A	3/7/2023 8:00	13	13.0	10.4	-2.7
4A	3/7/2023 9:00	13	13.0	10.4	-2.7
4A	3/7/2023 10:00	13	13.0	10.4	-2.7
4A	3/7/2023 11:00	13	13.0	10.4	-2.7
4A	3/7/2023 12:00	13	13.0	10.4	-2.7
4A	3/7/2023 13:00	13	13.0	10.4	-2.7
4A	3/7/2023 14:00	13	13.0	10.4	-2.7
4A	3/7/2023 15:00	13	13.0	10.4	-2.7
4A	3/7/2023 16:00	14	16.0	13.8	-2.2
4A	3/7/2023 17:00	14	16.0	13.8	-2.2
4A	3/7/2023 18:00	14	16.0	16.6	0.6
4A	3/7/2023 19:00	14	16.0	16.6	0.6
4A	3/7/2023 20:00	14	16.0	13.8	-2.2
4A	3/7/2023 21:00	14	16.0	13.8	-2.2
4A	3/7/2023 22:00	14	16.0	13.8	-2.2
4A	3/7/2023 23:00	14	16.0	13.8	-2.2
4A	3/8/2023 0:00	14	16.0	10.4	-5.7
4A	3/8/2023 1:00	14	16.0	10.4	-5.7
4A	3/8/2023 2:00	14	16.0	10.4	-5.7

4A	3/8/2023 3:00	14	16.0	10.4	-5.7
4A	3/8/2023 4:00	14	16.0	10.4	-5.7
4A	3/8/2023 5:00	14	16.0	10.4	-5.7
4A	3/8/2023 6:00	14	16.0	10.4	-5.7
4A	3/8/2023 7:00	14	16.0	10.4	-5.7
4A	3/8/2023 8:00	14	16.0	12.4	-3.6
4A	3/8/2023 9:00	14	16.0	12.4	-3.6
4A	3/8/2023 10:00	14	16.0	12.4	-3.6
4A	3/8/2023 11:00	14	16.0	12.4	-3.6
4A	3/8/2023 12:00	14	16.0	12.9	-3.1
4A	3/8/2023 13:00	14	16.0	12.9	-3.1
4A	3/8/2023 14:00	14	16.0	12.9	-3.1
4A	3/8/2023 15:00	14	16.0	12.9	-3.1
4A	3/8/2023 16:00	14	16.0	13.8	-2.2
4A	3/8/2023 17:00	14	16.0	13.8	-2.2
4A	3/8/2023 18:00	14	16.0	13.8	-2.2
4A	3/8/2023 19:00	14	16.0	13.8	-2.2
4A	3/8/2023 20:00	14	16.0	13.8	-2.2
4A	3/8/2023 21:00	14	16.0	13.8	-2.2
4A	3/8/2023 22:00	14	16.0	13.8	-2.2
4A	3/8/2023 23:00	14	16.0	13.8	-2.2
4A	3/9/2023 0:00	14	16.0	18.0	2.0
4A	3/9/2023 1:00	14	16.0	18.0	2.0
4A	3/9/2023 2:00	14	16.0	18.0	2.0
4A	3/9/2023 3:00	14	16.0	18.0	2.0
4A	3/9/2023 4:00	14	16.0	18.0	2.0
4A	3/9/2023 5:00	14	16.0	18.0	2.0
4A	3/9/2023 6:00	14	16.0	18.0	2.0
4A	3/9/2023 7:00	14	16.0	18.0	2.0
4A	3/9/2023 8:00	18	20.0	14.5	-5.5
4A	3/9/2023 9:00	18	20.0	14.5	-5.5
4A	3/9/2023 10:00	18	20.0	14.5	-5.5
4A	3/9/2023 11:00	18	20.0	14.5	-5.5
4A	3/9/2023 12:00	18	20.0	12.4	-7.6
4A	3/9/2023 13:00	18	20.0	12.4	-7.6
4A	3/9/2023 14:00	18	20.0	12.4	-7.6
4A	3/9/2023 15:00	18	20.0	12.4	-7.6
4A	3/9/2023 16:00	18	20.0	13.8	-6.2
4A	3/9/2023 17:00	18	20.0	13.8	-6.2
4A	3/9/2023 18:00	18	20.0	13.8	-6.2
4A	3/9/2023 19:00	18	20.0	13.8	-6.2
4A	3/9/2023 20:00	18	20.0	16.6	-3.5
4A	3/9/2023 21:00	18	20.0	16.6	-3.5
4A	3/9/2023 22:00	18	20.0	16.6	-3.5
4A	3/9/2023 23:00	18	20.0	16.6	-3.5
4A	3/10/2023 0:00	18	20.0	17.2	-2.8
4A	3/10/2023 1:00	18	20.0	17.2	-2.8

4A	3/10/2023 2:00	18	20.0	17.2	-2.8
4A	3/10/2023 3:00	18	20.0	17.2	-2.8
4A	3/10/2023 4:00	18	20.0	17.2	-2.8
4A	3/10/2023 5:00	18	20.0	17.2	-2.8
4A	3/10/2023 6:00	18	20.0	17.2	-2.8
4A	3/10/2023 7:00	18	20.0	17.2	-2.8
4A	3/10/2023 8:00	18	20.0	10.4	-9.7
4A	3/10/2023 9:00	18	20.0	10.4	-9.7
4A	3/10/2023 10:00	18	20.0	10.4	-9.7
4A	3/10/2023 11:00	18	20.0	10.4	-9.7
4A	3/10/2023 12:00	18	20.0	12.9	-7.1
4A	3/10/2023 13:00	18	20.0	12.9	-7.1
4A	3/10/2023 14:00	18	20.0	12.9	-7.1
4A	3/10/2023 15:00	18	20.0	12.9	-7.1
4A	3/10/2023 16:00	18	20.0	13.8	-6.2
4A	3/10/2023 17:00	18	20.0	13.8	-6.2
4A	3/10/2023 18:00	18	20.0	13.8	-6.2
4A	3/10/2023 19:00	18	20.0	13.8	-6.2
4A	3/10/2023 20:00	18	20.0	18.0	-2.0
4A	3/10/2023 21:00	18	20.0	18.0	-2.0
4A	3/10/2023 22:00	18	20.0	18.0	-2.0
4A	3/10/2023 23:00	18	20.0	18.0	-2.0
4A	3/11/2023 0:00	18	20.0	18.0	-2.0
4A	3/11/2023 1:00	18	20.0	18.0	-2.0
4A	3/11/2023 2:00	18	20.0	18.0	-2.0
4A	3/11/2023 3:00	18	20.0	18.0	-2.0
4A	3/11/2023 4:00	18	20.0	18.0	-2.0
4A	3/11/2023 5:00	18	20.0	18.0	-2.0
4A	3/11/2023 6:00	18	20.0	18.0	-2.0
4A	3/11/2023 7:00	18	20.0	18.0	-2.0
4A	3/11/2023 8:00	18	20.0	10.4	-9.7
4A	3/11/2023 9:00	18	20.0	10.4	-9.7
4A	3/11/2023 10:00	18	20.0	10.4	-9.7
4A	3/11/2023 11:00	18	20.0	10.4	-9.7
4A	3/11/2023 12:00	18	20.0	12.4	-7.6
4A	3/11/2023 13:00	18	20.0	12.4	-7.6
4A	3/11/2023 14:00	18	20.0	12.4	-7.6
4A	3/11/2023 15:00	18	20.0	12.4	-7.6
4A	3/11/2023 16:00	17	18.0	16.6	-1.5
4A	3/11/2023 17:00	17	18.0	16.6	-1.5
4A	3/11/2023 18:00	17	18.0	16.6	-1.5
4A	3/11/2023 19:00	17	18.0	16.6	-1.5
4A	3/11/2023 20:00	17	18.0	13.8	-4.2
4A	3/11/2023 21:00	17	18.0	13.8	-4.2
4A	3/11/2023 22:00	17	18.0	13.8	-4.2
4A	3/11/2023 23:00	17	18.0	13.8	-4.2
4A	3/12/2023 0:00	17	18.0	10.4	-7.7

4A	3/12/2023 1:00	17	18.0	10.4	-7.7
4A	3/12/2023 2:00	17	18.0	10.4	-7.7
4A	3/12/2023 3:00	17	18.0	10.4	-7.7
4A	3/12/2023 4:00	17	18.0	10.4	-7.7
4A	3/12/2023 5:00	17	18.0	10.4	-7.7
4A	3/12/2023 6:00	17	18.0	10.4	-7.7
4A	3/12/2023 7:00	17	18.0	10.4	-7.7
4A	3/12/2023 8:00	17	18.0	12.4	-5.6
4A	3/12/2023 9:00	17	22.0	12.4	-9.6
4A	3/12/2023 10:00	17	22.0	12.4	-9.6
4A	3/12/2023 11:00	17	22.0	12.4	-9.6
4A	3/12/2023 12:00	17	22.0	14.5	-7.5
4A	3/12/2023 13:00	17	22.0	14.5	-7.5
4A	3/12/2023 14:00	17	22.0	14.5	-7.5
4A	3/12/2023 15:00	17	22.0	14.5	-7.5
4A	3/12/2023 16:00	15	22.0	17.2	-4.8
4A	3/12/2023 17:00	15	22.0	17.2	-4.8
4A	3/12/2023 18:00	15	22.0	17.2	-4.8
4A	3/12/2023 19:00	15	22.0	17.2	-4.8
4A	3/12/2023 20:00	15	22.0	18.0	-4.0
4A	3/12/2023 21:00	15	22.0	18.0	-4.0
4A	3/12/2023 22:00	15	22.0	18.0	-4.0
4A	3/12/2023 23:00	15	22.0	18.0	-4.0
4A	3/13/2023 0:00	15	22.0	17.2	-4.8
4A	3/13/2023 1:00	17	22.0	17.2	-4.8
4A	3/13/2023 2:00	17	22.0	17.2	-4.8
4A	3/13/2023 3:00	17	22.0	17.2	-4.8
4A	3/13/2023 4:00	17	22.0	17.2	-4.8
4A	3/13/2023 5:00	17	22.0	17.2	-4.8
4A	3/13/2023 6:00	17	22.0	17.2	-4.8
4A	3/13/2023 7:00	17	22.0	17.2	-4.8
4A	3/13/2023 8:00	17	22.0	15.5	-6.5
4A	3/13/2023 9:00	17	22.0	15.5	-6.5
4A	3/13/2023 10:00	17	22.0	15.5	-6.5
4A	3/13/2023 11:00	17	22.0	15.5	-6.5
4A	3/13/2023 12:00	17	22.0	18.0	-4.0
4A	3/13/2023 13:00	17	22.0	18.0	-4.0
4A	3/13/2023 14:00	17	22.0	18.0	-4.0
4A	3/13/2023 15:00	17	22.0	18.0	-4.0
4A	3/13/2023 16:00	17	22.0	18.0	-4.0
4A	3/13/2023 17:00	17	22.0	18.0	-4.0
4A	3/13/2023 18:00	17	22.0	18.0	-4.0
4A	3/13/2023 19:00	17	22.0	18.0	-4.0
4A	3/13/2023 20:00	17	22.0	17.2	-4.8
4A	3/13/2023 21:00	17	22.0	17.2	-4.8
4A	3/13/2023 22:00	17	22.0	17.2	-4.8
4A	3/13/2023 23:00	17	22.0	17.2	-4.8

4A	3/14/2023 0:00	17	22.0	18.0	-4.0
4A	3/14/2023 1:00	17	22.0	18.0	-4.0
4A	3/14/2023 2:00	17	22.0	18.0	-4.0
4A	3/14/2023 3:00	17	22.0	18.0	-4.0
4A	3/14/2023 4:00	17	22.0	18.0	-4.0
4A	3/14/2023 5:00	17	22.0	18.0	-4.0
4A	3/14/2023 6:00	17	22.0	18.0	-4.0
4A	3/14/2023 7:00	17	22.0	18.0	-4.0
4A	3/14/2023 8:00	17	22.0	18.0	-4.0
4A	3/14/2023 9:00	17	22.0	18.0	-4.0
4A	3/14/2023 10:00	17	22.0	18.0	-4.0
4A	3/14/2023 11:00	17	22.0	18.0	-4.0
4A	3/14/2023 12:00	17	22.0	18.0	-4.0
4A	3/14/2023 13:00	17	22.0	18.0	-4.0
4A	3/14/2023 14:00	17	22.0	18.0	-4.0
4A	3/14/2023 15:00	17	22.0	18.0	-4.0
4A	3/14/2023 16:00	17	22.0	18.0	-4.0
4A	3/14/2023 17:00	17	22.0	18.0	-4.0
4A	3/14/2023 18:00	17	22.0	18.0	-4.0
4A	3/14/2023 19:00	17	22.0	18.0	-4.0
4A	3/14/2023 20:00	19	22.0	18.0	-4.0
4A	3/14/2023 21:00	19	22.0	18.0	-4.0
4A	3/14/2023 22:00	19	22.0	18.0	-4.0
4A	3/14/2023 23:00	19	22.0	18.0	-4.0
4A	3/15/2023 0:00	19	22.0	17.2	-4.8
4A	3/15/2023 1:00	19	22.0	17.2	-4.8
4A	3/15/2023 2:00	19	22.0	17.2	-4.8
4A	3/15/2023 3:00	19	22.0	17.2	-4.8
4A	3/15/2023 4:00	19	22.0	17.2	-4.8
4A	3/15/2023 5:00	19	22.0	17.2	-4.8
4A	3/15/2023 6:00	19	22.0	17.2	-4.8
4A	3/15/2023 7:00	19	22.0	17.2	-4.8
4A	3/15/2023 8:00	19	22.0	18.0	-4.0
4A	3/15/2023 9:00	19	22.0	18.0	-4.0
4A	3/15/2023 10:00	19	22.0	18.0	-4.0
4A	3/15/2023 11:00	19	22.0	18.0	-4.0
4A	3/15/2023 12:00	19	22.0	18.0	-4.0
4A	3/15/2023 13:00	19	22.0	18.0	-4.0
4A	3/15/2023 14:00	19	22.0	18.0	-4.0
4A	3/15/2023 15:00	19	22.0	18.0	-4.0
4A	3/15/2023 16:00	19	22.0	18.0	-4.0
4A	3/15/2023 17:00	19	22.0	18.0	-4.0
4A	3/15/2023 18:00	19	22.0	18.0	-4.0
4A	3/15/2023 19:00	19	22.0	18.0	-4.0
4A	3/15/2023 20:00	17	20.0	17.2	-2.8
4A	3/15/2023 21:00	17	20.0	17.2	-2.8
4A	3/15/2023 22:00	17	20.0	17.2	-2.8

4A	3/15/2023 23:00	17	20.0	17.2	-2.8
4A	3/16/2023 0:00	17	20.0	18.0	-2.0
4A	3/16/2023 1:00	17	20.0	18.0	-2.0
4A	3/16/2023 2:00	17	20.0	18.0	-2.0
4A	3/16/2023 3:00	17	20.0	18.0	-2.0
4A	3/16/2023 4:00	17	20.0	18.0	-2.0
4A	3/16/2023 5:00	17	20.0	18.0	-2.0
4A	3/16/2023 6:00	17	20.0	18.0	-2.0
4A	3/16/2023 7:00	17	20.0	18.0	-2.0
4A	3/16/2023 8:00	17	20.0	12.9	-7.1
4A	3/16/2023 9:00	17	20.0	12.9	-7.1
4A	3/16/2023 10:00	17	20.0	12.9	-7.1
4A	3/16/2023 11:00	17	20.0	12.9	-7.1
4A	3/16/2023 12:00	17	20.0	15.5	-4.5
4A	3/16/2023 13:00	17	20.0	15.5	-4.5
4A	3/16/2023 14:00	17	20.0	15.5	-4.5
4A	3/16/2023 15:00	17	20.0	15.5	-4.5
4A	3/16/2023 16:00	17	20.0	18.0	-2.0
4A	3/16/2023 17:00	8	17.0	18.0	1.0
4A	3/16/2023 18:00	8	17.0	18.0	1.0
4A	3/16/2023 19:00	8	17.0	18.0	1.0
4A	3/16/2023 20:00	8	17.0	18.0	1.0
4A	3/16/2023 21:00	8	17.0	18.0	1.0
4A	3/16/2023 22:00	8	17.0	18.0	1.0
4A	3/16/2023 23:00	8	17.0	18.0	1.0
4A	3/17/2023 0:00	8	17.0	17.2	0.2
4A	3/17/2023 1:00	8	17.0	17.2	0.2
4A	3/17/2023 2:00	8	17.0	17.2	0.2
4A	3/17/2023 3:00	8	17.0	17.2	0.2
4A	3/17/2023 4:00	8	17.0	17.2	0.2
4A	3/17/2023 5:00	8	17.0	17.2	0.2
4A	3/17/2023 6:00	8	17.0	17.2	0.2
4A	3/17/2023 7:00	8	17.0	17.2	0.2
4A	3/17/2023 8:00	8	17.0	12.9	-4.1
4A	3/17/2023 9:00	8	17.0	12.9	-4.1
4A	3/17/2023 10:00	8	17.0	12.9	-4.1
4A	3/17/2023 11:00	8	17.0	12.9	-4.1
4A	3/17/2023 12:00	8	17.0	12.9	-4.1
4A	3/17/2023 13:00	8	17.0	12.9	-4.1
4A	3/17/2023 14:00	8	17.0	12.9	-4.1
4A	3/17/2023 15:00	8	17.0	12.9	-4.1
4A	3/17/2023 16:00	8	17.0	10.4	-6.7
4A	3/17/2023 17:00	8	17.0	10.4	-6.7
4A	3/17/2023 18:00	8	17.0	10.4	-6.7
4A	3/17/2023 19:00	8	17.0	10.4	-6.7
4A	3/17/2023 20:00	8	17.0	17.2	0.2
4A	3/17/2023 21:00	8	17.0	17.2	0.2

4A	3/17/2023 22:00	8	17.0	17.2	0.2
4A	3/17/2023 23:00	8	17.0	17.2	0.2
4A	3/18/2023 0:00	8	17.0	17.2	0.2
4A	3/18/2023 1:00	8	17.0	17.2	0.2
4A	3/18/2023 2:00	8	17.0	17.2	0.2
4A	3/18/2023 3:00	8	17.0	17.2	0.2
4A	3/18/2023 4:00	8	17.0	17.2	0.2
4A	3/18/2023 5:00	8	17.0	17.2	0.2
4A	3/18/2023 6:00	8	17.0	17.2	0.2
4A	3/18/2023 7:00	8	17.0	17.2	0.2
4A	3/18/2023 8:00	8	17.0	18.0	1.0
4A	3/18/2023 9:00	8	17.0	18.0	1.0
4A	3/18/2023 10:00	8	17.0	18.0	1.0
4A	3/18/2023 11:00	8	17.0	18.0	1.0
4A	3/18/2023 12:00	8	17.0	18.0	1.0
4A	3/18/2023 13:00	8	17.0	18.0	1.0
4A	3/18/2023 14:00	8	17.0	18.0	1.0
4A	3/18/2023 15:00	8	17.0	18.0	1.0
4A	3/18/2023 16:00	8	17.0	18.0	1.0
4A	3/18/2023 17:00	8	17.0	18.0	1.0
4A	3/18/2023 18:00	8	17.0	18.0	1.0
4A	3/18/2023 19:00	8	17.0	18.0	1.0
4A	3/18/2023 20:00	8	17.0	18.0	1.0
4A	3/18/2023 21:00	8	17.0	18.0	1.0
4A	3/18/2023 22:00	8	17.0	18.0	1.0
4A	3/18/2023 23:00	8	17.0	18.0	1.0
4A	3/19/2023 0:00	8	17.0	17.2	0.2
4A	3/19/2023 1:00	8	17.0	17.2	0.2
4A	3/19/2023 2:00	8	17.0	17.2	0.2
4A	3/19/2023 3:00	8	17.0	17.2	0.2
4A	3/19/2023 4:00	8	17.0	17.2	0.2
4A	3/19/2023 5:00	8	17.0	17.2	0.2
4A	3/19/2023 6:00	8	17.0	17.2	0.2
4A	3/19/2023 7:00	8	17.0	17.2	0.2
4A	3/19/2023 8:00	8	17.0	18.0	1.0
4A	3/19/2023 9:00	8	17.0	18.0	1.0
4A	3/19/2023 10:00	8	17.0	18.0	1.0
4A	3/19/2023 11:00	8	17.0	18.0	1.0
4A	3/19/2023 12:00	8	17.0	18.0	1.0
4A	3/19/2023 13:00	8	17.0	18.0	1.0
4A	3/19/2023 14:00	8	17.0	18.0	1.0
4A	3/19/2023 15:00	8	17.0	18.0	1.0
4A	3/19/2023 16:00	8	17.0	18.0	1.0
4A	3/19/2023 17:00	8	17.0	18.0	1.0
4A	3/19/2023 18:00	8	17.0	18.0	1.0
4A	3/19/2023 19:00	8	17.0	18.0	1.0
4A	3/19/2023 20:00	8	17.0	18.0	1.0

4A	3/19/2023 21:00	8	17.0	18.0	1.0
4A	3/19/2023 22:00	8	17.0	18.0	1.0
4A	3/19/2023 23:00	8	17.0	18.0	1.0
4A	3/20/2023 0:00	16	17.0	13.8	-3.2
4A	3/20/2023 1:00	16	17.0	13.8	-3.2
4A	3/20/2023 2:00	16	17.0	13.8	-3.2
4A	3/20/2023 3:00	16	17.0	13.8	-3.2
4A	3/20/2023 4:00	16	17.0	13.8	-3.2
4A	3/20/2023 5:00	16	17.0	16.6	-0.5
4A	3/20/2023 6:00	16	17.0	13.8	-3.2
4A	3/20/2023 7:00	16	17.0	13.8	-3.2
4A	3/20/2023 8:00	16	17.0	7.8	-9.2
4A	3/20/2023 9:00	16	17.0	7.8	-9.2
4A	3/20/2023 10:00	16	17.0	7.8	-9.2
4A	3/20/2023 11:00	16	17.0	7.8	-9.2
4A	3/20/2023 12:00	16	17.0	12.4	-4.6
4A	3/20/2023 13:00	16	17.0	12.4	-4.6
4A	3/20/2023 14:00	16	17.0	12.4	-4.6
4A	3/20/2023 15:00	16	17.0	12.4	-4.6
4A	3/20/2023 16:00	16	17.0	17.2	0.2
4A	3/20/2023 17:00	16	17.0	17.2	0.2
4A	3/20/2023 18:00	16	17.0	17.2	0.2
4A	3/20/2023 19:00	16	17.0	17.2	0.2
4A	3/20/2023 20:00	16	17.0	18.0	1.0
4A	3/20/2023 21:00	16	17.0	18.0	1.0
4A	3/20/2023 22:00	16	17.0	18.0	1.0
4A	3/20/2023 23:00	16	17.0	18.0	1.0
4A	3/21/2023 0:00	16	17.0	16.6	-0.5
4A	3/21/2023 1:00	16	17.0	16.6	-0.5
4A	3/21/2023 2:00	16	17.0	16.6	-0.5
4A	3/21/2023 3:00	16	17.0	16.6	-0.5
4A	3/21/2023 4:00	16	17.0	16.6	-0.5
4A	3/21/2023 5:00	16	17.0	16.6	-0.5
4A	3/21/2023 6:00	16	17.0	16.6	-0.5
4A	3/21/2023 7:00	16	17.0	17.2	0.2
4A	3/21/2023 8:00	16	16.0	10.4	-5.7
4A	3/21/2023 9:00	16	16.0	10.4	-5.7
4A	3/21/2023 10:00	16	16.0	10.4	-5.7
4A	3/21/2023 11:00	16	16.0	10.4	-5.7
4A	3/21/2023 12:00	16	16.0	10.4	-5.7
4A	3/21/2023 13:00	16	16.0	10.4	-5.7
4A	3/21/2023 14:00	16	16.0	10.4	-5.7
4A	3/21/2023 15:00	16	16.0	10.4	-5.7
4A	3/21/2023 16:00	16	16.0	6.9	-9.1
4A	3/21/2023 17:00	16	16.0	6.9	-9.1
4A	3/21/2023 18:00	16	16.0	6.9	-9.1
4A	3/21/2023 19:00	16	16.0	6.9	-9.1

4A	3/21/2023 20:00	16	16.0	17.2	1.2
4A	3/21/2023 21:00	16	16.0	17.2	1.2
4A	3/21/2023 22:00	18	18.0	17.2	-0.8
4A	3/21/2023 23:00	18	18.0	17.2	-0.8
4A	3/22/2023 0:00	18	18.0	18.0	0.0
4A	3/22/2023 1:00	18	18.0	18.0	0.0
4A	3/22/2023 2:00	18	18.0	18.0	0.0
4A	3/22/2023 3:00	18	18.0	18.0	0.0
4A	3/22/2023 4:00	18	18.0	18.0	0.0
4A	3/22/2023 5:00	18	18.0	18.0	0.0
4A	3/22/2023 6:00	18	18.0	18.0	0.0
4A	3/22/2023 7:00	18	18.0	18.0	0.0
4A	3/22/2023 8:00	18	18.0	12.9	-5.1
4A	3/22/2023 9:00	18	18.0	12.9	-5.1
4A	3/22/2023 10:00	18	18.0	12.9	-5.1
4A	3/22/2023 11:00	18	18.0	9.8	-8.2
4A	3/22/2023 12:00	18	18.0	10.4	-7.7
4A	3/22/2023 13:00	18	18.0	10.4	-7.7
4A	3/22/2023 14:00	18	18.0	10.4	-7.7
4A	3/22/2023 15:00	18	18.0	10.4	-7.7
4A	3/22/2023 16:00	18	18.0	13.8	-4.2
4A	3/22/2023 17:00	18	18.0	13.8	-4.2
4A	3/22/2023 18:00	18	18.0	13.8	-4.2
4A	3/22/2023 19:00	18	18.0	13.8	-4.2
4A	3/22/2023 20:00	18	18.0	17.9	-0.1
4A	3/22/2023 21:00	18	18.0	18.0	0.0
4A	3/22/2023 22:00	18	18.0	18.0	0.0
4A	3/22/2023 23:00	18	18.0	18.0	0.0
4A	3/23/2023 0:00	18	18.0	18.0	0.0
4A	3/23/2023 1:00	18	18.0	18.0	0.0
4A	3/23/2023 2:00	18	18.0	18.0	0.0
4A	3/23/2023 3:00	19	18.0	18.0	0.0
4A	3/23/2023 4:00	19	18.0	18.0	0.0
4A	3/23/2023 5:00	19	18.0	18.0	0.0
4A	3/23/2023 6:00	19	18.0	18.0	0.0
4A	3/23/2023 7:00	19	18.0	18.0	0.0
4A	3/23/2023 8:00	19	20.0	15.5	-4.5
4A	3/23/2023 9:00	19	20.0	15.5	-4.5
4A	3/23/2023 10:00	19	20.0	15.5	-4.5
4A	3/23/2023 11:00	19	20.0	15.5	-4.5
4A	3/23/2023 12:00	19	20.0	15.5	-4.5
4A	3/23/2023 13:00	19	20.0	15.5	-4.5
4A	3/23/2023 14:00	19	20.0	15.5	-4.5
4A	3/23/2023 15:00	19	20.0	15.5	-4.5
4A	3/23/2023 16:00	19	20.0	17.2	-2.8
4A	3/23/2023 17:00	19	20.0	17.2	-2.8
4A	3/23/2023 18:00	19	20.0	17.2	-2.8

4A	3/23/2023 19:00	19	20.0	17.2	-2.8
4A	3/23/2023 20:00	16	20.0	18.0	-2.0
4A	3/23/2023 21:00	16	20.0	18.0	-2.0
4A	3/23/2023 22:00	16	20.0	18.0	-2.0
4A	3/23/2023 23:00	16	20.0	18.0	-2.0
4A	3/24/2023 0:00	16	20.0	16.6	-3.5
4A	3/24/2023 1:00	16	20.0	16.6	-3.5
4A	3/24/2023 2:00	16	20.0	16.6	-3.5
4A	3/24/2023 3:00	16	20.0	16.6	-3.5
4A	3/24/2023 4:00	16	20.0	16.6	-3.5
4A	3/24/2023 5:00	16	20.0	16.6	-3.5
4A	3/24/2023 6:00	16	20.0	16.6	-3.5
4A	3/24/2023 7:00	16	20.0	17.9	-2.1
4A	3/24/2023 8:00	16	20.0	12.4	-7.6
4A	3/24/2023 9:00	16	20.0	12.4	-7.6
4A	3/24/2023 10:00	16	20.0	12.4	-7.6
4A	3/24/2023 11:00	16	20.0	12.9	-7.1
4A	3/24/2023 12:00	16	20.0	10.4	-9.7
4A	3/24/2023 13:00	16	20.0	10.4	-9.7
4A	3/24/2023 14:00	16	20.0	10.4	-9.7
4A	3/24/2023 15:00	16	20.0	10.4	-9.7
4A	3/24/2023 16:00	16	20.0	13.8	-6.2
4A	3/24/2023 17:00	16	20.0	13.8	-6.2
4A	3/24/2023 18:00	16	20.0	13.8	-6.2
4A	3/24/2023 19:00	16	20.0	13.8	-6.2
4A	3/24/2023 20:00	14	20.0	18.0	-2.0
4A	3/24/2023 21:00	14	20.0	18.0	-2.0
4A	3/24/2023 22:00	14	20.0	18.0	-2.0
4A	3/24/2023 23:00	14	20.0	18.0	-2.0
4A	3/25/2023 0:00	14	20.0	18.0	-2.0
4A	3/25/2023 1:00	14	20.0	18.0	-2.0
4A	3/25/2023 2:00	15	20.0	18.0	-2.0
4A	3/25/2023 3:00	15	20.0	18.0	-2.0
4A	3/25/2023 4:00	15	20.0	18.0	-2.0
4A	3/25/2023 5:00	15	20.0	18.0	-2.0
4A	3/25/2023 6:00	15	20.0	18.0	-2.0
4A	3/25/2023 7:00	16	17.0	18.0	1.0
4A	3/25/2023 8:00	16	17.0	16.6	-0.5
4A	3/25/2023 9:00	16	17.0	16.6	-0.5
4A	3/25/2023 10:00	16	17.0	16.6	-0.5
4A	3/25/2023 11:00	16	17.0	16.6	-0.5
4A	3/25/2023 12:00	16	17.0	18.0	1.0
4A	3/25/2023 13:00	16	17.0	18.0	1.0
4A	3/25/2023 14:00	16	17.0	18.0	1.0
4A	3/25/2023 15:00	16	17.0	18.0	1.0
4A	3/25/2023 16:00	16	17.0	18.0	1.0
4A	3/25/2023 17:00	16	17.0	18.0	1.0

4A	3/25/2023 18:00	16	17.0	18.0	1.0
4A	3/25/2023 19:00	16	17.0	18.0	1.0
4A	3/25/2023 20:00	16	17.0	18.0	1.0
4A	3/25/2023 21:00	16	17.0	18.0	1.0
4A	3/25/2023 22:00	15	19.0	18.0	-1.0
4A	3/25/2023 23:00	15	19.0	18.0	-1.0
4A	3/26/2023 0:00	15	19.0	18.0	-1.0
4A	3/26/2023 1:00	15	19.0	18.0	-1.0
4A	3/26/2023 2:00	15	19.0	18.0	-1.0
4A	3/26/2023 3:00	15	19.0	18.0	-1.0
4A	3/26/2023 4:00	15	19.0	18.0	-1.0
4A	3/26/2023 5:00	15	19.0	18.0	-1.0
4A	3/26/2023 6:00	15	19.0	18.0	-1.0
4A	3/26/2023 7:00	15	19.0	18.0	-1.0
4A	3/26/2023 8:00	15	22.0	14.5	-7.5
4A	3/26/2023 9:00	15	22.0	14.5	-7.5
4A	3/26/2023 10:00	15	22.0	14.5	-7.5
4A	3/26/2023 11:00	15	22.0	14.5	-7.5
4A	3/26/2023 12:00	15	22.0	14.5	-7.5
4A	3/26/2023 13:00	15	22.0	14.5	-7.5
4A	3/26/2023 14:00	15	22.0	14.5	-7.5
4A	3/26/2023 15:00	15	22.0	14.5	-7.5
4A	3/26/2023 16:00	12	22.0	18.0	-4.0
4A	3/26/2023 17:00	12	22.0	18.0	-4.0
4A	3/26/2023 18:00	12	22.0	18.0	-4.0
4A	3/26/2023 19:00	12	22.0	18.0	-4.0
4A	3/26/2023 20:00	12	22.0	18.0	-4.0
4A	3/26/2023 21:00	12	22.0	18.0	-4.0
4A	3/26/2023 22:00	12	22.0	18.0	-4.0
4A	3/26/2023 23:00	12	22.0	17.2	-4.8
4A	3/27/2023 0:00	12	22.0	13.8	-8.2
4A	3/27/2023 1:00	12	22.0	16.6	-5.5
4A	3/27/2023 2:00	12	22.0	16.6	-5.5
4A	3/27/2023 3:00	12	22.0	16.6	-5.5
4A	3/27/2023 4:00	12	22.0	16.6	-5.5
4A	3/27/2023 5:00	12	22.0	16.6	-5.5
4A	3/27/2023 6:00	12	22.0	16.6	-5.5
4A	3/27/2023 7:00	12	22.0	17.2	-4.8
4A	3/27/2023 8:00	12	22.0	18.0	-4.0
4A	3/27/2023 9:00	12	22.0	18.0	-4.0
4A	3/27/2023 10:00	12	22.0	18.0	-4.0
4A	3/27/2023 11:00	12	22.0	18.0	-4.0
4A	3/27/2023 12:00	12	22.0	18.0	-4.0
4A	3/27/2023 13:00	12	22.0	18.0	-4.0
4A	3/27/2023 14:00	12	22.0	18.0	-4.0
4A	3/27/2023 15:00	12	22.0	18.0	-4.0
4A	3/27/2023 16:00	12	22.0	18.0	-4.0

4A	3/27/2023 17:00	12	22.0	18.0	-4.0
4A	3/27/2023 18:00	12	22.0	18.0	-4.0
4A	3/27/2023 19:00	12	22.0	18.0	-4.0
4A	3/27/2023 20:00	15	22.0	16.6	-5.5
4A	3/27/2023 21:00	15	22.0	16.6	-5.5
4A	3/27/2023 22:00	16	22.0	16.6	-5.5
4A	3/27/2023 23:00	16	22.0	16.6	-5.5
4A	3/28/2023 0:00	16	22.0	16.6	-5.5
4A	3/28/2023 1:00	16	22.0	16.6	-5.5
4A	3/28/2023 2:00	16	22.0	16.6	-5.5
4A	3/28/2023 3:00	16	22.0	16.6	-5.5
4A	3/28/2023 4:00	16	22.0	16.6	-5.5
4A	3/28/2023 5:00	16	22.0	16.6	-5.5
4A	3/28/2023 6:00	16	22.0	16.6	-5.5
4A	3/28/2023 7:00	16	22.0	17.9	-4.1
4A	3/28/2023 8:00	16	22.0	16.6	-5.5
4A	3/28/2023 9:00	16	22.0	16.6	-5.5
4A	3/28/2023 10:00	16	22.0	16.6	-5.5
4A	3/28/2023 11:00	16	22.0	16.6	-5.5
4A	3/28/2023 12:00	16	22.0	16.6	-5.5
4A	3/28/2023 13:00	16	22.0	16.6	-5.5
4A	3/28/2023 14:00	16	22.0	16.6	-5.5
4A	3/28/2023 15:00	16	22.0	15.5	-6.5
4A	3/28/2023 16:00	16	22.0	18.0	-4.0
4A	3/28/2023 17:00	16	22.0	18.0	-4.0
4A	3/28/2023 18:00	16	22.0	18.0	-4.0
4A	3/28/2023 19:00	16	22.0	18.0	-4.0
4A	3/28/2023 20:00	16	22.0	18.0	-4.0
4A	3/28/2023 21:00	16	22.0	18.0	-4.0
4A	3/28/2023 22:00	16	22.0	18.0	-4.0
4A	3/28/2023 23:00	16	22.0	18.0	-4.0
4A	3/29/2023 0:00	16	22.0	17.9	-4.1
4A	3/29/2023 1:00	14	22.0	18.0	-4.0
4A	3/29/2023 2:00	14	22.0	18.0	-4.0
4A	3/29/2023 3:00	14	22.0	18.0	-4.0
4A	3/29/2023 4:00	14	22.0	18.0	-4.0
4A	3/29/2023 5:00	14	22.0	18.0	-4.0
4A	3/29/2023 6:00	14	22.0	18.0	-4.0
4A	3/29/2023 7:00	14	22.0	18.0	-4.0
4A	3/29/2023 8:00	14	22.0	18.0	-4.0
4A	3/29/2023 9:00	14	22.0	18.0	-4.0
4A	3/29/2023 10:00	14	22.0	18.0	-4.0
4A	3/29/2023 11:00	14	22.0	18.0	-4.0
4A	3/29/2023 12:00	14	22.0	18.0	-4.0
4A	3/29/2023 13:00	14	22.0	18.0	-4.0
4A	3/29/2023 14:00	14	22.0	18.0	-4.0
4A	3/29/2023 15:00	14	22.0	18.0	-4.0

4A	3/29/2023 16:00	14	22.0	18.0	-4.0
4A	3/29/2023 17:00	14	22.0	18.0	-4.0
4A	3/29/2023 18:00	14	22.0	18.0	-4.0
4A	3/29/2023 19:00	14	22.0	18.0	-4.0
4A	3/29/2023 20:00	14	22.0	11.0	-11.0
4A	3/29/2023 21:00	14	22.0	11.0	-11.0
4A	3/29/2023 22:00	12	22.0	11.0	-11.0
4A	3/29/2023 23:00	12	22.0	11.0	-11.0
4A	3/30/2023 0:00	12	22.0	13.8	-8.2
4A	3/30/2023 1:00	13	22.0	13.8	-8.2
4A	3/30/2023 2:00	13	22.0	13.8	-8.2
4A	3/30/2023 3:00	13	22.0	13.8	-8.2
4A	3/30/2023 4:00	13	22.0	13.8	-8.2
4A	3/30/2023 5:00	13	22.0	13.8	-8.2
4A	3/30/2023 6:00	13	22.0	13.8	-8.2
4A	3/30/2023 7:00	13	22.0	13.8	-8.2
4A	3/30/2023 8:00	13	22.0	16.6	-5.5
4A	3/30/2023 9:00	13	22.0	16.6	-5.5
4A	3/30/2023 10:00	13	22.0	16.6	-5.5
4A	3/30/2023 11:00	13	22.0	16.6	-5.5
4A	3/30/2023 12:00	13	22.0	18.0	-4.0
4A	3/30/2023 13:00	13	22.0	18.0	-4.0
4A	3/30/2023 14:00	13	22.0	18.0	-4.0
4A	3/30/2023 15:00	13	22.0	18.0	-4.0
4A	3/30/2023 16:00	13	22.0	18.0	-4.0
4A	3/30/2023 17:00	13	22.0	18.0	-4.0
4A	3/30/2023 18:00	13	22.0	18.0	-4.0
4A	3/30/2023 19:00	13	22.0	18.0	-4.0
4A	3/30/2023 20:00	13	22.0	18.0	-4.0
4A	3/30/2023 21:00	13	22.0	18.0	-4.0
4A	3/30/2023 22:00	16	22.0	18.0	-4.0
4A	3/30/2023 23:00	16	22.0	18.0	-4.0
4A	3/31/2023 0:00	18	22.0	18.0	-4.0
4A	3/31/2023 1:00	18	22.0	18.0	-4.0
4A	3/31/2023 2:00	18	22.0	18.0	-4.0
4A	3/31/2023 3:00	18	22.0	18.0	-4.0
4A	3/31/2023 4:00	18	22.0	18.0	-4.0
4A	3/31/2023 5:00	18	22.0	18.0	-4.0
4A	3/31/2023 6:00	18	22.0	18.0	-4.0
4A	3/31/2023 7:00	18	22.0	18.0	-4.0
4A	3/31/2023 8:00	18	22.0	18.0	-4.0
4A	3/31/2023 9:00	18	22.0	18.0	-4.0
4A	3/31/2023 10:00	18	22.0	18.0	-4.0
4A	3/31/2023 11:00	18	22.0	18.0	-4.0
4A	3/31/2023 12:00	18	22.0	18.0	-4.0
4A	3/31/2023 13:00	18	22.0	18.0	-4.0
4A	3/31/2023 14:00	18	22.0	18.0	-4.0

4A	3/31/2023 15:00	18	22.0	18.0	-4.0
4A	3/31/2023 16:00	18	22.0	18.0	-4.0
4A	3/31/2023 17:00	18	22.0	18.0	-4.0
4A	3/31/2023 18:00	18	22.0	18.0	-4.0
4A	3/31/2023 19:00	18	22.0	18.0	-4.0
4A	3/31/2023 20:00	15	22.0	18.0	-4.0
4A	3/31/2023 21:00	15	22.0	18.0	-4.0
4A	3/31/2023 22:00	15	22.0	18.0	-4.0
4A	3/31/2023 23:00	15	22.0	18.0	-4.0
4A	4/1/2023 0:00	15	22.0	18.0	-4.0
4A	4/1/2023 1:00	15	22.0	18.0	-4.0
4A	4/1/2023 2:00	15	22.0	18.0	-4.0
4A	4/1/2023 3:00	15	22.0	18.0	-4.0
4A	4/1/2023 4:00	15	22.0	18.0	-4.0
4A	4/1/2023 5:00	15	22.0	18.0	-4.0
4A	4/1/2023 6:00	16	22.0	18.0	-4.0
4A	4/1/2023 7:00	16	22.0	18.0	-4.0
4A	4/1/2023 8:00	16	22.0	18.0	-4.0
4A	4/1/2023 9:00	16	22.0	18.0	-4.0
4A	4/1/2023 10:00	16	22.0	18.0	-4.0
4A	4/1/2023 11:00	16	22.0	18.0	-4.0
4A	4/1/2023 12:00	16	22.0	18.0	-4.0
4A	4/1/2023 13:00	16	22.0	18.0	-4.0
4A	4/1/2023 14:00	16	22.0	18.0	-4.0
4A	4/1/2023 15:00	16	22.0	18.0	-4.0
4A	4/1/2023 16:00	16	22.0	18.0	-4.0
4A	4/1/2023 17:00	16	22.0	18.0	-4.0
4A	4/1/2023 18:00	16	22.0	18.0	-4.0
4A	4/1/2023 19:00	16	22.0	18.0	-4.0
4A	4/1/2023 20:00	14	22.0	16.6	-5.5
4A	4/1/2023 21:00	14	22.0	16.6	-5.5
4A	4/1/2023 22:00	14	22.0	16.6	-5.5
4A	4/1/2023 23:00	14	22.0	16.6	-5.5
4A	4/2/2023 0:00	14	22.0	13.8	-8.2
4A	4/2/2023 1:00	14	22.0	13.8	-8.2
4A	4/2/2023 2:00	14	22.0	13.8	-8.2
4A	4/2/2023 3:00	14	22.0	13.8	-8.2
4A	4/2/2023 4:00	14	22.0	13.8	-8.2
4A	4/2/2023 5:00	14	22.0	13.8	-8.2
4A	4/2/2023 6:00	14	22.0	13.8	-8.2
4A	4/2/2023 7:00	14	22.0	13.8	-8.2
4A	4/2/2023 8:00	14	22.0	18.0	-4.0
4A	4/2/2023 9:00	14	22.0	18.0	-4.0
4A	4/2/2023 10:00	14	22.0	18.0	-4.0
4A	4/2/2023 11:00	14	22.0	18.0	-4.0
4A	4/2/2023 12:00	14	22.0	18.0	-4.0
4A	4/2/2023 13:00	14	22.0	18.0	-4.0

4A	4/2/2023 14:00	14	22.0	18.0	-4.0
4A	4/2/2023 15:00	14	22.0	18.0	-4.0
4A	4/2/2023 16:00	14	22.0	18.0	-4.0
4A	4/2/2023 17:00	14	22.0	18.0	-4.0
4A	4/2/2023 18:00	14	22.0	18.0	-4.0
4A	4/2/2023 19:00	14	22.0	18.0	-4.0
4A	4/2/2023 20:00	14	22.0	18.0	-4.0
4A	4/2/2023 21:00	14	22.0	18.0	-4.0
4A	4/2/2023 22:00	14	22.0	18.0	-4.0
4A	4/2/2023 23:00	14	22.0	18.0	-4.0
4A	4/3/2023 0:00	14	22.0	13.8	-8.2
4A	4/3/2023 1:00	14	22.0	13.8	-8.2
4A	4/3/2023 2:00	14	22.0	13.8	-8.2
4A	4/3/2023 3:00	14	22.0	13.8	-8.2
4A	4/3/2023 4:00	14	22.0	13.8	-8.2
4A	4/3/2023 5:00	14	22.0	13.8	-8.2
4A	4/3/2023 6:00	14	22.0	13.8	-8.2
4A	4/3/2023 7:00	14	22.0	13.8	-8.2
4A	4/3/2023 8:00	14	22.0	18.0	-4.0
4A	4/3/2023 9:00	14	22.0	18.0	-4.0
4A	4/3/2023 10:00	14	22.0	18.0	-4.0
4A	4/3/2023 11:00	14	22.0	18.0	-4.0
4A	4/3/2023 12:00	14	22.0	18.0	-4.0
4A	4/3/2023 13:00	14	22.0	18.0	-4.0
4A	4/3/2023 14:00	14	22.0	18.0	-4.0
4A	4/3/2023 15:00	14	22.0	18.0	-4.0
4A	4/3/2023 16:00	14	22.0	16.6	-5.5
4A	4/3/2023 17:00	14	22.0	16.6	-5.5
4A	4/3/2023 18:00	14	22.0	16.6	-5.5
4A	4/3/2023 19:00	14	22.0	16.6	-5.5
4A	4/3/2023 20:00	14	22.0	11.0	-11.0
4A	4/3/2023 21:00	16	22.0	11.0	-11.0
4A	4/3/2023 22:00	16	22.0	11.0	-11.0
4A	4/3/2023 23:00	16	22.0	11.0	-11.0
4A	4/4/2023 0:00	17	22.0	13.8	-8.2
4A	4/4/2023 1:00	17	22.0	11.0	-11.0
4A	4/4/2023 2:00	17	22.0	11.0	-11.0
4A	4/4/2023 3:00	17	22.0	11.0	-11.0
4A	4/4/2023 4:00	17	22.0	11.0	-11.0
4A	4/4/2023 5:00	17	22.0	11.0	-11.0
4A	4/4/2023 6:00	18	22.0	13.8	-8.2
4A	4/4/2023 7:00	18	22.0	13.8	-8.2
4A	4/4/2023 8:00	18	22.0	16.6	-5.5
4A	4/4/2023 9:00	18	22.0	16.6	-5.5
4A	4/4/2023 10:00	18	22.0	16.6	-5.5
4A	4/4/2023 11:00	18	22.0	16.6	-5.5
4A	4/4/2023 12:00	18	22.0	18.0	-4.0

4A	4/4/2023 13:00	18	22.0	18.0	-4.0
4A	4/4/2023 14:00	18	22.0	18.0	-4.0
4A	4/4/2023 15:00	18	22.0	18.0	-4.0
4A	4/4/2023 16:00	15	22.0	18.0	-4.0
4A	4/4/2023 17:00	15	22.0	18.0	-4.0
4A	4/4/2023 18:00	13	22.0	18.0	-4.0
4A	4/4/2023 19:00	13	22.0	18.0	-4.0
4A	4/4/2023 20:00	13	22.0	11.0	-11.0
4A	4/4/2023 21:00	15	22.0	13.8	-8.2
4A	4/4/2023 22:00	15	22.0	13.8	-8.2
4A	4/4/2023 23:00	15	22.0	14.5	-7.5
4A	4/5/2023 0:00	15	22.0	18.0	-4.0
4A	4/5/2023 1:00	15	22.0	18.0	-4.0
4A	4/5/2023 2:00	15	22.0	18.0	-4.0
4A	4/5/2023 3:00	17	22.0	18.0	-4.0
4A	4/5/2023 4:00	17	22.0	18.0	-4.0
4A	4/5/2023 5:00	17	22.0	18.0	-4.0
4A	4/5/2023 6:00	17	22.0	18.0	-4.0
4A	4/5/2023 7:00	17	22.0	18.0	-4.0
4A	4/5/2023 8:00	17	22.0	18.0	-4.0
4A	4/5/2023 9:00	18	22.0	18.0	-4.0
4A	4/5/2023 10:00	18	22.0	18.0	-4.0
4A	4/5/2023 11:00	18	22.0	18.0	-4.0
4A	4/5/2023 12:00	18	22.0	18.0	-4.0
4A	4/5/2023 13:00	18	22.0	18.0	-4.0
4A	4/5/2023 14:00	18	22.0	18.0	-4.0
4A	4/5/2023 15:00	15	22.0	18.0	-4.0
4A	4/5/2023 16:00	15	22.0	18.0	-4.0
4A	4/5/2023 17:00	15	22.0	18.0	-4.0
4A	4/5/2023 18:00	15	22.0	18.0	-4.0
4A	4/5/2023 19:00	15	22.0	18.0	-4.0
4A	4/5/2023 20:00	16	17.0	18.0	1.0
4A	4/5/2023 21:00	16	17.0	18.0	1.0
4A	4/5/2023 22:00	16	17.0	18.0	1.0
4A	4/5/2023 23:00	16	17.0	18.0	1.0
4A	4/6/2023 0:00	17	17.0	18.0	1.0
4A	4/6/2023 1:00	17	17.0	18.0	1.0
4A	4/6/2023 2:00	17	17.0	18.0	1.0
4A	4/6/2023 3:00	17	17.0	18.0	1.0
4A	4/6/2023 4:00	17	17.0	18.0	1.0
4A	4/6/2023 5:00	17	17.0	18.0	1.0
4A	4/6/2023 6:00	17	17.0	18.0	1.0
4A	4/6/2023 7:00	17	17.0	18.0	1.0
4A	4/6/2023 8:00	17	17.0	12.9	-4.1
4A	4/6/2023 9:00	17	17.0	12.9	-4.1
4A	4/6/2023 10:00	17	17.0	12.9	-4.1
4A	4/6/2023 11:00	17	17.0	12.9	-4.1

4A	4/6/2023 12:00	17	17.0	15.5	-1.5
4A	4/6/2023 13:00	17	17.0	15.5	-1.5
4A	4/6/2023 14:00	17	17.0	15.5	-1.5
4A	4/6/2023 15:00	17	17.0	15.5	-1.5
4A	4/6/2023 16:00	12	17.0	17.2	0.2
4A	4/6/2023 17:00	12	17.0	17.2	0.2
4A	4/6/2023 18:00	12	17.0	17.2	0.2
4A	4/6/2023 19:00	12	17.0	17.2	0.2
4A	4/6/2023 20:00	12	17.0	16.6	-0.5
4A	4/6/2023 21:00	12	17.0	16.6	-0.5
4A	4/6/2023 22:00	12	17.0	16.6	-0.5
4A	4/6/2023 23:00	12	12.0	16.6	4.6
4A	4/7/2023 0:00	15	12.0	13.8	1.8
4A	4/7/2023 1:00	15	12.0	13.8	1.8
4A	4/7/2023 2:00	15	12.0	13.8	1.8
4A	4/7/2023 3:00	15	12.0	13.8	1.8
4A	4/7/2023 4:00	15	12.0	13.8	1.8
4A	4/7/2023 5:00	15	12.0	13.8	1.8
4A	4/7/2023 6:00	15	12.0	13.8	1.8
4A	4/7/2023 7:00	15	12.0	13.8	1.8
4A	4/7/2023 8:00	15	12.0	12.9	0.9
4A	4/7/2023 9:00	15	12.0	12.9	0.9
4A	4/7/2023 10:00	15	12.0	12.9	0.9
4A	4/7/2023 11:00	15	12.0	12.9	0.9
4A	4/7/2023 12:00	15	12.0	15.5	3.5
4A	4/7/2023 13:00	15	12.0	15.5	3.5
4A	4/7/2023 14:00	15	12.0	15.5	3.5
4A	4/7/2023 15:00	15	12.0	15.5	3.5
4A	4/7/2023 16:00	15	12.0	18.0	6.0
4A	4/7/2023 17:00	15	12.0	18.0	6.0
4A	4/7/2023 18:00	15	12.0	18.0	6.0
4A	4/7/2023 19:00	15	12.0	18.0	6.0
4A	4/7/2023 20:00	15	18.0	17.2	-0.8
4A	4/7/2023 21:00	15	18.0	17.2	-0.8
4A	4/7/2023 22:00	15	18.0	17.2	-0.8
4A	4/7/2023 23:00	15	18.0	17.2	-0.8
4A	4/8/2023 0:00	15	18.0	13.8	-4.2
4A	4/8/2023 1:00	15	18.0	13.8	-4.2
4A	4/8/2023 2:00	15	18.0	13.8	-4.2
4A	4/8/2023 3:00	15	18.0	13.8	-4.2
4A	4/8/2023 4:00	15	18.0	13.8	-4.2
4A	4/8/2023 5:00	17	18.0	13.8	-4.2
4A	4/8/2023 6:00	17	18.0	13.8	-4.2
4A	4/8/2023 7:00	17	18.0	13.8	-4.2
4A	4/8/2023 8:00	17	18.0	14.5	-3.5
4A	4/8/2023 9:00	17	18.0	14.5	-3.5
4A	4/8/2023 10:00	17	18.0	14.5	-3.5

4A	4/8/2023 11:00	17	18.0	14.5	-3.5
4A	4/8/2023 12:00	17	18.0	14.5	-3.5
4A	4/8/2023 13:00	17	18.0	14.5	-3.5
4A	4/8/2023 14:00	17	18.0	14.5	-3.5
4A	4/8/2023 15:00	17	18.0	14.5	-3.5
4A	4/8/2023 16:00	17	18.0	18.0	0.0
4A	4/8/2023 17:00	17	18.0	18.0	0.0
4A	4/8/2023 18:00	17	18.0	18.0	0.0
4A	4/8/2023 19:00	17	18.0	18.0	0.0
4A	4/8/2023 20:00	17	18.0	18.0	0.0
4A	4/8/2023 21:00	15	18.0	18.0	0.0
4A	4/8/2023 22:00	15	18.0	18.0	0.0
4A	4/8/2023 23:00	15	18.0	18.0	0.0
4A	4/9/2023 0:00	15	18.0	17.2	-0.8
4A	4/9/2023 1:00	15	18.0	17.2	-0.8
4A	4/9/2023 2:00	15	18.0	17.2	-0.8
4A	4/9/2023 3:00	15	18.0	17.2	-0.8
4A	4/9/2023 4:00	15	18.0	17.2	-0.8
4A	4/9/2023 5:00	15	18.0	17.2	-0.8
4A	4/9/2023 6:00	15	18.0	17.2	-0.8
4A	4/9/2023 7:00	15	18.0	17.2	-0.8
4A	4/9/2023 8:00	15	18.0	18.0	0.0
4A	4/9/2023 9:00	15	18.0	18.0	0.0
4A	4/9/2023 10:00	15	18.0	18.0	0.0
4A	4/9/2023 11:00	15	18.0	18.0	0.0
4A	4/9/2023 12:00	15	18.0	18.0	0.0
4A	4/9/2023 13:00	15	18.0	18.0	0.0
4A	4/9/2023 14:00	15	18.0	18.0	0.0
4A	4/9/2023 15:00	15	18.0	18.0	0.0
4A	4/9/2023 16:00	15	18.0	18.0	0.0
4A	4/9/2023 17:00	15	18.0	18.0	0.0
4A	4/9/2023 18:00	15	18.0	18.0	0.0
4A	4/9/2023 19:00	15	18.0	18.0	0.0
4A	4/9/2023 20:00	15	18.0	17.2	-0.8
4A	4/9/2023 21:00	15	18.0	17.2	-0.8
4A	4/9/2023 22:00	15	18.0	17.2	-0.8
4A	4/9/2023 23:00	15	18.0	17.2	-0.8
4A	4/10/2023 0:00	16	18.0	17.2	-0.8
4A	4/10/2023 1:00	16	18.0	17.2	-0.8
4A	4/10/2023 2:00	16	18.0	17.2	-0.8
4A	4/10/2023 3:00	16	18.0	17.2	-0.8
4A	4/10/2023 4:00	16	18.0	17.2	-0.8
4A	4/10/2023 5:00	16	18.0	17.2	-0.8
4A	4/10/2023 6:00	16	18.0	17.2	-0.8
4A	4/10/2023 7:00	16	18.0	17.2	-0.8
4A	4/10/2023 8:00	16	18.0	15.5	-2.5
4A	4/10/2023 9:00	16	18.0	15.5	-2.5

4A	4/10/2023 10:00	16	18.0	15.5	-2.5
4A	4/10/2023 11:00	16	18.0	15.5	-2.5
4A	4/10/2023 12:00	16	18.0	18.0	0.0
4A	4/10/2023 13:00	16	18.0	18.0	0.0
4A	4/10/2023 14:00	16	18.0	18.0	0.0
4A	4/10/2023 15:00	16	18.0	17.6	-0.4
4A	4/10/2023 16:00	16	18.0	18.0	0.0
4A	4/10/2023 17:00	16	18.0	18.0	0.0
4A	4/10/2023 18:00	16	18.0	18.0	0.0
4A	4/10/2023 19:00	14	18.0	18.0	0.0
4A	4/10/2023 20:00	14	18.0	13.8	-4.2
4A	4/10/2023 21:00	14	18.0	13.8	-4.2
4A	4/10/2023 22:00	14	18.0	13.8	-4.2
4A	4/10/2023 23:00	16	18.0	13.8	-4.2
4A	4/11/2023 0:00	16	18.0	13.8	-4.2
4A	4/11/2023 1:00	16	18.0	13.8	-4.2
4A	4/11/2023 2:00	16	18.0	13.8	-4.2
4A	4/11/2023 3:00	16	18.0	13.8	-4.2
4A	4/11/2023 4:00	16	18.0	13.8	-4.2
4A	4/11/2023 5:00	16	18.0	13.8	-4.2
4A	4/11/2023 6:00	16	18.0	13.8	-4.2
4A	4/11/2023 7:00	16	18.0	13.8	-4.2
4A	4/11/2023 8:00	16	18.0	18.0	0.0
4A	4/11/2023 9:00	18	18.0	18.0	0.0
4A	4/11/2023 10:00	18	18.0	18.0	0.0
4A	4/11/2023 11:00	18	18.0	18.0	0.0
4A	4/11/2023 12:00	18	18.0	18.0	0.0
4A	4/11/2023 13:00	18	18.0	18.0	0.0
4A	4/11/2023 14:00	18	18.0	18.0	0.0
4A	4/11/2023 15:00	18	18.0	18.0	0.0
4A	4/11/2023 16:00	18	18.0	18.0	0.0
4A	4/11/2023 17:00	18	18.0	18.0	0.0
4A	4/11/2023 18:00	18	18.0	18.0	0.0
4A	4/11/2023 19:00	13	16.0	18.0	2.0
4A	4/11/2023 20:00	15	16.0	13.8	-2.2
4A	4/11/2023 21:00	15	16.0	13.8	-2.2
4A	4/11/2023 22:00	15	16.0	13.8	-2.2
4A	4/11/2023 23:00	15	16.0	13.8	-2.2
4A	4/12/2023 0:00	15	16.0	13.8	-2.2
4A	4/12/2023 1:00	15	16.0	13.8	-2.2
4A	4/12/2023 2:00	15	16.0	13.8	-2.2
4A	4/12/2023 3:00	15	16.0	13.8	-2.2
4A	4/12/2023 4:00	15	16.0	13.8	-2.2
4A	4/12/2023 5:00	15	16.0	13.8	-2.2
4A	4/12/2023 6:00	15	16.0	13.8	-2.2
4A	4/12/2023 7:00	15	16.0	13.8	-2.2
4A	4/12/2023 8:00	15	18.0	15.5	-2.5

4A	4/12/2023 9:00	15	18.0	15.5	-2.5
4A	4/12/2023 10:00	15	18.0	15.5	-2.5
4A	4/12/2023 11:00	15	18.0	15.5	-2.5
4A	4/12/2023 12:00	15	18.0	15.5	-2.5
4A	4/12/2023 13:00	15	18.0	15.5	-2.5
4A	4/12/2023 14:00	15	18.0	15.5	-2.5
4A	4/12/2023 15:00	15	18.0	15.5	-2.5
4A	4/12/2023 16:00	15	18.0	18.0	0.0
4A	4/12/2023 17:00	15	18.0	18.0	0.0
4A	4/12/2023 18:00	15	18.0	18.0	0.0
4A	4/12/2023 19:00	15	18.0	18.0	0.0
4A	4/12/2023 20:00	16	18.0	18.0	0.0
4A	4/12/2023 21:00	16	18.0	18.0	0.0
4A	4/12/2023 22:00	16	18.0	18.0	0.0
4A	4/12/2023 23:00	16	18.0	18.0	0.0
4A	4/13/2023 0:00	16	17.0	18.0	1.0
4A	4/13/2023 1:00	16	17.0	18.0	1.0
4A	4/13/2023 2:00	16	17.0	18.0	1.0
4A	4/13/2023 3:00	16	17.0	18.0	1.0
4A	4/13/2023 4:00	16	17.0	18.0	1.0
4A	4/13/2023 5:00	16	17.0	18.0	1.0
4A	4/13/2023 6:00	16	17.0	18.0	1.0
4A	4/13/2023 7:00	16	17.0	18.0	1.0
4A	4/13/2023 8:00	16	18.0	12.9	-5.1
4A	4/13/2023 9:00	16	18.0	12.9	-5.1
4A	4/13/2023 10:00	16	18.0	12.9	-5.1
4A	4/13/2023 11:00	16	18.0	12.9	-5.1
4A	4/13/2023 12:00	16	18.0	12.9	-5.1
4A	4/13/2023 13:00	16	18.0	12.9	-5.1
4A	4/13/2023 14:00	16	18.0	12.9	-5.1
4A	4/13/2023 15:00	16	18.0	12.9	-5.1
4A	4/13/2023 16:00	16	18.0	13.8	-4.2
4A	4/13/2023 17:00	16	18.0	13.8	-4.2
4A	4/13/2023 18:00	13	19.0	13.8	-5.2
4A	4/13/2023 19:00	13	19.0	13.8	-5.2
4A	4/13/2023 20:00	13	19.0	18.0	-1.0
4A	4/13/2023 21:00	13	19.0	18.0	-1.0
4A	4/13/2023 22:00	14	19.0	18.0	-1.0
4A	4/13/2023 23:00	14	19.0	18.0	-1.0
4A	4/14/2023 0:00	15	19.0	18.0	-1.0
4A	4/14/2023 1:00	16	19.0	18.0	-1.0
4A	4/14/2023 2:00	16	19.0	18.0	-1.0
4A	4/14/2023 3:00	16	19.0	18.0	-1.0
4A	4/14/2023 4:00	16	19.0	18.0	-1.0
4A	4/14/2023 5:00	16	19.0	18.0	-1.0
4A	4/14/2023 6:00	16	19.0	18.0	-1.0
4A	4/14/2023 7:00	16	19.0	18.0	-1.0

4A	4/14/2023 8:00	16	19.0	15.5	-3.5
4A	4/14/2023 9:00	16	19.0	15.5	-3.5
4A	4/14/2023 10:00	16	19.0	15.5	-3.5
4A	4/14/2023 11:00	16	19.0	15.5	-3.5
4A	4/14/2023 12:00	16	19.0	18.0	-1.0
4A	4/14/2023 13:00	16	19.0	18.0	-1.0
4A	4/14/2023 14:00	16	19.0	18.0	-1.0
4A	4/14/2023 15:00	16	19.0	18.0	-1.0
4A	4/14/2023 16:00	15	18.0	18.0	0.0
4A	4/14/2023 17:00	15	18.0	18.0	0.0
4A	4/14/2023 18:00	15	18.0	18.0	0.0
4A	4/14/2023 19:00	15	18.0	18.0	0.0
4A	4/14/2023 20:00	16	18.0	18.0	0.0
4A	4/14/2023 21:00	16	18.0	18.0	0.0
4A	4/14/2023 22:00	16	18.0	18.0	0.0
4A	4/14/2023 23:00	16	18.0	18.0	0.0
4A	4/15/2023 0:00	18	18.0	16.6	-1.5
4A	4/15/2023 1:00	18	18.0	16.6	-1.5
4A	4/15/2023 2:00	18	18.0	16.6	-1.5
4A	4/15/2023 3:00	18	18.0	16.6	-1.5
4A	4/15/2023 4:00	18	18.0	16.6	-1.5
4A	4/15/2023 5:00	18	18.0	16.6	-1.5
4A	4/15/2023 6:00	18	18.0	16.6	-1.5
4A	4/15/2023 7:00	18	18.0	17.9	-0.1
4A	4/15/2023 8:00	18	18.0	14.5	-3.5
4A	4/15/2023 9:00	18	18.0	14.5	-3.5
4A	4/15/2023 10:00	18	18.0	14.5	-3.5
4A	4/15/2023 11:00	18	18.0	14.5	-3.5
4A	4/15/2023 12:00	18	18.0	15.5	-2.5
4A	4/15/2023 13:00	18	18.0	15.5	-2.5
4A	4/15/2023 14:00	18	18.0	15.5	-2.5
4A	4/15/2023 15:00	18	18.0	15.5	-2.5
4A	4/15/2023 16:00	18	18.0	18.0	0.0
4A	4/15/2023 17:00	18	18.0	18.0	0.0
4A	4/15/2023 18:00	18	18.0	18.0	0.0
4A	4/15/2023 19:00	18	18.0	18.0	0.0
4A	4/15/2023 20:00	16	18.0	16.6	-1.5
4A	4/15/2023 21:00	16	18.0	16.6	-1.5
4A	4/15/2023 22:00	16	18.0	16.6	-1.5
4A	4/15/2023 23:00	16	18.0	16.6	-1.5
4A	4/16/2023 0:00	16	18.0	17.2	-0.8
4A	4/16/2023 1:00	16	18.0	17.2	-0.8
4A	4/16/2023 2:00	16	18.0	17.2	-0.8
4A	4/16/2023 3:00	16	18.0	17.2	-0.8
4A	4/16/2023 4:00	16	18.0	17.2	-0.8
4A	4/16/2023 5:00	16	18.0	17.2	-0.8
4A	4/16/2023 6:00	16	18.0	17.2	-0.8

4A	4/16/2023 7:00	16	18.0	17.2	-0.8
4A	4/16/2023 8:00	16	18.0	18.0	0.0
4A	4/16/2023 9:00	16	18.0	18.0	0.0
4A	4/16/2023 10:00	16	18.0	18.0	0.0
4A	4/16/2023 11:00	16	18.0	18.0	0.0
4A	4/16/2023 12:00	16	18.0	18.0	0.0
4A	4/16/2023 13:00	16	18.0	18.0	0.0
4A	4/16/2023 14:00	16	18.0	18.0	0.0
4A	4/16/2023 15:00	16	18.0	18.0	0.0
4A	4/16/2023 16:00	16	18.0	18.0	0.0
4A	4/16/2023 17:00	16	18.0	18.0	0.0
4A	4/16/2023 18:00	16	18.0	18.0	0.0
4A	4/16/2023 19:00	16	18.0	18.0	0.0
4A	4/16/2023 20:00	11	20.0	17.2	-2.8
4A	4/16/2023 21:00	11	20.0	17.2	-2.8
4A	4/16/2023 22:00	11	20.0	17.2	-2.8
4A	4/16/2023 23:00	11	20.0	17.2	-2.8
4A	4/17/2023 0:00	11	20.0	18.0	-2.0
4A	4/17/2023 1:00	12	20.0	18.0	-2.0
4A	4/17/2023 2:00	12	20.0	18.0	-2.0
4A	4/17/2023 3:00	12	20.0	18.0	-2.0
4A	4/17/2023 4:00	12	20.0	18.0	-2.0
4A	4/17/2023 5:00	13	20.0	18.0	-2.0
4A	4/17/2023 6:00	13	20.0	18.0	-2.0
4A	4/17/2023 7:00	13	20.0	18.0	-2.0
4A	4/17/2023 8:00	13	20.0	16.6	-3.5
4A	4/17/2023 9:00	13	20.0	16.6	-3.5
4A	4/17/2023 10:00	13	20.0	16.6	-3.5
4A	4/17/2023 11:00	13	20.0	16.6	-3.5
4A	4/17/2023 12:00	13	20.0	14.5	-5.5
4A	4/17/2023 13:00	13	20.0	14.5	-5.5
4A	4/17/2023 14:00	13	20.0	14.5	-5.5
4A	4/17/2023 15:00	13	20.0	15.5	-4.5
4A	4/17/2023 16:00	13	20.0	16.6	-3.5
4A	4/17/2023 17:00	13	20.0	16.6	-3.5
4A	4/17/2023 18:00	13	20.0	16.6	-3.5
4A	4/17/2023 19:00	13	20.0	16.6	-3.5
4A	4/17/2023 20:00	15	20.0	16.6	-3.5
4A	4/17/2023 21:00	15	20.0	16.6	-3.5
4A	4/17/2023 22:00	15	20.0	16.6	-3.5
4A	4/17/2023 23:00	15	20.0	16.6	-3.5
4A	4/18/2023 0:00	15	20.0	16.6	-3.5
4A	4/18/2023 1:00	15	20.0	16.6	-3.5
4A	4/18/2023 2:00	17	20.0	16.6	-3.5
4A	4/18/2023 3:00	17	20.0	16.6	-3.5
4A	4/18/2023 4:00	17	20.0	16.6	-3.5
4A	4/18/2023 5:00	17	20.0	16.6	-3.5

4A	4/18/2023 6:00	17	20.0	16.6	-3.5
4A	4/18/2023 7:00	17	20.0	17.2	-2.8
4A	4/18/2023 8:00	17	21.0	14.5	-6.5
4A	4/18/2023 9:00	17	20.0	14.5	-5.5
4A	4/18/2023 10:00	17	20.0	14.5	-5.5
4A	4/18/2023 11:00	17	20.0	14.5	-5.5
4A	4/18/2023 12:00	17	20.0	14.5	-5.5
4A	4/18/2023 13:00	17	20.0	14.5	-5.5
4A	4/18/2023 14:00	17	20.0	14.5	-5.5
4A	4/18/2023 15:00	17	20.0	13.5	-6.6
4A	4/18/2023 16:00	15	21.0	18.0	-3.0
4A	4/18/2023 17:00	15	21.0	18.0	-3.0
4A	4/18/2023 18:00	15	21.0	18.0	-3.0
4A	4/18/2023 19:00	15	21.0	18.0	-3.0
4A	4/18/2023 20:00	15	21.0	17.2	-3.8
4A	4/18/2023 21:00	15	21.0	17.2	-3.8
4A	4/18/2023 22:00	15	21.0	17.2	-3.8
4A	4/18/2023 23:00	15	21.0	17.2	-3.8
4A	4/19/2023 0:00	15	21.0	13.8	-7.2
4A	4/19/2023 1:00	15	21.0	13.8	-7.2
4A	4/19/2023 2:00	15	21.0	13.8	-7.2
4A	4/19/2023 3:00	15	21.0	13.8	-7.2
4A	4/19/2023 4:00	15	21.0	13.8	-7.2
4A	4/19/2023 5:00	15	21.0	13.8	-7.2
4A	4/19/2023 6:00	15	21.0	13.8	-7.2
4A	4/19/2023 7:00	17	21.0	13.8	-7.2
4A	4/19/2023 8:00	17	21.0	18.0	-3.0
4A	4/19/2023 9:00	17	21.0	18.0	-3.0
4A	4/19/2023 10:00	17	21.0	18.0	-3.0
4A	4/19/2023 11:00	17	21.0	18.0	-3.0
4A	4/19/2023 12:00	17	25.0	18.0	-7.0
4A	4/19/2023 13:00	17	25.0	18.0	-7.0
4A	4/19/2023 14:00	17	25.0	18.0	-7.0
4A	4/19/2023 15:00	17	25.0	18.0	-7.0
4A	4/19/2023 16:00	17	25.0	18.0	-7.0
4A	4/19/2023 17:00	17	25.0	18.0	-7.0
4A	4/19/2023 18:00	17	25.0	18.0	-7.0
4A	4/19/2023 19:00	17	25.0	18.0	-7.0
4A	4/19/2023 20:00	17	25.0	18.0	-7.0
4A	4/19/2023 21:00	18	25.0	18.0	-7.0
4A	4/19/2023 22:00	18	25.0	18.0	-7.0
4A	4/19/2023 23:00	18	25.0	18.0	-7.0
4A	4/20/2023 0:00	18	25.0	16.6	-8.5
4A	4/20/2023 1:00	18	25.0	16.6	-8.5
4A	4/20/2023 2:00	18	25.0	16.6	-8.5
4A	4/20/2023 3:00	18	25.0	16.6	-8.5
4A	4/20/2023 4:00	18	25.0	16.6	-8.5

4A	4/20/2023 5:00	18	25.0	16.6	-8.5
4A	4/20/2023 6:00	18	25.0	16.6	-8.5
4A	4/20/2023 7:00	18	25.0	17.9	-7.1
4A	4/20/2023 8:00	18	25.0	18.0	-7.0
4A	4/20/2023 9:00	18	25.0	18.0	-7.0
4A	4/20/2023 10:00	18	25.0	18.0	-7.0
4A	4/20/2023 11:00	18	25.0	18.0	-7.0
4A	4/20/2023 12:00	18	25.0	18.0	-7.0
4A	4/20/2023 13:00	18	25.0	18.0	-7.0
4A	4/20/2023 14:00	18	25.0	18.0	-7.0
4A	4/20/2023 15:00	18	25.0	18.0	-7.0
4A	4/20/2023 16:00	18	25.0	18.0	-7.0
4A	4/20/2023 17:00	18	25.0	18.0	-7.0
4A	4/20/2023 18:00	18	25.0	18.0	-7.0
4A	4/20/2023 19:00	18	25.0	18.0	-7.0
4A	4/20/2023 20:00	18	25.0	15.2	-9.8
4A	4/20/2023 21:00	17	25.0	17.2	-7.8
4A	4/20/2023 22:00	17	25.0	17.2	-7.8
4A	4/20/2023 23:00	17	25.0	17.2	-7.8
4A	4/21/2023 0:00	17	25.0	18.0	-7.0
4A	4/21/2023 1:00	17	25.0	18.0	-7.0
4A	4/21/2023 2:00	17	25.0	18.0	-7.0
4A	4/21/2023 3:00	17	25.0	18.0	-7.0
4A	4/21/2023 4:00	17	25.0	18.0	-7.0
4A	4/21/2023 5:00	17	25.0	18.0	-7.0
4A	4/21/2023 6:00	17	25.0	18.0	-7.0
4A	4/21/2023 7:00	17	25.0	18.0	-7.0
4A	4/21/2023 8:00	17	25.0	14.5	-10.5
4A	4/21/2023 9:00	17	25.0	14.5	-10.5
4A	4/21/2023 10:00	17	25.0	14.5	-10.5
4A	4/21/2023 11:00	17	25.0	14.5	-10.5
4A	4/21/2023 12:00	17	25.0	15.5	-9.5
4A	4/21/2023 13:00	17	25.0	15.5	-9.5
4A	4/21/2023 14:00	17	25.0	15.5	-9.5
4A	4/21/2023 15:00	17	25.0	16.2	-8.8
4A	4/21/2023 16:00	17	25.0	18.0	-7.0
4A	4/21/2023 17:00	17	25.0	18.0	-7.0
4A	4/21/2023 18:00	17	25.0	18.0	-7.0
4A	4/21/2023 19:00	17	25.0	18.0	-7.0
4A	4/21/2023 20:00	17	25.0	18.0	-7.0
4A	4/21/2023 21:00	17	25.0	18.0	-7.0
4A	4/21/2023 22:00	17	25.0	18.0	-7.0
4A	4/21/2023 23:00	17	25.0	18.0	-7.0
4A	4/22/2023 0:00	17	25.0	17.2	-7.8
4A	4/22/2023 1:00	17	25.0	17.2	-7.8
4A	4/22/2023 2:00	17	25.0	17.2	-7.8
4A	4/22/2023 3:00	18	25.0	17.2	-7.8

4A	4/22/2023 4:00	18	25.0	17.2	-7.8
4A	4/22/2023 5:00	18	25.0	17.2	-7.8
4A	4/22/2023 6:00	18	25.0	17.2	-7.8
4A	4/22/2023 7:00	18	25.0	17.2	-7.8
4A	4/22/2023 8:00	18	21.0	12.9	-8.1
4A	4/22/2023 9:00	18	21.0	12.9	-8.1
4A	4/22/2023 10:00	18	21.0	12.9	-8.1
4A	4/22/2023 11:00	18	21.0	12.9	-8.1
4A	4/22/2023 12:00	18	21.0	15.5	-5.5
4A	4/22/2023 13:00	15	21.0	15.5	-5.5
4A	4/22/2023 14:00	15	21.0	15.5	-5.5
4A	4/22/2023 15:00	15	21.0	15.5	-5.5
4A	4/22/2023 16:00	15	21.0	18.0	-3.0
4A	4/22/2023 17:00	15	21.0	18.0	-3.0
4A	4/22/2023 18:00	15	21.0	18.0	-3.0
4A	4/22/2023 19:00	15	21.0	18.0	-3.0
4A	4/22/2023 20:00	15	21.0	18.0	-3.0
4A	4/22/2023 21:00	14	21.0	18.0	-3.0
4A	4/22/2023 22:00	14	21.0	18.0	-3.0
4A	4/22/2023 23:00	14	21.0	18.0	-3.0
4A	4/23/2023 0:00	14	21.0	18.0	-3.0
4A	4/23/2023 1:00	14	21.0	18.0	-3.0
4A	4/23/2023 2:00	14	21.0	18.0	-3.0
4A	4/23/2023 3:00	14	21.0	18.0	-3.0
4A	4/23/2023 4:00	14	21.0	18.0	-3.0
4A	4/23/2023 5:00	14	21.0	18.0	-3.0
4A	4/23/2023 6:00	14	21.0	18.0	-3.0
4A	4/23/2023 7:00	14	21.0	18.0	-3.0
4A	4/23/2023 8:00	16	25.0	16.6	-8.5
4A	4/23/2023 9:00	16	25.0	16.6	-8.5
4A	4/23/2023 10:00	16	25.0	16.6	-8.5
4A	4/23/2023 11:00	16	25.0	16.6	-8.5
4A	4/23/2023 12:00	16	25.0	16.6	-8.5
4A	4/23/2023 13:00	16	25.0	16.6	-8.5
4A	4/23/2023 14:00	14	17.0	16.6	-0.5
4A	4/23/2023 15:00	14	17.0	16.6	-0.5
4A	4/23/2023 16:00	14	17.0	18.0	1.0
4A	4/23/2023 17:00	14	17.0	18.0	1.0
4A	4/23/2023 18:00	14	17.0	18.0	1.0
4A	4/23/2023 19:00	14	17.0	18.0	1.0
4A	4/23/2023 20:00	14	17.0	17.2	0.2
4A	4/23/2023 21:00	14	17.0	17.2	0.2
4A	4/23/2023 22:00	14	17.0	17.2	0.2
4A	4/23/2023 23:00	14	17.0	17.2	0.2
4A	4/24/2023 0:00	14	17.0	13.8	-3.2
4A	4/24/2023 1:00	14	17.0	13.8	-3.2
4A	4/24/2023 2:00	17	17.0	13.8	-3.2

4A	4/24/2023 3:00	17	17.0	13.8	-3.2
4A	4/24/2023 4:00	17	17.0	13.8	-3.2
4A	4/24/2023 5:00	17	17.0	13.8	-3.2
4A	4/24/2023 6:00	17	17.0	13.8	-3.2
4A	4/24/2023 7:00	17	17.0	13.8	-3.2
4A	4/24/2023 8:00	17	17.0	15.5	-1.5
4A	4/24/2023 9:00	17	17.0	15.5	-1.5
4A	4/24/2023 10:00	17	17.0	15.5	-1.5
4A	4/24/2023 11:00	17	17.0	15.5	-1.5
4A	4/24/2023 12:00	17	17.0	15.5	-1.5
4A	4/24/2023 13:00	17	17.0	15.5	-1.5
4A	4/24/2023 14:00	17	17.0	15.5	-1.5
4A	4/24/2023 15:00	17	17.0	15.5	-1.5
4A	4/24/2023 16:00	17	17.0	18.0	1.0
4A	4/24/2023 17:00	17	17.0	18.0	1.0
4A	4/24/2023 18:00	17	17.0	18.0	1.0
4A	4/24/2023 19:00	17	17.0	18.0	1.0
4A	4/24/2023 20:00	17	17.0	18.0	1.0
4A	4/24/2023 21:00	17	17.0	18.0	1.0
4A	4/24/2023 22:00	17	17.0	18.0	1.0
4A	4/24/2023 23:00	17	17.0	18.0	1.0
4A	4/25/2023 0:00	17	17.0	18.0	1.0
4A	4/25/2023 1:00	16	17.0	18.0	1.0
4A	4/25/2023 2:00	16	17.0	18.0	1.0
4A	4/25/2023 3:00	16	17.0	18.0	1.0
4A	4/25/2023 4:00	16	17.0	18.0	1.0
4A	4/25/2023 5:00	16	17.0	18.0	1.0
4A	4/25/2023 6:00	16	17.0	18.0	1.0
4A	4/25/2023 7:00	16	17.0	18.0	1.0
4A	4/25/2023 8:00	17	17.0	10.4	-6.7
4A	4/25/2023 9:00	17	17.0	10.4	-6.7
4A	4/25/2023 10:00	17	17.0	10.4	-6.7
4A	4/25/2023 11:00	17	17.0	10.4	-6.7
4A	4/25/2023 12:00	17	17.0	10.4	-6.7
4A	4/25/2023 13:00	17	17.0	10.4	-6.7
4A	4/25/2023 14:00	17	17.0	10.4	-6.7
4A	4/25/2023 15:00	17	17.0	10.4	-6.7
4A	4/25/2023 16:00	17	17.0	13.8	-3.2
4A	4/25/2023 17:00	17	17.0	13.8	-3.2
4A	4/25/2023 18:00	17	17.0	13.8	-3.2
4A	4/25/2023 19:00	17	17.0	13.8	-3.2
4A	4/25/2023 20:00	17	17.0	17.2	0.2
4A	4/25/2023 21:00	17	17.0	17.2	0.2
4A	4/25/2023 22:00	17	17.0	17.2	0.2
4A	4/25/2023 23:00	17	17.0	17.2	0.2
4A	4/26/2023 0:00	15	17.0	13.8	-3.2
4A	4/26/2023 1:00	15	17.0	13.8	-3.2

4A	4/26/2023 2:00	15	17.0	13.8	-3.2
4A	4/26/2023 3:00	15	17.0	13.8	-3.2
4A	4/26/2023 4:00	15	17.0	13.8	-3.2
4A	4/26/2023 5:00	15	17.0	13.8	-3.2
4A	4/26/2023 6:00	15	17.0	13.8	-3.2
4A	4/26/2023 7:00	15	17.0	13.8	-3.2
4A	4/26/2023 8:00	15	17.0	18.0	1.0
4A	4/26/2023 9:00	15	17.0	18.0	1.0
4A	4/26/2023 10:00	15	17.0	18.0	1.0
4A	4/26/2023 11:00	15	17.0	18.0	1.0
4A	4/26/2023 12:00	15	17.0	18.0	1.0
4A	4/26/2023 13:00	15	17.0	18.0	1.0
4A	4/26/2023 14:00	15	17.0	18.0	1.0
4A	4/26/2023 15:00	15	17.0	18.0	1.0
4A	4/26/2023 16:00	15	17.0	18.0	1.0
4A	4/26/2023 17:00	15	17.0	18.0	1.0
4A	4/26/2023 18:00	15	17.0	18.0	1.0
4A	4/26/2023 19:00	15	17.0	18.0	1.0
4A	4/26/2023 20:00	14	17.0	10.4	-6.7
4A	4/26/2023 21:00	14	17.0	10.4	-6.7
4A	4/26/2023 22:00	14	17.0	10.4	-6.7
4A	4/26/2023 23:00	14	17.0	10.4	-6.7
4A	4/27/2023 0:00	14	17.0	17.2	0.2
4A	4/27/2023 1:00	14	17.0	17.2	0.2
4A	4/27/2023 2:00	14	17.0	17.2	0.2
4A	4/27/2023 3:00	14	17.0	17.2	0.2
4A	4/27/2023 4:00	14	17.0	17.2	0.2
4A	4/27/2023 5:00	14	17.0	17.2	0.2
4A	4/27/2023 6:00	14	17.0	17.2	0.2
4A	4/27/2023 7:00	14	17.0	17.2	0.2
4A	4/27/2023 8:00	14	17.0	12.9	-4.1
4A	4/27/2023 9:00	14	17.0	12.9	-4.1
4A	4/27/2023 10:00	14	17.0	12.9	-4.1
4A	4/27/2023 11:00	14	17.0	12.9	-4.1
4A	4/27/2023 12:00	18	22.0	11.0	-11.0
4A	4/27/2023 13:00	18	22.0	10.4	-11.7
4A	4/27/2023 14:00	18	22.0	10.4	-11.7
4A	4/27/2023 15:00	18	22.0	10.4	-11.7
4A	4/27/2023 16:00	18	22.0	13.8	-8.2
4A	4/27/2023 17:00	18	22.0	13.8	-8.2
4A	4/27/2023 18:00	18	22.0	13.8	-8.2
4A	4/27/2023 19:00	18	22.0	13.8	-8.2
4A	4/27/2023 20:00	18	22.0	18.0	-4.0
4A	4/27/2023 21:00	18	22.0	18.0	-4.0
4A	4/27/2023 22:00	14	18.0	18.0	0.0
4A	4/27/2023 23:00	14	18.0	18.0	0.0
4A	4/28/2023 0:00	14	18.0	18.0	0.0

4A	4/28/2023 1:00	17	18.0	18.0	0.0
4A	4/28/2023 2:00	17	18.0	18.0	0.0
4A	4/28/2023 3:00	17	18.0	18.0	0.0
4A	4/28/2023 4:00	17	18.0	18.0	0.0
4A	4/28/2023 5:00	17	18.0	18.0	0.0
4A	4/28/2023 6:00	17	18.0	18.0	0.0
4A	4/28/2023 7:00	17	18.0	18.0	0.0
4A	4/28/2023 8:00	17	18.0	12.9	-5.1
4A	4/28/2023 9:00	17	18.0	12.9	-5.1
4A	4/28/2023 10:00	18	18.0	12.9	-5.1
4A	4/28/2023 11:00	18	18.0	12.9	-5.1
4A	4/28/2023 12:00	18	18.0	10.4	-7.7
4A	4/28/2023 13:00	18	18.0	10.4	-7.7
4A	4/28/2023 14:00	18	18.0	10.4	-7.7
4A	4/28/2023 15:00	18	18.0	10.4	-7.7
4A	4/28/2023 16:00	18	18.0	13.8	-4.2
4A	4/28/2023 17:00	18	18.0	13.8	-4.2
4A	4/28/2023 18:00	18	18.0	13.8	-4.2
4A	4/28/2023 19:00	18	18.0	13.8	-4.2
4A	4/28/2023 20:00	17	18.0	17.2	-0.8
4A	4/28/2023 21:00	17	18.0	17.2	-0.8
4A	4/28/2023 22:00	17	18.0	17.2	-0.8
4A	4/28/2023 23:00	17	18.0	17.2	-0.8
4A	4/29/2023 0:00	17	18.0	17.2	-0.8
4A	4/29/2023 1:00	17	18.0	17.2	-0.8
4A	4/29/2023 2:00	17	18.0	17.2	-0.8
4A	4/29/2023 3:00	17	18.0	17.2	-0.8
4A	4/29/2023 4:00	17	18.0	17.2	-0.8
4A	4/29/2023 5:00	17	18.0	17.2	-0.8
4A	4/29/2023 6:00	17	18.0	17.2	-0.8
4A	4/29/2023 7:00	19	21.0	13.8	-7.2
4A	4/29/2023 8:00	19	21.0	5.2	-15.8
4A	4/29/2023 9:00	19	21.0	5.2	-15.8
4A	4/29/2023 10:00	19	21.0	5.2	-15.8
4A	4/29/2023 11:00	19	21.0	5.2	-15.8
4A	4/29/2023 12:00	19	21.0	5.2	-15.8
4A	4/29/2023 13:00	19	21.0	5.2	-15.8
4A	4/29/2023 14:00	19	21.0	5.2	-15.8
4A	4/29/2023 15:00	19	21.0	5.2	-15.8
4A	4/29/2023 16:00	19	21.0	10.4	-10.7
4A	4/29/2023 17:00	19	21.0	10.4	-10.7
4A	4/29/2023 18:00	19	21.0	10.4	-10.7
4A	4/29/2023 19:00	19	21.0	10.4	-10.7
4A	4/29/2023 20:00	19	21.0	13.8	-7.2
4A	4/29/2023 21:00	20	21.0	13.8	-7.2
4A	4/29/2023 22:00	20	21.0	13.8	-7.2
4A	4/29/2023 23:00	20	21.0	13.8	-7.2

4A	4/30/2023 0:00	20	21.0	13.8	-7.2
4A	4/30/2023 1:00	20	21.0	13.8	-7.2
4A	4/30/2023 2:00	20	21.0	13.8	-7.2
4A	4/30/2023 3:00	20	21.0	13.8	-7.2
4A	4/30/2023 4:00	20	21.0	13.8	-7.2
4A	4/30/2023 5:00	20	21.0	13.8	-7.2
4A	4/30/2023 6:00	20	21.0	13.8	-7.2
4A	4/30/2023 7:00	20	21.0	13.8	-7.2
4A	4/30/2023 8:00	20	21.0	12.9	-8.1
4A	4/30/2023 9:00	20	21.0	12.9	-8.1
4A	4/30/2023 10:00	20	21.0	12.9	-8.1
4A	4/30/2023 11:00	20	21.0	12.9	-8.1
4A	4/30/2023 12:00	20	21.0	10.4	-10.7
4A	4/30/2023 13:00	20	21.0	10.4	-10.7
4A	4/30/2023 14:00	20	21.0	10.4	-10.7
4A	4/30/2023 15:00	20	21.0	10.4	-10.7
4A	4/30/2023 16:00	20	21.0	13.8	-7.2
4A	4/30/2023 17:00	20	21.0	13.8	-7.2
4A	4/30/2023 18:00	20	21.0	13.8	-7.2
4A	4/30/2023 19:00	20	21.0	13.8	-7.2
4A	4/30/2023 20:00	20	21.0	13.8	-7.2
4A	4/30/2023 21:00	20	21.0	13.8	-7.2
4A	4/30/2023 22:00	20	21.0	13.8	-7.2
4A	4/30/2023 23:00	20	21.0	13.8	-7.2
4A	5/1/2023 0:00	20	21.0	13.8	-7.2
4A	5/1/2023 1:00	20	21.0	13.8	-7.2
4A	5/1/2023 2:00	20	21.0	13.8	-7.2
4A	5/1/2023 3:00	20	21.0	13.8	-7.2
4A	5/1/2023 4:00	20	21.0	13.8	-7.2
4A	5/1/2023 5:00	20	21.0	13.8	-7.2
4A	5/1/2023 6:00	20	21.0	13.8	-7.2
4A	5/1/2023 7:00	20	21.0	13.8	-7.2
4A	5/1/2023 8:00	20	21.0	7.8	-13.2
4A	5/1/2023 9:00	20	21.0	7.8	-13.2
4A	5/1/2023 10:00	20	21.0	7.8	-13.2
4A	5/1/2023 11:00	20	21.0	7.8	-13.2
4A	5/1/2023 12:00	20	21.0	10.4	-10.7
4A	5/1/2023 13:00	20	21.0	10.4	-10.7
4A	5/1/2023 14:00	20	21.0	10.4	-10.7
4A	5/1/2023 15:00	20	21.0	10.4	-10.7
4A	5/1/2023 16:00	20	21.0	10.4	-10.7
4A	5/1/2023 17:00	20	21.0	10.4	-10.7
4A	5/1/2023 18:00	20	21.0	10.4	-10.7
4A	5/1/2023 19:00	20	21.0	10.4	-10.7
4A	5/1/2023 20:00	20	21.0	17.2	-3.8
4A	5/1/2023 21:00	20	21.0	17.2	-3.8
4A	5/1/2023 22:00	20	21.0	17.2	-3.8

4A	5/1/2023 23:00	20	21.0	17.2	-3.8
4A	5/2/2023 0:00	20	21.0	17.2	-3.8
4A	5/2/2023 1:00	20	21.0	17.2	-3.8
4A	5/2/2023 2:00	20	21.0	17.2	-3.8
4A	5/2/2023 3:00	20	21.0	17.2	-3.8
4A	5/2/2023 4:00	20	21.0	13.8	-7.2
4A	5/2/2023 5:00	20	21.0	13.8	-7.2
4A	5/2/2023 6:00	20	21.0	13.8	-7.2
4A	5/2/2023 7:00	20	21.0	13.8	-7.2
4A	5/2/2023 8:00	20	21.0	5.2	-15.8
4A	5/2/2023 9:00	20	21.0	5.2	-15.8
4A	5/2/2023 10:00	20	21.0	5.2	-15.8
4A	5/2/2023 11:00	20	21.0	5.2	-15.8
4A	5/2/2023 12:00	20	21.0	5.2	-15.8
4A	5/2/2023 13:00	20	21.0	5.2	-15.8
4A	5/2/2023 14:00	20	21.0	5.2	-15.8
4A	5/2/2023 15:00	20	21.0	5.2	-15.8
4A	5/2/2023 16:00	20	21.0	3.5	-17.6
4A	5/2/2023 17:00	20	21.0	3.5	-17.6
4A	5/2/2023 18:00	20	21.0	3.5	-17.6
4A	5/2/2023 19:00	20	21.0	3.5	-17.6
4A	5/2/2023 20:00	19	21.0	18.0	-3.0
4A	5/2/2023 21:00	20	22.0	18.0	-4.0
4A	5/2/2023 22:00	20	22.0	18.0	-4.0
4A	5/2/2023 23:00	20	22.0	18.0	-4.0
4A	5/3/2023 0:00	21	22.0	18.0	-4.0
4A	5/3/2023 1:00	21	22.0	18.0	-4.0
4A	5/3/2023 2:00	21	22.0	18.0	-4.0
4A	5/3/2023 3:00	21	22.0	18.0	-4.0
4A	5/3/2023 4:00	21	22.0	18.0	-4.0
4A	5/3/2023 5:00	21	22.0	18.0	-4.0
4A	5/3/2023 6:00	21	22.0	18.0	-4.0
4A	5/3/2023 7:00	21	22.0	18.0	-4.0
4A	5/3/2023 8:00	21	22.0	12.9	-9.1
4A	5/3/2023 9:00	21	22.0	12.9	-9.1
4A	5/3/2023 10:00	21	22.0	12.9	-9.1
4A	5/3/2023 11:00	21	22.0	12.9	-9.1
4A	5/3/2023 12:00	21	22.0	15.5	-6.5
4A	5/3/2023 13:00	21	22.0	15.5	-6.5
4A	5/3/2023 14:00	21	22.0	15.5	-6.5
4A	5/3/2023 15:00	21	22.0	15.5	-6.5
4A	5/3/2023 16:00	21	22.0	17.2	-4.8
4A	5/3/2023 17:00	21	22.0	17.2	-4.8
4A	5/3/2023 18:00	21	22.0	17.2	-4.8
4A	5/3/2023 19:00	21	22.0	17.2	-4.8
4A	5/3/2023 20:00	18	20.0	17.2	-2.8
4A	5/3/2023 21:00	18	20.0	17.2	-2.8

4A	5/3/2023 22:00	18	20.0	17.2	-2.8
4A	5/3/2023 23:00	18	20.0	17.2	-2.8
4A	5/4/2023 0:00	18	20.0	17.2	-2.8
4A	5/4/2023 1:00	18	20.0	17.2	-2.8
4A	5/4/2023 2:00	18	20.0	17.2	-2.8
4A	5/4/2023 3:00	18	20.0	17.2	-2.8
4A	5/4/2023 4:00	18	20.0	17.2	-2.8
4A	5/4/2023 5:00	18	20.0	17.2	-2.8
4A	5/4/2023 6:00	18	20.0	17.2	-2.8
4A	5/4/2023 7:00	18	21.0	17.2	-3.8
4A	5/4/2023 8:00	18	21.0	12.9	-8.1
4A	5/4/2023 9:00	18	21.0	12.9	-8.1
4A	5/4/2023 10:00	18	21.0	12.9	-8.1
4A	5/4/2023 11:00	18	21.0	12.9	-8.1
4A	5/4/2023 12:00	18	21.0	12.9	-8.1
4A	5/4/2023 13:00	18	21.0	12.9	-8.1
4A	5/4/2023 14:00	18	21.0	15.5	-5.5
4A	5/4/2023 15:00	18	21.0	15.5	-5.5
4A	5/4/2023 16:00	18	21.0	17.2	-3.8
4A	5/4/2023 17:00	18	21.0	17.2	-3.8
4A	5/4/2023 18:00	18	21.0	17.2	-3.8
4A	5/4/2023 19:00	18	21.0	17.2	-3.8
4A	5/4/2023 20:00	18	21.0	18.0	-3.0
4A	5/4/2023 21:00	21	21.0	18.0	-3.0
4A	5/4/2023 22:00	21	21.0	18.0	-3.0
4A	5/4/2023 23:00	21	21.0	18.0	-3.0
4A	5/5/2023 0:00	21	21.0	18.0	-3.0
4A	5/5/2023 1:00	22	21.0	18.0	-3.0
4A	5/5/2023 2:00	22	21.0	18.0	-3.0
4A	5/5/2023 3:00	22	21.0	18.0	-3.0
4A	5/5/2023 4:00	22	21.0	18.0	-3.0
4A	5/5/2023 5:00	22	21.0	18.0	-3.0
4A	5/5/2023 6:00	22	21.0	18.0	-3.0
4A	5/5/2023 7:00	22	21.0	18.0	-3.0
4A	5/5/2023 8:00	22	21.0	18.0	-3.0
4A	5/5/2023 9:00	22	21.0	18.0	-3.0
4A	5/5/2023 10:00	22	21.0	18.0	-3.0
4A	5/5/2023 11:00	22	21.0	18.0	-3.0
4A	5/5/2023 12:00	22	21.0	18.0	-3.0
4A	5/5/2023 13:00	22	21.0	18.0	-3.0
4A	5/5/2023 14:00	22	21.0	18.0	-3.0
4A	5/5/2023 15:00	22	21.0	18.0	-3.0
4A	5/5/2023 16:00	22	21.0	18.0	-3.0
4A	5/5/2023 17:00	22	21.0	18.0	-3.0
4A	5/5/2023 18:00	22	21.0	18.0	-3.0
4A	5/5/2023 19:00	22	21.0	18.0	-3.0
4A	5/5/2023 20:00	22	21.0	18.0	-3.0

4A	5/5/2023 21:00	22	21.0	18.0	-3.0
4A	5/5/2023 22:00	22	21.0	18.0	-3.0
4A	5/5/2023 23:00	22	21.0	18.0	-3.0
4A	5/6/2023 0:00	22	21.0	18.0	-3.0
4A	5/6/2023 1:00	23	21.0	18.0	-3.0
4A	5/6/2023 2:00	23	21.0	18.0	-3.0
4A	5/6/2023 3:00	23	21.0	18.0	-3.0
4A	5/6/2023 4:00	23	21.0	18.0	-3.0
4A	5/6/2023 5:00	23	21.0	18.0	-3.0
4A	5/6/2023 6:00	23	21.0	18.0	-3.0
4A	5/6/2023 7:00	23	21.0	18.0	-3.0
4A	5/6/2023 8:00	23	21.0	14.5	-6.5
4A	5/6/2023 9:00	23	21.0	14.5	-6.5
4A	5/6/2023 10:00	23	21.0	14.5	-6.5
4A	5/6/2023 11:00	23	21.0	14.5	-6.5
4A	5/6/2023 12:00	23	21.0	16.6	-4.5
4A	5/6/2023 13:00	23	21.0	16.6	-4.5
4A	5/6/2023 14:00	23	21.0	16.6	-4.5
4A	5/6/2023 15:00	23	21.0	16.6	-4.5
4A	5/6/2023 16:00	24	25.0	18.0	-7.0
4A	5/6/2023 17:00	24	25.0	18.0	-7.0
4A	5/6/2023 18:00	24	25.0	18.0	-7.0
4A	5/6/2023 19:00	24	25.0	18.0	-7.0
4A	5/6/2023 20:00	24	25.0	18.0	-7.0
4A	5/6/2023 21:00	24	25.0	18.0	-7.0
4A	5/6/2023 22:00	24	25.0	18.0	-7.0
4A	5/6/2023 23:00	24	25.0	18.0	-7.0
4A	5/7/2023 0:00	24	25.0	18.0	-7.0
4A	5/7/2023 1:00	24	25.0	18.0	-7.0
4A	5/7/2023 2:00	24	25.0	18.0	-7.0
4A	5/7/2023 3:00	24	25.0	18.0	-7.0
4A	5/7/2023 4:00	24	25.0	18.0	-7.0
4A	5/7/2023 5:00	24	25.0	18.0	-7.0
4A	5/7/2023 6:00	24	25.0	18.0	-7.0
4A	5/7/2023 7:00	24	25.0	18.0	-7.0
4A	5/7/2023 8:00	24	25.0	18.0	-7.0
4A	5/7/2023 9:00	24	25.0	18.0	-7.0
4A	5/7/2023 10:00	24	25.0	18.0	-7.0
4A	5/7/2023 11:00	24	25.0	18.0	-7.0
4A	5/7/2023 12:00	24	25.0	18.0	-7.0
4A	5/7/2023 13:00	24	25.0	18.0	-7.0
4A	5/7/2023 14:00	24	25.0	18.0	-7.0
4A	5/7/2023 15:00	24	25.0	18.0	-7.0
4A	5/7/2023 16:00	24	25.0	18.0	-7.0
4A	5/7/2023 17:00	24	25.0	18.0	-7.0
4A	5/7/2023 18:00	24	25.0	18.0	-7.0
4A	5/7/2023 19:00	24	25.0	18.0	-7.0

4A	5/7/2023 20:00	24	25.0	18.0	-7.0
4A	5/7/2023 21:00	24	25.0	18.0	-7.0
4A	5/7/2023 22:00	24	25.0	18.0	-7.0
4A	5/7/2023 23:00	24	25.0	18.0	-7.0
4A	5/8/2023 0:00	24	25.0	17.2	-7.8
4A	5/8/2023 1:00	24	25.0	17.2	-7.8
4A	5/8/2023 2:00	24	25.0	17.2	-7.8
4A	5/8/2023 3:00	24	25.0	17.2	-7.8
4A	5/8/2023 4:00	24	25.0	17.2	-7.8
4A	5/8/2023 5:00	24	25.0	17.2	-7.8
4A	5/8/2023 6:00	24	25.0	17.2	-7.8
4A	5/8/2023 7:00	24	25.0	17.2	-7.8
4A	5/8/2023 8:00	24	26.0	18.0	-8.0
4A	5/8/2023 9:00	24	26.0	18.0	-8.0
4A	5/8/2023 10:00	24	26.0	18.0	-8.0
4A	5/8/2023 11:00	24	26.0	18.0	-8.0
4A	5/8/2023 12:00	24	26.0	18.0	-8.0
4A	5/8/2023 13:00	24	26.0	18.0	-8.0
4A	5/8/2023 14:00	24	26.0	18.0	-8.0
4A	5/8/2023 15:00	24	26.0	18.0	-8.0
4A	5/8/2023 16:00	24	26.0	18.0	-8.0
4A	5/8/2023 17:00	24	26.0	18.0	-8.0
4A	5/8/2023 18:00	24	26.0	18.0	-8.0
4A	5/8/2023 19:00	24	26.0	18.0	-8.0
4A	5/8/2023 20:00	24	26.0	17.2	-8.8
4A	5/8/2023 21:00	24	26.0	17.2	-8.8
4A	5/8/2023 22:00	24	26.0	17.2	-8.8
4A	5/8/2023 23:00	24	26.0	17.2	-8.8
4A	5/9/2023 0:00	24	26.0	18.0	-8.0
4A	5/9/2023 1:00	21	26.0	18.0	-8.0
4A	5/9/2023 2:00	21	26.0	18.0	-8.0
4A	5/9/2023 3:00	21	26.0	18.0	-8.0
4A	5/9/2023 4:00	21	26.0	18.0	-8.0
4A	5/9/2023 5:00	21	26.0	18.0	-8.0
4A	5/9/2023 6:00	21	26.0	18.0	-8.0
4A	5/9/2023 7:00	21	26.0	18.0	-8.0
4A	5/9/2023 8:00	21	26.0	12.9	-13.1
4A	5/9/2023 9:00	21	26.0	12.9	-13.1
4A	5/9/2023 10:00	21	26.0	12.9	-13.1
4A	5/9/2023 11:00	21	26.0	12.9	-13.1
4A	5/9/2023 12:00	21	26.0	12.9	-13.1
4A	5/9/2023 13:00	21	26.0	12.9	-13.1
4A	5/9/2023 14:00	21	26.0	12.9	-13.1
4A	5/9/2023 15:00	21	26.0	12.9	-13.1
4A	5/9/2023 16:00	21	26.0	13.8	-12.2
4A	5/9/2023 17:00	21	26.0	13.8	-12.2
4A	5/9/2023 18:00	21	26.0	13.8	-12.2

4A	5/9/2023 19:00	21	26.0	13.8	-12.2
4A	5/9/2023 20:00	21	26.0	17.2	-8.8
4A	5/9/2023 21:00	21	26.0	17.2	-8.8
4A	5/9/2023 22:00	21	26.0	17.2	-8.8
4A	5/9/2023 23:00	21	26.0	17.2	-8.8
4A	5/10/2023 0:00	19	21.0	10.4	-10.7
4A	5/10/2023 1:00	19	21.0	10.4	-10.7
4A	5/10/2023 2:00	19	21.0	10.4	-10.7
4A	5/10/2023 3:00	19	21.0	10.4	-10.7
4A	5/10/2023 4:00	19	21.0	10.4	-10.7
4A	5/10/2023 5:00	19	21.0	10.4	-10.7
4A	5/10/2023 6:00	19	21.0	10.4	-10.7
4A	5/10/2023 7:00	19	21.0	10.4	-10.7
4A	5/10/2023 8:00	19	21.0	15.5	-5.5
4A	5/10/2023 9:00	19	21.0	15.5	-5.5
4A	5/10/2023 10:00	19	21.0	15.5	-5.5
4A	5/10/2023 11:00	19	21.0	15.5	-5.5
4A	5/10/2023 12:00	19	21.0	18.0	-3.0
4A	5/10/2023 13:00	19	21.0	18.0	-3.0
4A	5/10/2023 14:00	19	21.0	18.0	-3.0
4A	5/10/2023 15:00	19	21.0	18.0	-3.0
4A	5/10/2023 16:00	19	21.0	17.2	-3.8
4A	5/10/2023 17:00	19	21.0	17.2	-3.8
4A	5/10/2023 18:00	19	21.0	17.2	-3.8
4A	5/10/2023 19:00	19	21.0	17.2	-3.8
4A	5/10/2023 20:00	19	21.0	17.2	-3.8
4A	5/10/2023 21:00	19	21.0	17.2	-3.8
4A	5/10/2023 22:00	19	21.0	17.2	-3.8
4A	5/10/2023 23:00	19	21.0	17.2	-3.8
4A	5/11/2023 0:00	19	21.0	17.2	-3.8
4A	5/11/2023 1:00	19	21.0	17.2	-3.8
4A	5/11/2023 2:00	19	21.0	17.2	-3.8
4A	5/11/2023 3:00	19	21.0	17.2	-3.8
4A	5/11/2023 4:00	19	21.0	17.2	-3.8
4A	5/11/2023 5:00	19	21.0	17.2	-3.8
4A	5/11/2023 6:00	22	23.0	17.2	-5.8
4A	5/11/2023 7:00	22	23.0	17.2	-5.8
4A	5/11/2023 8:00	21	22.0	10.4	-11.7
4A	5/11/2023 9:00	21	22.0	10.4	-11.7
4A	5/11/2023 10:00	21	22.0	10.4	-11.7
4A	5/11/2023 11:00	21	22.0	10.4	-11.7
4A	5/11/2023 12:00	21	22.0	10.4	-11.7
4A	5/11/2023 13:00	22	22.0	12.4	-9.6
4A	5/11/2023 14:00	22	22.0	12.4	-9.6
4A	5/11/2023 15:00	22	22.0	12.4	-9.6
4A	5/11/2023 16:00	22	22.0	17.2	-4.8
4A	5/11/2023 17:00	22	22.0	17.2	-4.8

4A	5/11/2023 18:00	22	22.0	17.2	-4.8
4A	5/11/2023 19:00	22	22.0	17.2	-4.8
4A	5/11/2023 20:00	22	22.0	17.2	-4.8
4A	5/11/2023 21:00	22	22.0	17.2	-4.8
4A	5/11/2023 22:00	22	22.0	17.2	-4.8
4A	5/11/2023 23:00	22	22.0	17.2	-4.8
4A	5/12/2023 0:00	22	22.0	10.4	-11.7
4A	5/12/2023 1:00	22	22.0	10.4	-11.7
4A	5/12/2023 2:00	21	22.0	10.4	-11.7
4A	5/12/2023 3:00	21	22.0	10.4	-11.7
4A	5/12/2023 4:00	21	22.0	10.4	-11.7
4A	5/12/2023 5:00	21	22.0	10.4	-11.7
4A	5/12/2023 6:00	21	22.0	10.4	-11.7
4A	5/12/2023 7:00	21	22.0	10.4	-11.7
4A	5/12/2023 8:00	21	22.0	18.0	-4.0
4A	5/12/2023 9:00	21	22.0	18.0	-4.0
4A	5/12/2023 10:00	21	22.0	18.0	-4.0
4A	5/12/2023 11:00	21	22.0	18.0	-4.0
4A	5/12/2023 12:00	21	22.0	18.0	-4.0
4A	5/12/2023 13:00	21	22.0	18.0	-4.0
4A	5/12/2023 14:00	21	22.0	18.0	-4.0
4A	5/12/2023 15:00	21	22.0	18.0	-4.0
4A	5/12/2023 16:00	21	22.0	18.0	-4.0
4A	5/12/2023 17:00	21	22.0	18.0	-4.0
4A	5/12/2023 18:00	21	22.0	18.0	-4.0
4A	5/12/2023 19:00	21	22.0	18.0	-4.0
4A	5/12/2023 20:00	21	22.0	18.0	-4.0
4A	5/12/2023 21:00	21	22.0	18.0	-4.0
4A	5/12/2023 22:00	21	22.0	18.0	-4.0
4A	5/12/2023 23:00	21	22.0	18.0	-4.0
4A	5/13/2023 0:00	21	22.0	18.0	-4.0
4A	5/13/2023 1:00	23	22.0	18.0	-4.0
4A	5/13/2023 2:00	23	22.0	18.0	-4.0
4A	5/13/2023 3:00	23	22.0	18.0	-4.0
4A	5/13/2023 4:00	23	22.0	18.0	-4.0
4A	5/13/2023 5:00	23	22.0	18.0	-4.0
4A	5/13/2023 6:00	23	22.0	18.0	-4.0
4A	5/13/2023 7:00	23	22.0	18.0	-4.0
4A	5/13/2023 8:00	23	22.0	12.9	-9.1
4A	5/13/2023 9:00	23	22.0	12.9	-9.1
4A	5/13/2023 10:00	23	22.0	12.9	-9.1
4A	5/13/2023 11:00	23	22.0	12.9	-9.1
4A	5/13/2023 12:00	23	22.0	15.5	-6.5
4A	5/13/2023 13:00	23	22.0	15.5	-6.5
4A	5/13/2023 14:00	23	22.0	15.5	-6.5
4A	5/13/2023 15:00	23	22.0	15.5	-6.5
4A	5/13/2023 16:00	23	22.0	18.0	-4.0

4A	5/13/2023 17:00	23	22.0	18.0	-4.0
4A	5/13/2023 18:00	23	22.0	18.0	-4.0
4A	5/13/2023 19:00	23	22.0	18.0	-4.0
4A	5/13/2023 20:00	23	22.0	18.0	-4.0
4A	5/13/2023 21:00	23	22.0	18.0	-4.0
4A	5/13/2023 22:00	23	22.0	18.0	-4.0
4A	5/13/2023 23:00	23	22.0	18.0	-4.0
4A	5/14/2023 0:00	23	22.0	17.2	-4.8
4A	5/14/2023 1:00	23	22.0	17.2	-4.8
4A	5/14/2023 2:00	23	22.0	17.2	-4.8
4A	5/14/2023 3:00	23	22.0	17.2	-4.8
4A	5/14/2023 4:00	23	22.0	17.2	-4.8
4A	5/14/2023 5:00	23	22.0	17.2	-4.8
4A	5/14/2023 6:00	23	22.0	17.2	-4.8
4A	5/14/2023 7:00	23	22.0	17.2	-4.8
4A	5/14/2023 8:00	23	22.0	15.5	-6.5
4A	5/14/2023 9:00	23	22.0	15.5	-6.5
4A	5/14/2023 10:00	23	22.0	15.5	-6.5
4A	5/14/2023 11:00	23	22.0	15.5	-6.5
4A	5/14/2023 12:00	23	22.0	18.0	-4.0
4A	5/14/2023 13:00	23	22.0	18.0	-4.0
4A	5/14/2023 14:00	23	22.0	18.0	-4.0
4A	5/14/2023 15:00	23	22.0	18.0	-4.0
4A	5/14/2023 16:00	23	22.0	18.0	-4.0
4A	5/14/2023 17:00	23	22.0	18.0	-4.0
4A	5/14/2023 18:00	23	22.0	18.0	-4.0
4A	5/14/2023 19:00	23	22.0	18.0	-4.0
4A	5/14/2023 20:00	23	22.0	16.6	-5.5
4A	5/14/2023 21:00	20	22.0	16.6	-5.5
4A	5/14/2023 22:00	20	22.0	16.6	-5.5
4A	5/14/2023 23:00	20	22.0	16.6	-5.5
4A	5/15/2023 0:00	20	22.0	13.8	-8.2
4A	5/15/2023 1:00	20	22.0	13.8	-8.2
4A	5/15/2023 2:00	20	22.0	13.8	-8.2
4A	5/15/2023 3:00	20	22.0	13.8	-8.2
4A	5/15/2023 4:00	20	22.0	13.8	-8.2
4A	5/15/2023 5:00	20	22.0	13.8	-8.2
4A	5/15/2023 6:00	20	22.0	13.8	-8.2
4A	5/15/2023 7:00	20	22.0	13.8	-8.2
4A	5/15/2023 8:00	20	22.0	12.9	-9.1
4A	5/15/2023 9:00	20	22.0	12.9	-9.1
4A	5/15/2023 10:00	20	22.0	12.9	-9.1
4A	5/15/2023 11:00	20	22.0	12.9	-9.1
4A	5/15/2023 12:00	20	22.0	12.9	-9.1
4A	5/15/2023 13:00	20	22.0	12.9	-9.1
4A	5/15/2023 14:00	20	22.0	12.9	-9.1
4A	5/15/2023 15:00	20	22.0	12.9	-9.1

4A	5/15/2023 16:00	21	22.0	13.8	-8.2
4A	5/15/2023 17:00	21	22.0	13.8	-8.2
4A	5/15/2023 18:00	21	22.0	13.8	-8.2
4A	5/15/2023 19:00	21	22.0	13.8	-8.2
4A	5/15/2023 20:00	21	22.0	18.0	-4.0
4A	5/15/2023 21:00	21	22.0	18.0	-4.0
4A	5/15/2023 22:00	21	22.0	18.0	-4.0
4A	5/15/2023 23:00	21	22.0	18.0	-4.0
4A	5/16/2023 0:00	21	22.0	18.0	-4.0
4A	5/16/2023 1:00	21	22.0	18.0	-4.0
4A	5/16/2023 2:00	21	22.0	18.0	-4.0
4A	5/16/2023 3:00	21	22.0	18.0	-4.0
4A	5/16/2023 4:00	21	22.0	18.0	-4.0
4A	5/16/2023 5:00	21	22.0	18.0	-4.0
4A	5/16/2023 6:00	21	22.0	18.0	-4.0
4A	5/16/2023 7:00	21	22.0	18.0	-4.0
4A	5/16/2023 8:00	21	21.0	10.4	-10.7
4A	5/16/2023 9:00	21	21.0	10.4	-10.7
4A	5/16/2023 10:00	21	21.0	10.4	-10.7
4A	5/16/2023 11:00	21	21.0	10.4	-10.7
4A	5/16/2023 12:00	21	21.0	15.5	-5.5
4A	5/16/2023 13:00	21	21.0	15.5	-5.5
4A	5/16/2023 14:00	21	21.0	15.5	-5.5
4A	5/16/2023 15:00	21	21.0	14.2	-6.8
4A	5/16/2023 16:00	21	21.0	17.2	-3.8
4A	5/16/2023 17:00	21	21.0	17.2	-3.8
4A	5/16/2023 18:00	21	21.0	17.2	-3.8
4A	5/16/2023 19:00	18	18.0	17.2	-0.8
4A	5/16/2023 20:00	18	18.0	17.2	-0.8
4A	5/16/2023 21:00	18	18.0	17.2	-0.8
4A	5/16/2023 22:00	18	18.0	17.2	-0.8
4A	5/16/2023 23:00	18	18.0	17.2	-0.8
4A	5/17/2023 0:00	18	18.0	13.8	-4.2
4A	5/17/2023 1:00	18	18.0	13.8	-4.2
4A	5/17/2023 2:00	18	18.0	13.8	-4.2
4A	5/17/2023 3:00	18	18.0	13.8	-4.2
4A	5/17/2023 4:00	18	18.0	13.8	-4.2
4A	5/17/2023 5:00	18	18.0	13.8	-4.2
4A	5/17/2023 6:00	18	18.0	13.8	-4.2
4A	5/17/2023 7:00	18	18.0	15.2	-2.8
4A	5/17/2023 8:00	18	18.0	7.8	-10.2
4A	5/17/2023 9:00	18	18.0	7.8	-10.2
4A	5/17/2023 10:00	18	18.0	7.8	-10.2
4A	5/17/2023 11:00	18	18.0	7.8	-10.2
4A	5/17/2023 12:00	18	18.0	10.4	-7.7
4A	5/17/2023 13:00	18	18.0	10.4	-7.7
4A	5/17/2023 14:00	18	18.0	10.4	-7.7

4A	5/17/2023 15:00	18	18.0	10.4	-7.7
4A	5/17/2023 16:00	18	18.0	13.8	-4.2
4A	5/17/2023 17:00	18	18.0	13.8	-4.2
4A	5/17/2023 18:00	18	18.0	13.8	-4.2
4A	5/17/2023 19:00	18	18.0	13.8	-4.2
4A	5/17/2023 20:00	18	18.0	17.2	-0.8
4A	5/17/2023 21:00	18	18.0	17.2	-0.8
4A	5/17/2023 22:00	18	18.0	17.2	-0.8
4A	5/17/2023 23:00	18	18.0	17.2	-0.8
4A	5/18/2023 0:00	18	18.0	13.8	-4.2
4A	5/18/2023 1:00	18	18.0	13.8	-4.2
4A	5/18/2023 2:00	18	18.0	13.8	-4.2
4A	5/18/2023 3:00	18	18.0	13.8	-4.2
4A	5/18/2023 4:00	18	18.0	13.8	-4.2
4A	5/18/2023 5:00	18	18.0	13.8	-4.2
4A	5/18/2023 6:00	18	18.0	13.8	-4.2
4A	5/18/2023 7:00	18	18.0	15.2	-2.8
4A	5/18/2023 8:00	18	18.0	15.5	-2.5
4A	5/18/2023 9:00	18	18.0	15.5	-2.5
4A	5/18/2023 10:00	18	18.0	15.5	-2.5
4A	5/18/2023 11:00	18	18.0	15.5	-2.5
4A	5/18/2023 12:00	18	18.0	15.5	-2.5
4A	5/18/2023 13:00	18	18.0	15.5	-2.5
4A	5/18/2023 14:00	18	18.0	15.5	-2.5
4A	5/18/2023 15:00	18	18.0	15.5	-2.5
4A	5/18/2023 16:00	18	18.0	18.0	0.0
4A	5/18/2023 17:00	18	18.0	18.0	0.0
4A	5/18/2023 18:00	18	18.0	18.0	0.0
4A	5/18/2023 19:00	18	18.0	18.0	0.0
4A	5/18/2023 20:00	18	18.0	18.0	0.0
4A	5/18/2023 21:00	18	18.0	18.0	0.0
4A	5/18/2023 22:00	20	18.0	18.0	0.0
4A	5/18/2023 23:00	20	18.0	18.0	0.0
4A	5/19/2023 0:00	20	18.0	18.0	0.0
4A	5/19/2023 1:00	20	18.0	18.0	0.0
4A	5/19/2023 2:00	20	18.0	18.0	0.0
4A	5/19/2023 3:00	20	18.0	18.0	0.0
4A	5/19/2023 4:00	20	18.0	18.0	0.0
4A	5/19/2023 5:00	20	18.0	18.0	0.0
4A	5/19/2023 6:00	22	18.0	18.0	0.0
4A	5/19/2023 7:00	22	18.0	18.0	0.0
4A	5/19/2023 8:00	22	18.0	18.0	0.0
4A	5/19/2023 9:00	22	18.0	18.0	0.0
4A	5/19/2023 10:00	22	18.0	18.0	0.0
4A	5/19/2023 11:00	22	18.0	18.0	0.0
4A	5/19/2023 12:00	22	18.0	18.0	0.0
4A	5/19/2023 13:00	22	18.0	18.0	0.0

4A	5/19/2023 14:00	22	18.0	18.0	0.0
4A	5/19/2023 15:00	22	18.0	18.0	0.0
4A	5/19/2023 16:00	22	18.0	18.0	0.0
4A	5/19/2023 17:00	22	18.0	18.0	0.0
4A	5/19/2023 18:00	22	18.0	18.0	0.0
4A	5/19/2023 19:00	22	18.0	18.0	0.0
4A	5/19/2023 20:00	22	18.0	13.8	-4.2
4A	5/19/2023 21:00	22	18.0	13.8	-4.2
4A	5/19/2023 22:00	22	18.0	13.8	-4.2
4A	5/19/2023 23:00	22	18.0	13.8	-4.2
4A	5/20/2023 0:00	22	18.0	13.8	-4.2
4A	5/20/2023 1:00	22	18.0	13.8	-4.2
4A	5/20/2023 2:00	22	18.0	13.8	-4.2
4A	5/20/2023 3:00	22	18.0	13.8	-4.2
4A	5/20/2023 4:00	22	18.0	13.8	-4.2
4A	5/20/2023 5:00	15	18.0	13.8	-4.2
4A	5/20/2023 6:00	15	18.0	13.8	-4.2
4A	5/20/2023 7:00	15	18.0	13.8	-4.2
4A	5/20/2023 8:00	15	18.0	14.5	-3.5
4A	5/20/2023 9:00	18	18.0	14.5	-3.5
4A	5/20/2023 10:00	18	18.0	14.5	-3.5
4A	5/20/2023 11:00	18	18.0	14.5	-3.5
4A	5/20/2023 12:00	18	18.0	16.6	-1.5
4A	5/20/2023 13:00	18	18.0	16.6	-1.5
4A	5/20/2023 14:00	18	18.0	16.6	-1.5
4A	5/20/2023 15:00	18	18.0	16.6	-1.5
4A	5/20/2023 16:00	18	18.0	18.0	0.0
4A	5/20/2023 17:00	18	18.0	18.0	0.0
4A	5/20/2023 18:00	18	18.0	18.0	0.0
4A	5/20/2023 19:00	18	18.0	18.0	0.0
4A	5/20/2023 20:00	18	18.0	17.2	-0.8
4A	5/20/2023 21:00	18	18.0	17.2	-0.8
4A	5/20/2023 22:00	18	18.0	17.2	-0.8
4A	5/20/2023 23:00	18	18.0	17.2	-0.8
4A	5/21/2023 0:00	18	18.0	13.8	-4.2
4A	5/21/2023 1:00	18	18.0	13.8	-4.2
4A	5/21/2023 2:00	18	18.0	13.8	-4.2
4A	5/21/2023 3:00	17	18.0	13.8	-4.2
4A	5/21/2023 4:00	17	18.0	13.8	-4.2
4A	5/21/2023 5:00	17	18.0	13.8	-4.2
4A	5/21/2023 6:00	17	18.0	13.8	-4.2
4A	5/21/2023 7:00	17	18.0	13.8	-4.2
4A	5/21/2023 8:00	17	18.0	12.4	-5.6
4A	5/21/2023 9:00	17	18.0	12.4	-5.6
4A	5/21/2023 10:00	17	18.0	12.4	-5.6
4A	5/21/2023 11:00	17	18.0	12.4	-5.6
4A	5/21/2023 12:00	17	18.0	14.5	-3.5

4A	5/21/2023 13:00	17	18.0	14.5	-3.5
4A	5/21/2023 14:00	17	18.0	14.5	-3.5
4A	5/21/2023 15:00	17	18.0	14.5	-3.5
4A	5/21/2023 16:00	17	18.0	18.0	0.0
4A	5/21/2023 17:00	17	18.0	18.0	0.0
4A	5/21/2023 18:00	17	18.0	18.0	0.0
4A	5/21/2023 19:00	17	18.0	18.0	0.0
4A	5/21/2023 20:00	17	18.0	18.0	0.0
4A	5/21/2023 21:00	17	18.0	18.0	0.0
4A	5/21/2023 22:00	17	18.0	18.0	0.0
4A	5/21/2023 23:00	17	18.0	18.0	0.0
4A	5/22/2023 0:00	17	18.0	18.0	0.0
4A	5/22/2023 1:00	17	18.0	18.0	0.0
4A	5/22/2023 2:00	17	18.0	18.0	0.0
4A	5/22/2023 3:00	17	18.0	18.0	0.0
4A	5/22/2023 4:00	17	18.0	18.0	0.0
4A	5/22/2023 5:00	17	18.0	18.0	0.0
4A	5/22/2023 6:00	17	18.0	18.0	0.0
4A	5/22/2023 7:00	17	18.0	18.0	0.0
4A	5/22/2023 8:00	17	18.0	7.8	-10.2
4A	5/22/2023 9:00	17	18.0	7.8	-10.2
4A	5/22/2023 10:00	17	18.0	7.8	-10.2
4A	5/22/2023 11:00	17	18.0	7.8	-10.2
4A	5/22/2023 12:00	17	18.0	7.8	-10.2
4A	5/22/2023 13:00	17	18.0	7.8	-10.2
4A	5/22/2023 14:00	17	18.0	7.8	-10.2
4A	5/22/2023 15:00	17	18.0	7.8	-10.2
4A	5/22/2023 16:00	17	18.0	10.4	-7.7
4A	5/22/2023 17:00	17	18.0	10.4	-7.7
4A	5/22/2023 18:00	17	18.0	10.4	-7.7
4A	5/22/2023 19:00	17	18.0	10.4	-7.7
4A	5/22/2023 20:00	17	18.0	13.8	-4.2
4A	5/22/2023 21:00	17	18.0	13.8	-4.2
4A	5/22/2023 22:00	17	18.0	13.8	-4.2
4A	5/22/2023 23:00	17	18.0	13.8	-4.2
4A	5/23/2023 0:00	17	18.0	18.0	0.0
4A	5/23/2023 1:00	21	18.0	18.0	0.0
4A	5/23/2023 2:00	21	18.0	18.0	0.0
4A	5/23/2023 3:00	21	18.0	18.0	0.0
4A	5/23/2023 4:00	21	18.0	18.0	0.0
4A	5/23/2023 5:00	21	18.0	18.0	0.0
4A	5/23/2023 6:00	21	18.0	18.0	0.0
4A	5/23/2023 7:00	21	18.0	18.0	0.0
4A	5/23/2023 8:00	21	18.0	10.4	-7.7
4A	5/23/2023 9:00	21	18.0	10.4	-7.7
4A	5/23/2023 10:00	21	18.0	10.4	-7.7
4A	5/23/2023 11:00	21	18.0	10.4	-7.7

4A	5/23/2023 12:00	21	18.0	10.4	-7.7
4A	5/23/2023 13:00	21	18.0	10.4	-7.7
4A	5/23/2023 14:00	21	18.0	10.4	-7.7
4A	5/23/2023 15:00	21	18.0	10.4	-7.7
4A	5/23/2023 16:00	21	18.0	13.8	-4.2
4A	5/23/2023 17:00	21	18.0	13.8	-4.2
4A	5/23/2023 18:00	21	18.0	13.8	-4.2
4A	5/23/2023 19:00	21	18.0	13.8	-4.2
4A	5/23/2023 20:00	22	22.0	17.2	-4.8
4A	5/23/2023 21:00	22	22.0	17.2	-4.8
4A	5/23/2023 22:00	22	22.0	17.2	-4.8
4A	5/23/2023 23:00	22	22.0	17.2	-4.8
4A	5/24/2023 0:00	22	22.0	17.2	-4.8
4A	5/24/2023 1:00	22	22.0	17.2	-4.8
4A	5/24/2023 2:00	22	22.0	17.2	-4.8
4A	5/24/2023 3:00	22	22.0	17.2	-4.8
4A	5/24/2023 4:00	22	22.0	17.2	-4.8
4A	5/24/2023 5:00	22	22.0	17.2	-4.8
4A	5/24/2023 6:00	22	22.0	17.2	-4.8
4A	5/24/2023 7:00	22	22.0	17.2	-4.8
4A	5/24/2023 8:00	22	22.0	18.0	-4.0
4A	5/24/2023 9:00	22	22.0	18.0	-4.0
4A	5/24/2023 10:00	22	22.0	18.0	-4.0
4A	5/24/2023 11:00	22	22.0	18.0	-4.0
4A	5/24/2023 12:00	22	22.0	18.0	-4.0
4A	5/24/2023 13:00	22	22.0	18.0	-4.0
4A	5/24/2023 14:00	22	22.0	18.0	-4.0
4A	5/24/2023 15:00	22	22.0	18.0	-4.0
4A	5/24/2023 16:00	22	22.0	18.0	-4.0
4A	5/24/2023 17:00	22	22.0	18.0	-4.0
4A	5/24/2023 18:00	22	22.0	18.0	-4.0
4A	5/24/2023 19:00	18	18.0	18.0	0.0
4A	5/24/2023 20:00	18	18.0	17.2	-0.8
4A	5/24/2023 21:00	18	18.0	17.2	-0.8
4A	5/24/2023 22:00	18	18.0	17.2	-0.8
4A	5/24/2023 23:00	18	18.0	17.2	-0.8
4A	5/25/2023 0:00	19	19.0	16.6	-2.5
4A	5/25/2023 1:00	19	19.0	14.5	-4.5
4A	5/25/2023 2:00	19	19.0	16.6	-2.5
4A	5/25/2023 3:00	19	19.0	16.6	-2.5
4A	5/25/2023 4:00	19	19.0	16.6	-2.5
4A	5/25/2023 5:00	19	19.0	16.6	-2.5
4A	5/25/2023 6:00	19	19.0	16.6	-2.5
4A	5/25/2023 7:00	19	19.0	16.6	-2.5
4A	5/25/2023 8:00	19	21.0	15.5	-5.5
4A	5/25/2023 9:00	19	21.0	15.5	-5.5
4A	5/25/2023 10:00	19	21.0	15.5	-5.5

4A	5/25/2023 11:00	19	21.0	15.5	-5.5
4A	5/25/2023 12:00	19	21.0	15.5	-5.5
4A	5/25/2023 13:00	19	21.0	15.5	-5.5
4A	5/25/2023 14:00	19	21.0	15.5	-5.5
4A	5/25/2023 15:00	19	21.0	15.5	-5.5
4A	5/25/2023 16:00	19	21.0	17.2	-3.8
4A	5/25/2023 17:00	19	21.0	17.2	-3.8
4A	5/25/2023 18:00	19	21.0	17.2	-3.8
4A	5/25/2023 19:00	19	21.0	17.2	-3.8
4A	5/25/2023 20:00	19	21.0	18.0	-3.0
4A	5/25/2023 21:00	19	21.0	18.0	-3.0
4A	5/25/2023 22:00	19	21.0	18.0	-3.0
4A	5/25/2023 23:00	19	21.0	18.0	-3.0
ICU	1/1/2023 0:00	10	10.0	10.4	0.4
ICU	1/1/2023 1:00	10	10.0	10.4	0.4
ICU	1/1/2023 2:00	10	10.0	10.4	0.4
ICU	1/1/2023 3:00	10	10.0	10.4	0.4
ICU	1/1/2023 4:00	10	10.0	10.4	0.4
ICU	1/1/2023 5:00	10	10.0	10.4	0.4
ICU	1/1/2023 6:00	10	10.0	10.4	0.4
ICU	1/1/2023 7:00	10	10.0	10.4	0.4
ICU	1/1/2023 8:00	10	10.0	12.6	2.6
ICU	1/1/2023 9:00	10	10.0	12.6	2.6
ICU	1/1/2023 10:00	10	8.0	12.6	4.6
ICU	1/1/2023 11:00	10	8.0	12.6	4.6
ICU	1/1/2023 12:00	10	8.0	12.6	4.6
ICU	1/1/2023 13:00	10	8.0	12.6	4.6
ICU	1/1/2023 14:00	7	12.0	12.6	0.6
ICU	1/1/2023 15:00	7	12.0	12.6	0.6
ICU	1/1/2023 16:00	7	12.0	12.6	0.6
ICU	1/1/2023 17:00	7	12.0	12.6	0.6
ICU	1/1/2023 18:00	7	12.0	12.6	0.6
ICU	1/1/2023 19:00	7	12.0	12.6	0.6
ICU	1/1/2023 20:00	6	8.0	9.5	1.5
ICU	1/1/2023 21:00	6	8.0	9.5	1.5
ICU	1/1/2023 22:00	6	8.0	9.5	1.5
ICU	1/1/2023 23:00	6	8.0	9.5	1.5
ICU	1/2/2023 0:00	6	8.0	9.5	1.5
ICU	1/2/2023 1:00	6	8.0	9.5	1.5
ICU	1/2/2023 2:00	6	8.0	9.5	1.5
ICU	1/2/2023 3:00	6	8.0	9.5	1.5
ICU	1/2/2023 4:00	6	8.0	9.5	1.5
ICU	1/2/2023 5:00	6	8.0	9.5	1.5
ICU	1/2/2023 6:00	6	8.0	9.5	1.5
ICU	1/2/2023 7:00	6	8.0	9.5	1.5
ICU	1/2/2023 8:00	6	10.0	11.0	1.0
ICU	1/2/2023 9:00	6	10.0	11.0	1.0

ICU	1/2/2023 10:00	6	10.0	11.0	1.0
ICU	1/2/2023 11:00	6	10.0	11.0	1.0
ICU	1/2/2023 12:00	7	10.0	9.9	-0.1
ICU	1/2/2023 13:00	7	10.0	11.0	1.0
ICU	1/2/2023 14:00	7	10.0	11.0	1.0
ICU	1/2/2023 15:00	7	10.0	11.0	1.0
ICU	1/2/2023 16:00	7	10.0	11.0	1.0
ICU	1/2/2023 17:00	7	10.0	11.0	1.0
ICU	1/2/2023 18:00	7	10.0	11.0	1.0
ICU	1/2/2023 19:00	7	8.0	11.0	3.0
ICU	1/2/2023 20:00	8	8.0	9.9	1.9
ICU	1/2/2023 21:00	8	8.0	11.0	3.0
ICU	1/2/2023 22:00	8	8.0	11.0	3.0
ICU	1/2/2023 23:00	8	8.0	11.0	3.0
ICU	1/3/2023 0:00	8	8.0	11.0	3.0
ICU	1/3/2023 1:00	8	8.0	11.0	3.0
ICU	1/3/2023 2:00	8	8.0	11.0	3.0
ICU	1/3/2023 3:00	8	8.0	11.0	3.0
ICU	1/3/2023 4:00	8	8.0	11.0	3.0
ICU	1/3/2023 5:00	8	8.0	11.0	3.0
ICU	1/3/2023 6:00	8	8.0	11.0	3.0
ICU	1/3/2023 7:00	8	8.0	11.0	3.0
ICU	1/3/2023 8:00	8	8.0	12.6	4.6
ICU	1/3/2023 9:00	8	8.0	12.6	4.6
ICU	1/3/2023 10:00	8	8.0	12.6	4.6
ICU	1/3/2023 11:00	8	8.0	12.6	4.6
ICU	1/3/2023 12:00	8	8.0	14.2	6.2
ICU	1/3/2023 13:00	8	8.0	14.2	6.2
ICU	1/3/2023 14:00	8	8.0	14.2	6.2
ICU	1/3/2023 15:00	8	8.0	14.2	6.2
ICU	1/3/2023 16:00	8	8.0	11.0	3.0
ICU	1/3/2023 17:00	8	8.0	11.0	3.0
ICU	1/3/2023 18:00	8	8.0	11.0	3.0
ICU	1/3/2023 19:00	8	8.0	11.0	3.0
ICU	1/3/2023 20:00	9	9.0	9.5	0.5
ICU	1/3/2023 21:00	9	9.0	9.5	0.5
ICU	1/3/2023 22:00	9	9.0	8.4	-0.7
ICU	1/3/2023 23:00	9	9.0	9.5	0.5
ICU	1/4/2023 0:00	9	9.0	8.9	-0.1
ICU	1/4/2023 1:00	9	9.0	8.9	-0.1
ICU	1/4/2023 2:00	9	9.0	8.9	-0.1
ICU	1/4/2023 3:00	9	9.0	8.9	-0.1
ICU	1/4/2023 4:00	9	9.0	8.9	-0.1
ICU	1/4/2023 5:00	9	9.0	8.9	-0.1
ICU	1/4/2023 6:00	9	9.0	8.9	-0.1
ICU	1/4/2023 7:00	9	9.0	8.9	-0.1
ICU	1/4/2023 8:00	9	9.0	9.5	0.5

ICU	1/4/2023 9:00	9	9.0	9.5	0.5
ICU	1/4/2023 10:00	9	9.0	9.5	0.5
ICU	1/4/2023 11:00	9	9.0	9.5	0.5
ICU	1/4/2023 12:00	9	9.0	9.5	0.5
ICU	1/4/2023 13:00	9	9.0	9.5	0.5
ICU	1/4/2023 14:00	9	9.0	9.5	0.5
ICU	1/4/2023 15:00	9	9.0	9.5	0.5
ICU	1/4/2023 16:00	9	9.0	8.9	-0.1
ICU	1/4/2023 17:00	9	9.0	8.9	-0.1
ICU	1/4/2023 18:00	9	9.0	8.9	-0.1
ICU	1/4/2023 19:00	9	9.0	8.9	-0.1
ICU	1/4/2023 20:00	10	11.0	8.9	-2.1
ICU	1/4/2023 21:00	10	11.0	8.9	-2.1
ICU	1/4/2023 22:00	10	11.0	8.9	-2.1
ICU	1/4/2023 23:00	10	11.0	8.9	-2.1
ICU	1/5/2023 0:00	10	11.0	8.9	-2.1
ICU	1/5/2023 1:00	10	11.0	8.9	-2.1
ICU	1/5/2023 2:00	10	11.0	8.9	-2.1
ICU	1/5/2023 3:00	10	11.0	8.9	-2.1
ICU	1/5/2023 4:00	10	11.0	8.9	-2.1
ICU	1/5/2023 5:00	10	11.0	8.9	-2.1
ICU	1/5/2023 6:00	10	11.0	8.9	-2.1
ICU	1/5/2023 7:00	10	11.0	8.9	-2.1
ICU	1/5/2023 8:00	10	11.0	11.0	0.0
ICU	1/5/2023 9:00	10	11.0	9.5	-1.5
ICU	1/5/2023 10:00	10	11.0	9.5	-1.5
ICU	1/5/2023 11:00	10	11.0	9.5	-1.5
ICU	1/5/2023 12:00	10	11.0	9.5	-1.5
ICU	1/5/2023 13:00	10	11.0	9.5	-1.5
ICU	1/5/2023 14:00	9	11.0	8.4	-2.7
ICU	1/5/2023 15:00	9	11.0	9.5	-1.5
ICU	1/5/2023 16:00	9	11.0	9.5	-1.5
ICU	1/5/2023 17:00	9	11.0	9.5	-1.5
ICU	1/5/2023 18:00	9	11.0	9.5	-1.5
ICU	1/5/2023 19:00	9	11.0	9.5	-1.5
ICU	1/5/2023 20:00	8	9.0	9.5	0.5
ICU	1/5/2023 21:00	8	9.0	9.5	0.5
ICU	1/5/2023 22:00	8	9.0	8.4	-0.7
ICU	1/5/2023 23:00	9	9.0	8.4	-0.7
ICU	1/6/2023 0:00	9	9.0	11.0	2.0
ICU	1/6/2023 1:00	9	9.0	11.0	2.0
ICU	1/6/2023 2:00	9	9.0	11.0	2.0
ICU	1/6/2023 3:00	9	9.0	11.0	2.0
ICU	1/6/2023 4:00	9	9.0	11.0	2.0
ICU	1/6/2023 5:00	9	9.0	11.0	2.0
ICU	1/6/2023 6:00	9	9.0	11.0	2.0
ICU	1/6/2023 7:00	9	9.0	11.0	2.0

ICU	1/6/2023 8:00	9	9.0	9.5	0.5
ICU	1/6/2023 9:00	9	9.0	9.5	0.5
ICU	1/6/2023 10:00	9	9.0	9.5	0.5
ICU	1/6/2023 11:00	9	9.0	9.5	0.5
ICU	1/6/2023 12:00	9	9.0	9.5	0.5
ICU	1/6/2023 13:00	10	11.0	9.5	-1.5
ICU	1/6/2023 14:00	10	11.0	9.5	-1.5
ICU	1/6/2023 15:00	8	11.0	9.5	-1.5
ICU	1/6/2023 16:00	8	11.0	9.5	-1.5
ICU	1/6/2023 17:00	8	11.0	9.5	-1.5
ICU	1/6/2023 18:00	8	11.0	9.5	-1.5
ICU	1/6/2023 19:00	8	11.0	9.5	-1.5
ICU	1/6/2023 20:00	9	11.0	10.4	-0.6
ICU	1/6/2023 21:00	9	11.0	10.4	-0.6
ICU	1/6/2023 22:00	9	11.0	10.4	-0.6
ICU	1/6/2023 23:00	9	11.0	10.4	-0.6
ICU	1/7/2023 0:00	9	11.0	11.0	0.0
ICU	1/7/2023 1:00	9	11.0	11.0	0.0
ICU	1/7/2023 2:00	9	11.0	11.0	0.0
ICU	1/7/2023 3:00	9	11.0	11.0	0.0
ICU	1/7/2023 4:00	9	11.0	11.0	0.0
ICU	1/7/2023 5:00	9	11.0	11.0	0.0
ICU	1/7/2023 6:00	9	11.0	11.0	0.0
ICU	1/7/2023 7:00	9	11.0	11.0	0.0
ICU	1/7/2023 8:00	9	11.0	11.0	0.0
ICU	1/7/2023 9:00	9	9.0	11.0	2.0
ICU	1/7/2023 10:00	9	9.0	11.0	2.0
ICU	1/7/2023 11:00	9	9.0	11.0	2.0
ICU	1/7/2023 12:00	9	9.0	11.0	2.0
ICU	1/7/2023 13:00	7	12.0	12.6	0.6
ICU	1/7/2023 14:00	7	12.0	12.6	0.6
ICU	1/7/2023 15:00	7	12.0	12.6	0.6
ICU	1/7/2023 16:00	7	12.0	12.6	0.6
ICU	1/7/2023 17:00	7	12.0	12.6	0.6
ICU	1/7/2023 18:00	7	12.0	12.6	0.6
ICU	1/7/2023 19:00	9	12.0	12.6	0.6
ICU	1/7/2023 20:00	8	12.0	12.6	0.6
ICU	1/7/2023 21:00	8	12.0	12.6	0.6
ICU	1/7/2023 22:00	8	12.0	12.6	0.6
ICU	1/7/2023 23:00	8	12.0	12.6	0.6
ICU	1/8/2023 0:00	8	12.0	11.9	-0.2
ICU	1/8/2023 1:00	8	12.0	11.9	-0.2
ICU	1/8/2023 2:00	8	12.0	11.9	-0.2
ICU	1/8/2023 3:00	8	12.0	11.9	-0.2
ICU	1/8/2023 4:00	8	12.0	11.9	-0.2
ICU	1/8/2023 5:00	8	12.0	11.9	-0.2
ICU	1/8/2023 6:00	8	12.0	11.9	-0.2

ICU	1/8/2023 7:00	8	12.0	11.9	-0.2
ICU	1/8/2023 8:00	8	10.0	10.4	0.4
ICU	1/8/2023 9:00	8	10.0	11.0	1.0
ICU	1/8/2023 10:00	8	10.0	11.0	1.0
ICU	1/8/2023 11:00	8	10.0	11.0	1.0
ICU	1/8/2023 12:00	8	10.0	12.6	2.6
ICU	1/8/2023 13:00	8	10.0	12.6	2.6
ICU	1/8/2023 14:00	8	10.0	12.6	2.6
ICU	1/8/2023 15:00	8	10.0	12.6	2.6
ICU	1/8/2023 16:00	8	10.0	11.0	1.0
ICU	1/8/2023 17:00	8	10.0	11.0	1.0
ICU	1/8/2023 18:00	8	10.0	11.0	1.0
ICU	1/8/2023 19:00	8	11.0	11.0	0.0
ICU	1/8/2023 20:00	8	11.0	11.9	0.9
ICU	1/8/2023 21:00	8	11.0	10.4	-0.6
ICU	1/8/2023 22:00	8	11.0	10.4	-0.6
ICU	1/8/2023 23:00	8	11.0	10.4	-0.6
ICU	1/9/2023 0:00	8	11.0	10.4	-0.6
ICU	1/9/2023 1:00	8	11.0	10.4	-0.6
ICU	1/9/2023 2:00	8	11.0	10.4	-0.6
ICU	1/9/2023 3:00	8	11.0	10.4	-0.6
ICU	1/9/2023 4:00	8	11.0	10.4	-0.6
ICU	1/9/2023 5:00	8	11.0	10.4	-0.6
ICU	1/9/2023 6:00	8	11.0	10.4	-0.6
ICU	1/9/2023 7:00	8	11.0	10.4	-0.6
ICU	1/9/2023 8:00	9	11.0	10.4	-0.6
ICU	1/9/2023 9:00	9	11.0	10.4	-0.6
ICU	1/9/2023 10:00	9	11.0	10.4	-0.6
ICU	1/9/2023 11:00	9	11.0	10.4	-0.6
ICU	1/9/2023 12:00	9	11.0	10.4	-0.6
ICU	1/9/2023 13:00	9	11.0	10.4	-0.6
ICU	1/9/2023 14:00	9	11.0	10.4	-0.6
ICU	1/9/2023 15:00	9	11.0	10.4	-0.6
ICU	1/9/2023 16:00	9	11.0	11.0	0.0
ICU	1/9/2023 17:00	9	11.0	11.0	0.0
ICU	1/9/2023 18:00	9	11.0	11.0	0.0
ICU	1/9/2023 19:00	9	11.0	11.0	0.0
ICU	1/9/2023 20:00	9	11.0	11.0	0.0
ICU	1/9/2023 21:00	9	11.0	11.0	0.0
ICU	1/9/2023 22:00	9	11.0	11.0	0.0
ICU	1/9/2023 23:00	9	11.0	11.0	0.0
ICU	1/10/2023 0:00	9	11.0	11.0	0.0
ICU	1/10/2023 1:00	9	11.0	11.0	0.0
ICU	1/10/2023 2:00	9	11.0	11.0	0.0
ICU	1/10/2023 3:00	9	11.0	11.0	0.0
ICU	1/10/2023 4:00	9	11.0	11.0	0.0
ICU	1/10/2023 5:00	9	11.0	11.0	0.0

ICU	1/10/2023 6:00	9	11.0	11.0	0.0
ICU	1/10/2023 7:00	9	11.0	11.0	0.0
ICU	1/10/2023 8:00	11	12.0	12.6	0.6
ICU	1/10/2023 9:00	11	12.0	12.6	0.6
ICU	1/10/2023 10:00	11	12.0	12.6	0.6
ICU	1/10/2023 11:00	10	12.0	11.5	-0.5
ICU	1/10/2023 12:00	11	12.0	10.7	-1.3
ICU	1/10/2023 13:00	12	12.0	10.7	-1.3
ICU	1/10/2023 14:00	12	12.0	11.9	-0.2
ICU	1/10/2023 15:00	11	12.0	10.7	-1.3
ICU	1/10/2023 16:00	11	12.0	12.6	0.6
ICU	1/10/2023 17:00	12	12.0	11.5	-0.5
ICU	1/10/2023 18:00	12	12.0	12.6	0.6
ICU	1/10/2023 19:00	12	12.0	12.6	0.6
ICU	1/10/2023 20:00	12	12.0	12.6	0.6
ICU	1/10/2023 21:00	12	12.0	12.6	0.6
ICU	1/10/2023 22:00	12	12.0	12.6	0.6
ICU	1/10/2023 23:00	12	12.0	12.6	0.6
ICU	1/11/2023 0:00	12	12.0	12.6	0.6
ICU	1/11/2023 1:00	12	12.0	12.6	0.6
ICU	1/11/2023 2:00	12	12.0	12.6	0.6
ICU	1/11/2023 3:00	12	12.0	12.6	0.6
ICU	1/11/2023 4:00	12	12.0	12.6	0.6
ICU	1/11/2023 5:00	12	12.0	12.6	0.6
ICU	1/11/2023 6:00	12	12.0	12.6	0.6
ICU	1/11/2023 7:00	12	12.0	12.6	0.6
ICU	1/11/2023 8:00	12	12.0	12.6	0.6
ICU	1/11/2023 9:00	12	12.0	12.6	0.6
ICU	1/11/2023 10:00	12	12.0	12.6	0.6
ICU	1/11/2023 11:00	12	12.0	11.8	-0.2
ICU	1/11/2023 12:00	12	12.0	12.6	0.6
ICU	1/11/2023 13:00	12	12.0	12.6	0.6
ICU	1/11/2023 14:00	12	12.0	12.6	0.6
ICU	1/11/2023 15:00	12	12.0	12.6	0.6
ICU	1/11/2023 16:00	12	12.0	11.0	-1.0
ICU	1/11/2023 17:00	12	12.0	11.0	-1.0
ICU	1/11/2023 18:00	12	12.0	11.0	-1.0
ICU	1/11/2023 19:00	12	12.0	11.0	-1.0
ICU	1/11/2023 20:00	12	12.0	12.6	0.6
ICU	1/11/2023 21:00	11	12.0	12.6	0.6
ICU	1/11/2023 22:00	11	12.0	12.6	0.6
ICU	1/11/2023 23:00	11	12.0	12.6	0.6
ICU	1/12/2023 0:00	11	12.0	12.6	0.6
ICU	1/12/2023 1:00	11	12.0	12.6	0.6
ICU	1/12/2023 2:00	11	12.0	12.6	0.6
ICU	1/12/2023 3:00	11	12.0	12.6	0.6
ICU	1/12/2023 4:00	11	12.0	12.6	0.6

ICU	1/12/2023 5:00	11	12.0	12.6	0.6
ICU	1/12/2023 6:00	11	12.0	12.6	0.6
ICU	1/12/2023 7:00	11	12.0	12.6	0.6
ICU	1/12/2023 8:00	11	12.0	9.5	-2.5
ICU	1/12/2023 9:00	11	12.0	9.5	-2.5
ICU	1/12/2023 10:00	11	12.0	9.5	-2.5
ICU	1/12/2023 11:00	11	12.0	9.5	-2.5
ICU	1/12/2023 12:00	11	12.0	12.6	0.6
ICU	1/12/2023 13:00	11	12.0	12.6	0.6
ICU	1/12/2023 14:00	11	12.0	12.6	0.6
ICU	1/12/2023 15:00	11	12.0	12.6	0.6
ICU	1/12/2023 16:00	11	12.0	11.0	-1.0
ICU	1/12/2023 17:00	11	12.0	11.0	-1.0
ICU	1/12/2023 18:00	11	12.0	11.0	-1.0
ICU	1/12/2023 19:00	11	12.0	11.0	-1.0
ICU	1/12/2023 20:00	11	11.0	11.0	0.0
ICU	1/12/2023 21:00	11	11.0	11.0	0.0
ICU	1/12/2023 22:00	11	11.0	11.0	0.0
ICU	1/12/2023 23:00	11	11.0	11.0	0.0
ICU	1/13/2023 0:00	11	11.0	11.0	0.0
ICU	1/13/2023 1:00	11	11.0	11.0	0.0
ICU	1/13/2023 2:00	11	11.0	11.0	0.0
ICU	1/13/2023 3:00	11	11.0	11.0	0.0
ICU	1/13/2023 4:00	11	11.0	11.0	0.0
ICU	1/13/2023 5:00	11	11.0	11.0	0.0
ICU	1/13/2023 6:00	11	11.0	11.0	0.0
ICU	1/13/2023 7:00	11	11.0	11.0	0.0
ICU	1/13/2023 8:00	11	11.0	9.5	-1.5
ICU	1/13/2023 9:00	11	11.0	9.5	-1.5
ICU	1/13/2023 10:00	11	11.0	9.5	-1.5
ICU	1/13/2023 11:00	11	11.0	9.5	-1.5
ICU	1/13/2023 12:00	11	11.0	9.5	-1.5
ICU	1/13/2023 13:00	11	11.0	9.5	-1.5
ICU	1/13/2023 14:00	11	11.0	9.5	-1.5
ICU	1/13/2023 15:00	11	11.0	9.5	-1.5
ICU	1/13/2023 16:00	9	11.0	8.9	-2.1
ICU	1/13/2023 17:00	9	11.0	8.9	-2.1
ICU	1/13/2023 18:00	9	11.0	8.9	-2.1
ICU	1/13/2023 19:00	9	11.0	8.9	-2.1
ICU	1/13/2023 20:00	9	10.0	10.4	0.4
ICU	1/13/2023 21:00	9	10.0	10.4	0.4
ICU	1/13/2023 22:00	9	10.0	10.4	0.4
ICU	1/13/2023 23:00	9	10.0	10.4	0.4
ICU	1/14/2023 0:00	9	10.0	11.0	1.0
ICU	1/14/2023 1:00	9	10.0	11.0	1.0
ICU	1/14/2023 2:00	9	10.0	11.0	1.0
ICU	1/14/2023 3:00	9	10.0	11.0	1.0

ICU	1/14/2023 4:00	9	10.0	11.0	1.0
ICU	1/14/2023 5:00	9	9.0	9.5	0.5
ICU	1/14/2023 6:00	9	9.0	9.5	0.5
ICU	1/14/2023 7:00	9	9.0	9.5	0.5
ICU	1/14/2023 8:00	9	9.0	8.9	-0.1
ICU	1/14/2023 9:00	9	11.0	8.9	-2.1
ICU	1/14/2023 10:00	9	11.0	8.9	-2.1
ICU	1/14/2023 11:00	9	11.0	8.9	-2.1
ICU	1/14/2023 12:00	9	11.0	10.4	-0.6
ICU	1/14/2023 13:00	9	11.0	10.4	-0.6
ICU	1/14/2023 14:00	9	11.0	10.4	-0.6
ICU	1/14/2023 15:00	9	11.0	10.4	-0.6
ICU	1/14/2023 16:00	9	11.0	11.0	0.0
ICU	1/14/2023 17:00	9	11.0	11.0	0.0
ICU	1/14/2023 18:00	9	11.0	11.0	0.0
ICU	1/14/2023 19:00	9	11.0	11.0	0.0
ICU	1/14/2023 20:00	8	10.0	11.0	1.0
ICU	1/14/2023 21:00	8	10.0	11.0	1.0
ICU	1/14/2023 22:00	8	10.0	11.0	1.0
ICU	1/14/2023 23:00	8	10.0	11.0	1.0
ICU	1/15/2023 0:00	8	10.0	10.4	0.4
ICU	1/15/2023 1:00	8	10.0	10.4	0.4
ICU	1/15/2023 2:00	8	10.0	9.3	-0.7
ICU	1/15/2023 3:00	9	10.0	9.3	-0.7
ICU	1/15/2023 4:00	9	10.0	10.4	0.4
ICU	1/15/2023 5:00	9	10.0	10.4	0.4
ICU	1/15/2023 6:00	9	10.0	10.4	0.4
ICU	1/15/2023 7:00	9	10.0	10.4	0.4
ICU	1/15/2023 8:00	9	12.0	12.6	0.6
ICU	1/15/2023 9:00	9	12.0	12.6	0.6
ICU	1/15/2023 10:00	9	12.0	12.6	0.6
ICU	1/15/2023 11:00	9	12.0	12.6	0.6
ICU	1/15/2023 12:00	10	12.0	12.6	0.6
ICU	1/15/2023 13:00	9	12.0	12.6	0.6
ICU	1/15/2023 14:00	9	12.0	12.6	0.6
ICU	1/15/2023 15:00	9	12.0	12.6	0.6
ICU	1/15/2023 16:00	9	12.0	10.4	-1.6
ICU	1/15/2023 17:00	9	12.0	12.6	0.6
ICU	1/15/2023 18:00	9	12.0	12.6	0.6
ICU	1/15/2023 19:00	9	12.0	12.6	0.6
ICU	1/15/2023 20:00	9	10.0	11.0	1.0
ICU	1/15/2023 21:00	9	10.0	11.0	1.0
ICU	1/15/2023 22:00	9	10.0	11.0	1.0
ICU	1/15/2023 23:00	9	10.0	10.4	0.4
ICU	1/16/2023 0:00	9	10.0	10.3	0.2
ICU	1/16/2023 1:00	9	10.0	11.9	1.9
ICU	1/16/2023 2:00	9	10.0	11.9	1.9

ICU	1/16/2023 3:00	9	10.0	11.9	1.9
ICU	1/16/2023 4:00	9	10.0	11.9	1.9
ICU	1/16/2023 5:00	9	10.0	11.9	1.9
ICU	1/16/2023 6:00	9	10.0	11.9	1.9
ICU	1/16/2023 7:00	9	10.0	11.9	1.9
ICU	1/16/2023 8:00	9	10.0	10.0	0.0
ICU	1/16/2023 9:00	9	10.0	10.4	0.4
ICU	1/16/2023 10:00	9	10.0	10.4	0.4
ICU	1/16/2023 11:00	9	10.0	10.4	0.4
ICU	1/16/2023 12:00	9	10.0	10.4	0.4
ICU	1/16/2023 13:00	9	10.0	10.4	0.4
ICU	1/16/2023 14:00	8	10.0	10.4	0.4
ICU	1/16/2023 15:00	9	10.0	10.4	0.4
ICU	1/16/2023 16:00	8	10.0	8.9	-1.1
ICU	1/16/2023 17:00	8	10.0	8.9	-1.1
ICU	1/16/2023 18:00	8	10.0	8.9	-1.1
ICU	1/16/2023 19:00	8	10.0	8.9	-1.1
ICU	1/16/2023 20:00	9	12.0	13.3	1.3
ICU	1/16/2023 21:00	9	12.0	12.2	0.2
ICU	1/16/2023 22:00	9	12.0	13.3	1.3
ICU	1/16/2023 23:00	9	12.0	13.3	1.3
ICU	1/17/2023 0:00	9	12.0	11.9	-0.2
ICU	1/17/2023 1:00	9	12.0	11.9	-0.2
ICU	1/17/2023 2:00	9	12.0	11.9	-0.2
ICU	1/17/2023 3:00	9	12.0	11.9	-0.2
ICU	1/17/2023 4:00	9	12.0	11.9	-0.2
ICU	1/17/2023 5:00	9	12.0	11.9	-0.2
ICU	1/17/2023 6:00	9	12.0	11.9	-0.2
ICU	1/17/2023 7:00	9	12.0	11.9	-0.2
ICU	1/17/2023 8:00	9	12.0	11.0	-1.0
ICU	1/17/2023 9:00	9	12.0	11.0	-1.0
ICU	1/17/2023 10:00	9	12.0	11.0	-1.0
ICU	1/17/2023 11:00	9	12.0	11.0	-1.0
ICU	1/17/2023 12:00	9	12.0	12.6	0.6
ICU	1/17/2023 13:00	9	12.0	12.6	0.6
ICU	1/17/2023 14:00	9	12.0	12.6	0.6
ICU	1/17/2023 15:00	9	12.0	12.6	0.6
ICU	1/17/2023 16:00	9	12.0	10.4	-1.6
ICU	1/17/2023 17:00	9	12.0	10.4	-1.6
ICU	1/17/2023 18:00	9	12.0	10.4	-1.6
ICU	1/17/2023 19:00	9	12.0	10.4	-1.6
ICU	1/17/2023 20:00	7	10.0	12.6	2.6
ICU	1/17/2023 21:00	7	10.0	11.5	1.5
ICU	1/17/2023 22:00	7	10.0	12.6	2.6
ICU	1/17/2023 23:00	7	10.0	12.6	2.6
ICU	1/18/2023 0:00	7	10.0	13.3	3.3
ICU	1/18/2023 1:00	7	10.0	13.3	3.3

ICU	1/18/2023 2:00	7	10.0	13.3	3.3
ICU	1/18/2023 3:00	7	10.0	13.3	3.3
ICU	1/18/2023 4:00	7	10.0	13.3	3.3
ICU	1/18/2023 5:00	7	10.0	13.3	3.3
ICU	1/18/2023 6:00	7	10.0	13.3	3.3
ICU	1/18/2023 7:00	7	10.0	13.3	3.3
ICU	1/18/2023 8:00	7	10.0	14.2	4.2
ICU	1/18/2023 9:00	7	10.0	14.2	4.2
ICU	1/18/2023 10:00	7	10.0	14.2	4.2
ICU	1/18/2023 11:00	7	10.0	14.2	4.2
ICU	1/18/2023 12:00	7	10.0	15.0	5.0
ICU	1/18/2023 13:00	7	10.0	15.0	5.0
ICU	1/18/2023 14:00	7	10.0	15.0	5.0
ICU	1/18/2023 15:00	7	10.0	15.0	5.0
ICU	1/18/2023 16:00	7	10.0	11.9	1.9
ICU	1/18/2023 17:00	7	10.0	11.9	1.9
ICU	1/18/2023 18:00	7	10.0	11.9	1.9
ICU	1/18/2023 19:00	7	10.0	11.9	1.9
ICU	1/18/2023 20:00	7	12.0	11.9	-0.2
ICU	1/18/2023 21:00	7	12.0	10.7	-1.3
ICU	1/18/2023 22:00	7	12.0	11.9	-0.2
ICU	1/18/2023 23:00	7	12.0	11.9	-0.2
ICU	1/19/2023 0:00	7	12.0	14.1	2.1
ICU	1/19/2023 1:00	7	12.0	14.8	2.8
ICU	1/19/2023 2:00	7	12.0	14.8	2.8
ICU	1/19/2023 3:00	7	12.0	14.8	2.8
ICU	1/19/2023 4:00	7	12.0	14.8	2.8
ICU	1/19/2023 5:00	7	12.0	14.8	2.8
ICU	1/19/2023 6:00	7	12.0	14.8	2.8
ICU	1/19/2023 7:00	7	12.0	14.8	2.8
ICU	1/19/2023 8:00	7	12.0	9.5	-2.5
ICU	1/19/2023 9:00	7	12.0	9.5	-2.5
ICU	1/19/2023 10:00	7	12.0	9.5	-2.5
ICU	1/19/2023 11:00	7	12.0	9.5	-2.5
ICU	1/19/2023 12:00	7	12.0	12.6	0.6
ICU	1/19/2023 13:00	7	12.0	12.6	0.6
ICU	1/19/2023 14:00	7	12.0	12.6	0.6
ICU	1/19/2023 15:00	7	12.0	12.6	0.6
ICU	1/19/2023 16:00	7	12.0	8.9	-3.1
ICU	1/19/2023 17:00	7	12.0	8.9	-3.1
ICU	1/19/2023 18:00	7	12.0	8.9	-3.1
ICU	1/19/2023 19:00	7	12.0	9.6	-2.4
ICU	1/19/2023 20:00	9	12.0	12.6	0.6
ICU	1/19/2023 21:00	9	12.0	12.6	0.6
ICU	1/19/2023 22:00	9	12.0	12.6	0.6
ICU	1/19/2023 23:00	9	12.0	12.6	0.6
ICU	1/20/2023 0:00	10	12.0	12.6	0.6

ICU	1/20/2023 1:00	10	12.0	12.6	0.6
ICU	1/20/2023 2:00	10	12.0	12.6	0.6
ICU	1/20/2023 3:00	10	12.0	12.6	0.6
ICU	1/20/2023 4:00	10	10.0	12.6	2.6
ICU	1/20/2023 5:00	10	10.0	12.6	2.6
ICU	1/20/2023 6:00	10	10.0	12.6	2.6
ICU	1/20/2023 7:00	10	10.0	12.6	2.6
ICU	1/20/2023 8:00	10	10.0	12.6	2.6
ICU	1/20/2023 9:00	9	10.0	12.6	2.6
ICU	1/20/2023 10:00	9	10.0	12.6	2.6
ICU	1/20/2023 11:00	9	10.0	12.6	2.6
ICU	1/20/2023 12:00	9	10.0	12.6	2.6
ICU	1/20/2023 13:00	9	10.0	12.6	2.6
ICU	1/20/2023 14:00	9	10.0	12.6	2.6
ICU	1/20/2023 15:00	9	10.0	12.6	2.6
ICU	1/20/2023 16:00	8	10.0	11.0	1.0
ICU	1/20/2023 17:00	8	10.0	11.0	1.0
ICU	1/20/2023 18:00	8	10.0	11.0	1.0
ICU	1/20/2023 19:00	8	10.0	11.0	1.0
ICU	1/20/2023 20:00	9	12.0	11.0	-1.0
ICU	1/20/2023 21:00	9	12.0	11.0	-1.0
ICU	1/20/2023 22:00	9	12.0	11.0	-1.0
ICU	1/20/2023 23:00	9	12.0	11.0	-1.0
ICU	1/21/2023 0:00	9	12.0	10.4	-1.6
ICU	1/21/2023 1:00	9	12.0	10.4	-1.6
ICU	1/21/2023 2:00	9	12.0	10.4	-1.6
ICU	1/21/2023 3:00	9	12.0	10.4	-1.6
ICU	1/21/2023 4:00	9	12.0	10.4	-1.6
ICU	1/21/2023 5:00	9	12.0	10.4	-1.6
ICU	1/21/2023 6:00	9	12.0	10.4	-1.6
ICU	1/21/2023 7:00	9	12.0	10.4	-1.6
ICU	1/21/2023 8:00	9	12.0	12.6	0.6
ICU	1/21/2023 9:00	9	12.0	12.6	0.6
ICU	1/21/2023 10:00	9	12.0	12.6	0.6
ICU	1/21/2023 11:00	9	12.0	12.6	0.6
ICU	1/21/2023 12:00	10	12.0	14.2	2.2
ICU	1/21/2023 13:00	10	12.0	14.2	2.2
ICU	1/21/2023 14:00	9	12.0	14.2	2.2
ICU	1/21/2023 15:00	9	12.0	14.2	2.2
ICU	1/21/2023 16:00	9	12.0	9.5	-2.5
ICU	1/21/2023 17:00	10	12.0	9.5	-2.5
ICU	1/21/2023 18:00	10	12.0	9.5	-2.5
ICU	1/21/2023 19:00	10	12.0	9.5	-2.5
ICU	1/21/2023 20:00	11	12.0	12.6	0.6
ICU	1/21/2023 21:00	11	12.0	12.6	0.6
ICU	1/21/2023 22:00	11	12.0	12.6	0.6
ICU	1/21/2023 23:00	12	12.0	11.5	-0.5

ICU	1/22/2023 0:00	12	12.0	12.6	0.6
ICU	1/22/2023 1:00	12	12.0	12.6	0.6
ICU	1/22/2023 2:00	12	12.0	12.6	0.6
ICU	1/22/2023 3:00	12	12.0	12.6	0.6
ICU	1/22/2023 4:00	12	12.0	12.6	0.6
ICU	1/22/2023 5:00	12	12.0	12.6	0.6
ICU	1/22/2023 6:00	12	12.0	12.6	0.6
ICU	1/22/2023 7:00	12	12.0	12.6	0.6
ICU	1/22/2023 8:00	12	12.0	12.6	0.6
ICU	1/22/2023 9:00	12	12.0	12.6	0.6
ICU	1/22/2023 10:00	12	12.0	12.6	0.6
ICU	1/22/2023 11:00	11	12.0	12.6	0.6
ICU	1/22/2023 12:00	11	12.0	12.6	0.6
ICU	1/22/2023 13:00	10	12.0	12.6	0.6
ICU	1/22/2023 14:00	10	12.0	12.6	0.6
ICU	1/22/2023 15:00	10	12.0	11.0	-1.0
ICU	1/22/2023 16:00	10	12.0	9.5	-2.5
ICU	1/22/2023 17:00	10	12.0	9.5	-2.5
ICU	1/22/2023 18:00	9	12.0	9.5	-2.5
ICU	1/22/2023 19:00	9	12.0	9.5	-2.5
ICU	1/22/2023 20:00	9	10.0	11.0	1.0
ICU	1/22/2023 21:00	9	10.0	11.0	1.0
ICU	1/22/2023 22:00	9	10.0	11.0	1.0
ICU	1/22/2023 23:00	9	10.0	11.0	1.0
ICU	1/23/2023 0:00	9	10.0	10.4	0.4
ICU	1/23/2023 1:00	9	10.0	10.4	0.4
ICU	1/23/2023 2:00	9	10.0	10.4	0.4
ICU	1/23/2023 3:00	9	10.0	10.4	0.4
ICU	1/23/2023 4:00	9	10.0	10.4	0.4
ICU	1/23/2023 5:00	9	10.0	10.4	0.4
ICU	1/23/2023 6:00	9	10.0	10.4	0.4
ICU	1/23/2023 7:00	9	10.0	10.4	0.4
ICU	1/23/2023 8:00	9	10.0	6.3	-3.7
ICU	1/23/2023 9:00	9	10.0	6.3	-3.7
ICU	1/23/2023 10:00	9	10.0	6.3	-3.7
ICU	1/23/2023 11:00	9	10.0	6.3	-3.7
ICU	1/23/2023 12:00	9	10.0	6.3	-3.7
ICU	1/23/2023 13:00	9	10.0	6.3	-3.7
ICU	1/23/2023 14:00	9	10.0	6.3	-3.7
ICU	1/23/2023 15:00	9	10.0	6.3	-3.7
ICU	1/23/2023 16:00	9	10.0	6.3	-3.7
ICU	1/23/2023 17:00	9	10.0	6.3	-3.7
ICU	1/23/2023 18:00	9	10.0	6.3	-3.7
ICU	1/23/2023 19:00	9	10.0	6.3	-3.7
ICU	1/23/2023 20:00	9	11.0	9.5	-1.5
ICU	1/23/2023 21:00	10	11.0	9.5	-1.5
ICU	1/23/2023 22:00	10	11.0	9.5	-1.5

ICU	1/23/2023 23:00	10	11.0	9.5	-1.5
ICU	1/24/2023 0:00	10	11.0	9.5	-1.5
ICU	1/24/2023 1:00	10	11.0	9.5	-1.5
ICU	1/24/2023 2:00	11	11.0	9.5	-1.5
ICU	1/24/2023 3:00	11	11.0	9.5	-1.5
ICU	1/24/2023 4:00	11	11.0	9.5	-1.5
ICU	1/24/2023 5:00	11	11.0	9.5	-1.5
ICU	1/24/2023 6:00	11	11.0	9.5	-1.5
ICU	1/24/2023 7:00	11	11.0	9.5	-1.5
ICU	1/24/2023 8:00	11	11.0	8.7	-2.3
ICU	1/24/2023 9:00	11	11.0	9.5	-1.5
ICU	1/24/2023 10:00	11	11.0	9.5	-1.5
ICU	1/24/2023 11:00	11	11.0	9.5	-1.5
ICU	1/24/2023 12:00	11	11.0	9.5	-1.5
ICU	1/24/2023 13:00	11	11.0	9.5	-1.5
ICU	1/24/2023 14:00	11	11.0	9.5	-1.5
ICU	1/24/2023 15:00	11	11.0	9.5	-1.5
ICU	1/24/2023 16:00	11	11.0	7.9	-3.1
ICU	1/24/2023 17:00	11	11.0	7.9	-3.1
ICU	1/24/2023 18:00	11	11.0	7.9	-3.1
ICU	1/24/2023 19:00	11	11.0	7.9	-3.1
ICU	1/24/2023 20:00	9	12.0	7.9	-4.1
ICU	1/24/2023 21:00	9	12.0	7.9	-4.1
ICU	1/24/2023 22:00	9	12.0	7.9	-4.1
ICU	1/24/2023 23:00	9	12.0	7.9	-4.1
ICU	1/25/2023 0:00	9	12.0	7.9	-4.1
ICU	1/25/2023 1:00	9	12.0	7.9	-4.1
ICU	1/25/2023 2:00	9	12.0	7.9	-4.1
ICU	1/25/2023 3:00	9	12.0	7.9	-4.1
ICU	1/25/2023 4:00	9	12.0	7.9	-4.1
ICU	1/25/2023 5:00	9	12.0	7.9	-4.1
ICU	1/25/2023 6:00	9	12.0	7.9	-4.1
ICU	1/25/2023 7:00	9	12.0	7.9	-4.1
ICU	1/25/2023 8:00	9	12.0	9.5	-2.5
ICU	1/25/2023 9:00	9	12.0	9.5	-2.5
ICU	1/25/2023 10:00	9	12.0	9.5	-2.5
ICU	1/25/2023 11:00	9	12.0	9.5	-2.5
ICU	1/25/2023 12:00	10	10.0	9.5	-0.5
ICU	1/25/2023 13:00	10	10.0	9.5	-0.5
ICU	1/25/2023 14:00	10	10.0	8.4	-1.7
ICU	1/25/2023 15:00	10	10.0	9.5	-0.5
ICU	1/25/2023 16:00	10	10.0	6.3	-3.7
ICU	1/25/2023 17:00	9	12.0	7.9	-4.1
ICU	1/25/2023 18:00	9	12.0	6.8	-5.2
ICU	1/25/2023 19:00	9	12.0	7.9	-4.1
ICU	1/25/2023 20:00	9	12.0	9.5	-2.5
ICU	1/25/2023 21:00	9	12.0	9.5	-2.5

ICU	1/25/2023 22:00	9	12.0	9.5	-2.5
ICU	1/25/2023 23:00	9	12.0	9.5	-2.5
ICU	1/26/2023 0:00	9	12.0	8.9	-3.1
ICU	1/26/2023 1:00	9	12.0	8.9	-3.1
ICU	1/26/2023 2:00	9	12.0	8.9	-3.1
ICU	1/26/2023 3:00	9	12.0	8.9	-3.1
ICU	1/26/2023 4:00	9	12.0	8.9	-3.1
ICU	1/26/2023 5:00	9	12.0	8.9	-3.1
ICU	1/26/2023 6:00	10	12.0	8.9	-3.1
ICU	1/26/2023 7:00	10	12.0	8.9	-3.1
ICU	1/26/2023 8:00	10	12.0	12.6	0.6
ICU	1/26/2023 9:00	10	12.0	12.6	0.6
ICU	1/26/2023 10:00	10	12.0	12.6	0.6
ICU	1/26/2023 11:00	10	12.0	12.6	0.6
ICU	1/26/2023 12:00	10	12.0	12.6	0.6
ICU	1/26/2023 13:00	10	12.0	12.6	0.6
ICU	1/26/2023 14:00	10	12.0	12.6	0.6
ICU	1/26/2023 15:00	10	12.0	12.6	0.6
ICU	1/26/2023 16:00	10	10.0	10.4	0.4
ICU	1/26/2023 17:00	10	10.0	10.4	0.4
ICU	1/26/2023 18:00	10	10.0	10.4	0.4
ICU	1/26/2023 19:00	10	10.0	10.4	0.4
ICU	1/26/2023 20:00	10	11.0	10.4	-0.6
ICU	1/26/2023 21:00	10	11.0	10.4	-0.6
ICU	1/26/2023 22:00	10	11.0	10.4	-0.6
ICU	1/26/2023 23:00	10	11.0	10.4	-0.6
ICU	1/27/2023 0:00	10	11.0	10.4	-0.6
ICU	1/27/2023 1:00	10	11.0	8.9	-2.1
ICU	1/27/2023 2:00	10	11.0	8.9	-2.1
ICU	1/27/2023 3:00	10	11.0	8.9	-2.1
ICU	1/27/2023 4:00	10	11.0	8.9	-2.1
ICU	1/27/2023 5:00	10	11.0	8.9	-2.1
ICU	1/27/2023 6:00	10	11.0	8.9	-2.1
ICU	1/27/2023 7:00	10	11.0	8.9	-2.1
ICU	1/27/2023 8:00	10	13.0	10.4	-2.6
ICU	1/27/2023 9:00	10	13.0	10.4	-2.6
ICU	1/27/2023 10:00	10	13.0	10.4	-2.6
ICU	1/27/2023 11:00	10	13.0	10.4	-2.6
ICU	1/27/2023 12:00	10	13.0	10.4	-2.6
ICU	1/27/2023 13:00	10	13.0	10.4	-2.6
ICU	1/27/2023 14:00	12	13.0	8.2	-4.9
ICU	1/27/2023 15:00	10	13.0	8.2	-4.9
ICU	1/27/2023 16:00	10	13.0	11.0	-2.0
ICU	1/27/2023 17:00	10	13.0	11.0	-2.0
ICU	1/27/2023 18:00	9	13.0	9.9	-3.1
ICU	1/27/2023 19:00	9	13.0	11.0	-2.0
ICU	1/27/2023 20:00	9	13.0	11.0	-2.0

ICU	1/27/2023 21:00	11	13.0	11.0	-2.0
ICU	1/27/2023 22:00	11	13.0	11.0	-2.0
ICU	1/27/2023 23:00	11	13.0	10.4	-2.6
ICU	1/28/2023 0:00	11	13.0	8.9	-4.1
ICU	1/28/2023 1:00	11	13.0	8.9	-4.1
ICU	1/28/2023 2:00	11	13.0	8.9	-4.1
ICU	1/28/2023 3:00	11	13.0	8.9	-4.1
ICU	1/28/2023 4:00	11	13.0	8.9	-4.1
ICU	1/28/2023 5:00	11	13.0	8.9	-4.1
ICU	1/28/2023 6:00	11	13.0	8.9	-4.1
ICU	1/28/2023 7:00	11	13.0	8.9	-4.1
ICU	1/28/2023 8:00	11	13.0	10.4	-2.6
ICU	1/28/2023 9:00	11	13.0	10.4	-2.6
ICU	1/28/2023 10:00	10	13.0	9.3	-3.7
ICU	1/28/2023 11:00	10	13.0	10.4	-2.6
ICU	1/28/2023 12:00	10	13.0	10.4	-2.6
ICU	1/28/2023 13:00	10	13.0	10.4	-2.6
ICU	1/28/2023 14:00	10	13.0	10.4	-2.6
ICU	1/28/2023 15:00	10	13.0	8.2	-4.9
ICU	1/28/2023 16:00	9	13.0	9.3	-3.7
ICU	1/28/2023 17:00	9	13.0	10.4	-2.6
ICU	1/28/2023 18:00	11	13.0	8.2	-4.9
ICU	1/28/2023 19:00	11	13.0	10.4	-2.6
ICU	1/28/2023 20:00	11	13.0	11.9	-1.2
ICU	1/28/2023 21:00	11	13.0	11.9	-1.2
ICU	1/28/2023 22:00	11	13.0	11.9	-1.2
ICU	1/28/2023 23:00	11	13.0	11.9	-1.2
ICU	1/29/2023 0:00	11	13.0	10.4	-2.6
ICU	1/29/2023 1:00	11	13.0	10.4	-2.6
ICU	1/29/2023 2:00	12	12.0	10.4	-1.6
ICU	1/29/2023 3:00	12	12.0	10.4	-1.6
ICU	1/29/2023 4:00	12	12.0	10.4	-1.6
ICU	1/29/2023 5:00	12	12.0	10.4	-1.6
ICU	1/29/2023 6:00	12	12.0	10.4	-1.6
ICU	1/29/2023 7:00	12	12.0	10.4	-1.6
ICU	1/29/2023 8:00	12	12.0	7.4	-4.6
ICU	1/29/2023 9:00	12	12.0	7.4	-4.6
ICU	1/29/2023 10:00	12	12.0	8.2	-3.9
ICU	1/29/2023 11:00	12	12.0	8.9	-3.1
ICU	1/29/2023 12:00	12	12.0	10.4	-1.6
ICU	1/29/2023 13:00	12	12.0	10.4	-1.6
ICU	1/29/2023 14:00	12	12.0	10.4	-1.6
ICU	1/29/2023 15:00	12	12.0	10.4	-1.6
ICU	1/29/2023 16:00	12	12.0	8.9	-3.1
ICU	1/29/2023 17:00	12	12.0	8.9	-3.1
ICU	1/29/2023 18:00	12	12.0	8.9	-3.1
ICU	1/29/2023 19:00	12	12.0	8.9	-3.1

ICU	1/29/2023 20:00	11	12.0	11.0	-1.0
ICU	1/29/2023 21:00	11	12.0	11.0	-1.0
ICU	1/29/2023 22:00	11	12.0	11.0	-1.0
ICU	1/29/2023 23:00	11	12.0	11.0	-1.0
ICU	1/30/2023 0:00	12	12.0	11.0	-1.0
ICU	1/30/2023 1:00	12	12.0	11.0	-1.0
ICU	1/30/2023 2:00	12	12.0	11.0	-1.0
ICU	1/30/2023 3:00	12	12.0	11.0	-1.0
ICU	1/30/2023 4:00	12	12.0	11.0	-1.0
ICU	1/30/2023 5:00	12	12.0	11.0	-1.0
ICU	1/30/2023 6:00	12	12.0	11.0	-1.0
ICU	1/30/2023 7:00	12	12.0	11.0	-1.0
ICU	1/30/2023 8:00	12	12.0	12.6	0.6
ICU	1/30/2023 9:00	12	12.0	12.6	0.6
ICU	1/30/2023 10:00	12	12.0	12.6	0.6
ICU	1/30/2023 11:00	12	12.0	12.6	0.6
ICU	1/30/2023 12:00	12	12.0	14.2	2.2
ICU	1/30/2023 13:00	12	12.0	14.2	2.2
ICU	1/30/2023 14:00	10	12.0	14.2	2.2
ICU	1/30/2023 15:00	10	12.0	14.2	2.2
ICU	1/30/2023 16:00	10	12.0	11.0	-1.0
ICU	1/30/2023 17:00	10	12.0	11.0	-1.0
ICU	1/30/2023 18:00	10	12.0	11.0	-1.0
ICU	1/30/2023 19:00	10	12.0	11.0	-1.0
ICU	1/30/2023 20:00	10	12.0	12.6	0.6
ICU	1/30/2023 21:00	10	12.0	12.6	0.6
ICU	1/30/2023 22:00	10	10.0	9.5	-0.5
ICU	1/30/2023 23:00	10	10.0	9.5	-0.5
ICU	1/31/2023 0:00	10	10.0	8.9	-1.1
ICU	1/31/2023 1:00	10	10.0	8.9	-1.1
ICU	1/31/2023 2:00	10	10.0	8.9	-1.1
ICU	1/31/2023 3:00	10	10.0	8.9	-1.1
ICU	1/31/2023 4:00	10	10.0	8.9	-1.1
ICU	1/31/2023 5:00	10	10.0	8.9	-1.1
ICU	1/31/2023 6:00	10	10.0	8.9	-1.1
ICU	1/31/2023 7:00	10	10.0	8.9	-1.1
ICU	1/31/2023 8:00	12	12.0	12.6	0.6
ICU	1/31/2023 9:00	12	12.0	12.6	0.6
ICU	1/31/2023 10:00	12	12.0	12.6	0.6
ICU	1/31/2023 11:00	12	12.0	12.6	0.6
ICU	1/31/2023 12:00	12	12.0	12.6	0.6
ICU	1/31/2023 13:00	12	12.0	12.6	0.6
ICU	1/31/2023 14:00	12	12.0	12.6	0.6
ICU	1/31/2023 15:00	10	12.0	12.6	0.6
ICU	1/31/2023 16:00	9	12.0	11.0	-1.0
ICU	1/31/2023 17:00	8	12.0	11.0	-1.0
ICU	1/31/2023 18:00	8	12.0	11.0	-1.0

ICU	1/31/2023 19:00	8	12.0	11.0	-1.0
ICU	1/31/2023 20:00	9	10.0	8.9	-1.1
ICU	1/31/2023 21:00	9	10.0	7.8	-2.2
ICU	1/31/2023 22:00	9	10.0	8.9	-1.1
ICU	1/31/2023 23:00	9	10.0	7.8	-2.2
ICU	2/1/2023 0:00	9	10.0	10.4	0.4
ICU	2/1/2023 1:00	9	10.0	10.4	0.4
ICU	2/1/2023 2:00	9	10.0	10.4	0.4
ICU	2/1/2023 3:00	9	10.0	10.4	0.4
ICU	2/1/2023 4:00	9	10.0	10.4	0.4
ICU	2/1/2023 5:00	9	10.0	10.4	0.4
ICU	2/1/2023 6:00	9	10.0	10.4	0.4
ICU	2/1/2023 7:00	9	10.0	10.4	0.4
ICU	2/1/2023 8:00	11	12.0	12.6	0.6
ICU	2/1/2023 9:00	11	12.0	12.6	0.6
ICU	2/1/2023 10:00	11	12.0	12.6	0.6
ICU	2/1/2023 11:00	11	12.0	12.6	0.6
ICU	2/1/2023 12:00	11	12.0	14.2	2.2
ICU	2/1/2023 13:00	11	12.0	14.2	2.2
ICU	2/1/2023 14:00	11	12.0	14.2	2.2
ICU	2/1/2023 15:00	11	12.0	14.2	2.2
ICU	2/1/2023 16:00	11	12.0	11.9	-0.2
ICU	2/1/2023 17:00	11	12.0	11.1	-0.9
ICU	2/1/2023 18:00	11	12.0	10.4	-1.6
ICU	2/1/2023 19:00	11	12.0	10.4	-1.6
ICU	2/1/2023 20:00	11	12.0	11.0	-1.0
ICU	2/1/2023 21:00	11	12.0	11.0	-1.0
ICU	2/1/2023 22:00	9	10.0	7.7	-2.3
ICU	2/1/2023 23:00	9	10.0	11.0	1.0
ICU	2/2/2023 0:00	10	12.0	12.6	0.6
ICU	2/2/2023 1:00	10	12.0	12.6	0.6
ICU	2/2/2023 2:00	10	12.0	12.6	0.6
ICU	2/2/2023 3:00	10	12.0	12.6	0.6
ICU	2/2/2023 4:00	10	12.0	12.6	0.6
ICU	2/2/2023 5:00	10	12.0	12.6	0.6
ICU	2/2/2023 6:00	10	12.0	12.6	0.6
ICU	2/2/2023 7:00	10	12.0	12.6	0.6
ICU	2/2/2023 8:00	10	12.0	12.6	0.6
ICU	2/2/2023 9:00	10	12.0	12.6	0.6
ICU	2/2/2023 10:00	10	12.0	12.6	0.6
ICU	2/2/2023 11:00	10	12.0	12.6	0.6
ICU	2/2/2023 12:00	10	12.0	12.6	0.6
ICU	2/2/2023 13:00	10	12.0	12.6	0.6
ICU	2/2/2023 14:00	10	12.0	12.6	0.6
ICU	2/2/2023 15:00	10	12.0	12.6	0.6
ICU	2/2/2023 16:00	10	12.0	11.9	-0.2
ICU	2/2/2023 17:00	10	12.0	11.9	-0.2

ICU	2/2/2023 18:00	10	12.0	11.9	-0.2
ICU	2/2/2023 19:00	10	12.0	11.9	-0.2
ICU	2/2/2023 20:00	10	10.0	9.9	-0.1
ICU	2/2/2023 21:00	10	10.0	11.0	1.0
ICU	2/2/2023 22:00	10	10.0	11.0	1.0
ICU	2/2/2023 23:00	10	10.0	11.0	1.0
ICU	2/3/2023 0:00	10	10.0	11.0	1.0
ICU	2/3/2023 1:00	10	10.0	11.0	1.0
ICU	2/3/2023 2:00	10	10.0	11.0	1.0
ICU	2/3/2023 3:00	10	10.0	11.0	1.0
ICU	2/3/2023 4:00	10	10.0	11.0	1.0
ICU	2/3/2023 5:00	10	10.0	11.0	1.0
ICU	2/3/2023 6:00	10	10.0	11.0	1.0
ICU	2/3/2023 7:00	10	10.0	11.0	1.0
ICU	2/3/2023 8:00	10	10.0	14.2	4.2
ICU	2/3/2023 9:00	11	12.0	14.2	2.2
ICU	2/3/2023 10:00	11	12.0	14.2	2.2
ICU	2/3/2023 11:00	11	12.0	14.2	2.2
ICU	2/3/2023 12:00	11	12.0	14.2	2.2
ICU	2/3/2023 13:00	11	12.0	14.2	2.2
ICU	2/3/2023 14:00	11	12.0	14.2	2.2
ICU	2/3/2023 15:00	11	12.0	14.2	2.2
ICU	2/3/2023 16:00	11	12.0	14.2	2.2
ICU	2/3/2023 17:00	11	12.0	14.2	2.2
ICU	2/3/2023 18:00	11	12.0	14.2	2.2
ICU	2/3/2023 19:00	11	12.0	14.2	2.2
ICU	2/3/2023 20:00	11	12.0	12.6	0.6
ICU	2/3/2023 21:00	11	12.0	12.6	0.6
ICU	2/3/2023 22:00	11	12.0	12.6	0.6
ICU	2/3/2023 23:00	11	12.0	12.6	0.6
ICU	2/4/2023 0:00	11	12.0	12.6	0.6
ICU	2/4/2023 1:00	11	12.0	10.4	-1.6
ICU	2/4/2023 2:00	11	12.0	12.6	0.6
ICU	2/4/2023 3:00	11	12.0	12.6	0.6
ICU	2/4/2023 4:00	11	12.0	12.6	0.6
ICU	2/4/2023 5:00	11	12.0	12.6	0.6
ICU	2/4/2023 6:00	11	12.0	12.6	0.6
ICU	2/4/2023 7:00	11	12.0	12.6	0.6
ICU	2/4/2023 8:00	11	12.0	14.2	2.2
ICU	2/4/2023 9:00	11	12.0	14.2	2.2
ICU	2/4/2023 10:00	11	12.0	14.2	2.2
ICU	2/4/2023 11:00	10	10.0	12.6	2.6
ICU	2/4/2023 12:00	10	10.0	14.2	4.2
ICU	2/4/2023 13:00	10	10.0	14.2	4.2
ICU	2/4/2023 14:00	10	10.0	14.2	4.2
ICU	2/4/2023 15:00	10	10.0	14.2	4.2
ICU	2/4/2023 16:00	10	10.0	11.0	1.0

ICU	2/4/2023 17:00	10	10.0	11.0	1.0
ICU	2/4/2023 18:00	10	10.0	11.0	1.0
ICU	2/4/2023 19:00	10	10.0	11.0	1.0
ICU	2/4/2023 20:00	10	12.0	11.9	-0.2
ICU	2/4/2023 21:00	10	12.0	11.9	-0.2
ICU	2/4/2023 22:00	10	12.0	11.9	-0.2
ICU	2/4/2023 23:00	10	12.0	11.9	-0.2
ICU	2/5/2023 0:00	10	12.0	11.9	-0.2
ICU	2/5/2023 1:00	10	12.0	11.9	-0.2
ICU	2/5/2023 2:00	10	12.0	11.9	-0.2
ICU	2/5/2023 3:00	10	12.0	11.9	-0.2
ICU	2/5/2023 4:00	10	12.0	11.9	-0.2
ICU	2/5/2023 5:00	10	12.0	11.9	-0.2
ICU	2/5/2023 6:00	11	12.0	11.9	-0.2
ICU	2/5/2023 7:00	11	12.0	11.9	-0.2
ICU	2/5/2023 8:00	11	12.0	14.2	2.2
ICU	2/5/2023 9:00	11	12.0	14.2	2.2
ICU	2/5/2023 10:00	11	12.0	14.2	2.2
ICU	2/5/2023 11:00	10	12.0	14.2	2.2
ICU	2/5/2023 12:00	10	12.0	14.2	2.2
ICU	2/5/2023 13:00	10	12.0	14.2	2.2
ICU	2/5/2023 14:00	10	12.0	14.2	2.2
ICU	2/5/2023 15:00	9	12.0	14.2	2.2
ICU	2/5/2023 16:00	8	12.0	11.9	-0.2
ICU	2/5/2023 17:00	8	12.0	11.9	-0.2
ICU	2/5/2023 18:00	8	12.0	11.9	-0.2
ICU	2/5/2023 19:00	8	12.0	11.9	-0.2
ICU	2/5/2023 20:00	7	13.0	8.9	-4.1
ICU	2/5/2023 21:00	7	13.0	8.9	-4.1
ICU	2/5/2023 22:00	7	13.0	8.9	-4.1
ICU	2/5/2023 23:00	7	13.0	8.9	-4.1
ICU	2/6/2023 0:00	7	13.0	8.9	-4.1
ICU	2/6/2023 1:00	7	13.0	8.9	-4.1
ICU	2/6/2023 2:00	7	13.0	8.9	-4.1
ICU	2/6/2023 3:00	7	13.0	8.9	-4.1
ICU	2/6/2023 4:00	7	13.0	8.9	-4.1
ICU	2/6/2023 5:00	7	13.0	8.9	-4.1
ICU	2/6/2023 6:00	7	13.0	8.9	-4.1
ICU	2/6/2023 7:00	7	13.0	8.9	-4.1
ICU	2/6/2023 8:00	7	13.0	8.9	-4.1
ICU	2/6/2023 9:00	8	13.0	8.9	-4.1
ICU	2/6/2023 10:00	9	13.0	8.9	-4.1
ICU	2/6/2023 11:00	10	13.0	8.9	-4.1
ICU	2/6/2023 12:00	9	13.0	10.4	-2.6
ICU	2/6/2023 13:00	9	13.0	10.4	-2.6
ICU	2/6/2023 14:00	8	13.0	10.4	-2.6
ICU	2/6/2023 15:00	9	13.0	10.4	-2.6

ICU	2/6/2023 16:00	9	13.0	8.9	-4.1
ICU	2/6/2023 17:00	8	13.0	8.9	-4.1
ICU	2/6/2023 18:00	8	13.0	8.9	-4.1
ICU	2/6/2023 19:00	8	13.0	8.9	-4.1
ICU	2/6/2023 20:00	8	12.0	11.0	-1.0
ICU	2/6/2023 21:00	8	12.0	11.0	-1.0
ICU	2/6/2023 22:00	8	12.0	11.0	-1.0
ICU	2/6/2023 23:00	8	12.0	11.0	-1.0
ICU	2/7/2023 0:00	8	12.0	10.4	-1.6
ICU	2/7/2023 1:00	8	12.0	10.4	-1.6
ICU	2/7/2023 2:00	8	12.0	10.4	-1.6
ICU	2/7/2023 3:00	8	12.0	10.4	-1.6
ICU	2/7/2023 4:00	8	12.0	10.4	-1.6
ICU	2/7/2023 5:00	8	12.0	10.4	-1.6
ICU	2/7/2023 6:00	8	12.0	10.4	-1.6
ICU	2/7/2023 7:00	9	12.0	10.4	-1.6
ICU	2/7/2023 8:00	9	12.0	12.6	0.6
ICU	2/7/2023 9:00	8	12.0	11.5	-0.5
ICU	2/7/2023 10:00	8	12.0	12.6	0.6
ICU	2/7/2023 11:00	8	12.0	12.6	0.6
ICU	2/7/2023 12:00	9	12.0	11.5	-0.5
ICU	2/7/2023 13:00	9	12.0	10.4	-1.6
ICU	2/7/2023 14:00	9	12.0	12.6	0.6
ICU	2/7/2023 15:00	9	12.0	10.4	-1.6
ICU	2/7/2023 16:00	9	12.0	11.9	-0.2
ICU	2/7/2023 17:00	9	12.0	11.9	-0.2
ICU	2/7/2023 18:00	9	12.0	11.9	-0.2
ICU	2/7/2023 19:00	8	12.0	10.7	-1.3
ICU	2/7/2023 20:00	9	12.0	12.2	0.2
ICU	2/7/2023 21:00	9	12.0	11.1	-0.9
ICU	2/7/2023 22:00	9	12.0	13.3	1.3
ICU	2/7/2023 23:00	9	12.0	13.3	1.3
ICU	2/8/2023 0:00	9	12.0	14.2	2.2
ICU	2/8/2023 1:00	9	12.0	14.2	2.2
ICU	2/8/2023 2:00	9	12.0	14.2	2.2
ICU	2/8/2023 3:00	9	12.0	14.2	2.2
ICU	2/8/2023 4:00	9	12.0	14.2	2.2
ICU	2/8/2023 5:00	10	12.0	14.2	2.2
ICU	2/8/2023 6:00	10	12.0	14.2	2.2
ICU	2/8/2023 7:00	10	12.0	14.2	2.2
ICU	2/8/2023 8:00	9	12.0	12.6	0.6
ICU	2/8/2023 9:00	9	12.0	12.6	0.6
ICU	2/8/2023 10:00	9	12.0	12.6	0.6
ICU	2/8/2023 11:00	9	12.0	12.6	0.6
ICU	2/8/2023 12:00	9	12.0	12.6	0.6
ICU	2/8/2023 13:00	8	12.0	11.5	-0.5
ICU	2/8/2023 14:00	8	12.0	12.6	0.6

ICU	2/8/2023 15:00	8	12.0	12.6	0.6
ICU	2/8/2023 16:00	9	12.0	10.7	-1.3
ICU	2/8/2023 17:00	8	12.0	10.7	-1.3
ICU	2/8/2023 18:00	9	12.0	10.7	-1.3
ICU	2/8/2023 19:00	9	12.0	11.9	-0.2
ICU	2/8/2023 20:00	9	13.0	10.4	-2.6
ICU	2/8/2023 21:00	9	13.0	10.4	-2.6
ICU	2/8/2023 22:00	9	13.0	10.4	-2.6
ICU	2/8/2023 23:00	9	13.0	10.4	-2.6
ICU	2/9/2023 0:00	9	13.0	11.9	-1.2
ICU	2/9/2023 1:00	9	13.0	11.9	-1.2
ICU	2/9/2023 2:00	9	13.0	11.9	-1.2
ICU	2/9/2023 3:00	9	13.0	11.9	-1.2
ICU	2/9/2023 4:00	9	13.0	11.9	-1.2
ICU	2/9/2023 5:00	9	13.0	11.9	-1.2
ICU	2/9/2023 6:00	9	13.0	11.9	-1.2
ICU	2/9/2023 7:00	9	13.0	11.9	-1.2
ICU	2/9/2023 8:00	9	13.0	11.0	-2.0
ICU	2/9/2023 9:00	9	13.0	11.0	-2.0
ICU	2/9/2023 10:00	9	13.0	11.0	-2.0
ICU	2/9/2023 11:00	9	13.0	11.0	-2.0
ICU	2/9/2023 12:00	9	13.0	12.6	-0.4
ICU	2/9/2023 13:00	9	13.0	12.6	-0.4
ICU	2/9/2023 14:00	8	13.0	14.2	1.2
ICU	2/9/2023 15:00	8	13.0	14.2	1.2
ICU	2/9/2023 16:00	8	13.0	11.9	-1.2
ICU	2/9/2023 17:00	7	13.0	11.9	-1.2
ICU	2/9/2023 18:00	8	10.0	11.9	1.9
ICU	2/9/2023 19:00	9	12.0	11.9	-0.2
ICU	2/9/2023 20:00	11	12.0	11.9	-0.2
ICU	2/9/2023 21:00	11	12.0	11.9	-0.2
ICU	2/9/2023 22:00	11	12.0	11.9	-0.2
ICU	2/9/2023 23:00	11	12.0	11.9	-0.2
ICU	2/10/2023 0:00	11	12.0	12.6	0.6
ICU	2/10/2023 1:00	11	12.0	12.6	0.6
ICU	2/10/2023 2:00	11	12.0	12.6	0.6
ICU	2/10/2023 3:00	11	12.0	12.6	0.6
ICU	2/10/2023 4:00	11	12.0	12.6	0.6
ICU	2/10/2023 5:00	11	12.0	12.6	0.6
ICU	2/10/2023 6:00	11	12.0	12.6	0.6
ICU	2/10/2023 7:00	11	12.0	12.6	0.6
ICU	2/10/2023 8:00	11	12.0	15.0	3.0
ICU	2/10/2023 9:00	11	12.0	15.0	3.0
ICU	2/10/2023 10:00	11	12.0	15.0	3.0
ICU	2/10/2023 11:00	11	12.0	15.0	3.0
ICU	2/10/2023 12:00	11	12.0	15.0	3.0
ICU	2/10/2023 13:00	11	12.0	15.0	3.0

ICU	2/10/2023 14:00	11	12.0	15.0	3.0
ICU	2/10/2023 15:00	11	12.0	15.0	3.0
ICU	2/10/2023 16:00	11	13.0	14.2	1.2
ICU	2/10/2023 17:00	11	13.0	14.2	1.2
ICU	2/10/2023 18:00	11	13.0	14.2	1.2
ICU	2/10/2023 19:00	11	13.0	14.2	1.2
ICU	2/10/2023 20:00	11	13.0	12.6	-0.4
ICU	2/10/2023 21:00	11	13.0	12.6	-0.4
ICU	2/10/2023 22:00	11	13.0	12.6	-0.4
ICU	2/10/2023 23:00	11	13.0	12.6	-0.4
ICU	2/11/2023 0:00	11	13.0	12.6	-0.4
ICU	2/11/2023 1:00	11	13.0	12.6	-0.4
ICU	2/11/2023 2:00	11	13.0	12.6	-0.4
ICU	2/11/2023 3:00	11	13.0	12.6	-0.4
ICU	2/11/2023 4:00	11	13.0	12.6	-0.4
ICU	2/11/2023 5:00	11	13.0	12.6	-0.4
ICU	2/11/2023 6:00	11	13.0	12.6	-0.4
ICU	2/11/2023 7:00	12	12.0	11.5	-0.5
ICU	2/11/2023 8:00	12	12.0	14.2	2.2
ICU	2/11/2023 9:00	12	12.0	14.2	2.2
ICU	2/11/2023 10:00	12	12.0	14.2	2.2
ICU	2/11/2023 11:00	12	12.0	14.2	2.2
ICU	2/11/2023 12:00	12	12.0	14.2	2.2
ICU	2/11/2023 13:00	10	12.0	14.2	2.2
ICU	2/11/2023 14:00	10	12.0	14.2	2.2
ICU	2/11/2023 15:00	10	12.0	14.2	2.2
ICU	2/11/2023 16:00	10	12.0	14.2	2.2
ICU	2/11/2023 17:00	10	12.0	14.2	2.2
ICU	2/11/2023 18:00	10	12.0	14.2	2.2
ICU	2/11/2023 19:00	10	12.0	14.2	2.2
ICU	2/11/2023 20:00	10	12.0	14.2	2.2
ICU	2/11/2023 21:00	10	12.0	14.2	2.2
ICU	2/11/2023 22:00	10	12.0	14.2	2.2
ICU	2/11/2023 23:00	11	12.0	13.1	1.1
ICU	2/12/2023 0:00	11	12.0	14.2	2.2
ICU	2/12/2023 1:00	11	12.0	14.2	2.2
ICU	2/12/2023 2:00	11	12.0	14.2	2.2
ICU	2/12/2023 3:00	11	12.0	14.2	2.2
ICU	2/12/2023 4:00	11	12.0	14.2	2.2
ICU	2/12/2023 5:00	11	12.0	14.2	2.2
ICU	2/12/2023 6:00	11	12.0	14.2	2.2
ICU	2/12/2023 7:00	11	12.0	14.2	2.2
ICU	2/12/2023 8:00	11	12.0	12.6	0.6
ICU	2/12/2023 9:00	11	12.0	12.6	0.6
ICU	2/12/2023 10:00	11	12.0	12.6	0.6
ICU	2/12/2023 11:00	11	12.0	12.6	0.6
ICU	2/12/2023 12:00	11	12.0	14.2	2.2

ICU	2/12/2023 13:00	11	12.0	14.2	2.2
ICU	2/12/2023 14:00	11	12.0	14.2	2.2
ICU	2/12/2023 15:00	11	12.0	14.2	2.2
ICU	2/12/2023 16:00	11	12.0	12.6	0.6
ICU	2/12/2023 17:00	11	12.0	12.6	0.6
ICU	2/12/2023 18:00	11	12.0	12.6	0.6
ICU	2/12/2023 19:00	11	12.0	12.6	0.6
ICU	2/12/2023 20:00	11	12.0	11.0	-1.0
ICU	2/12/2023 21:00	11	12.0	11.0	-1.0
ICU	2/12/2023 22:00	11	12.0	11.0	-1.0
ICU	2/12/2023 23:00	11	12.0	11.0	-1.0
ICU	2/13/2023 0:00	11	12.0	11.0	-1.0
ICU	2/13/2023 1:00	11	12.0	11.0	-1.0
ICU	2/13/2023 2:00	11	12.0	11.0	-1.0
ICU	2/13/2023 3:00	11	12.0	11.0	-1.0
ICU	2/13/2023 4:00	11	12.0	11.0	-1.0
ICU	2/13/2023 5:00	11	12.0	11.0	-1.0
ICU	2/13/2023 6:00	11	12.0	11.0	-1.0
ICU	2/13/2023 7:00	11	12.0	11.0	-1.0
ICU	2/13/2023 8:00	11	12.0	11.0	-1.0
ICU	2/13/2023 9:00	11	12.0	11.0	-1.0
ICU	2/13/2023 10:00	11	12.0	11.0	-1.0
ICU	2/13/2023 11:00	11	12.0	11.0	-1.0
ICU	2/13/2023 12:00	11	12.0	11.0	-1.0
ICU	2/13/2023 13:00	11	12.0	11.0	-1.0
ICU	2/13/2023 14:00	11	12.0	11.0	-1.0
ICU	2/13/2023 15:00	11	12.0	10.4	-1.6
ICU	2/13/2023 16:00	11	12.0	11.0	-1.0
ICU	2/13/2023 17:00	11	12.0	11.0	-1.0
ICU	2/13/2023 18:00	11	12.0	11.0	-1.0
ICU	2/13/2023 19:00	11	12.0	11.0	-1.0
ICU	2/13/2023 20:00	11	12.0	11.0	-1.0
ICU	2/13/2023 21:00	11	12.0	11.0	-1.0
ICU	2/13/2023 22:00	11	12.0	11.0	-1.0
ICU	2/13/2023 23:00	11	12.0	11.0	-1.0
ICU	2/14/2023 0:00	11	12.0	11.0	-1.0
ICU	2/14/2023 1:00	11	12.0	11.0	-1.0
ICU	2/14/2023 2:00	11	12.0	11.0	-1.0
ICU	2/14/2023 3:00	11	12.0	11.0	-1.0
ICU	2/14/2023 4:00	11	12.0	11.0	-1.0
ICU	2/14/2023 5:00	11	12.0	11.0	-1.0
ICU	2/14/2023 6:00	11	12.0	10.4	-1.6
ICU	2/14/2023 7:00	11	12.0	10.4	-1.6
ICU	2/14/2023 8:00	11	12.0	12.6	0.6
ICU	2/14/2023 9:00	10	12.0	12.6	0.6
ICU	2/14/2023 10:00	11	12.0	12.6	0.6
ICU	2/14/2023 11:00	11	12.0	12.6	0.6

ICU	2/14/2023 12:00	8	12.0	12.6	0.6
ICU	2/14/2023 13:00	8	12.0	12.6	0.6
ICU	2/14/2023 14:00	8	12.0	12.6	0.6
ICU	2/14/2023 15:00	8	12.0	12.6	0.6
ICU	2/14/2023 16:00	8	12.0	12.6	0.6
ICU	2/14/2023 17:00	8	12.0	12.6	0.6
ICU	2/14/2023 18:00	8	12.0	12.6	0.6
ICU	2/14/2023 19:00	8	12.0	12.6	0.6
ICU	2/14/2023 20:00	8	12.0	9.5	-2.5
ICU	2/14/2023 21:00	8	12.0	9.5	-2.5
ICU	2/14/2023 22:00	9	10.0	9.5	-0.5
ICU	2/14/2023 23:00	9	10.0	9.5	-0.5
ICU	2/15/2023 0:00	9	10.0	8.9	-1.1
ICU	2/15/2023 1:00	9	10.0	8.9	-1.1
ICU	2/15/2023 2:00	9	10.0	8.9	-1.1
ICU	2/15/2023 3:00	9	10.0	8.9	-1.1
ICU	2/15/2023 4:00	9	10.0	8.9	-1.1
ICU	2/15/2023 5:00	9	10.0	8.9	-1.1
ICU	2/15/2023 6:00	9	10.0	8.9	-1.1
ICU	2/15/2023 7:00	9	10.0	8.9	-1.1
ICU	2/15/2023 8:00	9	12.0	9.5	-2.5
ICU	2/15/2023 9:00	9	12.0	9.5	-2.5
ICU	2/15/2023 10:00	9	12.0	9.5	-2.5
ICU	2/15/2023 11:00	9	12.0	9.5	-2.5
ICU	2/15/2023 12:00	9	12.0	11.0	-1.0
ICU	2/15/2023 13:00	9	12.0	11.0	-1.0
ICU	2/15/2023 14:00	9	12.0	11.0	-1.0
ICU	2/15/2023 15:00	9	12.0	11.0	-1.0
ICU	2/15/2023 16:00	9	12.0	7.4	-4.6
ICU	2/15/2023 17:00	9	12.0	7.4	-4.6
ICU	2/15/2023 18:00	9	12.0	7.4	-4.6
ICU	2/15/2023 19:00	9	12.0	7.4	-4.6
ICU	2/15/2023 20:00	7	9.0	7.4	-1.6
ICU	2/15/2023 21:00	7	9.0	7.4	-1.6
ICU	2/15/2023 22:00	5	9.0	7.4	-1.6
ICU	2/15/2023 23:00	5	9.0	7.4	-1.6
ICU	2/16/2023 0:00	5	9.0	7.4	-1.6
ICU	2/16/2023 1:00	5	9.0	7.4	-1.6
ICU	2/16/2023 2:00	5	9.0	5.9	-3.1
ICU	2/16/2023 3:00	5	9.0	5.9	-3.1
ICU	2/16/2023 4:00	5	9.0	5.9	-3.1
ICU	2/16/2023 5:00	5	9.0	5.9	-3.1
ICU	2/16/2023 6:00	5	9.0	5.9	-3.1
ICU	2/16/2023 7:00	5	9.0	5.9	-3.1
ICU	2/16/2023 8:00	5	9.0	10.4	1.4
ICU	2/16/2023 9:00	5	9.0	10.4	1.4
ICU	2/16/2023 10:00	5	9.0	10.4	1.4

ICU	2/16/2023 11:00	5	9.0	10.4	1.4
ICU	2/16/2023 12:00	5	9.0	10.4	1.4
ICU	2/16/2023 13:00	5	9.0	9.3	0.3
ICU	2/16/2023 14:00	5	9.0	8.9	-0.1
ICU	2/16/2023 15:00	5	9.0	8.9	-0.1
ICU	2/16/2023 16:00	5	9.0	10.4	1.4
ICU	2/16/2023 17:00	5	9.0	10.4	1.4
ICU	2/16/2023 18:00	5	9.0	10.4	1.4
ICU	2/16/2023 19:00	5	9.0	10.4	1.4
ICU	2/16/2023 20:00	7	9.0	11.0	2.0
ICU	2/16/2023 21:00	7	9.0	11.0	2.0
ICU	2/16/2023 22:00	7	9.0	11.0	2.0
ICU	2/16/2023 23:00	7	9.0	11.0	2.0
ICU	2/17/2023 0:00	7	9.0	11.0	2.0
ICU	2/17/2023 1:00	8	9.0	11.0	2.0
ICU	2/17/2023 2:00	8	9.0	11.0	2.0
ICU	2/17/2023 3:00	8	9.0	11.0	2.0
ICU	2/17/2023 4:00	8	9.0	11.0	2.0
ICU	2/17/2023 5:00	8	9.0	11.0	2.0
ICU	2/17/2023 6:00	8	9.0	11.0	2.0
ICU	2/17/2023 7:00	8	9.0	11.0	2.0
ICU	2/17/2023 8:00	8	9.0	11.9	2.9
ICU	2/17/2023 9:00	8	9.0	11.9	2.9
ICU	2/17/2023 10:00	8	9.0	11.9	2.9
ICU	2/17/2023 11:00	8	9.0	11.9	2.9
ICU	2/17/2023 12:00	8	9.0	11.9	2.9
ICU	2/17/2023 13:00	8	9.0	11.9	2.9
ICU	2/17/2023 14:00	8	9.0	11.9	2.9
ICU	2/17/2023 15:00	8	9.0	11.9	2.9
ICU	2/17/2023 16:00	8	9.0	12.6	3.6
ICU	2/17/2023 17:00	8	9.0	12.6	3.6
ICU	2/17/2023 18:00	8	9.0	12.6	3.6
ICU	2/17/2023 19:00	8	9.0	12.6	3.6
ICU	2/17/2023 20:00	8	9.0	11.0	2.0
ICU	2/17/2023 21:00	8	9.0	11.0	2.0
ICU	2/17/2023 22:00	8	9.0	11.0	2.0
ICU	2/17/2023 23:00	11	9.0	9.9	0.9
ICU	2/18/2023 0:00	11	9.0	11.0	2.0
ICU	2/18/2023 1:00	11	9.0	11.0	2.0
ICU	2/18/2023 2:00	11	9.0	11.0	2.0
ICU	2/18/2023 3:00	11	9.0	11.0	2.0
ICU	2/18/2023 4:00	11	9.0	11.0	2.0
ICU	2/18/2023 5:00	11	9.0	11.0	2.0
ICU	2/18/2023 6:00	11	9.0	11.0	2.0
ICU	2/18/2023 7:00	11	9.0	11.0	2.0
ICU	2/18/2023 8:00	9	10.0	9.6	-0.4
ICU	2/18/2023 9:00	9	10.0	10.4	0.4

ICU	2/18/2023 10:00	9	10.0	10.4	0.4
ICU	2/18/2023 11:00	9	10.0	10.4	0.4
ICU	2/18/2023 12:00	8	10.0	11.9	1.9
ICU	2/18/2023 13:00	8	10.0	11.9	1.9
ICU	2/18/2023 14:00	7	10.0	11.9	1.9
ICU	2/18/2023 15:00	7	10.0	11.1	1.1
ICU	2/18/2023 16:00	7	10.0	9.5	-0.5
ICU	2/18/2023 17:00	6	10.0	8.9	-1.1
ICU	2/18/2023 18:00	6	10.0	8.9	-1.1
ICU	2/18/2023 19:00	6	10.0	8.9	-1.1
ICU	2/18/2023 20:00	6	11.0	9.5	-1.5
ICU	2/18/2023 21:00	6	11.0	9.5	-1.5
ICU	2/18/2023 22:00	6	11.0	9.5	-1.5
ICU	2/18/2023 23:00	6	11.0	9.5	-1.5
ICU	2/19/2023 0:00	6	11.0	8.9	-2.1
ICU	2/19/2023 1:00	6	11.0	8.9	-2.1
ICU	2/19/2023 2:00	6	11.0	8.9	-2.1
ICU	2/19/2023 3:00	6	11.0	8.9	-2.1
ICU	2/19/2023 4:00	6	11.0	8.9	-2.1
ICU	2/19/2023 5:00	6	11.0	8.9	-2.1
ICU	2/19/2023 6:00	6	11.0	8.9	-2.1
ICU	2/19/2023 7:00	6	11.0	8.9	-2.1
ICU	2/19/2023 8:00	6	11.0	9.5	-1.5
ICU	2/19/2023 9:00	6	11.0	9.5	-1.5
ICU	2/19/2023 10:00	6	11.0	9.5	-1.5
ICU	2/19/2023 11:00	6	11.0	9.5	-1.5
ICU	2/19/2023 12:00	6	11.0	9.5	-1.5
ICU	2/19/2023 13:00	6	11.0	9.5	-1.5
ICU	2/19/2023 14:00	6	11.0	9.5	-1.5
ICU	2/19/2023 15:00	6	11.0	9.5	-1.5
ICU	2/19/2023 16:00	6	11.0	7.9	-3.1
ICU	2/19/2023 17:00	6	11.0	7.9	-3.1
ICU	2/19/2023 18:00	6	11.0	7.9	-3.1
ICU	2/19/2023 19:00	6	11.0	7.9	-3.1
ICU	2/19/2023 20:00	8	11.0	10.4	-0.6
ICU	2/19/2023 21:00	8	11.0	10.4	-0.6
ICU	2/19/2023 22:00	8	11.0	10.4	-0.6
ICU	2/19/2023 23:00	8	11.0	10.4	-0.6
ICU	2/20/2023 0:00	8	11.0	11.0	0.0
ICU	2/20/2023 1:00	8	11.0	11.0	0.0
ICU	2/20/2023 2:00	8	11.0	11.0	0.0
ICU	2/20/2023 3:00	8	11.0	11.0	0.0
ICU	2/20/2023 4:00	8	11.0	11.0	0.0
ICU	2/20/2023 5:00	8	11.0	11.0	0.0
ICU	2/20/2023 6:00	8	11.0	11.0	0.0
ICU	2/20/2023 7:00	8	11.0	11.0	0.0
ICU	2/20/2023 8:00	8	11.0	9.5	-1.5

ICU	2/20/2023 9:00	8	11.0	9.5	-1.5
ICU	2/20/2023 10:00	7	11.0	9.5	-1.5
ICU	2/20/2023 11:00	7	11.0	9.5	-1.5
ICU	2/20/2023 12:00	7	11.0	9.5	-1.5
ICU	2/20/2023 13:00	7	11.0	9.5	-1.5
ICU	2/20/2023 14:00	6	11.0	9.5	-1.5
ICU	2/20/2023 15:00	5	11.0	9.5	-1.5
ICU	2/20/2023 16:00	5	11.0	7.4	-3.6
ICU	2/20/2023 17:00	5	11.0	7.4	-3.6
ICU	2/20/2023 18:00	5	11.0	7.4	-3.6
ICU	2/20/2023 19:00	5	11.0	7.4	-3.6
ICU	2/20/2023 20:00	5	11.0	9.5	-1.5
ICU	2/20/2023 21:00	5	11.0	9.5	-1.5
ICU	2/20/2023 22:00	5	11.0	9.5	-1.5
ICU	2/20/2023 23:00	5	11.0	9.5	-1.5
ICU	2/21/2023 0:00	5	11.0	9.5	-1.5
ICU	2/21/2023 1:00	5	11.0	9.5	-1.5
ICU	2/21/2023 2:00	5	11.0	9.5	-1.5
ICU	2/21/2023 3:00	5	11.0	9.5	-1.5
ICU	2/21/2023 4:00	5	11.0	9.5	-1.5
ICU	2/21/2023 5:00	5	11.0	9.5	-1.5
ICU	2/21/2023 6:00	5	11.0	9.5	-1.5
ICU	2/21/2023 7:00	6	9.0	9.5	0.5
ICU	2/21/2023 8:00	6	9.0	9.5	0.5
ICU	2/21/2023 9:00	6	9.0	9.5	0.5
ICU	2/21/2023 10:00	6	9.0	9.5	0.5
ICU	2/21/2023 11:00	6	9.0	9.5	0.5
ICU	2/21/2023 12:00	6	9.0	9.5	0.5
ICU	2/21/2023 13:00	6	9.0	9.5	0.5
ICU	2/21/2023 14:00	6	9.0	9.5	0.5
ICU	2/21/2023 15:00	8	9.0	9.5	0.5
ICU	2/21/2023 16:00	8	9.0	7.9	-1.1
ICU	2/21/2023 17:00	6	9.0	9.5	0.5
ICU	2/21/2023 18:00	6	9.0	9.5	0.5
ICU	2/21/2023 19:00	6	9.0	9.5	0.5
ICU	2/21/2023 20:00	7	8.0	9.5	1.5
ICU	2/21/2023 21:00	7	8.0	9.5	1.5
ICU	2/21/2023 22:00	7	8.0	9.5	1.5
ICU	2/21/2023 23:00	7	8.0	9.5	1.5
ICU	2/22/2023 0:00	7	8.0	9.5	1.5
ICU	2/22/2023 1:00	7	8.0	9.5	1.5
ICU	2/22/2023 2:00	7	8.0	9.5	1.5
ICU	2/22/2023 3:00	8	10.0	8.4	-1.7
ICU	2/22/2023 4:00	8	10.0	9.5	-0.5
ICU	2/22/2023 5:00	8	10.0	9.5	-0.5
ICU	2/22/2023 6:00	8	10.0	9.5	-0.5
ICU	2/22/2023 7:00	8	10.0	9.5	-0.5

ICU	2/22/2023 8:00	8	10.0	11.0	1.0
ICU	2/22/2023 9:00	8	10.0	11.0	1.0
ICU	2/22/2023 10:00	8	10.0	11.0	1.0
ICU	2/22/2023 11:00	8	10.0	11.0	1.0
ICU	2/22/2023 12:00	8	10.0	11.0	1.0
ICU	2/22/2023 13:00	8	10.0	11.0	1.0
ICU	2/22/2023 14:00	8	10.0	11.0	1.0
ICU	2/22/2023 15:00	8	10.0	11.0	1.0
ICU	2/22/2023 16:00	8	10.0	10.4	0.4
ICU	2/22/2023 17:00	8	10.0	10.4	0.4
ICU	2/22/2023 18:00	6	8.0	10.4	2.4
ICU	2/22/2023 19:00	6	8.0	10.4	2.4
ICU	2/22/2023 20:00	6	8.0	6.3	-1.7
ICU	2/22/2023 21:00	6	8.0	6.3	-1.7
ICU	2/22/2023 22:00	5	8.0	6.3	-1.7
ICU	2/22/2023 23:00	5	8.0	6.3	-1.7
ICU	2/23/2023 0:00	5	8.0	7.9	-0.1
ICU	2/23/2023 1:00	5	8.0	7.9	-0.1
ICU	2/23/2023 2:00	5	8.0	7.9	-0.1
ICU	2/23/2023 3:00	5	8.0	7.9	-0.1
ICU	2/23/2023 4:00	5	8.0	7.9	-0.1
ICU	2/23/2023 5:00	5	8.0	7.9	-0.1
ICU	2/23/2023 6:00	5	8.0	7.9	-0.1
ICU	2/23/2023 7:00	5	8.0	7.9	-0.1
ICU	2/23/2023 8:00	5	8.0	9.5	1.5
ICU	2/23/2023 9:00	5	8.0	9.5	1.5
ICU	2/23/2023 10:00	5	8.0	9.5	1.5
ICU	2/23/2023 11:00	4	8.0	9.5	1.5
ICU	2/23/2023 12:00	5	8.0	7.9	-0.1
ICU	2/23/2023 13:00	5	8.0	7.9	-0.1
ICU	2/23/2023 14:00	5	8.0	7.9	-0.1
ICU	2/23/2023 15:00	5	8.0	7.9	-0.1
ICU	2/23/2023 16:00	5	8.0	7.4	-0.6
ICU	2/23/2023 17:00	5	8.0	7.4	-0.6
ICU	2/23/2023 18:00	5	8.0	7.4	-0.6
ICU	2/23/2023 19:00	5	8.0	7.4	-0.6
ICU	2/23/2023 20:00	6	8.0	11.0	3.0
ICU	2/23/2023 21:00	7	8.0	11.0	3.0
ICU	2/23/2023 22:00	7	8.0	11.0	3.0
ICU	2/23/2023 23:00	8	8.0	11.0	3.0
ICU	2/24/2023 0:00	8	10.0	11.0	1.0
ICU	2/24/2023 1:00	8	10.0	11.0	1.0
ICU	2/24/2023 2:00	8	10.0	11.0	1.0
ICU	2/24/2023 3:00	8	10.0	11.0	1.0
ICU	2/24/2023 4:00	8	10.0	11.0	1.0
ICU	2/24/2023 5:00	8	10.0	11.0	1.0
ICU	2/24/2023 6:00	8	10.0	11.0	1.0

ICU	2/24/2023 7:00	8	10.0	11.0	1.0
ICU	2/24/2023 8:00	8	10.0	11.0	1.0
ICU	2/24/2023 9:00	8	10.0	11.0	1.0
ICU	2/24/2023 10:00	8	10.0	11.0	1.0
ICU	2/24/2023 11:00	8	10.0	11.0	1.0
ICU	2/24/2023 12:00	8	10.0	12.6	2.6
ICU	2/24/2023 13:00	8	10.0	12.6	2.6
ICU	2/24/2023 14:00	8	10.0	12.6	2.6
ICU	2/24/2023 15:00	8	10.0	12.6	2.6
ICU	2/24/2023 16:00	8	10.0	12.6	2.6
ICU	2/24/2023 17:00	8	10.0	12.6	2.6
ICU	2/24/2023 18:00	8	10.0	12.6	2.6
ICU	2/24/2023 19:00	8	10.0	12.6	2.6
ICU	2/24/2023 20:00	8	10.0	11.0	1.0
ICU	2/24/2023 21:00	8	10.0	11.0	1.0
ICU	2/24/2023 22:00	7	10.0	11.0	1.0
ICU	2/24/2023 23:00	7	10.0	11.0	1.0
ICU	2/25/2023 0:00	7	10.0	10.4	0.4
ICU	2/25/2023 1:00	7	10.0	10.4	0.4
ICU	2/25/2023 2:00	7	10.0	10.4	0.4
ICU	2/25/2023 3:00	7	10.0	10.4	0.4
ICU	2/25/2023 4:00	7	10.0	10.4	0.4
ICU	2/25/2023 5:00	7	10.0	10.4	0.4
ICU	2/25/2023 6:00	7	10.0	10.4	0.4
ICU	2/25/2023 7:00	7	10.0	10.4	0.4
ICU	2/25/2023 8:00	7	10.0	8.9	-1.1
ICU	2/25/2023 9:00	6	10.0	8.9	-1.1
ICU	2/25/2023 10:00	6	10.0	8.9	-1.1
ICU	2/25/2023 11:00	6	10.0	8.9	-1.1
ICU	2/25/2023 12:00	6	10.0	8.9	-1.1
ICU	2/25/2023 13:00	6	10.0	8.9	-1.1
ICU	2/25/2023 14:00	6	10.0	8.9	-1.1
ICU	2/25/2023 15:00	6	10.0	8.9	-1.1
ICU	2/25/2023 16:00	5	10.0	9.5	-0.5
ICU	2/25/2023 17:00	5	10.0	9.5	-0.5
ICU	2/25/2023 18:00	5	10.0	9.5	-0.5
ICU	2/25/2023 19:00	5	10.0	9.5	-0.5
ICU	2/25/2023 20:00	4	10.0	9.5	-0.5
ICU	2/25/2023 21:00	4	10.0	9.5	-0.5
ICU	2/25/2023 22:00	4	10.0	9.5	-0.5
ICU	2/25/2023 23:00	4	10.0	9.5	-0.5
ICU	2/26/2023 0:00	4	10.0	8.9	-1.1
ICU	2/26/2023 1:00	4	10.0	8.9	-1.1
ICU	2/26/2023 2:00	4	10.0	8.9	-1.1
ICU	2/26/2023 3:00	5	10.0	8.9	-1.1
ICU	2/26/2023 4:00	5	10.0	8.9	-1.1
ICU	2/26/2023 5:00	6	10.0	8.9	-1.1

ICU	2/26/2023 6:00	6	10.0	8.9	-1.1
ICU	2/26/2023 7:00	6	10.0	8.9	-1.1
ICU	2/26/2023 8:00	6	10.0	12.6	2.6
ICU	2/26/2023 9:00	6	10.0	12.6	2.6
ICU	2/26/2023 10:00	6	10.0	12.6	2.6
ICU	2/26/2023 11:00	6	10.0	12.6	2.6
ICU	2/26/2023 12:00	6	10.0	12.6	2.6
ICU	2/26/2023 13:00	6	10.0	12.6	2.6
ICU	2/26/2023 14:00	6	10.0	12.6	2.6
ICU	2/26/2023 15:00	6	10.0	12.6	2.6
ICU	2/26/2023 16:00	6	10.0	11.0	1.0
ICU	2/26/2023 17:00	6	10.0	11.0	1.0
ICU	2/26/2023 18:00	6	10.0	11.0	1.0
ICU	2/26/2023 19:00	6	10.0	11.0	1.0
ICU	2/26/2023 20:00	6	10.0	11.0	1.0
ICU	2/26/2023 21:00	6	10.0	11.0	1.0
ICU	2/26/2023 22:00	6	10.0	11.0	1.0
ICU	2/26/2023 23:00	6	10.0	11.0	1.0
ICU	2/27/2023 0:00	6	10.0	11.0	1.0
ICU	2/27/2023 1:00	6	10.0	11.0	1.0
ICU	2/27/2023 2:00	6	10.0	11.0	1.0
ICU	2/27/2023 3:00	6	10.0	11.0	1.0
ICU	2/27/2023 4:00	6	10.0	11.0	1.0
ICU	2/27/2023 5:00	6	10.0	11.0	1.0
ICU	2/27/2023 6:00	6	10.0	11.0	1.0
ICU	2/27/2023 7:00	6	10.0	11.0	1.0
ICU	2/27/2023 8:00	6	10.0	14.2	4.2
ICU	2/27/2023 9:00	6	10.0	14.2	4.2
ICU	2/27/2023 10:00	6	10.0	14.2	4.2
ICU	2/27/2023 11:00	6	10.0	14.2	4.2
ICU	2/27/2023 12:00	6	10.0	14.2	4.2
ICU	2/27/2023 13:00	6	10.0	14.2	4.2
ICU	2/27/2023 14:00	6	10.0	14.2	4.2
ICU	2/27/2023 15:00	6	10.0	14.2	4.2
ICU	2/27/2023 16:00	6	10.0	12.6	2.6
ICU	2/27/2023 17:00	6	10.0	12.6	2.6
ICU	2/27/2023 18:00	6	10.0	12.6	2.6
ICU	2/27/2023 19:00	9	12.0	12.6	0.6
ICU	2/27/2023 20:00	9	12.0	11.0	-1.0
ICU	2/27/2023 21:00	9	12.0	11.0	-1.0
ICU	2/27/2023 22:00	9	12.0	11.0	-1.0
ICU	2/27/2023 23:00	9	12.0	11.0	-1.0
ICU	2/28/2023 0:00	9	12.0	11.0	-1.0
ICU	2/28/2023 1:00	9	12.0	11.0	-1.0
ICU	2/28/2023 2:00	9	12.0	11.0	-1.0
ICU	2/28/2023 3:00	9	12.0	11.0	-1.0
ICU	2/28/2023 4:00	9	12.0	11.0	-1.0

ICU	2/28/2023 5:00	9	12.0	11.0	-1.0
ICU	2/28/2023 6:00	9	12.0	11.0	-1.0
ICU	2/28/2023 7:00	9	12.0	11.0	-1.0
ICU	2/28/2023 8:00	10	11.0	12.6	1.6
ICU	2/28/2023 9:00	9	11.0	11.5	0.5
ICU	2/28/2023 10:00	10	11.0	11.5	0.5
ICU	2/28/2023 11:00	10	11.0	12.6	1.6
ICU	2/28/2023 12:00	10	11.0	12.6	1.6
ICU	2/28/2023 13:00	10	11.0	11.5	0.5
ICU	2/28/2023 14:00	10	11.0	11.5	0.5
ICU	2/28/2023 15:00	10	11.0	12.6	1.6
ICU	2/28/2023 16:00	10	11.0	11.9	0.9
ICU	2/28/2023 17:00	10	11.0	11.9	0.9
ICU	2/28/2023 18:00	10	11.0	9.6	-1.4
ICU	2/28/2023 19:00	10	11.0	11.9	0.9
ICU	2/28/2023 20:00	11	11.0	7.4	-3.6
ICU	2/28/2023 21:00	11	11.0	7.4	-3.6
ICU	2/28/2023 22:00	11	11.0	7.4	-3.6
ICU	2/28/2023 23:00	11	11.0	5.9	-5.1
ICU	3/1/2023 0:00	11	11.0	5.9	-5.1
ICU	3/1/2023 1:00	11	11.0	5.9	-5.1
ICU	3/1/2023 2:00	11	11.0	5.9	-5.1
ICU	3/1/2023 3:00	11	11.0	5.9	-5.1
ICU	3/1/2023 4:00	11	11.0	5.9	-5.1
ICU	3/1/2023 5:00	11	11.0	5.9	-5.1
ICU	3/1/2023 6:00	11	11.0	5.9	-5.1
ICU	3/1/2023 7:00	11	11.0	5.9	-5.1
ICU	3/1/2023 8:00	11	11.0	8.9	-2.1
ICU	3/1/2023 9:00	11	11.0	8.9	-2.1
ICU	3/1/2023 10:00	11	11.0	8.9	-2.1
ICU	3/1/2023 11:00	10	11.0	8.9	-2.1
ICU	3/1/2023 12:00	10	11.0	11.9	0.9
ICU	3/1/2023 13:00	10	11.0	11.9	0.9
ICU	3/1/2023 14:00	10	11.0	11.9	0.9
ICU	3/1/2023 15:00	9	11.0	11.9	0.9
ICU	3/1/2023 16:00	9	11.0	10.4	-0.6
ICU	3/1/2023 17:00	9	11.0	10.4	-0.6
ICU	3/1/2023 18:00	9	11.0	10.4	-0.6
ICU	3/1/2023 19:00	9	11.0	10.4	-0.6
ICU	3/1/2023 20:00	9	11.0	8.9	-2.1
ICU	3/1/2023 21:00	9	11.0	8.9	-2.1
ICU	3/1/2023 22:00	10	11.0	9.5	-1.5
ICU	3/1/2023 23:00	10	11.0	9.5	-1.5
ICU	3/2/2023 0:00	10	11.0	9.5	-1.5
ICU	3/2/2023 1:00	10	11.0	9.5	-1.5
ICU	3/2/2023 2:00	10	11.0	9.5	-1.5
ICU	3/2/2023 3:00	10	11.0	9.5	-1.5

ICU	3/2/2023 4:00	10	11.0	9.5	-1.5
ICU	3/2/2023 5:00	10	11.0	9.5	-1.5
ICU	3/2/2023 6:00	10	11.0	9.5	-1.5
ICU	3/2/2023 7:00	10	11.0	9.5	-1.5
ICU	3/2/2023 8:00	10	11.0	12.6	1.6
ICU	3/2/2023 9:00	10	11.0	12.6	1.6
ICU	3/2/2023 10:00	10	11.0	12.6	1.6
ICU	3/2/2023 11:00	10	11.0	12.6	1.6
ICU	3/2/2023 12:00	10	11.0	14.2	3.2
ICU	3/2/2023 13:00	10	11.0	14.2	3.2
ICU	3/2/2023 14:00	10	11.0	14.2	3.2
ICU	3/2/2023 15:00	10	11.0	14.2	3.2
ICU	3/2/2023 16:00	10	11.0	10.4	-0.6
ICU	3/2/2023 17:00	10	11.0	10.4	-0.6
ICU	3/2/2023 18:00	10	11.0	10.4	-0.6
ICU	3/2/2023 19:00	10	11.0	10.4	-0.6
ICU	3/2/2023 20:00	10	11.0	9.5	-1.5
ICU	3/2/2023 21:00	10	11.0	9.5	-1.5
ICU	3/2/2023 22:00	10	11.0	8.4	-2.7
ICU	3/2/2023 23:00	10	11.0	9.5	-1.5
ICU	3/3/2023 0:00	10	11.0	7.8	-3.2
ICU	3/3/2023 1:00	10	11.0	8.9	-2.1
ICU	3/3/2023 2:00	10	11.0	8.9	-2.1
ICU	3/3/2023 3:00	10	11.0	8.9	-2.1
ICU	3/3/2023 4:00	10	11.0	8.9	-2.1
ICU	3/3/2023 5:00	10	11.0	8.9	-2.1
ICU	3/3/2023 6:00	10	11.0	8.9	-2.1
ICU	3/3/2023 7:00	10	11.0	8.9	-2.1
ICU	3/3/2023 8:00	10	11.0	11.0	0.0
ICU	3/3/2023 9:00	10	11.0	11.0	0.0
ICU	3/3/2023 10:00	10	11.0	11.0	0.0
ICU	3/3/2023 11:00	10	11.0	11.0	0.0
ICU	3/3/2023 12:00	10	11.0	12.6	1.6
ICU	3/3/2023 13:00	10	11.0	12.6	1.6
ICU	3/3/2023 14:00	10	11.0	12.6	1.6
ICU	3/3/2023 15:00	10	11.0	12.6	1.6
ICU	3/3/2023 16:00	10	11.0	12.6	1.6
ICU	3/3/2023 17:00	10	11.0	12.6	1.6
ICU	3/3/2023 18:00	10	11.0	12.6	1.6
ICU	3/3/2023 19:00	10	11.0	12.6	1.6
ICU	3/3/2023 20:00	10	11.0	12.6	1.6
ICU	3/3/2023 21:00	10	11.0	12.6	1.6
ICU	3/3/2023 22:00	10	11.0	12.6	1.6
ICU	3/3/2023 23:00	10	11.0	12.6	1.6
ICU	3/4/2023 0:00	10	11.0	12.6	1.6
ICU	3/4/2023 1:00	10	11.0	12.6	1.6
ICU	3/4/2023 2:00	10	11.0	12.6	1.6

ICU	3/4/2023 3:00	10	11.0	12.6	1.6
ICU	3/4/2023 4:00	10	11.0	12.6	1.6
ICU	3/4/2023 5:00	10	11.0	12.6	1.6
ICU	3/4/2023 6:00	10	11.0	12.6	1.6
ICU	3/4/2023 7:00	10	11.0	12.6	1.6
ICU	3/4/2023 8:00	11	12.0	12.6	0.6
ICU	3/4/2023 9:00	11	12.0	12.6	0.6
ICU	3/4/2023 10:00	11	12.0	12.6	0.6
ICU	3/4/2023 11:00	11	12.0	12.6	0.6
ICU	3/4/2023 12:00	9	12.0	12.6	0.6
ICU	3/4/2023 13:00	9	12.0	12.6	0.6
ICU	3/4/2023 14:00	9	12.0	12.6	0.6
ICU	3/4/2023 15:00	9	12.0	12.6	0.6
ICU	3/4/2023 16:00	9	12.0	11.0	-1.0
ICU	3/4/2023 17:00	9	12.0	11.0	-1.0
ICU	3/4/2023 18:00	9	12.0	11.0	-1.0
ICU	3/4/2023 19:00	9	12.0	11.0	-1.0
ICU	3/4/2023 20:00	11	11.0	11.0	0.0
ICU	3/4/2023 21:00	11	11.0	11.0	0.0
ICU	3/4/2023 22:00	11	11.0	11.0	0.0
ICU	3/4/2023 23:00	11	11.0	11.0	0.0
ICU	3/5/2023 0:00	11	11.0	11.0	0.0
ICU	3/5/2023 1:00	11	11.0	11.0	0.0
ICU	3/5/2023 2:00	11	11.0	11.0	0.0
ICU	3/5/2023 3:00	11	11.0	11.0	0.0
ICU	3/5/2023 4:00	11	11.0	11.0	0.0
ICU	3/5/2023 5:00	11	11.0	11.0	0.0
ICU	3/5/2023 6:00	11	11.0	11.0	0.0
ICU	3/5/2023 7:00	11	11.0	11.0	0.0
ICU	3/5/2023 8:00	11	11.0	13.3	2.3
ICU	3/5/2023 9:00	11	11.0	13.3	2.3
ICU	3/5/2023 10:00	11	11.0	13.3	2.3
ICU	3/5/2023 11:00	11	11.0	13.3	2.3
ICU	3/5/2023 12:00	10	11.0	13.3	2.3
ICU	3/5/2023 13:00	10	11.0	13.3	2.3
ICU	3/5/2023 14:00	10	11.0	13.3	2.3
ICU	3/5/2023 15:00	11	11.0	13.3	2.3
ICU	3/5/2023 16:00	10	10.0	11.0	1.0
ICU	3/5/2023 17:00	10	10.0	11.0	1.0
ICU	3/5/2023 18:00	10	10.0	11.0	1.0
ICU	3/5/2023 19:00	10	10.0	11.0	1.0
ICU	3/5/2023 20:00	10	10.0	11.0	1.0
ICU	3/5/2023 21:00	10	10.0	9.5	-0.5
ICU	3/5/2023 22:00	10	10.0	9.5	-0.5
ICU	3/5/2023 23:00	10	10.0	7.9	-2.1
ICU	3/6/2023 0:00	10	10.0	7.9	-2.1
ICU	3/6/2023 1:00	10	10.0	7.9	-2.1

ICU	3/6/2023 2:00	10	10.0	7.9	-2.1
ICU	3/6/2023 3:00	10	10.0	7.9	-2.1
ICU	3/6/2023 4:00	10	10.0	7.9	-2.1
ICU	3/6/2023 5:00	10	10.0	7.9	-2.1
ICU	3/6/2023 6:00	10	10.0	7.9	-2.1
ICU	3/6/2023 7:00	10	10.0	7.9	-2.1
ICU	3/6/2023 8:00	10	10.0	9.5	-0.5
ICU	3/6/2023 9:00	10	10.0	9.5	-0.5
ICU	3/6/2023 10:00	10	10.0	9.5	-0.5
ICU	3/6/2023 11:00	9	10.0	9.5	-0.5
ICU	3/6/2023 12:00	9	10.0	9.5	-0.5
ICU	3/6/2023 13:00	9	10.0	9.5	-0.5
ICU	3/6/2023 14:00	9	10.0	9.5	-0.5
ICU	3/6/2023 15:00	9	10.0	9.5	-0.5
ICU	3/6/2023 16:00	9	10.0	9.5	-0.5
ICU	3/6/2023 17:00	9	10.0	9.5	-0.5
ICU	3/6/2023 18:00	9	10.0	9.5	-0.5
ICU	3/6/2023 19:00	9	10.0	9.5	-0.5
ICU	3/6/2023 20:00	10	10.0	8.4	-1.7
ICU	3/6/2023 21:00	10	10.0	9.5	-0.5
ICU	3/6/2023 22:00	10	10.0	9.5	-0.5
ICU	3/6/2023 23:00	10	10.0	9.5	-0.5
ICU	3/7/2023 0:00	10	10.0	9.5	-0.5
ICU	3/7/2023 1:00	10	10.0	9.5	-0.5
ICU	3/7/2023 2:00	10	10.0	9.5	-0.5
ICU	3/7/2023 3:00	10	10.0	9.5	-0.5
ICU	3/7/2023 4:00	10	10.0	9.5	-0.5
ICU	3/7/2023 5:00	10	10.0	9.5	-0.5
ICU	3/7/2023 6:00	10	10.0	9.5	-0.5
ICU	3/7/2023 7:00	10	10.0	9.5	-0.5
ICU	3/7/2023 8:00	10	10.0	9.5	-0.5
ICU	3/7/2023 9:00	10	10.0	9.5	-0.5
ICU	3/7/2023 10:00	10	10.0	9.5	-0.5
ICU	3/7/2023 11:00	10	10.0	9.5	-0.5
ICU	3/7/2023 12:00	10	10.0	9.5	-0.5
ICU	3/7/2023 13:00	10	10.0	9.5	-0.5
ICU	3/7/2023 14:00	10	10.0	9.5	-0.5
ICU	3/7/2023 15:00	10	10.0	9.5	-0.5
ICU	3/7/2023 16:00	10	10.0	8.9	-1.1
ICU	3/7/2023 17:00	10	10.0	8.9	-1.1
ICU	3/7/2023 18:00	10	10.0	8.9	-1.1
ICU	3/7/2023 19:00	10	10.0	8.9	-1.1
ICU	3/7/2023 20:00	10	10.0	8.4	-1.7
ICU	3/7/2023 21:00	10	10.0	9.5	-0.5
ICU	3/7/2023 22:00	10	10.0	9.5	-0.5
ICU	3/7/2023 23:00	10	10.0	9.5	-0.5
ICU	3/8/2023 0:00	10	10.0	9.5	-0.5

ICU	3/8/2023 1:00	10	10.0	9.5	-0.5
ICU	3/8/2023 2:00	10	10.0	9.5	-0.5
ICU	3/8/2023 3:00	10	10.0	9.5	-0.5
ICU	3/8/2023 4:00	10	10.0	9.5	-0.5
ICU	3/8/2023 5:00	11	10.0	8.4	-1.7
ICU	3/8/2023 6:00	11	10.0	9.5	-0.5
ICU	3/8/2023 7:00	11	10.0	9.5	-0.5
ICU	3/8/2023 8:00	11	12.0	7.9	-4.1
ICU	3/8/2023 9:00	11	12.0	7.9	-4.1
ICU	3/8/2023 10:00	11	12.0	7.9	-4.1
ICU	3/8/2023 11:00	10	12.0	8.4	-3.7
ICU	3/8/2023 12:00	10	12.0	12.6	0.6
ICU	3/8/2023 13:00	10	12.0	12.6	0.6
ICU	3/8/2023 14:00	10	12.0	12.6	0.6
ICU	3/8/2023 15:00	9	12.0	11.5	-0.5
ICU	3/8/2023 16:00	8	11.0	9.9	-1.1
ICU	3/8/2023 17:00	8	11.0	11.0	0.0
ICU	3/8/2023 18:00	8	11.0	11.0	0.0
ICU	3/8/2023 19:00	8	11.0	11.0	0.0
ICU	3/8/2023 20:00	8	11.0	6.3	-4.7
ICU	3/8/2023 21:00	10	9.0	6.3	-2.7
ICU	3/8/2023 22:00	10	9.0	6.3	-2.7
ICU	3/8/2023 23:00	10	9.0	6.3	-2.7
ICU	3/9/2023 0:00	10	9.0	6.3	-2.7
ICU	3/9/2023 1:00	10	9.0	6.3	-2.7
ICU	3/9/2023 2:00	10	9.0	6.3	-2.7
ICU	3/9/2023 3:00	10	9.0	6.3	-2.7
ICU	3/9/2023 4:00	10	9.0	6.3	-2.7
ICU	3/9/2023 5:00	8	10.0	7.9	-2.1
ICU	3/9/2023 6:00	8	10.0	7.9	-2.1
ICU	3/9/2023 7:00	8	10.0	7.9	-2.1
ICU	3/9/2023 8:00	8	14.0	14.2	0.2
ICU	3/9/2023 9:00	8	14.0	14.2	0.2
ICU	3/9/2023 10:00	9	14.0	13.1	-0.9
ICU	3/9/2023 11:00	10	14.0	13.1	-0.9
ICU	3/9/2023 12:00	10	14.0	14.2	0.2
ICU	3/9/2023 13:00	10	14.0	14.2	0.2
ICU	3/9/2023 14:00	10	14.0	14.2	0.2
ICU	3/9/2023 15:00	11	14.0	14.2	0.2
ICU	3/9/2023 16:00	11	13.0	13.3	0.3
ICU	3/9/2023 17:00	10	13.0	12.2	-0.8
ICU	3/9/2023 18:00	10	13.0	13.3	0.3
ICU	3/9/2023 19:00	10	13.0	13.3	0.3
ICU	3/9/2023 20:00	10	12.0	12.6	0.6
ICU	3/9/2023 21:00	10	12.0	12.6	0.6
ICU	3/9/2023 22:00	9	12.0	12.6	0.6
ICU	3/9/2023 23:00	9	12.0	12.6	0.6

ICU	3/10/2023 0:00	9	12.0	12.6	0.6
ICU	3/10/2023 1:00	9	12.0	12.6	0.6
ICU	3/10/2023 2:00	9	12.0	12.6	0.6
ICU	3/10/2023 3:00	10	12.0	12.6	0.6
ICU	3/10/2023 4:00	10	12.0	12.6	0.6
ICU	3/10/2023 5:00	10	12.0	12.6	0.6
ICU	3/10/2023 6:00	10	12.0	12.6	0.6
ICU	3/10/2023 7:00	10	12.0	12.6	0.6
ICU	3/10/2023 8:00	10	11.0	11.9	0.9
ICU	3/10/2023 9:00	10	11.0	11.9	0.9
ICU	3/10/2023 10:00	11	11.0	11.9	0.9
ICU	3/10/2023 11:00	9	11.0	11.9	0.9
ICU	3/10/2023 12:00	9	11.0	11.0	0.0
ICU	3/10/2023 13:00	7	11.0	11.9	0.9
ICU	3/10/2023 14:00	7	11.0	10.4	-0.6
ICU	3/10/2023 15:00	7	11.0	11.9	0.9
ICU	3/10/2023 16:00	9	10.0	10.4	0.4
ICU	3/10/2023 17:00	9	10.0	10.4	0.4
ICU	3/10/2023 18:00	9	10.0	10.4	0.4
ICU	3/10/2023 19:00	8	10.0	10.4	0.4
ICU	3/10/2023 20:00	8	10.0	11.0	1.0
ICU	3/10/2023 21:00	8	10.0	11.0	1.0
ICU	3/10/2023 22:00	8	10.0	11.0	1.0
ICU	3/10/2023 23:00	8	10.0	11.0	1.0
ICU	3/11/2023 0:00	8	10.0	11.0	1.0
ICU	3/11/2023 1:00	8	10.0	11.0	1.0
ICU	3/11/2023 2:00	8	10.0	8.8	-1.2
ICU	3/11/2023 3:00	8	10.0	11.0	1.0
ICU	3/11/2023 4:00	8	10.0	11.0	1.0
ICU	3/11/2023 5:00	8	10.0	11.0	1.0
ICU	3/11/2023 6:00	8	10.0	11.0	1.0
ICU	3/11/2023 7:00	8	10.0	11.0	1.0
ICU	3/11/2023 8:00	8	10.0	11.9	1.9
ICU	3/11/2023 9:00	8	10.0	11.9	1.9
ICU	3/11/2023 10:00	9	9.0	8.9	-0.1
ICU	3/11/2023 11:00	9	9.0	8.9	-0.1
ICU	3/11/2023 12:00	9	9.0	8.9	-0.1
ICU	3/11/2023 13:00	9	9.0	8.9	-0.1
ICU	3/11/2023 14:00	9	9.0	8.9	-0.1
ICU	3/11/2023 15:00	9	9.0	8.9	-0.1
ICU	3/11/2023 16:00	8	9.0	11.9	2.9
ICU	3/11/2023 17:00	8	9.0	11.9	2.9
ICU	3/11/2023 18:00	9	9.0	11.9	2.9
ICU	3/11/2023 19:00	9	9.0	12.6	3.6
ICU	3/11/2023 20:00	8	9.0	8.9	-0.1
ICU	3/11/2023 21:00	8	9.0	8.9	-0.1
ICU	3/11/2023 22:00	8	9.0	8.9	-0.1

ICU	3/11/2023 23:00	8	9.0	8.9	-0.1
ICU	3/12/2023 0:00	8	9.0	7.4	-1.6
ICU	3/12/2023 1:00	8	9.0	7.4	-1.6
ICU	3/12/2023 2:00	8	9.0	7.4	-1.6
ICU	3/12/2023 3:00	8	9.0	7.4	-1.6
ICU	3/12/2023 4:00	8	9.0	7.4	-1.6
ICU	3/12/2023 5:00	8	9.0	7.4	-1.6
ICU	3/12/2023 6:00	8	9.0	7.4	-1.6
ICU	3/12/2023 7:00	8	9.0	7.4	-1.6
ICU	3/12/2023 8:00	8	9.0	11.9	2.9
ICU	3/12/2023 9:00	8	9.0	11.9	2.9
ICU	3/12/2023 10:00	8	9.0	11.9	2.9
ICU	3/12/2023 11:00	8	9.0	11.9	2.9
ICU	3/12/2023 12:00	8	9.0	11.9	2.9
ICU	3/12/2023 13:00	8	9.0	11.9	2.9
ICU	3/12/2023 14:00	8	9.0	11.9	2.9
ICU	3/12/2023 15:00	8	9.0	11.9	2.9
ICU	3/12/2023 16:00	8	9.0	10.4	1.4
ICU	3/12/2023 17:00	8	9.0	10.4	1.4
ICU	3/12/2023 18:00	8	9.0	10.4	1.4
ICU	3/12/2023 19:00	8	9.0	9.5	0.5
ICU	3/12/2023 20:00	8	11.0	8.9	-2.1
ICU	3/12/2023 21:00	8	11.0	10.4	-0.6
ICU	3/12/2023 22:00	8	11.0	10.4	-0.6
ICU	3/12/2023 23:00	8	11.0	10.4	-0.6
ICU	3/13/2023 0:00	7	9.0	8.9	-0.1
ICU	3/13/2023 1:00	7	9.0	8.9	-0.1
ICU	3/13/2023 2:00	7	9.0	8.9	-0.1
ICU	3/13/2023 3:00	7	9.0	8.9	-0.1
ICU	3/13/2023 4:00	7	9.0	8.9	-0.1
ICU	3/13/2023 5:00	7	9.0	8.9	-0.1
ICU	3/13/2023 6:00	7	9.0	8.9	-0.1
ICU	3/13/2023 7:00	7	9.0	8.9	-0.1
ICU	3/13/2023 8:00	7	9.0	13.3	4.3
ICU	3/13/2023 9:00	7	9.0	13.3	4.3
ICU	3/13/2023 10:00	7	9.0	13.3	4.3
ICU	3/13/2023 11:00	7	9.0	13.3	4.3
ICU	3/13/2023 12:00	7	9.0	13.3	4.3
ICU	3/13/2023 13:00	7	9.0	13.3	4.3
ICU	3/13/2023 14:00	7	9.0	13.3	4.3
ICU	3/13/2023 15:00	7	9.0	13.3	4.3
ICU	3/13/2023 16:00	7	9.0	11.9	2.9
ICU	3/13/2023 17:00	7	9.0	11.9	2.9
ICU	3/13/2023 18:00	7	9.0	11.9	2.9
ICU	3/13/2023 19:00	7	9.0	11.9	2.9
ICU	3/13/2023 20:00	8	10.0	11.0	1.0
ICU	3/13/2023 21:00	8	10.0	11.0	1.0

ICU	3/13/2023 22:00	9	10.0	11.0	1.0
ICU	3/13/2023 23:00	9	10.0	11.0	1.0
ICU	3/14/2023 0:00	9	10.0	11.0	1.0
ICU	3/14/2023 1:00	9	10.0	11.0	1.0
ICU	3/14/2023 2:00	9	10.0	11.0	1.0
ICU	3/14/2023 3:00	9	10.0	11.0	1.0
ICU	3/14/2023 4:00	9	10.0	11.0	1.0
ICU	3/14/2023 5:00	9	10.0	11.0	1.0
ICU	3/14/2023 6:00	9	10.0	11.0	1.0
ICU	3/14/2023 7:00	9	10.0	11.0	1.0
ICU	3/14/2023 8:00	9	10.0	12.6	2.6
ICU	3/14/2023 9:00	9	10.0	12.6	2.6
ICU	3/14/2023 10:00	9	10.0	12.6	2.6
ICU	3/14/2023 11:00	9	10.0	12.6	2.6
ICU	3/14/2023 12:00	9	10.0	12.6	2.6
ICU	3/14/2023 13:00	9	10.0	12.6	2.6
ICU	3/14/2023 14:00	9	10.0	12.6	2.6
ICU	3/14/2023 15:00	9	10.0	12.6	2.6
ICU	3/14/2023 16:00	9	10.0	12.6	2.6
ICU	3/14/2023 17:00	9	10.0	12.6	2.6
ICU	3/14/2023 18:00	9	10.0	12.6	2.6
ICU	3/14/2023 19:00	9	10.0	12.6	2.6
ICU	3/14/2023 20:00	9	9.0	9.9	0.9
ICU	3/14/2023 21:00	9	9.0	9.9	0.9
ICU	3/14/2023 22:00	9	9.0	11.0	2.0
ICU	3/14/2023 23:00	9	9.0	11.0	2.0
ICU	3/15/2023 0:00	9	8.0	10.4	2.4
ICU	3/15/2023 1:00	9	8.0	10.4	2.4
ICU	3/15/2023 2:00	9	8.0	10.4	2.4
ICU	3/15/2023 3:00	9	8.0	10.4	2.4
ICU	3/15/2023 4:00	9	8.0	10.4	2.4
ICU	3/15/2023 5:00	9	8.0	10.4	2.4
ICU	3/15/2023 6:00	9	8.0	10.4	2.4
ICU	3/15/2023 7:00	9	8.0	10.4	2.4
ICU	3/15/2023 8:00	9	8.0	10.4	2.4
ICU	3/15/2023 9:00	9	8.0	10.4	2.4
ICU	3/15/2023 10:00	9	8.0	10.4	2.4
ICU	3/15/2023 11:00	9	8.0	10.4	2.4
ICU	3/15/2023 12:00	9	8.0	10.4	2.4
ICU	3/15/2023 13:00	9	8.0	10.4	2.4
ICU	3/15/2023 14:00	9	8.0	10.4	2.4
ICU	3/15/2023 15:00	9	8.0	10.4	2.4
ICU	3/15/2023 16:00	9	8.0	8.9	0.9
ICU	3/15/2023 17:00	9	8.0	8.9	0.9
ICU	3/15/2023 18:00	9	8.0	8.9	0.9
ICU	3/15/2023 19:00	9	8.0	8.9	0.9
ICU	3/15/2023 20:00	13	11.0	14.2	3.2

ICU	3/15/2023 21:00	13	11.0	14.2	3.2
ICU	3/15/2023 22:00	13	12.0	13.1	1.1
ICU	3/15/2023 23:00	13	12.0	14.2	2.2
ICU	3/16/2023 0:00	13	12.0	13.3	1.3
ICU	3/16/2023 1:00	13	12.0	13.3	1.3
ICU	3/16/2023 2:00	13	12.0	13.3	1.3
ICU	3/16/2023 3:00	13	12.0	13.3	1.3
ICU	3/16/2023 4:00	13	12.0	13.3	1.3
ICU	3/16/2023 5:00	13	12.0	13.3	1.3
ICU	3/16/2023 6:00	13	12.0	13.3	1.3
ICU	3/16/2023 7:00	13	12.0	13.3	1.3
ICU	3/16/2023 8:00	12	13.0	11.9	-1.2
ICU	3/16/2023 9:00	12	13.0	11.9	-1.2
ICU	3/16/2023 10:00	12	13.0	11.9	-1.2
ICU	3/16/2023 11:00	10	13.0	11.9	-1.2
ICU	3/16/2023 12:00	12	13.0	11.9	-1.2
ICU	3/16/2023 13:00	12	13.0	11.9	-1.2
ICU	3/16/2023 14:00	12	13.0	11.9	-1.2
ICU	3/16/2023 15:00	11	11.0	11.9	0.9
ICU	3/16/2023 16:00	11	11.0	10.4	-0.6
ICU	3/16/2023 17:00	10	10.0	10.4	0.4
ICU	3/16/2023 18:00	10	10.0	10.4	0.4
ICU	3/16/2023 19:00	10	10.0	10.4	0.4
ICU	3/16/2023 20:00	10	11.0	12.6	1.6
ICU	3/16/2023 21:00	10	11.0	12.6	1.6
ICU	3/16/2023 22:00	10	11.0	12.6	1.6
ICU	3/16/2023 23:00	10	11.0	12.6	1.6
ICU	3/17/2023 0:00	10	11.0	12.6	1.6
ICU	3/17/2023 1:00	10	11.0	12.6	1.6
ICU	3/17/2023 2:00	10	11.0	12.6	1.6
ICU	3/17/2023 3:00	10	10.0	12.6	2.6
ICU	3/17/2023 4:00	10	10.0	12.6	2.6
ICU	3/17/2023 5:00	10	10.0	12.6	2.6
ICU	3/17/2023 6:00	10	10.0	12.6	2.6
ICU	3/17/2023 7:00	10	10.0	12.6	2.6
ICU	3/17/2023 8:00	10	11.0	9.5	-1.5
ICU	3/17/2023 9:00	10	11.0	9.5	-1.5
ICU	3/17/2023 10:00	10	11.0	9.5	-1.5
ICU	3/17/2023 11:00	11	11.0	9.5	-1.5
ICU	3/17/2023 12:00	11	11.0	9.5	-1.5
ICU	3/17/2023 13:00	11	11.0	9.5	-1.5
ICU	3/17/2023 14:00	11	11.0	9.5	-1.5
ICU	3/17/2023 15:00	9	11.0	9.5	-1.5
ICU	3/17/2023 16:00	9	10.0	9.5	-0.5
ICU	3/17/2023 17:00	9	10.0	9.5	-0.5
ICU	3/17/2023 18:00	9	10.0	9.5	-0.5
ICU	3/17/2023 19:00	9	10.0	9.5	-0.5

ICU	3/17/2023 20:00	9	11.0	12.6	1.6
ICU	3/17/2023 21:00	9	11.0	12.6	1.6
ICU	3/17/2023 22:00	9	11.0	12.6	1.6
ICU	3/17/2023 23:00	9	11.0	12.6	1.6
ICU	3/18/2023 0:00	9	11.0	10.4	-0.6
ICU	3/18/2023 1:00	9	11.0	10.4	-0.6
ICU	3/18/2023 2:00	9	11.0	10.4	-0.6
ICU	3/18/2023 3:00	9	11.0	10.4	-0.6
ICU	3/18/2023 4:00	9	11.0	10.4	-0.6
ICU	3/18/2023 5:00	9	11.0	10.4	-0.6
ICU	3/18/2023 6:00	9	11.0	10.4	-0.6
ICU	3/18/2023 7:00	9	11.0	10.4	-0.6
ICU	3/18/2023 8:00	9	11.0	9.5	-1.5
ICU	3/18/2023 9:00	9	11.0	9.5	-1.5
ICU	3/18/2023 10:00	9	11.0	9.5	-1.5
ICU	3/18/2023 11:00	9	11.0	9.5	-1.5
ICU	3/18/2023 12:00	9	11.0	11.0	0.0
ICU	3/18/2023 13:00	8	11.0	11.0	0.0
ICU	3/18/2023 14:00	8	11.0	11.0	0.0
ICU	3/18/2023 15:00	7	11.0	11.0	0.0
ICU	3/18/2023 16:00	8	11.0	9.5	-1.5
ICU	3/18/2023 17:00	8	11.0	9.5	-1.5
ICU	3/18/2023 18:00	8	11.0	9.5	-1.5
ICU	3/18/2023 19:00	8	11.0	9.5	-1.5
ICU	3/18/2023 20:00	9	11.0	8.4	-2.7
ICU	3/18/2023 21:00	9	11.0	9.5	-1.5
ICU	3/18/2023 22:00	9	11.0	9.5	-1.5
ICU	3/18/2023 23:00	9	11.0	9.5	-1.5
ICU	3/19/2023 0:00	9	11.0	11.0	0.0
ICU	3/19/2023 1:00	9	11.0	11.0	0.0
ICU	3/19/2023 2:00	9	11.0	11.0	0.0
ICU	3/19/2023 3:00	9	11.0	11.0	0.0
ICU	3/19/2023 4:00	9	11.0	11.0	0.0
ICU	3/19/2023 5:00	9	11.0	11.0	0.0
ICU	3/19/2023 6:00	9	11.0	11.0	0.0
ICU	3/19/2023 7:00	9	11.0	11.0	0.0
ICU	3/19/2023 8:00	9	9.0	8.9	-0.1
ICU	3/19/2023 9:00	9	9.0	8.9	-0.1
ICU	3/19/2023 10:00	9	9.0	8.9	-0.1
ICU	3/19/2023 11:00	9	9.0	8.9	-0.1
ICU	3/19/2023 12:00	9	9.0	10.4	1.4
ICU	3/19/2023 13:00	9	9.0	10.4	1.4
ICU	3/19/2023 14:00	9	9.0	10.4	1.4
ICU	3/19/2023 15:00	9	9.0	10.4	1.4
ICU	3/19/2023 16:00	8	9.0	9.5	0.5
ICU	3/19/2023 17:00	8	9.0	9.5	0.5
ICU	3/19/2023 18:00	8	9.0	9.5	0.5

ICU	3/19/2023 19:00	9	9.0	9.5	0.5
ICU	3/19/2023 20:00	9	9.0	7.2	-1.8
ICU	3/19/2023 21:00	9	9.0	9.5	0.5
ICU	3/19/2023 22:00	9	9.0	9.5	0.5
ICU	3/19/2023 23:00	9	9.0	9.5	0.5
ICU	3/20/2023 0:00	9	9.0	8.9	-0.1
ICU	3/20/2023 1:00	9	9.0	8.9	-0.1
ICU	3/20/2023 2:00	9	9.0	8.9	-0.1
ICU	3/20/2023 3:00	9	9.0	8.9	-0.1
ICU	3/20/2023 4:00	9	9.0	8.9	-0.1
ICU	3/20/2023 5:00	9	9.0	8.9	-0.1
ICU	3/20/2023 6:00	9	9.0	8.9	-0.1
ICU	3/20/2023 7:00	9	9.0	8.9	-0.1
ICU	3/20/2023 8:00	9	9.0	11.0	2.0
ICU	3/20/2023 9:00	9	9.0	11.0	2.0
ICU	3/20/2023 10:00	9	9.0	11.0	2.0
ICU	3/20/2023 11:00	9	9.0	11.0	2.0
ICU	3/20/2023 12:00	9	9.0	11.0	2.0
ICU	3/20/2023 13:00	9	9.0	11.0	2.0
ICU	3/20/2023 14:00	9	9.0	11.0	2.0
ICU	3/20/2023 15:00	9	9.0	10.3	1.3
ICU	3/20/2023 16:00	8	9.0	9.5	0.5
ICU	3/20/2023 17:00	8	9.0	9.5	0.5
ICU	3/20/2023 18:00	8	9.0	9.5	0.5
ICU	3/20/2023 19:00	8	9.0	9.5	0.5
ICU	3/20/2023 20:00	9	11.0	11.0	0.0
ICU	3/20/2023 21:00	9	11.0	9.9	-1.1
ICU	3/20/2023 22:00	9	11.0	11.0	0.0
ICU	3/20/2023 23:00	9	11.0	11.0	0.0
ICU	3/21/2023 0:00	9	11.0	11.0	0.0
ICU	3/21/2023 1:00	9	11.0	11.0	0.0
ICU	3/21/2023 2:00	9	11.0	11.0	0.0
ICU	3/21/2023 3:00	9	11.0	11.0	0.0
ICU	3/21/2023 4:00	9	11.0	11.0	0.0
ICU	3/21/2023 5:00	9	11.0	11.0	0.0
ICU	3/21/2023 6:00	9	11.0	11.0	0.0
ICU	3/21/2023 7:00	9	11.0	11.0	0.0
ICU	3/21/2023 8:00	9	11.0	11.0	0.0
ICU	3/21/2023 9:00	10	11.0	11.0	0.0
ICU	3/21/2023 10:00	10	11.0	11.0	0.0
ICU	3/21/2023 11:00	10	11.0	11.0	0.0
ICU	3/21/2023 12:00	10	11.0	11.0	0.0
ICU	3/21/2023 13:00	10	11.0	11.0	0.0
ICU	3/21/2023 14:00	10	11.0	11.0	0.0
ICU	3/21/2023 15:00	10	11.0	11.0	0.0
ICU	3/21/2023 16:00	10	11.0	11.0	0.0
ICU	3/21/2023 17:00	10	11.0	11.0	0.0

ICU	3/21/2023 18:00	10	11.0	11.0	0.0
ICU	3/21/2023 19:00	10	11.0	11.0	0.0
ICU	3/21/2023 20:00	10	11.0	11.0	0.0
ICU	3/21/2023 21:00	10	11.0	11.0	0.0
ICU	3/21/2023 22:00	10	11.0	11.0	0.0
ICU	3/21/2023 23:00	13	11.0	11.0	0.0
ICU	3/22/2023 0:00	13	11.0	10.4	-0.6
ICU	3/22/2023 1:00	13	11.0	10.4	-0.6
ICU	3/22/2023 2:00	13	11.0	10.4	-0.6
ICU	3/22/2023 3:00	13	11.0	10.4	-0.6
ICU	3/22/2023 4:00	13	11.0	10.4	-0.6
ICU	3/22/2023 5:00	13	11.0	10.4	-0.6
ICU	3/22/2023 6:00	13	11.0	10.4	-0.6
ICU	3/22/2023 7:00	13	11.0	10.4	-0.6
ICU	3/22/2023 8:00	13	13.0	12.6	-0.4
ICU	3/22/2023 9:00	11	13.0	12.6	-0.4
ICU	3/22/2023 10:00	11	13.0	12.6	-0.4
ICU	3/22/2023 11:00	11	13.0	12.6	-0.4
ICU	3/22/2023 12:00	12	13.0	12.6	-0.4
ICU	3/22/2023 13:00	12	13.0	12.6	-0.4
ICU	3/22/2023 14:00	11	13.0	12.6	-0.4
ICU	3/22/2023 15:00	11	13.0	12.6	-0.4
ICU	3/22/2023 16:00	11	13.0	9.5	-3.5
ICU	3/22/2023 17:00	11	13.0	9.5	-3.5
ICU	3/22/2023 18:00	10	13.0	8.4	-4.7
ICU	3/22/2023 19:00	10	13.0	9.5	-3.5
ICU	3/22/2023 20:00	11	13.0	14.2	1.2
ICU	3/22/2023 21:00	11	13.0	14.2	1.2
ICU	3/22/2023 22:00	12	13.0	14.2	1.2
ICU	3/22/2023 23:00	12	13.0	14.2	1.2
ICU	3/23/2023 0:00	13	13.0	14.2	1.2
ICU	3/23/2023 1:00	13	13.0	14.2	1.2
ICU	3/23/2023 2:00	13	13.0	14.2	1.2
ICU	3/23/2023 3:00	13	13.0	14.2	1.2
ICU	3/23/2023 4:00	13	13.0	14.2	1.2
ICU	3/23/2023 5:00	13	13.0	14.2	1.2
ICU	3/23/2023 6:00	13	13.0	14.2	1.2
ICU	3/23/2023 7:00	13	14.0	14.2	0.2
ICU	3/23/2023 8:00	13	14.0	14.2	0.2
ICU	3/23/2023 9:00	13	14.0	14.2	0.2
ICU	3/23/2023 10:00	13	14.0	14.2	0.2
ICU	3/23/2023 11:00	14	14.0	14.2	0.2
ICU	3/23/2023 12:00	13	14.0	13.1	-0.9
ICU	3/23/2023 13:00	13	14.0	14.2	0.2
ICU	3/23/2023 14:00	12	14.0	13.1	-0.9
ICU	3/23/2023 15:00	12	14.0	14.2	0.2
ICU	3/23/2023 16:00	13	14.0	9.9	-4.1

ICU	3/23/2023 17:00	12	14.0	9.9	-4.1
ICU	3/23/2023 18:00	13	14.0	9.9	-4.1
ICU	3/23/2023 19:00	13	14.0	11.0	-3.0
ICU	3/23/2023 20:00	13	14.0	14.2	0.2
ICU	3/23/2023 21:00	13	14.0	12.0	-2.0
ICU	3/23/2023 22:00	13	14.0	14.2	0.2
ICU	3/23/2023 23:00	13	14.0	14.2	0.2
ICU	3/24/2023 0:00	13	14.0	14.2	0.2
ICU	3/24/2023 1:00	13	14.0	14.2	0.2
ICU	3/24/2023 2:00	13	14.0	14.2	0.2
ICU	3/24/2023 3:00	13	14.0	14.2	0.2
ICU	3/24/2023 4:00	13	14.0	14.2	0.2
ICU	3/24/2023 5:00	13	14.0	14.2	0.2
ICU	3/24/2023 6:00	13	14.0	14.2	0.2
ICU	3/24/2023 7:00	13	14.0	14.2	0.2
ICU	3/24/2023 8:00	13	14.0	12.6	-1.4
ICU	3/24/2023 9:00	13	14.0	12.6	-1.4
ICU	3/24/2023 10:00	13	14.0	12.6	-1.4
ICU	3/24/2023 11:00	13	14.0	12.6	-1.4
ICU	3/24/2023 12:00	13	14.0	12.6	-1.4
ICU	3/24/2023 13:00	13	14.0	12.6	-1.4
ICU	3/24/2023 14:00	13	14.0	12.6	-1.4
ICU	3/24/2023 15:00	13	14.0	12.6	-1.4
ICU	3/24/2023 16:00	13	14.0	11.0	-3.0
ICU	3/24/2023 17:00	13	14.0	11.0	-3.0
ICU	3/24/2023 18:00	13	14.0	11.0	-3.0
ICU	3/24/2023 19:00	13	14.0	11.0	-3.0
ICU	3/24/2023 20:00	13	14.0	9.5	-4.5
ICU	3/24/2023 21:00	13	14.0	9.5	-4.5
ICU	3/24/2023 22:00	13	14.0	9.5	-4.5
ICU	3/24/2023 23:00	13	14.0	9.5	-4.5
ICU	3/25/2023 0:00	13	14.0	11.0	-3.0
ICU	3/25/2023 1:00	13	14.0	11.0	-3.0
ICU	3/25/2023 2:00	9	12.0	11.0	-1.0
ICU	3/25/2023 3:00	9	12.0	11.0	-1.0
ICU	3/25/2023 4:00	9	12.0	11.0	-1.0
ICU	3/25/2023 5:00	9	12.0	11.0	-1.0
ICU	3/25/2023 6:00	9	12.0	11.0	-1.0
ICU	3/25/2023 7:00	9	12.0	11.0	-1.0
ICU	3/25/2023 8:00	9	12.0	11.0	-1.0
ICU	3/25/2023 9:00	9	12.0	11.0	-1.0
ICU	3/25/2023 10:00	9	12.0	11.0	-1.0
ICU	3/25/2023 11:00	9	12.0	11.0	-1.0
ICU	3/25/2023 12:00	9	12.0	11.0	-1.0
ICU	3/25/2023 13:00	9	12.0	11.0	-1.0
ICU	3/25/2023 14:00	9	12.0	11.0	-1.0
ICU	3/25/2023 15:00	9	12.0	11.0	-1.0

ICU	3/25/2023 16:00	9	12.0	11.0	-1.0
ICU	3/25/2023 17:00	9	12.0	11.0	-1.0
ICU	3/25/2023 18:00	9	12.0	11.0	-1.0
ICU	3/25/2023 19:00	9	12.0	11.0	-1.0
ICU	3/25/2023 20:00	9	12.0	7.9	-4.1
ICU	3/25/2023 21:00	7	10.0	7.9	-2.1
ICU	3/25/2023 22:00	7	10.0	7.9	-2.1
ICU	3/25/2023 23:00	7	10.0	7.9	-2.1
ICU	3/26/2023 0:00	7	10.0	9.5	-0.5
ICU	3/26/2023 1:00	7	10.0	9.5	-0.5
ICU	3/26/2023 2:00	7	10.0	9.5	-0.5
ICU	3/26/2023 3:00	7	10.0	9.5	-0.5
ICU	3/26/2023 4:00	7	10.0	9.5	-0.5
ICU	3/26/2023 5:00	7	10.0	9.5	-0.5
ICU	3/26/2023 6:00	7	10.0	9.5	-0.5
ICU	3/26/2023 7:00	7	10.0	9.5	-0.5
ICU	3/26/2023 8:00	7	10.0	12.6	2.6
ICU	3/26/2023 9:00	7	10.0	12.6	2.6
ICU	3/26/2023 10:00	7	10.0	12.6	2.6
ICU	3/26/2023 11:00	7	10.0	12.6	2.6
ICU	3/26/2023 12:00	7	10.0	12.6	2.6
ICU	3/26/2023 13:00	7	10.0	12.6	2.6
ICU	3/26/2023 14:00	7	10.0	12.6	2.6
ICU	3/26/2023 15:00	7	10.0	12.6	2.6
ICU	3/26/2023 16:00	7	10.0	12.6	2.6
ICU	3/26/2023 17:00	7	10.0	12.6	2.6
ICU	3/26/2023 18:00	7	10.0	12.6	2.6
ICU	3/26/2023 19:00	9	10.0	12.6	2.6
ICU	3/26/2023 20:00	9	10.0	12.6	2.6
ICU	3/26/2023 21:00	9	10.0	12.6	2.6
ICU	3/26/2023 22:00	9	10.0	12.6	2.6
ICU	3/26/2023 23:00	9	10.0	12.6	2.6
ICU	3/27/2023 0:00	9	10.0	12.6	2.6
ICU	3/27/2023 1:00	9	10.0	12.6	2.6
ICU	3/27/2023 2:00	9	10.0	12.6	2.6
ICU	3/27/2023 3:00	9	10.0	12.6	2.6
ICU	3/27/2023 4:00	9	10.0	12.6	2.6
ICU	3/27/2023 5:00	9	10.0	12.6	2.6
ICU	3/27/2023 6:00	9	10.0	12.6	2.6
ICU	3/27/2023 7:00	9	10.0	12.6	2.6
ICU	3/27/2023 8:00	9	10.0	11.0	1.0
ICU	3/27/2023 9:00	9	10.0	11.0	1.0
ICU	3/27/2023 10:00	9	10.0	11.0	1.0
ICU	3/27/2023 11:00	9	10.0	11.0	1.0
ICU	3/27/2023 12:00	9	10.0	11.0	1.0
ICU	3/27/2023 13:00	9	10.0	11.0	1.0
ICU	3/27/2023 14:00	9	10.0	11.0	1.0

ICU	3/27/2023 15:00	9	10.0	11.0	1.0
ICU	3/27/2023 16:00	9	10.0	11.0	1.0
ICU	3/27/2023 17:00	9	10.0	11.0	1.0
ICU	3/27/2023 18:00	9	10.0	11.0	1.0
ICU	3/27/2023 19:00	9	10.0	11.0	1.0
ICU	3/27/2023 20:00	9	12.0	11.0	-1.0
ICU	3/27/2023 21:00	9	12.0	11.0	-1.0
ICU	3/27/2023 22:00	9	12.0	11.0	-1.0
ICU	3/27/2023 23:00	9	12.0	11.0	-1.0
ICU	3/28/2023 0:00	9	12.0	12.6	0.6
ICU	3/28/2023 1:00	9	12.0	12.6	0.6
ICU	3/28/2023 2:00	11	12.0	12.6	0.6
ICU	3/28/2023 3:00	11	12.0	11.5	-0.5
ICU	3/28/2023 4:00	11	12.0	12.6	0.6
ICU	3/28/2023 5:00	11	12.0	12.6	0.6
ICU	3/28/2023 6:00	11	12.0	12.6	0.6
ICU	3/28/2023 7:00	11	12.0	12.6	0.6
ICU	3/28/2023 8:00	11	13.0	12.6	-0.4
ICU	3/28/2023 9:00	12	13.0	11.5	-1.5
ICU	3/28/2023 10:00	11	13.0	11.5	-1.5
ICU	3/28/2023 11:00	11	13.0	12.6	-0.4
ICU	3/28/2023 12:00	11	13.0	12.6	-0.4
ICU	3/28/2023 13:00	13	13.0	12.6	-0.4
ICU	3/28/2023 14:00	13	13.0	12.6	-0.4
ICU	3/28/2023 15:00	13	13.0	12.6	-0.4
ICU	3/28/2023 16:00	13	13.0	12.6	-0.4
ICU	3/28/2023 17:00	13	13.0	12.6	-0.4
ICU	3/28/2023 18:00	13	14.0	12.6	-1.4
ICU	3/28/2023 19:00	13	14.0	12.6	-1.4
ICU	3/28/2023 20:00	13	14.0	15.0	1.0
ICU	3/28/2023 21:00	13	14.0	15.0	1.0
ICU	3/28/2023 22:00	14	14.0	13.9	-0.1
ICU	3/28/2023 23:00	14	14.0	15.0	1.0
ICU	3/29/2023 0:00	14	14.0	14.2	0.2
ICU	3/29/2023 1:00	14	14.0	14.2	0.2
ICU	3/29/2023 2:00	14	14.0	14.2	0.2
ICU	3/29/2023 3:00	14	14.0	14.2	0.2
ICU	3/29/2023 4:00	14	14.0	14.2	0.2
ICU	3/29/2023 5:00	14	14.0	14.2	0.2
ICU	3/29/2023 6:00	14	14.0	14.2	0.2
ICU	3/29/2023 7:00	14	14.0	14.2	0.2
ICU	3/29/2023 8:00	14	14.0	12.6	-1.4
ICU	3/29/2023 9:00	14	14.0	12.6	-1.4
ICU	3/29/2023 10:00	14	14.0	12.6	-1.4
ICU	3/29/2023 11:00	14	14.0	12.6	-1.4
ICU	3/29/2023 12:00	14	14.0	12.6	-1.4
ICU	3/29/2023 13:00	14	14.0	12.6	-1.4

ICU	3/29/2023 14:00	14	14.0	15.0	1.0
ICU	3/29/2023 15:00	14	14.0	15.0	1.0
ICU	3/29/2023 16:00	14	14.0	14.2	0.2
ICU	3/29/2023 17:00	14	14.0	14.2	0.2
ICU	3/29/2023 18:00	14	14.0	14.2	0.2
ICU	3/29/2023 19:00	14	14.0	14.2	0.2
ICU	3/29/2023 20:00	14	14.0	12.6	-1.4
ICU	3/29/2023 21:00	14	14.0	12.6	-1.4
ICU	3/29/2023 22:00	11	12.0	12.6	0.6
ICU	3/29/2023 23:00	11	12.0	12.6	0.6
ICU	3/30/2023 0:00	11	12.0	12.6	0.6
ICU	3/30/2023 1:00	11	12.0	12.6	0.6
ICU	3/30/2023 2:00	11	12.0	12.6	0.6
ICU	3/30/2023 3:00	11	12.0	12.6	0.6
ICU	3/30/2023 4:00	11	12.0	12.6	0.6
ICU	3/30/2023 5:00	11	12.0	12.6	0.6
ICU	3/30/2023 6:00	11	12.0	12.6	0.6
ICU	3/30/2023 7:00	11	12.0	12.6	0.6
ICU	3/30/2023 8:00	11	12.0	14.2	2.2
ICU	3/30/2023 9:00	11	12.0	14.2	2.2
ICU	3/30/2023 10:00	11	12.0	14.2	2.2
ICU	3/30/2023 11:00	11	12.0	14.2	2.2
ICU	3/30/2023 12:00	11	12.0	14.2	2.2
ICU	3/30/2023 13:00	11	12.0	14.2	2.2
ICU	3/30/2023 14:00	11	12.0	14.2	2.2
ICU	3/30/2023 15:00	11	12.0	14.2	2.2
ICU	3/30/2023 16:00	11	12.0	14.2	2.2
ICU	3/30/2023 17:00	11	12.0	14.2	2.2
ICU	3/30/2023 18:00	11	12.0	14.2	2.2
ICU	3/30/2023 19:00	11	12.0	14.2	2.2
ICU	3/30/2023 20:00	10	12.0	8.4	-3.7
ICU	3/30/2023 21:00	10	12.0	11.0	-1.0
ICU	3/30/2023 22:00	10	12.0	11.0	-1.0
ICU	3/30/2023 23:00	10	12.0	11.0	-1.0
ICU	3/31/2023 0:00	10	12.0	10.4	-1.6
ICU	3/31/2023 1:00	10	12.0	10.4	-1.6
ICU	3/31/2023 2:00	10	12.0	10.4	-1.6
ICU	3/31/2023 3:00	10	12.0	10.4	-1.6
ICU	3/31/2023 4:00	10	12.0	10.4	-1.6
ICU	3/31/2023 5:00	10	12.0	10.4	-1.6
ICU	3/31/2023 6:00	10	12.0	10.4	-1.6
ICU	3/31/2023 7:00	10	12.0	10.4	-1.6
ICU	3/31/2023 8:00	10	12.0	11.0	-1.0
ICU	3/31/2023 9:00	10	12.0	11.0	-1.0
ICU	3/31/2023 10:00	10	12.0	11.0	-1.0
ICU	3/31/2023 11:00	10	12.0	11.0	-1.0
ICU	3/31/2023 12:00	10	12.0	11.0	-1.0

ICU	3/31/2023 13:00	10	12.0	11.0	-1.0
ICU	3/31/2023 14:00	10	12.0	11.0	-1.0
ICU	3/31/2023 15:00	10	12.0	11.0	-1.0
ICU	3/31/2023 16:00	10	12.0	11.0	-1.0
ICU	3/31/2023 17:00	10	12.0	11.0	-1.0
ICU	3/31/2023 18:00	10	12.0	11.0	-1.0
ICU	3/31/2023 19:00	10	12.0	11.0	-1.0
ICU	3/31/2023 20:00	10	9.0	12.6	3.6
ICU	3/31/2023 21:00	10	9.0	12.6	3.6
ICU	3/31/2023 22:00	10	9.0	12.6	3.6
ICU	3/31/2023 23:00	10	12.0	11.5	-0.5
ICU	4/1/2023 0:00	10	12.0	11.9	-0.2
ICU	4/1/2023 1:00	10	12.0	11.9	-0.2
ICU	4/1/2023 2:00	10	12.0	11.9	-0.2
ICU	4/1/2023 3:00	11	12.0	10.7	-1.3
ICU	4/1/2023 4:00	11	12.0	11.9	-0.2
ICU	4/1/2023 5:00	11	12.0	11.9	-0.2
ICU	4/1/2023 6:00	11	12.0	11.9	-0.2
ICU	4/1/2023 7:00	11	12.0	11.9	-0.2
ICU	4/1/2023 8:00	11	12.0	12.6	0.6
ICU	4/1/2023 9:00	11	12.0	12.6	0.6
ICU	4/1/2023 10:00	11	12.0	12.6	0.6
ICU	4/1/2023 11:00	11	12.0	12.6	0.6
ICU	4/1/2023 12:00	11	12.0	12.6	0.6
ICU	4/1/2023 13:00	11	12.0	12.6	0.6
ICU	4/1/2023 14:00	11	12.0	12.6	0.6
ICU	4/1/2023 15:00	11	12.0	12.6	0.6
ICU	4/1/2023 16:00	10	12.0	11.0	-1.0
ICU	4/1/2023 17:00	9	12.0	11.0	-1.0
ICU	4/1/2023 18:00	10	12.0	11.0	-1.0
ICU	4/1/2023 19:00	10	12.0	11.0	-1.0
ICU	4/1/2023 20:00	10	12.0	12.6	0.6
ICU	4/1/2023 21:00	10	12.0	12.6	0.6
ICU	4/1/2023 22:00	10	12.0	12.6	0.6
ICU	4/1/2023 23:00	10	12.0	12.6	0.6
ICU	4/2/2023 0:00	10	12.0	11.9	-0.2
ICU	4/2/2023 1:00	9	12.0	9.6	-2.4
ICU	4/2/2023 2:00	9	12.0	11.9	-0.2
ICU	4/2/2023 3:00	11	12.0	10.7	-1.3
ICU	4/2/2023 4:00	11	12.0	11.9	-0.2
ICU	4/2/2023 5:00	11	12.0	11.9	-0.2
ICU	4/2/2023 6:00	11	12.0	11.9	-0.2
ICU	4/2/2023 7:00	11	12.0	11.9	-0.2
ICU	4/2/2023 8:00	10	12.0	12.6	0.6
ICU	4/2/2023 9:00	10	12.0	12.6	0.6
ICU	4/2/2023 10:00	10	12.0	12.6	0.6
ICU	4/2/2023 11:00	10	12.0	12.6	0.6

ICU	4/2/2023 12:00	10	12.0	12.6	0.6
ICU	4/2/2023 13:00	11	12.0	12.6	0.6
ICU	4/2/2023 14:00	10	12.0	12.6	0.6
ICU	4/2/2023 15:00	10	12.0	12.6	0.6
ICU	4/2/2023 16:00	10	12.0	10.4	-1.6
ICU	4/2/2023 17:00	10	12.0	10.4	-1.6
ICU	4/2/2023 18:00	10	12.0	10.4	-1.6
ICU	4/2/2023 19:00	10	12.0	10.4	-1.6
ICU	4/2/2023 20:00	11	11.0	8.9	-2.1
ICU	4/2/2023 21:00	11	11.0	8.9	-2.1
ICU	4/2/2023 22:00	11	11.0	8.9	-2.1
ICU	4/2/2023 23:00	11	11.0	8.9	-2.1
ICU	4/3/2023 0:00	11	11.0	9.5	-1.5
ICU	4/3/2023 1:00	11	11.0	9.5	-1.5
ICU	4/3/2023 2:00	11	11.0	9.5	-1.5
ICU	4/3/2023 3:00	11	11.0	9.5	-1.5
ICU	4/3/2023 4:00	11	11.0	9.5	-1.5
ICU	4/3/2023 5:00	11	11.0	9.5	-1.5
ICU	4/3/2023 6:00	11	11.0	9.5	-1.5
ICU	4/3/2023 7:00	11	11.0	9.5	-1.5
ICU	4/3/2023 8:00	11	11.0	11.0	0.0
ICU	4/3/2023 9:00	11	11.0	11.0	0.0
ICU	4/3/2023 10:00	11	11.0	11.0	0.0
ICU	4/3/2023 11:00	11	11.0	11.0	0.0
ICU	4/3/2023 12:00	11	11.0	12.6	1.6
ICU	4/3/2023 13:00	10	11.0	12.6	1.6
ICU	4/3/2023 14:00	10	11.0	12.6	1.6
ICU	4/3/2023 15:00	10	11.0	12.6	1.6
ICU	4/3/2023 16:00	10	10.0	11.0	1.0
ICU	4/3/2023 17:00	10	10.0	11.0	1.0
ICU	4/3/2023 18:00	10	10.0	11.0	1.0
ICU	4/3/2023 19:00	10	10.0	11.0	1.0
ICU	4/3/2023 20:00	10	10.0	11.0	1.0
ICU	4/3/2023 21:00	10	10.0	11.0	1.0
ICU	4/3/2023 22:00	10	10.0	11.0	1.0
ICU	4/3/2023 23:00	10	10.0	11.0	1.0
ICU	4/4/2023 0:00	10	10.0	11.0	1.0
ICU	4/4/2023 1:00	10	10.0	11.0	1.0
ICU	4/4/2023 2:00	10	10.0	11.0	1.0
ICU	4/4/2023 3:00	10	10.0	11.0	1.0
ICU	4/4/2023 4:00	10	10.0	11.0	1.0
ICU	4/4/2023 5:00	10	10.0	11.0	1.0
ICU	4/4/2023 6:00	10	10.0	11.0	1.0
ICU	4/4/2023 7:00	10	10.0	11.0	1.0
ICU	4/4/2023 8:00	10	12.0	12.6	0.6
ICU	4/4/2023 9:00	10	12.0	12.6	0.6
ICU	4/4/2023 10:00	10	12.0	12.6	0.6

ICU	4/4/2023 11:00	11	12.0	12.6	0.6
ICU	4/4/2023 12:00	11	12.0	12.6	0.6
ICU	4/4/2023 13:00	11	12.0	12.6	0.6
ICU	4/4/2023 14:00	10	12.0	12.6	0.6
ICU	4/4/2023 15:00	10	12.0	11.8	-0.2
ICU	4/4/2023 16:00	10	12.0	8.9	-3.1
ICU	4/4/2023 17:00	10	12.0	8.9	-3.1
ICU	4/4/2023 18:00	7	10.0	8.9	-1.1
ICU	4/4/2023 19:00	7	10.0	8.9	-1.1
ICU	4/4/2023 20:00	8	12.0	10.4	-1.6
ICU	4/4/2023 21:00	8	12.0	10.4	-1.6
ICU	4/4/2023 22:00	8	12.0	10.4	-1.6
ICU	4/4/2023 23:00	8	12.0	10.4	-1.6
ICU	4/5/2023 0:00	8	12.0	10.4	-1.6
ICU	4/5/2023 1:00	8	12.0	10.4	-1.6
ICU	4/5/2023 2:00	8	12.0	10.4	-1.6
ICU	4/5/2023 3:00	8	12.0	10.4	-1.6
ICU	4/5/2023 4:00	8	12.0	10.4	-1.6
ICU	4/5/2023 5:00	8	12.0	10.4	-1.6
ICU	4/5/2023 6:00	8	12.0	10.4	-1.6
ICU	4/5/2023 7:00	8	12.0	10.4	-1.6
ICU	4/5/2023 8:00	9	12.0	12.6	0.6
ICU	4/5/2023 9:00	9	12.0	12.6	0.6
ICU	4/5/2023 10:00	9	12.0	12.6	0.6
ICU	4/5/2023 11:00	9	12.0	12.6	0.6
ICU	4/5/2023 12:00	9	12.0	14.2	2.2
ICU	4/5/2023 13:00	9	12.0	14.2	2.2
ICU	4/5/2023 14:00	9	12.0	14.2	2.2
ICU	4/5/2023 15:00	10	12.0	14.2	2.2
ICU	4/5/2023 16:00	8	12.0	11.9	-0.2
ICU	4/5/2023 17:00	8	12.0	11.9	-0.2
ICU	4/5/2023 18:00	8	12.0	11.9	-0.2
ICU	4/5/2023 19:00	8	12.0	11.9	-0.2
ICU	4/5/2023 20:00	8	12.0	11.9	-0.2
ICU	4/5/2023 21:00	8	12.0	11.9	-0.2
ICU	4/5/2023 22:00	8	12.0	10.4	-1.6
ICU	4/5/2023 23:00	8	12.0	10.4	-1.6
ICU	4/6/2023 0:00	8	12.0	10.4	-1.6
ICU	4/6/2023 1:00	8	12.0	8.2	-3.9
ICU	4/6/2023 2:00	8	12.0	10.4	-1.6
ICU	4/6/2023 3:00	8	12.0	10.4	-1.6
ICU	4/6/2023 4:00	8	12.0	10.4	-1.6
ICU	4/6/2023 5:00	8	12.0	10.4	-1.6
ICU	4/6/2023 6:00	8	12.0	10.4	-1.6
ICU	4/6/2023 7:00	8	12.0	10.4	-1.6
ICU	4/6/2023 8:00	8	10.0	11.0	1.0
ICU	4/6/2023 9:00	8	10.0	11.0	1.0

ICU	4/6/2023 10:00	8	10.0	11.0	1.0
ICU	4/6/2023 11:00	8	10.0	11.0	1.0
ICU	4/6/2023 12:00	8	10.0	11.0	1.0
ICU	4/6/2023 13:00	7	10.0	11.0	1.0
ICU	4/6/2023 14:00	7	10.0	11.0	1.0
ICU	4/6/2023 15:00	7	10.0	11.0	1.0
ICU	4/6/2023 16:00	7	10.0	7.4	-2.6
ICU	4/6/2023 17:00	7	10.0	7.4	-2.6
ICU	4/6/2023 18:00	6	10.0	8.9	-1.1
ICU	4/6/2023 19:00	6	10.0	8.9	-1.1
ICU	4/6/2023 20:00	6	10.0	8.9	-1.1
ICU	4/6/2023 21:00	5	8.0	7.8	-0.2
ICU	4/6/2023 22:00	5	8.0	8.9	0.9
ICU	4/6/2023 23:00	5	8.0	8.9	0.9
ICU	4/7/2023 0:00	5	8.0	8.9	0.9
ICU	4/7/2023 1:00	5	8.0	8.9	0.9
ICU	4/7/2023 2:00	5	8.0	8.9	0.9
ICU	4/7/2023 3:00	5	8.0	8.9	0.9
ICU	4/7/2023 4:00	5	8.0	8.9	0.9
ICU	4/7/2023 5:00	5	8.0	8.9	0.9
ICU	4/7/2023 6:00	5	8.0	8.9	0.9
ICU	4/7/2023 7:00	5	8.0	8.9	0.9
ICU	4/7/2023 8:00	5	8.0	12.6	4.6
ICU	4/7/2023 9:00	5	8.0	12.6	4.6
ICU	4/7/2023 10:00	5	8.0	12.6	4.6
ICU	4/7/2023 11:00	5	8.0	12.6	4.6
ICU	4/7/2023 12:00	5	8.0	14.2	6.2
ICU	4/7/2023 13:00	5	8.0	14.2	6.2
ICU	4/7/2023 14:00	5	8.0	14.2	6.2
ICU	4/7/2023 15:00	5	8.0	14.2	6.2
ICU	4/7/2023 16:00	5	8.0	11.9	3.9
ICU	4/7/2023 17:00	5	8.0	11.9	3.9
ICU	4/7/2023 18:00	5	8.0	11.9	3.9
ICU	4/7/2023 19:00	5	8.0	11.9	3.9
ICU	4/7/2023 20:00	5	12.0	11.9	-0.2
ICU	4/7/2023 21:00	5	12.0	11.9	-0.2
ICU	4/7/2023 22:00	5	12.0	11.9	-0.2
ICU	4/7/2023 23:00	5	12.0	11.9	-0.2
ICU	4/8/2023 0:00	5	12.0	11.9	-0.2
ICU	4/8/2023 1:00	5	12.0	11.9	-0.2
ICU	4/8/2023 2:00	5	12.0	11.9	-0.2
ICU	4/8/2023 3:00	5	12.0	11.9	-0.2
ICU	4/8/2023 4:00	5	12.0	11.9	-0.2
ICU	4/8/2023 5:00	5	12.0	11.9	-0.2
ICU	4/8/2023 6:00	5	12.0	11.9	-0.2
ICU	4/8/2023 7:00	6	10.0	11.9	1.9
ICU	4/8/2023 8:00	6	10.0	8.9	-1.1

ICU	4/8/2023 9:00	6	10.0	8.9	-1.1
ICU	4/8/2023 10:00	6	10.0	8.9	-1.1
ICU	4/8/2023 11:00	6	10.0	8.9	-1.1
ICU	4/8/2023 12:00	6	10.0	8.9	-1.1
ICU	4/8/2023 13:00	6	10.0	8.9	-1.1
ICU	4/8/2023 14:00	6	10.0	8.9	-1.1
ICU	4/8/2023 15:00	6	10.0	8.9	-1.1
ICU	4/8/2023 16:00	6	10.0	9.5	-0.5
ICU	4/8/2023 17:00	6	10.0	9.5	-0.5
ICU	4/8/2023 18:00	6	10.0	9.5	-0.5
ICU	4/8/2023 19:00	6	10.0	9.5	-0.5
ICU	4/8/2023 20:00	9	10.0	12.6	2.6
ICU	4/8/2023 21:00	9	10.0	10.4	0.4
ICU	4/8/2023 22:00	9	10.0	12.6	2.6
ICU	4/8/2023 23:00	9	10.0	12.6	2.6
ICU	4/9/2023 0:00	10	10.0	11.0	1.0
ICU	4/9/2023 1:00	10	10.0	11.0	1.0
ICU	4/9/2023 2:00	10	10.0	11.0	1.0
ICU	4/9/2023 3:00	10	10.0	11.0	1.0
ICU	4/9/2023 4:00	10	10.0	11.0	1.0
ICU	4/9/2023 5:00	10	10.0	11.0	1.0
ICU	4/9/2023 6:00	10	10.0	11.0	1.0
ICU	4/9/2023 7:00	10	10.0	11.0	1.0
ICU	4/9/2023 8:00	10	10.0	10.4	0.4
ICU	4/9/2023 9:00	10	10.0	10.4	0.4
ICU	4/9/2023 10:00	10	10.0	10.4	0.4
ICU	4/9/2023 11:00	10	10.0	10.4	0.4
ICU	4/9/2023 12:00	10	10.0	13.3	3.3
ICU	4/9/2023 13:00	10	10.0	13.3	3.3
ICU	4/9/2023 14:00	10	10.0	13.3	3.3
ICU	4/9/2023 15:00	10	10.0	13.3	3.3
ICU	4/9/2023 16:00	10	10.0	12.6	2.6
ICU	4/9/2023 17:00	10	10.0	12.6	2.6
ICU	4/9/2023 18:00	10	10.0	12.6	2.6
ICU	4/9/2023 19:00	8	11.0	12.6	1.6
ICU	4/9/2023 20:00	8	11.0	9.5	-1.5
ICU	4/9/2023 21:00	8	11.0	9.5	-1.5
ICU	4/9/2023 22:00	8	11.0	9.5	-1.5
ICU	4/9/2023 23:00	8	11.0	9.5	-1.5
ICU	4/10/2023 0:00	8	11.0	8.9	-2.1
ICU	4/10/2023 1:00	10	11.0	8.9	-2.1
ICU	4/10/2023 2:00	10	11.0	8.9	-2.1
ICU	4/10/2023 3:00	10	11.0	8.9	-2.1
ICU	4/10/2023 4:00	10	11.0	8.9	-2.1
ICU	4/10/2023 5:00	10	11.0	8.9	-2.1
ICU	4/10/2023 6:00	10	11.0	8.9	-2.1
ICU	4/10/2023 7:00	10	11.0	8.9	-2.1

ICU	4/10/2023 8:00	10	11.0	11.0	0.0
ICU	4/10/2023 9:00	10	11.0	11.0	0.0
ICU	4/10/2023 10:00	10	11.0	11.0	0.0
ICU	4/10/2023 11:00	10	11.0	11.0	0.0
ICU	4/10/2023 12:00	10	11.0	12.6	1.6
ICU	4/10/2023 13:00	10	11.0	12.6	1.6
ICU	4/10/2023 14:00	10	11.0	12.6	1.6
ICU	4/10/2023 15:00	10	11.0	12.6	1.6
ICU	4/10/2023 16:00	10	11.0	12.6	1.6
ICU	4/10/2023 17:00	10	11.0	12.6	1.6
ICU	4/10/2023 18:00	10	11.0	12.6	1.6
ICU	4/10/2023 19:00	10	11.0	12.6	1.6
ICU	4/10/2023 20:00	9	12.0	11.0	-1.0
ICU	4/10/2023 21:00	8	12.0	9.9	-2.1
ICU	4/10/2023 22:00	8	12.0	11.0	-1.0
ICU	4/10/2023 23:00	8	12.0	11.0	-1.0
ICU	4/11/2023 0:00	8	12.0	8.9	-3.1
ICU	4/11/2023 1:00	8	12.0	8.9	-3.1
ICU	4/11/2023 2:00	8	12.0	8.9	-3.1
ICU	4/11/2023 3:00	8	12.0	8.9	-3.1
ICU	4/11/2023 4:00	8	12.0	8.9	-3.1
ICU	4/11/2023 5:00	8	12.0	8.9	-3.1
ICU	4/11/2023 6:00	8	12.0	8.9	-3.1
ICU	4/11/2023 7:00	8	12.0	8.9	-3.1
ICU	4/11/2023 8:00	8	10.0	11.0	1.0
ICU	4/11/2023 9:00	9	10.0	11.0	1.0
ICU	4/11/2023 10:00	9	10.0	11.0	1.0
ICU	4/11/2023 11:00	9	10.0	11.0	1.0
ICU	4/11/2023 12:00	9	10.0	11.0	1.0
ICU	4/11/2023 13:00	9	10.0	11.0	1.0
ICU	4/11/2023 14:00	10	10.0	11.0	1.0
ICU	4/11/2023 15:00	10	10.0	11.0	1.0
ICU	4/11/2023 16:00	10	10.0	9.5	-0.5
ICU	4/11/2023 17:00	10	10.0	9.5	-0.5
ICU	4/11/2023 18:00	10	10.0	9.5	-0.5
ICU	4/11/2023 19:00	10	10.0	9.5	-0.5
ICU	4/11/2023 20:00	11	12.0	11.5	-0.5
ICU	4/11/2023 21:00	11	12.0	12.6	0.6
ICU	4/11/2023 22:00	11	12.0	11.5	-0.5
ICU	4/11/2023 23:00	10	12.0	12.6	0.6
ICU	4/12/2023 0:00	10	12.0	11.9	-0.2
ICU	4/12/2023 1:00	10	12.0	11.9	-0.2
ICU	4/12/2023 2:00	10	12.0	11.9	-0.2
ICU	4/12/2023 3:00	11	12.0	10.7	-1.3
ICU	4/12/2023 4:00	11	12.0	11.9	-0.2
ICU	4/12/2023 5:00	11	12.0	11.9	-0.2
ICU	4/12/2023 6:00	11	12.0	11.9	-0.2

ICU	4/12/2023 7:00	11	12.0	11.9	-0.2
ICU	4/12/2023 8:00	11	14.0	13.3	-0.7
ICU	4/12/2023 9:00	11	14.0	13.3	-0.7
ICU	4/12/2023 10:00	11	14.0	13.3	-0.7
ICU	4/12/2023 11:00	11	14.0	13.3	-0.7
ICU	4/12/2023 12:00	11	14.0	13.3	-0.7
ICU	4/12/2023 13:00	11	14.0	13.3	-0.7
ICU	4/12/2023 14:00	11	14.0	13.3	-0.7
ICU	4/12/2023 15:00	11	14.0	13.3	-0.7
ICU	4/12/2023 16:00	11	14.0	14.2	0.2
ICU	4/12/2023 17:00	11	14.0	14.2	0.2
ICU	4/12/2023 18:00	11	14.0	14.2	0.2
ICU	4/12/2023 19:00	12	12.0	14.2	2.2
ICU	4/12/2023 20:00	12	12.0	11.0	-1.0
ICU	4/12/2023 21:00	12	12.0	11.0	-1.0
ICU	4/12/2023 22:00	12	12.0	11.0	-1.0
ICU	4/12/2023 23:00	12	12.0	11.0	-1.0
ICU	4/13/2023 0:00	12	12.0	10.4	-1.6
ICU	4/13/2023 1:00	12	12.0	10.4	-1.6
ICU	4/13/2023 2:00	12	12.0	10.4	-1.6
ICU	4/13/2023 3:00	12	12.0	10.4	-1.6
ICU	4/13/2023 4:00	12	12.0	10.4	-1.6
ICU	4/13/2023 5:00	12	12.0	10.4	-1.6
ICU	4/13/2023 6:00	12	12.0	10.4	-1.6
ICU	4/13/2023 7:00	12	12.0	10.4	-1.6
ICU	4/13/2023 8:00	12	12.0	11.9	-0.2
ICU	4/13/2023 9:00	12	12.0	11.9	-0.2
ICU	4/13/2023 10:00	12	12.0	11.9	-0.2
ICU	4/13/2023 11:00	12	12.0	11.9	-0.2
ICU	4/13/2023 12:00	11	12.0	15.0	3.0
ICU	4/13/2023 13:00	10	12.0	15.0	3.0
ICU	4/13/2023 14:00	10	12.0	15.0	3.0
ICU	4/13/2023 15:00	9	12.0	14.5	2.5
ICU	4/13/2023 16:00	9	12.0	11.9	-0.2
ICU	4/13/2023 17:00	9	12.0	11.9	-0.2
ICU	4/13/2023 18:00	9	12.0	11.9	-0.2
ICU	4/13/2023 19:00	9	10.0	11.9	1.9
ICU	4/13/2023 20:00	9	10.0	11.0	1.0
ICU	4/13/2023 21:00	9	10.0	11.0	1.0
ICU	4/13/2023 22:00	9	10.0	11.0	1.0
ICU	4/13/2023 23:00	9	10.0	11.0	1.0
ICU	4/14/2023 0:00	9	10.0	10.4	0.4
ICU	4/14/2023 1:00	9	10.0	10.4	0.4
ICU	4/14/2023 2:00	9	10.0	10.4	0.4
ICU	4/14/2023 3:00	9	10.0	10.4	0.4
ICU	4/14/2023 4:00	10	10.0	9.3	-0.7
ICU	4/14/2023 5:00	10	10.0	10.4	0.4

ICU	4/14/2023 6:00	10	10.0	10.4	0.4
ICU	4/14/2023 7:00	10	10.0	10.4	0.4
ICU	4/14/2023 8:00	10	14.0	13.3	-0.7
ICU	4/14/2023 9:00	10	14.0	13.3	-0.7
ICU	4/14/2023 10:00	10	14.0	13.3	-0.7
ICU	4/14/2023 11:00	10	14.0	13.3	-0.7
ICU	4/14/2023 12:00	10	14.0	14.8	0.8
ICU	4/14/2023 13:00	9	14.0	14.8	0.8
ICU	4/14/2023 14:00	9	14.0	14.8	0.8
ICU	4/14/2023 15:00	9	12.0	13.7	1.7
ICU	4/14/2023 16:00	8	12.0	13.3	1.3
ICU	4/14/2023 17:00	8	12.0	13.3	1.3
ICU	4/14/2023 18:00	8	12.0	13.3	1.3
ICU	4/14/2023 19:00	8	12.0	13.3	1.3
ICU	4/14/2023 20:00	9	10.0	10.4	0.4
ICU	4/14/2023 21:00	9	10.0	10.4	0.4
ICU	4/14/2023 22:00	9	10.0	10.4	0.4
ICU	4/14/2023 23:00	9	10.0	6.7	-3.3
ICU	4/15/2023 0:00	9	10.0	7.4	-2.6
ICU	4/15/2023 1:00	9	10.0	7.4	-2.6
ICU	4/15/2023 2:00	9	10.0	7.4	-2.6
ICU	4/15/2023 3:00	9	10.0	7.4	-2.6
ICU	4/15/2023 4:00	9	10.0	7.4	-2.6
ICU	4/15/2023 5:00	9	10.0	7.4	-2.6
ICU	4/15/2023 6:00	9	10.0	7.4	-2.6
ICU	4/15/2023 7:00	9	10.0	7.4	-2.6
ICU	4/15/2023 8:00	9	14.0	8.9	-5.1
ICU	4/15/2023 9:00	9	14.0	8.9	-5.1
ICU	4/15/2023 10:00	9	14.0	8.9	-5.1
ICU	4/15/2023 11:00	9	14.0	8.9	-5.1
ICU	4/15/2023 12:00	9	14.0	10.4	-3.6
ICU	4/15/2023 13:00	9	14.0	10.4	-3.6
ICU	4/15/2023 14:00	9	14.0	10.4	-3.6
ICU	4/15/2023 15:00	9	14.0	10.4	-3.6
ICU	4/15/2023 16:00	8	12.0	11.9	-0.2
ICU	4/15/2023 17:00	8	12.0	11.9	-0.2
ICU	4/15/2023 18:00	8	12.0	11.9	-0.2
ICU	4/15/2023 19:00	8	12.0	11.9	-0.2
ICU	4/15/2023 20:00	8	10.0	9.5	-0.5
ICU	4/15/2023 21:00	8	10.0	9.5	-0.5
ICU	4/15/2023 22:00	8	10.0	9.5	-0.5
ICU	4/15/2023 23:00	8	10.0	9.5	-0.5
ICU	4/16/2023 0:00	8	10.0	8.9	-1.1
ICU	4/16/2023 1:00	8	10.0	8.9	-1.1
ICU	4/16/2023 2:00	8	10.0	8.9	-1.1
ICU	4/16/2023 3:00	8	10.0	8.9	-1.1
ICU	4/16/2023 4:00	8	10.0	8.9	-1.1

ICU	4/16/2023 5:00	8	10.0	8.9	-1.1
ICU	4/16/2023 6:00	8	10.0	8.9	-1.1
ICU	4/16/2023 7:00	8	10.0	8.9	-1.1
ICU	4/16/2023 8:00	8	10.0	9.5	-0.5
ICU	4/16/2023 9:00	8	10.0	9.5	-0.5
ICU	4/16/2023 10:00	8	10.0	9.5	-0.5
ICU	4/16/2023 11:00	8	10.0	9.5	-0.5
ICU	4/16/2023 12:00	8	10.0	9.5	-0.5
ICU	4/16/2023 13:00	6	10.0	11.9	1.9
ICU	4/16/2023 14:00	6	10.0	11.9	1.9
ICU	4/16/2023 15:00	6	10.0	11.9	1.9
ICU	4/16/2023 16:00	6	10.0	11.0	1.0
ICU	4/16/2023 17:00	6	10.0	11.0	1.0
ICU	4/16/2023 18:00	6	10.0	11.0	1.0
ICU	4/16/2023 19:00	6	10.0	11.0	1.0
ICU	4/16/2023 20:00	5	10.0	12.6	2.6
ICU	4/16/2023 21:00	5	10.0	12.6	2.6
ICU	4/16/2023 22:00	5	10.0	12.6	2.6
ICU	4/16/2023 23:00	5	10.0	12.6	2.6
ICU	4/17/2023 0:00	7	10.0	12.6	2.6
ICU	4/17/2023 1:00	7	10.0	12.6	2.6
ICU	4/17/2023 2:00	7	10.0	12.6	2.6
ICU	4/17/2023 3:00	7	10.0	12.6	2.6
ICU	4/17/2023 4:00	7	10.0	12.6	2.6
ICU	4/17/2023 5:00	7	10.0	12.6	2.6
ICU	4/17/2023 6:00	7	10.0	12.6	2.6
ICU	4/17/2023 7:00	5	10.0	12.6	2.6
ICU	4/17/2023 8:00	5	10.0	14.2	4.2
ICU	4/17/2023 9:00	5	10.0	12.6	2.6
ICU	4/17/2023 10:00	5	10.0	12.6	2.6
ICU	4/17/2023 11:00	5	10.0	12.6	2.6
ICU	4/17/2023 12:00	5	10.0	12.6	2.6
ICU	4/17/2023 13:00	5	10.0	12.6	2.6
ICU	4/17/2023 14:00	6	10.0	12.6	2.6
ICU	4/17/2023 15:00	7	10.0	12.6	2.6
ICU	4/17/2023 16:00	7	10.0	12.6	2.6
ICU	4/17/2023 17:00	7	10.0	12.6	2.6
ICU	4/17/2023 18:00	7	10.0	12.6	2.6
ICU	4/17/2023 19:00	7	10.0	12.6	2.6
ICU	4/17/2023 20:00	7	10.0	8.9	-1.1
ICU	4/17/2023 21:00	7	10.0	8.9	-1.1
ICU	4/17/2023 22:00	7	10.0	8.9	-1.1
ICU	4/17/2023 23:00	7	10.0	8.9	-1.1
ICU	4/18/2023 0:00	7	10.0	8.9	-1.1
ICU	4/18/2023 1:00	8	10.0	8.9	-1.1
ICU	4/18/2023 2:00	8	10.0	8.9	-1.1
ICU	4/18/2023 3:00	8	10.0	8.9	-1.1

ICU	4/18/2023 4:00	8	10.0	8.9	-1.1
ICU	4/18/2023 5:00	8	10.0	8.9	-1.1
ICU	4/18/2023 6:00	8	10.0	8.9	-1.1
ICU	4/18/2023 7:00	8	10.0	8.9	-1.1
ICU	4/18/2023 8:00	8	11.0	8.9	-2.1
ICU	4/18/2023 9:00	8	11.0	8.9	-2.1
ICU	4/18/2023 10:00	8	11.0	8.9	-2.1
ICU	4/18/2023 11:00	8	11.0	8.9	-2.1
ICU	4/18/2023 12:00	5	11.0	10.4	-0.6
ICU	4/18/2023 13:00	5	11.0	10.4	-0.6
ICU	4/18/2023 14:00	5	11.0	10.4	-0.6
ICU	4/18/2023 15:00	5	11.0	10.4	-0.6
ICU	4/18/2023 16:00	6	10.0	11.0	1.0
ICU	4/18/2023 17:00	6	10.0	11.0	1.0
ICU	4/18/2023 18:00	6	10.0	11.0	1.0
ICU	4/18/2023 19:00	6	10.0	11.0	1.0
ICU	4/18/2023 20:00	5	10.0	11.0	1.0
ICU	4/18/2023 21:00	5	10.0	11.0	1.0
ICU	4/18/2023 22:00	5	10.0	11.0	1.0
ICU	4/18/2023 23:00	5	10.0	11.0	1.0
ICU	4/19/2023 0:00	5	10.0	11.0	1.0
ICU	4/19/2023 1:00	5	10.0	11.0	1.0
ICU	4/19/2023 2:00	5	10.0	11.0	1.0
ICU	4/19/2023 3:00	5	10.0	11.0	1.0
ICU	4/19/2023 4:00	5	10.0	11.0	1.0
ICU	4/19/2023 5:00	5	10.0	11.0	1.0
ICU	4/19/2023 6:00	5	10.0	11.0	1.0
ICU	4/19/2023 7:00	5	10.0	11.0	1.0
ICU	4/19/2023 8:00	5	10.0	13.3	3.3
ICU	4/19/2023 9:00	5	10.0	13.3	3.3
ICU	4/19/2023 10:00	5	10.0	13.3	3.3
ICU	4/19/2023 11:00	5	10.0	13.3	3.3
ICU	4/19/2023 12:00	5	10.0	13.3	3.3
ICU	4/19/2023 13:00	5	10.0	13.3	3.3
ICU	4/19/2023 14:00	5	10.0	13.3	3.3
ICU	4/19/2023 15:00	5	10.0	14.1	4.1
ICU	4/19/2023 16:00	5	10.0	14.2	4.2
ICU	4/19/2023 17:00	5	10.0	14.2	4.2
ICU	4/19/2023 18:00	5	10.0	14.2	4.2
ICU	4/19/2023 19:00	5	10.0	14.2	4.2
ICU	4/19/2023 20:00	6	8.0	14.2	6.2
ICU	4/19/2023 21:00	6	8.0	13.1	5.1
ICU	4/19/2023 22:00	6	8.0	14.2	6.2
ICU	4/19/2023 23:00	6	8.0	14.2	6.2
ICU	4/20/2023 0:00	6	8.0	14.2	6.2
ICU	4/20/2023 1:00	6	8.0	14.2	6.2
ICU	4/20/2023 2:00	6	8.0	13.1	5.1

ICU	4/20/2023 3:00	6	8.0	14.2	6.2
ICU	4/20/2023 4:00	6	8.0	14.2	6.2
ICU	4/20/2023 5:00	7	8.0	14.2	6.2
ICU	4/20/2023 6:00	7	8.0	14.2	6.2
ICU	4/20/2023 7:00	7	8.0	14.2	6.2
ICU	4/20/2023 8:00	7	10.0	11.0	1.0
ICU	4/20/2023 9:00	7	10.0	11.0	1.0
ICU	4/20/2023 10:00	7	10.0	11.0	1.0
ICU	4/20/2023 11:00	7	10.0	11.0	1.0
ICU	4/20/2023 12:00	7	10.0	12.6	2.6
ICU	4/20/2023 13:00	7	10.0	12.6	2.6
ICU	4/20/2023 14:00	7	10.0	12.6	2.6
ICU	4/20/2023 15:00	7	10.0	12.6	2.6
ICU	4/20/2023 16:00	9	10.0	11.0	1.0
ICU	4/20/2023 17:00	9	10.0	11.0	1.0
ICU	4/20/2023 18:00	9	10.0	11.0	1.0
ICU	4/20/2023 19:00	9	10.0	11.0	1.0
ICU	4/20/2023 20:00	9	10.0	11.0	1.0
ICU	4/20/2023 21:00	9	10.0	11.0	1.0
ICU	4/20/2023 22:00	10	12.0	9.9	-2.1
ICU	4/20/2023 23:00	12	12.0	10.4	-1.6
ICU	4/21/2023 0:00	12	12.0	12.6	0.6
ICU	4/21/2023 1:00	12	12.0	12.6	0.6
ICU	4/21/2023 2:00	12	12.0	12.6	0.6
ICU	4/21/2023 3:00	12	12.0	12.6	0.6
ICU	4/21/2023 4:00	12	12.0	12.6	0.6
ICU	4/21/2023 5:00	12	12.0	12.6	0.6
ICU	4/21/2023 6:00	12	12.0	12.6	0.6
ICU	4/21/2023 7:00	12	12.0	12.6	0.6
ICU	4/21/2023 8:00	12	12.0	12.6	0.6
ICU	4/21/2023 9:00	12	12.0	12.6	0.6
ICU	4/21/2023 10:00	12	12.0	12.6	0.6
ICU	4/21/2023 11:00	12	12.0	12.6	0.6
ICU	4/21/2023 12:00	12	12.0	12.6	0.6
ICU	4/21/2023 13:00	12	12.0	12.6	0.6
ICU	4/21/2023 14:00	12	12.0	12.6	0.6
ICU	4/21/2023 15:00	12	12.0	12.6	0.6
ICU	4/21/2023 16:00	11	12.0	12.6	0.6
ICU	4/21/2023 17:00	11	12.0	12.6	0.6
ICU	4/21/2023 18:00	11	12.0	12.6	0.6
ICU	4/21/2023 19:00	11	12.0	12.6	0.6
ICU	4/21/2023 20:00	12	12.0	11.0	-1.0
ICU	4/21/2023 21:00	12	12.0	11.0	-1.0
ICU	4/21/2023 22:00	12	12.0	11.0	-1.0
ICU	4/21/2023 23:00	12	12.0	11.0	-1.0
ICU	4/22/2023 0:00	12	12.0	12.6	0.6
ICU	4/22/2023 1:00	12	12.0	12.6	0.6

ICU	4/22/2023 2:00	12	12.0	12.6	0.6
ICU	4/22/2023 3:00	12	12.0	12.6	0.6
ICU	4/22/2023 4:00	12	12.0	12.6	0.6
ICU	4/22/2023 5:00	12	12.0	12.6	0.6
ICU	4/22/2023 6:00	12	12.0	12.6	0.6
ICU	4/22/2023 7:00	12	12.0	12.6	0.6
ICU	4/22/2023 8:00	12	12.0	12.6	0.6
ICU	4/22/2023 9:00	12	12.0	12.6	0.6
ICU	4/22/2023 10:00	12	12.0	12.6	0.6
ICU	4/22/2023 11:00	12	12.0	12.6	0.6
ICU	4/22/2023 12:00	12	12.0	12.6	0.6
ICU	4/22/2023 13:00	10	10.0	11.9	1.9
ICU	4/22/2023 14:00	10	10.0	11.9	1.9
ICU	4/22/2023 15:00	10	10.0	11.9	1.9
ICU	4/22/2023 16:00	10	10.0	12.6	2.6
ICU	4/22/2023 17:00	10	10.0	12.6	2.6
ICU	4/22/2023 18:00	10	10.0	12.6	2.6
ICU	4/22/2023 19:00	7	10.0	12.6	2.6
ICU	4/22/2023 20:00	7	8.0	9.5	1.5
ICU	4/22/2023 21:00	7	8.0	9.5	1.5
ICU	4/22/2023 22:00	7	8.0	9.5	1.5
ICU	4/22/2023 23:00	7	8.0	9.5	1.5
ICU	4/23/2023 0:00	7	8.0	10.3	2.3
ICU	4/23/2023 1:00	7	8.0	11.0	3.0
ICU	4/23/2023 2:00	7	8.0	11.0	3.0
ICU	4/23/2023 3:00	7	8.0	11.0	3.0
ICU	4/23/2023 4:00	7	8.0	11.0	3.0
ICU	4/23/2023 5:00	7	8.0	11.0	3.0
ICU	4/23/2023 6:00	7	8.0	11.0	3.0
ICU	4/23/2023 7:00	7	8.0	11.0	3.0
ICU	4/23/2023 8:00	7	12.0	11.0	-1.0
ICU	4/23/2023 9:00	7	12.0	11.0	-1.0
ICU	4/23/2023 10:00	7	12.0	11.0	-1.0
ICU	4/23/2023 11:00	7	12.0	11.0	-1.0
ICU	4/23/2023 12:00	6	12.0	11.0	-1.0
ICU	4/23/2023 13:00	6	12.0	11.0	-1.0
ICU	4/23/2023 14:00	6	12.0	11.0	-1.0
ICU	4/23/2023 15:00	6	12.0	11.0	-1.0
ICU	4/23/2023 16:00	6	12.0	10.4	-1.6
ICU	4/23/2023 17:00	7	12.0	10.4	-1.6
ICU	4/23/2023 18:00	7	12.0	10.4	-1.6
ICU	4/23/2023 19:00	7	12.0	10.4	-1.6
ICU	4/23/2023 20:00	7	12.0	10.4	-1.6
ICU	4/23/2023 21:00	7	12.0	10.4	-1.6
ICU	4/23/2023 22:00	7	12.0	10.4	-1.6
ICU	4/23/2023 23:00	7	12.0	10.4	-1.6
ICU	4/24/2023 0:00	7	11.0	8.9	-2.1

ICU	4/24/2023 1:00	7	11.0	8.9	-2.1
ICU	4/24/2023 2:00	7	11.0	8.9	-2.1
ICU	4/24/2023 3:00	7	11.0	8.9	-2.1
ICU	4/24/2023 4:00	7	11.0	8.9	-2.1
ICU	4/24/2023 5:00	7	11.0	8.9	-2.1
ICU	4/24/2023 6:00	7	11.0	8.9	-2.1
ICU	4/24/2023 7:00	7	11.0	8.9	-2.1
ICU	4/24/2023 8:00	7	14.0	8.9	-5.1
ICU	4/24/2023 9:00	7	14.0	8.9	-5.1
ICU	4/24/2023 10:00	8	14.0	8.9	-5.1
ICU	4/24/2023 11:00	8	14.0	7.8	-6.2
ICU	4/24/2023 12:00	8	14.0	8.9	-5.1
ICU	4/24/2023 13:00	8	14.0	8.9	-5.1
ICU	4/24/2023 14:00	8	14.0	8.9	-5.1
ICU	4/24/2023 15:00	8	14.0	8.2	-5.9
ICU	4/24/2023 16:00	9	12.0	7.9	-4.1
ICU	4/24/2023 17:00	9	12.0	7.9	-4.1
ICU	4/24/2023 18:00	9	12.0	7.9	-4.1
ICU	4/24/2023 19:00	9	12.0	7.9	-4.1
ICU	4/24/2023 20:00	9	12.0	11.0	-1.0
ICU	4/24/2023 21:00	9	12.0	11.0	-1.0
ICU	4/24/2023 22:00	9	12.0	11.0	-1.0
ICU	4/24/2023 23:00	9	12.0	11.0	-1.0
ICU	4/25/2023 0:00	9	12.0	8.9	-3.1
ICU	4/25/2023 1:00	9	10.0	8.9	-1.1
ICU	4/25/2023 2:00	9	10.0	8.9	-1.1
ICU	4/25/2023 3:00	9	10.0	8.9	-1.1
ICU	4/25/2023 4:00	9	10.0	8.9	-1.1
ICU	4/25/2023 5:00	9	10.0	8.9	-1.1
ICU	4/25/2023 6:00	9	10.0	8.9	-1.1
ICU	4/25/2023 7:00	9	10.0	8.9	-1.1
ICU	4/25/2023 8:00	9	10.0	10.4	0.4
ICU	4/25/2023 9:00	9	10.0	10.4	0.4
ICU	4/25/2023 10:00	9	10.0	10.4	0.4
ICU	4/25/2023 11:00	9	10.0	10.4	0.4
ICU	4/25/2023 12:00	9	11.0	10.4	-0.6
ICU	4/25/2023 13:00	9	11.0	10.4	-0.6
ICU	4/25/2023 14:00	9	11.0	10.4	-0.6
ICU	4/25/2023 15:00	9	11.0	10.4	-0.6
ICU	4/25/2023 16:00	9	12.0	11.0	-1.0
ICU	4/25/2023 17:00	9	12.0	11.0	-1.0
ICU	4/25/2023 18:00	9	12.0	11.0	-1.0
ICU	4/25/2023 19:00	9	12.0	11.0	-1.0
ICU	4/25/2023 20:00	11	12.0	9.5	-2.5
ICU	4/25/2023 21:00	11	12.0	8.4	-3.7
ICU	4/25/2023 22:00	11	12.0	9.5	-2.5
ICU	4/25/2023 23:00	11	12.0	9.5	-2.5

ICU	4/26/2023 0:00	11	12.0	9.5	-2.5
ICU	4/26/2023 1:00	11	12.0	9.5	-2.5
ICU	4/26/2023 2:00	11	12.0	9.5	-2.5
ICU	4/26/2023 3:00	11	12.0	9.5	-2.5
ICU	4/26/2023 4:00	11	12.0	9.5	-2.5
ICU	4/26/2023 5:00	11	12.0	9.5	-2.5
ICU	4/26/2023 6:00	11	12.0	9.5	-2.5
ICU	4/26/2023 7:00	11	12.0	9.5	-2.5
ICU	4/26/2023 8:00	11	12.0	11.9	-0.2
ICU	4/26/2023 9:00	11	12.0	11.9	-0.2
ICU	4/26/2023 10:00	11	12.0	11.9	-0.2
ICU	4/26/2023 11:00	11	12.0	11.9	-0.2
ICU	4/26/2023 12:00	10	12.0	11.9	-0.2
ICU	4/26/2023 13:00	10	12.0	11.9	-0.2
ICU	4/26/2023 14:00	10	12.0	11.9	-0.2
ICU	4/26/2023 15:00	10	12.0	11.9	-0.2
ICU	4/26/2023 16:00	9	12.0	11.0	-1.0
ICU	4/26/2023 17:00	9	12.0	11.0	-1.0
ICU	4/26/2023 18:00	9	12.0	11.0	-1.0
ICU	4/26/2023 19:00	9	12.0	11.0	-1.0
ICU	4/26/2023 20:00	9	12.0	9.5	-2.5
ICU	4/26/2023 21:00	9	9.0	9.5	0.5
ICU	4/26/2023 22:00	9	9.0	9.5	0.5
ICU	4/26/2023 23:00	9	9.0	9.5	0.5
ICU	4/27/2023 0:00	9	9.0	8.9	-0.1
ICU	4/27/2023 1:00	9	9.0	8.9	-0.1
ICU	4/27/2023 2:00	9	9.0	8.9	-0.1
ICU	4/27/2023 3:00	9	9.0	8.9	-0.1
ICU	4/27/2023 4:00	9	9.0	8.9	-0.1
ICU	4/27/2023 5:00	9	9.0	8.9	-0.1
ICU	4/27/2023 6:00	9	9.0	8.9	-0.1
ICU	4/27/2023 7:00	9	9.0	8.9	-0.1
ICU	4/27/2023 8:00	9	9.0	11.9	2.9
ICU	4/27/2023 9:00	9	9.0	11.9	2.9
ICU	4/27/2023 10:00	9	9.0	11.9	2.9
ICU	4/27/2023 11:00	10	12.0	12.6	0.6
ICU	4/27/2023 12:00	10	12.0	12.6	0.6
ICU	4/27/2023 13:00	10	12.0	12.6	0.6
ICU	4/27/2023 14:00	10	12.0	12.6	0.6
ICU	4/27/2023 15:00	10	12.0	12.6	0.6
ICU	4/27/2023 16:00	6	9.0	9.5	0.5
ICU	4/27/2023 17:00	6	9.0	9.5	0.5
ICU	4/27/2023 18:00	6	9.0	9.5	0.5
ICU	4/27/2023 19:00	6	9.0	9.5	0.5
ICU	4/27/2023 20:00	6	9.0	10.4	1.4
ICU	4/27/2023 21:00	6	9.0	9.3	0.3
ICU	4/27/2023 22:00	6	9.0	10.4	1.4

ICU	4/27/2023 23:00	6	9.0	10.4	1.4
ICU	4/28/2023 0:00	6	9.0	10.4	1.4
ICU	4/28/2023 1:00	6	9.0	10.4	1.4
ICU	4/28/2023 2:00	6	9.0	10.4	1.4
ICU	4/28/2023 3:00	6	9.0	10.4	1.4
ICU	4/28/2023 4:00	6	9.0	10.4	1.4
ICU	4/28/2023 5:00	6	9.0	10.4	1.4
ICU	4/28/2023 6:00	6	9.0	10.4	1.4
ICU	4/28/2023 7:00	6	9.0	10.4	1.4
ICU	4/28/2023 8:00	6	9.0	7.4	-1.6
ICU	4/28/2023 9:00	8	10.0	7.4	-2.6
ICU	4/28/2023 10:00	8	10.0	7.4	-2.6
ICU	4/28/2023 11:00	7	10.0	7.4	-2.6
ICU	4/28/2023 12:00	7	10.0	7.4	-2.6
ICU	4/28/2023 13:00	7	10.0	7.4	-2.6
ICU	4/28/2023 14:00	9	10.0	7.4	-2.6
ICU	4/28/2023 15:00	9	10.0	7.4	-2.6
ICU	4/28/2023 16:00	9	10.0	8.9	-1.1
ICU	4/28/2023 17:00	7	10.0	8.9	-1.1
ICU	4/28/2023 18:00	7	10.0	8.9	-1.1
ICU	4/28/2023 19:00	7	10.0	8.9	-1.1
ICU	4/28/2023 20:00	7	10.0	11.9	1.9
ICU	4/28/2023 21:00	7	10.0	11.9	1.9
ICU	4/28/2023 22:00	7	10.0	11.9	1.9
ICU	4/28/2023 23:00	7	10.0	11.9	1.9
ICU	4/29/2023 0:00	7	10.0	10.4	0.4
ICU	4/29/2023 1:00	12	8.0	11.5	3.5
ICU	4/29/2023 2:00	12	8.0	12.6	4.6
ICU	4/29/2023 3:00	12	8.0	12.6	4.6
ICU	4/29/2023 4:00	12	8.0	12.6	4.6
ICU	4/29/2023 5:00	12	8.0	12.6	4.6
ICU	4/29/2023 6:00	12	8.0	12.6	4.6
ICU	4/29/2023 7:00	12	8.0	12.6	4.6
ICU	4/29/2023 8:00	10	8.0	11.0	3.0
ICU	4/29/2023 9:00	10	8.0	11.0	3.0
ICU	4/29/2023 10:00	10	8.0	11.0	3.0
ICU	4/29/2023 11:00	8	10.0	11.8	1.8
ICU	4/29/2023 12:00	8	10.0	12.6	2.6
ICU	4/29/2023 13:00	8	10.0	12.6	2.6
ICU	4/29/2023 14:00	8	10.0	12.6	2.6
ICU	4/29/2023 15:00	8	10.0	12.6	2.6
ICU	4/29/2023 16:00	8	10.0	9.5	-0.5
ICU	4/29/2023 17:00	8	10.0	9.5	-0.5
ICU	4/29/2023 18:00	8	10.0	9.5	-0.5
ICU	4/29/2023 19:00	8	10.0	9.5	-0.5
ICU	4/29/2023 20:00	7	10.0	7.4	-2.6
ICU	4/29/2023 21:00	7	10.0	7.4	-2.6

ICU	4/29/2023 22:00	7	10.0	7.4	-2.6
ICU	4/29/2023 23:00	7	10.0	7.4	-2.6
ICU	4/30/2023 0:00	8	10.0	7.4	-2.6
ICU	4/30/2023 1:00	8	10.0	7.4	-2.6
ICU	4/30/2023 2:00	8	10.0	7.4	-2.6
ICU	4/30/2023 3:00	8	10.0	7.4	-2.6
ICU	4/30/2023 4:00	8	10.0	7.4	-2.6
ICU	4/30/2023 5:00	8	10.0	7.4	-2.6
ICU	4/30/2023 6:00	8	10.0	7.4	-2.6
ICU	4/30/2023 7:00	8	10.0	7.4	-2.6
ICU	4/30/2023 8:00	8	10.0	9.5	-0.5
ICU	4/30/2023 9:00	8	10.0	9.5	-0.5
ICU	4/30/2023 10:00	8	10.0	9.5	-0.5
ICU	4/30/2023 11:00	8	10.0	9.5	-0.5
ICU	4/30/2023 12:00	8	10.0	11.0	1.0
ICU	4/30/2023 13:00	8	10.0	11.0	1.0
ICU	4/30/2023 14:00	8	10.0	11.0	1.0
ICU	4/30/2023 15:00	8	10.0	11.0	1.0
ICU	4/30/2023 16:00	8	10.0	9.5	-0.5
ICU	4/30/2023 17:00	8	10.0	9.5	-0.5
ICU	4/30/2023 18:00	8	10.0	9.5	-0.5
ICU	4/30/2023 19:00	8	13.0	9.5	-3.5
ICU	4/30/2023 20:00	8	13.0	8.9	-4.1
ICU	4/30/2023 21:00	8	13.0	8.9	-4.1
ICU	4/30/2023 22:00	8	13.0	8.9	-4.1
ICU	4/30/2023 23:00	8	13.0	8.9	-4.1
ICU	5/1/2023 0:00	8	13.0	8.9	-4.1
ICU	5/1/2023 1:00	8	13.0	8.9	-4.1
ICU	5/1/2023 2:00	8	13.0	8.9	-4.1
ICU	5/1/2023 3:00	8	13.0	8.9	-4.1
ICU	5/1/2023 4:00	8	13.0	8.9	-4.1
ICU	5/1/2023 5:00	8	13.0	8.9	-4.1
ICU	5/1/2023 6:00	8	13.0	8.9	-4.1
ICU	5/1/2023 7:00	8	13.0	8.9	-4.1
ICU	5/1/2023 8:00	8	12.0	11.0	-1.0
ICU	5/1/2023 9:00	8	12.0	11.0	-1.0
ICU	5/1/2023 10:00	8	12.0	11.0	-1.0
ICU	5/1/2023 11:00	9	12.0	9.9	-2.1
ICU	5/1/2023 12:00	9	12.0	11.0	-1.0
ICU	5/1/2023 13:00	9	12.0	11.0	-1.0
ICU	5/1/2023 14:00	7	12.0	9.9	-2.1
ICU	5/1/2023 15:00	7	12.0	11.0	-1.0
ICU	5/1/2023 16:00	7	12.0	11.0	-1.0
ICU	5/1/2023 17:00	8	12.0	11.0	-1.0
ICU	5/1/2023 18:00	8	12.0	11.0	-1.0
ICU	5/1/2023 19:00	8	12.0	11.0	-1.0
ICU	5/1/2023 20:00	8	14.0	10.4	-3.6

ICU	5/1/2023 21:00	8	14.0	10.4	-3.6
ICU	5/1/2023 22:00	8	14.0	10.4	-3.6
ICU	5/1/2023 23:00	9	14.0	10.4	-3.6
ICU	5/2/2023 0:00	9	14.0	8.9	-5.1
ICU	5/2/2023 1:00	9	14.0	8.9	-5.1
ICU	5/2/2023 2:00	9	14.0	8.9	-5.1
ICU	5/2/2023 3:00	10	14.0	8.9	-5.1
ICU	5/2/2023 4:00	10	14.0	8.9	-5.1
ICU	5/2/2023 5:00	10	14.0	8.9	-5.1
ICU	5/2/2023 6:00	10	14.0	8.9	-5.1
ICU	5/2/2023 7:00	10	14.0	8.9	-5.1
ICU	5/2/2023 8:00	9	13.0	11.9	-1.2
ICU	5/2/2023 9:00	9	13.0	11.9	-1.2
ICU	5/2/2023 10:00	9	13.0	11.9	-1.2
ICU	5/2/2023 11:00	9	13.0	11.9	-1.2
ICU	5/2/2023 12:00	9	13.0	13.3	0.3
ICU	5/2/2023 13:00	13	13.0	13.3	0.3
ICU	5/2/2023 14:00	11	12.0	13.3	1.3
ICU	5/2/2023 15:00	11	12.0	13.3	1.3
ICU	5/2/2023 16:00	11	12.0	11.0	-1.0
ICU	5/2/2023 17:00	11	12.0	11.0	-1.0
ICU	5/2/2023 18:00	11	12.0	11.0	-1.0
ICU	5/2/2023 19:00	11	12.0	11.0	-1.0
ICU	5/2/2023 20:00	11	12.0	11.9	-0.2
ICU	5/2/2023 21:00	12	13.0	14.2	1.2
ICU	5/2/2023 22:00	12	13.0	14.2	1.2
ICU	5/2/2023 23:00	12	13.0	14.2	1.2
ICU	5/3/2023 0:00	12	13.0	13.3	0.3
ICU	5/3/2023 1:00	12	13.0	13.3	0.3
ICU	5/3/2023 2:00	12	13.0	13.3	0.3
ICU	5/3/2023 3:00	12	13.0	13.3	0.3
ICU	5/3/2023 4:00	12	13.0	13.3	0.3
ICU	5/3/2023 5:00	12	13.0	13.3	0.3
ICU	5/3/2023 6:00	12	13.0	13.3	0.3
ICU	5/3/2023 7:00	12	13.0	13.3	0.3
ICU	5/3/2023 8:00	12	13.0	14.2	1.2
ICU	5/3/2023 9:00	12	13.0	14.2	1.2
ICU	5/3/2023 10:00	12	13.0	14.2	1.2
ICU	5/3/2023 11:00	12	13.0	14.2	1.2
ICU	5/3/2023 12:00	12	13.0	14.2	1.2
ICU	5/3/2023 13:00	12	13.0	14.2	1.2
ICU	5/3/2023 14:00	11	12.0	14.2	2.2
ICU	5/3/2023 15:00	11	12.0	14.2	2.2
ICU	5/3/2023 16:00	11	12.0	12.6	0.6
ICU	5/3/2023 17:00	11	12.0	12.6	0.6
ICU	5/3/2023 18:00	11	12.0	12.6	0.6
ICU	5/3/2023 19:00	11	12.0	12.6	0.6

ICU	5/3/2023 20:00	12	12.0	11.0	-1.0
ICU	5/3/2023 21:00	12	12.0	12.6	0.6
ICU	5/3/2023 22:00	12	12.0	12.6	0.6
ICU	5/3/2023 23:00	12	12.0	12.6	0.6
ICU	5/4/2023 0:00	12	12.0	12.6	0.6
ICU	5/4/2023 1:00	12	12.0	12.6	0.6
ICU	5/4/2023 2:00	12	12.0	12.6	0.6
ICU	5/4/2023 3:00	12	12.0	12.6	0.6
ICU	5/4/2023 4:00	12	12.0	12.6	0.6
ICU	5/4/2023 5:00	12	12.0	12.6	0.6
ICU	5/4/2023 6:00	12	12.0	12.6	0.6
ICU	5/4/2023 7:00	12	12.0	12.6	0.6
ICU	5/4/2023 8:00	12	12.0	14.2	2.2
ICU	5/4/2023 9:00	12	12.0	14.2	2.2
ICU	5/4/2023 10:00	12	12.0	14.2	2.2
ICU	5/4/2023 11:00	12	12.0	14.2	2.2
ICU	5/4/2023 12:00	12	12.0	14.2	2.2
ICU	5/4/2023 13:00	12	12.0	14.2	2.2
ICU	5/4/2023 14:00	12	12.0	14.2	2.2
ICU	5/4/2023 15:00	12	12.0	14.2	2.2
ICU	5/4/2023 16:00	11	13.0	12.6	-0.4
ICU	5/4/2023 17:00	11	13.0	12.6	-0.4
ICU	5/4/2023 18:00	10	13.0	12.6	-0.4
ICU	5/4/2023 19:00	10	13.0	12.6	-0.4
ICU	5/4/2023 20:00	10	13.0	12.6	-0.4
ICU	5/4/2023 21:00	11	13.0	12.6	-0.4
ICU	5/4/2023 22:00	11	13.0	12.6	-0.4
ICU	5/4/2023 23:00	11	13.0	12.6	-0.4
ICU	5/5/2023 0:00	11	13.0	12.6	-0.4
ICU	5/5/2023 1:00	11	13.0	12.6	-0.4
ICU	5/5/2023 2:00	11	13.0	12.6	-0.4
ICU	5/5/2023 3:00	11	13.0	12.6	-0.4
ICU	5/5/2023 4:00	11	13.0	12.6	-0.4
ICU	5/5/2023 5:00	11	13.0	12.6	-0.4
ICU	5/5/2023 6:00	11	13.0	12.6	-0.4
ICU	5/5/2023 7:00	11	13.0	12.6	-0.4
ICU	5/5/2023 8:00	12	12.0	12.6	0.6
ICU	5/5/2023 9:00	12	12.0	12.6	0.6
ICU	5/5/2023 10:00	12	12.0	12.6	0.6
ICU	5/5/2023 11:00	12	12.0	12.6	0.6
ICU	5/5/2023 12:00	12	12.0	14.2	2.2
ICU	5/5/2023 13:00	12	12.0	14.2	2.2
ICU	5/5/2023 14:00	12	12.0	14.2	2.2
ICU	5/5/2023 15:00	12	12.0	14.2	2.2
ICU	5/5/2023 16:00	12	12.0	14.2	2.2
ICU	5/5/2023 17:00	12	12.0	14.2	2.2
ICU	5/5/2023 18:00	12	12.0	14.2	2.2

ICU	5/5/2023 19:00	12	12.0	14.2	2.2
ICU	5/5/2023 20:00	11	12.0	11.0	-1.0
ICU	5/5/2023 21:00	11	12.0	11.0	-1.0
ICU	5/5/2023 22:00	11	12.0	11.0	-1.0
ICU	5/5/2023 23:00	11	12.0	11.0	-1.0
ICU	5/6/2023 0:00	11	12.0	12.6	0.6
ICU	5/6/2023 1:00	11	12.0	12.6	0.6
ICU	5/6/2023 2:00	11	12.0	12.6	0.6
ICU	5/6/2023 3:00	11	12.0	12.6	0.6
ICU	5/6/2023 4:00	12	12.0	12.6	0.6
ICU	5/6/2023 5:00	12	12.0	12.6	0.6
ICU	5/6/2023 6:00	12	12.0	12.6	0.6
ICU	5/6/2023 7:00	12	12.0	12.6	0.6
ICU	5/6/2023 8:00	12	12.0	11.9	-0.2
ICU	5/6/2023 9:00	12	12.0	11.9	-0.2
ICU	5/6/2023 10:00	12	12.0	11.9	-0.2
ICU	5/6/2023 11:00	11	12.0	11.9	-0.2
ICU	5/6/2023 12:00	11	12.0	11.9	-0.2
ICU	5/6/2023 13:00	11	12.0	11.9	-0.2
ICU	5/6/2023 14:00	11	12.0	11.9	-0.2
ICU	5/6/2023 15:00	11	12.0	11.9	-0.2
ICU	5/6/2023 16:00	11	12.0	12.6	0.6
ICU	5/6/2023 17:00	11	12.0	12.6	0.6
ICU	5/6/2023 18:00	11	12.0	12.6	0.6
ICU	5/6/2023 19:00	9	10.0	12.6	2.6
ICU	5/6/2023 20:00	9	10.0	7.9	-2.1
ICU	5/6/2023 21:00	9	10.0	7.9	-2.1
ICU	5/6/2023 22:00	9	10.0	7.9	-2.1
ICU	5/6/2023 23:00	9	10.0	7.9	-2.1
ICU	5/7/2023 0:00	9	10.0	7.9	-2.1
ICU	5/7/2023 1:00	9	10.0	7.9	-2.1
ICU	5/7/2023 2:00	9	10.0	7.9	-2.1
ICU	5/7/2023 3:00	9	10.0	7.9	-2.1
ICU	5/7/2023 4:00	9	10.0	7.9	-2.1
ICU	5/7/2023 5:00	9	10.0	7.9	-2.1
ICU	5/7/2023 6:00	9	10.0	7.9	-2.1
ICU	5/7/2023 7:00	9	10.0	7.9	-2.1
ICU	5/7/2023 8:00	9	11.0	11.0	0.0
ICU	5/7/2023 9:00	9	11.0	11.0	0.0
ICU	5/7/2023 10:00	9	11.0	11.0	0.0
ICU	5/7/2023 11:00	9	11.0	11.0	0.0
ICU	5/7/2023 12:00	9	11.0	11.0	0.0
ICU	5/7/2023 13:00	9	11.0	11.0	0.0
ICU	5/7/2023 14:00	9	11.0	11.0	0.0
ICU	5/7/2023 15:00	9	11.0	11.0	0.0
ICU	5/7/2023 16:00	9	11.0	11.0	0.0
ICU	5/7/2023 17:00	9	11.0	11.0	0.0

ICU	5/7/2023 18:00	9	11.0	11.0	0.0
ICU	5/7/2023 19:00	9	11.0	11.0	0.0
ICU	5/7/2023 20:00	10	11.0	9.5	-1.5
ICU	5/7/2023 21:00	10	11.0	9.5	-1.5
ICU	5/7/2023 22:00	11	12.0	11.8	-0.2
ICU	5/7/2023 23:00	11	12.0	12.6	0.6
ICU	5/8/2023 0:00	11	11.0	10.4	-0.6
ICU	5/8/2023 1:00	11	11.0	10.4	-0.6
ICU	5/8/2023 2:00	11	11.0	10.4	-0.6
ICU	5/8/2023 3:00	11	11.0	10.4	-0.6
ICU	5/8/2023 4:00	11	11.0	10.4	-0.6
ICU	5/8/2023 5:00	11	11.0	10.4	-0.6
ICU	5/8/2023 6:00	11	11.0	10.4	-0.6
ICU	5/8/2023 7:00	11	11.0	10.4	-0.6
ICU	5/8/2023 8:00	11	11.0	12.6	1.6
ICU	5/8/2023 9:00	11	11.0	12.6	1.6
ICU	5/8/2023 10:00	11	11.0	12.6	1.6
ICU	5/8/2023 11:00	12	12.0	12.6	0.6
ICU	5/8/2023 12:00	13	13.0	12.6	-0.4
ICU	5/8/2023 13:00	12	12.0	12.6	0.6
ICU	5/8/2023 14:00	12	12.0	12.6	0.6
ICU	5/8/2023 15:00	12	12.0	12.6	0.6
ICU	5/8/2023 16:00	12	12.0	11.0	-1.0
ICU	5/8/2023 17:00	12	12.0	11.0	-1.0
ICU	5/8/2023 18:00	12	12.0	11.0	-1.0
ICU	5/8/2023 19:00	12	12.0	11.0	-1.0
ICU	5/8/2023 20:00	12	12.0	11.0	-1.0
ICU	5/8/2023 21:00	12	12.0	11.0	-1.0
ICU	5/8/2023 22:00	12	12.0	11.0	-1.0
ICU	5/8/2023 23:00	12	12.0	11.0	-1.0
ICU	5/9/2023 0:00	12	12.0	10.4	-1.6
ICU	5/9/2023 1:00	12	12.0	10.4	-1.6
ICU	5/9/2023 2:00	12	12.0	10.4	-1.6
ICU	5/9/2023 3:00	12	12.0	10.4	-1.6
ICU	5/9/2023 4:00	12	12.0	10.4	-1.6
ICU	5/9/2023 5:00	12	12.0	10.4	-1.6
ICU	5/9/2023 6:00	12	12.0	10.4	-1.6
ICU	5/9/2023 7:00	12	12.0	10.4	-1.6
ICU	5/9/2023 8:00	12	14.0	14.2	0.2
ICU	5/9/2023 9:00	12	14.0	12.0	-2.0
ICU	5/9/2023 10:00	12	14.0	14.2	0.2
ICU	5/9/2023 11:00	13	14.0	2.0	-12.0
ICU	5/9/2023 12:00	13	14.0	14.2	0.2
ICU	5/9/2023 13:00	12	14.0	13.1	-0.9
ICU	5/9/2023 14:00	12	14.0	14.2	0.2
ICU	5/9/2023 15:00	11	14.0	13.1	-0.9
ICU	5/9/2023 16:00	11	14.0	14.2	0.2

ICU	5/9/2023 17:00	11	14.0	14.2	0.2
ICU	5/9/2023 18:00	11	14.0	14.2	0.2
ICU	5/9/2023 19:00	13	14.0	14.2	0.2
ICU	5/9/2023 20:00	13	14.0	11.0	-3.0
ICU	5/9/2023 21:00	13	14.0	11.0	-3.0
ICU	5/9/2023 22:00	13	14.0	11.0	-3.0
ICU	5/9/2023 23:00	13	14.0	11.0	-3.0
ICU	5/10/2023 0:00	13	14.0	11.9	-2.2
ICU	5/10/2023 1:00	13	14.0	11.9	-2.2
ICU	5/10/2023 2:00	13	14.0	11.9	-2.2
ICU	5/10/2023 3:00	13	14.0	11.9	-2.2
ICU	5/10/2023 4:00	13	14.0	11.9	-2.2
ICU	5/10/2023 5:00	13	14.0	11.9	-2.2
ICU	5/10/2023 6:00	13	14.0	11.9	-2.2
ICU	5/10/2023 7:00	13	14.0	11.9	-2.2
ICU	5/10/2023 8:00	13	14.0	12.6	-1.4
ICU	5/10/2023 9:00	13	14.0	12.6	-1.4
ICU	5/10/2023 10:00	13	14.0	12.6	-1.4
ICU	5/10/2023 11:00	13	14.0	12.6	-1.4
ICU	5/10/2023 12:00	13	14.0	12.6	-1.4
ICU	5/10/2023 13:00	14	14.0	12.6	-1.4
ICU	5/10/2023 14:00	15	14.0	12.6	-1.4
ICU	5/10/2023 15:00	15	14.0	12.6	-1.4
ICU	5/10/2023 16:00	14	14.0	11.0	-3.0
ICU	5/10/2023 17:00	13	14.0	9.9	-4.1
ICU	5/10/2023 18:00	13	14.0	11.0	-3.0
ICU	5/10/2023 19:00	13	14.0	8.8	-5.2
ICU	5/10/2023 20:00	13	14.0	11.0	-3.0
ICU	5/10/2023 21:00	14	14.0	11.0	-3.0
ICU	5/10/2023 22:00	14	14.0	11.0	-3.0
ICU	5/10/2023 23:00	14	14.0	11.0	-3.0
ICU	5/11/2023 0:00	14	14.0	11.0	-3.0
ICU	5/11/2023 1:00	14	14.0	11.0	-3.0
ICU	5/11/2023 2:00	14	14.0	11.0	-3.0
ICU	5/11/2023 3:00	14	14.0	11.0	-3.0
ICU	5/11/2023 4:00	14	14.0	11.0	-3.0
ICU	5/11/2023 5:00	14	14.0	11.0	-3.0
ICU	5/11/2023 6:00	14	14.0	11.0	-3.0
ICU	5/11/2023 7:00	14	14.0	11.0	-3.0
ICU	5/11/2023 8:00	14	14.0	11.0	-3.0
ICU	5/11/2023 9:00	14	14.0	11.0	-3.0
ICU	5/11/2023 10:00	14	14.0	11.0	-3.0
ICU	5/11/2023 11:00	14	14.0	11.0	-3.0
ICU	5/11/2023 12:00	12	14.0	8.8	-5.2
ICU	5/11/2023 13:00	11	14.0	11.0	-3.0
ICU	5/11/2023 14:00	11	14.0	11.0	-3.0
ICU	5/11/2023 15:00	12	13.0	11.0	-2.0

ICU	5/11/2023 16:00	13	13.0	9.9	-3.1
ICU	5/11/2023 17:00	12	13.0	9.9	-3.1
ICU	5/11/2023 18:00	11	13.0	9.9	-3.1
ICU	5/11/2023 19:00	11	13.0	11.0	-2.0
ICU	5/11/2023 20:00	11	13.0	12.6	-0.4
ICU	5/11/2023 21:00	11	13.0	12.6	-0.4
ICU	5/11/2023 22:00	12	13.0	10.4	-2.6
ICU	5/11/2023 23:00	12	13.0	12.6	-0.4
ICU	5/12/2023 0:00	12	13.0	11.5	-1.5
ICU	5/12/2023 1:00	12	13.0	12.6	-0.4
ICU	5/12/2023 2:00	12	13.0	12.6	-0.4
ICU	5/12/2023 3:00	12	13.0	12.6	-0.4
ICU	5/12/2023 4:00	12	13.0	12.6	-0.4
ICU	5/12/2023 5:00	12	13.0	12.6	-0.4
ICU	5/12/2023 6:00	12	13.0	12.6	-0.4
ICU	5/12/2023 7:00	12	13.0	12.6	-0.4
ICU	5/12/2023 8:00	12	13.0	12.6	-0.4
ICU	5/12/2023 9:00	12	13.0	12.6	-0.4
ICU	5/12/2023 10:00	12	13.0	12.6	-0.4
ICU	5/12/2023 11:00	12	13.0	12.6	-0.4
ICU	5/12/2023 12:00	12	13.0	12.6	-0.4
ICU	5/12/2023 13:00	12	13.0	12.6	-0.4
ICU	5/12/2023 14:00	12	13.0	12.6	-0.4
ICU	5/12/2023 15:00	12	13.0	12.6	-0.4
ICU	5/12/2023 16:00	12	13.0	11.0	-2.0
ICU	5/12/2023 17:00	12	13.0	11.0	-2.0
ICU	5/12/2023 18:00	12	13.0	11.0	-2.0
ICU	5/12/2023 19:00	12	13.0	11.0	-2.0
ICU	5/12/2023 20:00	14	13.0	13.1	0.1
ICU	5/12/2023 21:00	14	13.0	14.2	1.2
ICU	5/12/2023 22:00	14	14.0	14.2	0.2
ICU	5/12/2023 23:00	14	14.0	14.2	0.2
ICU	5/13/2023 0:00	14	14.0	13.3	-0.7
ICU	5/13/2023 1:00	14	14.0	13.3	-0.7
ICU	5/13/2023 2:00	14	14.0	13.3	-0.7
ICU	5/13/2023 3:00	14	14.0	13.3	-0.7
ICU	5/13/2023 4:00	14	14.0	13.3	-0.7
ICU	5/13/2023 5:00	14	14.0	13.3	-0.7
ICU	5/13/2023 6:00	14	14.0	13.3	-0.7
ICU	5/13/2023 7:00	14	14.0	13.3	-0.7
ICU	5/13/2023 8:00	14	14.0	14.8	0.8
ICU	5/13/2023 9:00	14	14.0	14.8	0.8
ICU	5/13/2023 10:00	14	14.0	14.8	0.8
ICU	5/13/2023 11:00	14	14.0	14.8	0.8
ICU	5/13/2023 12:00	14	14.0	14.8	0.8
ICU	5/13/2023 13:00	13	14.0	14.8	0.8
ICU	5/13/2023 14:00	13	14.0	14.8	0.8

ICU	5/13/2023 15:00	13	14.0	14.8	0.8
ICU	5/13/2023 16:00	13	14.0	12.6	-1.4
ICU	5/13/2023 17:00	13	14.0	12.6	-1.4
ICU	5/13/2023 18:00	13	14.0	12.6	-1.4
ICU	5/13/2023 19:00	13	14.0	12.6	-1.4
ICU	5/13/2023 20:00	13	14.0	12.6	-1.4
ICU	5/13/2023 21:00	13	14.0	12.6	-1.4
ICU	5/13/2023 22:00	13	14.0	12.6	-1.4
ICU	5/13/2023 23:00	13	14.0	12.6	-1.4
ICU	5/14/2023 0:00	13	14.0	11.9	-2.2
ICU	5/14/2023 1:00	13	14.0	11.9	-2.2
ICU	5/14/2023 2:00	13	14.0	11.9	-2.2
ICU	5/14/2023 3:00	13	14.0	11.9	-2.2
ICU	5/14/2023 4:00	13	14.0	11.9	-2.2
ICU	5/14/2023 5:00	13	14.0	11.9	-2.2
ICU	5/14/2023 6:00	13	14.0	11.9	-2.2
ICU	5/14/2023 7:00	13	14.0	11.9	-2.2
ICU	5/14/2023 8:00	13	14.0	15.0	1.0
ICU	5/14/2023 9:00	13	14.0	15.0	1.0
ICU	5/14/2023 10:00	13	14.0	15.0	1.0
ICU	5/14/2023 11:00	13	14.0	15.0	1.0
ICU	5/14/2023 12:00	13	14.0	15.0	1.0
ICU	5/14/2023 13:00	13	14.0	15.0	1.0
ICU	5/14/2023 14:00	13	14.0	15.0	1.0
ICU	5/14/2023 15:00	13	14.0	15.0	1.0
ICU	5/14/2023 16:00	13	14.0	12.6	-1.4
ICU	5/14/2023 17:00	14	13.0	12.6	-0.4
ICU	5/14/2023 18:00	11	14.0	12.6	-1.4
ICU	5/14/2023 19:00	12	14.0	12.6	-1.4
ICU	5/14/2023 20:00	11	12.0	11.0	-1.0
ICU	5/14/2023 21:00	12	12.0	11.0	-1.0
ICU	5/14/2023 22:00	12	12.0	11.0	-1.0
ICU	5/14/2023 23:00	12	12.0	11.0	-1.0
ICU	5/15/2023 0:00	12	12.0	10.4	-1.6
ICU	5/15/2023 1:00	12	12.0	10.4	-1.6
ICU	5/15/2023 2:00	12	12.0	10.4	-1.6
ICU	5/15/2023 3:00	12	12.0	10.4	-1.6
ICU	5/15/2023 4:00	12	12.0	10.4	-1.6
ICU	5/15/2023 5:00	12	12.0	10.4	-1.6
ICU	5/15/2023 6:00	12	12.0	10.4	-1.6
ICU	5/15/2023 7:00	12	12.0	10.4	-1.6
ICU	5/15/2023 8:00	13	13.0	14.2	1.2
ICU	5/15/2023 9:00	13	13.0	14.2	1.2
ICU	5/15/2023 10:00	13	13.0	14.2	1.2
ICU	5/15/2023 11:00	13	13.0	14.2	1.2
ICU	5/15/2023 12:00	13	13.0	14.2	1.2
ICU	5/15/2023 13:00	13	13.0	14.2	1.2

ICU	5/15/2023 14:00	13	13.0	14.2	1.2
ICU	5/15/2023 15:00	13	13.0	14.2	1.2
ICU	5/15/2023 16:00	13	13.0	12.6	-0.4
ICU	5/15/2023 17:00	13	13.0	12.6	-0.4
ICU	5/15/2023 18:00	13	13.0	12.6	-0.4
ICU	5/15/2023 19:00	13	13.0	12.6	-0.4
ICU	5/15/2023 20:00	10	10.0	8.8	-1.2
ICU	5/15/2023 21:00	10	10.0	11.0	1.0
ICU	5/15/2023 22:00	10	10.0	9.9	-0.1
ICU	5/15/2023 23:00	10	10.0	11.0	1.0
ICU	5/16/2023 0:00	10	10.0	10.4	0.4
ICU	5/16/2023 1:00	10	10.0	10.4	0.4
ICU	5/16/2023 2:00	10	10.0	10.4	0.4
ICU	5/16/2023 3:00	10	10.0	10.4	0.4
ICU	5/16/2023 4:00	10	10.0	10.4	0.4
ICU	5/16/2023 5:00	10	10.0	10.4	0.4
ICU	5/16/2023 6:00	10	10.0	10.4	0.4
ICU	5/16/2023 7:00	10	10.0	10.4	0.4
ICU	5/16/2023 8:00	10	10.0	11.0	1.0
ICU	5/16/2023 9:00	10	12.0	11.0	-1.0
ICU	5/16/2023 10:00	10	12.0	11.0	-1.0
ICU	5/16/2023 11:00	10	12.0	11.0	-1.0
ICU	5/16/2023 12:00	10	12.0	12.6	0.6
ICU	5/16/2023 13:00	10	12.0	12.6	0.6
ICU	5/16/2023 14:00	10	12.0	12.6	0.6
ICU	5/16/2023 15:00	10	12.0	12.6	0.6
ICU	5/16/2023 16:00	10	10.0	9.5	-0.5
ICU	5/16/2023 17:00	10	10.0	9.5	-0.5
ICU	5/16/2023 18:00	10	10.0	9.5	-0.5
ICU	5/16/2023 19:00	10	10.0	9.5	-0.5
ICU	5/16/2023 20:00	10	10.0	11.0	1.0
ICU	5/16/2023 21:00	10	10.0	11.0	1.0
ICU	5/16/2023 22:00	10	10.0	11.0	1.0
ICU	5/16/2023 23:00	9	10.0	9.9	-0.1
ICU	5/17/2023 0:00	9	10.0	10.4	0.4
ICU	5/17/2023 1:00	9	10.0	10.4	0.4
ICU	5/17/2023 2:00	9	10.0	10.4	0.4
ICU	5/17/2023 3:00	9	10.0	10.4	0.4
ICU	5/17/2023 4:00	9	10.0	10.4	0.4
ICU	5/17/2023 5:00	9	10.0	10.4	0.4
ICU	5/17/2023 6:00	9	10.0	10.4	0.4
ICU	5/17/2023 7:00	9	10.0	10.4	0.4
ICU	5/17/2023 8:00	9	12.0	12.6	0.6
ICU	5/17/2023 9:00	9	12.0	12.6	0.6
ICU	5/17/2023 10:00	9	12.0	12.6	0.6
ICU	5/17/2023 11:00	9	12.0	12.6	0.6
ICU	5/17/2023 12:00	9	12.0	12.6	0.6

ICU	5/17/2023 13:00	9	12.0	12.6	0.6
ICU	5/17/2023 14:00	9	12.0	12.6	0.6
ICU	5/17/2023 15:00	9	12.0	12.6	0.6
ICU	5/17/2023 16:00	10	12.0	9.9	-2.1
ICU	5/17/2023 17:00	10	12.0	11.0	-1.0
ICU	5/17/2023 18:00	10	12.0	11.0	-1.0
ICU	5/17/2023 19:00	10	12.0	11.0	-1.0
ICU	5/17/2023 20:00	10	12.0	12.6	0.6
ICU	5/17/2023 21:00	10	12.0	12.6	0.6
ICU	5/17/2023 22:00	10	12.0	12.6	0.6
ICU	5/17/2023 23:00	10	12.0	12.6	0.6
ICU	5/18/2023 0:00	10	12.0	11.9	-0.2
ICU	5/18/2023 1:00	10	12.0	11.9	-0.2
ICU	5/18/2023 2:00	10	12.0	11.9	-0.2
ICU	5/18/2023 3:00	11	12.0	10.7	-1.3
ICU	5/18/2023 4:00	11	12.0	11.9	-0.2
ICU	5/18/2023 5:00	11	12.0	11.9	-0.2
ICU	5/18/2023 6:00	11	12.0	11.9	-0.2
ICU	5/18/2023 7:00	11	12.0	11.9	-0.2
ICU	5/18/2023 8:00	11	12.0	15.0	3.0
ICU	5/18/2023 9:00	11	12.0	15.0	3.0
ICU	5/18/2023 10:00	13	14.0	15.0	1.0
ICU	5/18/2023 11:00	13	14.0	15.0	1.0
ICU	5/18/2023 12:00	13	14.0	15.0	1.0
ICU	5/18/2023 13:00	13	14.0	15.0	1.0
ICU	5/18/2023 14:00	13	14.0	15.0	1.0
ICU	5/18/2023 15:00	13	14.0	15.0	1.0
ICU	5/18/2023 16:00	12	12.0	12.6	0.6
ICU	5/18/2023 17:00	9	12.0	12.6	0.6
ICU	5/18/2023 18:00	9	12.0	12.6	0.6
ICU	5/18/2023 19:00	9	12.0	12.6	0.6
ICU	5/18/2023 20:00	9	12.0	12.6	0.6
ICU	5/18/2023 21:00	9	12.0	12.6	0.6
ICU	5/18/2023 22:00	9	12.0	12.6	0.6
ICU	5/18/2023 23:00	9	12.0	12.6	0.6
ICU	5/19/2023 0:00	9	12.0	11.9	-0.2
ICU	5/19/2023 1:00	9	12.0	11.9	-0.2
ICU	5/19/2023 2:00	9	12.0	11.9	-0.2
ICU	5/19/2023 3:00	9	12.0	11.9	-0.2
ICU	5/19/2023 4:00	9	12.0	11.9	-0.2
ICU	5/19/2023 5:00	9	12.0	11.9	-0.2
ICU	5/19/2023 6:00	9	12.0	11.9	-0.2
ICU	5/19/2023 7:00	9	12.0	11.9	-0.2
ICU	5/19/2023 8:00	10	10.0	11.0	1.0
ICU	5/19/2023 9:00	10	10.0	11.0	1.0
ICU	5/19/2023 10:00	10	10.0	11.0	1.0
ICU	5/19/2023 11:00	10	10.0	11.0	1.0

ICU	5/19/2023 12:00	10	10.0	12.6	2.6
ICU	5/19/2023 13:00	10	10.0	12.6	2.6
ICU	5/19/2023 14:00	10	12.0	12.6	0.6
ICU	5/19/2023 15:00	10	12.0	12.6	0.6
ICU	5/19/2023 16:00	10	12.0	11.0	-1.0
ICU	5/19/2023 17:00	11	12.0	11.0	-1.0
ICU	5/19/2023 18:00	11	12.0	11.0	-1.0
ICU	5/19/2023 19:00	11	12.0	11.0	-1.0
ICU	5/19/2023 20:00	11	14.0	13.3	-0.7
ICU	5/19/2023 21:00	11	14.0	13.3	-0.7
ICU	5/19/2023 22:00	11	14.0	13.3	-0.7
ICU	5/19/2023 23:00	11	14.0	13.3	-0.7
ICU	5/20/2023 0:00	12	14.0	13.3	-0.7
ICU	5/20/2023 1:00	12	14.0	13.3	-0.7
ICU	5/20/2023 2:00	12	14.0	13.3	-0.7
ICU	5/20/2023 3:00	12	14.0	13.3	-0.7
ICU	5/20/2023 4:00	12	14.0	13.3	-0.7
ICU	5/20/2023 5:00	12	14.0	13.3	-0.7
ICU	5/20/2023 6:00	12	14.0	13.3	-0.7
ICU	5/20/2023 7:00	12	14.0	13.3	-0.7
ICU	5/20/2023 8:00	12	14.0	11.9	-2.2
ICU	5/20/2023 9:00	12	14.0	11.9	-2.2
ICU	5/20/2023 10:00	12	14.0	11.9	-2.2
ICU	5/20/2023 11:00	12	14.0	10.7	-3.3
ICU	5/20/2023 12:00	10	14.0	11.9	-2.2
ICU	5/20/2023 13:00	10	14.0	11.9	-2.2
ICU	5/20/2023 14:00	11	14.0	11.9	-2.2
ICU	5/20/2023 15:00	11	14.0	11.9	-2.2
ICU	5/20/2023 16:00	11	14.0	12.6	-1.4
ICU	5/20/2023 17:00	11	14.0	12.6	-1.4
ICU	5/20/2023 18:00	11	14.0	12.6	-1.4
ICU	5/20/2023 19:00	11	14.0	12.6	-1.4
ICU	5/20/2023 20:00	12	10.0	12.6	2.6
ICU	5/20/2023 21:00	12	10.0	12.6	2.6
ICU	5/20/2023 22:00	12	10.0	12.6	2.6
ICU	5/20/2023 23:00	12	10.0	12.6	2.6
ICU	5/21/2023 0:00	12	10.0	12.6	2.6
ICU	5/21/2023 1:00	12	10.0	12.6	2.6
ICU	5/21/2023 2:00	12	10.0	12.6	2.6
ICU	5/21/2023 3:00	12	10.0	12.6	2.6
ICU	5/21/2023 4:00	12	10.0	12.6	2.6
ICU	5/21/2023 5:00	12	10.0	12.6	2.6
ICU	5/21/2023 6:00	12	10.0	12.6	2.6
ICU	5/21/2023 7:00	12	10.0	12.6	2.6
ICU	5/21/2023 8:00	12	10.0	11.0	1.0
ICU	5/21/2023 9:00	12	10.0	11.0	1.0
ICU	5/21/2023 10:00	12	10.0	11.0	1.0

ICU	5/21/2023 11:00	12	10.0	11.0	1.0
ICU	5/21/2023 12:00	12	10.0	11.0	1.0
ICU	5/21/2023 13:00	12	10.0	11.0	1.0
ICU	5/21/2023 14:00	12	10.0	11.0	1.0
ICU	5/21/2023 15:00	12	10.0	11.0	1.0
ICU	5/21/2023 16:00	12	10.0	11.0	1.0
ICU	5/21/2023 17:00	8	10.0	11.0	1.0
ICU	5/21/2023 18:00	8	10.0	11.0	1.0
ICU	5/21/2023 19:00	8	10.0	11.0	1.0
ICU	5/21/2023 20:00	9	12.0	12.6	0.6
ICU	5/21/2023 21:00	9	12.0	12.6	0.6
ICU	5/21/2023 22:00	9	12.0	12.6	0.6
ICU	5/21/2023 23:00	9	12.0	12.6	0.6
ICU	5/22/2023 0:00	9	12.0	12.6	0.6
ICU	5/22/2023 1:00	9	12.0	12.6	0.6
ICU	5/22/2023 2:00	9	12.0	12.6	0.6
ICU	5/22/2023 3:00	9	12.0	12.6	0.6
ICU	5/22/2023 4:00	9	12.0	12.6	0.6
ICU	5/22/2023 5:00	9	12.0	12.6	0.6
ICU	5/22/2023 6:00	9	12.0	12.6	0.6
ICU	5/22/2023 7:00	10	12.0	12.6	0.6
ICU	5/22/2023 8:00	10	12.0	12.6	0.6
ICU	5/22/2023 9:00	10	12.0	12.6	0.6
ICU	5/22/2023 10:00	10	12.0	12.6	0.6
ICU	5/22/2023 11:00	10	12.0	12.6	0.6
ICU	5/22/2023 12:00	11	12.0	12.6	0.6
ICU	5/22/2023 13:00	11	12.0	12.6	0.6
ICU	5/22/2023 14:00	10	12.0	11.9	-0.2
ICU	5/22/2023 15:00	10	12.0	11.9	-0.2
ICU	5/22/2023 16:00	10	12.0	11.0	-1.0
ICU	5/22/2023 17:00	11	12.0	11.0	-1.0
ICU	5/22/2023 18:00	11	12.0	11.0	-1.0
ICU	5/22/2023 19:00	11	12.0	11.0	-1.0
ICU	5/22/2023 20:00	11	12.0	12.6	0.6
ICU	5/22/2023 21:00	11	12.0	12.6	0.6
ICU	5/22/2023 22:00	11	12.0	12.6	0.6
ICU	5/22/2023 23:00	12	12.0	12.6	0.6
ICU	5/23/2023 0:00	12	12.0	10.7	-1.3
ICU	5/23/2023 1:00	12	12.0	11.9	-0.2
ICU	5/23/2023 2:00	12	12.0	11.9	-0.2
ICU	5/23/2023 3:00	12	12.0	11.9	-0.2
ICU	5/23/2023 4:00	12	12.0	11.9	-0.2
ICU	5/23/2023 5:00	12	12.0	11.9	-0.2
ICU	5/23/2023 6:00	12	12.0	11.9	-0.2
ICU	5/23/2023 7:00	12	12.0	11.9	-0.2
ICU	5/23/2023 8:00	11	12.0	11.9	-0.2
ICU	5/23/2023 9:00	11	12.0	11.9	-0.2

ICU	5/23/2023 10:00	10	12.0	11.9	-0.2
ICU	5/23/2023 11:00	10	12.0	11.9	-0.2
ICU	5/23/2023 12:00	10	12.0	10.4	-1.6
ICU	5/23/2023 13:00	10	12.0	10.4	-1.6
ICU	5/23/2023 14:00	10	12.0	10.4	-1.6
ICU	5/23/2023 15:00	10	12.0	11.1	-0.9
ICU	5/23/2023 16:00	10	12.0	11.0	-1.0
ICU	5/23/2023 17:00	10	12.0	11.0	-1.0
ICU	5/23/2023 18:00	10	12.0	11.0	-1.0
ICU	5/23/2023 19:00	10	12.0	11.0	-1.0
ICU	5/23/2023 20:00	10	12.0	11.0	-1.0
ICU	5/23/2023 21:00	11	12.0	11.0	-1.0
ICU	5/23/2023 22:00	11	12.0	11.0	-1.0
ICU	5/23/2023 23:00	11	12.0	11.0	-1.0
ICU	5/24/2023 0:00	11	12.0	12.6	0.6
ICU	5/24/2023 1:00	11	12.0	12.6	0.6
ICU	5/24/2023 2:00	11	12.0	12.6	0.6
ICU	5/24/2023 3:00	11	12.0	12.6	0.6
ICU	5/24/2023 4:00	11	12.0	12.6	0.6
ICU	5/24/2023 5:00	11	12.0	12.6	0.6
ICU	5/24/2023 6:00	11	12.0	12.6	0.6
ICU	5/24/2023 7:00	11	12.0	12.6	0.6
ICU	5/24/2023 8:00	11	12.0	14.2	2.2
ICU	5/24/2023 9:00	11	12.0	14.2	2.2
ICU	5/24/2023 10:00	11	12.0	14.2	2.2
ICU	5/24/2023 11:00	11	12.0	14.2	2.2
ICU	5/24/2023 12:00	12	12.0	15.0	3.0
ICU	5/24/2023 13:00	12	12.0	15.0	3.0
ICU	5/24/2023 14:00	12	12.0	15.0	3.0
ICU	5/24/2023 15:00	12	12.0	15.0	3.0
ICU	5/24/2023 16:00	11	12.0	12.6	0.6
ICU	5/24/2023 17:00	11	12.0	12.6	0.6
ICU	5/24/2023 18:00	11	12.0	12.6	0.6
ICU	5/24/2023 19:00	11	12.0	12.6	0.6
ICU	5/24/2023 20:00	11	12.0	14.2	2.2
ICU	5/24/2023 21:00	11	12.0	13.1	1.1
ICU	5/24/2023 22:00	11	12.0	13.1	1.1
ICU	5/24/2023 23:00	11	12.0	14.2	2.2
ICU	5/25/2023 0:00	11	12.0	14.2	2.2
ICU	5/25/2023 1:00	11	12.0	14.2	2.2
ICU	5/25/2023 2:00	11	12.0	14.2	2.2
ICU	5/25/2023 3:00	11	12.0	14.2	2.2
ICU	5/25/2023 4:00	11	12.0	13.1	1.1
ICU	5/25/2023 5:00	11	12.0	14.2	2.2
ICU	5/25/2023 6:00	11	12.0	14.2	2.2
ICU	5/25/2023 7:00	11	12.0	14.2	2.2
ICU	5/25/2023 8:00	11	12.0	12.6	0.6

ICU	5/25/2023 9:00	11	12.0	12.6	0.6
ICU	5/25/2023 10:00	11	12.0	12.6	0.6
ICU	5/25/2023 11:00	11	12.0	12.6	0.6
ICU	5/25/2023 12:00	11	12.0	12.6	0.6
ICU	5/25/2023 13:00	11	12.0	12.6	0.6
ICU	5/25/2023 14:00	11	12.0	12.6	0.6
ICU	5/25/2023 15:00	11	12.0	12.6	0.6
ICU	5/25/2023 16:00	11	12.0	11.0	-1.0
ICU	5/25/2023 17:00	11	12.0	11.0	-1.0
ICU	5/25/2023 18:00	11	12.0	11.0	-1.0
ICU	5/25/2023 19:00	11	12.0	11.0	-1.0
ICU	5/25/2023 20:00	11	12.0	12.6	0.6
ICU	5/25/2023 21:00	11	12.0	12.6	0.6
ICU	5/25/2023 22:00	11	12.0	12.6	0.6
ICU	5/25/2023 23:00	11	12.0	12.6	0.6
Med- Surg ICU	1/1/2023 0:00	8	9.0	7.7	-1.3
Med- Surg ICU	1/1/2023 1:00	8	9.0	7.7	-1.3
Med- Surg ICU	1/1/2023 2:00	8	9.0	7.7	-1.3
Med- Surg ICU	1/1/2023 3:00	8	9.0	7.7	-1.3
Med- Surg ICU	1/1/2023 4:00	8	9.0	7.7	-1.3
Med- Surg ICU	1/1/2023 5:00	8	9.0	7.7	-1.3
Med- Surg ICU	1/1/2023 6:00	8	9.0	7.7	-1.3
Med- Surg ICU	1/1/2023 7:00	8	9.0	7.7	-1.3
Med- Surg ICU	1/1/2023 8:00	8	9.0	7.5	-1.5
Med- Surg ICU	1/1/2023 9:00	8	9.0	7.5	-1.5
Med- Surg ICU	1/1/2023 10:00	8	13.0	8.7	-4.3
Med- Surg ICU	1/1/2023 11:00	8	13.0	8.7	-4.3
Med- Surg ICU	1/1/2023 12:00	7	13.0	8.7	-4.3
Med- Surg ICU	1/1/2023 13:00	7	13.0	8.7	-4.3
Med- Surg ICU	1/1/2023 14:00	8	13.0	8.7	-4.3
Med- Surg ICU	1/1/2023 15:00	8	13.0	8.7	-4.3
Med- Surg ICU	1/1/2023 16:00	7	13.0	9.0	-4.0
Med- Surg ICU	1/1/2023 17:00	8	13.0	9.0	-4.0
Med- Surg ICU	1/1/2023 18:00	8	13.0	9.0	-4.0
Med- Surg ICU	1/1/2023 19:00	8	13.0	9.0	-4.0
Med- Surg ICU	1/1/2023 20:00	8	13.0	9.0	-4.0
Med- Surg ICU	1/1/2023 21:00	8	13.0	9.0	-4.0
Med- Surg ICU	1/1/2023 22:00	8	13.0	9.0	-4.0
Med- Surg ICU	1/1/2023 23:00	8	13.0	9.0	-4.0
Med- Surg ICU	1/2/2023 0:00	8	13.0	9.0	-4.0
Med- Surg ICU	1/2/2023 1:00	8	13.0	7.7	-5.3
Med- Surg ICU	1/2/2023 2:00	8	13.0	7.7	-5.3
Med- Surg ICU	1/2/2023 3:00	8	13.0	7.7	-5.3
Med- Surg ICU	1/2/2023 4:00	8	13.0	7.7	-5.3
Med- Surg ICU	1/2/2023 5:00	8	13.0	7.7	-5.3
Med- Surg ICU	1/2/2023 6:00	8	13.0	7.7	-5.3
Med- Surg ICU	1/2/2023 7:00	8	11.0	7.7	-3.3

Med- Surg ICU	1/2/2023 8:00	8	12.0	7.5	-4.5
Med- Surg ICU	1/2/2023 9:00	8	12.0	7.5	-4.5
Med- Surg ICU	1/2/2023 10:00	7	12.0	7.5	-4.5
Med- Surg ICU	1/2/2023 11:00	7	12.0	7.5	-4.5
Med- Surg ICU	1/2/2023 12:00	7	12.0	7.5	-4.5
Med- Surg ICU	1/2/2023 13:00	7	12.0	6.5	-5.5
Med- Surg ICU	1/2/2023 14:00	7	12.0	7.5	-4.5
Med- Surg ICU	1/2/2023 15:00	7	12.0	7.5	-4.5
Med- Surg ICU	1/2/2023 16:00	7	12.0	9.1	-3.0
Med- Surg ICU	1/2/2023 17:00	7	12.0	9.1	-3.0
Med- Surg ICU	1/2/2023 18:00	7	12.0	9.1	-3.0
Med- Surg ICU	1/2/2023 19:00	7	12.0	9.1	-3.0
Med- Surg ICU	1/2/2023 20:00	7	10.0	6.0	-4.0
Med- Surg ICU	1/2/2023 21:00	7	10.0	6.0	-4.0
Med- Surg ICU	1/2/2023 22:00	7	10.0	6.0	-4.0
Med- Surg ICU	1/2/2023 23:00	7	10.0	6.0	-4.0
Med- Surg ICU	1/3/2023 0:00	7	10.0	5.8	-4.2
Med- Surg ICU	1/3/2023 1:00	7	10.0	6.4	-3.6
Med- Surg ICU	1/3/2023 2:00	7	10.0	6.4	-3.6
Med- Surg ICU	1/3/2023 3:00	7	10.0	6.4	-3.6
Med- Surg ICU	1/3/2023 4:00	7	10.0	6.4	-3.6
Med- Surg ICU	1/3/2023 5:00	7	10.0	6.4	-3.6
Med- Surg ICU	1/3/2023 6:00	7	10.0	6.4	-3.6
Med- Surg ICU	1/3/2023 7:00	7	10.0	6.4	-3.6
Med- Surg ICU	1/3/2023 8:00	7	10.0	8.1	-1.9
Med- Surg ICU	1/3/2023 9:00	7	10.0	8.7	-1.3
Med- Surg ICU	1/3/2023 10:00	7	10.0	8.7	-1.3
Med- Surg ICU	1/3/2023 11:00	7	10.0	8.7	-1.3
Med- Surg ICU	1/3/2023 12:00	7	10.0	8.7	-1.3
Med- Surg ICU	1/3/2023 13:00	7	10.0	7.8	-2.2
Med- Surg ICU	1/3/2023 14:00	8	11.0	8.7	-2.3
Med- Surg ICU	1/3/2023 15:00	8	11.0	8.7	-2.3
Med- Surg ICU	1/3/2023 16:00	8	11.0	9.0	-2.0
Med- Surg ICU	1/3/2023 17:00	8	11.0	9.0	-2.0
Med- Surg ICU	1/3/2023 18:00	8	11.0	8.3	-2.7
Med- Surg ICU	1/3/2023 19:00	8	11.0	8.3	-2.7
Med- Surg ICU	1/3/2023 20:00	8	11.0	6.4	-4.6
Med- Surg ICU	1/3/2023 21:00	7	11.0	6.4	-4.6
Med- Surg ICU	1/3/2023 22:00	6	10.0	6.4	-3.6
Med- Surg ICU	1/3/2023 23:00	6	10.0	6.4	-3.6
Med- Surg ICU	1/4/2023 0:00	6	10.0	6.4	-3.6
Med- Surg ICU	1/4/2023 1:00	6	10.0	6.4	-3.6
Med- Surg ICU	1/4/2023 2:00	6	10.0	6.4	-3.6
Med- Surg ICU	1/4/2023 3:00	6	10.0	6.4	-3.6
Med- Surg ICU	1/4/2023 4:00	6	10.0	6.4	-3.6
Med- Surg ICU	1/4/2023 5:00	7	10.0	6.4	-3.6
Med- Surg ICU	1/4/2023 6:00	7	10.0	6.4	-3.6

Med- Surg ICU	1/4/2023 7:00	7	10.0	6.4	-3.6
Med- Surg ICU	1/4/2023 8:00	7	13.0	8.7	-4.3
Med- Surg ICU	1/4/2023 9:00	7	13.0	8.7	-4.3
Med- Surg ICU	1/4/2023 10:00	6	13.0	8.7	-4.3
Med- Surg ICU	1/4/2023 11:00	6	13.0	8.7	-4.3
Med- Surg ICU	1/4/2023 12:00	6	13.0	8.7	-4.3
Med- Surg ICU	1/4/2023 13:00	6	13.0	8.7	-4.3
Med- Surg ICU	1/4/2023 14:00	6	13.0	7.8	-5.2
Med- Surg ICU	1/4/2023 15:00	6	11.0	8.7	-2.3
Med- Surg ICU	1/4/2023 16:00	6	11.0	7.7	-3.3
Med- Surg ICU	1/4/2023 17:00	6	11.0	7.7	-3.3
Med- Surg ICU	1/4/2023 18:00	6	11.0	7.7	-3.3
Med- Surg ICU	1/4/2023 19:00	6	11.0	7.7	-3.3
Med- Surg ICU	1/4/2023 20:00	6	9.0	6.4	-2.6
Med- Surg ICU	1/4/2023 21:00	6	9.0	6.4	-2.6
Med- Surg ICU	1/4/2023 22:00	6	9.0	6.4	-2.6
Med- Surg ICU	1/4/2023 23:00	6	9.0	6.4	-2.6
Med- Surg ICU	1/5/2023 0:00	6	9.0	7.6	-1.5
Med- Surg ICU	1/5/2023 1:00	7	9.0	7.6	-1.5
Med- Surg ICU	1/5/2023 2:00	7	9.0	7.6	-1.5
Med- Surg ICU	1/5/2023 3:00	7	9.0	7.6	-1.5
Med- Surg ICU	1/5/2023 4:00	7	9.0	7.6	-1.5
Med- Surg ICU	1/5/2023 5:00	7	9.0	7.6	-1.5
Med- Surg ICU	1/5/2023 6:00	7	9.0	7.6	-1.5
Med- Surg ICU	1/5/2023 7:00	7	9.0	7.6	-1.5
Med- Surg ICU	1/5/2023 8:00	7	13.0	10.0	-3.0
Med- Surg ICU	1/5/2023 9:00	8	13.0	8.7	-4.3
Med- Surg ICU	1/5/2023 10:00	8	13.0	8.7	-4.3
Med- Surg ICU	1/5/2023 11:00	8	13.0	8.7	-4.3
Med- Surg ICU	1/5/2023 12:00	9	13.0	8.7	-4.3
Med- Surg ICU	1/5/2023 13:00	9	13.0	8.7	-4.3
Med- Surg ICU	1/5/2023 14:00	9	13.0	8.7	-4.3
Med- Surg ICU	1/5/2023 15:00	9	13.0	8.7	-4.3
Med- Surg ICU	1/5/2023 16:00	9	13.0	9.0	-4.0
Med- Surg ICU	1/5/2023 17:00	9	13.0	9.0	-4.0
Med- Surg ICU	1/5/2023 18:00	9	13.0	9.0	-4.0
Med- Surg ICU	1/5/2023 19:00	9	13.0	9.0	-4.0
Med- Surg ICU	1/5/2023 20:00	9	13.0	7.6	-5.5
Med- Surg ICU	1/5/2023 21:00	9	13.0	7.6	-5.5
Med- Surg ICU	1/5/2023 22:00	9	13.0	7.6	-5.5
Med- Surg ICU	1/5/2023 23:00	9	13.0	7.6	-5.5
Med- Surg ICU	1/6/2023 0:00	9	10.0	7.6	-2.5
Med- Surg ICU	1/6/2023 1:00	9	10.0	7.6	-2.5
Med- Surg ICU	1/6/2023 2:00	9	10.0	7.6	-2.5
Med- Surg ICU	1/6/2023 3:00	9	10.0	7.6	-2.5
Med- Surg ICU	1/6/2023 4:00	9	10.0	7.6	-2.5
Med- Surg ICU	1/6/2023 5:00	9	10.0	7.6	-2.5

Med- Surg ICU	1/6/2023 6:00	9	10.0	7.6	-2.5
Med- Surg ICU	1/6/2023 7:00	9	13.0	7.6	-5.5
Med- Surg ICU	1/6/2023 8:00	9	13.0	10.0	-3.0
Med- Surg ICU	1/6/2023 9:00	9	13.0	10.0	-3.0
Med- Surg ICU	1/6/2023 10:00	9	13.0	10.0	-3.0
Med- Surg ICU	1/6/2023 11:00	9	13.0	8.7	-4.3
Med- Surg ICU	1/6/2023 12:00	9	13.0	8.7	-4.3
Med- Surg ICU	1/6/2023 13:00	9	13.0	8.7	-4.3
Med- Surg ICU	1/6/2023 14:00	9	13.0	8.7	-4.3
Med- Surg ICU	1/6/2023 15:00	9	13.0	8.7	-4.3
Med- Surg ICU	1/6/2023 16:00	9	13.0	10.3	-2.7
Med- Surg ICU	1/6/2023 17:00	9	13.0	10.3	-2.7
Med- Surg ICU	1/6/2023 18:00	9	13.0	10.3	-2.7
Med- Surg ICU	1/6/2023 19:00	9	13.0	10.3	-2.7
Med- Surg ICU	1/6/2023 20:00	10	13.0	10.6	-2.4
Med- Surg ICU	1/6/2023 21:00	10	12.0	10.6	-1.4
Med- Surg ICU	1/6/2023 22:00	10	12.0	10.6	-1.4
Med- Surg ICU	1/6/2023 23:00	10	12.0	10.6	-1.4
Med- Surg ICU	1/7/2023 0:00	10	12.0	10.3	-1.7
Med- Surg ICU	1/7/2023 1:00	10	12.0	10.3	-1.7
Med- Surg ICU	1/7/2023 2:00	10	12.0	10.3	-1.7
Med- Surg ICU	1/7/2023 3:00	10	12.0	10.3	-1.7
Med- Surg ICU	1/7/2023 4:00	10	12.0	10.3	-1.7
Med- Surg ICU	1/7/2023 5:00	10	12.0	10.3	-1.7
Med- Surg ICU	1/7/2023 6:00	10	12.0	10.3	-1.7
Med- Surg ICU	1/7/2023 7:00	10	12.0	10.3	-1.7
Med- Surg ICU	1/7/2023 8:00	10	12.0	8.7	-3.3
Med- Surg ICU	1/7/2023 9:00	10	12.0	8.7	-3.3
Med- Surg ICU	1/7/2023 10:00	10	12.0	8.7	-3.3
Med- Surg ICU	1/7/2023 11:00	10	12.0	8.7	-3.3
Med- Surg ICU	1/7/2023 12:00	11	13.0	10.0	-3.0
Med- Surg ICU	1/7/2023 13:00	11	13.0	10.0	-3.0
Med- Surg ICU	1/7/2023 14:00	11	13.0	10.0	-3.0
Med- Surg ICU	1/7/2023 15:00	11	13.0	10.0	-3.0
Med- Surg ICU	1/7/2023 16:00	9	13.0	10.3	-2.7
Med- Surg ICU	1/7/2023 17:00	9	13.0	10.3	-2.7
Med- Surg ICU	1/7/2023 18:00	9	13.0	10.3	-2.7
Med- Surg ICU	1/7/2023 19:00	9	13.0	10.3	-2.7
Med- Surg ICU	1/7/2023 20:00	9	13.0	10.3	-2.7
Med- Surg ICU	1/7/2023 21:00	9	13.0	10.3	-2.7
Med- Surg ICU	1/7/2023 22:00	9	13.0	10.3	-2.7
Med- Surg ICU	1/7/2023 23:00	9	13.0	10.3	-2.7
Med- Surg ICU	1/8/2023 0:00	9	13.0	10.3	-2.7
Med- Surg ICU	1/8/2023 1:00	9	13.0	10.3	-2.7
Med- Surg ICU	1/8/2023 2:00	9	13.0	10.3	-2.7
Med- Surg ICU	1/8/2023 3:00	9	13.0	10.3	-2.7
Med- Surg ICU	1/8/2023 4:00	9	13.0	10.3	-2.7

Med- Surg ICU	1/8/2023 5:00	9	13.0	10.3	-2.7
Med- Surg ICU	1/8/2023 6:00	9	13.0	10.3	-2.7
Med- Surg ICU	1/8/2023 7:00	9	13.0	10.3	-2.7
Med- Surg ICU	1/8/2023 8:00	9	13.0	7.5	-5.5
Med- Surg ICU	1/8/2023 9:00	9	13.0	7.5	-5.5
Med- Surg ICU	1/8/2023 10:00	9	13.0	7.5	-5.5
Med- Surg ICU	1/8/2023 11:00	9	13.0	7.5	-5.5
Med- Surg ICU	1/8/2023 12:00	9	13.0	7.5	-5.5
Med- Surg ICU	1/8/2023 13:00	11	13.0	6.2	-6.8
Med- Surg ICU	1/8/2023 14:00	11	13.0	6.2	-6.8
Med- Surg ICU	1/8/2023 15:00	11	13.0	6.2	-6.8
Med- Surg ICU	1/8/2023 16:00	10	13.0	6.4	-6.6
Med- Surg ICU	1/8/2023 17:00	10	13.0	6.4	-6.6
Med- Surg ICU	1/8/2023 18:00	10	13.0	7.7	-5.3
Med- Surg ICU	1/8/2023 19:00	10	13.0	9.0	-4.0
Med- Surg ICU	1/8/2023 20:00	10	13.0	10.3	-2.7
Med- Surg ICU	1/8/2023 21:00	10	13.0	9.6	-3.4
Med- Surg ICU	1/8/2023 22:00	10	13.0	9.0	-4.0
Med- Surg ICU	1/8/2023 23:00	10	13.0	9.0	-4.0
Med- Surg ICU	1/9/2023 0:00	10	13.0	9.0	-4.0
Med- Surg ICU	1/9/2023 1:00	10	13.0	9.0	-4.0
Med- Surg ICU	1/9/2023 2:00	10	13.0	9.0	-4.0
Med- Surg ICU	1/9/2023 3:00	10	13.0	9.0	-4.0
Med- Surg ICU	1/9/2023 4:00	10	13.0	9.0	-4.0
Med- Surg ICU	1/9/2023 5:00	10	13.0	9.0	-4.0
Med- Surg ICU	1/9/2023 6:00	10	13.0	9.0	-4.0
Med- Surg ICU	1/9/2023 7:00	10	12.0	6.4	-5.6
Med- Surg ICU	1/9/2023 8:00	10	12.0	8.7	-3.3
Med- Surg ICU	1/9/2023 9:00	10	12.0	8.7	-3.3
Med- Surg ICU	1/9/2023 10:00	10	12.0	8.7	-3.3
Med- Surg ICU	1/9/2023 11:00	10	12.0	8.7	-3.3
Med- Surg ICU	1/9/2023 12:00	10	12.0	9.3	-2.7
Med- Surg ICU	1/9/2023 13:00	10	12.0	8.7	-3.3
Med- Surg ICU	1/9/2023 14:00	10	12.0	8.7	-3.3
Med- Surg ICU	1/9/2023 15:00	10	12.0	8.7	-3.3
Med- Surg ICU	1/9/2023 16:00	10	12.0	7.7	-4.3
Med- Surg ICU	1/9/2023 17:00	10	12.0	7.7	-4.3
Med- Surg ICU	1/9/2023 18:00	10	12.0	7.7	-4.3
Med- Surg ICU	1/9/2023 19:00	10	10.0	9.0	-1.0
Med- Surg ICU	1/9/2023 20:00	10	11.0	7.7	-3.3
Med- Surg ICU	1/9/2023 21:00	10	11.0	7.7	-3.3
Med- Surg ICU	1/9/2023 22:00	10	11.0	7.7	-3.3
Med- Surg ICU	1/9/2023 23:00	10	11.0	7.7	-3.3
Med- Surg ICU	1/10/2023 0:00	10	11.0	7.7	-3.3
Med- Surg ICU	1/10/2023 1:00	10	11.0	7.7	-3.3
Med- Surg ICU	1/10/2023 2:00	10	11.0	7.7	-3.3
Med- Surg ICU	1/10/2023 3:00	10	11.0	7.7	-3.3

Med- Surg ICU	1/10/2023 4:00	10	11.0	7.7	-3.3
Med- Surg ICU	1/10/2023 5:00	10	11.0	7.7	-3.3
Med- Surg ICU	1/10/2023 6:00	10	11.0	7.7	-3.3
Med- Surg ICU	1/10/2023 7:00	10	11.0	7.7	-3.3
Med- Surg ICU	1/10/2023 8:00	10	13.0	11.2	-1.8
Med- Surg ICU	1/10/2023 9:00	10	13.0	11.2	-1.8
Med- Surg ICU	1/10/2023 10:00	10	13.0	10.3	-2.7
Med- Surg ICU	1/10/2023 11:00	10	13.0	11.2	-1.8
Med- Surg ICU	1/10/2023 12:00	10	13.0	10.3	-2.7
Med- Surg ICU	1/10/2023 13:00	10	13.0	10.3	-2.7
Med- Surg ICU	1/10/2023 14:00	10	13.0	11.2	-1.8
Med- Surg ICU	1/10/2023 15:00	12	13.0	10.0	-3.0
Med- Surg ICU	1/10/2023 16:00	12	13.0	9.3	-3.7
Med- Surg ICU	1/10/2023 17:00	12	13.0	10.3	-2.7
Med- Surg ICU	1/10/2023 18:00	12	13.0	10.3	-2.7
Med- Surg ICU	1/10/2023 19:00	12	13.0	10.3	-2.7
Med- Surg ICU	1/10/2023 20:00	12	13.0	10.3	-2.7
Med- Surg ICU	1/10/2023 21:00	12	13.0	7.7	-5.3
Med- Surg ICU	1/10/2023 22:00	12	13.0	6.7	-6.3
Med- Surg ICU	1/10/2023 23:00	12	13.0	7.7	-5.3
Med- Surg ICU	1/11/2023 0:00	12	13.0	7.7	-5.3
Med- Surg ICU	1/11/2023 1:00	12	13.0	7.7	-5.3
Med- Surg ICU	1/11/2023 2:00	12	13.0	7.7	-5.3
Med- Surg ICU	1/11/2023 3:00	12	13.0	7.7	-5.3
Med- Surg ICU	1/11/2023 4:00	12	13.0	7.7	-5.3
Med- Surg ICU	1/11/2023 5:00	12	13.0	7.7	-5.3
Med- Surg ICU	1/11/2023 6:00	12	13.0	7.7	-5.3
Med- Surg ICU	1/11/2023 7:00	12	13.0	7.7	-5.3
Med- Surg ICU	1/11/2023 8:00	12	13.0	10.0	-3.0
Med- Surg ICU	1/11/2023 9:00	12	13.0	10.0	-3.0
Med- Surg ICU	1/11/2023 10:00	12	13.0	10.0	-3.0
Med- Surg ICU	1/11/2023 11:00	12	13.0	10.0	-3.0
Med- Surg ICU	1/11/2023 12:00	12	13.0	11.2	-1.8
Med- Surg ICU	1/11/2023 13:00	12	13.0	10.3	-2.7
Med- Surg ICU	1/11/2023 14:00	12	13.0	11.2	-1.8
Med- Surg ICU	1/11/2023 15:00	12	13.0	11.2	-1.8
Med- Surg ICU	1/11/2023 16:00	12	13.0	10.4	-2.6
Med- Surg ICU	1/11/2023 17:00	12	13.0	12.1	-0.9
Med- Surg ICU	1/11/2023 18:00	12	13.0	12.1	-0.9
Med- Surg ICU	1/11/2023 19:00	12	13.0	12.1	-0.9
Med- Surg ICU	1/11/2023 20:00	10	12.0	9.0	-3.0
Med- Surg ICU	1/11/2023 21:00	10	12.0	9.0	-3.0
Med- Surg ICU	1/11/2023 22:00	10	12.0	9.0	-3.0
Med- Surg ICU	1/11/2023 23:00	10	12.0	9.0	-3.0
Med- Surg ICU	1/12/2023 0:00	10	12.0	10.3	-1.7
Med- Surg ICU	1/12/2023 1:00	10	12.0	9.3	-2.7
Med- Surg ICU	1/12/2023 2:00	10	12.0	10.3	-1.7

Med- Surg ICU	1/12/2023 3:00	10	12.0	10.3	-1.7
Med- Surg ICU	1/12/2023 4:00	10	12.0	10.3	-1.7
Med- Surg ICU	1/12/2023 5:00	10	12.0	10.3	-1.7
Med- Surg ICU	1/12/2023 6:00	10	12.0	10.3	-1.7
Med- Surg ICU	1/12/2023 7:00	10	12.0	10.3	-1.7
Med- Surg ICU	1/12/2023 8:00	10	12.0	11.2	-0.8
Med- Surg ICU	1/12/2023 9:00	11	13.0	12.5	-0.6
Med- Surg ICU	1/12/2023 10:00	11	13.0	12.5	-0.6
Med- Surg ICU	1/12/2023 11:00	11	13.0	12.5	-0.6
Med- Surg ICU	1/12/2023 12:00	12	13.0	11.2	-1.8
Med- Surg ICU	1/12/2023 13:00	12	13.0	11.2	-1.8
Med- Surg ICU	1/12/2023 14:00	12	13.0	11.2	-1.8
Med- Surg ICU	1/12/2023 15:00	12	13.0	11.2	-1.8
Med- Surg ICU	1/12/2023 16:00	12	13.0	10.3	-2.7
Med- Surg ICU	1/12/2023 17:00	12	13.0	10.3	-2.7
Med- Surg ICU	1/12/2023 18:00	12	13.0	10.3	-2.7
Med- Surg ICU	1/12/2023 19:00	12	13.0	10.3	-2.7
Med- Surg ICU	1/12/2023 20:00	11	13.0	9.0	-4.0
Med- Surg ICU	1/12/2023 21:00	11	13.0	9.0	-4.0
Med- Surg ICU	1/12/2023 22:00	11	13.0	9.0	-4.0
Med- Surg ICU	1/12/2023 23:00	11	13.0	9.0	-4.0
Med- Surg ICU	1/13/2023 0:00	11	13.0	9.0	-4.0
Med- Surg ICU	1/13/2023 1:00	11	13.0	9.0	-4.0
Med- Surg ICU	1/13/2023 2:00	11	13.0	9.0	-4.0
Med- Surg ICU	1/13/2023 3:00	11	13.0	9.0	-4.0
Med- Surg ICU	1/13/2023 4:00	11	13.0	9.6	-3.4
Med- Surg ICU	1/13/2023 5:00	11	13.0	10.3	-2.7
Med- Surg ICU	1/13/2023 6:00	11	13.0	10.3	-2.7
Med- Surg ICU	1/13/2023 7:00	11	13.0	10.3	-2.7
Med- Surg ICU	1/13/2023 8:00	11	13.0	10.0	-3.0
Med- Surg ICU	1/13/2023 9:00	11	13.0	10.0	-3.0
Med- Surg ICU	1/13/2023 10:00	11	13.0	10.0	-3.0
Med- Surg ICU	1/13/2023 11:00	10	13.0	10.0	-3.0
Med- Surg ICU	1/13/2023 12:00	10	13.0	10.0	-3.0
Med- Surg ICU	1/13/2023 13:00	11	13.0	10.0	-3.0
Med- Surg ICU	1/13/2023 14:00	11	13.0	10.0	-3.0
Med- Surg ICU	1/13/2023 15:00	11	13.0	10.0	-3.0
Med- Surg ICU	1/13/2023 16:00	11	13.0	12.2	-0.8
Med- Surg ICU	1/13/2023 17:00	11	13.0	12.8	-0.2
Med- Surg ICU	1/13/2023 18:00	11	13.0	12.8	-0.2
Med- Surg ICU	1/13/2023 19:00	11	13.0	12.8	-0.2
Med- Surg ICU	1/13/2023 20:00	11	13.0	9.0	-4.0
Med- Surg ICU	1/13/2023 21:00	12	13.0	10.3	-2.7
Med- Surg ICU	1/13/2023 22:00	12	13.0	10.3	-2.7
Med- Surg ICU	1/13/2023 23:00	12	13.0	10.6	-2.4
Med- Surg ICU	1/14/2023 0:00	12	13.0	10.3	-2.7
Med- Surg ICU	1/14/2023 1:00	12	13.0	10.3	-2.7

Med- Surg ICU	1/14/2023 2:00	12	13.0	10.3	-2.7
Med- Surg ICU	1/14/2023 3:00	12	13.0	10.3	-2.7
Med- Surg ICU	1/14/2023 4:00	12	13.0	10.3	-2.7
Med- Surg ICU	1/14/2023 5:00	12	13.0	10.3	-2.7
Med- Surg ICU	1/14/2023 6:00	12	13.0	10.3	-2.7
Med- Surg ICU	1/14/2023 7:00	12	13.0	10.3	-2.7
Med- Surg ICU	1/14/2023 8:00	12	13.0	10.0	-3.0
Med- Surg ICU	1/14/2023 9:00	12	13.0	10.0	-3.0
Med- Surg ICU	1/14/2023 10:00	12	13.0	10.0	-3.0
Med- Surg ICU	1/14/2023 11:00	12	13.0	10.0	-3.0
Med- Surg ICU	1/14/2023 12:00	12	13.0	11.2	-1.8
Med- Surg ICU	1/14/2023 13:00	12	13.0	11.2	-1.8
Med- Surg ICU	1/14/2023 14:00	10	13.0	11.2	-1.8
Med- Surg ICU	1/14/2023 15:00	10	13.0	11.2	-1.8
Med- Surg ICU	1/14/2023 16:00	10	13.0	10.6	-2.4
Med- Surg ICU	1/14/2023 17:00	10	13.0	10.6	-2.4
Med- Surg ICU	1/14/2023 18:00	10	13.0	10.6	-2.4
Med- Surg ICU	1/14/2023 19:00	10	13.0	10.6	-2.4
Med- Surg ICU	1/14/2023 20:00	10	13.0	11.5	-1.5
Med- Surg ICU	1/14/2023 21:00	10	13.0	10.3	-2.7
Med- Surg ICU	1/14/2023 22:00	10	13.0	10.3	-2.7
Med- Surg ICU	1/14/2023 23:00	10	13.0	10.3	-2.7
Med- Surg ICU	1/15/2023 0:00	10	13.0	10.3	-2.7
Med- Surg ICU	1/15/2023 1:00	10	13.0	10.3	-2.7
Med- Surg ICU	1/15/2023 2:00	10	13.0	10.3	-2.7
Med- Surg ICU	1/15/2023 3:00	10	13.0	10.3	-2.7
Med- Surg ICU	1/15/2023 4:00	10	13.0	10.3	-2.7
Med- Surg ICU	1/15/2023 5:00	10	13.0	10.3	-2.7
Med- Surg ICU	1/15/2023 6:00	10	13.0	10.3	-2.7
Med- Surg ICU	1/15/2023 7:00	10	13.0	10.3	-2.7
Med- Surg ICU	1/15/2023 8:00	10	13.0	10.0	-3.0
Med- Surg ICU	1/15/2023 9:00	10	13.0	10.0	-3.0
Med- Surg ICU	1/15/2023 10:00	10	13.0	10.0	-3.0
Med- Surg ICU	1/15/2023 11:00	10	13.0	10.0	-3.0
Med- Surg ICU	1/15/2023 12:00	10	13.0	10.0	-3.0
Med- Surg ICU	1/15/2023 13:00	10	13.0	10.0	-3.0
Med- Surg ICU	1/15/2023 14:00	10	13.0	10.0	-3.0
Med- Surg ICU	1/15/2023 15:00	10	13.0	10.0	-3.0
Med- Surg ICU	1/15/2023 16:00	10	13.0	9.1	-4.0
Med- Surg ICU	1/15/2023 17:00	10	13.0	9.1	-4.0
Med- Surg ICU	1/15/2023 18:00	10	13.0	9.1	-4.0
Med- Surg ICU	1/15/2023 19:00	9	12.0	9.1	-3.0
Med- Surg ICU	1/15/2023 20:00	9	12.0	9.0	-3.0
Med- Surg ICU	1/15/2023 21:00	9	12.0	9.0	-3.0
Med- Surg ICU	1/15/2023 22:00	9	12.0	9.0	-3.0
Med- Surg ICU	1/15/2023 23:00	9	12.0	9.0	-3.0
Med- Surg ICU	1/16/2023 0:00	9	12.0	9.0	-3.0

Med- Surg ICU	1/16/2023 1:00	9	12.0	9.0	-3.0
Med- Surg ICU	1/16/2023 2:00	9	12.0	9.0	-3.0
Med- Surg ICU	1/16/2023 3:00	9	12.0	9.0	-3.0
Med- Surg ICU	1/16/2023 4:00	9	12.0	9.0	-3.0
Med- Surg ICU	1/16/2023 5:00	9	12.0	9.0	-3.0
Med- Surg ICU	1/16/2023 6:00	9	12.0	9.0	-3.0
Med- Surg ICU	1/16/2023 7:00	9	12.0	9.0	-3.0
Med- Surg ICU	1/16/2023 8:00	9	12.0	10.6	-1.4
Med- Surg ICU	1/16/2023 9:00	9	12.0	10.0	-2.0
Med- Surg ICU	1/16/2023 10:00	9	12.0	10.0	-2.0
Med- Surg ICU	1/16/2023 11:00	9	12.0	10.0	-2.0
Med- Surg ICU	1/16/2023 12:00	9	12.0	10.0	-2.0
Med- Surg ICU	1/16/2023 13:00	9	12.0	10.0	-2.0
Med- Surg ICU	1/16/2023 14:00	9	12.0	10.0	-2.0
Med- Surg ICU	1/16/2023 15:00	9	12.0	10.0	-2.0
Med- Surg ICU	1/16/2023 16:00	9	12.0	10.3	-1.7
Med- Surg ICU	1/16/2023 17:00	9	12.0	10.3	-1.7
Med- Surg ICU	1/16/2023 18:00	9	12.0	10.3	-1.7
Med- Surg ICU	1/16/2023 19:00	9	12.0	10.3	-1.7
Med- Surg ICU	1/16/2023 20:00	9	13.0	10.3	-2.7
Med- Surg ICU	1/16/2023 21:00	9	13.0	10.3	-2.7
Med- Surg ICU	1/16/2023 22:00	9	13.0	10.3	-2.7
Med- Surg ICU	1/16/2023 23:00	9	13.0	10.3	-2.7
Med- Surg ICU	1/17/2023 0:00	9	13.0	9.0	-4.0
Med- Surg ICU	1/17/2023 1:00	9	13.0	9.0	-4.0
Med- Surg ICU	1/17/2023 2:00	9	13.0	9.0	-4.0
Med- Surg ICU	1/17/2023 3:00	9	13.0	9.0	-4.0
Med- Surg ICU	1/17/2023 4:00	9	13.0	9.0	-4.0
Med- Surg ICU	1/17/2023 5:00	9	13.0	9.0	-4.0
Med- Surg ICU	1/17/2023 6:00	9	13.0	9.0	-4.0
Med- Surg ICU	1/17/2023 7:00	9	13.0	9.0	-4.0
Med- Surg ICU	1/17/2023 8:00	9	12.0	10.0	-2.0
Med- Surg ICU	1/17/2023 9:00	9	12.0	10.0	-2.0
Med- Surg ICU	1/17/2023 10:00	9	12.0	10.0	-2.0
Med- Surg ICU	1/17/2023 11:00	9	12.0	10.0	-2.0
Med- Surg ICU	1/17/2023 12:00	10	12.0	10.0	-2.0
Med- Surg ICU	1/17/2023 13:00	10	12.0	9.0	-3.0
Med- Surg ICU	1/17/2023 14:00	10	12.0	10.0	-2.0
Med- Surg ICU	1/17/2023 15:00	10	12.0	10.0	-2.0
Med- Surg ICU	1/17/2023 16:00	10	12.0	9.0	-3.0
Med- Surg ICU	1/17/2023 17:00	10	12.0	9.0	-3.0
Med- Surg ICU	1/17/2023 18:00	10	12.0	9.0	-3.0
Med- Surg ICU	1/17/2023 19:00	10	12.0	9.0	-3.0
Med- Surg ICU	1/17/2023 20:00	10	12.0	10.3	-1.7
Med- Surg ICU	1/17/2023 21:00	10	12.0	10.3	-1.7
Med- Surg ICU	1/17/2023 22:00	10	12.0	10.3	-1.7
Med- Surg ICU	1/17/2023 23:00	9	12.0	10.3	-1.7

Med- Surg ICU	1/18/2023 0:00	9	12.0	11.5	-0.5
Med- Surg ICU	1/18/2023 1:00	9	12.0	11.5	-0.5
Med- Surg ICU	1/18/2023 2:00	9	12.0	11.5	-0.5
Med- Surg ICU	1/18/2023 3:00	9	12.0	11.5	-0.5
Med- Surg ICU	1/18/2023 4:00	9	12.0	11.5	-0.5
Med- Surg ICU	1/18/2023 5:00	9	12.0	11.5	-0.5
Med- Surg ICU	1/18/2023 6:00	10	13.0	12.8	-0.2
Med- Surg ICU	1/18/2023 7:00	10	13.0	12.8	-0.2
Med- Surg ICU	1/18/2023 8:00	10	13.0	12.5	-0.6
Med- Surg ICU	1/18/2023 9:00	10	13.0	12.5	-0.6
Med- Surg ICU	1/18/2023 10:00	10	13.0	12.5	-0.6
Med- Surg ICU	1/18/2023 11:00	10	13.0	12.5	-0.6
Med- Surg ICU	1/18/2023 12:00	10	13.0	12.5	-0.6
Med- Surg ICU	1/18/2023 13:00	10	13.0	12.5	-0.6
Med- Surg ICU	1/18/2023 14:00	10	13.0	12.5	-0.6
Med- Surg ICU	1/18/2023 15:00	11	12.0	11.2	-0.8
Med- Surg ICU	1/18/2023 16:00	11	12.0	9.8	-2.2
Med- Surg ICU	1/18/2023 17:00	11	12.0	10.6	-1.4
Med- Surg ICU	1/18/2023 18:00	11	12.0	10.6	-1.4
Med- Surg ICU	1/18/2023 19:00	11	12.0	10.6	-1.4
Med- Surg ICU	1/18/2023 20:00	11	12.0	10.3	-1.7
Med- Surg ICU	1/18/2023 21:00	11	12.0	10.3	-1.7
Med- Surg ICU	1/18/2023 22:00	11	12.0	10.3	-1.7
Med- Surg ICU	1/18/2023 23:00	11	12.0	10.3	-1.7
Med- Surg ICU	1/19/2023 0:00	11	12.0	9.0	-3.0
Med- Surg ICU	1/19/2023 1:00	11	12.0	9.0	-3.0
Med- Surg ICU	1/19/2023 2:00	11	12.0	9.0	-3.0
Med- Surg ICU	1/19/2023 3:00	11	12.0	9.0	-3.0
Med- Surg ICU	1/19/2023 4:00	11	12.0	9.0	-3.0
Med- Surg ICU	1/19/2023 5:00	11	12.0	9.0	-3.0
Med- Surg ICU	1/19/2023 6:00	11	12.0	9.0	-3.0
Med- Surg ICU	1/19/2023 7:00	11	12.0	9.0	-3.0
Med- Surg ICU	1/19/2023 8:00	11	12.0	10.0	-2.0
Med- Surg ICU	1/19/2023 9:00	11	12.0	10.0	-2.0
Med- Surg ICU	1/19/2023 10:00	11	12.0	10.0	-2.0
Med- Surg ICU	1/19/2023 11:00	11	12.0	10.0	-2.0
Med- Surg ICU	1/19/2023 12:00	11	12.0	10.0	-2.0
Med- Surg ICU	1/19/2023 13:00	11	12.0	10.0	-2.0
Med- Surg ICU	1/19/2023 14:00	11	12.0	10.0	-2.0
Med- Surg ICU	1/19/2023 15:00	11	12.0	10.0	-2.0
Med- Surg ICU	1/19/2023 16:00	11	12.0	11.5	-0.5
Med- Surg ICU	1/19/2023 17:00	11	12.0	11.5	-0.5
Med- Surg ICU	1/19/2023 18:00	11	12.0	11.5	-0.5
Med- Surg ICU	1/19/2023 19:00	11	12.0	11.5	-0.5
Med- Surg ICU	1/19/2023 20:00	9	12.0	9.0	-3.0
Med- Surg ICU	1/19/2023 21:00	9	12.0	9.0	-3.0
Med- Surg ICU	1/19/2023 22:00	8	12.0	9.0	-3.0

Med- Surg ICU	1/19/2023 23:00	8	12.0	9.0	-3.0
Med- Surg ICU	1/20/2023 0:00	8	12.0	7.7	-4.3
Med- Surg ICU	1/20/2023 1:00	8	12.0	7.7	-4.3
Med- Surg ICU	1/20/2023 2:00	8	12.0	7.7	-4.3
Med- Surg ICU	1/20/2023 3:00	8	12.0	7.7	-4.3
Med- Surg ICU	1/20/2023 4:00	8	12.0	7.7	-4.3
Med- Surg ICU	1/20/2023 5:00	8	12.0	7.7	-4.3
Med- Surg ICU	1/20/2023 6:00	8	12.0	7.7	-4.3
Med- Surg ICU	1/20/2023 7:00	8	12.0	7.7	-4.3
Med- Surg ICU	1/20/2023 8:00	8	12.0	7.5	-4.5
Med- Surg ICU	1/20/2023 9:00	8	12.0	7.5	-4.5
Med- Surg ICU	1/20/2023 10:00	8	12.0	7.5	-4.5
Med- Surg ICU	1/20/2023 11:00	8	12.0	7.5	-4.5
Med- Surg ICU	1/20/2023 12:00	8	12.0	7.5	-4.5
Med- Surg ICU	1/20/2023 13:00	8	12.0	7.5	-4.5
Med- Surg ICU	1/20/2023 14:00	8	12.0	7.5	-4.5
Med- Surg ICU	1/20/2023 15:00	8	12.0	7.5	-4.5
Med- Surg ICU	1/20/2023 16:00	8	12.0	5.8	-6.2
Med- Surg ICU	1/20/2023 17:00	7	12.0	7.7	-4.3
Med- Surg ICU	1/20/2023 18:00	7	12.0	7.7	-4.3
Med- Surg ICU	1/20/2023 19:00	7	12.0	7.7	-4.3
Med- Surg ICU	1/20/2023 20:00	8	13.0	7.7	-5.3
Med- Surg ICU	1/20/2023 21:00	8	13.0	7.7	-5.3
Med- Surg ICU	1/20/2023 22:00	8	13.0	7.7	-5.3
Med- Surg ICU	1/20/2023 23:00	8	13.0	7.7	-5.3
Med- Surg ICU	1/21/2023 0:00	8	13.0	7.7	-5.3
Med- Surg ICU	1/21/2023 1:00	8	13.0	7.7	-5.3
Med- Surg ICU	1/21/2023 2:00	8	13.0	7.7	-5.3
Med- Surg ICU	1/21/2023 3:00	8	13.0	7.7	-5.3
Med- Surg ICU	1/21/2023 4:00	8	13.0	7.7	-5.3
Med- Surg ICU	1/21/2023 5:00	8	13.0	7.7	-5.3
Med- Surg ICU	1/21/2023 6:00	8	13.0	7.7	-5.3
Med- Surg ICU	1/21/2023 7:00	8	13.0	7.7	-5.3
Med- Surg ICU	1/21/2023 8:00	8	13.0	6.2	-6.8
Med- Surg ICU	1/21/2023 9:00	8	13.0	6.2	-6.8
Med- Surg ICU	1/21/2023 10:00	8	13.0	6.2	-6.8
Med- Surg ICU	1/21/2023 11:00	8	13.0	6.2	-6.8
Med- Surg ICU	1/21/2023 12:00	8	13.0	7.5	-5.5
Med- Surg ICU	1/21/2023 13:00	8	13.0	7.5	-5.5
Med- Surg ICU	1/21/2023 14:00	8	13.0	7.5	-5.5
Med- Surg ICU	1/21/2023 15:00	8	13.0	7.5	-5.5
Med- Surg ICU	1/21/2023 16:00	8	13.0	7.7	-5.3
Med- Surg ICU	1/21/2023 17:00	8	13.0	7.7	-5.3
Med- Surg ICU	1/21/2023 18:00	8	13.0	7.7	-5.3
Med- Surg ICU	1/21/2023 19:00	5	10.0	6.4	-3.6
Med- Surg ICU	1/21/2023 20:00	5	9.0	5.1	-3.9
Med- Surg ICU	1/21/2023 21:00	5	9.0	5.1	-3.9

Med- Surg ICU	1/21/2023 22:00	5	9.0	5.1	-3.9
Med- Surg ICU	1/21/2023 23:00	5	9.0	5.1	-3.9
Med- Surg ICU	1/22/2023 0:00	5	9.0	5.1	-3.9
Med- Surg ICU	1/22/2023 1:00	5	9.0	5.1	-3.9
Med- Surg ICU	1/22/2023 2:00	5	9.0	5.1	-3.9
Med- Surg ICU	1/22/2023 3:00	5	9.0	5.1	-3.9
Med- Surg ICU	1/22/2023 4:00	5	9.0	5.1	-3.9
Med- Surg ICU	1/22/2023 5:00	5	9.0	5.1	-3.9
Med- Surg ICU	1/22/2023 6:00	5	9.0	5.1	-3.9
Med- Surg ICU	1/22/2023 7:00	5	9.0	5.1	-3.9
Med- Surg ICU	1/22/2023 8:00	5	13.0	5.0	-8.0
Med- Surg ICU	1/22/2023 9:00	5	13.0	5.0	-8.0
Med- Surg ICU	1/22/2023 10:00	5	13.0	5.0	-8.0
Med- Surg ICU	1/22/2023 11:00	5	13.0	5.0	-8.0
Med- Surg ICU	1/22/2023 12:00	5	13.0	5.0	-8.0
Med- Surg ICU	1/22/2023 13:00	5	13.0	5.0	-8.0
Med- Surg ICU	1/22/2023 14:00	5	13.0	5.0	-8.0
Med- Surg ICU	1/22/2023 15:00	5	13.0	5.0	-8.0
Med- Surg ICU	1/22/2023 16:00	5	13.0	5.1	-7.9
Med- Surg ICU	1/22/2023 17:00	5	13.0	5.1	-7.9
Med- Surg ICU	1/22/2023 18:00	5	13.0	5.1	-7.9
Med- Surg ICU	1/22/2023 19:00	6	13.0	3.9	-9.2
Med- Surg ICU	1/22/2023 20:00	6	13.0	3.9	-9.2
Med- Surg ICU	1/22/2023 21:00	6	13.0	3.9	-9.2
Med- Surg ICU	1/22/2023 22:00	6	13.0	3.9	-9.2
Med- Surg ICU	1/22/2023 23:00	6	13.0	3.9	-9.2
Med- Surg ICU	1/23/2023 0:00	6	13.0	3.9	-9.2
Med- Surg ICU	1/23/2023 1:00	6	13.0	3.9	-9.2
Med- Surg ICU	1/23/2023 2:00	6	13.0	3.9	-9.2
Med- Surg ICU	1/23/2023 3:00	6	13.0	3.9	-9.2
Med- Surg ICU	1/23/2023 4:00	6	13.0	3.9	-9.2
Med- Surg ICU	1/23/2023 5:00	6	13.0	3.9	-9.2
Med- Surg ICU	1/23/2023 6:00	6	13.0	3.9	-9.2
Med- Surg ICU	1/23/2023 7:00	6	13.0	3.9	-9.2
Med- Surg ICU	1/23/2023 8:00	6	13.0	3.7	-9.3
Med- Surg ICU	1/23/2023 9:00	6	13.0	3.7	-9.3
Med- Surg ICU	1/23/2023 10:00	6	13.0	3.7	-9.3
Med- Surg ICU	1/23/2023 11:00	6	13.0	3.7	-9.3
Med- Surg ICU	1/23/2023 12:00	5	13.0	7.5	-5.5
Med- Surg ICU	1/23/2023 13:00	6	13.0	6.2	-6.8
Med- Surg ICU	1/23/2023 14:00	6	13.0	6.2	-6.8
Med- Surg ICU	1/23/2023 15:00	6	13.0	5.6	-7.4
Med- Surg ICU	1/23/2023 16:00	5	13.0	3.9	-9.2
Med- Surg ICU	1/23/2023 17:00	5	13.0	3.9	-9.2
Med- Surg ICU	1/23/2023 18:00	5	13.0	3.9	-9.2
Med- Surg ICU	1/23/2023 19:00	5	13.0	3.9	-9.2
Med- Surg ICU	1/23/2023 20:00	5	8.0	5.1	-2.9

Med- Surg ICU	1/23/2023 21:00	5	8.0	5.1	-2.9
Med- Surg ICU	1/23/2023 22:00	5	8.0	5.1	-2.9
Med- Surg ICU	1/23/2023 23:00	5	8.0	5.1	-2.9
Med- Surg ICU	1/24/2023 0:00	5	8.0	5.1	-2.9
Med- Surg ICU	1/24/2023 1:00	5	8.0	5.1	-2.9
Med- Surg ICU	1/24/2023 2:00	5	8.0	5.1	-2.9
Med- Surg ICU	1/24/2023 3:00	5	8.0	5.1	-2.9
Med- Surg ICU	1/24/2023 4:00	5	8.0	5.1	-2.9
Med- Surg ICU	1/24/2023 5:00	5	8.0	5.1	-2.9
Med- Surg ICU	1/24/2023 6:00	5	8.0	5.1	-2.9
Med- Surg ICU	1/24/2023 7:00	5	8.0	5.1	-2.9
Med- Surg ICU	1/24/2023 8:00	10	6.0	7.5	1.5
Med- Surg ICU	1/24/2023 9:00	10	6.0	7.5	1.5
Med- Surg ICU	1/24/2023 10:00	10	6.0	7.5	1.5
Med- Surg ICU	1/24/2023 11:00	10	6.0	7.5	1.5
Med- Surg ICU	1/24/2023 12:00	10	6.0	7.5	1.5
Med- Surg ICU	1/24/2023 13:00	10	6.0	7.5	1.5
Med- Surg ICU	1/24/2023 14:00	10	6.0	7.5	1.5
Med- Surg ICU	1/24/2023 15:00	10	6.0	7.5	1.5
Med- Surg ICU	1/24/2023 16:00	9	10.0	6.4	-3.6
Med- Surg ICU	1/24/2023 17:00	9	10.0	6.4	-3.6
Med- Surg ICU	1/24/2023 18:00	7	10.0	6.4	-3.6
Med- Surg ICU	1/24/2023 19:00	7	10.0	6.4	-3.6
Med- Surg ICU	1/24/2023 20:00	7	10.0	6.4	-3.6
Med- Surg ICU	1/24/2023 21:00	7	10.0	6.4	-3.6
Med- Surg ICU	1/24/2023 22:00	7	10.0	6.4	-3.6
Med- Surg ICU	1/24/2023 23:00	7	10.0	6.4	-3.6
Med- Surg ICU	1/25/2023 0:00	7	10.0	7.7	-2.3
Med- Surg ICU	1/25/2023 1:00	7	10.0	7.7	-2.3
Med- Surg ICU	1/25/2023 2:00	7	10.0	7.7	-2.3
Med- Surg ICU	1/25/2023 3:00	7	10.0	7.7	-2.3
Med- Surg ICU	1/25/2023 4:00	7	10.0	7.7	-2.3
Med- Surg ICU	1/25/2023 5:00	7	10.0	7.7	-2.3
Med- Surg ICU	1/25/2023 6:00	7	10.0	7.7	-2.3
Med- Surg ICU	1/25/2023 7:00	7	10.0	7.7	-2.3
Med- Surg ICU	1/25/2023 8:00	7	9.0	8.7	-0.3
Med- Surg ICU	1/25/2023 9:00	7	9.0	8.7	-0.3
Med- Surg ICU	1/25/2023 10:00	7	9.0	8.7	-0.3
Med- Surg ICU	1/25/2023 11:00	7	9.0	8.7	-0.3
Med- Surg ICU	1/25/2023 12:00	7	9.0	8.7	-0.3
Med- Surg ICU	1/25/2023 13:00	7	9.0	8.7	-0.3
Med- Surg ICU	1/25/2023 14:00	7	9.0	8.7	-0.3
Med- Surg ICU	1/25/2023 15:00	7	8.0	8.7	0.7
Med- Surg ICU	1/25/2023 16:00	7	8.0	7.6	-0.5
Med- Surg ICU	1/25/2023 17:00	7	8.0	7.6	-0.5
Med- Surg ICU	1/25/2023 18:00	7	8.0	7.6	-0.5
Med- Surg ICU	1/25/2023 19:00	7	8.0	7.6	-0.5

Med- Surg ICU	1/25/2023 20:00	8	11.0	7.7	-3.3
Med- Surg ICU	1/25/2023 21:00	8	11.0	7.7	-3.3
Med- Surg ICU	1/25/2023 22:00	8	11.0	7.7	-3.3
Med- Surg ICU	1/25/2023 23:00	8	11.0	7.7	-3.3
Med- Surg ICU	1/26/2023 0:00	8	11.0	7.7	-3.3
Med- Surg ICU	1/26/2023 1:00	8	11.0	7.7	-3.3
Med- Surg ICU	1/26/2023 2:00	8	11.0	7.7	-3.3
Med- Surg ICU	1/26/2023 3:00	8	11.0	7.7	-3.3
Med- Surg ICU	1/26/2023 4:00	8	11.0	7.7	-3.3
Med- Surg ICU	1/26/2023 5:00	8	11.0	7.7	-3.3
Med- Surg ICU	1/26/2023 6:00	8	11.0	7.7	-3.3
Med- Surg ICU	1/26/2023 7:00	8	11.0	7.7	-3.3
Med- Surg ICU	1/26/2023 8:00	7	13.0	6.9	-6.2
Med- Surg ICU	1/26/2023 9:00	7	13.0	7.5	-5.5
Med- Surg ICU	1/26/2023 10:00	7	13.0	7.5	-5.5
Med- Surg ICU	1/26/2023 11:00	7	13.0	7.5	-5.5
Med- Surg ICU	1/26/2023 12:00	7	13.0	7.5	-5.5
Med- Surg ICU	1/26/2023 13:00	7	13.0	7.5	-5.5
Med- Surg ICU	1/26/2023 14:00	7	13.0	7.5	-5.5
Med- Surg ICU	1/26/2023 15:00	7	13.0	7.5	-5.5
Med- Surg ICU	1/26/2023 16:00	7	13.0	6.4	-6.6
Med- Surg ICU	1/26/2023 17:00	7	13.0	6.4	-6.6
Med- Surg ICU	1/26/2023 18:00	7	13.0	5.8	-7.2
Med- Surg ICU	1/26/2023 19:00	7	13.0	5.1	-7.9
Med- Surg ICU	1/26/2023 20:00	9	13.0	9.0	-4.0
Med- Surg ICU	1/26/2023 21:00	9	13.0	9.0	-4.0
Med- Surg ICU	1/26/2023 22:00	9	13.0	9.0	-4.0
Med- Surg ICU	1/26/2023 23:00	9	13.0	9.0	-4.0
Med- Surg ICU	1/27/2023 0:00	9	13.0	10.3	-2.7
Med- Surg ICU	1/27/2023 1:00	9	13.0	10.3	-2.7
Med- Surg ICU	1/27/2023 2:00	9	13.0	10.3	-2.7
Med- Surg ICU	1/27/2023 3:00	9	13.0	10.3	-2.7
Med- Surg ICU	1/27/2023 4:00	9	13.0	7.7	-5.3
Med- Surg ICU	1/27/2023 5:00	9	13.0	7.7	-5.3
Med- Surg ICU	1/27/2023 6:00	9	13.0	7.7	-5.3
Med- Surg ICU	1/27/2023 7:00	9	13.0	7.7	-5.3
Med- Surg ICU	1/27/2023 8:00	10	13.0	8.7	-4.3
Med- Surg ICU	1/27/2023 9:00	10	13.0	8.7	-4.3
Med- Surg ICU	1/27/2023 10:00	10	13.0	8.7	-4.3
Med- Surg ICU	1/27/2023 11:00	10	13.0	8.7	-4.3
Med- Surg ICU	1/27/2023 12:00	10	13.0	8.7	-4.3
Med- Surg ICU	1/27/2023 13:00	12	13.0	8.7	-4.3
Med- Surg ICU	1/27/2023 14:00	12	13.0	8.7	-4.3
Med- Surg ICU	1/27/2023 15:00	11	13.0	8.7	-4.3
Med- Surg ICU	1/27/2023 16:00	11	13.0	10.3	-2.7
Med- Surg ICU	1/27/2023 17:00	10	13.0	10.3	-2.7
Med- Surg ICU	1/27/2023 18:00	10	13.0	10.3	-2.7

Med- Surg ICU	1/27/2023 19:00	11	13.0	11.5	-1.5
Med- Surg ICU	1/27/2023 20:00	10	13.0	10.3	-2.7
Med- Surg ICU	1/27/2023 21:00	9	13.0	10.3	-2.7
Med- Surg ICU	1/27/2023 22:00	9	13.0	10.3	-2.7
Med- Surg ICU	1/27/2023 23:00	9	13.0	10.3	-2.7
Med- Surg ICU	1/28/2023 0:00	9	13.0	10.3	-2.7
Med- Surg ICU	1/28/2023 1:00	9	13.0	10.3	-2.7
Med- Surg ICU	1/28/2023 2:00	9	13.0	10.3	-2.7
Med- Surg ICU	1/28/2023 3:00	9	13.0	10.3	-2.7
Med- Surg ICU	1/28/2023 4:00	9	13.0	10.3	-2.7
Med- Surg ICU	1/28/2023 5:00	9	13.0	10.3	-2.7
Med- Surg ICU	1/28/2023 6:00	9	13.0	10.3	-2.7
Med- Surg ICU	1/28/2023 7:00	9	13.0	10.3	-2.7
Med- Surg ICU	1/28/2023 8:00	9	13.0	10.0	-3.0
Med- Surg ICU	1/28/2023 9:00	9	13.0	10.0	-3.0
Med- Surg ICU	1/28/2023 10:00	9	13.0	10.0	-3.0
Med- Surg ICU	1/28/2023 11:00	8	13.0	10.0	-3.0
Med- Surg ICU	1/28/2023 12:00	8	13.0	10.0	-3.0
Med- Surg ICU	1/28/2023 13:00	8	13.0	10.0	-3.0
Med- Surg ICU	1/28/2023 14:00	7	13.0	10.0	-3.0
Med- Surg ICU	1/28/2023 15:00	7	13.0	10.0	-3.0
Med- Surg ICU	1/28/2023 16:00	7	13.0	9.0	-4.0
Med- Surg ICU	1/28/2023 17:00	7	13.0	9.0	-4.0
Med- Surg ICU	1/28/2023 18:00	7	13.0	9.0	-4.0
Med- Surg ICU	1/28/2023 19:00	7	10.0	10.3	0.3
Med- Surg ICU	1/28/2023 20:00	7	10.0	9.0	-1.0
Med- Surg ICU	1/28/2023 21:00	7	10.0	9.0	-1.0
Med- Surg ICU	1/28/2023 22:00	7	10.0	9.0	-1.0
Med- Surg ICU	1/28/2023 23:00	7	10.0	9.0	-1.0
Med- Surg ICU	1/29/2023 0:00	7	10.0	9.0	-1.0
Med- Surg ICU	1/29/2023 1:00	7	10.0	9.0	-1.0
Med- Surg ICU	1/29/2023 2:00	7	10.0	9.0	-1.0
Med- Surg ICU	1/29/2023 3:00	7	10.0	9.0	-1.0
Med- Surg ICU	1/29/2023 4:00	7	10.0	9.0	-1.0
Med- Surg ICU	1/29/2023 5:00	7	10.0	9.0	-1.0
Med- Surg ICU	1/29/2023 6:00	7	10.0	9.0	-1.0
Med- Surg ICU	1/29/2023 7:00	7	10.0	9.0	-1.0
Med- Surg ICU	1/29/2023 8:00	7	10.0	10.0	0.0
Med- Surg ICU	1/29/2023 9:00	7	10.0	10.0	0.0
Med- Surg ICU	1/29/2023 10:00	7	10.0	8.7	-1.3
Med- Surg ICU	1/29/2023 11:00	7	10.0	8.7	-1.3
Med- Surg ICU	1/29/2023 12:00	7	10.0	8.7	-1.3
Med- Surg ICU	1/29/2023 13:00	7	10.0	8.7	-1.3
Med- Surg ICU	1/29/2023 14:00	7	10.0	8.7	-1.3
Med- Surg ICU	1/29/2023 15:00	7	10.0	8.7	-1.3
Med- Surg ICU	1/29/2023 16:00	7	10.0	10.3	0.3
Med- Surg ICU	1/29/2023 17:00	7	10.0	10.3	0.3

Med- Surg ICU	1/29/2023 18:00	7	10.0	10.3	0.3
Med- Surg ICU	1/29/2023 19:00	7	10.0	10.3	0.3
Med- Surg ICU	1/29/2023 20:00	7	10.0	5.1	-4.9
Med- Surg ICU	1/29/2023 21:00	7	10.0	5.1	-4.9
Med- Surg ICU	1/29/2023 22:00	7	10.0	5.1	-4.9
Med- Surg ICU	1/29/2023 23:00	7	10.0	5.1	-4.9
Med- Surg ICU	1/30/2023 0:00	7	10.0	3.9	-6.2
Med- Surg ICU	1/30/2023 1:00	7	10.0	3.9	-6.2
Med- Surg ICU	1/30/2023 2:00	7	10.0	3.9	-6.2
Med- Surg ICU	1/30/2023 3:00	7	10.0	3.9	-6.2
Med- Surg ICU	1/30/2023 4:00	7	10.0	3.9	-6.2
Med- Surg ICU	1/30/2023 5:00	7	10.0	3.9	-6.2
Med- Surg ICU	1/30/2023 6:00	7	10.0	3.9	-6.2
Med- Surg ICU	1/30/2023 7:00	7	10.0	3.9	-6.2
Med- Surg ICU	1/30/2023 8:00	7	12.0	10.0	-2.0
Med- Surg ICU	1/30/2023 9:00	7	12.0	10.0	-2.0
Med- Surg ICU	1/30/2023 10:00	7	12.0	10.0	-2.0
Med- Surg ICU	1/30/2023 11:00	7	12.0	10.0	-2.0
Med- Surg ICU	1/30/2023 12:00	7	12.0	10.0	-2.0
Med- Surg ICU	1/30/2023 13:00	7	12.0	10.0	-2.0
Med- Surg ICU	1/30/2023 14:00	7	12.0	10.0	-2.0
Med- Surg ICU	1/30/2023 15:00	11	12.0	7.5	-4.5
Med- Surg ICU	1/30/2023 16:00	11	12.0	8.3	-3.7
Med- Surg ICU	1/30/2023 17:00	11	12.0	9.1	-3.0
Med- Surg ICU	1/30/2023 18:00	11	12.0	9.1	-3.0
Med- Surg ICU	1/30/2023 19:00	11	12.0	9.1	-3.0
Med- Surg ICU	1/30/2023 20:00	12	12.0	7.7	-4.3
Med- Surg ICU	1/30/2023 21:00	12	12.0	7.7	-4.3
Med- Surg ICU	1/30/2023 22:00	12	12.0	7.7	-4.3
Med- Surg ICU	1/30/2023 23:00	12	12.0	7.7	-4.3
Med- Surg ICU	1/31/2023 0:00	12	12.0	5.1	-6.9
Med- Surg ICU	1/31/2023 1:00	12	12.0	5.1	-6.9
Med- Surg ICU	1/31/2023 2:00	12	12.0	5.1	-6.9
Med- Surg ICU	1/31/2023 3:00	12	12.0	5.1	-6.9
Med- Surg ICU	1/31/2023 4:00	12	12.0	5.1	-6.9
Med- Surg ICU	1/31/2023 5:00	12	12.0	5.1	-6.9
Med- Surg ICU	1/31/2023 6:00	12	12.0	5.1	-6.9
Med- Surg ICU	1/31/2023 7:00	12	12.0	5.1	-6.9
Med- Surg ICU	1/31/2023 8:00	13	13.0	12.5	-0.6
Med- Surg ICU	1/31/2023 9:00	13	13.0	12.5	-0.6
Med- Surg ICU	1/31/2023 10:00	13	13.0	12.5	-0.6
Med- Surg ICU	1/31/2023 11:00	13	13.0	12.5	-0.6
Med- Surg ICU	1/31/2023 12:00	13	13.0	12.5	-0.6
Med- Surg ICU	1/31/2023 13:00	13	13.0	12.5	-0.6
Med- Surg ICU	1/31/2023 14:00	13	13.0	12.5	-0.6
Med- Surg ICU	1/31/2023 15:00	13	13.0	12.5	-0.6
Med- Surg ICU	1/31/2023 16:00	13	13.0	10.3	-2.7

Med- Surg ICU	1/31/2023 17:00	13	13.0	10.3	-2.7
Med- Surg ICU	1/31/2023 18:00	13	13.0	10.3	-2.7
Med- Surg ICU	1/31/2023 19:00	13	13.0	10.3	-2.7
Med- Surg ICU	1/31/2023 20:00	13	13.0	9.0	-4.0
Med- Surg ICU	1/31/2023 21:00	13	13.0	9.0	-4.0
Med- Surg ICU	1/31/2023 22:00	13	13.0	9.0	-4.0
Med- Surg ICU	1/31/2023 23:00	13	13.0	9.0	-4.0
Med- Surg ICU	2/1/2023 0:00	11	13.0	10.9	-2.1
Med- Surg ICU	2/1/2023 1:00	11	13.0	11.5	-1.5
Med- Surg ICU	2/1/2023 2:00	11	13.0	11.5	-1.5
Med- Surg ICU	2/1/2023 3:00	11	13.0	11.5	-1.5
Med- Surg ICU	2/1/2023 4:00	11	13.0	11.5	-1.5
Med- Surg ICU	2/1/2023 5:00	11	13.0	11.5	-1.5
Med- Surg ICU	2/1/2023 6:00	11	13.0	11.5	-1.5
Med- Surg ICU	2/1/2023 7:00	11	13.0	11.5	-1.5
Med- Surg ICU	2/1/2023 8:00	11	13.0	12.5	-0.6
Med- Surg ICU	2/1/2023 9:00	11	13.0	12.5	-0.6
Med- Surg ICU	2/1/2023 10:00	11	13.0	12.5	-0.6
Med- Surg ICU	2/1/2023 11:00	11	13.0	12.5	-0.6
Med- Surg ICU	2/1/2023 12:00	11	13.0	12.5	-0.6
Med- Surg ICU	2/1/2023 13:00	11	13.0	12.5	-0.6
Med- Surg ICU	2/1/2023 14:00	11	13.0	12.5	-0.6
Med- Surg ICU	2/1/2023 15:00	12	13.0	12.5	-0.6
Med- Surg ICU	2/1/2023 16:00	12	13.0	12.1	-0.9
Med- Surg ICU	2/1/2023 17:00	12	13.0	12.1	-0.9
Med- Surg ICU	2/1/2023 18:00	12	13.0	12.1	-0.9
Med- Surg ICU	2/1/2023 19:00	12	13.0	12.1	-0.9
Med- Surg ICU	2/1/2023 20:00	12	13.0	11.5	-1.5
Med- Surg ICU	2/1/2023 21:00	11	13.0	11.5	-1.5
Med- Surg ICU	2/1/2023 22:00	11	13.0	11.5	-1.5
Med- Surg ICU	2/1/2023 23:00	11	13.0	11.5	-1.5
Med- Surg ICU	2/2/2023 0:00	11	13.0	11.5	-1.5
Med- Surg ICU	2/2/2023 1:00	11	13.0	11.5	-1.5
Med- Surg ICU	2/2/2023 2:00	11	13.0	11.5	-1.5
Med- Surg ICU	2/2/2023 3:00	11	13.0	11.5	-1.5
Med- Surg ICU	2/2/2023 4:00	11	13.0	11.5	-1.5
Med- Surg ICU	2/2/2023 5:00	11	13.0	11.5	-1.5
Med- Surg ICU	2/2/2023 6:00	11	13.0	11.5	-1.5
Med- Surg ICU	2/2/2023 7:00	11	13.0	11.5	-1.5
Med- Surg ICU	2/2/2023 8:00	11	13.0	14.9	1.9
Med- Surg ICU	2/2/2023 9:00	11	13.0	14.9	1.9
Med- Surg ICU	2/2/2023 10:00	11	13.0	14.9	1.9
Med- Surg ICU	2/2/2023 11:00	11	13.0	14.9	1.9
Med- Surg ICU	2/2/2023 12:00	11	13.0	14.9	1.9
Med- Surg ICU	2/2/2023 13:00	11	13.0	14.9	1.9
Med- Surg ICU	2/2/2023 14:00	11	13.0	14.9	1.9
Med- Surg ICU	2/2/2023 15:00	11	13.0	14.9	1.9

Med- Surg ICU	2/2/2023 16:00	11	13.0	9.0	-4.0
Med- Surg ICU	2/2/2023 17:00	11	13.0	9.0	-4.0
Med- Surg ICU	2/2/2023 18:00	11	13.0	9.0	-4.0
Med- Surg ICU	2/2/2023 19:00	11	13.0	9.0	-4.0
Med- Surg ICU	2/2/2023 20:00	11	12.0	6.4	-5.6
Med- Surg ICU	2/2/2023 21:00	11	12.0	6.4	-5.6
Med- Surg ICU	2/2/2023 22:00	11	12.0	6.4	-5.6
Med- Surg ICU	2/2/2023 23:00	11	12.0	6.4	-5.6
Med- Surg ICU	2/3/2023 0:00	11	12.0	7.7	-4.3
Med- Surg ICU	2/3/2023 1:00	11	12.0	7.7	-4.3
Med- Surg ICU	2/3/2023 2:00	11	12.0	7.7	-4.3
Med- Surg ICU	2/3/2023 3:00	11	12.0	7.7	-4.3
Med- Surg ICU	2/3/2023 4:00	11	12.0	7.7	-4.3
Med- Surg ICU	2/3/2023 5:00	11	12.0	7.7	-4.3
Med- Surg ICU	2/3/2023 6:00	10	12.0	7.7	-4.3
Med- Surg ICU	2/3/2023 7:00	10	12.0	7.7	-4.3
Med- Surg ICU	2/3/2023 8:00	9	11.0	10.0	-1.0
Med- Surg ICU	2/3/2023 9:00	9	11.0	10.0	-1.0
Med- Surg ICU	2/3/2023 10:00	9	11.0	10.0	-1.0
Med- Surg ICU	2/3/2023 11:00	9	11.0	10.0	-1.0
Med- Surg ICU	2/3/2023 12:00	9	11.0	10.0	-1.0
Med- Surg ICU	2/3/2023 13:00	9	11.0	10.0	-1.0
Med- Surg ICU	2/3/2023 14:00	9	11.0	10.0	-1.0
Med- Surg ICU	2/3/2023 15:00	9	11.0	10.0	-1.0
Med- Surg ICU	2/3/2023 16:00	9	11.0	11.5	0.5
Med- Surg ICU	2/3/2023 17:00	9	11.0	11.5	0.5
Med- Surg ICU	2/3/2023 18:00	9	11.0	11.5	0.5
Med- Surg ICU	2/3/2023 19:00	9	11.0	11.5	0.5
Med- Surg ICU	2/3/2023 20:00	9	13.0	9.0	-4.0
Med- Surg ICU	2/3/2023 21:00	9	13.0	9.0	-4.0
Med- Surg ICU	2/3/2023 22:00	9	13.0	9.0	-4.0
Med- Surg ICU	2/3/2023 23:00	9	13.0	9.0	-4.0
Med- Surg ICU	2/4/2023 0:00	9	13.0	7.7	-5.3
Med- Surg ICU	2/4/2023 1:00	9	13.0	7.7	-5.3
Med- Surg ICU	2/4/2023 2:00	9	13.0	7.7	-5.3
Med- Surg ICU	2/4/2023 3:00	9	13.0	7.7	-5.3
Med- Surg ICU	2/4/2023 4:00	9	13.0	7.7	-5.3
Med- Surg ICU	2/4/2023 5:00	9	13.0	7.7	-5.3
Med- Surg ICU	2/4/2023 6:00	9	13.0	7.7	-5.3
Med- Surg ICU	2/4/2023 7:00	9	13.0	7.7	-5.3
Med- Surg ICU	2/4/2023 8:00	9	13.0	7.5	-5.5
Med- Surg ICU	2/4/2023 9:00	9	13.0	7.5	-5.5
Med- Surg ICU	2/4/2023 10:00	9	13.0	7.5	-5.5
Med- Surg ICU	2/4/2023 11:00	9	13.0	7.5	-5.5
Med- Surg ICU	2/4/2023 12:00	9	13.0	8.7	-4.3
Med- Surg ICU	2/4/2023 13:00	9	13.0	8.7	-4.3
Med- Surg ICU	2/4/2023 14:00	9	13.0	8.7	-4.3

Med- Surg ICU	2/4/2023 15:00	9	13.0	8.7	-4.3
Med- Surg ICU	2/4/2023 16:00	9	13.0	9.0	-4.0
Med- Surg ICU	2/4/2023 17:00	9	13.0	9.0	-4.0
Med- Surg ICU	2/4/2023 18:00	9	13.0	9.0	-4.0
Med- Surg ICU	2/4/2023 19:00	6	11.0	8.3	-2.7
Med- Surg ICU	2/4/2023 20:00	6	11.0	6.4	-4.6
Med- Surg ICU	2/4/2023 21:00	6	11.0	6.4	-4.6
Med- Surg ICU	2/4/2023 22:00	6	11.0	6.4	-4.6
Med- Surg ICU	2/4/2023 23:00	6	11.0	6.4	-4.6
Med- Surg ICU	2/5/2023 0:00	6	11.0	6.4	-4.6
Med- Surg ICU	2/5/2023 1:00	7	11.0	6.4	-4.6
Med- Surg ICU	2/5/2023 2:00	7	11.0	6.4	-4.6
Med- Surg ICU	2/5/2023 3:00	7	11.0	6.4	-4.6
Med- Surg ICU	2/5/2023 4:00	7	11.0	6.4	-4.6
Med- Surg ICU	2/5/2023 5:00	7	11.0	6.4	-4.6
Med- Surg ICU	2/5/2023 6:00	7	11.0	6.4	-4.6
Med- Surg ICU	2/5/2023 7:00	7	11.0	6.4	-4.6
Med- Surg ICU	2/5/2023 8:00	7	11.0	0.0	-11.0
Med- Surg ICU	2/5/2023 9:00	7	11.0	0.0	-11.0
Med- Surg ICU	2/5/2023 10:00	7	11.0	0.0	-11.0
Med- Surg ICU	2/5/2023 11:00	7	11.0	0.0	-11.0
Med- Surg ICU	2/5/2023 12:00	7	11.0	0.0	-11.0
Med- Surg ICU	2/5/2023 13:00	7	11.0	0.0	-11.0
Med- Surg ICU	2/5/2023 14:00	7	11.0	0.0	-11.0
Med- Surg ICU	2/5/2023 15:00	7	11.0	0.0	-11.0
Med- Surg ICU	2/5/2023 16:00	7	11.0	0.0	-11.0
Med- Surg ICU	2/5/2023 17:00	7	11.0	0.0	-11.0
Med- Surg ICU	2/5/2023 18:00	7	11.0	0.0	-11.0
Med- Surg ICU	2/5/2023 19:00	7	11.0	0.0	-11.0
Med- Surg ICU	2/5/2023 20:00	6	9.0	5.1	-3.9
Med- Surg ICU	2/5/2023 21:00	6	9.0	5.1	-3.9
Med- Surg ICU	2/5/2023 22:00	6	9.0	5.1	-3.9
Med- Surg ICU	2/5/2023 23:00	6	9.0	5.1	-3.9
Med- Surg ICU	2/6/2023 0:00	6	9.0	5.1	-3.9
Med- Surg ICU	2/6/2023 1:00	6	9.0	5.1	-3.9
Med- Surg ICU	2/6/2023 2:00	6	9.0	5.1	-3.9
Med- Surg ICU	2/6/2023 3:00	6	9.0	5.1	-3.9
Med- Surg ICU	2/6/2023 4:00	6	9.0	5.1	-3.9
Med- Surg ICU	2/6/2023 5:00	6	9.0	5.1	-3.9
Med- Surg ICU	2/6/2023 6:00	6	9.0	5.1	-3.9
Med- Surg ICU	2/6/2023 7:00	6	9.0	5.1	-3.9
Med- Surg ICU	2/6/2023 8:00	6	9.0	11.2	2.2
Med- Surg ICU	2/6/2023 9:00	6	9.0	11.2	2.2
Med- Surg ICU	2/6/2023 10:00	6	9.0	11.2	2.2
Med- Surg ICU	2/6/2023 11:00	6	9.0	11.2	2.2
Med- Surg ICU	2/6/2023 12:00	6	9.0	11.2	2.2
Med- Surg ICU	2/6/2023 13:00	6	9.0	11.2	2.2

Med- Surg ICU	2/6/2023 14:00	6	9.0	11.2	2.2
Med- Surg ICU	2/6/2023 15:00	6	9.0	11.2	2.2
Med- Surg ICU	2/6/2023 16:00	6	9.0	12.8	3.8
Med- Surg ICU	2/6/2023 17:00	6	9.0	12.8	3.8
Med- Surg ICU	2/6/2023 18:00	6	9.0	12.8	3.8
Med- Surg ICU	2/6/2023 19:00	6	9.0	12.8	3.8
Med- Surg ICU	2/6/2023 20:00	8	12.0	7.7	-4.3
Med- Surg ICU	2/6/2023 21:00	8	12.0	7.7	-4.3
Med- Surg ICU	2/6/2023 22:00	8	12.0	7.7	-4.3
Med- Surg ICU	2/6/2023 23:00	8	12.0	7.7	-4.3
Med- Surg ICU	2/7/2023 0:00	8	12.0	6.4	-5.6
Med- Surg ICU	2/7/2023 1:00	8	12.0	6.4	-5.6
Med- Surg ICU	2/7/2023 2:00	8	12.0	6.4	-5.6
Med- Surg ICU	2/7/2023 3:00	8	12.0	6.4	-5.6
Med- Surg ICU	2/7/2023 4:00	8	12.0	6.4	-5.6
Med- Surg ICU	2/7/2023 5:00	8	12.0	6.4	-5.6
Med- Surg ICU	2/7/2023 6:00	8	12.0	6.4	-5.6
Med- Surg ICU	2/7/2023 7:00	8	12.0	6.4	-5.6
Med- Surg ICU	2/7/2023 8:00	9	13.0	10.0	-3.0
Med- Surg ICU	2/7/2023 9:00	9	13.0	10.0	-3.0
Med- Surg ICU	2/7/2023 10:00	9	13.0	9.0	-4.0
Med- Surg ICU	2/7/2023 11:00	9	13.0	10.0	-3.0
Med- Surg ICU	2/7/2023 12:00	8	13.0	8.1	-4.9
Med- Surg ICU	2/7/2023 13:00	8	13.0	6.5	-6.5
Med- Surg ICU	2/7/2023 14:00	8	13.0	7.5	-5.5
Med- Surg ICU	2/7/2023 15:00	8	13.0	7.5	-5.5
Med- Surg ICU	2/7/2023 16:00	8	13.0	5.5	-7.6
Med- Surg ICU	2/7/2023 17:00	8	13.0	6.4	-6.6
Med- Surg ICU	2/7/2023 18:00	8	13.0	6.4	-6.6
Med- Surg ICU	2/7/2023 19:00	8	13.0	6.4	-6.6
Med- Surg ICU	2/7/2023 20:00	8	13.0	9.0	-4.0
Med- Surg ICU	2/7/2023 21:00	6	13.0	9.0	-4.0
Med- Surg ICU	2/7/2023 22:00	6	13.0	9.0	-4.0
Med- Surg ICU	2/7/2023 23:00	6	13.0	9.0	-4.0
Med- Surg ICU	2/8/2023 0:00	6	13.0	7.7	-5.3
Med- Surg ICU	2/8/2023 1:00	6	13.0	7.7	-5.3
Med- Surg ICU	2/8/2023 2:00	6	13.0	7.7	-5.3
Med- Surg ICU	2/8/2023 3:00	6	13.0	7.7	-5.3
Med- Surg ICU	2/8/2023 4:00	6	13.0	7.7	-5.3
Med- Surg ICU	2/8/2023 5:00	6	13.0	7.7	-5.3
Med- Surg ICU	2/8/2023 6:00	6	13.0	7.7	-5.3
Med- Surg ICU	2/8/2023 7:00	6	10.0	7.7	-2.3
Med- Surg ICU	2/8/2023 8:00	6	10.0	7.5	-2.5
Med- Surg ICU	2/8/2023 9:00	6	10.0	7.5	-2.5
Med- Surg ICU	2/8/2023 10:00	6	10.0	7.5	-2.5
Med- Surg ICU	2/8/2023 11:00	7	10.0	7.5	-2.5
Med- Surg ICU	2/8/2023 12:00	7	10.0	7.5	-2.5

Med- Surg ICU	2/8/2023 13:00	7	10.0	6.5	-3.5
Med- Surg ICU	2/8/2023 14:00	7	10.0	7.5	-2.5
Med- Surg ICU	2/8/2023 15:00	7	10.0	7.5	-2.5
Med- Surg ICU	2/8/2023 16:00	7	10.0	7.6	-2.5
Med- Surg ICU	2/8/2023 17:00	7	10.0	7.6	-2.5
Med- Surg ICU	2/8/2023 18:00	7	10.0	7.6	-2.5
Med- Surg ICU	2/8/2023 19:00	7	10.0	6.4	-3.6
Med- Surg ICU	2/8/2023 20:00	7	10.0	7.7	-2.3
Med- Surg ICU	2/8/2023 21:00	7	10.0	7.7	-2.3
Med- Surg ICU	2/8/2023 22:00	7	10.0	7.7	-2.3
Med- Surg ICU	2/8/2023 23:00	7	10.0	7.7	-2.3
Med- Surg ICU	2/9/2023 0:00	7	10.0	7.7	-2.3
Med- Surg ICU	2/9/2023 1:00	7	10.0	7.7	-2.3
Med- Surg ICU	2/9/2023 2:00	7	10.0	7.7	-2.3
Med- Surg ICU	2/9/2023 3:00	7	10.0	7.7	-2.3
Med- Surg ICU	2/9/2023 4:00	7	10.0	7.7	-2.3
Med- Surg ICU	2/9/2023 5:00	7	10.0	7.7	-2.3
Med- Surg ICU	2/9/2023 6:00	7	10.0	7.7	-2.3
Med- Surg ICU	2/9/2023 7:00	7	13.0	9.0	-4.0
Med- Surg ICU	2/9/2023 8:00	7	13.0	10.0	-3.0
Med- Surg ICU	2/9/2023 9:00	8	13.0	10.0	-3.0
Med- Surg ICU	2/9/2023 10:00	8	13.0	10.0	-3.0
Med- Surg ICU	2/9/2023 11:00	10	13.0	9.0	-4.0
Med- Surg ICU	2/9/2023 12:00	10	13.0	10.0	-3.0
Med- Surg ICU	2/9/2023 13:00	10	13.0	9.0	-4.0
Med- Surg ICU	2/9/2023 14:00	10	13.0	10.0	-3.0
Med- Surg ICU	2/9/2023 15:00	10	13.0	10.0	-3.0
Med- Surg ICU	2/9/2023 16:00	9	10.0	10.3	0.3
Med- Surg ICU	2/9/2023 17:00	9	10.0	10.3	0.3
Med- Surg ICU	2/9/2023 18:00	9	10.0	9.3	-0.7
Med- Surg ICU	2/9/2023 19:00	9	10.0	10.3	0.3
Med- Surg ICU	2/9/2023 20:00	9	10.0	7.7	-2.3
Med- Surg ICU	2/9/2023 21:00	9	10.0	7.7	-2.3
Med- Surg ICU	2/9/2023 22:00	9	10.0	7.7	-2.3
Med- Surg ICU	2/9/2023 23:00	9	10.0	7.7	-2.3
Med- Surg ICU	2/10/2023 0:00	9	10.0	7.7	-2.3
Med- Surg ICU	2/10/2023 1:00	9	10.0	7.7	-2.3
Med- Surg ICU	2/10/2023 2:00	9	10.0	7.7	-2.3
Med- Surg ICU	2/10/2023 3:00	9	10.0	7.7	-2.3
Med- Surg ICU	2/10/2023 4:00	9	10.0	7.7	-2.3
Med- Surg ICU	2/10/2023 5:00	9	10.0	7.7	-2.3
Med- Surg ICU	2/10/2023 6:00	9	10.0	7.7	-2.3
Med- Surg ICU	2/10/2023 7:00	9	13.0	10.3	-2.7
Med- Surg ICU	2/10/2023 8:00	9	13.0	13.7	0.7
Med- Surg ICU	2/10/2023 9:00	9	13.0	13.7	0.7
Med- Surg ICU	2/10/2023 10:00	9	13.0	13.1	0.1
Med- Surg ICU	2/10/2023 11:00	9	13.0	12.5	-0.6

Med- Surg ICU	2/10/2023 12:00	6	13.0	12.5	-0.6
Med- Surg ICU	2/10/2023 13:00	6	13.0	12.5	-0.6
Med- Surg ICU	2/10/2023 14:00	6	13.0	12.5	-0.6
Med- Surg ICU	2/10/2023 15:00	6	13.0	11.8	-1.2
Med- Surg ICU	2/10/2023 16:00	6	13.0	7.7	-5.3
Med- Surg ICU	2/10/2023 17:00	6	13.0	7.7	-5.3
Med- Surg ICU	2/10/2023 18:00	6	13.0	7.7	-5.3
Med- Surg ICU	2/10/2023 19:00	6	13.0	7.7	-5.3
Med- Surg ICU	2/10/2023 20:00	6	11.0	7.7	-3.3
Med- Surg ICU	2/10/2023 21:00	6	11.0	7.7	-3.3
Med- Surg ICU	2/10/2023 22:00	6	11.0	7.7	-3.3
Med- Surg ICU	2/10/2023 23:00	6	11.0	7.7	-3.3
Med- Surg ICU	2/11/2023 0:00	6	11.0	9.0	-2.0
Med- Surg ICU	2/11/2023 1:00	6	11.0	9.0	-2.0
Med- Surg ICU	2/11/2023 2:00	6	11.0	9.0	-2.0
Med- Surg ICU	2/11/2023 3:00	6	11.0	9.0	-2.0
Med- Surg ICU	2/11/2023 4:00	6	11.0	9.0	-2.0
Med- Surg ICU	2/11/2023 5:00	6	11.0	9.0	-2.0
Med- Surg ICU	2/11/2023 6:00	6	11.0	9.0	-2.0
Med- Surg ICU	2/11/2023 7:00	6	11.0	9.0	-2.0
Med- Surg ICU	2/11/2023 8:00	7	11.0	6.2	-4.8
Med- Surg ICU	2/11/2023 9:00	7	11.0	6.2	-4.8
Med- Surg ICU	2/11/2023 10:00	8	11.0	6.2	-4.8
Med- Surg ICU	2/11/2023 11:00	8	11.0	6.2	-4.8
Med- Surg ICU	2/11/2023 12:00	8	11.0	7.5	-3.5
Med- Surg ICU	2/11/2023 13:00	8	11.0	7.5	-3.5
Med- Surg ICU	2/11/2023 14:00	8	11.0	7.5	-3.5
Med- Surg ICU	2/11/2023 15:00	8	11.0	7.5	-3.5
Med- Surg ICU	2/11/2023 16:00	8	11.0	9.0	-2.0
Med- Surg ICU	2/11/2023 17:00	8	11.0	9.0	-2.0
Med- Surg ICU	2/11/2023 18:00	12	8.0	10.6	2.6
Med- Surg ICU	2/11/2023 19:00	12	8.0	10.3	2.3
Med- Surg ICU	2/11/2023 20:00	8	11.0	9.0	-2.0
Med- Surg ICU	2/11/2023 21:00	8	11.0	9.0	-2.0
Med- Surg ICU	2/11/2023 22:00	8	11.0	9.0	-2.0
Med- Surg ICU	2/11/2023 23:00	8	11.0	9.0	-2.0
Med- Surg ICU	2/12/2023 0:00	8	11.0	9.0	-2.0
Med- Surg ICU	2/12/2023 1:00	8	11.0	9.0	-2.0
Med- Surg ICU	2/12/2023 2:00	8	11.0	9.0	-2.0
Med- Surg ICU	2/12/2023 3:00	8	11.0	9.0	-2.0
Med- Surg ICU	2/12/2023 4:00	8	11.0	9.0	-2.0
Med- Surg ICU	2/12/2023 5:00	8	11.0	9.0	-2.0
Med- Surg ICU	2/12/2023 6:00	8	11.0	9.0	-2.0
Med- Surg ICU	2/12/2023 7:00	8	11.0	9.0	-2.0
Med- Surg ICU	2/12/2023 8:00	8	12.0	7.5	-4.5
Med- Surg ICU	2/12/2023 9:00	8	12.0	7.5	-4.5
Med- Surg ICU	2/12/2023 10:00	8	12.0	7.5	-4.5

Med- Surg ICU	2/12/2023 11:00	8	12.0	7.5	-4.5
Med- Surg ICU	2/12/2023 12:00	8	12.0	7.5	-4.5
Med- Surg ICU	2/12/2023 13:00	9	12.0	7.5	-4.5
Med- Surg ICU	2/12/2023 14:00	9	12.0	7.5	-4.5
Med- Surg ICU	2/12/2023 15:00	7	12.0	7.5	-4.5
Med- Surg ICU	2/12/2023 16:00	7	12.0	9.0	-3.0
Med- Surg ICU	2/12/2023 17:00	7	12.0	9.0	-3.0
Med- Surg ICU	2/12/2023 18:00	7	12.0	9.0	-3.0
Med- Surg ICU	2/12/2023 19:00	7	12.0	9.0	-3.0
Med- Surg ICU	2/12/2023 20:00	7	12.0	10.3	-1.7
Med- Surg ICU	2/12/2023 21:00	7	12.0	10.3	-1.7
Med- Surg ICU	2/12/2023 22:00	7	12.0	10.3	-1.7
Med- Surg ICU	2/12/2023 23:00	7	12.0	10.3	-1.7
Med- Surg ICU	2/13/2023 0:00	7	12.0	9.0	-3.0
Med- Surg ICU	2/13/2023 1:00	7	12.0	9.0	-3.0
Med- Surg ICU	2/13/2023 2:00	7	12.0	9.0	-3.0
Med- Surg ICU	2/13/2023 3:00	7	12.0	9.0	-3.0
Med- Surg ICU	2/13/2023 4:00	7	12.0	9.0	-3.0
Med- Surg ICU	2/13/2023 5:00	7	12.0	9.0	-3.0
Med- Surg ICU	2/13/2023 6:00	7	12.0	9.0	-3.0
Med- Surg ICU	2/13/2023 7:00	7	12.0	9.0	-3.0
Med- Surg ICU	2/13/2023 8:00	7	12.0	6.2	-5.8
Med- Surg ICU	2/13/2023 9:00	7	12.0	6.2	-5.8
Med- Surg ICU	2/13/2023 10:00	7	12.0	6.2	-5.8
Med- Surg ICU	2/13/2023 11:00	8	12.0	7.5	-4.5
Med- Surg ICU	2/13/2023 12:00	8	12.0	10.0	-2.0
Med- Surg ICU	2/13/2023 13:00	7	12.0	10.0	-2.0
Med- Surg ICU	2/13/2023 14:00	7	12.0	10.0	-2.0
Med- Surg ICU	2/13/2023 15:00	7	12.0	10.0	-2.0
Med- Surg ICU	2/13/2023 16:00	7	12.0	10.6	-1.4
Med- Surg ICU	2/13/2023 17:00	7	12.0	10.6	-1.4
Med- Surg ICU	2/13/2023 18:00	7	12.0	10.6	-1.4
Med- Surg ICU	2/13/2023 19:00	7	12.0	10.6	-1.4
Med- Surg ICU	2/13/2023 20:00	8	12.0	10.3	-1.7
Med- Surg ICU	2/13/2023 21:00	8	12.0	10.3	-1.7
Med- Surg ICU	2/13/2023 22:00	8	12.0	10.3	-1.7
Med- Surg ICU	2/13/2023 23:00	8	12.0	10.3	-1.7
Med- Surg ICU	2/14/2023 0:00	8	12.0	10.3	-1.7
Med- Surg ICU	2/14/2023 1:00	8	12.0	10.3	-1.7
Med- Surg ICU	2/14/2023 2:00	8	12.0	10.3	-1.7
Med- Surg ICU	2/14/2023 3:00	8	12.0	10.3	-1.7
Med- Surg ICU	2/14/2023 4:00	8	12.0	10.3	-1.7
Med- Surg ICU	2/14/2023 5:00	8	12.0	10.3	-1.7
Med- Surg ICU	2/14/2023 6:00	8	12.0	10.3	-1.7
Med- Surg ICU	2/14/2023 7:00	8	12.0	10.3	-1.7
Med- Surg ICU	2/14/2023 8:00	9	12.0	10.0	-2.0
Med- Surg ICU	2/14/2023 9:00	9	12.0	10.0	-2.0

Med- Surg ICU	2/14/2023 10:00	9	12.0	10.0	-2.0
Med- Surg ICU	2/14/2023 11:00	9	12.0	10.0	-2.0
Med- Surg ICU	2/14/2023 12:00	9	12.0	10.0	-2.0
Med- Surg ICU	2/14/2023 13:00	9	12.0	10.0	-2.0
Med- Surg ICU	2/14/2023 14:00	9	12.0	10.0	-2.0
Med- Surg ICU	2/14/2023 15:00	9	12.0	10.0	-2.0
Med- Surg ICU	2/14/2023 16:00	9	12.0	7.7	-4.3
Med- Surg ICU	2/14/2023 17:00	9	12.0	7.7	-4.3
Med- Surg ICU	2/14/2023 18:00	9	12.0	7.7	-4.3
Med- Surg ICU	2/14/2023 19:00	7	12.0	7.7	-4.3
Med- Surg ICU	2/14/2023 20:00	7	12.0	9.0	-3.0
Med- Surg ICU	2/14/2023 21:00	7	12.0	9.0	-3.0
Med- Surg ICU	2/14/2023 22:00	7	12.0	9.0	-3.0
Med- Surg ICU	2/14/2023 23:00	7	12.0	9.0	-3.0
Med- Surg ICU	2/15/2023 0:00	7	10.0	8.3	-1.7
Med- Surg ICU	2/15/2023 1:00	7	10.0	7.7	-2.3
Med- Surg ICU	2/15/2023 2:00	7	10.0	7.7	-2.3
Med- Surg ICU	2/15/2023 3:00	7	10.0	7.7	-2.3
Med- Surg ICU	2/15/2023 4:00	8	10.0	7.7	-2.3
Med- Surg ICU	2/15/2023 5:00	8	10.0	7.7	-2.3
Med- Surg ICU	2/15/2023 6:00	8	10.0	7.7	-2.3
Med- Surg ICU	2/15/2023 7:00	8	10.0	7.7	-2.3
Med- Surg ICU	2/15/2023 8:00	8	10.0	8.7	-1.3
Med- Surg ICU	2/15/2023 9:00	8	10.0	8.7	-1.3
Med- Surg ICU	2/15/2023 10:00	7	10.0	8.7	-1.3
Med- Surg ICU	2/15/2023 11:00	7	10.0	8.7	-1.3
Med- Surg ICU	2/15/2023 12:00	7	10.0	8.7	-1.3
Med- Surg ICU	2/15/2023 13:00	7	10.0	8.7	-1.3
Med- Surg ICU	2/15/2023 14:00	7	10.0	8.7	-1.3
Med- Surg ICU	2/15/2023 15:00	7	10.0	8.7	-1.3
Med- Surg ICU	2/15/2023 16:00	7	10.0	10.6	0.6
Med- Surg ICU	2/15/2023 17:00	7	10.0	10.6	0.6
Med- Surg ICU	2/15/2023 18:00	7	10.0	10.6	0.6
Med- Surg ICU	2/15/2023 19:00	7	10.0	10.6	0.6
Med- Surg ICU	2/15/2023 20:00	9	13.0	10.3	-2.7
Med- Surg ICU	2/15/2023 21:00	9	13.0	10.3	-2.7
Med- Surg ICU	2/15/2023 22:00	9	13.0	10.3	-2.7
Med- Surg ICU	2/15/2023 23:00	9	13.0	10.3	-2.7
Med- Surg ICU	2/16/2023 0:00	10	13.0	10.3	-2.7
Med- Surg ICU	2/16/2023 1:00	10	13.0	10.3	-2.7
Med- Surg ICU	2/16/2023 2:00	10	13.0	10.3	-2.7
Med- Surg ICU	2/16/2023 3:00	10	13.0	10.3	-2.7
Med- Surg ICU	2/16/2023 4:00	10	13.0	10.3	-2.7
Med- Surg ICU	2/16/2023 5:00	10	13.0	10.3	-2.7
Med- Surg ICU	2/16/2023 6:00	10	13.0	10.3	-2.7
Med- Surg ICU	2/16/2023 7:00	10	13.0	10.3	-2.7
Med- Surg ICU	2/16/2023 8:00	10	13.0	12.5	-0.6

Med- Surg ICU	2/16/2023 9:00	10	13.0	12.5	-0.6
Med- Surg ICU	2/16/2023 10:00	10	13.0	12.5	-0.6
Med- Surg ICU	2/16/2023 11:00	10	13.0	12.5	-0.6
Med- Surg ICU	2/16/2023 12:00	10	13.0	12.5	-0.6
Med- Surg ICU	2/16/2023 13:00	10	13.0	12.5	-0.6
Med- Surg ICU	2/16/2023 14:00	10	13.0	12.5	-0.6
Med- Surg ICU	2/16/2023 15:00	9	13.0	12.5	-0.6
Med- Surg ICU	2/16/2023 16:00	9	13.0	13.6	0.6
Med- Surg ICU	2/16/2023 17:00	9	13.0	13.6	0.6
Med- Surg ICU	2/16/2023 18:00	9	13.0	13.6	0.6
Med- Surg ICU	2/16/2023 19:00	9	13.0	13.6	0.6
Med- Surg ICU	2/16/2023 20:00	9	13.0	10.3	-2.7
Med- Surg ICU	2/16/2023 21:00	9	13.0	10.3	-2.7
Med- Surg ICU	2/16/2023 22:00	9	13.0	10.3	-2.7
Med- Surg ICU	2/16/2023 23:00	9	13.0	10.3	-2.7
Med- Surg ICU	2/17/2023 0:00	9	13.0	10.3	-2.7
Med- Surg ICU	2/17/2023 1:00	9	13.0	10.3	-2.7
Med- Surg ICU	2/17/2023 2:00	9	13.0	10.3	-2.7
Med- Surg ICU	2/17/2023 3:00	9	13.0	10.3	-2.7
Med- Surg ICU	2/17/2023 4:00	9	13.0	10.3	-2.7
Med- Surg ICU	2/17/2023 5:00	9	13.0	10.3	-2.7
Med- Surg ICU	2/17/2023 6:00	9	13.0	10.3	-2.7
Med- Surg ICU	2/17/2023 7:00	9	13.0	10.3	-2.7
Med- Surg ICU	2/17/2023 8:00	9	13.0	10.0	-3.0
Med- Surg ICU	2/17/2023 9:00	9	13.0	10.0	-3.0
Med- Surg ICU	2/17/2023 10:00	9	13.0	10.0	-3.0
Med- Surg ICU	2/17/2023 11:00	9	13.0	10.0	-3.0
Med- Surg ICU	2/17/2023 12:00	9	13.0	10.0	-3.0
Med- Surg ICU	2/17/2023 13:00	9	13.0	10.0	-3.0
Med- Surg ICU	2/17/2023 14:00	9	13.0	10.0	-3.0
Med- Surg ICU	2/17/2023 15:00	9	13.0	10.0	-3.0
Med- Surg ICU	2/17/2023 16:00	9	13.0	10.3	-2.7
Med- Surg ICU	2/17/2023 17:00	9	13.0	10.3	-2.7
Med- Surg ICU	2/17/2023 18:00	9	13.0	10.3	-2.7
Med- Surg ICU	2/17/2023 19:00	9	13.0	10.3	-2.7
Med- Surg ICU	2/17/2023 20:00	10	13.0	10.3	-2.7
Med- Surg ICU	2/17/2023 21:00	10	13.0	10.3	-2.7
Med- Surg ICU	2/17/2023 22:00	10	13.0	10.3	-2.7
Med- Surg ICU	2/17/2023 23:00	10	13.0	10.3	-2.7
Med- Surg ICU	2/18/2023 0:00	10	13.0	10.3	-2.7
Med- Surg ICU	2/18/2023 1:00	10	13.0	10.3	-2.7
Med- Surg ICU	2/18/2023 2:00	10	13.0	10.3	-2.7
Med- Surg ICU	2/18/2023 3:00	10	13.0	10.3	-2.7
Med- Surg ICU	2/18/2023 4:00	10	13.0	10.3	-2.7
Med- Surg ICU	2/18/2023 5:00	10	13.0	10.3	-2.7
Med- Surg ICU	2/18/2023 6:00	10	13.0	10.3	-2.7
Med- Surg ICU	2/18/2023 7:00	10	13.0	10.3	-2.7

Med- Surg ICU	2/18/2023 8:00	10	11.0	8.7	-2.3
Med- Surg ICU	2/18/2023 9:00	10	11.0	8.7	-2.3
Med- Surg ICU	2/18/2023 10:00	10	11.0	8.7	-2.3
Med- Surg ICU	2/18/2023 11:00	10	11.0	8.7	-2.3
Med- Surg ICU	2/18/2023 12:00	10	11.0	10.0	-1.0
Med- Surg ICU	2/18/2023 13:00	10	11.0	10.0	-1.0
Med- Surg ICU	2/18/2023 14:00	10	11.0	10.0	-1.0
Med- Surg ICU	2/18/2023 15:00	10	11.0	10.6	-0.4
Med- Surg ICU	2/18/2023 16:00	10	11.0	10.3	-0.7
Med- Surg ICU	2/18/2023 17:00	10	11.0	10.3	-0.7
Med- Surg ICU	2/18/2023 18:00	10	11.0	10.3	-0.7
Med- Surg ICU	2/18/2023 19:00	10	11.0	10.3	-0.7
Med- Surg ICU	2/18/2023 20:00	9	13.0	10.3	-2.7
Med- Surg ICU	2/18/2023 21:00	9	13.0	10.3	-2.7
Med- Surg ICU	2/18/2023 22:00	9	13.0	10.3	-2.7
Med- Surg ICU	2/18/2023 23:00	9	13.0	10.3	-2.7
Med- Surg ICU	2/19/2023 0:00	9	13.0	10.3	-2.7
Med- Surg ICU	2/19/2023 1:00	9	13.0	10.3	-2.7
Med- Surg ICU	2/19/2023 2:00	9	13.0	10.3	-2.7
Med- Surg ICU	2/19/2023 3:00	8	13.0	9.3	-3.7
Med- Surg ICU	2/19/2023 4:00	8	13.0	10.3	-2.7
Med- Surg ICU	2/19/2023 5:00	8	13.0	10.3	-2.7
Med- Surg ICU	2/19/2023 6:00	8	13.0	10.3	-2.7
Med- Surg ICU	2/19/2023 7:00	8	13.0	10.3	-2.7
Med- Surg ICU	2/19/2023 8:00	8	13.0	7.5	-5.5
Med- Surg ICU	2/19/2023 9:00	8	13.0	7.5	-5.5
Med- Surg ICU	2/19/2023 10:00	8	13.0	7.5	-5.5
Med- Surg ICU	2/19/2023 11:00	8	13.0	7.5	-5.5
Med- Surg ICU	2/19/2023 12:00	8	13.0	7.5	-5.5
Med- Surg ICU	2/19/2023 13:00	8	13.0	7.5	-5.5
Med- Surg ICU	2/19/2023 14:00	7	13.0	7.5	-5.5
Med- Surg ICU	2/19/2023 15:00	7	13.0	7.5	-5.5
Med- Surg ICU	2/19/2023 16:00	7	13.0	7.7	-5.3
Med- Surg ICU	2/19/2023 17:00	7	13.0	7.7	-5.3
Med- Surg ICU	2/19/2023 18:00	6	13.0	7.7	-5.3
Med- Surg ICU	2/19/2023 19:00	6	13.0	7.7	-5.3
Med- Surg ICU	2/19/2023 20:00	6	10.0	6.4	-3.6
Med- Surg ICU	2/19/2023 21:00	6	10.0	6.4	-3.6
Med- Surg ICU	2/19/2023 22:00	6	10.0	6.4	-3.6
Med- Surg ICU	2/19/2023 23:00	6	10.0	6.4	-3.6
Med- Surg ICU	2/20/2023 0:00	6	10.0	6.4	-3.6
Med- Surg ICU	2/20/2023 1:00	6	10.0	6.4	-3.6
Med- Surg ICU	2/20/2023 2:00	6	10.0	6.4	-3.6
Med- Surg ICU	2/20/2023 3:00	6	10.0	6.4	-3.6
Med- Surg ICU	2/20/2023 4:00	6	10.0	6.4	-3.6
Med- Surg ICU	2/20/2023 5:00	6	10.0	6.4	-3.6
Med- Surg ICU	2/20/2023 6:00	6	10.0	6.4	-3.6

Med- Surg ICU	2/20/2023 7:00	6	10.0	6.4	-3.6
Med- Surg ICU	2/20/2023 8:00	6	10.0	6.2	-3.8
Med- Surg ICU	2/20/2023 9:00	6	10.0	6.2	-3.8
Med- Surg ICU	2/20/2023 10:00	6	10.0	6.2	-3.8
Med- Surg ICU	2/20/2023 11:00	6	10.0	6.2	-3.8
Med- Surg ICU	2/20/2023 12:00	6	10.0	6.2	-3.8
Med- Surg ICU	2/20/2023 13:00	6	10.0	6.2	-3.8
Med- Surg ICU	2/20/2023 14:00	6	10.0	6.2	-3.8
Med- Surg ICU	2/20/2023 15:00	6	10.0	6.2	-3.8
Med- Surg ICU	2/20/2023 16:00	6	10.0	6.4	-3.6
Med- Surg ICU	2/20/2023 17:00	6	10.0	6.4	-3.6
Med- Surg ICU	2/20/2023 18:00	6	10.0	6.4	-3.6
Med- Surg ICU	2/20/2023 19:00	6	10.0	6.4	-3.6
Med- Surg ICU	2/20/2023 20:00	7	13.0	7.7	-5.3
Med- Surg ICU	2/20/2023 21:00	7	13.0	7.7	-5.3
Med- Surg ICU	2/20/2023 22:00	7	13.0	7.7	-5.3
Med- Surg ICU	2/20/2023 23:00	7	13.0	7.7	-5.3
Med- Surg ICU	2/21/2023 0:00	7	13.0	7.7	-5.3
Med- Surg ICU	2/21/2023 1:00	7	13.0	7.7	-5.3
Med- Surg ICU	2/21/2023 2:00	7	13.0	7.7	-5.3
Med- Surg ICU	2/21/2023 3:00	7	13.0	7.7	-5.3
Med- Surg ICU	2/21/2023 4:00	7	13.0	7.7	-5.3
Med- Surg ICU	2/21/2023 5:00	7	13.0	7.7	-5.3
Med- Surg ICU	2/21/2023 6:00	7	13.0	7.7	-5.3
Med- Surg ICU	2/21/2023 7:00	7	13.0	7.7	-5.3
Med- Surg ICU	2/21/2023 8:00	7	13.0	11.2	-1.8
Med- Surg ICU	2/21/2023 9:00	7	13.0	11.2	-1.8
Med- Surg ICU	2/21/2023 10:00	7	13.0	11.2	-1.8
Med- Surg ICU	2/21/2023 11:00	7	13.0	11.2	-1.8
Med- Surg ICU	2/21/2023 12:00	7	13.0	11.2	-1.8
Med- Surg ICU	2/21/2023 13:00	7	13.0	11.2	-1.8
Med- Surg ICU	2/21/2023 14:00	7	13.0	11.2	-1.8
Med- Surg ICU	2/21/2023 15:00	7	13.0	11.2	-1.8
Med- Surg ICU	2/21/2023 16:00	7	13.0	11.5	-1.5
Med- Surg ICU	2/21/2023 17:00	7	13.0	11.5	-1.5
Med- Surg ICU	2/21/2023 18:00	7	13.0	11.5	-1.5
Med- Surg ICU	2/21/2023 19:00	7	13.0	11.5	-1.5
Med- Surg ICU	2/21/2023 20:00	7	13.0	9.0	-4.0
Med- Surg ICU	2/21/2023 21:00	7	13.0	9.0	-4.0
Med- Surg ICU	2/21/2023 22:00	7	13.0	9.0	-4.0
Med- Surg ICU	2/21/2023 23:00	7	13.0	9.0	-4.0
Med- Surg ICU	2/22/2023 0:00	7	13.0	10.3	-2.7
Med- Surg ICU	2/22/2023 1:00	7	13.0	10.3	-2.7
Med- Surg ICU	2/22/2023 2:00	7	13.0	10.3	-2.7
Med- Surg ICU	2/22/2023 3:00	7	13.0	10.3	-2.7
Med- Surg ICU	2/22/2023 4:00	7	13.0	10.3	-2.7
Med- Surg ICU	2/22/2023 5:00	7	13.0	10.3	-2.7

Med- Surg ICU	2/22/2023 6:00	7	13.0	10.3	-2.7
Med- Surg ICU	2/22/2023 7:00	7	13.0	10.3	-2.7
Med- Surg ICU	2/22/2023 8:00	6	11.0	11.2	0.2
Med- Surg ICU	2/22/2023 9:00	6	11.0	11.2	0.2
Med- Surg ICU	2/22/2023 10:00	6	11.0	11.2	0.2
Med- Surg ICU	2/22/2023 11:00	6	10.0	9.0	-1.0
Med- Surg ICU	2/22/2023 12:00	6	10.0	10.0	0.0
Med- Surg ICU	2/22/2023 13:00	6	10.0	10.0	0.0
Med- Surg ICU	2/22/2023 14:00	6	10.0	9.0	-1.0
Med- Surg ICU	2/22/2023 15:00	6	10.0	10.0	0.0
Med- Surg ICU	2/22/2023 16:00	6	10.0	7.6	-2.5
Med- Surg ICU	2/22/2023 17:00	6	10.0	7.6	-2.5
Med- Surg ICU	2/22/2023 18:00	6	10.0	7.6	-2.5
Med- Surg ICU	2/22/2023 19:00	7	10.0	7.6	-2.5
Med- Surg ICU	2/22/2023 20:00	7	10.0	9.0	-1.0
Med- Surg ICU	2/22/2023 21:00	8	11.0	9.0	-2.0
Med- Surg ICU	2/22/2023 22:00	8	11.0	9.0	-2.0
Med- Surg ICU	2/22/2023 23:00	8	11.0	9.0	-2.0
Med- Surg ICU	2/23/2023 0:00	8	11.0	9.0	-2.0
Med- Surg ICU	2/23/2023 1:00	8	11.0	9.0	-2.0
Med- Surg ICU	2/23/2023 2:00	8	11.0	9.0	-2.0
Med- Surg ICU	2/23/2023 3:00	8	11.0	9.0	-2.0
Med- Surg ICU	2/23/2023 4:00	8	11.0	9.0	-2.0
Med- Surg ICU	2/23/2023 5:00	8	11.0	9.0	-2.0
Med- Surg ICU	2/23/2023 6:00	8	11.0	9.0	-2.0
Med- Surg ICU	2/23/2023 7:00	8	11.0	9.0	-2.0
Med- Surg ICU	2/23/2023 8:00	8	11.0	10.0	-1.0
Med- Surg ICU	2/23/2023 9:00	8	11.0	10.0	-1.0
Med- Surg ICU	2/23/2023 10:00	8	11.0	10.0	-1.0
Med- Surg ICU	2/23/2023 11:00	8	11.0	10.0	-1.0
Med- Surg ICU	2/23/2023 12:00	8	11.0	10.0	-1.0
Med- Surg ICU	2/23/2023 13:00	8	11.0	10.0	-1.0
Med- Surg ICU	2/23/2023 14:00	8	11.0	10.0	-1.0
Med- Surg ICU	2/23/2023 15:00	8	11.0	10.0	-1.0
Med- Surg ICU	2/23/2023 16:00	8	11.0	9.0	-2.0
Med- Surg ICU	2/23/2023 17:00	9	10.0	6.0	-4.0
Med- Surg ICU	2/23/2023 18:00	9	10.0	6.0	-4.0
Med- Surg ICU	2/23/2023 19:00	9	10.0	6.0	-4.0
Med- Surg ICU	2/23/2023 20:00	8	10.0	9.0	-1.0
Med- Surg ICU	2/23/2023 21:00	8	10.0	9.0	-1.0
Med- Surg ICU	2/23/2023 22:00	8	10.0	9.0	-1.0
Med- Surg ICU	2/23/2023 23:00	8	10.0	9.0	-1.0
Med- Surg ICU	2/24/2023 0:00	9	10.0	10.3	0.3
Med- Surg ICU	2/24/2023 1:00	9	10.0	10.3	0.3
Med- Surg ICU	2/24/2023 2:00	9	10.0	10.3	0.3
Med- Surg ICU	2/24/2023 3:00	9	10.0	10.3	0.3
Med- Surg ICU	2/24/2023 4:00	9	10.0	10.3	0.3

Med- Surg ICU	2/24/2023 5:00	9	10.0	10.3	0.3
Med- Surg ICU	2/24/2023 6:00	9	10.0	10.3	0.3
Med- Surg ICU	2/24/2023 7:00	9	10.0	10.3	0.3
Med- Surg ICU	2/24/2023 8:00	9	10.0	8.7	-1.3
Med- Surg ICU	2/24/2023 9:00	9	10.0	8.7	-1.3
Med- Surg ICU	2/24/2023 10:00	9	10.0	8.7	-1.3
Med- Surg ICU	2/24/2023 11:00	9	10.0	8.7	-1.3
Med- Surg ICU	2/24/2023 12:00	9	10.0	8.7	-1.3
Med- Surg ICU	2/24/2023 13:00	9	10.0	8.7	-1.3
Med- Surg ICU	2/24/2023 14:00	9	10.0	8.7	-1.3
Med- Surg ICU	2/24/2023 15:00	9	10.0	8.7	-1.3
Med- Surg ICU	2/24/2023 16:00	9	10.0	7.7	-2.3
Med- Surg ICU	2/24/2023 17:00	9	10.0	7.7	-2.3
Med- Surg ICU	2/24/2023 18:00	9	10.0	7.7	-2.3
Med- Surg ICU	2/24/2023 19:00	9	10.0	7.7	-2.3
Med- Surg ICU	2/24/2023 20:00	9	10.0	9.0	-1.0
Med- Surg ICU	2/24/2023 21:00	7	12.0	7.7	-4.3
Med- Surg ICU	2/24/2023 22:00	7	12.0	7.7	-4.3
Med- Surg ICU	2/24/2023 23:00	7	12.0	7.7	-4.3
Med- Surg ICU	2/25/2023 0:00	7	12.0	9.0	-3.0
Med- Surg ICU	2/25/2023 1:00	7	12.0	9.0	-3.0
Med- Surg ICU	2/25/2023 2:00	7	12.0	9.0	-3.0
Med- Surg ICU	2/25/2023 3:00	7	12.0	9.0	-3.0
Med- Surg ICU	2/25/2023 4:00	7	12.0	9.0	-3.0
Med- Surg ICU	2/25/2023 5:00	7	12.0	9.0	-3.0
Med- Surg ICU	2/25/2023 6:00	7	12.0	9.0	-3.0
Med- Surg ICU	2/25/2023 7:00	7	12.0	9.0	-3.0
Med- Surg ICU	2/25/2023 8:00	7	12.0	6.2	-5.8
Med- Surg ICU	2/25/2023 9:00	7	12.0	6.2	-5.8
Med- Surg ICU	2/25/2023 10:00	7	12.0	6.2	-5.8
Med- Surg ICU	2/25/2023 11:00	7	12.0	6.2	-5.8
Med- Surg ICU	2/25/2023 12:00	7	12.0	6.2	-5.8
Med- Surg ICU	2/25/2023 13:00	7	12.0	6.2	-5.8
Med- Surg ICU	2/25/2023 14:00	7	12.0	6.2	-5.8
Med- Surg ICU	2/25/2023 15:00	7	12.0	6.2	-5.8
Med- Surg ICU	2/25/2023 16:00	7	12.0	4.5	-7.5
Med- Surg ICU	2/25/2023 17:00	7	12.0	4.5	-7.5
Med- Surg ICU	2/25/2023 18:00	7	12.0	4.5	-7.5
Med- Surg ICU	2/25/2023 19:00	7	12.0	4.5	-7.5
Med- Surg ICU	2/25/2023 20:00	6	12.0	9.0	-3.0
Med- Surg ICU	2/25/2023 21:00	6	12.0	9.0	-3.0
Med- Surg ICU	2/25/2023 22:00	6	12.0	9.0	-3.0
Med- Surg ICU	2/25/2023 23:00	6	12.0	9.0	-3.0
Med- Surg ICU	2/26/2023 0:00	6	12.0	7.7	-4.3
Med- Surg ICU	2/26/2023 1:00	6	12.0	7.7	-4.3
Med- Surg ICU	2/26/2023 2:00	6	12.0	7.7	-4.3
Med- Surg ICU	2/26/2023 3:00	6	12.0	7.7	-4.3

Med- Surg ICU	2/26/2023 4:00	6	12.0	7.7	-4.3
Med- Surg ICU	2/26/2023 5:00	6	12.0	7.7	-4.3
Med- Surg ICU	2/26/2023 6:00	6	12.0	7.7	-4.3
Med- Surg ICU	2/26/2023 7:00	6	12.0	7.7	-4.3
Med- Surg ICU	2/26/2023 8:00	6	12.0	8.7	-3.3
Med- Surg ICU	2/26/2023 9:00	6	12.0	8.7	-3.3
Med- Surg ICU	2/26/2023 10:00	6	12.0	8.7	-3.3
Med- Surg ICU	2/26/2023 11:00	6	12.0	8.7	-3.3
Med- Surg ICU	2/26/2023 12:00	6	12.0	8.7	-3.3
Med- Surg ICU	2/26/2023 13:00	6	12.0	8.7	-3.3
Med- Surg ICU	2/26/2023 14:00	6	12.0	8.7	-3.3
Med- Surg ICU	2/26/2023 15:00	6	12.0	8.7	-3.3
Med- Surg ICU	2/26/2023 16:00	6	12.0	7.6	-4.5
Med- Surg ICU	2/26/2023 17:00	6	12.0	7.6	-4.5
Med- Surg ICU	2/26/2023 18:00	6	12.0	7.6	-4.5
Med- Surg ICU	2/26/2023 19:00	6	12.0	7.6	-4.5
Med- Surg ICU	2/26/2023 20:00	7	12.0	7.7	-4.3
Med- Surg ICU	2/26/2023 21:00	7	12.0	7.7	-4.3
Med- Surg ICU	2/26/2023 22:00	7	12.0	7.7	-4.3
Med- Surg ICU	2/26/2023 23:00	7	12.0	7.7	-4.3
Med- Surg ICU	2/27/2023 0:00	7	12.0	7.7	-4.3
Med- Surg ICU	2/27/2023 1:00	7	12.0	7.7	-4.3
Med- Surg ICU	2/27/2023 2:00	7	12.0	7.7	-4.3
Med- Surg ICU	2/27/2023 3:00	7	12.0	7.7	-4.3
Med- Surg ICU	2/27/2023 4:00	7	12.0	7.7	-4.3
Med- Surg ICU	2/27/2023 5:00	7	12.0	7.7	-4.3
Med- Surg ICU	2/27/2023 6:00	7	12.0	7.7	-4.3
Med- Surg ICU	2/27/2023 7:00	7	12.0	7.7	-4.3
Med- Surg ICU	2/27/2023 8:00	7	12.0	10.6	-1.4
Med- Surg ICU	2/27/2023 9:00	7	12.0	10.0	-2.0
Med- Surg ICU	2/27/2023 10:00	7	12.0	10.0	-2.0
Med- Surg ICU	2/27/2023 11:00	7	12.0	10.0	-2.0
Med- Surg ICU	2/27/2023 12:00	7	12.0	10.0	-2.0
Med- Surg ICU	2/27/2023 13:00	7	12.0	10.0	-2.0
Med- Surg ICU	2/27/2023 14:00	7	12.0	10.0	-2.0
Med- Surg ICU	2/27/2023 15:00	7	12.0	10.0	-2.0
Med- Surg ICU	2/27/2023 16:00	7	12.0	10.6	-1.4
Med- Surg ICU	2/27/2023 17:00	7	12.0	10.6	-1.4
Med- Surg ICU	2/27/2023 18:00	7	12.0	10.6	-1.4
Med- Surg ICU	2/27/2023 19:00	7	12.0	10.6	-1.4
Med- Surg ICU	2/27/2023 20:00	9	12.0	10.3	-1.7
Med- Surg ICU	2/27/2023 21:00	9	12.0	10.3	-1.7
Med- Surg ICU	2/27/2023 22:00	10	12.0	10.3	-1.7
Med- Surg ICU	2/27/2023 23:00	10	12.0	10.3	-1.7
Med- Surg ICU	2/28/2023 0:00	10	12.0	7.7	-4.3
Med- Surg ICU	2/28/2023 1:00	10	12.0	7.7	-4.3
Med- Surg ICU	2/28/2023 2:00	10	12.0	7.7	-4.3

Med- Surg ICU	2/28/2023 3:00	10	12.0	7.7	-4.3
Med- Surg ICU	2/28/2023 4:00	10	12.0	7.7	-4.3
Med- Surg ICU	2/28/2023 5:00	10	12.0	7.7	-4.3
Med- Surg ICU	2/28/2023 6:00	10	12.0	7.7	-4.3
Med- Surg ICU	2/28/2023 7:00	10	12.0	5.1	-6.9
Med- Surg ICU	2/28/2023 8:00	10	12.0	7.5	-4.5
Med- Surg ICU	2/28/2023 9:00	10	12.0	7.5	-4.5
Med- Surg ICU	2/28/2023 10:00	10	13.0	8.7	-4.3
Med- Surg ICU	2/28/2023 11:00	10	13.0	8.7	-4.3
Med- Surg ICU	2/28/2023 12:00	10	13.0	8.7	-4.3
Med- Surg ICU	2/28/2023 13:00	10	13.0	8.7	-4.3
Med- Surg ICU	2/28/2023 14:00	10	13.0	8.7	-4.3
Med- Surg ICU	2/28/2023 15:00	10	13.0	8.7	-4.3
Med- Surg ICU	2/28/2023 16:00	10	13.0	6.4	-6.6
Med- Surg ICU	2/28/2023 17:00	10	13.0	6.4	-6.6
Med- Surg ICU	2/28/2023 18:00	10	13.0	6.4	-6.6
Med- Surg ICU	2/28/2023 19:00	10	13.0	6.4	-6.6
Med- Surg ICU	2/28/2023 20:00	10	13.0	7.7	-5.3
Med- Surg ICU	2/28/2023 21:00	10	13.0	7.7	-5.3
Med- Surg ICU	2/28/2023 22:00	10	13.0	7.7	-5.3
Med- Surg ICU	2/28/2023 23:00	10	13.0	7.7	-5.3
Med- Surg ICU	3/1/2023 0:00	10	13.0	9.0	-4.0
Med- Surg ICU	3/1/2023 1:00	10	13.0	9.0	-4.0
Med- Surg ICU	3/1/2023 2:00	10	13.0	9.0	-4.0
Med- Surg ICU	3/1/2023 3:00	10	13.0	9.0	-4.0
Med- Surg ICU	3/1/2023 4:00	10	13.0	9.0	-4.0
Med- Surg ICU	3/1/2023 5:00	10	13.0	9.0	-4.0
Med- Surg ICU	3/1/2023 6:00	10	13.0	9.0	-4.0
Med- Surg ICU	3/1/2023 7:00	10	13.0	9.0	-4.0
Med- Surg ICU	3/1/2023 8:00	10	13.0	8.7	-4.3
Med- Surg ICU	3/1/2023 9:00	10	13.0	8.7	-4.3
Med- Surg ICU	3/1/2023 10:00	10	13.0	8.7	-4.3
Med- Surg ICU	3/1/2023 11:00	10	13.0	8.7	-4.3
Med- Surg ICU	3/1/2023 12:00	10	13.0	8.7	-4.3
Med- Surg ICU	3/1/2023 13:00	11	12.0	8.7	-3.3
Med- Surg ICU	3/1/2023 14:00	11	12.0	8.7	-3.3
Med- Surg ICU	3/1/2023 15:00	11	12.0	8.7	-3.3
Med- Surg ICU	3/1/2023 16:00	11	12.0	9.0	-3.0
Med- Surg ICU	3/1/2023 17:00	11	12.0	9.0	-3.0
Med- Surg ICU	3/1/2023 18:00	11	12.0	9.0	-3.0
Med- Surg ICU	3/1/2023 19:00	11	12.0	9.0	-3.0
Med- Surg ICU	3/1/2023 20:00	11	11.0	6.4	-4.6
Med- Surg ICU	3/1/2023 21:00	11	11.0	6.4	-4.6
Med- Surg ICU	3/1/2023 22:00	11	11.0	6.4	-4.6
Med- Surg ICU	3/1/2023 23:00	11	11.0	6.4	-4.6
Med- Surg ICU	3/2/2023 0:00	11	11.0	7.6	-3.5
Med- Surg ICU	3/2/2023 1:00	11	11.0	7.6	-3.5

Med- Surg ICU	3/2/2023 2:00	11	11.0	7.6	-3.5
Med- Surg ICU	3/2/2023 3:00	11	11.0	7.6	-3.5
Med- Surg ICU	3/2/2023 4:00	11	11.0	7.6	-3.5
Med- Surg ICU	3/2/2023 5:00	11	11.0	7.6	-3.5
Med- Surg ICU	3/2/2023 6:00	11	11.0	7.6	-3.5
Med- Surg ICU	3/2/2023 7:00	11	11.0	7.6	-3.5
Med- Surg ICU	3/2/2023 8:00	11	11.0	8.7	-2.3
Med- Surg ICU	3/2/2023 9:00	11	12.0	11.2	-0.8
Med- Surg ICU	3/2/2023 10:00	11	12.0	11.2	-0.8
Med- Surg ICU	3/2/2023 11:00	11	12.0	11.2	-0.8
Med- Surg ICU	3/2/2023 12:00	11	12.0	11.2	-0.8
Med- Surg ICU	3/2/2023 13:00	11	12.0	10.3	-1.7
Med- Surg ICU	3/2/2023 14:00	11	12.0	11.2	-0.8
Med- Surg ICU	3/2/2023 15:00	11	12.0	11.2	-0.8
Med- Surg ICU	3/2/2023 16:00	11	12.0	9.1	-3.0
Med- Surg ICU	3/2/2023 17:00	11	12.0	9.1	-3.0
Med- Surg ICU	3/2/2023 18:00	11	12.0	9.1	-3.0
Med- Surg ICU	3/2/2023 19:00	11	12.0	9.1	-3.0
Med- Surg ICU	3/2/2023 20:00	10	12.0	7.7	-4.3
Med- Surg ICU	3/2/2023 21:00	10	12.0	7.7	-4.3
Med- Surg ICU	3/2/2023 22:00	10	12.0	7.7	-4.3
Med- Surg ICU	3/2/2023 23:00	10	12.0	7.7	-4.3
Med- Surg ICU	3/3/2023 0:00	10	12.0	7.7	-4.3
Med- Surg ICU	3/3/2023 1:00	10	12.0	7.7	-4.3
Med- Surg ICU	3/3/2023 2:00	10	12.0	7.7	-4.3
Med- Surg ICU	3/3/2023 3:00	10	12.0	7.7	-4.3
Med- Surg ICU	3/3/2023 4:00	10	12.0	7.7	-4.3
Med- Surg ICU	3/3/2023 5:00	10	12.0	7.7	-4.3
Med- Surg ICU	3/3/2023 6:00	10	12.0	7.7	-4.3
Med- Surg ICU	3/3/2023 7:00	10	12.0	7.7	-4.3
Med- Surg ICU	3/3/2023 8:00	10	12.0	10.0	-2.0
Med- Surg ICU	3/3/2023 9:00	10	12.0	10.0	-2.0
Med- Surg ICU	3/3/2023 10:00	10	12.0	10.0	-2.0
Med- Surg ICU	3/3/2023 11:00	10	12.0	10.0	-2.0
Med- Surg ICU	3/3/2023 12:00	10	12.0	10.0	-2.0
Med- Surg ICU	3/3/2023 13:00	13	12.0	11.2	-0.8
Med- Surg ICU	3/3/2023 14:00	13	12.0	11.2	-0.8
Med- Surg ICU	3/3/2023 15:00	12	12.0	11.2	-0.8
Med- Surg ICU	3/3/2023 16:00	12	12.0	8.3	-3.7
Med- Surg ICU	3/3/2023 17:00	12	12.0	7.7	-4.3
Med- Surg ICU	3/3/2023 18:00	12	12.0	7.7	-4.3
Med- Surg ICU	3/3/2023 19:00	12	12.0	7.7	-4.3
Med- Surg ICU	3/3/2023 20:00	12	12.0	9.0	-3.0
Med- Surg ICU	3/3/2023 21:00	13	12.0	9.0	-3.0
Med- Surg ICU	3/3/2023 22:00	11	15.0	9.0	-6.0
Med- Surg ICU	3/3/2023 23:00	11	15.0	9.0	-6.0
Med- Surg ICU	3/4/2023 0:00	11	15.0	10.3	-4.7

Med- Surg ICU	3/4/2023 1:00	11	15.0	10.3	-4.7
Med- Surg ICU	3/4/2023 2:00	11	15.0	10.3	-4.7
Med- Surg ICU	3/4/2023 3:00	11	15.0	10.3	-4.7
Med- Surg ICU	3/4/2023 4:00	11	15.0	10.3	-4.7
Med- Surg ICU	3/4/2023 5:00	11	15.0	10.3	-4.7
Med- Surg ICU	3/4/2023 6:00	11	15.0	10.3	-4.7
Med- Surg ICU	3/4/2023 7:00	11	15.0	10.3	-4.7
Med- Surg ICU	3/4/2023 8:00	11	12.0	10.0	-2.0
Med- Surg ICU	3/4/2023 9:00	11	12.0	10.0	-2.0
Med- Surg ICU	3/4/2023 10:00	11	12.0	10.0	-2.0
Med- Surg ICU	3/4/2023 11:00	11	12.0	10.0	-2.0
Med- Surg ICU	3/4/2023 12:00	11	12.0	11.2	-0.8
Med- Surg ICU	3/4/2023 13:00	11	12.0	11.2	-0.8
Med- Surg ICU	3/4/2023 14:00	11	12.0	11.2	-0.8
Med- Surg ICU	3/4/2023 15:00	11	12.0	11.2	-0.8
Med- Surg ICU	3/4/2023 16:00	11	12.0	10.3	-1.7
Med- Surg ICU	3/4/2023 17:00	11	12.0	10.3	-1.7
Med- Surg ICU	3/4/2023 18:00	11	12.0	10.3	-1.7
Med- Surg ICU	3/4/2023 19:00	11	12.0	10.3	-1.7
Med- Surg ICU	3/4/2023 20:00	12	13.0	10.3	-2.7
Med- Surg ICU	3/4/2023 21:00	12	13.0	10.3	-2.7
Med- Surg ICU	3/4/2023 22:00	12	13.0	10.3	-2.7
Med- Surg ICU	3/4/2023 23:00	12	13.0	10.3	-2.7
Med- Surg ICU	3/5/2023 0:00	12	13.0	11.5	-1.5
Med- Surg ICU	3/5/2023 1:00	12	13.0	11.5	-1.5
Med- Surg ICU	3/5/2023 2:00	12	13.0	11.5	-1.5
Med- Surg ICU	3/5/2023 3:00	12	13.0	11.5	-1.5
Med- Surg ICU	3/5/2023 4:00	12	13.0	11.5	-1.5
Med- Surg ICU	3/5/2023 5:00	12	13.0	11.5	-1.5
Med- Surg ICU	3/5/2023 6:00	12	13.0	11.5	-1.5
Med- Surg ICU	3/5/2023 7:00	12	13.0	11.5	-1.5
Med- Surg ICU	3/5/2023 8:00	12	13.0	11.2	-1.8
Med- Surg ICU	3/5/2023 9:00	12	13.0	11.2	-1.8
Med- Surg ICU	3/5/2023 10:00	12	13.0	11.2	-1.8
Med- Surg ICU	3/5/2023 11:00	12	13.0	11.2	-1.8
Med- Surg ICU	3/5/2023 12:00	12	13.0	11.2	-1.8
Med- Surg ICU	3/5/2023 13:00	12	13.0	11.2	-1.8
Med- Surg ICU	3/5/2023 14:00	12	13.0	11.2	-1.8
Med- Surg ICU	3/5/2023 15:00	12	13.0	11.2	-1.8
Med- Surg ICU	3/5/2023 16:00	12	13.0	11.5	-1.5
Med- Surg ICU	3/5/2023 17:00	12	13.0	11.5	-1.5
Med- Surg ICU	3/5/2023 18:00	12	13.0	11.5	-1.5
Med- Surg ICU	3/5/2023 19:00	12	13.0	11.5	-1.5
Med- Surg ICU	3/5/2023 20:00	12	13.0	9.0	-4.0
Med- Surg ICU	3/5/2023 21:00	12	13.0	9.0	-4.0
Med- Surg ICU	3/5/2023 22:00	12	13.0	9.0	-4.0
Med- Surg ICU	3/5/2023 23:00	12	13.0	9.0	-4.0

Med- Surg ICU	3/6/2023 0:00	12	13.0	9.0	-4.0
Med- Surg ICU	3/6/2023 1:00	12	13.0	9.0	-4.0
Med- Surg ICU	3/6/2023 2:00	12	13.0	9.0	-4.0
Med- Surg ICU	3/6/2023 3:00	12	13.0	9.0	-4.0
Med- Surg ICU	3/6/2023 4:00	12	13.0	9.0	-4.0
Med- Surg ICU	3/6/2023 5:00	12	13.0	9.0	-4.0
Med- Surg ICU	3/6/2023 6:00	12	13.0	9.0	-4.0
Med- Surg ICU	3/6/2023 7:00	12	13.0	9.0	-4.0
Med- Surg ICU	3/6/2023 8:00	12	13.0	8.7	-4.3
Med- Surg ICU	3/6/2023 9:00	12	13.0	8.7	-4.3
Med- Surg ICU	3/6/2023 10:00	12	13.0	11.2	-1.8
Med- Surg ICU	3/6/2023 11:00	12	13.0	11.2	-1.8
Med- Surg ICU	3/6/2023 12:00	12	13.0	11.2	-1.8
Med- Surg ICU	3/6/2023 13:00	12	13.0	11.2	-1.8
Med- Surg ICU	3/6/2023 14:00	12	13.0	11.2	-1.8
Med- Surg ICU	3/6/2023 15:00	12	13.0	11.2	-1.8
Med- Surg ICU	3/6/2023 16:00	12	13.0	10.6	-2.4
Med- Surg ICU	3/6/2023 17:00	12	13.0	10.6	-2.4
Med- Surg ICU	3/6/2023 18:00	12	13.0	10.6	-2.4
Med- Surg ICU	3/6/2023 19:00	11	12.0	10.6	-1.4
Med- Surg ICU	3/6/2023 20:00	11	12.0	9.1	-3.0
Med- Surg ICU	3/6/2023 21:00	11	12.0	9.1	-3.0
Med- Surg ICU	3/6/2023 22:00	11	12.0	9.1	-3.0
Med- Surg ICU	3/6/2023 23:00	11	12.0	9.1	-3.0
Med- Surg ICU	3/7/2023 0:00	11	12.0	7.7	-4.3
Med- Surg ICU	3/7/2023 1:00	11	12.0	7.7	-4.3
Med- Surg ICU	3/7/2023 2:00	11	12.0	7.7	-4.3
Med- Surg ICU	3/7/2023 3:00	11	12.0	7.7	-4.3
Med- Surg ICU	3/7/2023 4:00	11	12.0	7.7	-4.3
Med- Surg ICU	3/7/2023 5:00	11	12.0	7.7	-4.3
Med- Surg ICU	3/7/2023 6:00	11	12.0	7.7	-4.3
Med- Surg ICU	3/7/2023 7:00	11	12.0	7.7	-4.3
Med- Surg ICU	3/7/2023 8:00	11	13.0	10.0	-3.0
Med- Surg ICU	3/7/2023 9:00	11	13.0	10.0	-3.0
Med- Surg ICU	3/7/2023 10:00	11	13.0	10.0	-3.0
Med- Surg ICU	3/7/2023 11:00	11	13.0	10.0	-3.0
Med- Surg ICU	3/7/2023 12:00	11	13.0	10.0	-3.0
Med- Surg ICU	3/7/2023 13:00	11	13.0	10.0	-3.0
Med- Surg ICU	3/7/2023 14:00	11	13.0	10.0	-3.0
Med- Surg ICU	3/7/2023 15:00	11	13.0	10.0	-3.0
Med- Surg ICU	3/7/2023 16:00	11	13.0	6.0	-7.0
Med- Surg ICU	3/7/2023 17:00	11	13.0	6.0	-7.0
Med- Surg ICU	3/7/2023 18:00	11	13.0	6.0	-7.0
Med- Surg ICU	3/7/2023 19:00	11	11.0	6.0	-5.0
Med- Surg ICU	3/7/2023 20:00	11	11.0	10.3	-0.7
Med- Surg ICU	3/7/2023 21:00	11	11.0	10.3	-0.7
Med- Surg ICU	3/7/2023 22:00	11	11.0	10.3	-0.7

Med- Surg ICU	3/7/2023 23:00	12	11.0	9.3	-1.7
Med- Surg ICU	3/8/2023 0:00	11	11.0	9.0	-2.0
Med- Surg ICU	3/8/2023 1:00	11	11.0	9.0	-2.0
Med- Surg ICU	3/8/2023 2:00	11	11.0	9.0	-2.0
Med- Surg ICU	3/8/2023 3:00	11	11.0	9.0	-2.0
Med- Surg ICU	3/8/2023 4:00	11	11.0	9.0	-2.0
Med- Surg ICU	3/8/2023 5:00	11	11.0	9.0	-2.0
Med- Surg ICU	3/8/2023 6:00	11	11.0	9.0	-2.0
Med- Surg ICU	3/8/2023 7:00	11	11.0	9.0	-2.0
Med- Surg ICU	3/8/2023 8:00	11	11.0	12.5	1.5
Med- Surg ICU	3/8/2023 9:00	11	11.0	12.5	1.5
Med- Surg ICU	3/8/2023 10:00	11	11.0	12.5	1.5
Med- Surg ICU	3/8/2023 11:00	11	11.0	12.5	1.5
Med- Surg ICU	3/8/2023 12:00	11	11.0	12.5	1.5
Med- Surg ICU	3/8/2023 13:00	11	11.0	12.5	1.5
Med- Surg ICU	3/8/2023 14:00	11	11.0	12.5	1.5
Med- Surg ICU	3/8/2023 15:00	11	11.0	12.5	1.5
Med- Surg ICU	3/8/2023 16:00	11	11.0	12.1	1.1
Med- Surg ICU	3/8/2023 17:00	11	11.0	12.1	1.1
Med- Surg ICU	3/8/2023 18:00	11	11.0	12.1	1.1
Med- Surg ICU	3/8/2023 19:00	12	13.0	10.6	-2.4
Med- Surg ICU	3/8/2023 20:00	12	13.0	11.5	-1.5
Med- Surg ICU	3/8/2023 21:00	12	13.0	11.5	-1.5
Med- Surg ICU	3/8/2023 22:00	12	13.0	11.5	-1.5
Med- Surg ICU	3/8/2023 23:00	12	13.0	11.5	-1.5
Med- Surg ICU	3/9/2023 0:00	12	13.0	10.3	-2.7
Med- Surg ICU	3/9/2023 1:00	12	13.0	10.3	-2.7
Med- Surg ICU	3/9/2023 2:00	12	13.0	10.3	-2.7
Med- Surg ICU	3/9/2023 3:00	12	13.0	10.3	-2.7
Med- Surg ICU	3/9/2023 4:00	12	13.0	10.3	-2.7
Med- Surg ICU	3/9/2023 5:00	12	13.0	10.3	-2.7
Med- Surg ICU	3/9/2023 6:00	12	13.0	10.3	-2.7
Med- Surg ICU	3/9/2023 7:00	12	13.0	10.3	-2.7
Med- Surg ICU	3/9/2023 8:00	13	15.0	10.0	-5.0
Med- Surg ICU	3/9/2023 9:00	13	15.0	10.0	-5.0
Med- Surg ICU	3/9/2023 10:00	13	15.0	10.0	-5.0
Med- Surg ICU	3/9/2023 11:00	13	15.0	10.0	-5.0
Med- Surg ICU	3/9/2023 12:00	13	15.0	10.0	-5.0
Med- Surg ICU	3/9/2023 13:00	13	15.0	10.0	-5.0
Med- Surg ICU	3/9/2023 14:00	13	15.0	10.0	-5.0
Med- Surg ICU	3/9/2023 15:00	13	15.0	10.0	-5.0
Med- Surg ICU	3/9/2023 16:00	13	15.0	5.1	-9.9
Med- Surg ICU	3/9/2023 17:00	13	15.0	5.1	-9.9
Med- Surg ICU	3/9/2023 18:00	13	15.0	5.1	-9.9
Med- Surg ICU	3/9/2023 19:00	13	15.0	5.1	-9.9
Med- Surg ICU	3/9/2023 20:00	13	15.0	7.7	-7.3
Med- Surg ICU	3/9/2023 21:00	13	15.0	7.7	-7.3

Med- Surg ICU	3/9/2023 22:00	11	15.0	7.7	-7.3
Med- Surg ICU	3/9/2023 23:00	11	15.0	7.7	-7.3
Med- Surg ICU	3/10/2023 0:00	11	15.0	7.7	-7.3
Med- Surg ICU	3/10/2023 1:00	11	15.0	7.7	-7.3
Med- Surg ICU	3/10/2023 2:00	11	15.0	7.7	-7.3
Med- Surg ICU	3/10/2023 3:00	11	15.0	7.7	-7.3
Med- Surg ICU	3/10/2023 4:00	11	15.0	7.7	-7.3
Med- Surg ICU	3/10/2023 5:00	11	15.0	7.7	-7.3
Med- Surg ICU	3/10/2023 6:00	11	15.0	7.7	-7.3
Med- Surg ICU	3/10/2023 7:00	11	15.0	7.7	-7.3
Med- Surg ICU	3/10/2023 8:00	11	15.0	7.5	-7.5
Med- Surg ICU	3/10/2023 9:00	11	15.0	7.5	-7.5
Med- Surg ICU	3/10/2023 10:00	11	14.0	10.0	-4.0
Med- Surg ICU	3/10/2023 11:00	11	14.0	10.0	-4.0
Med- Surg ICU	3/10/2023 12:00	11	14.0	10.0	-4.0
Med- Surg ICU	3/10/2023 13:00	11	14.0	10.0	-4.0
Med- Surg ICU	3/10/2023 14:00	11	14.0	10.0	-4.0
Med- Surg ICU	3/10/2023 15:00	10	14.0	10.0	-4.0
Med- Surg ICU	3/10/2023 16:00	10	14.0	10.3	-3.7
Med- Surg ICU	3/10/2023 17:00	10	14.0	10.3	-3.7
Med- Surg ICU	3/10/2023 18:00	10	14.0	10.3	-3.7
Med- Surg ICU	3/10/2023 19:00	10	14.0	10.3	-3.7
Med- Surg ICU	3/10/2023 20:00	10	14.0	10.3	-3.7
Med- Surg ICU	3/10/2023 21:00	10	14.0	10.3	-3.7
Med- Surg ICU	3/10/2023 22:00	10	14.0	10.3	-3.7
Med- Surg ICU	3/10/2023 23:00	10	14.0	10.3	-3.7
Med- Surg ICU	3/11/2023 0:00	10	14.0	11.5	-2.5
Med- Surg ICU	3/11/2023 1:00	10	14.0	11.5	-2.5
Med- Surg ICU	3/11/2023 2:00	10	14.0	11.5	-2.5
Med- Surg ICU	3/11/2023 3:00	10	14.0	11.5	-2.5
Med- Surg ICU	3/11/2023 4:00	10	14.0	11.5	-2.5
Med- Surg ICU	3/11/2023 5:00	10	14.0	11.5	-2.5
Med- Surg ICU	3/11/2023 6:00	10	14.0	11.5	-2.5
Med- Surg ICU	3/11/2023 7:00	10	14.0	11.5	-2.5
Med- Surg ICU	3/11/2023 8:00	10	14.0	10.0	-4.0
Med- Surg ICU	3/11/2023 9:00	10	14.0	10.0	-4.0
Med- Surg ICU	3/11/2023 10:00	10	14.0	10.0	-4.0
Med- Surg ICU	3/11/2023 11:00	10	14.0	10.0	-4.0
Med- Surg ICU	3/11/2023 12:00	10	14.0	11.2	-2.8
Med- Surg ICU	3/11/2023 13:00	10	14.0	11.2	-2.8
Med- Surg ICU	3/11/2023 14:00	10	14.0	11.2	-2.8
Med- Surg ICU	3/11/2023 15:00	10	14.0	11.2	-2.8
Med- Surg ICU	3/11/2023 16:00	10	14.0	12.1	-1.9
Med- Surg ICU	3/11/2023 17:00	10	14.0	12.1	-1.9
Med- Surg ICU	3/11/2023 18:00	10	14.0	12.1	-1.9
Med- Surg ICU	3/11/2023 19:00	10	14.0	12.1	-1.9
Med- Surg ICU	3/11/2023 20:00	11	14.0	11.5	-2.5

Med- Surg ICU	3/11/2023 21:00	11	14.0	11.5	-2.5
Med- Surg ICU	3/11/2023 22:00	11	14.0	11.5	-2.5
Med- Surg ICU	3/11/2023 23:00	11	14.0	11.5	-2.5
Med- Surg ICU	3/12/2023 0:00	12	14.0	10.3	-3.7
Med- Surg ICU	3/12/2023 1:00	12	14.0	10.3	-3.7
Med- Surg ICU	3/12/2023 2:00	12	14.0	10.3	-3.7
Med- Surg ICU	3/12/2023 3:00	12	14.0	10.3	-3.7
Med- Surg ICU	3/12/2023 4:00	12	14.0	10.3	-3.7
Med- Surg ICU	3/12/2023 5:00	12	14.0	10.3	-3.7
Med- Surg ICU	3/12/2023 6:00	12	14.0	10.3	-3.7
Med- Surg ICU	3/12/2023 7:00	12	14.0	10.3	-3.7
Med- Surg ICU	3/12/2023 8:00	12	14.0	11.2	-2.8
Med- Surg ICU	3/12/2023 9:00	12	14.0	11.2	-2.8
Med- Surg ICU	3/12/2023 10:00	12	14.0	11.2	-2.8
Med- Surg ICU	3/12/2023 11:00	12	14.0	11.2	-2.8
Med- Surg ICU	3/12/2023 12:00	12	14.0	11.2	-2.8
Med- Surg ICU	3/12/2023 13:00	12	14.0	11.2	-2.8
Med- Surg ICU	3/12/2023 14:00	12	14.0	11.2	-2.8
Med- Surg ICU	3/12/2023 15:00	12	14.0	11.2	-2.8
Med- Surg ICU	3/12/2023 16:00	12	14.0	12.1	-1.9
Med- Surg ICU	3/12/2023 17:00	12	14.0	12.1	-1.9
Med- Surg ICU	3/12/2023 18:00	12	14.0	12.1	-1.9
Med- Surg ICU	3/12/2023 19:00	12	14.0	12.1	-1.9
Med- Surg ICU	3/12/2023 20:00	11	12.0	10.3	-1.7
Med- Surg ICU	3/12/2023 21:00	11	12.0	10.3	-1.7
Med- Surg ICU	3/12/2023 22:00	11	12.0	10.3	-1.7
Med- Surg ICU	3/12/2023 23:00	11	12.0	10.3	-1.7
Med- Surg ICU	3/13/2023 0:00	11	12.0	9.0	-3.0
Med- Surg ICU	3/13/2023 1:00	11	12.0	9.0	-3.0
Med- Surg ICU	3/13/2023 2:00	11	12.0	9.0	-3.0
Med- Surg ICU	3/13/2023 3:00	11	12.0	9.0	-3.0
Med- Surg ICU	3/13/2023 4:00	11	12.0	9.0	-3.0
Med- Surg ICU	3/13/2023 5:00	11	12.0	7.7	-4.3
Med- Surg ICU	3/13/2023 6:00	11	12.0	7.7	-4.3
Med- Surg ICU	3/13/2023 7:00	11	12.0	8.3	-3.7
Med- Surg ICU	3/13/2023 8:00	11	12.0	8.7	-3.3
Med- Surg ICU	3/13/2023 9:00	10	11.0	8.7	-2.3
Med- Surg ICU	3/13/2023 10:00	10	11.0	8.7	-2.3
Med- Surg ICU	3/13/2023 11:00	10	11.0	8.7	-2.3
Med- Surg ICU	3/13/2023 12:00	9	11.0	8.7	-2.3
Med- Surg ICU	3/13/2023 13:00	9	11.0	8.7	-2.3
Med- Surg ICU	3/13/2023 14:00	9	11.0	8.7	-2.3
Med- Surg ICU	3/13/2023 15:00	9	11.0	8.7	-2.3
Med- Surg ICU	3/13/2023 16:00	10	12.0	10.6	-1.4
Med- Surg ICU	3/13/2023 17:00	10	12.0	10.6	-1.4
Med- Surg ICU	3/13/2023 18:00	10	12.0	10.6	-1.4
Med- Surg ICU	3/13/2023 19:00	10	12.0	9.8	-2.2

Med- Surg ICU	3/13/2023 20:00	9	11.0	5.1	-5.9
Med- Surg ICU	3/13/2023 21:00	9	11.0	5.1	-5.9
Med- Surg ICU	3/13/2023 22:00	9	11.0	5.1	-5.9
Med- Surg ICU	3/13/2023 23:00	9	11.0	5.1	-5.9
Med- Surg ICU	3/14/2023 0:00	9	11.0	5.1	-5.9
Med- Surg ICU	3/14/2023 1:00	9	11.0	5.1	-5.9
Med- Surg ICU	3/14/2023 2:00	9	11.0	5.1	-5.9
Med- Surg ICU	3/14/2023 3:00	9	11.0	5.1	-5.9
Med- Surg ICU	3/14/2023 4:00	9	11.0	5.1	-5.9
Med- Surg ICU	3/14/2023 5:00	9	11.0	5.1	-5.9
Med- Surg ICU	3/14/2023 6:00	9	11.0	5.1	-5.9
Med- Surg ICU	3/14/2023 7:00	9	11.0	5.8	-5.2
Med- Surg ICU	3/14/2023 8:00	11	12.0	7.5	-4.5
Med- Surg ICU	3/14/2023 9:00	11	12.0	7.5	-4.5
Med- Surg ICU	3/14/2023 10:00	11	12.0	7.5	-4.5
Med- Surg ICU	3/14/2023 11:00	11	12.0	7.5	-4.5
Med- Surg ICU	3/14/2023 12:00	11	12.0	7.5	-4.5
Med- Surg ICU	3/14/2023 13:00	11	12.0	7.5	-4.5
Med- Surg ICU	3/14/2023 14:00	11	12.0	7.5	-4.5
Med- Surg ICU	3/14/2023 15:00	11	12.0	7.5	-4.5
Med- Surg ICU	3/14/2023 16:00	11	12.0	5.1	-6.9
Med- Surg ICU	3/14/2023 17:00	10	11.0	7.7	-3.3
Med- Surg ICU	3/14/2023 18:00	10	11.0	7.7	-3.3
Med- Surg ICU	3/14/2023 19:00	10	11.0	7.1	-4.0
Med- Surg ICU	3/14/2023 20:00	10	11.0	6.7	-4.3
Med- Surg ICU	3/14/2023 21:00	10	11.0	7.7	-3.3
Med- Surg ICU	3/14/2023 22:00	10	11.0	7.7	-3.3
Med- Surg ICU	3/14/2023 23:00	10	11.0	7.7	-3.3
Med- Surg ICU	3/15/2023 0:00	10	11.0	9.0	-2.0
Med- Surg ICU	3/15/2023 1:00	10	11.0	9.0	-2.0
Med- Surg ICU	3/15/2023 2:00	10	11.0	9.0	-2.0
Med- Surg ICU	3/15/2023 3:00	10	11.0	9.0	-2.0
Med- Surg ICU	3/15/2023 4:00	10	11.0	9.0	-2.0
Med- Surg ICU	3/15/2023 5:00	10	11.0	9.0	-2.0
Med- Surg ICU	3/15/2023 6:00	10	11.0	9.0	-2.0
Med- Surg ICU	3/15/2023 7:00	10	11.0	9.6	-1.4
Med- Surg ICU	3/15/2023 8:00	10	13.0	13.7	0.7
Med- Surg ICU	3/15/2023 9:00	10	13.0	13.7	0.7
Med- Surg ICU	3/15/2023 10:00	10	13.0	13.7	0.7
Med- Surg ICU	3/15/2023 11:00	10	13.0	13.7	0.7
Med- Surg ICU	3/15/2023 12:00	10	13.0	13.7	0.7
Med- Surg ICU	3/15/2023 13:00	10	13.0	13.7	0.7
Med- Surg ICU	3/15/2023 14:00	10	13.0	13.7	0.7
Med- Surg ICU	3/15/2023 15:00	10	13.0	13.1	0.1
Med- Surg ICU	3/15/2023 16:00	10	13.0	10.3	-2.7
Med- Surg ICU	3/15/2023 17:00	10	13.0	10.3	-2.7
Med- Surg ICU	3/15/2023 18:00	10	13.0	10.3	-2.7

Med- Surg ICU	3/15/2023 19:00	10	13.0	10.3	-2.7
Med- Surg ICU	3/15/2023 20:00	10	15.0	11.5	-3.5
Med- Surg ICU	3/15/2023 21:00	10	15.0	11.5	-3.5
Med- Surg ICU	3/15/2023 22:00	10	15.0	11.5	-3.5
Med- Surg ICU	3/15/2023 23:00	10	15.0	11.5	-3.5
Med- Surg ICU	3/16/2023 0:00	10	15.0	10.3	-4.7
Med- Surg ICU	3/16/2023 1:00	10	15.0	10.3	-4.7
Med- Surg ICU	3/16/2023 2:00	10	15.0	10.3	-4.7
Med- Surg ICU	3/16/2023 3:00	10	15.0	10.3	-4.7
Med- Surg ICU	3/16/2023 4:00	10	15.0	10.3	-4.7
Med- Surg ICU	3/16/2023 5:00	10	15.0	10.3	-4.7
Med- Surg ICU	3/16/2023 6:00	10	15.0	10.3	-4.7
Med- Surg ICU	3/16/2023 7:00	10	15.0	10.9	-4.1
Med- Surg ICU	3/16/2023 8:00	12	10.0	11.2	1.2
Med- Surg ICU	3/16/2023 9:00	12	10.0	11.2	1.2
Med- Surg ICU	3/16/2023 10:00	12	10.0	11.2	1.2
Med- Surg ICU	3/16/2023 11:00	12	10.0	11.2	1.2
Med- Surg ICU	3/16/2023 12:00	12	10.0	10.0	0.0
Med- Surg ICU	3/16/2023 13:00	12	10.0	10.0	0.0
Med- Surg ICU	3/16/2023 14:00	12	10.0	10.0	0.0
Med- Surg ICU	3/16/2023 15:00	12	10.0	10.0	0.0
Med- Surg ICU	3/16/2023 16:00	12	10.0	9.6	-0.4
Med- Surg ICU	3/16/2023 17:00	12	10.0	9.0	-1.0
Med- Surg ICU	3/16/2023 18:00	12	10.0	9.0	-1.0
Med- Surg ICU	3/16/2023 19:00	10	14.0	9.0	-5.0
Med- Surg ICU	3/16/2023 20:00	9	14.0	10.3	-3.7
Med- Surg ICU	3/16/2023 21:00	9	14.0	10.3	-3.7
Med- Surg ICU	3/16/2023 22:00	10	14.0	10.3	-3.7
Med- Surg ICU	3/16/2023 23:00	10	14.0	10.3	-3.7
Med- Surg ICU	3/17/2023 0:00	10	14.0	10.3	-3.7
Med- Surg ICU	3/17/2023 1:00	10	14.0	10.3	-3.7
Med- Surg ICU	3/17/2023 2:00	10	14.0	10.3	-3.7
Med- Surg ICU	3/17/2023 3:00	10	14.0	10.3	-3.7
Med- Surg ICU	3/17/2023 4:00	10	14.0	10.3	-3.7
Med- Surg ICU	3/17/2023 5:00	10	14.0	10.3	-3.7
Med- Surg ICU	3/17/2023 6:00	10	14.0	10.3	-3.7
Med- Surg ICU	3/17/2023 7:00	10	11.0	10.3	-0.7
Med- Surg ICU	3/17/2023 8:00	10	11.0	10.0	-1.0
Med- Surg ICU	3/17/2023 9:00	10	12.0	10.0	-2.0
Med- Surg ICU	3/17/2023 10:00	10	12.0	10.0	-2.0
Med- Surg ICU	3/17/2023 11:00	10	12.0	10.0	-2.0
Med- Surg ICU	3/17/2023 12:00	10	12.0	10.0	-2.0
Med- Surg ICU	3/17/2023 13:00	10	12.0	10.0	-2.0
Med- Surg ICU	3/17/2023 14:00	10	12.0	10.0	-2.0
Med- Surg ICU	3/17/2023 15:00	10	12.0	10.0	-2.0
Med- Surg ICU	3/17/2023 16:00	10	12.0	9.0	-3.0
Med- Surg ICU	3/17/2023 17:00	10	12.0	9.0	-3.0

Med- Surg ICU	3/17/2023 18:00	10	12.0	9.0	-3.0
Med- Surg ICU	3/17/2023 19:00	10	12.0	9.0	-3.0
Med- Surg ICU	3/17/2023 20:00	10	12.0	10.3	-1.7
Med- Surg ICU	3/17/2023 21:00	10	12.0	9.0	-3.0
Med- Surg ICU	3/17/2023 22:00	10	12.0	9.0	-3.0
Med- Surg ICU	3/17/2023 23:00	10	12.0	9.0	-3.0
Med- Surg ICU	3/18/2023 0:00	13	14.0	10.3	-3.7
Med- Surg ICU	3/18/2023 1:00	13	14.0	10.3	-3.7
Med- Surg ICU	3/18/2023 2:00	13	14.0	10.3	-3.7
Med- Surg ICU	3/18/2023 3:00	13	14.0	10.3	-3.7
Med- Surg ICU	3/18/2023 4:00	13	14.0	10.3	-3.7
Med- Surg ICU	3/18/2023 5:00	13	14.0	10.3	-3.7
Med- Surg ICU	3/18/2023 6:00	13	14.0	10.3	-3.7
Med- Surg ICU	3/18/2023 7:00	13	14.0	10.3	-3.7
Med- Surg ICU	3/18/2023 8:00	13	13.0	8.7	-4.3
Med- Surg ICU	3/18/2023 9:00	13	13.0	8.7	-4.3
Med- Surg ICU	3/18/2023 10:00	13	13.0	8.7	-4.3
Med- Surg ICU	3/18/2023 11:00	13	13.0	8.7	-4.3
Med- Surg ICU	3/18/2023 12:00	13	13.0	10.0	-3.0
Med- Surg ICU	3/18/2023 13:00	13	13.0	10.0	-3.0
Med- Surg ICU	3/18/2023 14:00	13	13.0	10.0	-3.0
Med- Surg ICU	3/18/2023 15:00	13	13.0	10.0	-3.0
Med- Surg ICU	3/18/2023 16:00	13	13.0	9.0	-4.0
Med- Surg ICU	3/18/2023 17:00	13	13.0	9.0	-4.0
Med- Surg ICU	3/18/2023 18:00	13	13.0	9.0	-4.0
Med- Surg ICU	3/18/2023 19:00	13	13.0	9.0	-4.0
Med- Surg ICU	3/18/2023 20:00	10	12.0	10.3	-1.7
Med- Surg ICU	3/18/2023 21:00	10	12.0	10.3	-1.7
Med- Surg ICU	3/18/2023 22:00	10	12.0	10.3	-1.7
Med- Surg ICU	3/18/2023 23:00	10	12.0	10.3	-1.7
Med- Surg ICU	3/19/2023 0:00	10	12.0	9.0	-3.0
Med- Surg ICU	3/19/2023 1:00	10	12.0	9.0	-3.0
Med- Surg ICU	3/19/2023 2:00	10	12.0	9.0	-3.0
Med- Surg ICU	3/19/2023 3:00	10	12.0	9.0	-3.0
Med- Surg ICU	3/19/2023 4:00	10	11.0	9.0	-2.0
Med- Surg ICU	3/19/2023 5:00	10	12.0	9.0	-3.0
Med- Surg ICU	3/19/2023 6:00	10	12.0	9.0	-3.0
Med- Surg ICU	3/19/2023 7:00	10	12.0	9.0	-3.0
Med- Surg ICU	3/19/2023 8:00	10	12.0	9.3	-2.7
Med- Surg ICU	3/19/2023 9:00	10	12.0	10.0	-2.0
Med- Surg ICU	3/19/2023 10:00	10	12.0	10.0	-2.0
Med- Surg ICU	3/19/2023 11:00	10	12.0	10.0	-2.0
Med- Surg ICU	3/19/2023 12:00	10	12.0	10.0	-2.0
Med- Surg ICU	3/19/2023 13:00	10	12.0	10.0	-2.0
Med- Surg ICU	3/19/2023 14:00	10	12.0	10.0	-2.0
Med- Surg ICU	3/19/2023 15:00	10	12.0	10.0	-2.0
Med- Surg ICU	3/19/2023 16:00	10	12.0	8.3	-3.7

Med- Surg ICU	3/19/2023 17:00	10	12.0	9.0	-3.0
Med- Surg ICU	3/19/2023 18:00	10	12.0	9.0	-3.0
Med- Surg ICU	3/19/2023 19:00	10	12.0	9.0	-3.0
Med- Surg ICU	3/19/2023 20:00	8	12.0	7.7	-4.3
Med- Surg ICU	3/19/2023 21:00	8	12.0	7.7	-4.3
Med- Surg ICU	3/19/2023 22:00	8	12.0	7.7	-4.3
Med- Surg ICU	3/19/2023 23:00	8	12.0	7.7	-4.3
Med- Surg ICU	3/20/2023 0:00	8	12.0	7.7	-4.3
Med- Surg ICU	3/20/2023 1:00	8	12.0	7.7	-4.3
Med- Surg ICU	3/20/2023 2:00	8	12.0	7.7	-4.3
Med- Surg ICU	3/20/2023 3:00	8	12.0	7.7	-4.3
Med- Surg ICU	3/20/2023 4:00	8	12.0	7.7	-4.3
Med- Surg ICU	3/20/2023 5:00	8	12.0	7.7	-4.3
Med- Surg ICU	3/20/2023 6:00	8	12.0	7.7	-4.3
Med- Surg ICU	3/20/2023 7:00	8	12.0	8.3	-3.7
Med- Surg ICU	3/20/2023 8:00	8	12.0	10.0	-2.0
Med- Surg ICU	3/20/2023 9:00	8	12.0	10.0	-2.0
Med- Surg ICU	3/20/2023 10:00	8	12.0	10.0	-2.0
Med- Surg ICU	3/20/2023 11:00	8	12.0	10.0	-2.0
Med- Surg ICU	3/20/2023 12:00	8	12.0	10.0	-2.0
Med- Surg ICU	3/20/2023 13:00	8	12.0	10.0	-2.0
Med- Surg ICU	3/20/2023 14:00	8	12.0	9.0	-3.0
Med- Surg ICU	3/20/2023 15:00	11	12.0	8.4	-3.6
Med- Surg ICU	3/20/2023 16:00	11	12.0	9.0	-3.0
Med- Surg ICU	3/20/2023 17:00	11	12.0	9.0	-3.0
Med- Surg ICU	3/20/2023 18:00	11	12.0	9.0	-3.0
Med- Surg ICU	3/20/2023 19:00	11	12.0	9.0	-3.0
Med- Surg ICU	3/20/2023 20:00	11	11.0	9.0	-2.0
Med- Surg ICU	3/20/2023 21:00	11	11.0	9.0	-2.0
Med- Surg ICU	3/20/2023 22:00	11	11.0	9.0	-2.0
Med- Surg ICU	3/20/2023 23:00	11	11.0	9.0	-2.0
Med- Surg ICU	3/21/2023 0:00	11	11.0	9.0	-2.0
Med- Surg ICU	3/21/2023 1:00	11	11.0	9.0	-2.0
Med- Surg ICU	3/21/2023 2:00	11	11.0	9.0	-2.0
Med- Surg ICU	3/21/2023 3:00	11	11.0	9.0	-2.0
Med- Surg ICU	3/21/2023 4:00	11	11.0	9.0	-2.0
Med- Surg ICU	3/21/2023 5:00	11	11.0	9.0	-2.0
Med- Surg ICU	3/21/2023 6:00	11	11.0	9.0	-2.0
Med- Surg ICU	3/21/2023 7:00	11	11.0	9.6	-1.4
Med- Surg ICU	3/21/2023 8:00	11	11.0	10.0	-1.0
Med- Surg ICU	3/21/2023 9:00	11	12.0	11.2	-0.8
Med- Surg ICU	3/21/2023 10:00	11	12.0	11.2	-0.8
Med- Surg ICU	3/21/2023 11:00	11	12.0	11.2	-0.8
Med- Surg ICU	3/21/2023 12:00	11	12.0	11.2	-0.8
Med- Surg ICU	3/21/2023 13:00	11	12.0	11.2	-0.8
Med- Surg ICU	3/21/2023 14:00	11	12.0	11.2	-0.8
Med- Surg ICU	3/21/2023 15:00	10	11.0	11.2	0.2

Med- Surg ICU	3/21/2023 16:00	11	11.0	9.0	-2.0
Med- Surg ICU	3/21/2023 17:00	11	11.0	9.0	-2.0
Med- Surg ICU	3/21/2023 18:00	11	11.0	9.0	-2.0
Med- Surg ICU	3/21/2023 19:00	11	11.0	8.3	-2.7
Med- Surg ICU	3/21/2023 20:00	11	11.0	10.3	-0.7
Med- Surg ICU	3/21/2023 21:00	11	11.0	10.3	-0.7
Med- Surg ICU	3/21/2023 22:00	11	11.0	10.3	-0.7
Med- Surg ICU	3/21/2023 23:00	11	11.0	10.3	-0.7
Med- Surg ICU	3/22/2023 0:00	12	12.0	11.5	-0.5
Med- Surg ICU	3/22/2023 1:00	12	12.0	11.5	-0.5
Med- Surg ICU	3/22/2023 2:00	12	12.0	11.5	-0.5
Med- Surg ICU	3/22/2023 3:00	12	12.0	11.5	-0.5
Med- Surg ICU	3/22/2023 4:00	12	12.0	11.5	-0.5
Med- Surg ICU	3/22/2023 5:00	12	12.0	11.5	-0.5
Med- Surg ICU	3/22/2023 6:00	12	12.0	11.5	-0.5
Med- Surg ICU	3/22/2023 7:00	12	12.0	12.2	0.2
Med- Surg ICU	3/22/2023 8:00	12	12.0	11.2	-0.8
Med- Surg ICU	3/22/2023 9:00	12	12.0	11.2	-0.8
Med- Surg ICU	3/22/2023 10:00	12	12.0	11.2	-0.8
Med- Surg ICU	3/22/2023 11:00	12	12.0	11.2	-0.8
Med- Surg ICU	3/22/2023 12:00	12	12.0	11.2	-0.8
Med- Surg ICU	3/22/2023 13:00	12	12.0	11.2	-0.8
Med- Surg ICU	3/22/2023 14:00	12	12.0	11.2	-0.8
Med- Surg ICU	3/22/2023 15:00	12	12.0	11.2	-0.8
Med- Surg ICU	3/22/2023 16:00	12	12.0	10.3	-1.7
Med- Surg ICU	3/22/2023 17:00	12	12.0	10.3	-1.7
Med- Surg ICU	3/22/2023 18:00	12	12.0	10.3	-1.7
Med- Surg ICU	3/22/2023 19:00	12	12.0	9.6	-2.4
Med- Surg ICU	3/22/2023 20:00	9	12.0	9.0	-3.0
Med- Surg ICU	3/22/2023 21:00	9	12.0	9.0	-3.0
Med- Surg ICU	3/22/2023 22:00	9	12.0	9.0	-3.0
Med- Surg ICU	3/22/2023 23:00	9	12.0	9.0	-3.0
Med- Surg ICU	3/23/2023 0:00	9	12.0	10.3	-1.7
Med- Surg ICU	3/23/2023 1:00	9	12.0	10.3	-1.7
Med- Surg ICU	3/23/2023 2:00	9	12.0	10.3	-1.7
Med- Surg ICU	3/23/2023 3:00	9	12.0	10.3	-1.7
Med- Surg ICU	3/23/2023 4:00	9	12.0	11.5	-0.5
Med- Surg ICU	3/23/2023 5:00	9	12.0	11.5	-0.5
Med- Surg ICU	3/23/2023 6:00	9	12.0	11.5	-0.5
Med- Surg ICU	3/23/2023 7:00	9	12.0	12.2	0.2
Med- Surg ICU	3/23/2023 8:00	9	12.0	13.7	1.7
Med- Surg ICU	3/23/2023 9:00	9	12.0	13.7	1.7
Med- Surg ICU	3/23/2023 10:00	9	12.0	13.7	1.7
Med- Surg ICU	3/23/2023 11:00	9	12.0	13.7	1.7
Med- Surg ICU	3/23/2023 12:00	9	12.0	13.7	1.7
Med- Surg ICU	3/23/2023 13:00	9	12.0	13.7	1.7
Med- Surg ICU	3/23/2023 14:00	9	12.0	13.7	1.7

Med- Surg ICU	3/23/2023 15:00	9	12.0	13.1	1.1
Med- Surg ICU	3/23/2023 16:00	9	12.0	9.0	-3.0
Med- Surg ICU	3/23/2023 17:00	9	12.0	9.0	-3.0
Med- Surg ICU	3/23/2023 18:00	9	10.0	9.0	-1.0
Med- Surg ICU	3/23/2023 19:00	9	10.0	9.0	-1.0
Med- Surg ICU	3/23/2023 20:00	9	10.0	6.4	-3.6
Med- Surg ICU	3/23/2023 21:00	9	10.0	6.4	-3.6
Med- Surg ICU	3/23/2023 22:00	10	13.0	6.4	-6.6
Med- Surg ICU	3/23/2023 23:00	10	13.0	6.4	-6.6
Med- Surg ICU	3/24/2023 0:00	10	13.0	7.6	-5.5
Med- Surg ICU	3/24/2023 1:00	10	13.0	7.6	-5.5
Med- Surg ICU	3/24/2023 2:00	10	13.0	7.6	-5.5
Med- Surg ICU	3/24/2023 3:00	10	13.0	7.6	-5.5
Med- Surg ICU	3/24/2023 4:00	10	13.0	7.6	-5.5
Med- Surg ICU	3/24/2023 5:00	10	13.0	7.6	-5.5
Med- Surg ICU	3/24/2023 6:00	10	13.0	7.6	-5.5
Med- Surg ICU	3/24/2023 7:00	10	13.0	7.6	-5.5
Med- Surg ICU	3/24/2023 8:00	9	12.0	7.3	-4.7
Med- Surg ICU	3/24/2023 9:00	9	12.0	7.3	-4.7
Med- Surg ICU	3/24/2023 10:00	9	12.0	7.3	-4.7
Med- Surg ICU	3/24/2023 11:00	9	12.0	7.3	-4.7
Med- Surg ICU	3/24/2023 12:00	9	12.0	7.3	-4.7
Med- Surg ICU	3/24/2023 13:00	9	12.0	7.3	-4.7
Med- Surg ICU	3/24/2023 14:00	9	12.0	7.3	-4.7
Med- Surg ICU	3/24/2023 15:00	9	12.0	7.3	-4.7
Med- Surg ICU	3/24/2023 16:00	9	10.0	5.1	-4.9
Med- Surg ICU	3/24/2023 17:00	9	10.0	5.1	-4.9
Med- Surg ICU	3/24/2023 18:00	9	10.0	5.1	-4.9
Med- Surg ICU	3/24/2023 19:00	9	12.0	7.7	-4.3
Med- Surg ICU	3/24/2023 20:00	9	12.0	9.0	-3.0
Med- Surg ICU	3/24/2023 21:00	10	12.0	9.0	-3.0
Med- Surg ICU	3/24/2023 22:00	10	12.0	9.0	-3.0
Med- Surg ICU	3/24/2023 23:00	10	12.0	9.0	-3.0
Med- Surg ICU	3/25/2023 0:00	10	12.0	10.3	-1.7
Med- Surg ICU	3/25/2023 1:00	10	12.0	10.3	-1.7
Med- Surg ICU	3/25/2023 2:00	10	12.0	10.3	-1.7
Med- Surg ICU	3/25/2023 3:00	10	12.0	10.3	-1.7
Med- Surg ICU	3/25/2023 4:00	10	12.0	10.3	-1.7
Med- Surg ICU	3/25/2023 5:00	20	12.0	10.3	-1.7
Med- Surg ICU	3/25/2023 6:00	20	12.0	10.3	-1.7
Med- Surg ICU	3/25/2023 7:00	20	12.0	10.3	-1.7
Med- Surg ICU	3/25/2023 8:00	20	12.0	11.2	-0.8
Med- Surg ICU	3/25/2023 9:00	20	12.0	11.2	-0.8
Med- Surg ICU	3/25/2023 10:00	20	12.0	11.2	-0.8
Med- Surg ICU	3/25/2023 11:00	20	12.0	11.2	-0.8
Med- Surg ICU	3/25/2023 12:00	11	14.0	0.0	-14.0
Med- Surg ICU	3/25/2023 13:00	11	14.0	0.0	-14.0

Med- Surg ICU	3/25/2023 14:00	11	14.0	0.0	-14.0
Med- Surg ICU	3/25/2023 15:00	11	14.0	0.0	-14.0
Med- Surg ICU	3/25/2023 16:00	11	14.0	0.0	-14.0
Med- Surg ICU	3/25/2023 17:00	11	14.0	0.0	-14.0
Med- Surg ICU	3/25/2023 18:00	11	14.0	0.0	-14.0
Med- Surg ICU	3/25/2023 19:00	8	13.0	0.0	-13.0
Med- Surg ICU	3/25/2023 20:00	8	14.0	9.0	-5.0
Med- Surg ICU	3/25/2023 21:00	8	14.0	9.0	-5.0
Med- Surg ICU	3/25/2023 22:00	8	14.0	9.0	-5.0
Med- Surg ICU	3/25/2023 23:00	8	14.0	9.0	-5.0
Med- Surg ICU	3/26/2023 0:00	8	14.0	10.3	-3.7
Med- Surg ICU	3/26/2023 1:00	8	14.0	10.3	-3.7
Med- Surg ICU	3/26/2023 2:00	8	14.0	10.3	-3.7
Med- Surg ICU	3/26/2023 3:00	8	14.0	10.3	-3.7
Med- Surg ICU	3/26/2023 4:00	8	14.0	10.3	-3.7
Med- Surg ICU	3/26/2023 5:00	8	14.0	10.3	-3.7
Med- Surg ICU	3/26/2023 6:00	8	14.0	10.3	-3.7
Med- Surg ICU	3/26/2023 7:00	8	14.0	10.3	-3.7
Med- Surg ICU	3/26/2023 8:00	8	10.0	10.0	0.0
Med- Surg ICU	3/26/2023 9:00	8	10.0	10.0	0.0
Med- Surg ICU	3/26/2023 10:00	8	10.0	10.0	0.0
Med- Surg ICU	3/26/2023 11:00	8	10.0	10.0	0.0
Med- Surg ICU	3/26/2023 12:00	8	10.0	10.0	0.0
Med- Surg ICU	3/26/2023 13:00	8	10.0	10.0	0.0
Med- Surg ICU	3/26/2023 14:00	8	10.0	10.0	0.0
Med- Surg ICU	3/26/2023 15:00	8	10.0	10.0	0.0
Med- Surg ICU	3/26/2023 16:00	8	10.0	9.0	-1.0
Med- Surg ICU	3/26/2023 17:00	8	10.0	9.0	-1.0
Med- Surg ICU	3/26/2023 18:00	8	10.0	9.0	-1.0
Med- Surg ICU	3/26/2023 19:00	8	10.0	9.0	-1.0
Med- Surg ICU	3/26/2023 20:00	7	14.0	9.0	-5.0
Med- Surg ICU	3/26/2023 21:00	7	14.0	9.0	-5.0
Med- Surg ICU	3/26/2023 22:00	7	14.0	9.0	-5.0
Med- Surg ICU	3/26/2023 23:00	7	14.0	9.0	-5.0
Med- Surg ICU	3/27/2023 0:00	7	14.0	7.7	-6.3
Med- Surg ICU	3/27/2023 1:00	7	14.0	7.7	-6.3
Med- Surg ICU	3/27/2023 2:00	7	14.0	7.7	-6.3
Med- Surg ICU	3/27/2023 3:00	7	14.0	7.7	-6.3
Med- Surg ICU	3/27/2023 4:00	7	14.0	7.7	-6.3
Med- Surg ICU	3/27/2023 5:00	7	14.0	7.7	-6.3
Med- Surg ICU	3/27/2023 6:00	7	14.0	7.7	-6.3
Med- Surg ICU	3/27/2023 7:00	7	14.0	8.3	-5.7
Med- Surg ICU	3/27/2023 8:00	7	14.0	12.5	-1.6
Med- Surg ICU	3/27/2023 9:00	7	14.0	12.5	-1.6
Med- Surg ICU	3/27/2023 10:00	7	14.0	12.5	-1.6
Med- Surg ICU	3/27/2023 11:00	7	14.0	12.5	-1.6
Med- Surg ICU	3/27/2023 12:00	7	14.0	12.5	-1.6

Med- Surg ICU	3/27/2023 13:00	7	14.0	11.2	-2.8
Med- Surg ICU	3/27/2023 14:00	7	14.0	11.2	-2.8
Med- Surg ICU	3/27/2023 15:00	14	7.0	11.2	4.2
Med- Surg ICU	3/27/2023 16:00	14	7.0	12.1	5.1
Med- Surg ICU	3/27/2023 17:00	14	7.0	12.1	5.1
Med- Surg ICU	3/27/2023 18:00	14	7.0	12.1	5.1
Med- Surg ICU	3/27/2023 19:00	14	7.0	11.3	4.3
Med- Surg ICU	3/27/2023 20:00	7	14.0	7.7	-6.3
Med- Surg ICU	3/27/2023 21:00	7	14.0	7.7	-6.3
Med- Surg ICU	3/27/2023 22:00	7	14.0	9.0	-5.0
Med- Surg ICU	3/27/2023 23:00	7	14.0	9.0	-5.0
Med- Surg ICU	3/28/2023 0:00	7	14.0	6.4	-7.6
Med- Surg ICU	3/28/2023 1:00	7	14.0	6.4	-7.6
Med- Surg ICU	3/28/2023 2:00	7	14.0	6.4	-7.6
Med- Surg ICU	3/28/2023 3:00	7	14.0	6.4	-7.6
Med- Surg ICU	3/28/2023 4:00	7	14.0	6.4	-7.6
Med- Surg ICU	3/28/2023 5:00	7	14.0	6.4	-7.6
Med- Surg ICU	3/28/2023 6:00	7	14.0	5.5	-8.6
Med- Surg ICU	3/28/2023 7:00	8	14.0	6.4	-7.6
Med- Surg ICU	3/28/2023 8:00	8	14.0	8.7	-5.3
Med- Surg ICU	3/28/2023 9:00	8	14.0	8.7	-5.3
Med- Surg ICU	3/28/2023 10:00	8	14.0	8.7	-5.3
Med- Surg ICU	3/28/2023 11:00	8	14.0	8.7	-5.3
Med- Surg ICU	3/28/2023 12:00	8	14.0	8.7	-5.3
Med- Surg ICU	3/28/2023 13:00	8	14.0	8.7	-5.3
Med- Surg ICU	3/28/2023 14:00	8	14.0	8.7	-5.3
Med- Surg ICU	3/28/2023 15:00	8	14.0	8.7	-5.3
Med- Surg ICU	3/28/2023 16:00	8	14.0	7.7	-6.3
Med- Surg ICU	3/28/2023 17:00	8	14.0	7.7	-6.3
Med- Surg ICU	3/28/2023 18:00	8	14.0	7.7	-6.3
Med- Surg ICU	3/28/2023 19:00	11	14.0	7.7	-6.3
Med- Surg ICU	3/28/2023 20:00	11	13.0	7.7	-5.3
Med- Surg ICU	3/28/2023 21:00	11	13.0	7.7	-5.3
Med- Surg ICU	3/28/2023 22:00	11	13.0	7.7	-5.3
Med- Surg ICU	3/28/2023 23:00	11	13.0	7.7	-5.3
Med- Surg ICU	3/29/2023 0:00	11	13.0	9.6	-3.4
Med- Surg ICU	3/29/2023 1:00	11	13.0	10.3	-2.7
Med- Surg ICU	3/29/2023 2:00	11	13.0	10.3	-2.7
Med- Surg ICU	3/29/2023 3:00	11	13.0	10.3	-2.7
Med- Surg ICU	3/29/2023 4:00	11	13.0	10.3	-2.7
Med- Surg ICU	3/29/2023 5:00	11	13.0	10.3	-2.7
Med- Surg ICU	3/29/2023 6:00	11	13.0	10.3	-2.7
Med- Surg ICU	3/29/2023 7:00	11	13.0	10.9	-2.1
Med- Surg ICU	3/29/2023 8:00	11	13.0	12.5	-0.6
Med- Surg ICU	3/29/2023 9:00	11	13.0	12.5	-0.6
Med- Surg ICU	3/29/2023 10:00	11	13.0	12.5	-0.6
Med- Surg ICU	3/29/2023 11:00	11	13.0	11.5	-1.5

Med- Surg ICU	3/29/2023 12:00	11	13.0	12.5	-0.6
Med- Surg ICU	3/29/2023 13:00	11	13.0	12.5	-0.6
Med- Surg ICU	3/29/2023 14:00	11	13.0	12.5	-0.6
Med- Surg ICU	3/29/2023 15:00	5	13.0	11.8	-1.2
Med- Surg ICU	3/29/2023 16:00	10	12.0	9.0	-3.0
Med- Surg ICU	3/29/2023 17:00	10	12.0	9.0	-3.0
Med- Surg ICU	3/29/2023 18:00	10	12.0	9.0	-3.0
Med- Surg ICU	3/29/2023 19:00	10	12.0	9.6	-2.4
Med- Surg ICU	3/29/2023 20:00	9	10.0	9.0	-1.0
Med- Surg ICU	3/29/2023 21:00	9	10.0	9.0	-1.0
Med- Surg ICU	3/29/2023 22:00	9	10.0	9.0	-1.0
Med- Surg ICU	3/29/2023 23:00	9	10.0	9.0	-1.0
Med- Surg ICU	3/30/2023 0:00	9	10.0	10.3	0.3
Med- Surg ICU	3/30/2023 1:00	9	10.0	10.3	0.3
Med- Surg ICU	3/30/2023 2:00	9	10.0	10.3	0.3
Med- Surg ICU	3/30/2023 3:00	9	10.0	10.3	0.3
Med- Surg ICU	3/30/2023 4:00	9	10.0	10.3	0.3
Med- Surg ICU	3/30/2023 5:00	9	10.0	10.3	0.3
Med- Surg ICU	3/30/2023 6:00	9	10.0	9.3	-0.7
Med- Surg ICU	3/30/2023 7:00	9	10.0	10.9	0.9
Med- Surg ICU	3/30/2023 8:00	10	10.0	0.0	-10.0
Med- Surg ICU	3/30/2023 9:00	10	10.0	0.0	-10.0
Med- Surg ICU	3/30/2023 10:00	10	10.0	0.0	-10.0
Med- Surg ICU	3/30/2023 11:00	10	10.0	0.0	-10.0
Med- Surg ICU	3/30/2023 12:00	10	10.0	0.0	-10.0
Med- Surg ICU	3/30/2023 13:00	10	10.0	0.0	-10.0
Med- Surg ICU	3/30/2023 14:00	10	10.0	0.0	-10.0
Med- Surg ICU	3/30/2023 15:00	10	10.0	0.0	-10.0
Med- Surg ICU	3/30/2023 16:00	10	10.0	0.0	-10.0
Med- Surg ICU	3/30/2023 17:00	10	10.0	0.0	-10.0
Med- Surg ICU	3/30/2023 18:00	10	10.0	0.0	-10.0
Med- Surg ICU	3/30/2023 19:00	10	10.0	0.0	-10.0
Med- Surg ICU	3/30/2023 20:00	9	9.0	9.0	0.0
Med- Surg ICU	3/30/2023 21:00	9	9.0	9.0	0.0
Med- Surg ICU	3/30/2023 22:00	9	9.0	9.0	0.0
Med- Surg ICU	3/30/2023 23:00	9	9.0	9.0	0.0
Med- Surg ICU	3/31/2023 0:00	9	9.0	10.3	1.3
Med- Surg ICU	3/31/2023 1:00	9	9.0	10.3	1.3
Med- Surg ICU	3/31/2023 2:00	9	9.0	10.3	1.3
Med- Surg ICU	3/31/2023 3:00	9	9.0	10.3	1.3
Med- Surg ICU	3/31/2023 4:00	9	9.0	10.3	1.3
Med- Surg ICU	3/31/2023 5:00	9	9.0	10.3	1.3
Med- Surg ICU	3/31/2023 6:00	9	9.0	10.3	1.3
Med- Surg ICU	3/31/2023 7:00	9	9.0	10.3	1.3
Med- Surg ICU	3/31/2023 8:00	9	9.0	8.7	-0.3
Med- Surg ICU	3/31/2023 9:00	9	9.0	8.7	-0.3
Med- Surg ICU	3/31/2023 10:00	9	9.0	8.7	-0.3

Med- Surg ICU	3/31/2023 11:00	9	9.0	8.7	-0.3
Med- Surg ICU	3/31/2023 12:00	9	9.0	8.7	-0.3
Med- Surg ICU	3/31/2023 13:00	9	9.0	8.7	-0.3
Med- Surg ICU	3/31/2023 14:00	6	10.0	8.7	-1.3
Med- Surg ICU	3/31/2023 15:00	6	10.0	8.7	-1.3
Med- Surg ICU	3/31/2023 16:00	6	10.0	7.7	-2.3
Med- Surg ICU	3/31/2023 17:00	6	10.0	7.7	-2.3
Med- Surg ICU	3/31/2023 18:00	6	10.0	7.7	-2.3
Med- Surg ICU	3/31/2023 19:00	6	10.0	7.7	-2.3
Med- Surg ICU	3/31/2023 20:00	8	9.0	9.0	0.0
Med- Surg ICU	3/31/2023 21:00	8	9.0	9.0	0.0
Med- Surg ICU	3/31/2023 22:00	8	9.0	9.0	0.0
Med- Surg ICU	3/31/2023 23:00	8	9.0	9.0	0.0
Med- Surg ICU	4/1/2023 0:00	8	9.0	10.3	1.3
Med- Surg ICU	4/1/2023 1:00	8	9.0	10.3	1.3
Med- Surg ICU	4/1/2023 2:00	8	9.0	10.3	1.3
Med- Surg ICU	4/1/2023 3:00	8	9.0	10.3	1.3
Med- Surg ICU	4/1/2023 4:00	8	9.0	10.3	1.3
Med- Surg ICU	4/1/2023 5:00	8	9.0	10.3	1.3
Med- Surg ICU	4/1/2023 6:00	8	9.0	10.3	1.3
Med- Surg ICU	4/1/2023 7:00	8	9.0	10.3	1.3
Med- Surg ICU	4/1/2023 8:00	7	7.0	7.5	0.5
Med- Surg ICU	4/1/2023 9:00	7	7.0	7.5	0.5
Med- Surg ICU	4/1/2023 10:00	7	7.0	7.5	0.5
Med- Surg ICU	4/1/2023 11:00	7	7.0	7.5	0.5
Med- Surg ICU	4/1/2023 12:00	7	7.0	7.5	0.5
Med- Surg ICU	4/1/2023 13:00	7	7.0	7.5	0.5
Med- Surg ICU	4/1/2023 14:00	7	7.0	7.5	0.5
Med- Surg ICU	4/1/2023 15:00	8	8.0	7.5	-0.5
Med- Surg ICU	4/1/2023 16:00	8	8.0	9.0	1.0
Med- Surg ICU	4/1/2023 17:00	8	8.0	9.0	1.0
Med- Surg ICU	4/1/2023 18:00	8	8.0	9.0	1.0
Med- Surg ICU	4/1/2023 19:00	8	10.0	6.4	-3.6
Med- Surg ICU	4/1/2023 20:00	8	10.0	7.7	-2.3
Med- Surg ICU	4/1/2023 21:00	8	10.0	7.7	-2.3
Med- Surg ICU	4/1/2023 22:00	8	10.0	7.7	-2.3
Med- Surg ICU	4/1/2023 23:00	8	10.0	7.7	-2.3
Med- Surg ICU	4/2/2023 0:00	8	10.0	6.4	-3.6
Med- Surg ICU	4/2/2023 1:00	8	10.0	6.4	-3.6
Med- Surg ICU	4/2/2023 2:00	8	10.0	6.4	-3.6
Med- Surg ICU	4/2/2023 3:00	8	10.0	6.4	-3.6
Med- Surg ICU	4/2/2023 4:00	8	10.0	6.4	-3.6
Med- Surg ICU	4/2/2023 5:00	8	10.0	6.4	-3.6
Med- Surg ICU	4/2/2023 6:00	8	10.0	6.4	-3.6
Med- Surg ICU	4/2/2023 7:00	8	10.0	6.4	-3.6
Med- Surg ICU	4/2/2023 8:00	9	10.0	6.2	-3.8
Med- Surg ICU	4/2/2023 9:00	9	10.0	6.2	-3.8

Med- Surg ICU	4/2/2023 10:00	9	10.0	6.2	-3.8
Med- Surg ICU	4/2/2023 11:00	9	10.0	6.2	-3.8
Med- Surg ICU	4/2/2023 12:00	9	10.0	6.2	-3.8
Med- Surg ICU	4/2/2023 13:00	10	10.0	8.7	-1.3
Med- Surg ICU	4/2/2023 14:00	10	10.0	8.7	-1.3
Med- Surg ICU	4/2/2023 15:00	10	10.0	8.7	-1.3
Med- Surg ICU	4/2/2023 16:00	10	10.0	9.0	-1.0
Med- Surg ICU	4/2/2023 17:00	10	10.0	9.0	-1.0
Med- Surg ICU	4/2/2023 18:00	10	10.0	9.0	-1.0
Med- Surg ICU	4/2/2023 19:00	10	10.0	9.0	-1.0
Med- Surg ICU	4/2/2023 20:00	11	10.0	9.0	-1.0
Med- Surg ICU	4/2/2023 21:00	11	10.0	9.0	-1.0
Med- Surg ICU	4/2/2023 22:00	11	10.0	9.0	-1.0
Med- Surg ICU	4/2/2023 23:00	11	10.0	9.0	-1.0
Med- Surg ICU	4/3/2023 0:00	11	10.0	9.0	-1.0
Med- Surg ICU	4/3/2023 1:00	11	10.0	9.0	-1.0
Med- Surg ICU	4/3/2023 2:00	11	10.0	9.0	-1.0
Med- Surg ICU	4/3/2023 3:00	11	10.0	9.0	-1.0
Med- Surg ICU	4/3/2023 4:00	11	10.0	9.0	-1.0
Med- Surg ICU	4/3/2023 5:00	11	10.0	9.0	-1.0
Med- Surg ICU	4/3/2023 6:00	11	10.0	9.0	-1.0
Med- Surg ICU	4/3/2023 7:00	11	10.0	9.6	-0.4
Med- Surg ICU	4/3/2023 8:00	10	13.0	12.5	-0.6
Med- Surg ICU	4/3/2023 9:00	10	13.0	13.7	0.7
Med- Surg ICU	4/3/2023 10:00	10	13.0	13.7	0.7
Med- Surg ICU	4/3/2023 11:00	10	13.0	13.7	0.7
Med- Surg ICU	4/3/2023 12:00	10	13.0	13.7	0.7
Med- Surg ICU	4/3/2023 13:00	10	13.0	12.5	-0.6
Med- Surg ICU	4/3/2023 14:00	10	11.0	12.5	1.5
Med- Surg ICU	4/3/2023 15:00	10	11.0	11.8	0.8
Med- Surg ICU	4/3/2023 16:00	10	11.0	11.5	0.5
Med- Surg ICU	4/3/2023 17:00	10	11.0	11.5	0.5
Med- Surg ICU	4/3/2023 18:00	10	11.0	11.5	0.5
Med- Surg ICU	4/3/2023 19:00	10	11.0	11.5	0.5
Med- Surg ICU	4/3/2023 20:00	10	11.0	10.3	-0.7
Med- Surg ICU	4/3/2023 21:00	10	11.0	10.3	-0.7
Med- Surg ICU	4/3/2023 22:00	11	11.0	10.3	-0.7
Med- Surg ICU	4/3/2023 23:00	11	11.0	10.3	-0.7
Med- Surg ICU	4/4/2023 0:00	11	11.0	9.0	-2.0
Med- Surg ICU	4/4/2023 1:00	11	11.0	9.0	-2.0
Med- Surg ICU	4/4/2023 2:00	11	11.0	9.0	-2.0
Med- Surg ICU	4/4/2023 3:00	11	11.0	9.0	-2.0
Med- Surg ICU	4/4/2023 4:00	11	11.0	9.0	-2.0
Med- Surg ICU	4/4/2023 5:00	11	11.0	9.0	-2.0
Med- Surg ICU	4/4/2023 6:00	11	11.0	9.0	-2.0
Med- Surg ICU	4/4/2023 7:00	11	11.0	9.6	-1.4
Med- Surg ICU	4/4/2023 8:00	11	11.0	11.2	0.2

Med- Surg ICU	4/4/2023 9:00	11	11.0	11.2	0.2
Med- Surg ICU	4/4/2023 10:00	11	11.0	11.2	0.2
Med- Surg ICU	4/4/2023 11:00	11	11.0	11.2	0.2
Med- Surg ICU	4/4/2023 12:00	11	11.0	11.2	0.2
Med- Surg ICU	4/4/2023 13:00	11	11.0	11.2	0.2
Med- Surg ICU	4/4/2023 14:00	11	11.0	11.2	0.2
Med- Surg ICU	4/4/2023 15:00	11	11.0	10.6	-0.4
Med- Surg ICU	4/4/2023 16:00	11	11.0	11.5	0.5
Med- Surg ICU	4/4/2023 17:00	11	11.0	11.5	0.5
Med- Surg ICU	4/4/2023 18:00	11	11.0	11.5	0.5
Med- Surg ICU	4/4/2023 19:00	11	11.0	11.5	0.5
Med- Surg ICU	4/4/2023 20:00	12	13.0	11.5	-1.5
Med- Surg ICU	4/4/2023 21:00	12	13.0	11.5	-1.5
Med- Surg ICU	4/4/2023 22:00	12	13.0	11.5	-1.5
Med- Surg ICU	4/4/2023 23:00	12	13.0	11.5	-1.5
Med- Surg ICU	4/5/2023 0:00	12	13.0	10.3	-2.7
Med- Surg ICU	4/5/2023 1:00	12	13.0	10.3	-2.7
Med- Surg ICU	4/5/2023 2:00	12	13.0	10.3	-2.7
Med- Surg ICU	4/5/2023 3:00	12	13.0	10.3	-2.7
Med- Surg ICU	4/5/2023 4:00	12	13.0	10.3	-2.7
Med- Surg ICU	4/5/2023 5:00	12	13.0	10.3	-2.7
Med- Surg ICU	4/5/2023 6:00	12	13.0	10.3	-2.7
Med- Surg ICU	4/5/2023 7:00	12	13.0	10.9	-2.1
Med- Surg ICU	4/5/2023 8:00	12	13.0	13.7	0.7
Med- Surg ICU	4/5/2023 9:00	12	13.0	13.7	0.7
Med- Surg ICU	4/5/2023 10:00	12	13.0	13.7	0.7
Med- Surg ICU	4/5/2023 11:00	12	13.0	13.7	0.7
Med- Surg ICU	4/5/2023 12:00	12	13.0	12.5	-0.6
Med- Surg ICU	4/5/2023 13:00	12	13.0	12.5	-0.6
Med- Surg ICU	4/5/2023 14:00	12	13.0	12.5	-0.6
Med- Surg ICU	4/5/2023 15:00	12	13.0	11.8	-1.2
Med- Surg ICU	4/5/2023 16:00	12	13.0	10.3	-2.7
Med- Surg ICU	4/5/2023 17:00	12	13.0	10.3	-2.7
Med- Surg ICU	4/5/2023 18:00	12	13.0	10.3	-2.7
Med- Surg ICU	4/5/2023 19:00	12	13.0	9.6	-3.4
Med- Surg ICU	4/5/2023 20:00	12	11.0	10.3	-0.7
Med- Surg ICU	4/5/2023 21:00	12	11.0	10.3	-0.7
Med- Surg ICU	4/5/2023 22:00	12	11.0	10.3	-0.7
Med- Surg ICU	4/5/2023 23:00	12	11.0	10.3	-0.7
Med- Surg ICU	4/6/2023 0:00	12	11.0	11.5	0.5
Med- Surg ICU	4/6/2023 1:00	12	11.0	11.5	0.5
Med- Surg ICU	4/6/2023 2:00	12	11.0	11.5	0.5
Med- Surg ICU	4/6/2023 3:00	12	11.0	11.5	0.5
Med- Surg ICU	4/6/2023 4:00	12	11.0	11.5	0.5
Med- Surg ICU	4/6/2023 5:00	12	11.0	11.5	0.5
Med- Surg ICU	4/6/2023 6:00	12	11.0	11.5	0.5
Med- Surg ICU	4/6/2023 7:00	12	11.0	12.2	1.2

Med- Surg ICU	4/6/2023 8:00	11	12.0	6.2	-5.8
Med- Surg ICU	4/6/2023 9:00	11	12.0	7.5	-4.5
Med- Surg ICU	4/6/2023 10:00	11	12.0	7.5	-4.5
Med- Surg ICU	4/6/2023 11:00	11	12.0	7.5	-4.5
Med- Surg ICU	4/6/2023 12:00	11	12.0	7.5	-4.5
Med- Surg ICU	4/6/2023 13:00	11	12.0	7.5	-4.5
Med- Surg ICU	4/6/2023 14:00	11	12.0	7.5	-4.5
Med- Surg ICU	4/6/2023 15:00	11	12.0	6.9	-5.2
Med- Surg ICU	4/6/2023 16:00	11	12.0	5.1	-6.9
Med- Surg ICU	4/6/2023 17:00	11	12.0	5.1	-6.9
Med- Surg ICU	4/6/2023 18:00	11	12.0	5.1	-6.9
Med- Surg ICU	4/6/2023 19:00	11	12.0	5.1	-6.9
Med- Surg ICU	4/6/2023 20:00	11	13.0	10.3	-2.7
Med- Surg ICU	4/6/2023 21:00	11	13.0	10.3	-2.7
Med- Surg ICU	4/6/2023 22:00	11	13.0	10.3	-2.7
Med- Surg ICU	4/6/2023 23:00	11	13.0	10.3	-2.7
Med- Surg ICU	4/7/2023 0:00	11	13.0	10.3	-2.7
Med- Surg ICU	4/7/2023 1:00	11	13.0	10.3	-2.7
Med- Surg ICU	4/7/2023 2:00	11	13.0	10.3	-2.7
Med- Surg ICU	4/7/2023 3:00	11	13.0	10.3	-2.7
Med- Surg ICU	4/7/2023 4:00	11	13.0	10.3	-2.7
Med- Surg ICU	4/7/2023 5:00	11	13.0	10.3	-2.7
Med- Surg ICU	4/7/2023 6:00	11	13.0	10.3	-2.7
Med- Surg ICU	4/7/2023 7:00	11	13.0	10.9	-2.1
Med- Surg ICU	4/7/2023 8:00	10	13.0	10.6	-2.4
Med- Surg ICU	4/7/2023 9:00	10	13.0	11.2	-1.8
Med- Surg ICU	4/7/2023 10:00	10	13.0	11.2	-1.8
Med- Surg ICU	4/7/2023 11:00	10	13.0	11.2	-1.8
Med- Surg ICU	4/7/2023 12:00	10	13.0	11.2	-1.8
Med- Surg ICU	4/7/2023 13:00	10	13.0	11.2	-1.8
Med- Surg ICU	4/7/2023 14:00	10	13.0	11.2	-1.8
Med- Surg ICU	4/7/2023 15:00	10	13.0	10.6	-2.4
Med- Surg ICU	4/7/2023 16:00	10	13.0	10.3	-2.7
Med- Surg ICU	4/7/2023 17:00	10	13.0	10.3	-2.7
Med- Surg ICU	4/7/2023 18:00	10	13.0	10.3	-2.7
Med- Surg ICU	4/7/2023 19:00	10	13.0	10.3	-2.7
Med- Surg ICU	4/7/2023 20:00	9	14.0	10.3	-3.7
Med- Surg ICU	4/7/2023 21:00	9	14.0	10.3	-3.7
Med- Surg ICU	4/7/2023 22:00	9	14.0	10.3	-3.7
Med- Surg ICU	4/7/2023 23:00	9	14.0	10.3	-3.7
Med- Surg ICU	4/8/2023 0:00	9	14.0	10.3	-3.7
Med- Surg ICU	4/8/2023 1:00	9	14.0	10.3	-3.7
Med- Surg ICU	4/8/2023 2:00	9	14.0	10.3	-3.7
Med- Surg ICU	4/8/2023 3:00	9	14.0	10.3	-3.7
Med- Surg ICU	4/8/2023 4:00	9	14.0	10.3	-3.7
Med- Surg ICU	4/8/2023 5:00	9	14.0	10.3	-3.7
Med- Surg ICU	4/8/2023 6:00	9	14.0	10.3	-3.7

Med- Surg ICU	4/8/2023 7:00	9	14.0	11.5	-2.5
Med- Surg ICU	4/8/2023 8:00	9	14.0	7.8	-6.2
Med- Surg ICU	4/8/2023 9:00	9	13.0	7.5	-5.5
Med- Surg ICU	4/8/2023 10:00	9	13.0	7.5	-5.5
Med- Surg ICU	4/8/2023 11:00	9	13.0	7.5	-5.5
Med- Surg ICU	4/8/2023 12:00	9	13.0	8.7	-4.3
Med- Surg ICU	4/8/2023 13:00	9	13.0	8.7	-4.3
Med- Surg ICU	4/8/2023 14:00	8	13.0	8.7	-4.3
Med- Surg ICU	4/8/2023 15:00	8	13.0	8.7	-4.3
Med- Surg ICU	4/8/2023 16:00	8	13.0	9.0	-4.0
Med- Surg ICU	4/8/2023 17:00	8	13.0	9.0	-4.0
Med- Surg ICU	4/8/2023 18:00	8	13.0	9.0	-4.0
Med- Surg ICU	4/8/2023 19:00	8	13.0	9.0	-4.0
Med- Surg ICU	4/8/2023 20:00	8	13.0	10.3	-2.7
Med- Surg ICU	4/8/2023 21:00	8	13.0	10.3	-2.7
Med- Surg ICU	4/8/2023 22:00	8	13.0	10.3	-2.7
Med- Surg ICU	4/8/2023 23:00	8	13.0	10.3	-2.7
Med- Surg ICU	4/9/2023 0:00	8	13.0	9.0	-4.0
Med- Surg ICU	4/9/2023 1:00	9	13.0	9.0	-4.0
Med- Surg ICU	4/9/2023 2:00	9	13.0	9.0	-4.0
Med- Surg ICU	4/9/2023 3:00	9	13.0	9.0	-4.0
Med- Surg ICU	4/9/2023 4:00	9	13.0	9.0	-4.0
Med- Surg ICU	4/9/2023 5:00	9	13.0	9.0	-4.0
Med- Surg ICU	4/9/2023 6:00	9	13.0	9.0	-4.0
Med- Surg ICU	4/9/2023 7:00	9	13.0	9.0	-4.0
Med- Surg ICU	4/9/2023 8:00	9	13.0	10.0	-3.0
Med- Surg ICU	4/9/2023 9:00	9	13.0	10.0	-3.0
Med- Surg ICU	4/9/2023 10:00	9	13.0	10.0	-3.0
Med- Surg ICU	4/9/2023 11:00	9	13.0	10.0	-3.0
Med- Surg ICU	4/9/2023 12:00	9	13.0	10.0	-3.0
Med- Surg ICU	4/9/2023 13:00	9	13.0	10.0	-3.0
Med- Surg ICU	4/9/2023 14:00	9	13.0	10.0	-3.0
Med- Surg ICU	4/9/2023 15:00	9	13.0	10.0	-3.0
Med- Surg ICU	4/9/2023 16:00	9	13.0	10.6	-2.4
Med- Surg ICU	4/9/2023 17:00	9	13.0	10.6	-2.4
Med- Surg ICU	4/9/2023 18:00	9	13.0	10.6	-2.4
Med- Surg ICU	4/9/2023 19:00	9	13.0	10.6	-2.4
Med- Surg ICU	4/9/2023 20:00	9	13.0	10.3	-2.7
Med- Surg ICU	4/9/2023 21:00	9	13.0	10.3	-2.7
Med- Surg ICU	4/9/2023 22:00	9	13.0	10.3	-2.7
Med- Surg ICU	4/9/2023 23:00	9	13.0	10.3	-2.7
Med- Surg ICU	4/10/2023 0:00	9	13.0	9.0	-4.0
Med- Surg ICU	4/10/2023 1:00	9	13.0	9.0	-4.0
Med- Surg ICU	4/10/2023 2:00	9	13.0	9.0	-4.0
Med- Surg ICU	4/10/2023 3:00	9	13.0	9.0	-4.0
Med- Surg ICU	4/10/2023 4:00	8	13.0	7.7	-5.3
Med- Surg ICU	4/10/2023 5:00	8	13.0	7.7	-5.3

Med- Surg ICU	4/10/2023 6:00	8	13.0	7.7	-5.3
Med- Surg ICU	4/10/2023 7:00	8	13.0	9.0	-4.0
Med- Surg ICU	4/10/2023 8:00	8	13.0	6.2	-6.8
Med- Surg ICU	4/10/2023 9:00	8	13.0	6.2	-6.8
Med- Surg ICU	4/10/2023 10:00	8	13.0	6.2	-6.8
Med- Surg ICU	4/10/2023 11:00	8	13.0	6.2	-6.8
Med- Surg ICU	4/10/2023 12:00	8	13.0	6.2	-6.8
Med- Surg ICU	4/10/2023 13:00	8	13.0	6.2	-6.8
Med- Surg ICU	4/10/2023 14:00	8	13.0	6.2	-6.8
Med- Surg ICU	4/10/2023 15:00	8	13.0	6.2	-6.8
Med- Surg ICU	4/10/2023 16:00	9	13.0	7.7	-5.3
Med- Surg ICU	4/10/2023 17:00	9	13.0	7.7	-5.3
Med- Surg ICU	4/10/2023 18:00	9	13.0	7.7	-5.3
Med- Surg ICU	4/10/2023 19:00	9	13.0	7.1	-6.0
Med- Surg ICU	4/10/2023 20:00	9	12.0	9.0	-3.0
Med- Surg ICU	4/10/2023 21:00	9	12.0	9.0	-3.0
Med- Surg ICU	4/10/2023 22:00	9	12.0	9.0	-3.0
Med- Surg ICU	4/10/2023 23:00	9	12.0	9.0	-3.0
Med- Surg ICU	4/11/2023 0:00	9	12.0	9.0	-3.0
Med- Surg ICU	4/11/2023 1:00	9	12.0	9.0	-3.0
Med- Surg ICU	4/11/2023 2:00	9	12.0	9.0	-3.0
Med- Surg ICU	4/11/2023 3:00	9	12.0	9.0	-3.0
Med- Surg ICU	4/11/2023 4:00	9	12.0	9.0	-3.0
Med- Surg ICU	4/11/2023 5:00	9	12.0	9.0	-3.0
Med- Surg ICU	4/11/2023 6:00	9	12.0	9.0	-3.0
Med- Surg ICU	4/11/2023 7:00	9	12.0	9.6	-2.4
Med- Surg ICU	4/11/2023 8:00	9	12.0	13.7	1.7
Med- Surg ICU	4/11/2023 9:00	9	12.0	13.7	1.7
Med- Surg ICU	4/11/2023 10:00	9	11.0	12.5	1.5
Med- Surg ICU	4/11/2023 11:00	9	11.0	12.5	1.5
Med- Surg ICU	4/11/2023 12:00	9	11.0	12.5	1.5
Med- Surg ICU	4/11/2023 13:00	9	11.0	12.5	1.5
Med- Surg ICU	4/11/2023 14:00	9	11.0	12.5	1.5
Med- Surg ICU	4/11/2023 15:00	9	11.0	11.8	0.8
Med- Surg ICU	4/11/2023 16:00	9	11.0	10.6	-0.4
Med- Surg ICU	4/11/2023 17:00	9	11.0	10.6	-0.4
Med- Surg ICU	4/11/2023 18:00	11	12.0	7.6	-4.5
Med- Surg ICU	4/11/2023 19:00	11	12.0	7.6	-4.5
Med- Surg ICU	4/11/2023 20:00	11	14.0	8.0	-6.0
Med- Surg ICU	4/11/2023 21:00	11	14.0	9.0	-5.0
Med- Surg ICU	4/11/2023 22:00	11	14.0	9.0	-5.0
Med- Surg ICU	4/11/2023 23:00	11	14.0	9.0	-5.0
Med- Surg ICU	4/12/2023 0:00	11	14.0	10.3	-3.7
Med- Surg ICU	4/12/2023 1:00	11	14.0	10.3	-3.7
Med- Surg ICU	4/12/2023 2:00	11	14.0	10.3	-3.7
Med- Surg ICU	4/12/2023 3:00	11	14.0	10.3	-3.7
Med- Surg ICU	4/12/2023 4:00	11	14.0	10.3	-3.7

Med- Surg ICU	4/12/2023 5:00	11	12.0	10.3	-1.7
Med- Surg ICU	4/12/2023 6:00	11	12.0	10.3	-1.7
Med- Surg ICU	4/12/2023 7:00	11	12.0	10.9	-1.1
Med- Surg ICU	4/12/2023 8:00	11	12.0	10.0	-2.0
Med- Surg ICU	4/12/2023 9:00	11	12.0	10.0	-2.0
Med- Surg ICU	4/12/2023 10:00	11	12.0	10.0	-2.0
Med- Surg ICU	4/12/2023 11:00	11	12.0	10.0	-2.0
Med- Surg ICU	4/12/2023 12:00	11	12.0	10.0	-2.0
Med- Surg ICU	4/12/2023 13:00	11	12.0	10.0	-2.0
Med- Surg ICU	4/12/2023 14:00	11	12.0	10.0	-2.0
Med- Surg ICU	4/12/2023 15:00	11	12.0	10.0	-2.0
Med- Surg ICU	4/12/2023 16:00	11	12.0	9.0	-3.0
Med- Surg ICU	4/12/2023 17:00	11	12.0	9.0	-3.0
Med- Surg ICU	4/12/2023 18:00	11	12.0	9.0	-3.0
Med- Surg ICU	4/12/2023 19:00	11	12.0	8.3	-3.7
Med- Surg ICU	4/12/2023 20:00	11	12.0	10.3	-1.7
Med- Surg ICU	4/12/2023 21:00	11	12.0	10.3	-1.7
Med- Surg ICU	4/12/2023 22:00	11	12.0	10.3	-1.7
Med- Surg ICU	4/12/2023 23:00	11	12.0	9.3	-2.7
Med- Surg ICU	4/13/2023 0:00	12	13.0	11.5	-1.5
Med- Surg ICU	4/13/2023 1:00	12	13.0	11.5	-1.5
Med- Surg ICU	4/13/2023 2:00	12	13.0	10.3	-2.7
Med- Surg ICU	4/13/2023 3:00	12	13.0	10.3	-2.7
Med- Surg ICU	4/13/2023 4:00	12	13.0	10.3	-2.7
Med- Surg ICU	4/13/2023 5:00	12	13.0	10.3	-2.7
Med- Surg ICU	4/13/2023 6:00	12	13.0	10.3	-2.7
Med- Surg ICU	4/13/2023 7:00	12	13.0	10.9	-2.1
Med- Surg ICU	4/13/2023 8:00	12	13.0	10.0	-3.0
Med- Surg ICU	4/13/2023 9:00	12	13.0	10.0	-3.0
Med- Surg ICU	4/13/2023 10:00	12	13.0	10.0	-3.0
Med- Surg ICU	4/13/2023 11:00	12	13.0	10.0	-3.0
Med- Surg ICU	4/13/2023 12:00	12	13.0	10.0	-3.0
Med- Surg ICU	4/13/2023 13:00	12	13.0	10.0	-3.0
Med- Surg ICU	4/13/2023 14:00	12	13.0	10.0	-3.0
Med- Surg ICU	4/13/2023 15:00	12	13.0	10.0	-3.0
Med- Surg ICU	4/13/2023 16:00	12	13.0	9.0	-4.0
Med- Surg ICU	4/13/2023 17:00	12	13.0	9.0	-4.0
Med- Surg ICU	4/13/2023 18:00	12	13.0	9.0	-4.0
Med- Surg ICU	4/13/2023 19:00	12	13.0	8.3	-4.7
Med- Surg ICU	4/13/2023 20:00	11	14.0	10.3	-3.7
Med- Surg ICU	4/13/2023 21:00	11	14.0	10.3	-3.7
Med- Surg ICU	4/13/2023 22:00	11	14.0	10.3	-3.7
Med- Surg ICU	4/13/2023 23:00	11	14.0	10.3	-3.7
Med- Surg ICU	4/14/2023 0:00	11	14.0	9.0	-5.0
Med- Surg ICU	4/14/2023 1:00	11	14.0	9.0	-5.0
Med- Surg ICU	4/14/2023 2:00	11	14.0	9.0	-5.0
Med- Surg ICU	4/14/2023 3:00	11	14.0	9.0	-5.0

Med- Surg ICU	4/14/2023 4:00	11	14.0	9.0	-5.0
Med- Surg ICU	4/14/2023 5:00	11	14.0	9.0	-5.0
Med- Surg ICU	4/14/2023 6:00	11	14.0	9.0	-5.0
Med- Surg ICU	4/14/2023 7:00	11	14.0	9.6	-4.4
Med- Surg ICU	4/14/2023 8:00	11	14.0	11.2	-2.8
Med- Surg ICU	4/14/2023 9:00	11	14.0	11.2	-2.8
Med- Surg ICU	4/14/2023 10:00	11	14.0	11.2	-2.8
Med- Surg ICU	4/14/2023 11:00	11	14.0	11.2	-2.8
Med- Surg ICU	4/14/2023 12:00	10	14.0	10.3	-3.7
Med- Surg ICU	4/14/2023 13:00	11	14.0	10.3	-3.7
Med- Surg ICU	4/14/2023 14:00	12	14.0	11.2	-2.8
Med- Surg ICU	4/14/2023 15:00	12	14.0	10.6	-3.4
Med- Surg ICU	4/14/2023 16:00	12	14.0	10.3	-3.7
Med- Surg ICU	4/14/2023 17:00	12	14.0	10.3	-3.7
Med- Surg ICU	4/14/2023 18:00	12	14.0	10.3	-3.7
Med- Surg ICU	4/14/2023 19:00	12	14.0	10.3	-3.7
Med- Surg ICU	4/14/2023 20:00	12	13.0	10.3	-2.7
Med- Surg ICU	4/14/2023 21:00	12	13.0	10.3	-2.7
Med- Surg ICU	4/14/2023 22:00	12	13.0	10.3	-2.7
Med- Surg ICU	4/14/2023 23:00	12	13.0	10.3	-2.7
Med- Surg ICU	4/15/2023 0:00	12	13.0	10.3	-2.7
Med- Surg ICU	4/15/2023 1:00	12	13.0	10.3	-2.7
Med- Surg ICU	4/15/2023 2:00	12	13.0	10.3	-2.7
Med- Surg ICU	4/15/2023 3:00	12	13.0	10.3	-2.7
Med- Surg ICU	4/15/2023 4:00	12	13.0	9.0	-4.0
Med- Surg ICU	4/15/2023 5:00	12	13.0	9.0	-4.0
Med- Surg ICU	4/15/2023 6:00	12	13.0	9.0	-4.0
Med- Surg ICU	4/15/2023 7:00	12	13.0	9.0	-4.0
Med- Surg ICU	4/15/2023 8:00	12	13.0	10.0	-3.0
Med- Surg ICU	4/15/2023 9:00	12	13.0	10.0	-3.0
Med- Surg ICU	4/15/2023 10:00	12	13.0	11.2	-1.8
Med- Surg ICU	4/15/2023 11:00	12	13.0	11.2	-1.8
Med- Surg ICU	4/15/2023 12:00	12	13.0	12.5	-0.6
Med- Surg ICU	4/15/2023 13:00	12	13.0	12.5	-0.6
Med- Surg ICU	4/15/2023 14:00	12	13.0	12.5	-0.6
Med- Surg ICU	4/15/2023 15:00	12	13.0	12.5	-0.6
Med- Surg ICU	4/15/2023 16:00	12	13.0	12.8	-0.2
Med- Surg ICU	4/15/2023 17:00	12	13.0	12.8	-0.2
Med- Surg ICU	4/15/2023 18:00	12	13.0	12.8	-0.2
Med- Surg ICU	4/15/2023 19:00	10	13.0	11.5	-1.5
Med- Surg ICU	4/15/2023 20:00	10	13.0	10.3	-2.7
Med- Surg ICU	4/15/2023 21:00	10	13.0	10.3	-2.7
Med- Surg ICU	4/15/2023 22:00	10	13.0	10.3	-2.7
Med- Surg ICU	4/15/2023 23:00	10	13.0	10.3	-2.7
Med- Surg ICU	4/16/2023 0:00	10	13.0	9.0	-4.0
Med- Surg ICU	4/16/2023 1:00	10	13.0	9.0	-4.0
Med- Surg ICU	4/16/2023 2:00	10	13.0	9.0	-4.0

Med- Surg ICU	4/16/2023 3:00	10	13.0	9.0	-4.0
Med- Surg ICU	4/16/2023 4:00	10	13.0	9.0	-4.0
Med- Surg ICU	4/16/2023 5:00	10	13.0	9.0	-4.0
Med- Surg ICU	4/16/2023 6:00	10	13.0	9.0	-4.0
Med- Surg ICU	4/16/2023 7:00	10	13.0	9.0	-4.0
Med- Surg ICU	4/16/2023 8:00	10	13.0	8.7	-4.3
Med- Surg ICU	4/16/2023 9:00	10	13.0	8.7	-4.3
Med- Surg ICU	4/16/2023 10:00	10	13.0	10.0	-3.0
Med- Surg ICU	4/16/2023 11:00	10	13.0	10.0	-3.0
Med- Surg ICU	4/16/2023 12:00	10	13.0	10.0	-3.0
Med- Surg ICU	4/16/2023 13:00	10	13.0	10.0	-3.0
Med- Surg ICU	4/16/2023 14:00	10	13.0	10.0	-3.0
Med- Surg ICU	4/16/2023 15:00	10	13.0	10.0	-3.0
Med- Surg ICU	4/16/2023 16:00	10	13.0	9.6	-3.4
Med- Surg ICU	4/16/2023 17:00	10	13.0	10.3	-2.7
Med- Surg ICU	4/16/2023 18:00	10	13.0	10.3	-2.7
Med- Surg ICU	4/16/2023 19:00	9	13.0	7.7	-5.3
Med- Surg ICU	4/16/2023 20:00	9	13.0	7.7	-5.3
Med- Surg ICU	4/16/2023 21:00	9	13.0	7.7	-5.3
Med- Surg ICU	4/16/2023 22:00	9	13.0	7.7	-5.3
Med- Surg ICU	4/16/2023 23:00	9	13.0	7.7	-5.3
Med- Surg ICU	4/17/2023 0:00	9	13.0	7.7	-5.3
Med- Surg ICU	4/17/2023 1:00	8	13.0	9.0	-4.0
Med- Surg ICU	4/17/2023 2:00	8	13.0	9.0	-4.0
Med- Surg ICU	4/17/2023 3:00	8	13.0	9.0	-4.0
Med- Surg ICU	4/17/2023 4:00	8	13.0	9.0	-4.0
Med- Surg ICU	4/17/2023 5:00	9	13.0	9.0	-4.0
Med- Surg ICU	4/17/2023 6:00	9	13.0	9.0	-4.0
Med- Surg ICU	4/17/2023 7:00	9	13.0	9.0	-4.0
Med- Surg ICU	4/17/2023 8:00	9	12.0	7.5	-4.5
Med- Surg ICU	4/17/2023 9:00	9	12.0	7.5	-4.5
Med- Surg ICU	4/17/2023 10:00	9	12.0	7.5	-4.5
Med- Surg ICU	4/17/2023 11:00	9	12.0	8.1	-3.9
Med- Surg ICU	4/17/2023 12:00	9	12.0	9.3	-2.7
Med- Surg ICU	4/17/2023 13:00	9	11.0	10.0	-1.0
Med- Surg ICU	4/17/2023 14:00	9	11.0	10.0	-1.0
Med- Surg ICU	4/17/2023 15:00	9	11.0	10.0	-1.0
Med- Surg ICU	4/17/2023 16:00	9	11.0	9.0	-2.0
Med- Surg ICU	4/17/2023 17:00	9	11.0	9.0	-2.0
Med- Surg ICU	4/17/2023 18:00	9	11.0	9.0	-2.0
Med- Surg ICU	4/17/2023 19:00	9	11.0	8.3	-2.7
Med- Surg ICU	4/17/2023 20:00	9	11.0	9.0	-2.0
Med- Surg ICU	4/17/2023 21:00	9	11.0	9.0	-2.0
Med- Surg ICU	4/17/2023 22:00	9	11.0	9.0	-2.0
Med- Surg ICU	4/17/2023 23:00	9	11.0	8.3	-2.7
Med- Surg ICU	4/18/2023 0:00	9	11.0	6.7	-4.3
Med- Surg ICU	4/18/2023 1:00	9	11.0	7.7	-3.3

Med- Surg ICU	4/18/2023 2:00	9	11.0	7.7	-3.3
Med- Surg ICU	4/18/2023 3:00	9	11.0	7.7	-3.3
Med- Surg ICU	4/18/2023 4:00	9	11.0	7.7	-3.3
Med- Surg ICU	4/18/2023 5:00	9	11.0	7.7	-3.3
Med- Surg ICU	4/18/2023 6:00	9	12.0	7.7	-4.3
Med- Surg ICU	4/18/2023 7:00	9	12.0	7.7	-4.3
Med- Surg ICU	4/18/2023 8:00	9	13.0	10.0	-3.0
Med- Surg ICU	4/18/2023 9:00	9	13.0	10.0	-3.0
Med- Surg ICU	4/18/2023 10:00	9	13.0	10.0	-3.0
Med- Surg ICU	4/18/2023 11:00	9	13.0	10.0	-3.0
Med- Surg ICU	4/18/2023 12:00	8	13.0	10.0	-3.0
Med- Surg ICU	4/18/2023 13:00	8	13.0	10.0	-3.0
Med- Surg ICU	4/18/2023 14:00	8	13.0	10.0	-3.0
Med- Surg ICU	4/18/2023 15:00	8	13.0	10.0	-3.0
Med- Surg ICU	4/18/2023 16:00	8	13.0	9.0	-4.0
Med- Surg ICU	4/18/2023 17:00	8	13.0	9.0	-4.0
Med- Surg ICU	4/18/2023 18:00	8	13.0	9.0	-4.0
Med- Surg ICU	4/18/2023 19:00	8	13.0	9.0	-4.0
Med- Surg ICU	4/18/2023 20:00	8	13.0	10.3	-2.7
Med- Surg ICU	4/18/2023 21:00	8	13.0	10.3	-2.7
Med- Surg ICU	4/18/2023 22:00	8	13.0	10.3	-2.7
Med- Surg ICU	4/18/2023 23:00	8	13.0	10.3	-2.7
Med- Surg ICU	4/19/2023 0:00	9	13.0	10.3	-2.7
Med- Surg ICU	4/19/2023 1:00	9	13.0	10.3	-2.7
Med- Surg ICU	4/19/2023 2:00	9	13.0	10.3	-2.7
Med- Surg ICU	4/19/2023 3:00	9	13.0	10.3	-2.7
Med- Surg ICU	4/19/2023 4:00	9	13.0	10.3	-2.7
Med- Surg ICU	4/19/2023 5:00	9	13.0	10.3	-2.7
Med- Surg ICU	4/19/2023 6:00	9	13.0	10.3	-2.7
Med- Surg ICU	4/19/2023 7:00	9	13.0	10.3	-2.7
Med- Surg ICU	4/19/2023 8:00	9	13.0	12.5	-0.6
Med- Surg ICU	4/19/2023 9:00	9	13.0	12.5	-0.6
Med- Surg ICU	4/19/2023 10:00	9	13.0	12.5	-0.6
Med- Surg ICU	4/19/2023 11:00	9	13.0	12.5	-0.6
Med- Surg ICU	4/19/2023 12:00	9	13.0	10.3	-2.7
Med- Surg ICU	4/19/2023 13:00	9	13.0	10.0	-3.0
Med- Surg ICU	4/19/2023 14:00	10	12.0	10.0	-2.0
Med- Surg ICU	4/19/2023 15:00	9	12.0	10.0	-2.0
Med- Surg ICU	4/19/2023 16:00	9	12.0	9.1	-3.0
Med- Surg ICU	4/19/2023 17:00	9	12.0	9.1	-3.0
Med- Surg ICU	4/19/2023 18:00	9	12.0	9.1	-3.0
Med- Surg ICU	4/19/2023 19:00	9	12.0	9.1	-3.0
Med- Surg ICU	4/19/2023 20:00	10	13.0	6.6	-6.4
Med- Surg ICU	4/19/2023 21:00	10	13.0	7.6	-5.5
Med- Surg ICU	4/19/2023 22:00	9	13.0	9.0	-4.0
Med- Surg ICU	4/19/2023 23:00	9	13.0	9.0	-4.0
Med- Surg ICU	4/20/2023 0:00	9	13.0	7.7	-5.3

Med- Surg ICU	4/20/2023 1:00	9	13.0	7.7	-5.3
Med- Surg ICU	4/20/2023 2:00	9	13.0	7.7	-5.3
Med- Surg ICU	4/20/2023 3:00	9	13.0	7.7	-5.3
Med- Surg ICU	4/20/2023 4:00	9	13.0	7.7	-5.3
Med- Surg ICU	4/20/2023 5:00	9	13.0	7.7	-5.3
Med- Surg ICU	4/20/2023 6:00	9	13.0	7.7	-5.3
Med- Surg ICU	4/20/2023 7:00	9	13.0	9.0	-4.0
Med- Surg ICU	4/20/2023 8:00	9	13.0	8.7	-4.3
Med- Surg ICU	4/20/2023 9:00	9	13.0	8.7	-4.3
Med- Surg ICU	4/20/2023 10:00	9	13.0	8.7	-4.3
Med- Surg ICU	4/20/2023 11:00	9	13.0	8.7	-4.3
Med- Surg ICU	4/20/2023 12:00	9	13.0	10.0	-3.0
Med- Surg ICU	4/20/2023 13:00	9	13.0	10.0	-3.0
Med- Surg ICU	4/20/2023 14:00	9	13.0	10.0	-3.0
Med- Surg ICU	4/20/2023 15:00	9	13.0	10.0	-3.0
Med- Surg ICU	4/20/2023 16:00	9	13.0	10.3	-2.7
Med- Surg ICU	4/20/2023 17:00	9	13.0	10.3	-2.7
Med- Surg ICU	4/20/2023 18:00	9	13.0	10.3	-2.7
Med- Surg ICU	4/20/2023 19:00	9	13.0	10.3	-2.7
Med- Surg ICU	4/20/2023 20:00	9	13.0	9.0	-4.0
Med- Surg ICU	4/20/2023 21:00	9	13.0	9.0	-4.0
Med- Surg ICU	4/20/2023 22:00	9	13.0	8.3	-4.7
Med- Surg ICU	4/20/2023 23:00	9	13.0	7.7	-5.3
Med- Surg ICU	4/21/2023 0:00	9	13.0	9.0	-4.0
Med- Surg ICU	4/21/2023 1:00	9	13.0	9.0	-4.0
Med- Surg ICU	4/21/2023 2:00	9	13.0	9.0	-4.0
Med- Surg ICU	4/21/2023 3:00	9	13.0	9.0	-4.0
Med- Surg ICU	4/21/2023 4:00	9	13.0	9.0	-4.0
Med- Surg ICU	4/21/2023 5:00	9	13.0	9.0	-4.0
Med- Surg ICU	4/21/2023 6:00	9	13.0	9.0	-4.0
Med- Surg ICU	4/21/2023 7:00	9	13.0	9.0	-4.0
Med- Surg ICU	4/21/2023 8:00	9	13.0	10.0	-3.0
Med- Surg ICU	4/21/2023 9:00	9	13.0	10.0	-3.0
Med- Surg ICU	4/21/2023 10:00	9	13.0	10.0	-3.0
Med- Surg ICU	4/21/2023 11:00	9	13.0	10.0	-3.0
Med- Surg ICU	4/21/2023 12:00	8	13.0	11.2	-1.8
Med- Surg ICU	4/21/2023 13:00	8	13.0	11.2	-1.8
Med- Surg ICU	4/21/2023 14:00	8	13.0	11.2	-1.8
Med- Surg ICU	4/21/2023 15:00	8	13.0	11.2	-1.8
Med- Surg ICU	4/21/2023 16:00	8	13.0	11.5	-1.5
Med- Surg ICU	4/21/2023 17:00	8	13.0	11.5	-1.5
Med- Surg ICU	4/21/2023 18:00	8	13.0	11.5	-1.5
Med- Surg ICU	4/21/2023 19:00	8	13.0	10.9	-2.1
Med- Surg ICU	4/21/2023 20:00	9	13.0	6.4	-6.6
Med- Surg ICU	4/21/2023 21:00	9	13.0	6.4	-6.6
Med- Surg ICU	4/21/2023 22:00	9	13.0	6.4	-6.6
Med- Surg ICU	4/21/2023 23:00	9	13.0	6.4	-6.6

Med- Surg ICU	4/22/2023 0:00	9	13.0	6.4	-6.6
Med- Surg ICU	4/22/2023 1:00	9	13.0	6.4	-6.6
Med- Surg ICU	4/22/2023 2:00	9	13.0	6.4	-6.6
Med- Surg ICU	4/22/2023 3:00	9	13.0	6.4	-6.6
Med- Surg ICU	4/22/2023 4:00	9	13.0	6.4	-6.6
Med- Surg ICU	4/22/2023 5:00	9	13.0	6.4	-6.6
Med- Surg ICU	4/22/2023 6:00	9	10.0	6.4	-3.6
Med- Surg ICU	4/22/2023 7:00	9	10.0	6.4	-3.6
Med- Surg ICU	4/22/2023 8:00	9	10.0	3.7	-6.3
Med- Surg ICU	4/22/2023 9:00	9	10.0	3.7	-6.3
Med- Surg ICU	4/22/2023 10:00	9	10.0	3.7	-6.3
Med- Surg ICU	4/22/2023 11:00	9	10.0	3.7	-6.3
Med- Surg ICU	4/22/2023 12:00	9	10.0	5.0	-5.0
Med- Surg ICU	4/22/2023 13:00	9	10.0	5.0	-5.0
Med- Surg ICU	4/22/2023 14:00	9	10.0	5.0	-5.0
Med- Surg ICU	4/22/2023 15:00	9	10.0	5.0	-5.0
Med- Surg ICU	4/22/2023 16:00	9	10.0	5.1	-4.9
Med- Surg ICU	4/22/2023 17:00	9	10.0	5.1	-4.9
Med- Surg ICU	4/22/2023 18:00	9	10.0	5.1	-4.9
Med- Surg ICU	4/22/2023 19:00	9	10.0	5.1	-4.9
Med- Surg ICU	4/22/2023 20:00	9	12.0	7.7	-4.3
Med- Surg ICU	4/22/2023 21:00	9	12.0	7.7	-4.3
Med- Surg ICU	4/22/2023 22:00	9	12.0	7.7	-4.3
Med- Surg ICU	4/22/2023 23:00	9	12.0	7.7	-4.3
Med- Surg ICU	4/23/2023 0:00	9	12.0	6.4	-5.6
Med- Surg ICU	4/23/2023 1:00	9	12.0	6.4	-5.6
Med- Surg ICU	4/23/2023 2:00	9	12.0	6.4	-5.6
Med- Surg ICU	4/23/2023 3:00	9	12.0	6.4	-5.6
Med- Surg ICU	4/23/2023 4:00	9	12.0	6.4	-5.6
Med- Surg ICU	4/23/2023 5:00	9	11.0	6.4	-4.6
Med- Surg ICU	4/23/2023 6:00	9	11.0	6.4	-4.6
Med- Surg ICU	4/23/2023 7:00	9	11.0	6.4	-4.6
Med- Surg ICU	4/23/2023 8:00	9	10.0	6.2	-3.8
Med- Surg ICU	4/23/2023 9:00	9	10.0	6.2	-3.8
Med- Surg ICU	4/23/2023 10:00	9	10.0	6.2	-3.8
Med- Surg ICU	4/23/2023 11:00	9	10.0	6.2	-3.8
Med- Surg ICU	4/23/2023 12:00	9	10.0	6.2	-3.8
Med- Surg ICU	4/23/2023 13:00	9	10.0	6.2	-3.8
Med- Surg ICU	4/23/2023 14:00	9	10.0	5.6	-4.4
Med- Surg ICU	4/23/2023 15:00	8	10.0	5.0	-5.0
Med- Surg ICU	4/23/2023 16:00	8	10.0	6.4	-3.6
Med- Surg ICU	4/23/2023 17:00	8	10.0	6.4	-3.6
Med- Surg ICU	4/23/2023 18:00	8	10.0	6.4	-3.6
Med- Surg ICU	4/23/2023 19:00	8	10.0	6.4	-3.6
Med- Surg ICU	4/23/2023 20:00	8	10.0	6.4	-3.6
Med- Surg ICU	4/23/2023 21:00	8	10.0	6.4	-3.6
Med- Surg ICU	4/23/2023 22:00	8	10.0	6.4	-3.6

Med- Surg ICU	4/23/2023 23:00	8	10.0	6.4	-3.6
Med- Surg ICU	4/24/2023 0:00	8	10.0	5.1	-4.9
Med- Surg ICU	4/24/2023 1:00	8	10.0	5.1	-4.9
Med- Surg ICU	4/24/2023 2:00	8	10.0	5.1	-4.9
Med- Surg ICU	4/24/2023 3:00	8	10.0	5.1	-4.9
Med- Surg ICU	4/24/2023 4:00	8	10.0	5.1	-4.9
Med- Surg ICU	4/24/2023 5:00	8	10.0	5.1	-4.9
Med- Surg ICU	4/24/2023 6:00	8	10.0	5.1	-4.9
Med- Surg ICU	4/24/2023 7:00	8	10.0	5.1	-4.9
Med- Surg ICU	4/24/2023 8:00	8	10.0	6.2	-3.8
Med- Surg ICU	4/24/2023 9:00	8	10.0	6.2	-3.8
Med- Surg ICU	4/24/2023 10:00	8	10.0	6.2	-3.8
Med- Surg ICU	4/24/2023 11:00	8	10.0	6.2	-3.8
Med- Surg ICU	4/24/2023 12:00	8	10.0	6.2	-3.8
Med- Surg ICU	4/24/2023 13:00	8	10.0	6.2	-3.8
Med- Surg ICU	4/24/2023 14:00	8	10.0	6.2	-3.8
Med- Surg ICU	4/24/2023 15:00	8	10.0	6.2	-3.8
Med- Surg ICU	4/24/2023 16:00	8	10.0	5.1	-4.9
Med- Surg ICU	4/24/2023 17:00	8	10.0	5.1	-4.9
Med- Surg ICU	4/24/2023 18:00	8	10.0	5.1	-4.9
Med- Surg ICU	4/24/2023 19:00	8	10.0	5.1	-4.9
Med- Surg ICU	4/24/2023 20:00	8	10.0	5.1	-4.9
Med- Surg ICU	4/24/2023 21:00	8	10.0	5.1	-4.9
Med- Surg ICU	4/24/2023 22:00	8	10.0	5.1	-4.9
Med- Surg ICU	4/24/2023 23:00	8	10.0	5.1	-4.9
Med- Surg ICU	4/25/2023 0:00	8	10.0	5.1	-4.9
Med- Surg ICU	4/25/2023 1:00	8	10.0	5.1	-4.9
Med- Surg ICU	4/25/2023 2:00	8	10.0	5.1	-4.9
Med- Surg ICU	4/25/2023 3:00	8	10.0	5.1	-4.9
Med- Surg ICU	4/25/2023 4:00	8	10.0	5.1	-4.9
Med- Surg ICU	4/25/2023 5:00	8	10.0	5.1	-4.9
Med- Surg ICU	4/25/2023 6:00	8	10.0	5.1	-4.9
Med- Surg ICU	4/25/2023 7:00	8	10.0	5.1	-4.9
Med- Surg ICU	4/25/2023 8:00	8	12.0	6.2	-5.8
Med- Surg ICU	4/25/2023 9:00	8	12.0	6.2	-5.8
Med- Surg ICU	4/25/2023 10:00	8	12.0	6.2	-5.8
Med- Surg ICU	4/25/2023 11:00	8	12.0	6.2	-5.8
Med- Surg ICU	4/25/2023 12:00	8	12.0	6.2	-5.8
Med- Surg ICU	4/25/2023 13:00	8	12.0	6.2	-5.8
Med- Surg ICU	4/25/2023 14:00	8	12.0	6.2	-5.8
Med- Surg ICU	4/25/2023 15:00	8	12.0	6.2	-5.8
Med- Surg ICU	4/25/2023 16:00	8	12.0	5.1	-6.9
Med- Surg ICU	4/25/2023 17:00	8	12.0	5.1	-6.9
Med- Surg ICU	4/25/2023 18:00	11	12.0	5.1	-6.9
Med- Surg ICU	4/25/2023 19:00	11	12.0	5.1	-6.9
Med- Surg ICU	4/25/2023 20:00	11	12.0	6.4	-5.6
Med- Surg ICU	4/25/2023 21:00	11	12.0	6.4	-5.6

Med- Surg ICU	4/25/2023 22:00	11	12.0	7.1	-5.0
Med- Surg ICU	4/25/2023 23:00	11	12.0	7.7	-4.3
Med- Surg ICU	4/26/2023 0:00	11	12.0	7.7	-4.3
Med- Surg ICU	4/26/2023 1:00	11	12.0	7.7	-4.3
Med- Surg ICU	4/26/2023 2:00	11	12.0	7.7	-4.3
Med- Surg ICU	4/26/2023 3:00	11	12.0	7.7	-4.3
Med- Surg ICU	4/26/2023 4:00	11	12.0	7.7	-4.3
Med- Surg ICU	4/26/2023 5:00	11	12.0	7.7	-4.3
Med- Surg ICU	4/26/2023 6:00	11	12.0	7.7	-4.3
Med- Surg ICU	4/26/2023 7:00	11	12.0	7.7	-4.3
Med- Surg ICU	4/26/2023 8:00	10	12.0	7.5	-4.5
Med- Surg ICU	4/26/2023 9:00	10	12.0	7.5	-4.5
Med- Surg ICU	4/26/2023 10:00	10	12.0	7.5	-4.5
Med- Surg ICU	4/26/2023 11:00	10	12.0	7.5	-4.5
Med- Surg ICU	4/26/2023 12:00	11	12.0	7.5	-4.5
Med- Surg ICU	4/26/2023 13:00	11	12.0	7.5	-4.5
Med- Surg ICU	4/26/2023 14:00	11	12.0	7.5	-4.5
Med- Surg ICU	4/26/2023 15:00	11	12.0	7.5	-4.5
Med- Surg ICU	4/26/2023 16:00	11	12.0	9.0	-3.0
Med- Surg ICU	4/26/2023 17:00	11	12.0	9.0	-3.0
Med- Surg ICU	4/26/2023 18:00	12	12.0	9.0	-3.0
Med- Surg ICU	4/26/2023 19:00	12	12.0	9.0	-3.0
Med- Surg ICU	4/26/2023 20:00	12	12.0	7.7	-4.3
Med- Surg ICU	4/26/2023 21:00	12	12.0	7.7	-4.3
Med- Surg ICU	4/26/2023 22:00	12	12.0	7.7	-4.3
Med- Surg ICU	4/26/2023 23:00	12	12.0	7.7	-4.3
Med- Surg ICU	4/27/2023 0:00	12	12.0	7.7	-4.3
Med- Surg ICU	4/27/2023 1:00	12	12.0	7.7	-4.3
Med- Surg ICU	4/27/2023 2:00	12	12.0	7.7	-4.3
Med- Surg ICU	4/27/2023 3:00	12	12.0	7.7	-4.3
Med- Surg ICU	4/27/2023 4:00	12	12.0	6.4	-5.6
Med- Surg ICU	4/27/2023 5:00	12	12.0	6.4	-5.6
Med- Surg ICU	4/27/2023 6:00	12	12.0	6.4	-5.6
Med- Surg ICU	4/27/2023 7:00	12	12.0	6.4	-5.6
Med- Surg ICU	4/27/2023 8:00	12	12.0	6.2	-5.8
Med- Surg ICU	4/27/2023 9:00	12	12.0	6.2	-5.8
Med- Surg ICU	4/27/2023 10:00	12	12.0	6.2	-5.8
Med- Surg ICU	4/27/2023 11:00	12	12.0	4.4	-7.6
Med- Surg ICU	4/27/2023 12:00	12	12.0	5.3	-6.7
Med- Surg ICU	4/27/2023 13:00	12	12.0	6.2	-5.8
Med- Surg ICU	4/27/2023 14:00	12	12.0	6.2	-5.8
Med- Surg ICU	4/27/2023 15:00	12	12.0	6.2	-5.8
Med- Surg ICU	4/27/2023 16:00	12	12.0	6.4	-5.6
Med- Surg ICU	4/27/2023 17:00	12	12.0	6.4	-5.6
Med- Surg ICU	4/27/2023 18:00	12	12.0	5.5	-6.6
Med- Surg ICU	4/27/2023 19:00	12	12.0	6.4	-5.6
Med- Surg ICU	4/27/2023 20:00	12	14.0	10.3	-3.7

Med- Surg ICU	4/27/2023 21:00	12	14.0	10.3	-3.7
Med- Surg ICU	4/27/2023 22:00	12	14.0	10.3	-3.7
Med- Surg ICU	4/27/2023 23:00	12	14.0	10.3	-3.7
Med- Surg ICU	4/28/2023 0:00	12	14.0	10.3	-3.7
Med- Surg ICU	4/28/2023 1:00	12	14.0	10.3	-3.7
Med- Surg ICU	4/28/2023 2:00	12	14.0	10.3	-3.7
Med- Surg ICU	4/28/2023 3:00	12	14.0	10.3	-3.7
Med- Surg ICU	4/28/2023 4:00	12	14.0	9.0	-5.0
Med- Surg ICU	4/28/2023 5:00	12	14.0	9.0	-5.0
Med- Surg ICU	4/28/2023 6:00	12	14.0	9.0	-5.0
Med- Surg ICU	4/28/2023 7:00	12	14.0	9.0	-5.0
Med- Surg ICU	4/28/2023 8:00	12	13.0	5.0	-8.0
Med- Surg ICU	4/28/2023 9:00	12	13.0	5.0	-8.0
Med- Surg ICU	4/28/2023 10:00	12	13.0	5.0	-8.0
Med- Surg ICU	4/28/2023 11:00	12	13.0	5.0	-8.0
Med- Surg ICU	4/28/2023 12:00	11	13.0	5.0	-8.0
Med- Surg ICU	4/28/2023 13:00	11	13.0	5.0	-8.0
Med- Surg ICU	4/28/2023 14:00	11	13.0	5.0	-8.0
Med- Surg ICU	4/28/2023 15:00	11	13.0	4.4	-8.6
Med- Surg ICU	4/28/2023 16:00	12	13.0	5.1	-7.9
Med- Surg ICU	4/28/2023 17:00	12	13.0	5.1	-7.9
Med- Surg ICU	4/28/2023 18:00	12	13.0	5.1	-7.9
Med- Surg ICU	4/28/2023 19:00	11	13.0	5.1	-7.9
Med- Surg ICU	4/28/2023 20:00	11	14.0	11.5	-2.5
Med- Surg ICU	4/28/2023 21:00	11	14.0	11.5	-2.5
Med- Surg ICU	4/28/2023 22:00	11	14.0	11.5	-2.5
Med- Surg ICU	4/28/2023 23:00	11	14.0	11.5	-2.5
Med- Surg ICU	4/29/2023 0:00	11	14.0	11.5	-2.5
Med- Surg ICU	4/29/2023 1:00	11	14.0	11.5	-2.5
Med- Surg ICU	4/29/2023 2:00	11	14.0	11.5	-2.5
Med- Surg ICU	4/29/2023 3:00	11	14.0	11.5	-2.5
Med- Surg ICU	4/29/2023 4:00	11	14.0	11.5	-2.5
Med- Surg ICU	4/29/2023 5:00	11	14.0	11.5	-2.5
Med- Surg ICU	4/29/2023 6:00	11	14.0	11.5	-2.5
Med- Surg ICU	4/29/2023 7:00	11	14.0	11.5	-2.5
Med- Surg ICU	4/29/2023 8:00	11	14.0	11.2	-2.8
Med- Surg ICU	4/29/2023 9:00	11	14.0	11.2	-2.8
Med- Surg ICU	4/29/2023 10:00	11	14.0	11.2	-2.8
Med- Surg ICU	4/29/2023 11:00	11	14.0	11.2	-2.8
Med- Surg ICU	4/29/2023 12:00	11	14.0	11.2	-2.8
Med- Surg ICU	4/29/2023 13:00	11	14.0	11.2	-2.8
Med- Surg ICU	4/29/2023 14:00	11	14.0	11.2	-2.8
Med- Surg ICU	4/29/2023 15:00	11	14.0	11.2	-2.8
Med- Surg ICU	4/29/2023 16:00	11	14.0	11.5	-2.5
Med- Surg ICU	4/29/2023 17:00	11	14.0	11.5	-2.5
Med- Surg ICU	4/29/2023 18:00	11	14.0	11.5	-2.5
Med- Surg ICU	4/29/2023 19:00	11	14.0	11.5	-2.5

Med- Surg ICU	4/29/2023 20:00	10	14.0	10.3	-3.7
Med- Surg ICU	4/29/2023 21:00	10	14.0	10.3	-3.7
Med- Surg ICU	4/29/2023 22:00	10	14.0	10.3	-3.7
Med- Surg ICU	4/29/2023 23:00	10	14.0	10.3	-3.7
Med- Surg ICU	4/30/2023 0:00	10	14.0	11.5	-2.5
Med- Surg ICU	4/30/2023 1:00	9	14.0	11.5	-2.5
Med- Surg ICU	4/30/2023 2:00	9	14.0	11.5	-2.5
Med- Surg ICU	4/30/2023 3:00	9	14.0	11.5	-2.5
Med- Surg ICU	4/30/2023 4:00	9	14.0	11.5	-2.5
Med- Surg ICU	4/30/2023 5:00	9	14.0	11.5	-2.5
Med- Surg ICU	4/30/2023 6:00	9	14.0	11.5	-2.5
Med- Surg ICU	4/30/2023 7:00	9	14.0	11.5	-2.5
Med- Surg ICU	4/30/2023 8:00	9	14.0	10.0	-4.0
Med- Surg ICU	4/30/2023 9:00	9	10.0	8.7	-1.3
Med- Surg ICU	4/30/2023 10:00	9	10.0	8.7	-1.3
Med- Surg ICU	4/30/2023 11:00	9	10.0	8.7	-1.3
Med- Surg ICU	4/30/2023 12:00	9	10.0	8.7	-1.3
Med- Surg ICU	4/30/2023 13:00	9	10.0	8.7	-1.3
Med- Surg ICU	4/30/2023 14:00	9	10.0	8.7	-1.3
Med- Surg ICU	4/30/2023 15:00	9	10.0	8.7	-1.3
Med- Surg ICU	4/30/2023 16:00	9	10.0	7.6	-2.5
Med- Surg ICU	4/30/2023 17:00	9	10.0	7.6	-2.5
Med- Surg ICU	4/30/2023 18:00	9	10.0	7.6	-2.5
Med- Surg ICU	4/30/2023 19:00	9	15.0	7.6	-7.5
Med- Surg ICU	4/30/2023 20:00	9	15.0	9.0	-6.0
Med- Surg ICU	4/30/2023 21:00	9	15.0	9.0	-6.0
Med- Surg ICU	4/30/2023 22:00	9	15.0	9.0	-6.0
Med- Surg ICU	4/30/2023 23:00	9	15.0	9.0	-6.0
Med- Surg ICU	5/1/2023 0:00	9	15.0	9.6	-5.4
Med- Surg ICU	5/1/2023 1:00	9	15.0	10.3	-4.7
Med- Surg ICU	5/1/2023 2:00	9	15.0	10.3	-4.7
Med- Surg ICU	5/1/2023 3:00	9	15.0	10.3	-4.7
Med- Surg ICU	5/1/2023 4:00	9	15.0	10.3	-4.7
Med- Surg ICU	5/1/2023 5:00	9	15.0	10.3	-4.7
Med- Surg ICU	5/1/2023 6:00	9	15.0	10.3	-4.7
Med- Surg ICU	5/1/2023 7:00	9	15.0	10.3	-4.7
Med- Surg ICU	5/1/2023 8:00	9	15.0	7.5	-7.5
Med- Surg ICU	5/1/2023 9:00	9	11.0	7.5	-3.5
Med- Surg ICU	5/1/2023 10:00	9	11.0	7.5	-3.5
Med- Surg ICU	5/1/2023 11:00	9	11.0	7.5	-3.5
Med- Surg ICU	5/1/2023 12:00	9	11.0	7.5	-3.5
Med- Surg ICU	5/1/2023 13:00	9	11.0	7.5	-3.5
Med- Surg ICU	5/1/2023 14:00	9	11.0	7.5	-3.5
Med- Surg ICU	5/1/2023 15:00	9	11.0	7.5	-3.5
Med- Surg ICU	5/1/2023 16:00	9	11.0	10.6	-0.4
Med- Surg ICU	5/1/2023 17:00	9	11.0	10.6	-0.4
Med- Surg ICU	5/1/2023 18:00	9	11.0	9.1	-2.0

Med- Surg ICU	5/1/2023 19:00	9	11.0	9.1	-2.0
Med- Surg ICU	5/1/2023 20:00	10	14.0	10.6	-3.4
Med- Surg ICU	5/1/2023 21:00	10	14.0	10.6	-3.4
Med- Surg ICU	5/1/2023 22:00	10	14.0	10.6	-3.4
Med- Surg ICU	5/1/2023 23:00	10	14.0	10.6	-3.4
Med- Surg ICU	5/2/2023 0:00	10	14.0	9.0	-5.0
Med- Surg ICU	5/2/2023 1:00	10	14.0	9.0	-5.0
Med- Surg ICU	5/2/2023 2:00	10	14.0	9.0	-5.0
Med- Surg ICU	5/2/2023 3:00	10	14.0	9.0	-5.0
Med- Surg ICU	5/2/2023 4:00	10	14.0	7.7	-6.3
Med- Surg ICU	5/2/2023 5:00	10	14.0	7.7	-6.3
Med- Surg ICU	5/2/2023 6:00	10	14.0	7.7	-6.3
Med- Surg ICU	5/2/2023 7:00	10	14.0	7.7	-6.3
Med- Surg ICU	5/2/2023 8:00	11	12.0	11.2	-0.8
Med- Surg ICU	5/2/2023 9:00	11	12.0	10.0	-2.0
Med- Surg ICU	5/2/2023 10:00	11	12.0	10.0	-2.0
Med- Surg ICU	5/2/2023 11:00	11	12.0	9.3	-2.7
Med- Surg ICU	5/2/2023 12:00	11	12.0	8.7	-3.3
Med- Surg ICU	5/2/2023 13:00	11	12.0	8.7	-3.3
Med- Surg ICU	5/2/2023 14:00	11	12.0	8.7	-3.3
Med- Surg ICU	5/2/2023 15:00	11	12.0	8.7	-3.3
Med- Surg ICU	5/2/2023 16:00	11	12.0	6.0	-6.0
Med- Surg ICU	5/2/2023 17:00	11	12.0	6.0	-6.0
Med- Surg ICU	5/2/2023 18:00	11	12.0	4.5	-7.5
Med- Surg ICU	5/2/2023 19:00	11	12.0	4.5	-7.5
Med- Surg ICU	5/2/2023 20:00	11	12.0	9.0	-3.0
Med- Surg ICU	5/2/2023 21:00	11	12.0	9.0	-3.0
Med- Surg ICU	5/2/2023 22:00	11	12.0	9.0	-3.0
Med- Surg ICU	5/2/2023 23:00	11	12.0	9.0	-3.0
Med- Surg ICU	5/3/2023 0:00	11	12.0	9.0	-3.0
Med- Surg ICU	5/3/2023 1:00	11	12.0	9.0	-3.0
Med- Surg ICU	5/3/2023 2:00	11	12.0	9.0	-3.0
Med- Surg ICU	5/3/2023 3:00	11	12.0	9.0	-3.0
Med- Surg ICU	5/3/2023 4:00	11	12.0	9.0	-3.0
Med- Surg ICU	5/3/2023 5:00	11	12.0	9.0	-3.0
Med- Surg ICU	5/3/2023 6:00	11	12.0	9.0	-3.0
Med- Surg ICU	5/3/2023 7:00	11	12.0	9.0	-3.0
Med- Surg ICU	5/3/2023 8:00	10	13.0	11.2	-1.8
Med- Surg ICU	5/3/2023 9:00	10	13.0	11.2	-1.8
Med- Surg ICU	5/3/2023 10:00	10	13.0	11.2	-1.8
Med- Surg ICU	5/3/2023 11:00	10	13.0	11.2	-1.8
Med- Surg ICU	5/3/2023 12:00	10	13.0	12.5	-0.6
Med- Surg ICU	5/3/2023 13:00	10	13.0	12.5	-0.6
Med- Surg ICU	5/3/2023 14:00	11	14.0	10.0	-4.0
Med- Surg ICU	5/3/2023 15:00	11	14.0	10.0	-4.0
Med- Surg ICU	5/3/2023 16:00	14	15.0	7.6	-7.5
Med- Surg ICU	5/3/2023 17:00	14	15.0	7.6	-7.5

Med- Surg ICU	5/3/2023 18:00	14	15.0	6.0	-9.0
Med- Surg ICU	5/3/2023 19:00	14	15.0	6.0	-9.0
Med- Surg ICU	5/3/2023 20:00	13	15.0	9.0	-6.0
Med- Surg ICU	5/3/2023 21:00	13	15.0	9.0	-6.0
Med- Surg ICU	5/3/2023 22:00	13	15.0	9.0	-6.0
Med- Surg ICU	5/3/2023 23:00	13	15.0	9.0	-6.0
Med- Surg ICU	5/4/2023 0:00	13	15.0	9.0	-6.0
Med- Surg ICU	5/4/2023 1:00	13	15.0	9.0	-6.0
Med- Surg ICU	5/4/2023 2:00	13	15.0	9.0	-6.0
Med- Surg ICU	5/4/2023 3:00	14	15.0	10.3	-4.7
Med- Surg ICU	5/4/2023 4:00	14	15.0	10.3	-4.7
Med- Surg ICU	5/4/2023 5:00	14	15.0	10.3	-4.7
Med- Surg ICU	5/4/2023 6:00	14	15.0	10.3	-4.7
Med- Surg ICU	5/4/2023 7:00	14	15.0	10.3	-4.7
Med- Surg ICU	5/4/2023 8:00	14	15.0	7.3	-7.7
Med- Surg ICU	5/4/2023 9:00	14	15.0	6.2	-8.8
Med- Surg ICU	5/4/2023 10:00	14	15.0	6.2	-8.8
Med- Surg ICU	5/4/2023 11:00	14	15.0	6.2	-8.8
Med- Surg ICU	5/4/2023 12:00	14	15.0	6.2	-8.8
Med- Surg ICU	5/4/2023 13:00	14	15.0	6.2	-8.8
Med- Surg ICU	5/4/2023 14:00	14	15.0	6.2	-8.8
Med- Surg ICU	5/4/2023 15:00	14	15.0	6.2	-8.8
Med- Surg ICU	5/4/2023 16:00	14	15.0	5.1	-9.9
Med- Surg ICU	5/4/2023 17:00	14	15.0	5.1	-9.9
Med- Surg ICU	5/4/2023 18:00	14	15.0	3.9	-11.2
Med- Surg ICU	5/4/2023 19:00	14	15.0	3.9	-11.2
Med- Surg ICU	5/4/2023 20:00	12	14.0	3.9	-10.2
Med- Surg ICU	5/4/2023 21:00	12	14.0	3.9	-10.2
Med- Surg ICU	5/4/2023 22:00	12	14.0	3.9	-10.2
Med- Surg ICU	5/4/2023 23:00	14	14.0	5.1	-8.9
Med- Surg ICU	5/5/2023 0:00	14	14.0	6.0	-8.0
Med- Surg ICU	5/5/2023 1:00	14	14.0	6.0	-8.0
Med- Surg ICU	5/5/2023 2:00	14	14.0	6.0	-8.0
Med- Surg ICU	5/5/2023 3:00	14	14.0	6.0	-8.0
Med- Surg ICU	5/5/2023 4:00	14	14.0	6.0	-8.0
Med- Surg ICU	5/5/2023 5:00	14	14.0	6.0	-8.0
Med- Surg ICU	5/5/2023 6:00	14	14.0	6.0	-8.0
Med- Surg ICU	5/5/2023 7:00	14	14.0	6.0	-8.0
Med- Surg ICU	5/5/2023 8:00	12	13.0	8.7	-4.3
Med- Surg ICU	5/5/2023 9:00	12	13.0	8.7	-4.3
Med- Surg ICU	5/5/2023 10:00	12	13.0	8.7	-4.3
Med- Surg ICU	5/5/2023 11:00	12	13.0	8.7	-4.3
Med- Surg ICU	5/5/2023 12:00	12	13.0	8.7	-4.3
Med- Surg ICU	5/5/2023 13:00	12	13.0	8.7	-4.3
Med- Surg ICU	5/5/2023 14:00	12	13.0	8.7	-4.3
Med- Surg ICU	5/5/2023 15:00	12	13.0	8.1	-4.9
Med- Surg ICU	5/5/2023 16:00	12	13.0	6.0	-7.0

Med- Surg ICU	5/5/2023 17:00	11	13.0	6.0	-7.0
Med- Surg ICU	5/5/2023 18:00	11	13.0	6.0	-7.0
Med- Surg ICU	5/5/2023 19:00	11	13.0	6.0	-7.0
Med- Surg ICU	5/5/2023 20:00	11	13.0	7.6	-5.5
Med- Surg ICU	5/5/2023 21:00	11	13.0	7.6	-5.5
Med- Surg ICU	5/5/2023 22:00	11	13.0	7.6	-5.5
Med- Surg ICU	5/5/2023 23:00	11	13.0	10.3	-2.7
Med- Surg ICU	5/6/2023 0:00	11	13.0	9.0	-4.0
Med- Surg ICU	5/6/2023 1:00	11	13.0	9.0	-4.0
Med- Surg ICU	5/6/2023 2:00	11	13.0	9.0	-4.0
Med- Surg ICU	5/6/2023 3:00	11	13.0	9.0	-4.0
Med- Surg ICU	5/6/2023 4:00	11	13.0	9.0	-4.0
Med- Surg ICU	5/6/2023 5:00	11	13.0	9.0	-4.0
Med- Surg ICU	5/6/2023 6:00	11	13.0	9.0	-4.0
Med- Surg ICU	5/6/2023 7:00	11	13.0	9.0	-4.0
Med- Surg ICU	5/6/2023 8:00	11	13.0	7.5	-5.5
Med- Surg ICU	5/6/2023 9:00	11	13.0	7.5	-5.5
Med- Surg ICU	5/6/2023 10:00	11	13.0	7.5	-5.5
Med- Surg ICU	5/6/2023 11:00	11	13.0	7.5	-5.5
Med- Surg ICU	5/6/2023 12:00	11	13.0	8.7	-4.3
Med- Surg ICU	5/6/2023 13:00	11	13.0	8.7	-4.3
Med- Surg ICU	5/6/2023 14:00	11	13.0	8.7	-4.3
Med- Surg ICU	5/6/2023 15:00	11	13.0	8.7	-4.3
Med- Surg ICU	5/6/2023 16:00	11	13.0	10.3	-2.7
Med- Surg ICU	5/6/2023 17:00	11	13.0	10.3	-2.7
Med- Surg ICU	5/6/2023 18:00	11	13.0	10.3	-2.7
Med- Surg ICU	5/6/2023 19:00	11	13.0	12.1	-0.9
Med- Surg ICU	5/6/2023 20:00	11	13.0	9.0	-4.0
Med- Surg ICU	5/6/2023 21:00	11	13.0	9.0	-4.0
Med- Surg ICU	5/6/2023 22:00	11	13.0	9.0	-4.0
Med- Surg ICU	5/6/2023 23:00	11	13.0	9.0	-4.0
Med- Surg ICU	5/7/2023 0:00	11	13.0	7.7	-5.3
Med- Surg ICU	5/7/2023 1:00	11	13.0	7.7	-5.3
Med- Surg ICU	5/7/2023 2:00	11	13.0	7.7	-5.3
Med- Surg ICU	5/7/2023 3:00	11	13.0	7.7	-5.3
Med- Surg ICU	5/7/2023 4:00	10	13.0	7.7	-5.3
Med- Surg ICU	5/7/2023 5:00	10	13.0	7.7	-5.3
Med- Surg ICU	5/7/2023 6:00	10	13.0	7.7	-5.3
Med- Surg ICU	5/7/2023 7:00	10	13.0	7.7	-5.3
Med- Surg ICU	5/7/2023 8:00	10	13.0	10.0	-3.0
Med- Surg ICU	5/7/2023 9:00	10	11.0	8.7	-2.3
Med- Surg ICU	5/7/2023 10:00	10	11.0	8.7	-2.3
Med- Surg ICU	5/7/2023 11:00	10	11.0	8.7	-2.3
Med- Surg ICU	5/7/2023 12:00	10	11.0	8.7	-2.3
Med- Surg ICU	5/7/2023 13:00	11	11.0	8.7	-2.3
Med- Surg ICU	5/7/2023 14:00	11	11.0	8.7	-2.3
Med- Surg ICU	5/7/2023 15:00	11	11.0	8.7	-2.3

Med- Surg ICU	5/7/2023 16:00	11	11.0	10.3	-0.7
Med- Surg ICU	5/7/2023 17:00	11	11.0	10.3	-0.7
Med- Surg ICU	5/7/2023 18:00	9	11.0	10.3	-0.7
Med- Surg ICU	5/7/2023 19:00	9	11.0	10.3	-0.7
Med- Surg ICU	5/7/2023 20:00	9	11.0	9.0	-2.0
Med- Surg ICU	5/7/2023 21:00	9	11.0	9.0	-2.0
Med- Surg ICU	5/7/2023 22:00	9	11.0	9.0	-2.0
Med- Surg ICU	5/7/2023 23:00	9	11.0	9.0	-2.0
Med- Surg ICU	5/8/2023 0:00	9	11.0	6.7	-4.3
Med- Surg ICU	5/8/2023 1:00	9	11.0	7.7	-3.3
Med- Surg ICU	5/8/2023 2:00	9	11.0	7.7	-3.3
Med- Surg ICU	5/8/2023 3:00	9	11.0	7.7	-3.3
Med- Surg ICU	5/8/2023 4:00	9	11.0	7.7	-3.3
Med- Surg ICU	5/8/2023 5:00	9	11.0	7.7	-3.3
Med- Surg ICU	5/8/2023 6:00	9	11.0	7.7	-3.3
Med- Surg ICU	5/8/2023 7:00	9	11.0	9.0	-2.0
Med- Surg ICU	5/8/2023 8:00	9	11.0	8.7	-2.3
Med- Surg ICU	5/8/2023 9:00	9	11.0	8.7	-2.3
Med- Surg ICU	5/8/2023 10:00	9	11.0	8.7	-2.3
Med- Surg ICU	5/8/2023 11:00	9	11.0	8.7	-2.3
Med- Surg ICU	5/8/2023 12:00	9	11.0	8.7	-2.3
Med- Surg ICU	5/8/2023 13:00	9	11.0	8.7	-2.3
Med- Surg ICU	5/8/2023 14:00	9	11.0	8.7	-2.3
Med- Surg ICU	5/8/2023 15:00	9	9.0	8.7	-0.3
Med- Surg ICU	5/8/2023 16:00	10	11.0	7.6	-3.5
Med- Surg ICU	5/8/2023 17:00	10	11.0	7.6	-3.5
Med- Surg ICU	5/8/2023 18:00	10	11.0	7.6	-3.5
Med- Surg ICU	5/8/2023 19:00	10	11.0	7.6	-3.5
Med- Surg ICU	5/8/2023 20:00	10	12.0	9.0	-3.0
Med- Surg ICU	5/8/2023 21:00	10	12.0	8.0	-4.0
Med- Surg ICU	5/8/2023 22:00	10	12.0	9.0	-3.0
Med- Surg ICU	5/8/2023 23:00	10	12.0	9.0	-3.0
Med- Surg ICU	5/9/2023 0:00	10	12.0	9.0	-3.0
Med- Surg ICU	5/9/2023 1:00	10	12.0	9.0	-3.0
Med- Surg ICU	5/9/2023 2:00	10	12.0	9.0	-3.0
Med- Surg ICU	5/9/2023 3:00	10	12.0	9.0	-3.0
Med- Surg ICU	5/9/2023 4:00	12	12.0	9.0	-3.0
Med- Surg ICU	5/9/2023 5:00	12	12.0	9.0	-3.0
Med- Surg ICU	5/9/2023 6:00	12	12.0	9.0	-3.0
Med- Surg ICU	5/9/2023 7:00	10	12.0	9.0	-3.0
Med- Surg ICU	5/9/2023 8:00	11	12.0	10.0	-2.0
Med- Surg ICU	5/9/2023 9:00	11	12.0	10.0	-2.0
Med- Surg ICU	5/9/2023 10:00	11	12.0	10.0	-2.0
Med- Surg ICU	5/9/2023 11:00	11	12.0	9.0	-3.0
Med- Surg ICU	5/9/2023 12:00	11	12.0	10.0	-2.0
Med- Surg ICU	5/9/2023 13:00	11	12.0	9.0	-3.0
Med- Surg ICU	5/9/2023 14:00	11	12.0	10.0	-2.0

Med- Surg ICU	5/9/2023 15:00	11	12.0	10.0	-2.0
Med- Surg ICU	5/9/2023 16:00	11	12.0	9.0	-3.0
Med- Surg ICU	5/9/2023 17:00	11	12.0	9.0	-3.0
Med- Surg ICU	5/9/2023 18:00	11	12.0	9.0	-3.0
Med- Surg ICU	5/9/2023 19:00	10	14.0	7.6	-6.5
Med- Surg ICU	5/9/2023 20:00	10	13.0	9.0	-4.0
Med- Surg ICU	5/9/2023 21:00	10	13.0	9.0	-4.0
Med- Surg ICU	5/9/2023 22:00	10	13.0	9.0	-4.0
Med- Surg ICU	5/9/2023 23:00	10	13.0	9.0	-4.0
Med- Surg ICU	5/10/2023 0:00	10	13.0	10.9	-2.1
Med- Surg ICU	5/10/2023 1:00	10	13.0	11.5	-1.5
Med- Surg ICU	5/10/2023 2:00	10	13.0	11.5	-1.5
Med- Surg ICU	5/10/2023 3:00	10	13.0	11.5	-1.5
Med- Surg ICU	5/10/2023 4:00	10	13.0	11.5	-1.5
Med- Surg ICU	5/10/2023 5:00	10	13.0	11.5	-1.5
Med- Surg ICU	5/10/2023 6:00	10	13.0	11.5	-1.5
Med- Surg ICU	5/10/2023 7:00	10	13.0	11.5	-1.5
Med- Surg ICU	5/10/2023 8:00	10	13.0	8.7	-4.3
Med- Surg ICU	5/10/2023 9:00	10	13.0	8.7	-4.3
Med- Surg ICU	5/10/2023 10:00	10	13.0	8.7	-4.3
Med- Surg ICU	5/10/2023 11:00	10	13.0	8.7	-4.3
Med- Surg ICU	5/10/2023 12:00	10	13.0	10.0	-3.0
Med- Surg ICU	5/10/2023 13:00	10	13.0	10.0	-3.0
Med- Surg ICU	5/10/2023 14:00	10	13.0	10.0	-3.0
Med- Surg ICU	5/10/2023 15:00	10	13.0	10.0	-3.0
Med- Surg ICU	5/10/2023 16:00	10	13.0	9.1	-4.0
Med- Surg ICU	5/10/2023 17:00	10	13.0	9.1	-4.0
Med- Surg ICU	5/10/2023 18:00	10	13.0	9.1	-4.0
Med- Surg ICU	5/10/2023 19:00	10	13.0	9.8	-3.2
Med- Surg ICU	5/10/2023 20:00	10	13.0	11.5	-1.5
Med- Surg ICU	5/10/2023 21:00	10	13.0	11.5	-1.5
Med- Surg ICU	5/10/2023 22:00	10	13.0	11.5	-1.5
Med- Surg ICU	5/10/2023 23:00	10	13.0	11.5	-1.5
Med- Surg ICU	5/11/2023 0:00	10	13.0	11.5	-1.5
Med- Surg ICU	5/11/2023 1:00	10	13.0	11.5	-1.5
Med- Surg ICU	5/11/2023 2:00	10	13.0	11.5	-1.5
Med- Surg ICU	5/11/2023 3:00	10	13.0	11.5	-1.5
Med- Surg ICU	5/11/2023 4:00	10	13.0	11.5	-1.5
Med- Surg ICU	5/11/2023 5:00	10	13.0	11.5	-1.5
Med- Surg ICU	5/11/2023 6:00	10	13.0	11.5	-1.5
Med- Surg ICU	5/11/2023 7:00	10	13.0	11.5	-1.5
Med- Surg ICU	5/11/2023 8:00	13	14.0	11.2	-2.8
Med- Surg ICU	5/11/2023 9:00	13	14.0	11.2	-2.8
Med- Surg ICU	5/11/2023 10:00	13	14.0	11.2	-2.8
Med- Surg ICU	5/11/2023 11:00	13	14.0	11.2	-2.8
Med- Surg ICU	5/11/2023 12:00	13	14.0	11.2	-2.8
Med- Surg ICU	5/11/2023 13:00	13	14.0	11.2	-2.8

Med- Surg ICU	5/11/2023 14:00	13	14.0	11.2	-2.8
Med- Surg ICU	5/11/2023 15:00	13	14.0	11.2	-2.8
Med- Surg ICU	5/11/2023 16:00	13	14.0	10.6	-3.4
Med- Surg ICU	5/11/2023 17:00	13	14.0	10.6	-3.4
Med- Surg ICU	5/11/2023 18:00	13	14.0	10.6	-3.4
Med- Surg ICU	5/11/2023 19:00	13	14.0	10.6	-3.4
Med- Surg ICU	5/11/2023 20:00	13	15.0	12.8	-2.2
Med- Surg ICU	5/11/2023 21:00	13	15.0	12.8	-2.2
Med- Surg ICU	5/11/2023 22:00	13	15.0	12.8	-2.2
Med- Surg ICU	5/11/2023 23:00	13	15.0	12.8	-2.2
Med- Surg ICU	5/12/2023 0:00	13	15.0	12.8	-2.2
Med- Surg ICU	5/12/2023 1:00	13	15.0	12.8	-2.2
Med- Surg ICU	5/12/2023 2:00	13	15.0	12.8	-2.2
Med- Surg ICU	5/12/2023 3:00	14	15.0	12.8	-2.2
Med- Surg ICU	5/12/2023 4:00	14	15.0	12.8	-2.2
Med- Surg ICU	5/12/2023 5:00	14	15.0	12.8	-2.2
Med- Surg ICU	5/12/2023 6:00	14	15.0	12.8	-2.2
Med- Surg ICU	5/12/2023 7:00	14	15.0	12.8	-2.2
Med- Surg ICU	5/12/2023 8:00	14	15.0	11.2	-3.8
Med- Surg ICU	5/12/2023 9:00	14	14.0	11.2	-2.8
Med- Surg ICU	5/12/2023 10:00	14	14.0	11.2	-2.8
Med- Surg ICU	5/12/2023 11:00	14	14.0	11.2	-2.8
Med- Surg ICU	5/12/2023 12:00	14	14.0	10.9	-3.1
Med- Surg ICU	5/12/2023 13:00	14	14.0	10.0	-4.0
Med- Surg ICU	5/12/2023 14:00	14	14.0	10.0	-4.0
Med- Surg ICU	5/12/2023 15:00	14	14.0	10.0	-4.0
Med- Surg ICU	5/12/2023 16:00	14	14.0	9.0	-5.0
Med- Surg ICU	5/12/2023 17:00	14	14.0	9.0	-5.0
Med- Surg ICU	5/12/2023 18:00	14	14.0	9.0	-5.0
Med- Surg ICU	5/12/2023 19:00	14	14.0	9.0	-5.0
Med- Surg ICU	5/12/2023 20:00	14	14.0	9.0	-5.0
Med- Surg ICU	5/12/2023 21:00	14	14.0	9.0	-5.0
Med- Surg ICU	5/12/2023 22:00	14	14.0	9.0	-5.0
Med- Surg ICU	5/12/2023 23:00	14	14.0	9.0	-5.0
Med- Surg ICU	5/13/2023 0:00	14	14.0	10.3	-3.7
Med- Surg ICU	5/13/2023 1:00	14	14.0	10.3	-3.7
Med- Surg ICU	5/13/2023 2:00	14	14.0	10.3	-3.7
Med- Surg ICU	5/13/2023 3:00	14	14.0	10.3	-3.7
Med- Surg ICU	5/13/2023 4:00	14	14.0	10.3	-3.7
Med- Surg ICU	5/13/2023 5:00	13	14.0	10.3	-3.7
Med- Surg ICU	5/13/2023 6:00	13	14.0	10.3	-3.7
Med- Surg ICU	5/13/2023 7:00	13	14.0	10.3	-3.7
Med- Surg ICU	5/13/2023 8:00	13	14.0	8.7	-5.3
Med- Surg ICU	5/13/2023 9:00	13	14.0	8.7	-5.3
Med- Surg ICU	5/13/2023 10:00	13	14.0	8.7	-5.3
Med- Surg ICU	5/13/2023 11:00	13	14.0	8.7	-5.3
Med- Surg ICU	5/13/2023 12:00	13	14.0	10.0	-4.0

Med- Surg ICU	5/13/2023 13:00	13	14.0	10.0	-4.0
Med- Surg ICU	5/13/2023 14:00	13	14.0	10.0	-4.0
Med- Surg ICU	5/13/2023 15:00	13	14.0	10.0	-4.0
Med- Surg ICU	5/13/2023 16:00	12	14.0	9.0	-5.0
Med- Surg ICU	5/13/2023 17:00	12	14.0	9.0	-5.0
Med- Surg ICU	5/13/2023 18:00	12	14.0	9.0	-5.0
Med- Surg ICU	5/13/2023 19:00	12	14.0	9.0	-5.0
Med- Surg ICU	5/13/2023 20:00	13	12.0	9.0	-3.0
Med- Surg ICU	5/13/2023 21:00	13	12.0	9.0	-3.0
Med- Surg ICU	5/13/2023 22:00	13	12.0	9.0	-3.0
Med- Surg ICU	5/13/2023 23:00	13	12.0	9.0	-3.0
Med- Surg ICU	5/14/2023 0:00	13	12.0	9.0	-3.0
Med- Surg ICU	5/14/2023 1:00	13	12.0	9.0	-3.0
Med- Surg ICU	5/14/2023 2:00	13	12.0	9.0	-3.0
Med- Surg ICU	5/14/2023 3:00	13	12.0	9.0	-3.0
Med- Surg ICU	5/14/2023 4:00	13	12.0	9.0	-3.0
Med- Surg ICU	5/14/2023 5:00	13	12.0	9.0	-3.0
Med- Surg ICU	5/14/2023 6:00	13	12.0	9.0	-3.0
Med- Surg ICU	5/14/2023 7:00	13	12.0	9.0	-3.0
Med- Surg ICU	5/14/2023 8:00	13	12.0	10.0	-2.0
Med- Surg ICU	5/14/2023 9:00	13	12.0	10.0	-2.0
Med- Surg ICU	5/14/2023 10:00	13	12.0	10.0	-2.0
Med- Surg ICU	5/14/2023 11:00	13	12.0	10.0	-2.0
Med- Surg ICU	5/14/2023 12:00	13	12.0	10.0	-2.0
Med- Surg ICU	5/14/2023 13:00	13	12.0	10.0	-2.0
Med- Surg ICU	5/14/2023 14:00	13	12.0	10.0	-2.0
Med- Surg ICU	5/14/2023 15:00	13	12.0	10.0	-2.0
Med- Surg ICU	5/14/2023 16:00	13	12.0	10.3	-1.7
Med- Surg ICU	5/14/2023 17:00	13	12.0	10.3	-1.7
Med- Surg ICU	5/14/2023 18:00	13	12.0	10.3	-1.7
Med- Surg ICU	5/14/2023 19:00	13	12.0	10.3	-1.7
Med- Surg ICU	5/14/2023 20:00	12	14.0	10.3	-3.7
Med- Surg ICU	5/14/2023 21:00	11	14.0	10.3	-3.7
Med- Surg ICU	5/14/2023 22:00	11	14.0	10.3	-3.7
Med- Surg ICU	5/14/2023 23:00	11	14.0	10.3	-3.7
Med- Surg ICU	5/15/2023 0:00	11	14.0	10.3	-3.7
Med- Surg ICU	5/15/2023 1:00	11	14.0	10.3	-3.7
Med- Surg ICU	5/15/2023 2:00	11	14.0	10.3	-3.7
Med- Surg ICU	5/15/2023 3:00	11	14.0	10.3	-3.7
Med- Surg ICU	5/15/2023 4:00	11	14.0	9.0	-5.0
Med- Surg ICU	5/15/2023 5:00	11	14.0	9.0	-5.0
Med- Surg ICU	5/15/2023 6:00	11	14.0	9.0	-5.0
Med- Surg ICU	5/15/2023 7:00	11	14.0	9.0	-5.0
Med- Surg ICU	5/15/2023 8:00	11	14.0	11.2	-2.8
Med- Surg ICU	5/15/2023 9:00	11	14.0	11.2	-2.8
Med- Surg ICU	5/15/2023 10:00	11	14.0	11.2	-2.8
Med- Surg ICU	5/15/2023 11:00	11	14.0	9.3	-4.7

Med- Surg ICU	5/15/2023 12:00	11	14.0	11.2	-2.8
Med- Surg ICU	5/15/2023 13:00	11	14.0	11.2	-2.8
Med- Surg ICU	5/15/2023 14:00	11	14.0	10.3	-3.7
Med- Surg ICU	5/15/2023 15:00	11	14.0	11.8	-2.2
Med- Surg ICU	5/15/2023 16:00	11	14.0	12.1	-1.9
Med- Surg ICU	5/15/2023 17:00	14	14.0	12.1	-1.9
Med- Surg ICU	5/15/2023 18:00	14	14.0	12.1	-1.9
Med- Surg ICU	5/15/2023 19:00	12	14.0	12.1	-1.9
Med- Surg ICU	5/15/2023 20:00	12	14.0	12.1	-1.9
Med- Surg ICU	5/15/2023 21:00	12	14.0	12.1	-1.9
Med- Surg ICU	5/15/2023 22:00	12	14.0	12.1	-1.9
Med- Surg ICU	5/15/2023 23:00	12	14.0	12.1	-1.9
Med- Surg ICU	5/16/2023 0:00	12	14.0	11.5	-2.5
Med- Surg ICU	5/16/2023 1:00	12	14.0	11.5	-2.5
Med- Surg ICU	5/16/2023 2:00	12	14.0	11.5	-2.5
Med- Surg ICU	5/16/2023 3:00	12	14.0	11.5	-2.5
Med- Surg ICU	5/16/2023 4:00	12	14.0	11.5	-2.5
Med- Surg ICU	5/16/2023 5:00	12	14.0	11.5	-2.5
Med- Surg ICU	5/16/2023 6:00	12	14.0	11.5	-2.5
Med- Surg ICU	5/16/2023 7:00	12	14.0	11.5	-2.5
Med- Surg ICU	5/16/2023 8:00	12	12.0	10.0	-2.0
Med- Surg ICU	5/16/2023 9:00	12	12.0	10.0	-2.0
Med- Surg ICU	5/16/2023 10:00	12	12.0	10.0	-2.0
Med- Surg ICU	5/16/2023 11:00	12	12.0	10.0	-2.0
Med- Surg ICU	5/16/2023 12:00	12	12.0	10.0	-2.0
Med- Surg ICU	5/16/2023 13:00	12	12.0	10.0	-2.0
Med- Surg ICU	5/16/2023 14:00	12	12.0	10.0	-2.0
Med- Surg ICU	5/16/2023 15:00	12	12.0	10.0	-2.0
Med- Surg ICU	5/16/2023 16:00	12	12.0	8.3	-3.7
Med- Surg ICU	5/16/2023 17:00	12	12.0	9.1	-3.0
Med- Surg ICU	5/16/2023 18:00	12	12.0	9.1	-3.0
Med- Surg ICU	5/16/2023 19:00	12	12.0	9.1	-3.0
Med- Surg ICU	5/16/2023 20:00	13	15.0	12.8	-2.2
Med- Surg ICU	5/16/2023 21:00	13	15.0	12.8	-2.2
Med- Surg ICU	5/16/2023 22:00	13	15.0	12.8	-2.2
Med- Surg ICU	5/16/2023 23:00	13	15.0	12.8	-2.2
Med- Surg ICU	5/17/2023 0:00	13	15.0	12.8	-2.2
Med- Surg ICU	5/17/2023 1:00	13	15.0	12.8	-2.2
Med- Surg ICU	5/17/2023 2:00	13	15.0	12.8	-2.2
Med- Surg ICU	5/17/2023 3:00	13	15.0	12.8	-2.2
Med- Surg ICU	5/17/2023 4:00	13	15.0	12.8	-2.2
Med- Surg ICU	5/17/2023 5:00	13	15.0	12.8	-2.2
Med- Surg ICU	5/17/2023 6:00	13	15.0	12.8	-2.2
Med- Surg ICU	5/17/2023 7:00	13	15.0	12.8	-2.2
Med- Surg ICU	5/17/2023 8:00	13	14.0	12.5	-1.6
Med- Surg ICU	5/17/2023 9:00	13	14.0	12.5	-1.6
Med- Surg ICU	5/17/2023 10:00	13	14.0	12.5	-1.6

Med- Surg ICU	5/17/2023 11:00	13	14.0	12.5	-1.6
Med- Surg ICU	5/17/2023 12:00	13	14.0	12.5	-1.6
Med- Surg ICU	5/17/2023 13:00	13	14.0	12.5	-1.6
Med- Surg ICU	5/17/2023 14:00	13	14.0	12.5	-1.6
Med- Surg ICU	5/17/2023 15:00	13	14.0	11.5	-2.5
Med- Surg ICU	5/17/2023 16:00	13	14.0	10.6	-3.4
Med- Surg ICU	5/17/2023 17:00	13	14.0	10.6	-3.4
Med- Surg ICU	5/17/2023 18:00	13	14.0	10.6	-3.4
Med- Surg ICU	5/17/2023 19:00	12	15.0	10.6	-4.4
Med- Surg ICU	5/17/2023 20:00	12	15.0	12.8	-2.2
Med- Surg ICU	5/17/2023 21:00	12	15.0	12.8	-2.2
Med- Surg ICU	5/17/2023 22:00	11	15.0	12.8	-2.2
Med- Surg ICU	5/17/2023 23:00	11	15.0	12.8	-2.2
Med- Surg ICU	5/18/2023 0:00	11	15.0	11.5	-3.5
Med- Surg ICU	5/18/2023 1:00	11	15.0	11.5	-3.5
Med- Surg ICU	5/18/2023 2:00	11	15.0	11.5	-3.5
Med- Surg ICU	5/18/2023 3:00	11	15.0	11.5	-3.5
Med- Surg ICU	5/18/2023 4:00	11	15.0	11.5	-3.5
Med- Surg ICU	5/18/2023 5:00	11	15.0	11.5	-3.5
Med- Surg ICU	5/18/2023 6:00	11	15.0	11.5	-3.5
Med- Surg ICU	5/18/2023 7:00	11	15.0	11.5	-3.5
Med- Surg ICU	5/18/2023 8:00	11	15.0	10.0	-5.0
Med- Surg ICU	5/18/2023 9:00	11	15.0	10.0	-5.0
Med- Surg ICU	5/18/2023 10:00	12	14.0	10.0	-4.0
Med- Surg ICU	5/18/2023 11:00	11	14.0	8.7	-5.3
Med- Surg ICU	5/18/2023 12:00	11	14.0	8.7	-5.3
Med- Surg ICU	5/18/2023 13:00	11	14.0	8.7	-5.3
Med- Surg ICU	5/18/2023 14:00	11	14.0	8.7	-5.3
Med- Surg ICU	5/18/2023 15:00	11	14.0	8.7	-5.3
Med- Surg ICU	5/18/2023 16:00	11	14.0	9.0	-5.0
Med- Surg ICU	5/18/2023 17:00	11	14.0	9.0	-5.0
Med- Surg ICU	5/18/2023 18:00	11	14.0	9.0	-5.0
Med- Surg ICU	5/18/2023 19:00	11	14.0	9.0	-5.0
Med- Surg ICU	5/18/2023 20:00	11	14.0	10.3	-3.7
Med- Surg ICU	5/18/2023 21:00	11	14.0	10.3	-3.7
Med- Surg ICU	5/18/2023 22:00	11	14.0	10.3	-3.7
Med- Surg ICU	5/18/2023 23:00	11	15.0	9.0	-6.0
Med- Surg ICU	5/19/2023 0:00	11	15.0	10.3	-4.7
Med- Surg ICU	5/19/2023 1:00	11	15.0	10.3	-4.7
Med- Surg ICU	5/19/2023 2:00	11	15.0	10.3	-4.7
Med- Surg ICU	5/19/2023 3:00	11	15.0	10.3	-4.7
Med- Surg ICU	5/19/2023 4:00	11	15.0	10.3	-4.7
Med- Surg ICU	5/19/2023 5:00	11	15.0	10.3	-4.7
Med- Surg ICU	5/19/2023 6:00	11	15.0	10.3	-4.7
Med- Surg ICU	5/19/2023 7:00	11	14.0	10.3	-3.7
Med- Surg ICU	5/19/2023 8:00	11	14.0	8.7	-5.3
Med- Surg ICU	5/19/2023 9:00	11	14.0	8.7	-5.3

Med- Surg ICU	5/19/2023 10:00	11	14.0	8.7	-5.3
Med- Surg ICU	5/19/2023 11:00	11	14.0	8.7	-5.3
Med- Surg ICU	5/19/2023 12:00	11	14.0	8.7	-5.3
Med- Surg ICU	5/19/2023 13:00	11	14.0	8.7	-5.3
Med- Surg ICU	5/19/2023 14:00	11	14.0	8.7	-5.3
Med- Surg ICU	5/19/2023 15:00	14	14.0	8.7	-5.3
Med- Surg ICU	5/19/2023 16:00	14	14.0	6.4	-7.6
Med- Surg ICU	5/19/2023 17:00	14	14.0	6.4	-7.6
Med- Surg ICU	5/19/2023 18:00	14	14.0	6.4	-7.6
Med- Surg ICU	5/19/2023 19:00	14	14.0	6.4	-7.6
Med- Surg ICU	5/19/2023 20:00	14	14.0	11.5	-2.5
Med- Surg ICU	5/19/2023 21:00	13	14.0	11.5	-2.5
Med- Surg ICU	5/19/2023 22:00	13	14.0	11.5	-2.5
Med- Surg ICU	5/19/2023 23:00	13	14.0	11.5	-2.5
Med- Surg ICU	5/20/2023 0:00	13	14.0	11.5	-2.5
Med- Surg ICU	5/20/2023 1:00	13	14.0	11.5	-2.5
Med- Surg ICU	5/20/2023 2:00	13	14.0	11.5	-2.5
Med- Surg ICU	5/20/2023 3:00	13	14.0	11.5	-2.5
Med- Surg ICU	5/20/2023 4:00	13	14.0	11.5	-2.5
Med- Surg ICU	5/20/2023 5:00	13	14.0	11.5	-2.5
Med- Surg ICU	5/20/2023 6:00	13	14.0	11.5	-2.5
Med- Surg ICU	5/20/2023 7:00	13	14.0	11.5	-2.5
Med- Surg ICU	5/20/2023 8:00	13	14.0	7.5	-6.5
Med- Surg ICU	5/20/2023 9:00	13	14.0	7.5	-6.5
Med- Surg ICU	5/20/2023 10:00	13	14.0	7.5	-6.5
Med- Surg ICU	5/20/2023 11:00	13	14.0	7.5	-6.5
Med- Surg ICU	5/20/2023 12:00	13	14.0	8.7	-5.3
Med- Surg ICU	5/20/2023 13:00	13	14.0	8.7	-5.3
Med- Surg ICU	5/20/2023 14:00	13	14.0	8.7	-5.3
Med- Surg ICU	5/20/2023 15:00	13	14.0	8.7	-5.3
Med- Surg ICU	5/20/2023 16:00	13	14.0	10.3	-3.7
Med- Surg ICU	5/20/2023 17:00	13	14.0	10.3	-3.7
Med- Surg ICU	5/20/2023 18:00	13	14.0	10.3	-3.7
Med- Surg ICU	5/20/2023 19:00	13	14.0	9.6	-4.4
Med- Surg ICU	5/20/2023 20:00	10	14.0	9.0	-5.0
Med- Surg ICU	5/20/2023 21:00	10	14.0	9.0	-5.0
Med- Surg ICU	5/20/2023 22:00	10	14.0	9.0	-5.0
Med- Surg ICU	5/20/2023 23:00	10	14.0	9.0	-5.0
Med- Surg ICU	5/21/2023 0:00	10	14.0	7.7	-6.3
Med- Surg ICU	5/21/2023 1:00	10	14.0	7.7	-6.3
Med- Surg ICU	5/21/2023 2:00	10	14.0	7.7	-6.3
Med- Surg ICU	5/21/2023 3:00	10	14.0	7.7	-6.3
Med- Surg ICU	5/21/2023 4:00	10	14.0	7.7	-6.3
Med- Surg ICU	5/21/2023 5:00	10	14.0	7.7	-6.3
Med- Surg ICU	5/21/2023 6:00	10	14.0	7.7	-6.3
Med- Surg ICU	5/21/2023 7:00	10	14.0	7.7	-6.3
Med- Surg ICU	5/21/2023 8:00	10	14.0	8.7	-5.3

Med- Surg ICU	5/21/2023 9:00	10	14.0	8.7	-5.3
Med- Surg ICU	5/21/2023 10:00	10	14.0	8.7	-5.3
Med- Surg ICU	5/21/2023 11:00	11	12.0	8.7	-3.3
Med- Surg ICU	5/21/2023 12:00	11	12.0	8.7	-3.3
Med- Surg ICU	5/21/2023 13:00	11	12.0	8.7	-3.3
Med- Surg ICU	5/21/2023 14:00	11	12.0	8.7	-3.3
Med- Surg ICU	5/21/2023 15:00	11	12.0	8.7	-3.3
Med- Surg ICU	5/21/2023 16:00	11	12.0	9.0	-3.0
Med- Surg ICU	5/21/2023 17:00	11	12.0	9.0	-3.0
Med- Surg ICU	5/21/2023 18:00	11	12.0	9.0	-3.0
Med- Surg ICU	5/21/2023 19:00	11	12.0	9.0	-3.0
Med- Surg ICU	5/21/2023 20:00	11	14.0	9.0	-5.0
Med- Surg ICU	5/21/2023 21:00	11	14.0	9.0	-5.0
Med- Surg ICU	5/21/2023 22:00	11	14.0	9.0	-5.0
Med- Surg ICU	5/21/2023 23:00	11	14.0	9.0	-5.0
Med- Surg ICU	5/22/2023 0:00	11	14.0	9.0	-5.0
Med- Surg ICU	5/22/2023 1:00	11	14.0	9.0	-5.0
Med- Surg ICU	5/22/2023 2:00	11	14.0	9.0	-5.0
Med- Surg ICU	5/22/2023 3:00	11	14.0	9.0	-5.0
Med- Surg ICU	5/22/2023 4:00	11	14.0	9.0	-5.0
Med- Surg ICU	5/22/2023 5:00	11	14.0	9.0	-5.0
Med- Surg ICU	5/22/2023 6:00	11	14.0	9.0	-5.0
Med- Surg ICU	5/22/2023 7:00	11	14.0	9.0	-5.0
Med- Surg ICU	5/22/2023 8:00	11	14.0	5.9	-8.1
Med- Surg ICU	5/22/2023 9:00	11	14.0	5.9	-8.1
Med- Surg ICU	5/22/2023 10:00	11	14.0	5.9	-8.1
Med- Surg ICU	5/22/2023 11:00	12	12.0	7.3	-4.7
Med- Surg ICU	5/22/2023 12:00	12	12.0	7.3	-4.7
Med- Surg ICU	5/22/2023 13:00	12	12.0	7.3	-4.7
Med- Surg ICU	5/22/2023 14:00	12	12.0	7.3	-4.7
Med- Surg ICU	5/22/2023 15:00	12	12.0	7.3	-4.7
Med- Surg ICU	5/22/2023 16:00	12	12.0	4.5	-7.5
Med- Surg ICU	5/22/2023 17:00	12	12.0	4.5	-7.5
Med- Surg ICU	5/22/2023 18:00	12	12.0	4.5	-7.5
Med- Surg ICU	5/22/2023 19:00	12	12.0	4.5	-7.5
Med- Surg ICU	5/22/2023 20:00	12	12.0	12.1	0.1
Med- Surg ICU	5/22/2023 21:00	12	12.0	12.1	0.1
Med- Surg ICU	5/22/2023 22:00	12	12.0	12.1	0.1
Med- Surg ICU	5/22/2023 23:00	12	12.0	12.1	0.1
Med- Surg ICU	5/23/2023 0:00	12	12.0	10.3	-1.7
Med- Surg ICU	5/23/2023 1:00	12	12.0	10.3	-1.7
Med- Surg ICU	5/23/2023 2:00	12	12.0	10.3	-1.7
Med- Surg ICU	5/23/2023 3:00	12	12.0	10.3	-1.7
Med- Surg ICU	5/23/2023 4:00	12	12.0	10.3	-1.7
Med- Surg ICU	5/23/2023 5:00	12	12.0	10.3	-1.7
Med- Surg ICU	5/23/2023 6:00	12	12.0	10.3	-1.7
Med- Surg ICU	5/23/2023 7:00	12	12.0	10.3	-1.7

Med- Surg ICU	5/23/2023 8:00	12	12.0	12.5	0.5
Med- Surg ICU	5/23/2023 9:00	12	12.0	12.5	0.5
Med- Surg ICU	5/23/2023 10:00	12	12.0	12.5	0.5
Med- Surg ICU	5/23/2023 11:00	12	12.0	12.5	0.5
Med- Surg ICU	5/23/2023 12:00	12	12.0	12.5	0.5
Med- Surg ICU	5/23/2023 13:00	12	12.0	12.5	0.5
Med- Surg ICU	5/23/2023 14:00	12	12.0	10.0	-2.0
Med- Surg ICU	5/23/2023 15:00	12	12.0	10.0	-2.0
Med- Surg ICU	5/23/2023 16:00	12	12.0	9.0	-3.0
Med- Surg ICU	5/23/2023 17:00	12	12.0	9.0	-3.0
Med- Surg ICU	5/23/2023 18:00	12	12.0	9.0	-3.0
Med- Surg ICU	5/23/2023 19:00	12	12.0	9.0	-3.0
Med- Surg ICU	5/23/2023 20:00	12	12.0	9.0	-3.0
Med- Surg ICU	5/23/2023 21:00	12	12.0	9.0	-3.0
Med- Surg ICU	5/23/2023 22:00	12	12.0	10.3	-1.7
Med- Surg ICU	5/23/2023 23:00	12	12.0	10.3	-1.7
Med- Surg ICU	5/24/2023 0:00	12	12.0	10.3	-1.7
Med- Surg ICU	5/24/2023 1:00	12	12.0	10.3	-1.7
Med- Surg ICU	5/24/2023 2:00	12	12.0	10.3	-1.7
Med- Surg ICU	5/24/2023 3:00	12	12.0	10.3	-1.7
Med- Surg ICU	5/24/2023 4:00	12	13.0	10.3	-2.7
Med- Surg ICU	5/24/2023 5:00	12	13.0	10.3	-2.7
Med- Surg ICU	5/24/2023 6:00	12	13.0	10.3	-2.7
Med- Surg ICU	5/24/2023 7:00	12	13.0	10.3	-2.7
Med- Surg ICU	5/24/2023 8:00	12	13.0	11.2	-1.8
Med- Surg ICU	5/24/2023 9:00	15	15.0	11.2	-3.8
Med- Surg ICU	5/24/2023 10:00	15	15.0	11.2	-3.8
Med- Surg ICU	5/24/2023 11:00	15	15.0	11.2	-3.8
Med- Surg ICU	5/24/2023 12:00	15	15.0	11.2	-3.8
Med- Surg ICU	5/24/2023 13:00	15	15.0	11.2	-3.8
Med- Surg ICU	5/24/2023 14:00	15	15.0	11.2	-3.8
Med- Surg ICU	5/24/2023 15:00	15	15.0	11.2	-3.8
Med- Surg ICU	5/24/2023 16:00	14	15.0	10.6	-4.4
Med- Surg ICU	5/24/2023 17:00	14	15.0	9.6	-5.4
Med- Surg ICU	5/24/2023 18:00	14	15.0	10.6	-4.4
Med- Surg ICU	5/24/2023 19:00	14	15.0	10.6	-4.4
Med- Surg ICU	5/24/2023 20:00	14	15.0	11.5	-3.5
Med- Surg ICU	5/24/2023 21:00	14	15.0	11.5	-3.5
Med- Surg ICU	5/24/2023 22:00	12	15.0	11.5	-3.5
Med- Surg ICU	5/24/2023 23:00	12	15.0	11.5	-3.5
Med- Surg ICU	5/25/2023 0:00	12	15.0	11.5	-3.5
Med- Surg ICU	5/25/2023 1:00	12	15.0	11.5	-3.5
Med- Surg ICU	5/25/2023 2:00	12	15.0	11.5	-3.5
Med- Surg ICU	5/25/2023 3:00	12	15.0	11.5	-3.5
Med- Surg ICU	5/25/2023 4:00	12	15.0	11.5	-3.5
Med- Surg ICU	5/25/2023 5:00	12	15.0	11.5	-3.5
Med- Surg ICU	5/25/2023 6:00	12	15.0	11.5	-3.5

Med- Surg ICU	5/25/2023 7:00	12	15.0	11.5	-3.5
Med- Surg ICU	5/25/2023 8:00	12	12.0	10.0	-2.0
Med- Surg ICU	5/25/2023 9:00	12	12.0	10.0	-2.0
Med- Surg ICU	5/25/2023 10:00	12	12.0	10.0	-2.0
Med- Surg ICU	5/25/2023 11:00	12	15.0	10.0	-5.0
Med- Surg ICU	5/25/2023 12:00	12	15.0	10.0	-5.0
Med- Surg ICU	5/25/2023 13:00	12	15.0	10.0	-5.0
Med- Surg ICU	5/25/2023 14:00	12	15.0	10.0	-5.0
Med- Surg ICU	5/25/2023 15:00	12	15.0	10.0	-5.0
Med- Surg ICU	5/25/2023 16:00	12	15.0	10.3	-4.7
Med- Surg ICU	5/25/2023 17:00	12	15.0	10.3	-4.7
Med- Surg ICU	5/25/2023 18:00	12	15.0	10.3	-4.7
Med- Surg ICU	5/25/2023 19:00	12	15.0	10.3	-4.7
Med- Surg ICU	5/25/2023 20:00	12	15.0	10.3	-4.7
Med- Surg ICU	5/25/2023 21:00	12	15.0	10.3	-4.7
Med- Surg ICU	5/25/2023 22:00	12	15.0	10.3	-4.7
Med- Surg ICU	5/25/2023 23:00	12	15.0	10.3	-4.7

Med- Surg ICU = Medical Surgical Intensive Care Unit; ICU = Intensive Care Unit. The "Difference" column compares the P/N ratio and the Dynamic Bed Count, where a negative value indicates that the unit should have less patient capacity while a positive value indicates the unit could have taken on additional patients. The amplitude of the value defines how many less (negative value) or more (positive value) patients the unit could have handled during any given hour.
